# Proteomic and Transcriptomic Changes in Hibernating Grizzly Bears Reveal Metabolic and Signaling Pathways that Protect against Muscle Atrophy

**DOI:** 10.1038/s41598-019-56007-8

**Published:** 2019-12-27

**Authors:** D. A. Mugahid, T. G. Sengul, X. You, Y. Wang, L. Steil, N. Bergmann, M. H. Radke, A. Ofenbauer, M. Gesell-Salazar, A. Balogh, S. Kempa, B. Tursun, C. T. Robbins, U. Völker, W. Chen, L. Nelson, M. Gotthardt

**Affiliations:** 1 Neuromuscular and Cardiovascular Cell Biology, Max Delbrück Center for Molecular Medicine, Berlin, Germany; 2 Berlin Institute for Medical Systems Biology, Max Delbrück Center for Molecular Medicine, Berlin, Germany; 3 Interfaculty Institute for Genetics and Functional Genomics, University Medicine Greifswald, Greifswald, Germany; 4 College of Veterinary Medicine and Department of Veterinary Clinical Science, Washington State University, Pullman, Washington USA; 5 School of the Environment and School of Biological Sciences, Washington State University, Pullman, Washington USA; 6 DZHK (German Centre for Cardiovascular Research), partner site Greifswald, Greifswald, Germany; 7 Experimental and Clinical Research Center, Charité & Max Delbrück Center for Molecular Medicine, Berlin, Germany; 8 Charité Universitätsmedizin Berlin, Berlin, Germany; 9 DZHK (German Center for Cardiovascular Research), partner site Berlin, Berlin, Germany

**Keywords:** Translational research, Experimental models of disease, Molecular medicine, Skeletal muscle, Cell signalling, Mechanisms of disease, Systems biology, Metabolism, Proteome

## Abstract

Muscle atrophy is a physiological response to disuse and malnutrition, but hibernating bears are largely resistant to this phenomenon. Unlike other mammals, they efficiently reabsorb amino acids from urine, periodically activate muscle contraction, and their adipocytes differentially responds to insulin. The contribution of myocytes to the reduced atrophy remains largely unknown. Here we show how metabolism and atrophy signaling are regulated in skeletal muscle of hibernating grizzly bear. Metabolic modeling of proteomic changes suggests an autonomous increase of non-essential amino acids (NEAA) in muscle and treatment of differentiated myoblasts with NEAA is sufficient to induce hypertrophy. Our comparison of gene expression in hibernation versus muscle atrophy identified several genes differentially regulated during hibernation, including Pdk4 and Serpinf1. Their trophic effects extend to myoblasts from non-hibernating species (including C. elegans), as documented by a knockdown approach. Together, these changes reflect evolutionary favored adaptations that, once translated to the clinics, could help improve atrophy treatment.

## Introduction

Muscle wasting accompanies a range of human conditions such as aging, prolonged bed rest, space flight, malnutrition and cancer^1,2^. A severe form is disuse atrophy of the diaphragm that sets in within hours after patients are put on life support, and interferes with attempts to wean them off mechanical ventilation^3^. Therapeutic strategies that reduce the loss of muscle mass and help regain full muscle function after periods of inactivity or in association with disease can improve outcome and reduce time of hospitalization^4^. Several cellular processes and signaling pathways have been associated with the regulation of muscle mass in animal models, including ubiquitination via the E3 ligases MuRF1 and MAFbx^5^, proteolysis^6^, autophagy^7^, and signaling via the Igf-1-Akt-mTor-^1^, NFκB-^8^, HDAC^9^, and Bmp-pathways^10^.

Nature presents us with examples of species that are largely resistant to atrophy^11^, such as the hibernating squirrel^12^ or grizzly bear (*Ursus arctos horribilis*). Studying how these animals preserve muscle mass despite the challenges of hibernation could teach us how to preserve muscle mass in humans. Contrary to humans and mice, bears lose far less muscle mass and strength during hibernation, a period of 5 to 7 months of restricted caloric intake and immobilization, sufficient to cause almost twice as much muscle loss in bed-ridden or malnourished humans^13–16^. The underlying systemic adaptations include the increased ability to utilize the fat reserves accumulated before hibernation and to maintain blood glucose levels constant despite caloric restriction^17,18^. Furthermore, hibernating bears efficiently reabsorb urea from their urine, which decreases the need to mobilize amino-acids from muscle protein^19^. Finally, periodic neural stimulation of muscles in the form of shivers during hibernation has been proposed to help conserve muscle mass. However, hibernating bears are resistant to muscle atrophy even after denervation^13^, suggesting that intramuscular changes contribute to atrophy resistance. Accordingly, we interrogated the skeletal muscle proteome and transcriptome to reveal the molecular changes that limit atrophy in hibernating bears and evaluated the conservation of these pathways between species as well as their potential as therapeutic targets using an *in vivo* knockdown approach in C. elegans and a murine cell-based atrophy model.

## Results

### Proteomic and transcriptomic changes in skeletal muscle during hibernation relate to metabolism and growth signaling

To identify genes that protect grizzly muscle from atrophy and understand the underlying changes in metabolism and cell signaling, we used a complementary proteomics and transcriptomics approach. We obtained gastrocnemius (GA) muscle biopsies from two cubs and two older grizzly bears before (October) and during hibernation (February) and isolated total protein for analysis by mass spectrometry.

**Figure 1:**
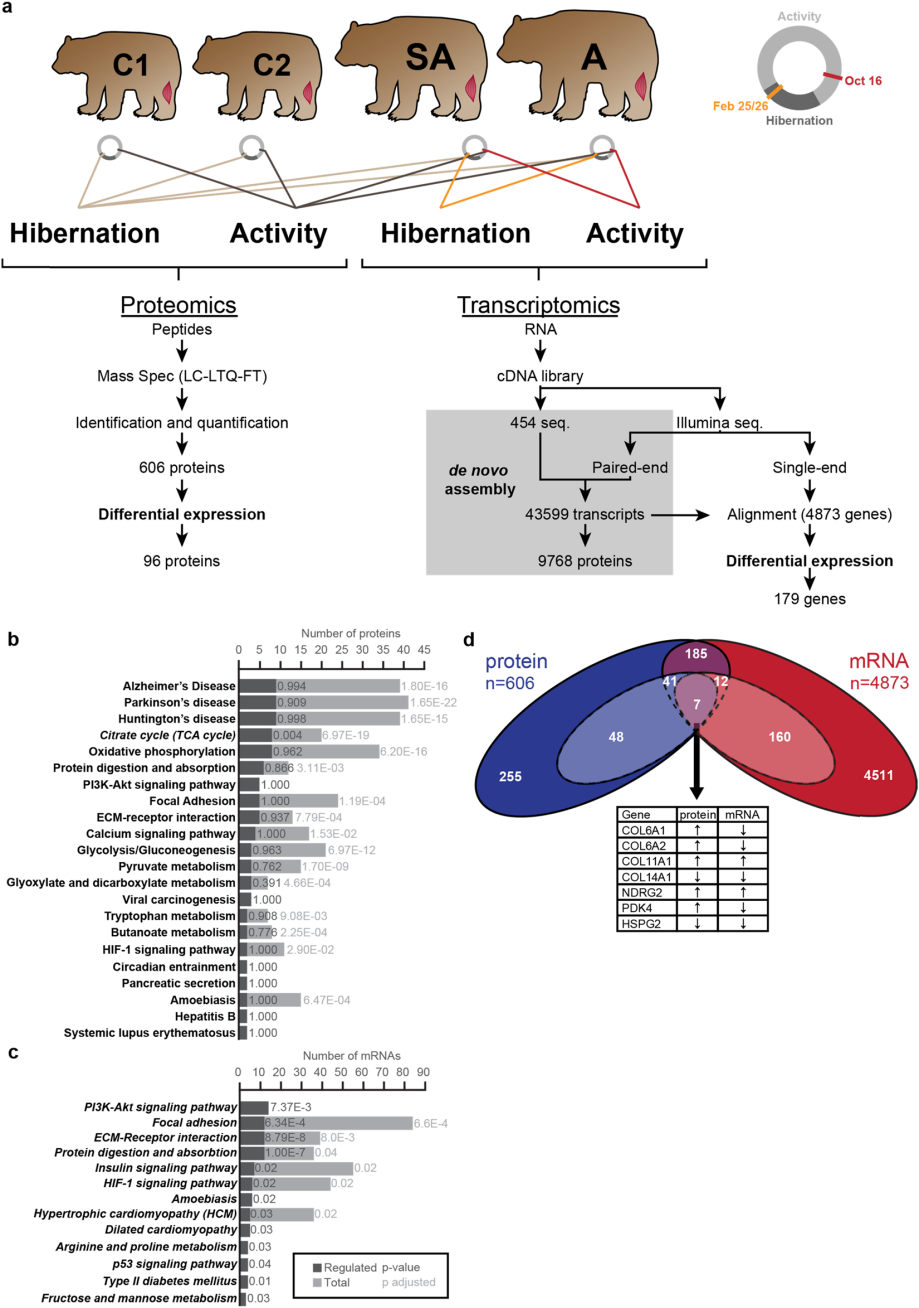
Identification and annotation of proteins and transcripts regulated in skeletal muscle of *U. arctos* during hibernation. (**a**) Experimental layout and data processing. (**b**, **c**) KEGG pathway analysis of proteins and transcripts identified in gastrocnemius muscle (GA) with total identified species (light grey) and regulated genes (dark grey). (**d**) Overlap (purple) between the identified proteins (blue) and mRNAs (red). Regulated species are indicated in lighter colors with 7 genes regulated in both datasets (table). C, cub; SA, subadult; A, adult. See also Fig. S1. Adapted from thesis by D.M.^66^.

As an annotated grizzly proteome is not available, we identified peptides by homology to the human proteome. A total of 606 unique proteins were identified, of which 96 were regulated during hibernation irrespective of age (two-way ANOVA, *p* < 0.05; Fig. 1a; Supplementary Table S1). A KEGG enrichment analysis revealed a bias for the identification of proteins related to distinct metabolic pathways, with a considerable number being enzymes of the TCA cycle (Fig. 1b; Supplementary Table S3).

To expand the range of biological processes we can address, we used matching biopsies from the adult and sub- adult to generate RNA-seq data. After *de novo* transcriptome assembly by homology to human transcripts, we quantified reads mapping to 4873 annotated genes (Fig. 1a; Supplementary Table S2). These genes associate with a diverse set of KEGG pathways (Fig. 1c, Supplementary Table S3). Differential gene expression analysis using NOIseq identified 208 genes regulated in hibernation (Supplementary Table S2, predictive-score ≥ 0.8). A KEGG enrichment analysis correcting for identification bias revealed changes in more biological processes than the proteomic data with little overlap between the two data sets. We attribute the limited overlap to the relatively lower coverage of the proteomics data (Fig. 1D). While the majority of the regulated proteins are metabolic enzymes, the majority of the regulated transcripts are associated with signal transduction through the Pi3k-Akt pathway (Fig. 1c; Suppl. Table 3), which plays a pivotal role in regulating organ growth and metabolism across species^20–23^. Changes on the transcript level were mapped to the corresponding human Pi3k-Akt KEGG pathway (Supplementary Fig. S1) and suggest an overall increase in pathway activity: The insulin-sensitive insulin receptor substrate Irs-1 is upregulated during hibernation, while the less insulin-sensitive homologue Irs-2^24^ is suppressed. This is accompanied by increased levels of its downstream effector Ip3r3, and decreased levels of depTOR - an inhibitor of the Irs downstream effector mTor. In a published set of insulin-sensitive individuals^25^ IRS1, PIK3R3 and FAS were similarly regulated, suggesting that insulin-sensitivity is increased in skeletal muscle during hibernation. Consistently, transcripts of the Irs-binding protein Grb2, as well as Angpt4 (upstream of Irs- signaling) were increased. An additional component of this signaling pathway is Sgk1, which is upregulated to protect hibernating squirrels from muscle atrophy^12^. In our dataset, Sgk1 was below the detection limit of RNAseq or MS analysis. Another set of enriched genes are frequently mutated in cardiomyopathies. Many of these are structural proteins of the sarcomere and their upregulation at the mRNA level would be consistent with increased hypertrophy and reduced atrophy signaling during hibernation.

### Proteomic changes and metabolic modeling predict an increase in non-essential amino acid levels (NEAA) in hibernating bear muscle and a decrease in aging humans

**Figure 2:**
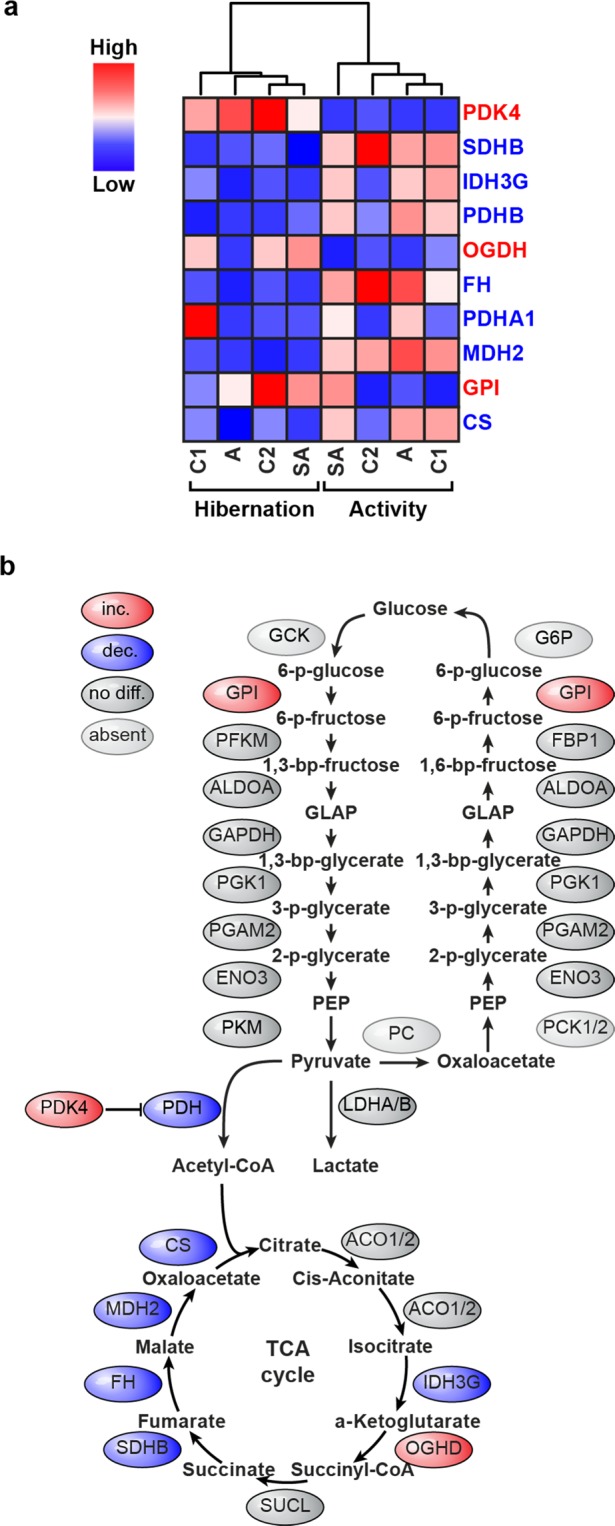
Enzymes related to glucose metabolism are regulated at the protein level in skeletal muscle during hibernation. (**a**) Clustering of samples based on enzymes related to central glucose metabolism segregates hibernation and activity irrespective of age (*p*-value < 0.05, two-way ANOVA). (**b**) Pathway of central glucose metabolism overlaid with hibernation-associated changes in proteins depicted in (**a**). Proteins in red are increased (inc) during hibernation, proteins in blue are decreased (dec). Adapted from thesis by D.M.^66^.

**Figure 3:**
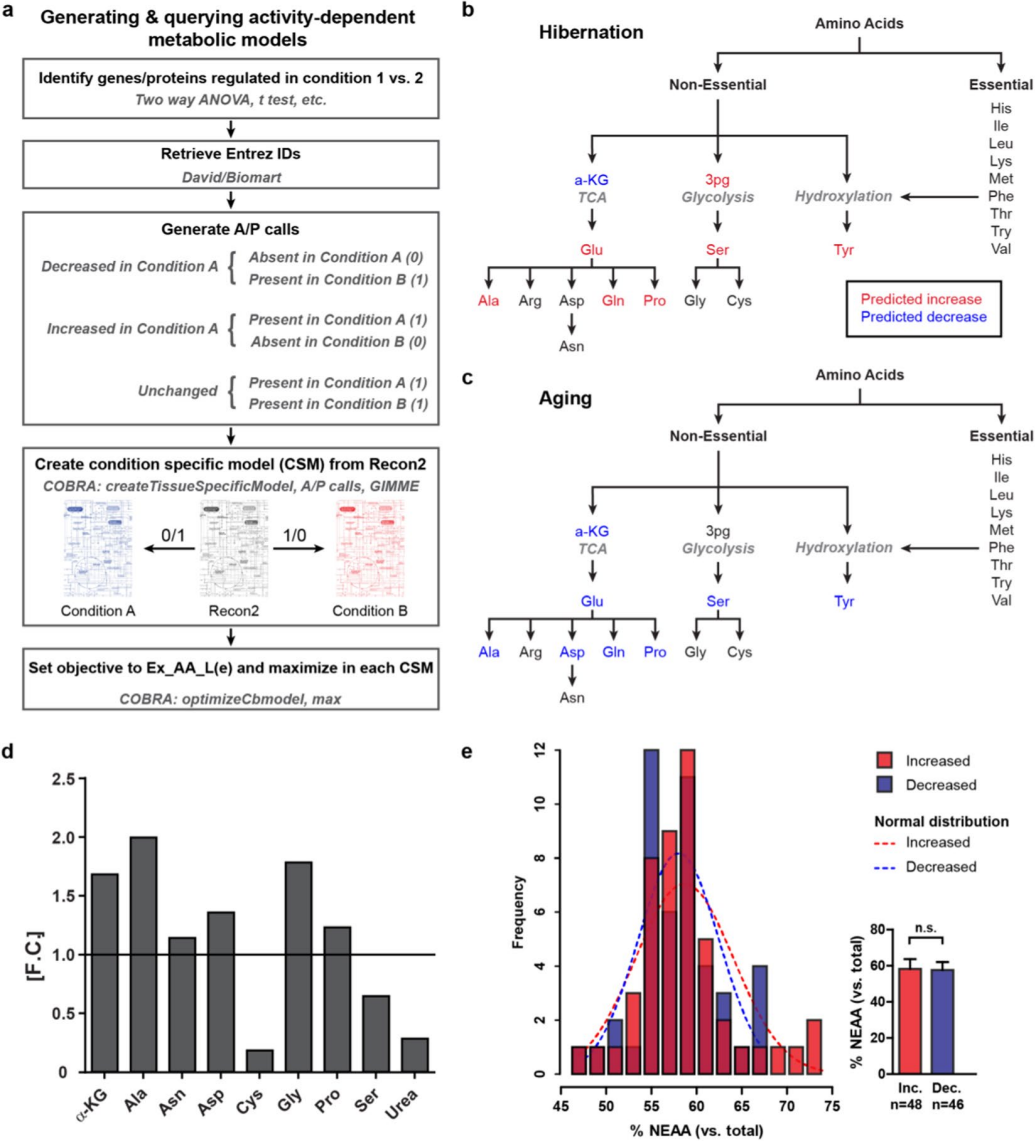
Metabolic modeling predicts differential regulation of non-essential amino acid (NEAA) levels in hibernation versus human ageing. (**a**) Generation of activity-dependent metabolic models. (**b**) Predicted changes in steady state levels of NEAAs and their main precursors in GA during hibernation. (**c**) Prediction of age-dependent changes in NEAA metabolism in human vastus lateralis muscle. See also Fig. S2. (**d**) Average changes in NEAAs and their TCA cycle precursors during hibernation as determined by GC-MS (*n* = 3). (**e**) The distribution of NEAA content in proteins with increased expression in hibernation (red; inc.) or decreased expression (blue; dec.). n.s. – not significant. Adapted from thesis by D.M.^66^.

Changes in glucose metabolic enzymes were the most prominent on the protein level and resulted in the segregation of bear samples into two main groups active and hibernating upon unsupervised clustering (two-way ANOVA; Fig. 2a). These changes are largely within the tricarbocylic acid (TCA) cycle and extend to glycolysis/gluconeogenesis (Fig. 2b). The decrease in the alpha and beta subunits of pyruvate dehydrogenase (Pdh), which produces acetyl-CoA and the increased levels of its inhibitor pyruvate dehydrogenase kinase 4 (Pdk4), suggests a decrease in the production of the TCA cycle substrate acetyl-CoA. This is accompanied by a decrease in the majority of TCA cycle enzymes, which process acetyl-CoA confirming prior art^26^. In addition to providing energy equivalents, glycolysis-, gluconeogenesis- and TCA cycle intermediates serve as precursors for NEAA synthesis and the availability of NEAAs directly affects the ability of muscle to maintain protein levels. Thus, we modeled the effect of hibernation-induced changes in protein expression on the NEAA level. Based on the relative changes in muscle protein levels, we constructed two activity-dependent metabolic models building on the human metabolic network Recon2^27^. We then optimized for maximum NEAA production as well as for that of their precursors, glycerate-3-phosphate (3 pg) and alpha-ketogluterate (a-KG). The relative changes in steady state levels of NEAA during hibernation (Fig. 3) indicate an increase in six of 11 NEAAs. We used the identical approach to learn how NEAAs and their precursors are affected in aging human muscle as an example of a physiological atrophy response to reduced activity and calorie intake. The choice of aging humans as a reference was based on the similarity in physiological changes as compared to hibernating grizzly bears (reduced food consumption with unchanged blood glucose levels and reduced activity), which nevertheless lead to less muscle wasting in the latter. To build the age-dependent models we used publicly available transcriptional data from skeletal muscle of young and older women (GSE674)^28^.

A KEGG enrichment analysis illustrated the age-dependent regulation of genes associated with a large number of metabolic pathways (Supplementary Fig. S2; Supplementary Table S3), indicating that metabolic modeling is appropriate for contextualizing such data. We constructed and queried two models that reflect metabolic changes in the young and elderly female human muscle biopsies, which predicted a decrease in the levels of seven NEAA in aging muscle, including all those increased during hibernation (Fig. 3c). Alpha-ketogluterate, the TCA cycle-derived precursor was also decreased while glycerate-3-phosphate remained unchanged. Profiling of metabolite levels by mass spectrometry on matching muscle samples from hibernation and activity supports an increase in most of the non-essential amino acids that were detected during hibernation (Ala, Asn, Asp Gly, Pro; Fig. 3d) – with cysteine and serine, both glycolysis-derived NEAAs, as notable exceptions. Levels of α-KG were increased during hibernation while urea levels were decreased. Together this supports the hypothesis that changes in metabolism during hibernation improve the availability of NEAAs, particularly of those derived from the TCA cycle.

To determine whether the differential availability of NEAAs during hibernation resulted in the differential synthesis of NEAA-rich proteins, we combined expression data and sequence information of proteins regulated in hibernation and found a minor increase in NEAA-rich proteins (>65% NEAA) during hibernation (Fig. 3e) and no difference in the distribution of total NEAAs in the increased and decreased protein groups. These data suggest that the increase in NEAA availability does not significantly affect the synthesis of NEAA-rich proteins.

### NEAA supplementation decreases atrophy in C2C12 cells

**Figure 4:**
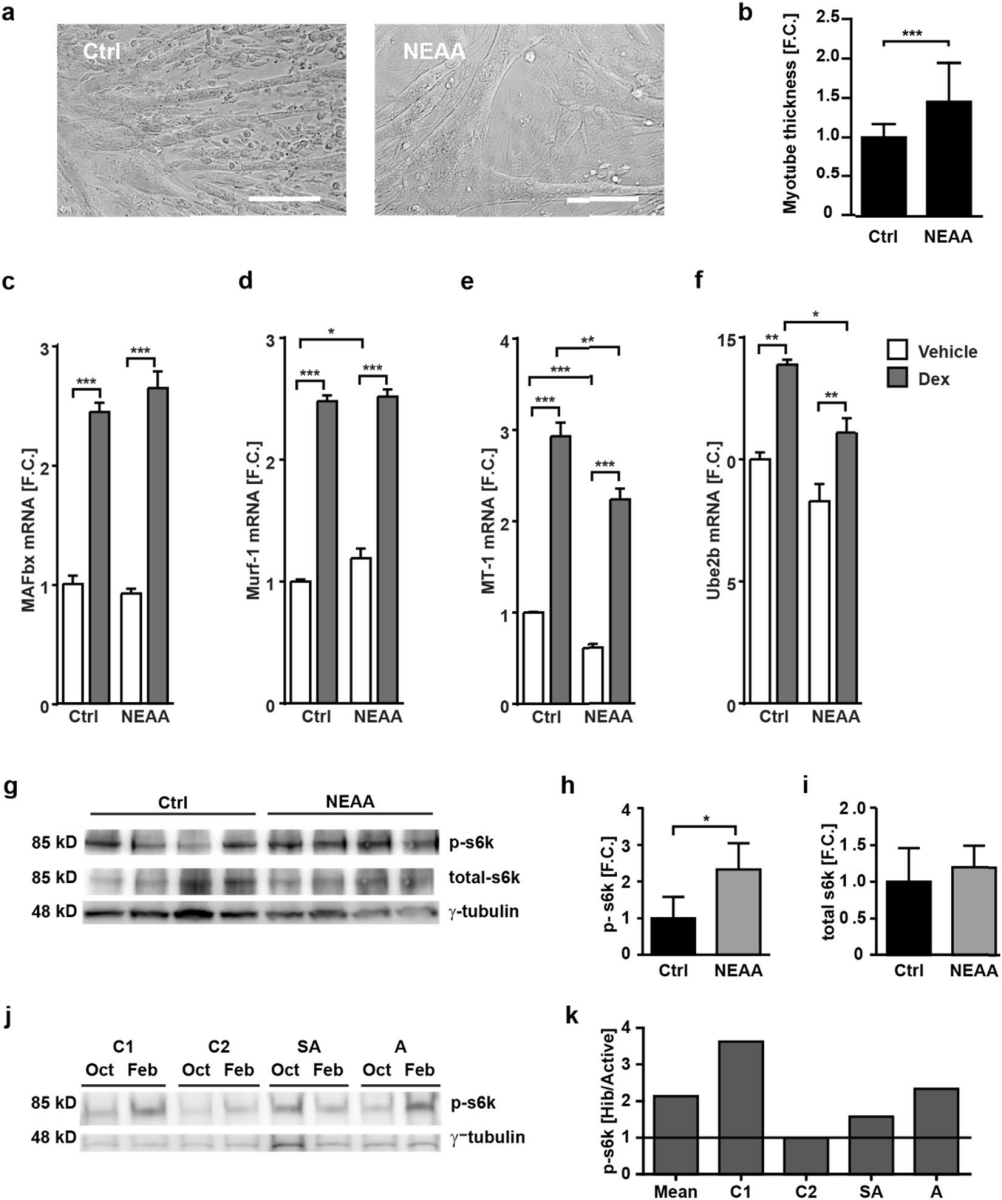
Effect of NEAAs on myotube thickness and trophic signaling. (**a**,**b**) NEAA supplementation at 10x the levels of basic DMEM significantly increases myotube diameter (mean ± s.d., t-test). Size bar; 50 μM. (**c–f**) Effect of NEAA supplementation on MAFbx, MT-1, Murf1, and Ube2b in differentiated C2C12 cells. Comparison of vehicle for normal growth and dexamethasone treatment for induction of atrophy (*n* = 6, mean ± s.d., two-way ANOVA, Bonferroni post-hoc test). (**g**) Phospho- and total S6k protein levels and S6k phosphorylation after NEAA treatment, quantified in (**h**) and (**i**), respectively (*n* = 4, mean + s.d., t-test). (**j**) Phospho-S6k levels in hibernating versus active grizzly bears, with the ratios quantified in (**k**). Ctrl, control; Dex, Dexamethasone; NEAA, non-essential amino acids. *p < 0.05, **p < 0.01, ***p < 0.001. Adapted from thesis by D.M^.66^.

To evaluate if an increase in NEAAs affects myotube size, we quantified changes in the diameter of differentiated myotubes derived from C2C12 cells and the expression of muscle atrophy markers. After treatment with NEAA fortified DMEM (10x elevated NEAA levels), myotubes were larger than controls cultured in DMEM with the standard NEAA content (Fig. 4a,b). As a cell-based model for muscle atrophy we used differentiated C2C12 cells treated with the synthetic glucocorticoid dexamethasone (Dex) or DMSO (vehicle). Dex-treatment led to an increased expression of atrophy markers - consistent with previous work^29^. After NEAA supplementation, both MT-1 and Ube2b were decreased even in DMSO-treated controls, but the effect was more pronounced after Dex-induced atrophy signaling. The effect was specific and did not extend to the ubiquitin ligases MAFbx and Murf1 (Fig. 4c–f). These results suggest that MAFbx and Murf1 levels are less sensitive to an excessive increase in NEAA than MT-1 and Ube2b. However, we did not test whether starving cells completely of NEAAs would affect MAFbx and Murf1 expression.

Amino acids exert an additional effect on muscle growth as they serve as nutrients, which increase mTOR activity and lead to an increase in protein synthesis upon mTOR-mediated phosphorylation of S6k^30^. We therefore evaluated changes in phosphorylation of S6k in NEAA-supplemented myotubes and in bear muscle. Indeed, levels of phospho-S6k were significantly higher in myotubes treated with NEAAs (Fig. 4g–i) as well as in hibernating bear muscle (Fig. 4j,k), and might therefore contribute to the observed change in myotube/muscle size.

### Differential transcript expression during hibernation versus conditions of immobilization and/or caloric restriction in humans and mice

**Figure 5:**
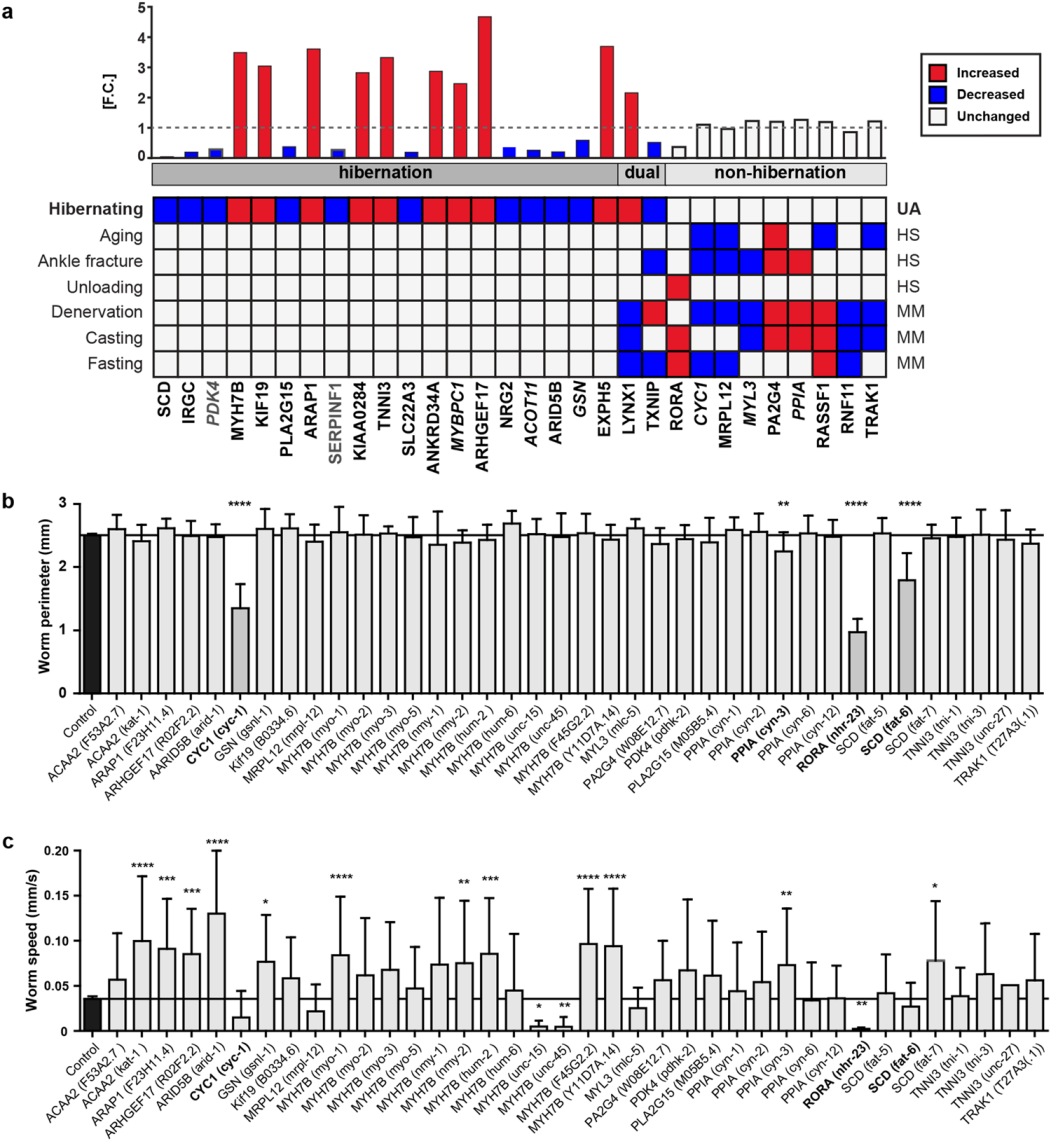
Differential gene expression in hibernating vs. non-hibernating species identifies regulators of skeletal muscle atrophy and metabolism. (**a**) U. arctos mRNA levels in hibernation vs. activity compared to other forms of immobilization or food-deprivation (description of the sciatic nerve lesion and analysis of pre-exising transcriptomic datasets in Methods). Genes also detected in the proteomics dataset are italicized. MM, *M. musculus*; HS, *H. sapiens*; UA, *U. arctos*. (**b**) Downregulation of CYC1, RORA, and SCD homologues in *C. elegans* significantly and reproducibly reduced worm size (*n* > 15 per group, one-way ANOVA, Dunnett’s multiple comparisons test, ****p < 0.001; *p < 0.05). Genes with significant regulation in bold, genes with independent confirmation with dark grey columns. (**c**) Knockdown of *C. elegans* homologues of hibernation-dependent genes results in altered mobility, predominantly of sarcomeric genes. Adapted from thesis by D.M.^66^.

To identify novel target genes that regulate muscle mass, but have not previously been linked to trophic signaling, we compared changes in mRNA regulation during hibernation and six atrophy-related datasets from mice and humans (Fig. 5a). We found 18 genes that were only regulated during hibernation (group 1; hibernation). Two genes were regulated during hibernation as well as in at least half of the other datasets (group 2; dual). Six genes were only regulated in the non-hibernating samples (group 3; non-hibernation). Several of these genes have been linked to striated muscle disease or organ growth through mutations or changes in expression levels, while some affect atrophy-associated signaling pathways but have not been shown to alter muscle size or function (Supplementary Table S4). We used a “guilt-by-association” algorithm^31^ that determines which genes are often co-expressed with an input set and found that the genes in group 3 associate with metabolic, cardiac and growth regulatory genes (Supplementary Table S3), suggesting that they belong to similar functional groups.

To determine whether these genes have an evolutionarily conserved role in the regulation of muscle size, we systematically knocked down the homologous genes in *C. elegans* and determined their effect on worm perimeter (size) and motor activity (speed). Four of our candidates affected worm perimeter (Fig. 5b) and 9 genes affected the worm’s motor function (Fig. 5c). This suggests that select trophic pathways are conserved from planaria to mammals, with a stronger evolutionary pressure on genes that relate to muscle function.

### Pdk4 and Serpinf1 differentially affect the atrophy response of differentiated C2C12 myoblasts

For genes that were not validated in C. elegans, we asked if their role in muscle size regulation was acquired later in evolution and evaluated their effect on myotube size in C2C12 cells. We chose two genes not previously linked to the regulation of muscle growth, but with roles in related signaling pathways. The pyruvate dehydrogenase kinase Pdk4 regulates energy metabolism and ROS production, which results in oxidative damage and protein degradation^32^. Pdk4 protein levels were also elevated in striated muscle of hibernating squirrels^33^.

**Figure 6:**
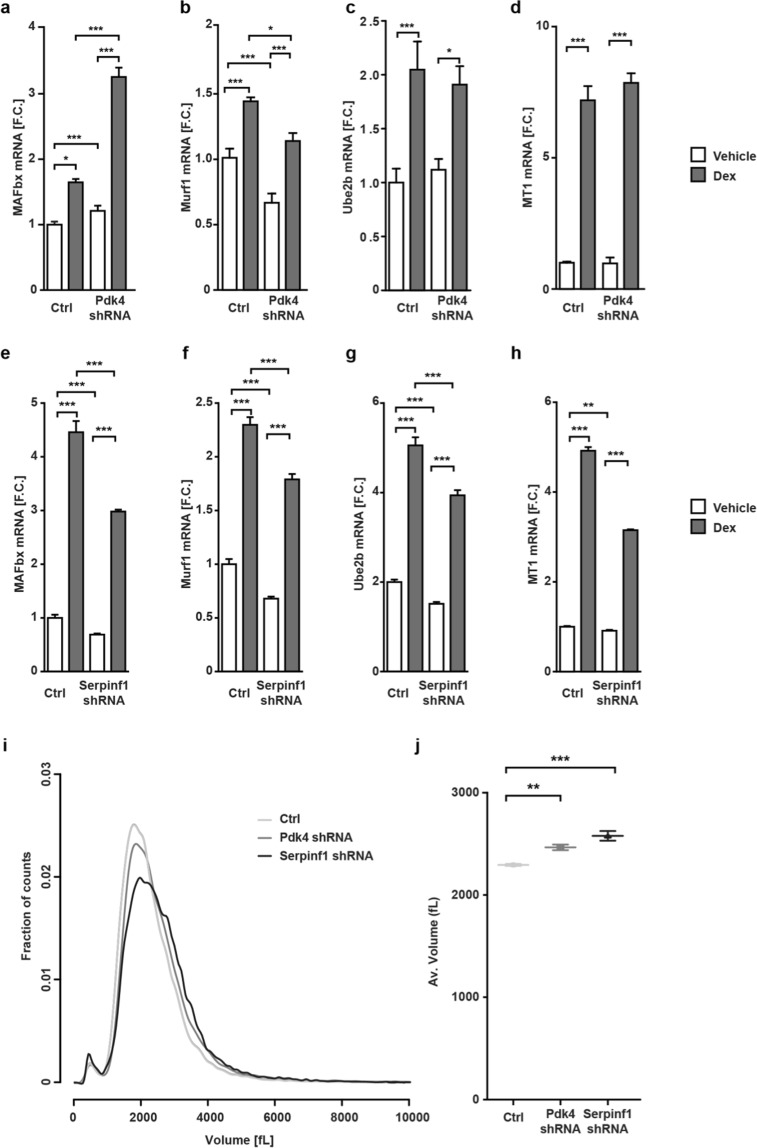
Pdk4 and Serpinf1 reduce myotube size and alter atrophy marker expression. (**a**) Knockdown of Pdk4 increases RNA levels of the atrophy marker MAFbx, but reduces Murf-1 (**b**). (**c**,**d**) Ube2b and MT1 remain unchanged. (*n* = 6, two-way ANOVA, Bonferroni post-hoc test). (**e–h**) *MAFbx*, *Murf1*, *Ube2b* and* MT-1* mRNA levels are decreased in Serpinf1 knockdowns (*n* = 6, two-way ANOVA, Bonferroni post-hoc test). (**i**) The size of undifferentiated C2C12 cells is increased after Pdk4- and Serpinf1- knockdown compared to controls (Ctrl). (**j**) Average cell sizes are significantly different. ***p < 0.001; **p < 0.01, *p < 0.05.

Serpinf1, is a secreted protein which does not have a homologue in *C. elegans*. It is the plasma protein most strongly downregulated in human subjects after weight loss^34^ and enhances Nfkb activation in higher organisms, which has been linked to muscle wasting^8,35^. Decreasing the expression of Pdk4 in differentiated C2C12 cells to mimic its regulation during hibernation leads to an increase in MAFbx mRNA levels, but a decrease in Murf1 levels both in Dexamethasone and control treated myotubes, while Ube2b and MT1 levels are unchanged (Fig. 6a–d). On the other hand, decreasing Serpinf1 expression decreases the expression levels of all four markers whether treated with Dexamethasone or vehicle (Fig. 6e–h), suggesting that Serpinf1 has a stronger effect on atrophy signaling as compared to Pdk4. Indeed, the Serpinf1 knockdown cells are larger than Pdk4 knockdown cells, while both are significantly larger than controls (Fig. 6i,j). Thus, both Pdk4 and Serpinf1 contribute to the regulation of myotube size in higher organisms.

In summary, we identified and validated two novel therapeutic targets, which affect atrophy signaling in myotubes: Serpinf1 and Pdk4 were differentially regulated in immobilization with or without atrophy and  knocking them down enhanced C2C12 cell growth in tissue culture. We furthermore validated three additional targets *in vivo* (Cyc1, Rora, Scd), indicating that their trophic effect extends back in evolution to non-mammals. Finally, we document metabolic changes that alter non-essential amino acid levels in muscle, which are consistent with maintaining muscle size.

## Discussion

We used *de novo* proteomic and transcriptomic analysis of hibernating grizzly bears to understand the molecular basis of preserved muscle mass in periods of inactivity and reduced calorie intake. Translating these findings to adapt the human atrophy response can help improve recovery and outcome for patients with muscle disease and preserve muscle strength in healthy individuals with reduced mobility or exposure to microgravity during spaceflight.

Our proteomic data indicates a decrease in mitochondrial protein content during hibernation, primarily TCA cycle enzymes (Fig. 2) and similar results have recently been reported^26^. In addition, Pdk4 protein levels were elevated, which would lead to the reduction in pyruvate shuttling through the TCA cycle. Higher Pdk4 protein levels have been reported in skeletal muscle of hibernating squirrels^33^, suggesting that this might be a conserved response among hibernating mammals. A decrease in TCA cycle activity has been linked to an increase in mammalian cell size *in vivo*^36^. Our metabolic models—built on this data and that from ageing humans—predicted that 6 NEAAs are decreased in aging muscle but increased during hibernation, which we confirmed by metabolomic profiling of grizzly bear muscle a subset of TCA-derived by not glycolysis-derived NEAAs. These changes in NEAA availability could be advantageous to muscle growth during hibernation. We did not find a significant increase in NEAA-rich proteins during hibernation, suggesting that the production of NEAAs might not simply increase substrate availability for protein synthesis. Rather, NEAAs might exert a direct effect on trophic signaling as supported by an increase in phospho-S6k levels during hibernation, which implies that atrophy signaling via the mTor pathway^37,38^ is regulated to maintain muscle mass. Indeed, supplementing differentiated C2C12 cells with NEAAs increased myotube thickness, which was accompanied by a similar increase in phospho-S6k levels (Fig. 4j,k) and decreased mRNA levels of the E2 ligase Ube2b, as well as the oxidative-response protein MT-1, which both have been reported to increase in multiple cases of muscle atrophy^39^.

Since* C. elegans* has successfully been used as a model for neuromuscular disease with the perimeter largely determined by the worm’s muscle mass^40^, we tested the effect of NEAA supplementation on *C. elegans* diameter in both agar and liquid cultures. However, we did not find a significant effect (Fig. 5b/data not shown). This is in line with what has been reported in patients, where exogenous NEAA supplementation has yielded limited benefit. As the route of application might be critical, we would expect a potential benefit from stimulating endogenous AA synthesis versus exogenous supply.

In skeletal muscle of hibernating grizzly bear, transcriptional changes affect genes associated with insulin-Akt-mTor signaling, suggesting that changes in NEAA levels are not the only reason we observe an increase in mTOR activity during hibernation, but that several mechanisms cooperate to maximize the effect on mTOR signaling.

Towards the identification of therapeutic targets, we compared transcriptional regulation during hibernation with other conditions associated with immobilization and reduced caloric intake. Genes regulated only during hibernation, could help explain the bears’ ability to preserve muscle mass under conditions that are detrimental in other species. Similar approaches in hibernating squirrels have led to the successful identification of genes such as Sgk1 as regulators of muscle size in non-hibernating mammals^12^. Unfortunately Sgk1 was not detected on the transcriptional or protein level in our samples making it difficult to determine whether it also plays an important role in preserving hibernating muscle mass in bears. We found that the expression of structural proteins (Mybpc1, Tnni3, Myh7b) was increased, while expression of the actin-severing protein gelsolin (Gsn) was decreased. This is consistent with transcriptional changes that favor muscle-building.

To prioritize potential therapeutic targets, we investigated the differentially regulated genes in *C. elegans*, as evolutionary conserved pathways would provide the most robust candidates for therapy. Indeed, knockdown of metabolic genes such as the homologues of Cyc1 and Scd decreased worm perimeter. A human condition in which CYC1 levels are decreased due to protein instability is accompanied by growth retardation (Table S4) and Scd is expressed at higher levels upon exercise suggesting that these genes are evolutionary conserved regulators of muscle size. The strongest reduction in body size resulted from the knockdown of Rora - an effect, which has already been reported in mice and could in part relate to metabolism through an associated decrease in Scd-1/2^41^.

Among the genes not previously associated with muscle growth and had no effect on worm perimeter, we confirmed the role of Pdk4 and Serpinf1 on atrophy signaling in a tissue culture model of muscle atrophy. We suggest that the related downstream pathways are conserved between mammalian species, but not in lower organisms, with *C. elegans* lacking a Serpinf1 homologue altogether.

**Figure 7:**
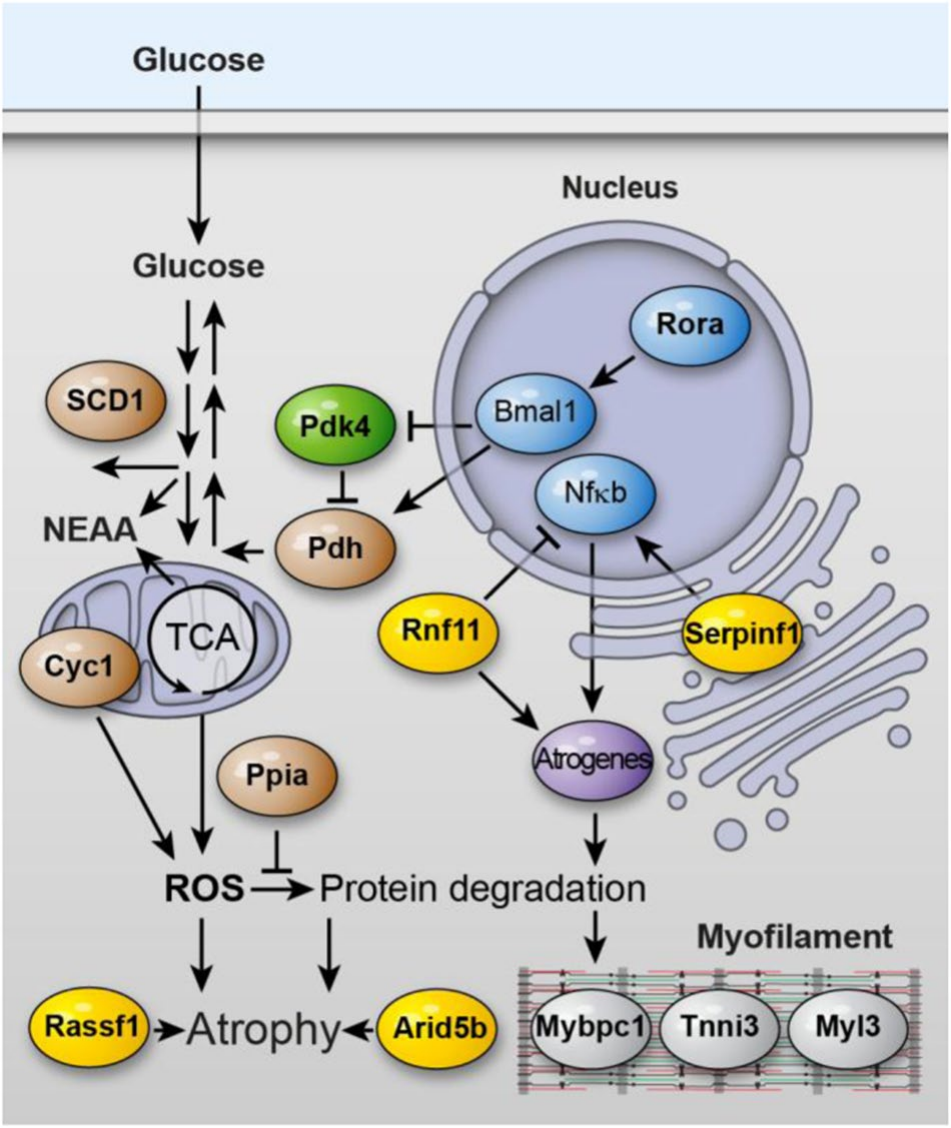
Trophic signaling in skeletal muscle. Genes differentially regulated in hibernation vs. skeletal muscle atrophy (bold) relate to trophic signaling and metabolism. NEAA - non-essential amino acids; ROS - reactive oxygen species.

Knocking down Pdk4 in C2C12 cells lead to a mild increase in size. The increase in size was accompanied by a decrease in Murf1 in differentiated myotubes. Murf1 binds to and regulates the ubiquitination of cytoskeletal and metabolic enzymes that constitute a large fraction of myotube mass^42^. It thus appears favorable to reduce Pdk4 levels in atrophy-promoting conditions, which is indeed what we observed on the mRNA level. However, in hibernation we and others^33^ find an increase in Pdk4 protein (Figs. 1D, 2A). An important difference between the culture system and hibernation is the limited availability of circulating NEAA during hibernation, while culture medium has an abundance of it. It is thus possible that in conditions where an autonomous increase in NEAA is desirable such as hibernation, post-transcriptional mechanisms that increase PDK4 protein levels might be evolutionarily important to counter those that decrease it at the mRNA level. The downregulation of Serpinf1 mRNA during hibernation would also help preserve muscle mass, as its suppression in myotubes reduces atrophy signaling and increases muscle size. While the exact mechanism by which Serpinf1 affects muscle mass is still unknown, it might involve Nfκb-signaling, which enhances muscle wasting and has been linked to Serpinf1 activity in neurons^35^. The combined hibernation dependent effects on muscle mass are summarized in Fig. 7.

In summary, this study demonstrates how thousands of years of selective pressure have produced coordinated changes in bears making them resistant to atrophy under conditions that normally induce muscle loss. By regulating the expression of genes related to organ growth, energy metabolism, and amino acid availability, bears are largely resistant to muscle atrophy during periods of immobilization or reduced caloric intake. We have identified several genes previously not associated with the regulation of muscle mass and found evidence that NEAAs suppress atrophy signaling as a first step to improving muscle atrophy treatment in patients.

## Methods

### Grizzly bear samples

For this study, we used four bears housed at the Washington State University Bear Research Education and Conservation Center. The bears included 2 cubs, a subadult and an adult. All animals were cared for according to the Bear Care and Colony Health Standard Operating Procedures approved by the Washington State Institutional Animal Care and Use Committee (IACUC) in accordance with the US National Institutes of Health guidelines. Feeding ceased the first week of November marking the onset of hibernation, and resumed the second week of March, which marked the end of hibernation. During hibernation bears were paired and housed in unheated dens (3 × 3 × 2.5 m^3^) with straw bedding and water. They were afforded continuous access to an outdoor area (3 × 5 × 5 m^3^) via a small door, which ensured exposure to daily temperature and light fluctuations. Camera surveillance (Silent Witness) indicated that bears were resting 98% of the time while hibernating. Air dart intramuscular delivery of tiletamine HCl/zolazepam HCl (5 mg/kg while active and 2 mg/kg when hibernating) was used to anesthetize the animals. A fixed plane of anesthesia for muscle biopsies was maintained by the endotracheal administration of isoflurane in 100% oxygen. Animals were considered adequately anesthetized based on their irresponsiveness to instrumentation, lack of a palpebral reflex and lack of jaw tone. Approximately 1 × 1 × 2 cm^3^ biopsies were obtained from a ~4 cm incision over the gastrocnemius muscle after the surface of the leg was shaved, prepped and kept sterile. Biopsies were immediately snap frozen, then stored at −80 °C.

### Sciatic nerve lesion in mice

All experiments involving mice were performed according to governmental and institutional guidelines for animal use in research and have been approved by the local German authorities (LaGeSo Berlin, Germany). Denervation was performed as previously described^43^. In brief, adult male (12-week) mice were anesthetized with isoflurane. The sciatic nerve of the left leg was exposed and a 2 mm piece was removed. The right leg remained innervated and was used as a control. Mice were sacrificed and the gastrocnemius muscle was then prepared and snap frozen in liquid nitrogen 14 days after denervation. The subsequent analysis was unblinded.

#### Protein extraction

Tissue samples were frozen in liquid nitrogen and disrupted using a bead mill as described earlier^44^ with slight modifications. In short the powder resulting from the bead mill treatment was resolved in an appropriate volume of sample buffer (8 M urea; 2 M thiourea) and sonicated three times for three seconds on ice (Sonoplus HD 2200). After removal of insoluble cell debris by centrifugation (15,300 rpm; 1 h; 4 °C) the supernatant, containing the soluble cytosolic proteins, was transferred and used for further analysis. Protein concentrations were determined by Bradford assay (Biorad).

### Peptide sample preparation

For each sample, we tryptically digested two technical replicates of 4 µg each. Each replicate was diluted with 20 mM ammonium bicarbonate such that the final concentration of urea was <1 M. Samples were subsequently reduced in 2.4 mM DTT at 60 °C for 1 hour, then alkylated in 10 mM iodoacetamide at 37 °C for 30 min. Samples were digested in trypsin (1:50) at 37 °C, overnight. 1% acetic acid was added to terminate digestion, after which the peptides were desalted on a C-18 reverse phase column (ZipTip µ-C18, Millipore).

### Mass spectrometry FTICR analysis

The resulting peptide mixtures were separated using nanoAcquity UPLC reverse phase column (BEH130, C18, 100μm–100mm) on a nanoAcquity UPLC system (both Waters, Waters Corporation) as previously described^44^. MS data were generated using a LTQ-FT mass spectrometer (Thermo Electron). For each sample two technical replicates were measured.

### Quantitative analysis and protein identification

The raw data from the LC-ESI-MS/MS measurement were imported into the Rosetta Elucidator software version 3.3 (Ceiba Solutions) and processed as described earlier^45^ with slight modifications. In brief, the following workflow was used: (i) feature detection; (ii) feature alignment across all MS runs; (iii) feature identification via SEQUEST database search (via Sorcerer build 4.04, Sage-N Research Inc.) against a forward reverse database containing human entries obtained from Uniprot; (iv) feature annotation based on protein teller score (>0.95), (v) median normalization using the annotated features, (vi) Filtering for unique peptides. The obtained feature intensities were averaged for each sample, summed up to peptide and protein intensities. These steps were carried in a visual script within the software Elucidator. The obtained protein intensities were exported to Excel for further analysis. Differential protein expression was determined based on results of a two-way ANOVA taking age and activity state as factors. Proteins significantly regulated based on activity were used for all further analyses (*p*-value < 0.05; Table S1).

### RNA preparation for RNA-seq

We isolated total RNA from grizzly bear muscle using Invitrogen’s standard protocol for TRIzol. RNA was precipitated in 70% ethanol at the end. mRNA was then Poly(A)- purified on Dynabeads (Invitrogen) over two rounds, according to the manufacturer’s instructions. Sample quality was assessed using Bioanalyzer (Agilent Technologies).

### Full-length enriched cDNA library construction and normalization

The construction of normalized full-length-enriched cDNA libraries for 454 sequencing requires three steps: (1) the synthesis of double strand cDNA using a modified RACE technique, (2) the subsequent removal of poly(A):T tails using the methylation sensitive type II restriction enzyme GsuI, followed by the ligation of a new DNA adaptor, and (3) the normalization of the resulting cDNA library using duplex-specific nuclease (DSN). The DSN normalization method is based on the denaturation–reassociation of double-stranded (ds) cDNA coupled with the degradation of the ds cDNA fraction formed by abundant transcripts^46,47^, and requires the presence of adaptor sequences at each terminus of the cDNA to prime PCR amplification.

### 454 FLX titanium sequencing

cDNA libraries were normalized then quantified using Quant-iT dsDNA HS Assay Kit (Invitrogen). 5 μg of the normalized libraries was used to prepare the sequencing library using the 454 GS FLX Titanium General Library Preparation Kit according to the manufacturer’s instructions. The library was then sequenced over 200 cycles on a 454 GS FLX sequencer according to the manufacturer’s instructions.

### Single-end cDNA sequencing using Illumina GAIIX

Single-end sequencing libraries were constructed using 5 µg of full-length cDNA as per the instructions for Illumina’s Genomic DNA Single End Sample Prep kit. 10 nM of adaptor-ligated library material was hybridized to the surface of flow cells, after which DNA clusters were generated using the Illumina cluster station, followed by 120 cycles of sequencing on the GAIIX as per the manufacturer’s recommendations.

### Paired-end RNA-Seq using Illumina GAIIX

300 ng of poly(A) RNA were fragmented in a total volume of 20 mL using 5× fragmentation buffer (150 mM MgOA, 500 mM KOAc, 200 mM Tris-Acetate, pH 8.1) for 3.5 min at 94 °C. The fragmented RNA was subsequently precipitated then converted to first-strand cDNA using Superscript II (Invitrogen), then second-strand cDNA using RNase H (Invitrogen) and *E. coli* DNA PolI (Invitrogen). The paired-end sequencing libraries were prepared using Illumina’s Genomic DNA Paired End Sample Prep kit as per the manufacturer’s instructions. Libraries were diluted to 10 nM, adapter-ligated DNA was hybridized to the surface of flow cells, DNA clusters were generated using the Illumina/Illumina cluster station, and 2 × 76 cycles of sequencing were performed on the GAIIX as per the manufacturer’s instructions.

### *De novo* transcriptome assembly

The 454 reads were assembled using Newbler 2.3 (Roche) with default parameters. The Illumina assembly comprised of Illumina paired-end was obtained by using SOAPdenovo software^48^ (http://soap.genomics.org.cn) with default parameters. The contigs longer than 100 bp in the Illumina assembly were combined together with 454 reads for the final assembly using Newbler with default parameters.

### Transcriptome annotation

Out of 43599 transcripts, 22641 were aligned to human protein database (Ensembl version 66) using BLAST with *e*-value ≤ 1E-10, corresponding to 9768 human proteins.

### Differential transcript expression

Single-end Illumina reads were aligned to transcripts using SOAP2 allowing at most 2 mismatches to estimate the expression of each transcript. Transcripts corresponding to a total of 4783 genes were aligned. NOISeq^49^ was used to estimate the likelihood of differential expression (probability >0.8; Table S2).

### Illumina microarrays

mRNA was extracted by homogenizing tissue in TRIzol (Invitrogen), followed by chloroform extraction and ethanol precipitation. RNA was subsequently purified on RNeasy columns (Qiagen) then kept at −80 °C. Ribosomal RNA was depleted using Invitrogen’s RiboMinus kit. Approximately 100 ng of total RNA was then used to generate biotinyated cRNA using Illumina’s TotalPrep-96 RNA amplification Kit (Life technologies) as per the manufacturer’s instructions. Expression profiling was done on Illumina’s MouseRef-8 v2.0 Expression Bead chip. The Gene Expression Module V1.9.0 of Genome Studio V2011.1 was used to quantile-normalize chips. Gene profile data was subsequently used for analysis, with only genes significantly higher than background (*p* < 0.05). Differential expression was considered at *p* < 0.05.

### Pre-existing transcriptomic datasets

To increase the repertoire of muscle atrophy models we were comparing, we picked GEO datasets: GSE674^28^ (Aging, HS), GSE8872^50^ (Ankle fracture, HS), GSE21496^51^ (Unloading, HS), GSE25908^52^ (Casting, MM) and GSE24504^53^ (Fasting, MM). Dataset GSE24504 was missing the respective CEL files, so to keep the analysis consistent across datasets, all were analyzed using GEO2R. The subsamples we compared in GSE674 were those from young women between 20–29 years of age and others between 65–71 years. Differential expression was considered at *p* < 0.01. In GSE8872, the casted legs from ankle-fracture patients were compared to the contralateral legs as controls and differential expression was considered significant at *p* < 0.01. In the case of GSE25908, we compared the right legs casted for 14 days to the left legs from the same mouse. Differential expression was considered if the BH-adjusted *p*-value was <0.05. As for GSE24504, samples after 72 h of fasting were compared to controls, and differential expression was considered significant if the adjusted *p*-value was <0.05.

### Meta-analysis of atrophy associated differential gene expression

In order to identify genes that are important in the atrophy response under different atrophy-inducing conditions, and across species, we compared changes in gene expression across a total of seven datasets. Since the data was acquired on different platforms, comparing FCs does not make much sense. We decided to discretize FC values, to −1 if the gene was less expressed under the atrophy-inducing condition; 1 if the gene was overexpressed and 0 if there was no significant change. We only considered genes that were present on the chips and were also sequenced. Genes were assigned to three groups: Group 1 consists of those regulated during hibernation only; Group 2 are those regulated during hibernation and at least half of the other conditions and Group 3 are those that are not regulated during hibernation, but are regulated in at least half of the other conditions.

### Guilt-by-association functional inference

Coexpressed genes were determined using the software GeneFriends^54^, which queries a vast set of RNAseq datasets for genes that are often coexpressed (either increased or decreased) with the input gene list. Genes increased or decreased during hibernation (group 3) were used as input gene lists for the algorithm. The resulting list of co-expressed genes were filtered for a *p*-value ≤ 0.01, then only the genes that were associated with ≥5 genes in the input lists were considered and utilized for the subsequent enrichment analysis.

### KEGG pathway enrichment analysis

All analyses were done using the CLUEGO plugin in Cytoscape^55–57^. Kappa-score threshold was always set to 0.3, min % genes to 0 and min number of genes to 2. *P*-values were Benjamini-Hochberg corrected for multiple testing and denoted (*p*-adj.).

### Metabolic modeling

Condition specific models were constructed from Recon2 as a reference model since not much is known about grizzly bear metabolism and how it deviates from metabolism in other mammals, while Recon2 is the most exhaustive reconstruction of a mammalian system at the time of the analysis^27^. Also most proteins were identified based on their homology to human counterparts impeding the identification of any bear-specific metabolic enzymes, even if they existed. The GIMME algorithm^58^ within the createTissueSpecificModel function in the COBRA toolbox^59^ was used with the biomass reaction as the objective function. Expression data was provided as 0/1 based on relative changes in expression levels between the 2 conditions compared. If a protein/mRNA species was increased in one condition compared to the other it was assigned a value of 1 for that condition and 0 for the other, while a value of 1 was assigned for all species expressed but unchanged between the conditions.

When creating the hibernation and activity-dependent models for *U. arctos* the expression data was provided based on changes in protein levels, while in case of the aging models of *H. sapiens* muscle mRNA levels were used.

All computations were done locally on a 64-bit machine and optimization was done using a CPLEX solver (version 7, Tomlab) in MATLAB.

### Metabolite analysis

Muscle samples were stored in liquid nitrogen. Powder was prepared and a mixture of methanol-chloroform-water (MCW) (5:2:1/v:v:v) (Methanol LC-MS-grade, Chloroform Reagent Plus 99,8% Sigma-Aldrich) with cinnamic acid as internal standard (Sigma-Aldrich) was added. Samples were shaken at 750 rpm and 4 °C for 60 min. After addition of water (half volume), samples were centrifuged for 10 min at 5000 g to separate the polar (top), lipid (bottom) and interface (tissue debris) layers. The polar phase was dried under vacuum for 12 h. Metabolite analysis was performed on a gas chromatography coupled to time of flight mass spectrometer (Pegasus III- TOF-MS-System, LECO Corp., St. Joseph, MI, USA), complemented with an auto-sampler (MultiPurpose Sampler 2 XL, Gerstel, Mülheim an der Ruhr, Germany) as described in^60^. Data analysis was performed using ChromaTOF Version 4.42 (LECO). The Golm metabolome database (GMD) was used to identify substances with respect to spectra-similarity and retention index. Data matrices for relative quantification were extracted from the mass spectra using MetMax software^61^. Data were normalized to cinnamic acid for further analysis.

### Non-essential amino acid (NEAA) distribution in regulated proteins

As peptide identification in U. arctos were based on sequence homology to those in *H. sapiens*, we assumed the amino acid composition of the proteins regulated during hibernation to be that of their human counterparts. The percentage of NEAA is calculated as a function of the total number of amino acids per protein.

### Short hairpin RNA (shRNA) construct generation

shRNA oligo design was based on the entries of the RNAi consortium^62^ with the highest adjusted score for our gene of interest, indicating a high knockdown efficiency with minimal off-target effects. Constructs TRCN0000361693 and TRCN0000313900 were selected for Pdk4 and Serpinf1 shRNAs respectively. 5′ phospho-oligonucleotides were ordered with the same sequence, except that the BglII (GATC) and XhoI (TCGA) restriction sites were appended at the 5′ end of the forward (fwd) and reverse (rev) strands, respectively (MWG; Pdk4-fwd: 5′ p- gatcagacgctatcatctacttaaactcgagtttaagtagatgatagcgtcttttttg-3′, Pdk4-rev: 5′ p- tcgacaaaaaagacgctatcatctacttaaactcgagtttaagtagatgatagcgtct -3′, Serpinf1-fwd: 5′ p- gatctcaccttcccgctagactatcctcgaggatagtctagcgggaaggtgatttttg-3′, Serpinf1-rev: 5′ p- tcgacaaaaatcaccttcccgctagactatcctcgaggatagtctagcgggaaggtga-3′). 5 ul of fwd and rev oligos (20 uM) were annealed together in NEB buffer 2 placed in a beaker of boiling water and left to cool overnight. 2 μl of the annealed oligos were then ligated into 20 ng of pSUPER Retro Stuffer (OligoEngine; digested with XhoI and BglII). The resulting constructs are termed pshRNA-SRS.

### C. elegans knockdown and phenotyping (size and motility measurements)

C. elegans WT line (N2) was maintained at 20 °C on NGM plates seeded with OP50 *E. coli* bacteria following standard methods. *E. coli* RNAi feeding strains for all tested genes were obtained from the Ahringer RNAi library^63^. Bacterial cultures of *E. coli* HT115 containing L4440 empty vector (EV) were used as control. Worm populations were synchronized by bleaching using standard protocols and synchronized L1 larvae were placed on RNAi plates and grown until egg-laying started (after ~3 days). Videos where captured using a light microscope, DinoEye eye piece camera and VirtualDub 1.10.4 software, with the following parameters: 640 × 480 pixels, 16 seconds, 30 frames per second. The videos where imported into ImageJ software, cutting the first second of each video to circumvent flickering of the first few frames. Subsequently the videos where analyzed using the ImageJ plugin wrMTrck^64^, choosing appropriate settings to measure worms with their corresponding tracks and to exclude artifacts (below). The final list of measured events was filtered as follows to include only events with: time ≥7.5, stdPerim ≤0.4 and bends ≤20. The time threshold ensured that no worm was measured twice (if a worm was crossed by another during the 15 seconds video, measurements for this worm were interrupted, resulting in two events of the same worm with e.g. 9 and 4 seconds). The stdPerim threshold sorted out worms with a high standard deviation of their average perimeter over the course of the image stack being analyzed (this were usually worms that crawled in or out of the field of view of the camera, resulting in a too small perimeter). Finally, the bends threshold was used to remove remaining artifacts (e.g. dirt particles). Using this approach and analyzing 397 movies we extracted the following data for each RNAi clone and EV control: number of worms (n), mean perimeter (in mm) and mean speed (calculated dividing the distance covered by the time in mm/sec).

Settings for wrMTrck batch were as follows:

fileTypeNr = 1; imageType = 0; backSub = 4; threshMode = 6; fixedThresh = 0; skeletonize = 0; movieDuration = 0; fps = 30; pixPrMm = 84.4; minSize = 300; maxSize = 1200; maxVelocity = 50; maxAreaChange = 70; minTrackLength = 20; bendThreshold = 4; bendDetect = 1; rawData = 0; Additionally, the batch file was edited to include the following functions after the creation of binary image stacks: dilate, fill holes. For worms that were much smaller in size (nhr-23, cyc-1, and fat-6), alternative size values where used: minSize = 80; maxSize = 700.

### Tissue culture

Cells were maintained in Dulbecco’s Modified Eagle’s Medium (Gibco) supplemented with 10% FCS (Invitrogen) and 0.1% Pen/Strep (Invitrogen; FGM). C2C12 cells were differentiated by switching to 2% HS (Sigma, Lot# I2J013; DM) at approximately 80% confluence. DM was changed every 2 days and cells were maintained for up to 8 days until nicely differentiated before further treatment. Atrophy was induced with 0.1 mM dexamethasone in DMSO (Sigma) for 2 days. MEM-NE-AA (Gibco) was supplemented at 10 × concentration to regular DMEM.

For viral packaging, Phoenix cells were grown to 80% confluence in 10 ml dishes then switched to fresh FGM before they were CaCl_2_-transfected with pshRNA-SRS or pBabe-H2B-eGFP (Addgene, plasmid 26790) as a control (30 µg DNA, 250 µM CaCl_2_, 550 µM chloroquine, 2xHEPES-buffered saline). Pdk4 and Serpinf1 shRNA expression vectors were generated using pSUPER Retro (OligoEngine) as a backbone and inserts derived from constructs TRCN0000361693 and TRCN0000313900 of the RNAi consortium^62^. 24 h post-transfection, the medium was changed to 6 ml FGM which was harvested 48 h later, sterile filtered and transferred to a plate of 50% confluent C2C12 cells. The same Phoenix cells were then kept in 4 ml FGM which was harvested and added to the C2C12 cells 6 h later. 24 h later the same procedure was repeated using the same cells so that a total of 4 infections were achieved. Stable shRNA-expressing polyclonal cultures were obtained by selection in puromycin (1.2 µg/ml; Sigma) for at least 1 week.

### Cell size quantification

Cell size of C2C12 myoblasts was quantified using a Z2 Coulter particle count and size analyzer (Beckman Coulter). 18 hours prior to counting, cells were counted and seeded at equal densities for Control and Pdk4/Serpinf1- knockdown cells. Data was acquired using the accompanying Z2 software and subject to further analysis using a custom script in R.

Myotube quantification was adapted from a method described previously^21^. In short, at least 20 random bright field images were taken at 20x magnification and subsequently imported into and analyzed in ImageJ by measuring the thickness of the widest area across myotubes of similar morphology. In each group 20 ± 5 cells were used for quantification.

### qRT-PCR

Cells were washed once in PBS, lysed in RLT buffer (Qiagen) and either snap-frozen and stored at −80 °C or immediately processed. RNA extraction was done using the RNeasy Mini kit (Qiagen) with DNAse digestion as per the provider’s protocol. 1 µg of RNA was reverse transcribed into cDNA using High Capacity reverse transcriptase (Applied Biosystems). 10 ng of cDNA per well was used together with TaqMan gene expression master mix (Applied Biosystems) and the following TaqMan probes (Applied Biosystems) with β-actin as endogenous control: Pdk4, Mm01166879_m1; Serpinf1, Mm00441270_m1; MAFbx, Mm01207878_m1; β-actin, 4352341E. Experiments were done using 3 biological replicates, pipetted in triplicates. The values corresponding to technical replicates were averaged and the mean values of the biological replicates were then used for statistical testing.

### Western blotting

Samples were prepared similarly as previously described^65^ with slight modifications. In short, cells were washed once in PBS, lysed in RIPA, treated with benzonase (Sigma) and rotated at 4 °C after which they were stored at −20 °C. Protein concentration estimation was done using a BCA estimation kit (Pierce). Equal concentrations of protein were prepared in 4x Lammeli buffer and boiled for 10 min before loading on an SDS-PAGE gel. Resolved proteins were then transferred onto a nitrocellulose membrane (Amersham), blocked in 5% BSA/PBS and then incubated overnight with one of the following antibodies, washed in TBS-T and followed by the respective secondary Ab for 2 h. γ-tubulin was used last as a loading control: anti-Pdk4 (1:1000, Abcam, ab38242), anti-Serpinf1 (1:2000, Sigma-Aldrich, AV20020-50UG), anti-γ-tubulin (1:1000, Sigma-Aldrich, T6557), ECL anti-rabbit IgG-HRP (1:5000, GE Healthcare), ECL anti-sheep IgG-HRP (1:5000, GE Healthcare). Blots were developed with ECL substrate (Pierce) and the signal detected on a Fusion-FX7 Chemiluminescence detection system (PeQlab). Originals are provided in Supplementary Fig. S4. Each experiment involved biological triplicates, which were subsequently used for statistical testing.

### Statistical analysis

All statistical tests were done in R including a batch two-way ANOVA, Student’s t-test and multiple-correction testing. Data was visualized in Excel (Microsoft) or GraphPad Prims 5. * Indicates 0.01 < *p*-value < 0.05, **0.01 < *p*-value < 0.001, ***p-value < 0.001.

### Accession numbers

Illumina chip data generated during this study is deposited in the GEO database under accession number GSE62812 and RNA Seq data under GSE63864.

## Supplementary information

Supplementary information
