# Author Correction: Proteomic and Transcriptomic Changes in Hibernating Grizzly Bears Reveal Metabolic and Signaling Pathways that Protect against Muscle Atrophy

**DOI:** 10.1038/s41598-020-61340-4

**Published:** 2020-03-04

**Authors:** D. A. Mugahid, T. G. Sengul, X. You, Y. Wang, L. Steil, N. Bergmann, M. H. Radke, A. Ofenbauer, M. Gesell-Salazar, A. Balogh, S. Kempa, B. Tursun, C. T. Robbins, U. Völker, W. Chen, L. Nelson, M. Gotthardt

**Affiliations:** 1 Neuromuscular and Cardiovascular Cell Biology, Max Delbrück Center for Molecular Medicine, Berlin, Germany; 2 Berlin Institute for Medical Systems Biology, Max Delbrück Center for Molecular Medicine, Berlin, Germany; 3 Interfaculty Institute for Genetics and Functional Genomics, University Medicine Greifswald, Greifswald, Germany; 4 College of Veterinary Medicine and Department of Veterinary Clinical Science, Washington State University, Pullman, Washington USA; 5 School of the Environment and School of Biological Sciences, Washington State University, Pullman, Washington USA; 6 DZHK (German Centre for Cardiovascular Research), partner site Greifswald, Greifswald, Germany; 7 Experimental and Clinical Research Center, Charité & Max Delbrück Center for Molecular Medicine, Berlin, Germany; 8 Charité Universitätsmedizin Berlin, Berlin, Germany; 9 DZHK (German Center for Cardiovascular Research), partner site Berlin, Berlin, Germany

Correction to: *Scientific Reports* 10.1038/s41598-019-56007-8, published online 27 December 2019

The link to the original data is incomplete. The following Data Availability text should be included:

“The grizzly proteomics data is available at the ProteomeXchange Consortium (PXD016974), and the grizzly RNA-seq data is available at Gene Expression Omnibus (GSE63864).”

