# Supplementary material for: Proteomic and Transcriptomic Changes in Hibernating Grizzly Bears Reveal Metabolic and Signaling Pathways that Protect against Muscle Atrophy
Source: Sci Rep. 2019 Dec 27;9:19976. doi: 10.1038/s41598-019-56007-8 (PMC6934745; doi:10.1038/s41598-019-56007-8)
Supplement: Supplementary file 1 — Supplementary information [file 41598_2019_56007_MOESM1_ESM.pdf]

Proteomic and transcriptomic changes in hibernating grizzly bears reveal mechanisms that protect against muscle atrophy.

Douaa A. Mugahid, Tutku G. Sengul, Xintian You, Yongbo Wang, Leif Steil, Nora Bergmann, Michael H. Radke, Andreas Ofenbauer, Manuela Gesell-Salazar, Andras Balogh, Stefan Kempa, Baris Tursun, Charles T. Robbins, Uwe Völker, Wei Chen, Lynne Nelson, Michael Gotthardt

Supplement:

Figures S1-S4

Tables S1-S4

Figures

Supplement Figure 1, related to Figure 1

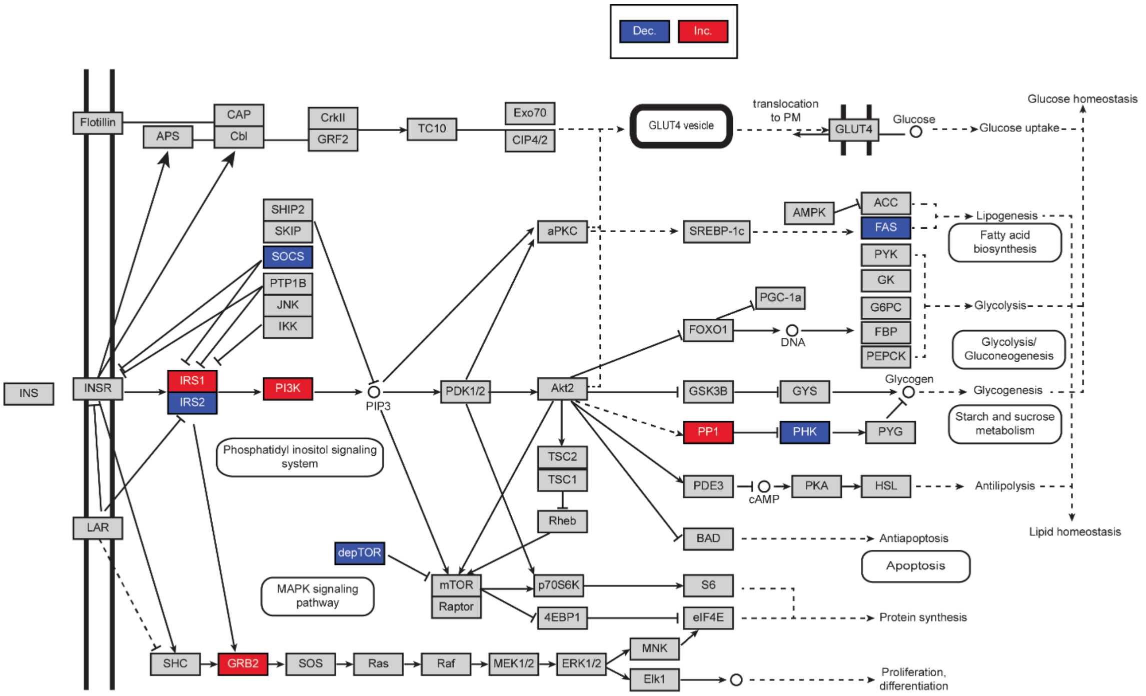

Fig. S1: Mapping changes in mRNA levels of insulin signaling. Pathway during hibernation (adapted from KEGG). Genes with decreased expression are depicted in blue, with increased expression in red.

Supplement Figure 2, related to Figure 3

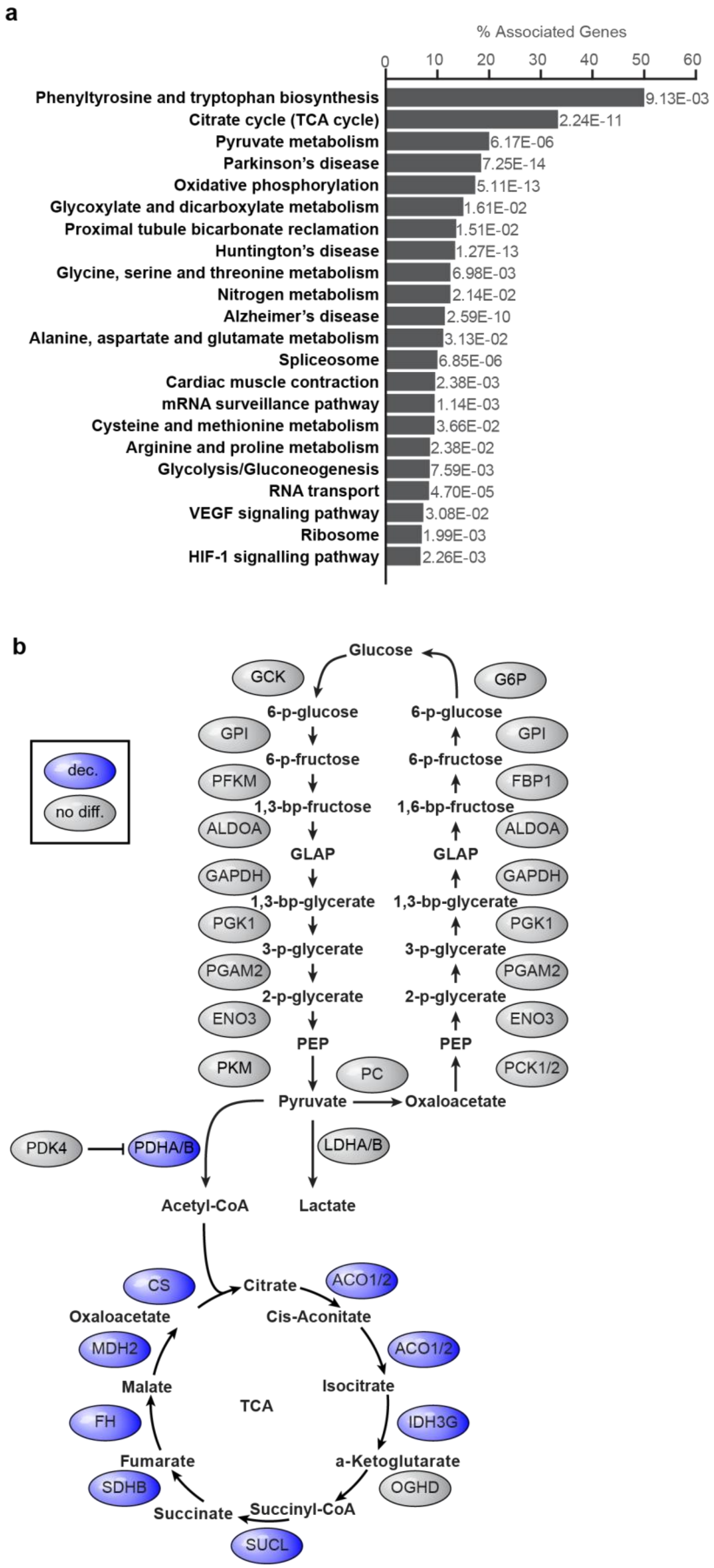

Fig. S2: Changes in mRNA levels in aging skeletal muscle indicate alterations in metabolic regulation. (**a**) KEGG pathways with mRNAs regulated in aging female muscle. In red are the adjusted p-values per term. (**b**) Transcriptional changes in central glucose metabolism in aging muscle.

Supplement Figure 3, related to Figure 6

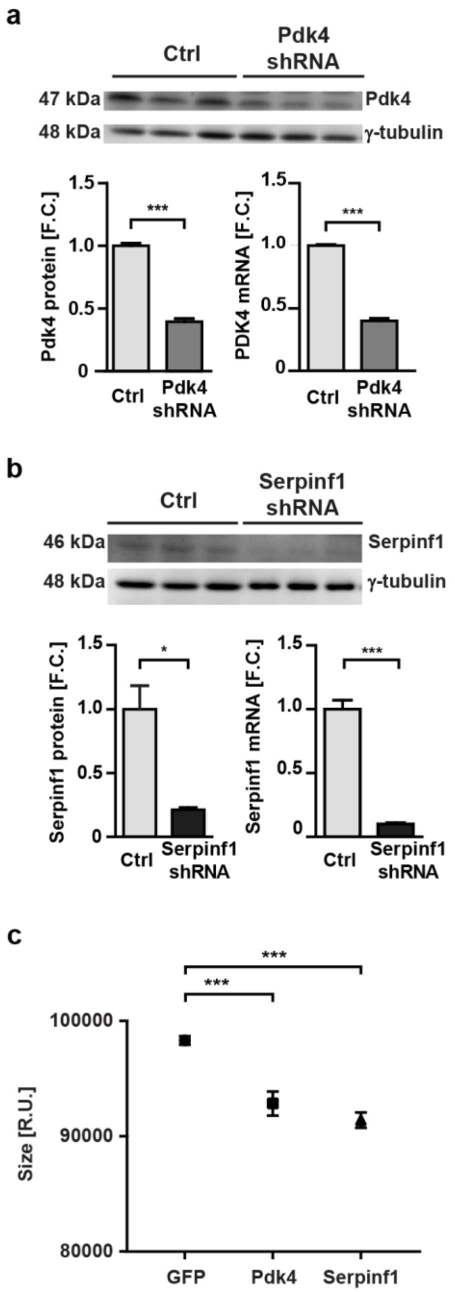

Fig. S3: Validation of the shRNA mediated knock-down in C2C12 cells. Protein expression was normalized to γ-tubulin. (**a**) Pdk4 protein and mRNA levels were reduced to <50% after shRNA treatment (n=3, mean± s.e.m., t-test, ***P < 0.001). (**b**) Serpinf1 protein and mRNA levels were reduced to <25% after shRNA treatment (n=3, mean± s.e.m., t-test, *P < 0.05, ***P < 0.001). (**c**) Over-expressing eGFP-tagged Pdk4 or Serpinf1 leads to a decrease in cell size (FSC-A) compared to eGFP controls (n=10000, mean± s.e.m., Bonferroni post-test, ***P < 0.001)

Supplement Figure 4, related to Figure 4 and S3

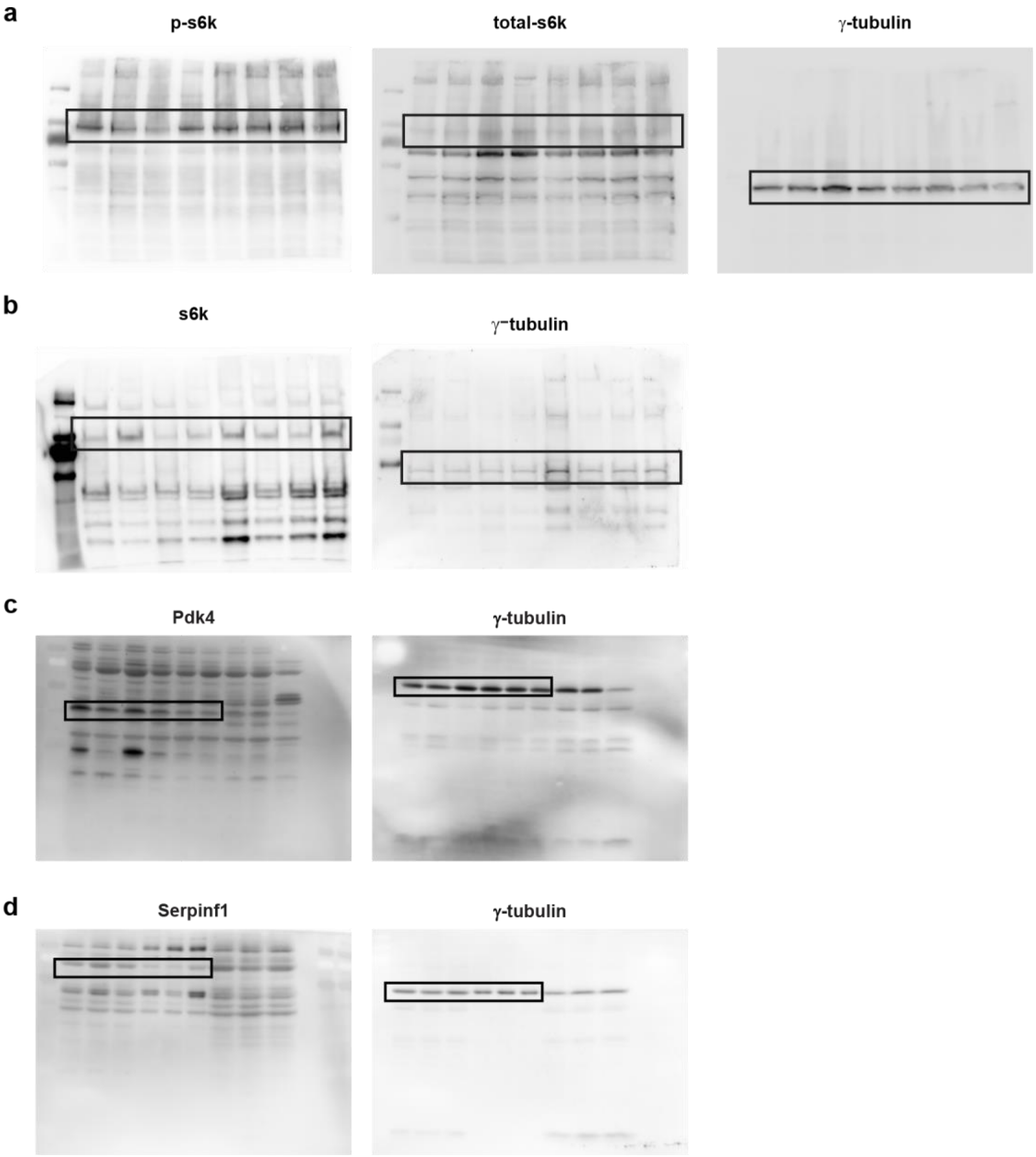

Fig. S4: Original gels for western blots indicating the cropped area. (**a**) Western Gels Figure 4g. (**b**) Western Gels Figure 4j. (**c**) Western Gels Figure S3a. (**d**) Western Gels Figure S3b.

Tables

Table S1: Proteins quantified by Mass Spectrometry (separate pdf), Excel file available

Table S2: Transcripts quantified by RNA seq (separate pdf), Excel file available

Table S3: KEGG pathway enrichment analyses (separate pdf), Excel file available

Table S4: Genes differentially regulated in hibernation relate to striated muscle growth.

Gene	OMIM	Summary	Human Relevance
RASSF1	605082	Increased expression in patients with heart failure. Larger hearts in Rassf1 knockout mice after TAC-surgery to induce pressure overload.[1]	Heart failure, cardiac hypertrophy
MYL3	16079	Essential myosin light chain. Mutations have been associated with cardiac hypertrophy and skeletal muscle disease.[2,3]	Cardiac hypertrophy, skeletal muscle disease
CYC1	123980	Component of the electron transfer chain (ETC). A mutation in CYC1 reduces protein stability in muscle of a patient with growth retardation.[4]	Skeletal myopathy, growth retardation
MYBPC1	160794	Myosin binding protein. Mutations are associated with Distal arthrogryposis type I, characterized by reduced muscle mass.[5]	Distal arthrogryposis type I, muscle atrophy
TNNI3	191044	Associated with hypertrophic cardiomyopathy.[6]	Hypertrophic cardiomyopathy
RNF11	612598	Involved in NFKB signaling.[7,8]	n.a.
PPIA	123840	Encodes Cyclophilin A, which is secreted in response to increased ROS production. Knockout mice have smaller hearts in response to high ROS levels.[9]	n.a.
PA2G4	602145	Knockouts are smaller with reduced circulating IGF-1.[10]	n.a.
RORA	600825	Involved in circadian regulation and induces Bmal1 expression,[11,12] which affects muscle function and size, increases PDK4 while reducing Pdha activity.[13]	n.a.
ARID5B	608538	Decreased genomic methylation has been associated with reduced birth weight.[14]	n.a.
NRG2	603818	Growth retardation in knockout mice.[15]	n.a.
SERPINF1	172860	Enhances NFκB signaling.[16]	n.a.

References

1. Oceandy, D. *et al.* Tumor suppressor Ras-association domain family 1 isoform A is a novel regulator of cardiac hypertrophy. *Circulation* **120,** 607–616 (2009).
2. Poetter, K. *et al.* Mutations in either the essential or regulatory light chains of myosin are associated with a rare myopathy in human heart and skeletal muscle. *Nat. Genet.* **13,** 63–69 (1996).
3. Olson, T. M., Karst, M. L., Whitby, F. G. & Driscoll, D. J. Myosin light chain mutation causes autosomal recessive cardiomyopathy with mid-cavitary hypertrophy and restrictive physiology. *Circulation* **105,** 2337–2340 (2002).
4. Gaignard, P. *et al.* Mutations in CYC1, encoding cytochrome c1 subunit of respiratory chain complex III, cause insulin-responsive hyperglycemia. *Am. J. Hum. Genet.* **93,** 384–389 (2013).
5. Gurnett, C. A. *et al.* Myosin binding protein C1: a novel gene for autosomal dominant distal arthrogryposis type 1. *Hum. Mol. Genet.* **19,** 1165–1173 (2010).
6. Kimura, A. *et al.* Mutations in the cardiac troponin I gene associated with hypertrophic cardiomyopathy. *Nat. Genet.* **16,** 379–382 (1997).
7. Shembade, N., Parvatiyar, K., Harhaj, N. S. & Harhaj, E. W. The ubiquitin-editing enzyme A20 requires RNF11 to downregulate NF-kappaB signalling. *EMBO J.* **28,** 513–522 (2009).
8. Maddirevula, S., Anuppalle, M., Huh, T.-L., Kim, S. H. & Rhee, M. Rnf11-like is a novel component of NF-κB signaling, governing the posterior patterning in the zebrafish embryos. *Biochem. Biophys. Res. Commun.* **422,** 602–606 (2012).
9. Satoh, K. *et al.* Cyclophilin A promotes cardiac hypertrophy in apolipoprotein E-deficient mice. *Arterioscler. Thromb. Vasc. Biol.* **31,** 1116–1123 (2011).
10. Zhang, Y. *et al.* Alterations in cell growth and signaling in ErbB3 binding protein-1 (Ebp1) deficient mice. *BMC Cell Biol.* **9,** 69 (2008).

11. Akashi, M. & Takumi, T. The orphan nuclear receptor RORalpha regulates circadian transcription of the mammalian core-clock Bmal1. *Nat. Struct. Mol. Biol.* **12,** 441–448 (2005).

12. Sato, T. K. *et al.* A functional genomics strategy reveals Rora as a component of the mammalian circadian clock. *Neuron* **43,** 527–537 (2004).

13. Dyar, K. A. *et al.* Muscle insulin sensitivity and glucose metabolism are controlled by the intrinsic muscle clock. *Mol Metab* **3,** 29–41 (2014).

14. Engel, S. M. *et al.* Neonatal genome-wide methylation patterns in relation to birth weight in the Norwegian Mother and Child Cohort. *Am. J. Epidemiol.* **179,** 834–842 (2014).

15. Britto, J. M. *et al.* Generation and characterization of neuregulin-2-deficient mice. *Mol. Cell. Biol.* **24,** 8221–8226 (2004).

16. Yabe, T., Sanagi, T., Schwartz, J. P. & Yamada, H. Pigment epithelium-derived factor induces pro-inflammatory genes in neonatal astrocytes through activation of NF-kappa B and CREB. *Glia* **50,** 223–234 (2005).

Supplementary table S1: Proteins quantified by Mass Spectrometry

Primary.Protein.Name	Sybmol	Protein.Description	SA_Active	A_Active	C2_Active	C1_Active	SA_Hibernation	A_Hibernation	C2_Hibernation	C1_Hibernation	p-value Activity	p-value Age	p-value Interaction
PRDX3_HUMAN	PRDX3	Thioredoxin-dependent peroxide reductase, mitochondrial OS=Homo sapiens GN=PRDX3 PE=1 SV=3	161482.2	117631.2	167560.9	149663.9	70567.58	89352.1	88619.39	96890.27	8.27E-03	2.85E-01	8.20E-01
FKBP3_HUMAN	FKBP3	Peptidyl-prolyl cis-trans isomerase FKBP3 OS=Homo sapiens GN=FKBP3 PE=1 SV=1	22601.64	24929.97	20030.69	18531.02	28560.94	25989.35	25795.3	22983.8	2.16E-02	3.52E-02	5.34E-01
NDUS2_HUMAN	NDUFS2	NADH dehydrogenase [ubiquinone] iron-sulfur protein 2, mitochondrial OS=Homo sapiens GN=NDUFS2 PE=1 SV	41785.93	43633.75	31596.21	34064.82	23385.2	19089.09	41156.4	36979.53	1.06E-02	7.77E-02	1.19E-03
G6PI_HUMAN	GPI	Glucose-6-phosphate isomerase OS=Homo sapiens GN=GPI PE=1 SV=4	540308	334053	216598.8	231263.7	534012.75	657969.12	440073.97	383743.84	4.87E-02	3.26E-02	8.25E-01
ODPB_HUMAN	PDHB	Pyruvate dehydrogenase E1 component subunit beta, mitochondrial OS=Homo sapiens GN=PDHB PE=1 SV=3	882959.9	831176.5	708099.3	884739	648777.12	592426.31	610186.25	574431.69	1.08E-02	4.15E-01	7.58E-01
LEG1_HUMAN	LGALS1	Galectin-1 OS=Homo sapiens GN=LGALS1 PE=1 SV=2	112953.4	130414.6	147095.7	143857.1	204433.64	199484.25	209957.11	172125.33	3.93E-03	5.74E-01	1.74E-01
CAZA2_HUMAN	CAPZA2	F-actin-capping protein subunit alpha-2 OS=Homo sapiens GN=CAPZA2 PE=1 SV=3	130210.4	135367.3	133938.3	112673.2	102768.62	98811.41	91443.65	94306	5.09E-03	1.96E-01	8.96E-01
CATA_HUMAN	CAT	Catalase OS=Homo sapiens GN=CAT PE=1 SV=3	23430.05	16023.12	28397.99	32902.75	45158.78	60153.01	50919.24	73116.64	1.03E-02	2.23E-01	9.17E-01
SCG2_HUMAN	SCG2	Secretogranin-2 OS=Homo sapiens GN=SCG2 PE=1 SV=2	10295.79	6845.84	9899.9	10008.21	64792.81	95403.58	18074.76	4606.31	1.23E-02	1.60E-02	1.40E-02
NDRG2_HUMAN	NDRG2	Protein NDRG2 OS=Homo sapiens GN=NDRG2 PE=1 SV=2	99327.66	145680	56727.93	64999.53	310046.12	198522.81	245779.12	228472.66	7.29E-03	2.67E-01	5.07E-01
IMA4_HUMAN	KPNA4	Importin subunit alpha-4 OS=Homo sapiens GN=KPNA4 PE=1 SV=1	3192.11	7044.49	3246.44	2809.08	10685.01	10039.32	6720.41	9596.3	1.31E-02	1.53E-01	9.65E-01
PHS_HUMAN	PCBD1	Pterin-4-alpha-carbinolamine dehydratase OS=Homo sapiens GN=PCBD1 PE=1 SV=2	16859.67	13996.25	15622.82	10919.19	51930.26	25551.42	35456.54	30619.6	3.46E-02	5.97E-01	8.08E-01
CH10_HUMAN	HSPE1	10 kDa heat shock protein, mitochondrial OS=Homo sapiens GN=HSPE1 PE=1 SV=2	58697.41	33825.93	92876.98	79033.93	29859.24	29427.82	40000.32	34480.71	1.08E-02	3.10E-02	9.12E-02
PCSK1_HUMAN	PCSK1N	ProSAAS OS=Homo sapiens GN=PCSK1N PE=1 SV=1	36593.58	25465.44	21479.35	17178.29	57192.74	55946.88	22005.87	23914.88	8.63E-03	1.72E-03	2.26E-02
IDH3G_HUMAN	IDH3G	Isocitrate dehydrogenase [NAD] subunit gamma, mitochondrial OS=Homo sapiens GN=IDH3G PE=1 SV=1	130471.7	129124.2	93678.37	125692.4	86674.2	80367.52	95099.37	103780.52	2.87E-02	8.16E-01	9.99E-02
CISY_HUMAN	CS	Citrate synthase, mitochondrial OS=Homo sapiens GN=CS PE=1 SV=2	559682.4	521152.8	386764.9	536291.4	338519.03	272612.62	415742	413649.06	2.84E-02	7.37E-01	8.87E-02
MYL1_HUMAN	MYL1	Myosin light chain 1/3, skeletal muscle isoform OS=Homo sapiens GN=MYL1 PE=1 SV=3	19800000	16800000	19300000	14800000	12300000	13100000	10400000	10700000	1.15E-02	2.82E-01	7.59E-01
PRDX2_HUMAN	PRDX2	Peroxiredoxin-2 OS=Homo sapiens GN=PRDX2 PE=1 SV=5	117750.7	83060.16	140567.3	96725.03	77721.91	60079.62	66464.82	54093.73	3.99E-02	7.64E-01	4.21E-01
DDX25_HUMAN	DDX25	ATP-dependent RNA helicase DDX25 OS=Homo sapiens GN=DDX25 PE=1 SV=2	43899.29	64585.34	60163.04	50626.61	178807.78	168174.94	32942.26	15692.56	4.44E-03	6.30E-04	5.93E-04
EFC4A_HUMAN	EFCAB4A	EF-hand calcium-binding domain-containing protein 4A OS=Homo sapiens GN=EFCAB4A PE=2 SV=3	51727.66	49037.43	25052.62	37679.91	52872.36	56552.04	66911.54	49541.64	4.68E-02	2.31E-01	1.09E-01
PIMT_HUMAN	PCMT1	Protein-L-isoaspartate(D-aspartate) O-methyltransferase OS=Homo sapiens GN=PCMT1 PE=1 SV=3	96070.42	93203.65	102912.9	95637.53	145181.7	128339.62	104427.38	93831.76	1.71E-02	3.67E-02	1.68E-02
FABP4_HUMAN	FABP4	Fatty acid-binding protein, adipocyte OS=Homo sapiens GN=FABP4 PE=1 SV=3	28765.34	30677.29	28472.39	38996.88	40048.61	44304.05	47557.81	63745.12	2.58E-02	1.53E-01	3.95E-01
MYL6_HUMAN	MYL6	Myosin light polypeptide 6 OS=Homo sapiens GN=MYL6 PE=1 SV=2	9866.83	18065.93	40008	43843.55	52482.79	60445.66	23370.03	29455.6	1.62E-02	7.72E-01	1.01E-03
VDAC3_HUMAN	VDAC3	Voltage-dependent anion-selective channel protein 3 OS=Homo sapiens GN=VDAC3 PE=1 SV=1	219563.4	197044.7	153500.3	159736.7	115057.35	151472.39	141672.42	148504.75	1.68E-02	1.43E-01	4.41E-02
F10A5_HUMAN	FAM10A5	Hsc70-interacting protein OS=Homo sapiens GN=ST13 PE=1 SV=2	13876.52	12259.02	12575.04	13518.23	9590.94	9889.98	10280.65	5703.98	2.77E-02	5.15E-01	5.24E-01
CB043_HUMAN	C2ORF43	UPF0554 protein C2orf43 OS=Homo sapiens GN=C2orf43 PE=1 SV=1	1413.13	1352.48	970.71	2384.92	22786.84	12429.68	5153.13	9597.34	1.81E-02	1.55E-01	1.37E-01
CCD96_HUMAN	CCDC96	Coiled-coil domain-containing protein 96 OS=Homo sapiens GN=CCDC96 PE=2 SV=2	16077.2	24213.93	23040.3	24020.6	43510.77	28471.25	46970.86	35000.03	3.33E-02	4.67E-01	8.85E-01
ENASE_HUMAN	ENGASE	Cytosolic endo-beta-N-acetylglucosaminidase OS=Homo sapiens GN=ENGASE PE=1 SV=1	115845.8	138507.7	141434.2	50537.57	44387.34	57613.31	31257.64	28511.6	3.97E-02	3.31E-01	8.42E-01
AT2A1_HUMAN	ATP2A1	Sarcoplasmic/endoplasmic reticulum calcium ATPase 1 OS=Homo sapiens GN=ATP2A1 PE=1 SV=1	1052874	791724.6	1333994	1777811	1905199.75	1814284.38	1761723.62	1758783.75	1.20E-02	1.11E-01	4.86E-02
FIS1_HUMAN	FIS1	Mitochondrial fission 1 protein OS=Homo sapiens GN=FIS1 PE=1 SV=2	11432.94	10405.06	11430.33	11577.01	5367.46	7094.86	7874.06	9149.79	2.99E-03	7.41E-02	2.28E-01
RTN4_HUMAN	RTN4	Reticulon-4 OS=Homo sapiens GN=RTN4 PE=1 SV=2	0	1472.39	45.96	1324.23	6161.91	6398.69	7817.02	4466.88	4.79E-03	9.27E-01	9.66E-01
PSRC1_HUMAN	PSRC1	Proline/serine-rich coiled-coil protein 1 OS=Homo sapiens GN=PSRC1 PE=1 SV=1	99196.68	56663	69215.64	69751.98	150460.11	213894.72	86944.2	76834.37	3.88E-02	4.77E-02	7.56E-02
PTRF_HUMAN	PTRF	Polymerase I and transcript release factor OS=Homo sapiens GN=PTRF PE=1 SV=1	92484.83	96249.03	145830.5	134082.6	162310.53	157980.69	156844.31	126434.45	1.51E-02	1.77E-01	1.80E-02
TUT4_HUMAN	ZCCHC11	Terminal uridylyltransferase 4 OS=Homo sapiens GN=ZCCHC11 PE=1 SV=3	3485.64	1965.11	1059.26	3354.05	13971.58	14367.67	4036.45	4975.49	7.24E-04	2.27E-03	3.38E-03
KTN1_HUMAN	KTN1	Kinectin OS=Homo sapiens GN=KTN1 PE=1 SV=1	686113	622867.9	414222.6	366020.3	324159.72	372393.5	541222.31	431480.91	4.33E-02	1.54E-01	5.01E-03
ACDSB_HUMAN	ACADSB	Short/branched chain specific acyl-CoA dehydrogenase, mitochondrial OS=Homo sapiens GN=ACADSB PE=1 SV=	12806.95	9074.1	12156.56	13711.28	4862.79	4568.47	7679.88	7766.24	4.87E-03	6.92E-02	6.43E-01
ARHL1_HUMAN	ADPRHL1	[Protein ADP-ribosylarginine] hydrolase-like protein 1 OS=Homo sapiens GN=ADPRHL1 PE=2 SV=1	7772.28	16364.2	6731.52	5205.5	19226.99	22085.64	18242.13	25939.86	1.46E-02	4.80E-01	2.77E-01
SMTL2_HUMAN	SMTNL2	Smoothelin-like protein 2 OS=Homo sapiens GN=SMTNL2 PE=2 SV=1	12565.86	13213.04	10634.41	10279.91	12265.48	15741.55	15422.33	15251.09	2.81E-02	5.70E-01	1.02E-01
COBA1_HUMAN	COL11A1	Collagen alpha-1(XI) chain OS=Homo sapiens GN=COL11A1 PE=1 SV=4	30658.44	20102.91	19383.15	12885.2	49773.15	62450.56	24964.18	34532.85	1.17E-02	2.41E-02	1.65E-01
SPTA1_HUMAN	SPTA1	Spectrin alpha chain, erythrocyte OS=Homo sapiens GN=SPTA1 PE=1 SV=5	253072.4	282644.8	280094.7	281860.4	227800.38	193804.16	161629.3	144318.53	1.56E-03	1.38E-01	4.25E-02
DYH1_HUMAN	DNAH1	Dynein heavy chain 1, axonemal OS=Homo sapiens GN=DNAH1 PE=1 SV=3	6076.74	9760.84	5625.76	4335.17	28401.31	15324.84	12676.95	10119.19	4.27E-02	1.26E-01	3.39E-01
CDT1_HUMAN	CDT1	DNA replication factor Cdt1 OS=Homo sapiens GN=CDT1 PE=1 SV=3	45133.06	39697.41	48886.72	27221.96	9397.92	18167.42	21583.74	26855.44	2.59E-02	6.47E-01	2.95E-01
NDUV2_HUMAN	NDUFV2	NADH dehydrogenase [ubiquinone] flavoprotein 2, mitochondrial OS=Homo sapiens GN=NDUFV2 PE=1 SV=2	139433.5	119499	117239.4	151396.2	96750.62	88240.38	92683.93	100399.34	2.22E-02	6.88E-01	9.71E-01
ODO1_HUMAN	OGDH	2-oxoglutarate dehydrogenase, mitochondrial OS=Homo sapiens GN=OGDH PE=1 SV=3	32278.37	33397.12	36475.68	41826.09	47022.91	33593.92	52281.12	54463.41	4.16E-02	5.74E-02	4.09E-01
MYOTI_HUMAN	MYOT	Myotilin OS=Homo sapiens GN=MYOT PE=1 SV=2	673353.7	624426.4	490307.7	509920.3	529387.69	544973.69	397983.94	385270.72	1.45E-03	4.77E-04	9.15E-01
SYP2L_HUMAN	SYNPO2L	Synaptopodin 2-like protein OS=Homo sapiens GN=SYNPO2L PE=2 SV=3	15355.29	15519.73	12348.15	11052.97	13730.26	12928.61	8845.33	10968.15	4.07E-02	5.44E-03	8.22E-01
RYR1_HUMAN	RYR1	Ryanodine receptor 1 OS=Homo sapiens GN=RYR1 PE=1 SV=3	16148.49	20563.86	21727.1	32029.73	101922.93	59400.12	51733.75	56890.32	1.54E-02	4.66E-01	1.90E-01
TNNI2_HUMAN	TNNI2	Troponin I, fast skeletal muscle OS=Homo sapiens GN=TNNI2 PE=1 SV=2	723151.6	714041.9	693127.4	888247.6	1655677.12	1585815.88	1527300.12	1144804.88	2.65E-03	3.84E-01	1.76E-01
EF1B_HUMAN	EEF1B2	Elongation factor 1-beta OS=Homo sapiens GN=EEF1B2 PE=1 SV=3	19381.49	18601.03	36636.59	24976.73	16825.64	13236.7	9474.02	11902.54	1.81E-02	2.97E-01	6.05E-02
RTN2_HUMAN	RTN2	Reticulon-2 OS=Homo sapiens GN=RTN2 PE=2 SV=1	17542.18	20383.17	22147.79	35011.79	35113.17	39788.66	45249.31	39613.41	1.27E-02	1.25E-01	5.72E-01
FKB1A_HUMAN	FKBP1A	Peptidyl-prolyl cis-trans isomerase FKBP1A OS=Homo sapiens GN=FKBP1A PE=1 SV=2	51660.48	53723.01	64943.68	60083.86	59329.43	74807.88	72512.17	83894.6	3.92E-02	1.03E-01	9.02E-01
PGBM_HUMAN	HSPG2	Basement membrane-specific heparan sulfate proteoglycan core protein OS=Homo sapiens GN=HSPG2 PE=1 SV	60781.3	76740.98	109447.9	103933.8	57611.2	63624.4	69651.62	71806.53	8.12E-03	6.00E-03	3.69E-02
GRP75_HUMAN	HSPA9	Stress-70 protein, mitochondrial OS=Homo sapiens GN=HSPA9 PE=1 SV=2	243975.7	177778.6	214600.7	234231.2	173748.83	116564.12	145562.7	160931.14	3.96E-02	6.59E-01	9.10E-01
PDK4_HUMAN	PDK4	[Pyruvate dehydrogenase [lipoamide]] kinase isozyme 4, mitochondrial OS=Homo sapiens GN=PDK4 PE=1 SV=1	1127.26	1015.81	2369.22	1477.09	10458.72	6991.83	5902.68	8386.35	4.11E-03	7.55E-01	3.27E-01
COEA1_HUMAN	COL14A1	Collagen alpha-1(XIV) chain OS=Homo sapiens GN=COL14A1 PE=1 SV=3	798.66	629.76	2987.04	2869.66	638.45	496.7	2199.91	1537.87	2.71E-02	5.76E-04	6.13E-02
ATPB_HUMAN	ATP5B	ATP synthase subunit beta, mitochondrial OS=Homo sapiens GN=ATP5B PE=1 SV=3	1.20E+07	12300000	13100000	15200000	10900000	7530017.5	9095162	10700000	2.86E-02	2.80E-01	5.73E-01
CO6A2_HUMAN	COL6A2	Collagen alpha-2(VI) chain OS=Homo sapiens GN=COL6A2 PE=1 SV=4	73833.06	164873.8	566083.4	600132.4	143777.53	174516.39	126358.59	192641.38	3.22E-03	1.58E-03	1.59E-03
IMMT_HUMAN	IMMT	Mitochondrial inner membrane protein OS=Homo sapiens GN=IMMT PE=1 SV=1	127362.8	100235.7	122305.4	148014.7	77270.8	70718.6	89592.53	85011.62	1.01E-02	1.44E-01	6.95E-01
PADI2_HUMAN	PADI2	Protein-arginine deiminase type-2 OS=Homo sapiens GN=PADI2 PE=1 SV=2	105572.4	226211.3	117483	71194.74	422468.06	317345.25	301617.31	196288.17	2.20E-02	1.22E-01	6.42E-01
CX7A2_HUMAN	COX7A2	Cytochrome c oxidase subunit 7A2, mitochondrial OS=Homo sapiens GN=COX7A2 PE=1 SV=1	16876.78	18510.79	21030.62	16668.03	10117.32	9571.23	7958.94	13511.79	1.18E-02	6.03E-01	9.45E-01
NDUA4_HUMAN	NDUFA4	NADH dehydrogenase [ubiquinone] 1 alpha subcomplex subunit 4 OS=Homo sapiens GN=NDUFA4 PE=1 SV=1	515104	268563.5	403316	522566	153482.78	188780	292588.38	255065.17	4.23E-02	2.80E-01	8.32E-01
MDHM_HUMAN	MDH2	Malate dehydrogenase, mitochondrial OS=Homo sapiens GN=MDH2 PE=1 SV=3	2790477	2257113	2507962	2320461	1250094.62	1336394.12	1155369.25	1529370.38	2.53E-03	8.68E-01	6.66E-01
PSB1_HUMAN	PSMB1	Proteasome subunit beta type-1 OS=Homo sapiens GN=PSMB1 PE=1 SV=2	16576.93	15443.4	21467.96	18412.78	12422.07	16219.34	11601.23	12004.94	1.73E-02	6.04E-01	6.21E-02
ATPA_HUMAN	ATP5A1	ATP synthase subunit alpha, mitochondrial OS=Homo sapiens GN=ATP5A1 PE=1 SV=1	8473472	8145147	8549903	9711337	5713252	4929059	5429723.5	6251554.5	1.63E-03	1.81E-01	7.34E-01
ETFD_HUMAN	ETFDH	Electron transfer flavoprotein-ubiquinone oxidoreductase, mitochondrial OS=Homo sapiens GN=ETFDH PE=1 SV	46836.37	39586.99	49913.91	52869.98	47958.79	53539.74	79488.84	78169.69	1.96E-03	1.71E-03	1.48E-02
ACTN2_HUMAN	ACTN2	Alpha-actinin-2 OS=Homo sapiens GN=ACTN2 PE=1 SV=1	25300000	25500000	18400000	18600000	21400000	20600000	19100000	16600000	1.87E-02	1.60E-03	4.68E-02
NDUBA_HUMAN	NDUFB10	NADH dehydrogenase [ubiquinone] 1 beta subcomplex subunit 10 OS=Homo sapiens GN=NDUFB10 PE=1 SV=3	60523.41	61783.97	62381.68	66535.12	42476.53	34580.28	42326.98	46553.02	1.02E-03	1.38E-01	6.28E-01
KCRM_HUMAN	CKM	Creatine kinase M-type OS=Homo sapiens GN=CKM PE=1 SV=2	58100000	49900000	50600000	32800000	36900000	34200000	33500000	30400000	4.80E-02	1.87E-01	4.34E-01
CCD19_HUMAN	CCDC19	Coiled-coil domain-containing protein 19, mitochondrial OS=Homo sapiens GN=CCDC19 PE=1 SV=2	3198.8	3617.35	1828.86	1802.21	5227.41	3070.47	4709.77	5007.15	2.70E-02	4.70E-01	1.06E-01
PER3_HUMAN	PER3	Period circadian protein homolog 3 OS=Homo sapiens GN=PER3 PE=2 SV=4	9017.07	13126	9616.02	12264.14	6892.34	7321.59	6806.62	5159.53	2.61E-02	6.53E-01	7.21E-01
MAGB3_HUMAN	MAGEB3	Melanoma-associated antigen B3 OS=Homo sapiens GN=MAGEB3 PE=2 SV=2	6656.03	5577.3	4452.97	5661.76	7538.1	8275.01	7385.33	8192.3	9.83E-03	2.95E-01	3.90E-01
CC141_HUMAN	CCDC141	Coiled-coil domain-containing protein 141 OS=Homo sapiens GN=CCDC141 PE=1 SV=1	4090.56	3069.83	3504.18	3808.44	5005.22	3689.46	5193.64	6392.18	4.88E-02	2.16E-01	2.57E-01
ZN217_HUMAN	ZNF217	Zinc finger protein 217 OS=Homo sapiens GN=ZNF217 PE=1 SV=1	14070.05	14515.55	12750.86	15555.27	57379.12	37592.15	15337.21	13746.26	2.86E-02	2.99E-02	3.07E-02
CO6A1_HUMAN	COL6A1	Collagen alpha-1(VI) chain OS=Homo sapiens GN=COL6A1 PE=1 SV=3	65006.39	181787	680060.1	603769.1	134826.75	206203.11	140703.67	204222.92	7.51E-03	3.53E-03	3.63E-03
NDUS8_HUMAN	NDUFS8	NADH dehydrogenase [ubiquinone] iron-sulfur protein 8, mitochondrial OS=Homo sapiens GN=NDUFS8 PE=1 SV	84417.64	74086.82	44546.36	67249.67	31627.98	32472.41	40566.8	51379.82	1.37E-02	5.26E-01	5.18E-02
TBB4B_HUMAN		Tubulin beta-4B chain OS=Homo sapiens GN=TUBB4B PE=1 SV=1	77201.75	72316.61	104149.7	68755.41	125456.91	124423.55	95356.99	98293.39	2.79E-02	4.12E-01	9.06E-02
TBB4A_HUMAN		Tubulin beta-4A chain OS=Homo sapiens GN=TUBB4A PE=1 SV=2	21391.38	22208.4	28345.93	27211.14	34440.73	32079.96	24257.79	25181.3	4.38E-03	1.51E-01	5.55E-04
CO6A3_HUMAN	COL6A3	Collagen alpha-3(VI) chain OS=Homo sapiens GN=COL6A3 PE=1 SV=5	190072	478534.4	1690901	1773901	708975	598935.38	493008.84	674918.44	1.08E-02	1.95E-03	1.34E-03
ODPA_HUMAN	PDHA1	Pyruvate dehydrogenase E1 component subunit alpha, somatic form, mitochondrial OS=Homo sapiens GN=PDH	398792.5	373177.1	251705.2	286393.3	257198.12	256084.2	268417.88	302613.34	1.48E-02	3.29E-02	6.10E-03
PRELP_HUMAN	PRELP	Prolargin OS=Homo sapiens GN=PRELP PE=1 SV=1	36423.09	54790.64	180538	238607.3	37248.51	75372.29	39861.42	95230.78	4.43E-02	1.81E-02	2.81E-02
COCA1_HUMAN	COL12A1	Collagen alpha-1(XII) chain OS=Homo sapiens GN=COL12A1 PE=1 SV=2	8829.23	28312.71	117737.2	153193.5	6859.69	5323.95	2586.67	20478.57	3.52E-03	5.23E-03	7.30E-03
WDR72_HUMAN	WDR72	WD repeat-containing protein 72 OS=Homo sapiens GN=WDR72 PE=1 SV=2	21545.82	19208.78	19911.85	18588.66	33836.48	24894.72	41825.19	47660.34	3.31E-03	6.08E-02	4.00E-02
GAMT_HUMAN	GAMT	Guanidinoacetate N-methyltransferase OS=Homo sapiens GN=GAMT PE=1 SV=1	22479.11	35378.05	17562.06	19248.87	61566.05	35817.04	53587.23	48489.89	2.32E-02	6.06E-01	4.29E-01
SAHH_HUMAN	AHCY	Adenosylhomocysteinase OS=Homo sapiens GN=AHCY PE=1 SV=4	154853.3	139749.9	138277.6	132174.8	164757.56	224428.17	204419.91	203832.81	1.98E-02	9.38E-01	5.23E-01
CAPZB_HUMAN	CAPZB	F-actin-capping protein subunit beta OS=Homo sapiens GN=CAPZB PE=1 SV=4	207157.4	196106.4	145693.3	205075.5	346236.5	276824.41	222281	260294.47	2.42E-02	1.25E-01	4.27E-01
H2A1B_HUMAN	HIST1H2AB	Histone H2A type 1-B/E OS=Homo sapiens GN=HIST1H2AB PE=1 SV=2	62886.6	75575.82	146705.8	118743.3	54107.68	52392.45	92705.05	62381.59	3.01E-02	1.53E-02	1.44E-01
PURA1_HUMAN	ADSSL1	Adenylosuccinate synthetase isozyme 1 OS=Homo sapiens GN=ADSSL1 PE=1 SV=1	42151.29	55936.35	38166.58	32867.15	22839.18	26874.77	20731.7	24909.18	9.67E-03	1.21E-01	2.21E-01
HSPB7_HUMAN	HSPB7	Heat shock protein beta-7 OS=Homo sapiens GN=HSPB7 PE=1 SV=1	7662.73	10527.18	8942.32	8819.64	19911.92	20408	4968.53	4161.18	1.11E-02	4.70E-04	5.23E-04
UN13A_MOUSE	UNC13A	Protein unc-13 homolog A OS=Mus musculus GN=Unc13a PE=1 SV=2	243707.3	294313.1	204469	189365.7	313933.16	343286.84	273217.69	305366.56	1.13E-02	3.12E-02	3.92E-01
HBAZ_HUMAN	HBZ	Hemoglobin subunit alpha OS=Homo sapiens GN=HBA1 PE=1 SV=2	1358532	320774.2	1497860	1417344	301556.88	168163.05	90129.71	445700.31	3.17E-02	3.05E-01	3.51E-01
XIRP2_HUMAN	XIRP2	Xin actin-binding repeat-containing protein 2 OS=Homo sapiens GN=XIRP2 PE=1 SV=2	36937.05	29136.4	30115.09	21738.21	51347.46	58720.58	46433.01	59021.52	6.24E-03	3.67E-01	6.32E-01
UGPA_HUMAN	UGP2	UTP--glucose-1-phosphate uridylyltransferase OS=Homo sapiens GN=UGP2 PE=1 SV=5	147865.1	142662.9	139310.3	118896.9	113400.94	133880.28	90057.08	110316.33	4.72E-02	9.06E-02	7.04E-01
B3A2_HUMAN	SLC4A2	Anion exchange protein 2 OS=Homo sapiens GN=SLC4A2 PE=1 SV=4	6046.85	3478.41	5327.88	5996.12	11388.25	7127.39	9915.64	9827.18	2.56E-02	5.79E-01	9.15E-01
RAB1A_HUMAN	RAB1A	Ras-related protein Rab-1A OS=Homo sapiens GN=RAB1A PE=1 SV=3	3831.3	3248.75	7131.34	7458.77	5431.73	3520.81	2841.07	3287.46	3.36E-02	8.66E-02	7.57E-03
SYPL2_HUMAN	SYPL2	Synaptophysin-like protein 2 OS=Homo sapiens GN=SYPL2 PE=2 SV=1	3442.1	4885.6	3650.33	1767.49	6779.11	8971.26	14179.17	11368.88	3.00E-03	1.83E-01	4.12E-02
TNNC2_HUMAN	TNNC2	Troponin C, skeletal muscle OS=Homo sapiens GN=TNNC2 PE=1 SV=2	7760365	6282528	5936430	6709298	3914490.75	6643076.5	3092347.75	3707722	4.57E-02	1.89E-01	5.09E-01
SMYD1_HUMAN	SMYD1	SET and MYND domain-containing protein 1 OS=Homo sapiens GN=SMYD1 PE=2 SV=1	17086.95	22569.37	15226.23	19315.71	35824.5	29791.02	32281.32	35618.93	3.64E-03	7.85E-01	4.89E-01
GRAK_HUMAN	GZMK	Granzyme K OS=Homo sapiens GN=GZMK PE=1 SV=1	20037.19	57337.91	169709.7	153279.3	39936.13	62292.71	36682.99	55576.36	1.48E-02	9.34E-03	7.02E-03
LAP2_HUMAN	ERBB2IP	Protein LAP2 OS=Homo sapiens GN=ERBB2IP PE=1 SV=2	19775.14	26445.89	23056.88	17980.93	53587.54	53516.31	35225.1	42271.23	8.85E-04	3.36E-02	8.96E-02
PDLI1_HUMAN	PDLIM1	PDZ and LIM domain protein 1 OS=Homo sapiens GN=PDLIM1 PE=1 SV=4	16935.16	31114.95	44154.39	27196.95	7682.66	11501.79	11592.46	10005.49	2.50E-02	3.17E-01	4.06E-01
BEST2_HUMAN	BEST2	Bestrophin-2 OS=Homo sapiens GN=BEST2 PE=2 SV=1	11042.38	6560.7	7963.9	8947.88	13360.32	11561.76	13047.08	15992.42	2.76E-02	5.83E-01	4.49E-01
1433G_HUMAN	YWHAG	14-3-3 protein gamma OS=Homo sapiens GN=YWHAG PE=1 SV=2	490992.3	546536.3	506896.8	385081.4	962795.56	881695.06	489930.84	464423.78	5.39E-03	2.84E-03	9.34E-03
HBB_HUMAN	HBB	Hemoglobin subunit beta OS=Homo sapiens GN=HBB PE=1 SV=2	22500000	6444321	18500000	15700000	3640408.5	3507200.5	1294718.25	5578771.5	4.35E-02	7.82E-01	7.59E-01
PSA_HUMAN	NPEPPS	Puromycin-sensitive aminopeptidase OS=Homo sapiens GN=NPEPPS PE=1 SV=2	45805.86	52450.69	32816.25	28485.1	50653.89	54618.85	54845	47694.74	1.33E-02	2.53E-02	3.98E-02
BIG1_HUMAN	ARFGEF1	Brefeldin A-inhibited guanine nucleotide-exchange protein 1 OS=Homo sapiens GN=ARFGEF1 PE=1 SV=2	2036.96	7627.63	6839.42	4822.09	17551.8	12479.8	22639.56	10198.99	4.74E-02	7.60E-01	9.59E-01
FUMH_HUMAN	FH	Fumarate hydratase, mitochondrial OS=Homo sapiens GN=FH PE=1 SV=3	377990.1	330944.1	318848.1	395766.7	262544.53	237807.83	266379.62	280802.19	1.65E-02	6.08E-01	6.86E-01
ACADL_MOUSE	ACADL	Long-chain specific acyl-CoA dehydrogenase, mitochondrial OS=Mus musculus GN=Acadl PE=2 SV=2 [MASS=479	16785.32	10962.77	18289.09	23081.75	11297.48	7087.12	13236.98	8672.72	4.19E-02	1.54E-01	3.60E-01
AMACR_MOUSE	AMACR	Alpha-methylacyl-CoA racemase OS=Mus musculus GN=Amacr PE=1 SV=3	4825.34	5850.04	6689.55	7207.04	1991.35	1604.08	3675	3194.62	4.13E-04	7.58E-03	9.70E-01
APC5_MOUSE	ANAPC5	Anaphase-promoting complex subunit 5 OS=Mus musculus GN=Anapc5 PE=2 SV=1	32582.17	23408.79	31583.86	23159.4	14963.77	16391.36	20519.95	13871.21	3.38E-02	9.06E-01	7.78E-01
ATP5I_MOUSE	GM2972	ATP synthase subunit e, mitochondrial OS=Mus musculus GN=Atp5i PE=1 SV=2 [MASS=8236]	233058.8	177384.8	278662.8	241948.3	136250.22	183044.06	136338.19	171151.98	2.64E-02	3.29E-01	2.41E-01
CAD23_MOUSE	CDH23	Cadherin-23 OS=Mus musculus GN=Cdh23 PE=1 SV=2	76838.55	53320.96	68919	65265.97	30184.6	37879.13	27546.24	30624.57	5.40E-03	8.27E-01	6.10E-01
CX7A1_MOUSE	COX7A1	Cytochrome c oxidase polypeptide 7A1, mitochondrial OS=Mus musculus GN=Cox7a1 PE=2 SV=1 [MASS=8986]	269221.3	261949.9	266279.9	335000.8	147616.64	176002.03	192863.95	209110.08	6.00E-03	1.24E-01	9.19E-01
DHSB_HUMAN	SDHB	Succinate dehydrogenase [ubiquinone] iron-sulfur subunit, mitochondrial OS=Homo sapiens GN=SDHB PE=1 SV	169123.9	165080.9	145967.3	153742.8	99966.73	117724.38	126115.77	115646.16	1.47E-03	6.66E-01	5.91E-02
ECH1_MOUSE	ECH1	Delta(3,5)-Delta(2,4)-dienoyl-CoA isomerase, mitochondrial OS=Mus musculus GN=Ech1 PE=2 SV=1 [MASS=361	10582.01	8843.54	10775.99	12182.15	6296.61	6805.21	3822.45	7800.67	1.84E-02	6.78E-01	3.36E-01
EFCB3_MOUSE	EFCAB3	EF-hand calcium-binding domain-containing protein 3 OS=Mus musculus GN=Efcab3 PE=2 SV=1	3376.36	2665.86	3083.37	4865.56	7415.63	9495.17	3891.24	3383.48	2.39E-02	5.47E-02	1.59E-02
EPIPL_MOUSE	EPPK1	Epiplakin OS=Mus musculus GN=Eppk1 PE=1 SV=2	26422.82	29655.03	32479.38	34727.12	34782.62	33179.41	43305.38	39103.8	1.06E-02	1.29E-02	6.08E-01
EST1E_MOUSE		Carboxylesterase 1E OS=Mus musculus GN=Ces1e PE=1 SV=1	5830.35	6301.81	9974.71	8491.91	5330.9	4248.81	4760.54	4221.19	3.63E-03	4.36E-02	2.45E-02
H2A1F_MOUSE	HIST1H2AF	Histone H2A type 2-C OS=Mus musculus GN=Hist2h2ac PE=1 SV=3 [MASS=13988]	63016.19	75234.9	148158.5	118825	54300.59	53660.48	90905.33	61539.68	2.87E-02	1.61E-02	1.23E-01
H2B3A_MOUSE	HIST3H2BA	Histone H2B type 2-E OS=Mus musculus GN=Hist2h2be PE=1 SV=3	53646.39	53088.83	68128.62	50704.07	89135.25	76567.61	102062.07	79973.78	1.66E-02	4.08E-01	8.97E-01
HINT1_MOUSE	HINT1	Histidine triad nucleotide-binding protein 1 OS=Mus musculus GN=Hint1 PE=1 SV=3	19422.39	12348.64	17468.66	11342.95	24914.95	30698.16	17918.72	21153.44	4.10E-02	1.64E-01	3.02E-01
IDHG1_MOUSE		Isocitrate dehydrogenase [NAD] subunit gamma 1, mitochondrial OS=Mus musculus GN=Idh3g PE=1 SV=1	130658.5	128567.3	94697.52	125774.3	86929.58	82283.38	93253.13	102477.18	2.50E-02	7.28E-01	1.18E-01
KAD2_MOUSE	AK2	Adenylate kinase 2, mitochondrial OS=Mus musculus GN=Ak2 PE=1 SV=5 [MASS=26469]	2216.9	2208.99	892.39	795.98	12754.97	10235.11	3917.13	1879.87	2.20E-03	3.55E-03	1.12E-02
MNS1_MOUSE	MNS1	Meiosis-specific nuclear structural protein 1 OS=Mus musculus GN=Mns1 PE=1 SV=1	907.86	977.89	1307.95	1216.15	4280.97	2792.53	1140.37	1429.24	2.63E-02	6.38E-02	2.77E-02
MYH14_MOUSE	MYH14	Myosin-14 OS=Mus musculus GN=Myh14 PE=1 SV=1	17255.46	26748.38	21660	27319.25	45509.61	52399.23	36449.34	27061.44	1.30E-02	1.41E-01	7.03E-02
NID2_MOUSE	NID2	Nidogen-1 OS=Mus musculus GN=Nid1 PE=1 SV=2	18245.42	16114.4	20276.45	14879.73	6421.04	7768.4	8074.81	10920.69	5.32E-03	4.44E-01	5.76E-01
OXYR_MOUSE	OXTR	Oxytocin receptor OS=Mus musculus GN=Oxtr PE=2 SV=1	9299325	3450508	10800000	10100000	2671264.5	2446150.5	1243784	3490546.25	1.96E-02	2.86E-01	2.48E-01
PADI1_MOUSE	PADI1	Protein-arginine deiminase type-1 OS=Mus musculus GN=Padi1 PE=1 SV=1	14847.62	29746.62	17984.55	10329.66	52005.8	56701.36	50956.91	31532.09	1.05E-02	1.78E-01	7.22E-01
QCR6_MOUSE	UQCRH	Cytochrome b-c1 complex subunit 6, mitochondrial OS=Mus musculus GN=Uqcrh PE=1 SV=2 [MASS=10435]	301458.2	452340.2	545436.1	497759.7	251224.8	259356.97	310963.97	312196.5	1.39E-02	6.42E-02	3.27E-01
RBM3_MOUSE	RBM3	Putative RNA-binding protein 3 OS=Mus musculus GN=Rbm3 PE=2 SV=1	1176.72	3667.79	2830.53	908.12	20980.13	16199.62	12967.66	11662.6	8.20E-04	8.06E-02	1.23E-01
RRP5_MOUSE	PDCD11	Protein RRP5 homolog OS=Mus musculus GN=Pdcd11 PE=2 SV=2	5260.86	18152.37	127676.8	124006.5	34173.78	22249.87	24065.81	31327.29	1.08E-03	3.01E-04	2.91E-04
S26A8_MOUSE	SLC26A8	Testis anion transporter 1 OS=Mus musculus GN=Slc26a8 PE=2 SV=2 [MASS=112977]	54412.51	55586.12	55544.97	57609.08	39180.18	33063.21	38131.22	42776.3	9.58E-04	2.15E-01	5.31E-01
SPT5H_MOUSE	SUPT5H	Transcription elongation factor SPT5 OS=Mus musculus GN=Supt5h PE=1 SV=1	221680.8	228466.1	162790.8	148386.5	143999.69	155221.06	174897	148474.19	1.34E-02	2.49E-02	7.66E-03
TOR4A_MOUSE		Torsin-4A OS=Mus musculus GN=Tor4a PE=2 SV=1	32359.91	29188.81	25247	38217.87	20357.53	13535.82	19577.86	17889.52	2.37E-02	7.34E-01	9.18E-01
YR010_HUMAN		Putative uncharacterized protein ENSP00000344348 OS=Homo sapiens PE=2 SV=2	18955.33	15847.3	9569.23	12950.64	22430.49	19462.34	18081.16	17343.83	2.23E-02	2.73E-02	3.51E-01

Supplementary table 2: Transcripts quantified by RNA seq

id	SA_Hibernation	SA_Active	A_Hibernation	A_Active	gene_length	sign_hibernation	ids	Ensembl.gene.id	Ensembl.transcript.id	Official.gene.symbol
contig00011	36.304	14.355	34.068	24.151	2575	0.830878485	isogroup00001	ENSG00000150938	ENST00000280527	CRIM1
contig00016	4127.913	2344.621	3766.795	3053.738	1165	0.710246487	isogroup00001	ENSG00000239474	ENST00000284669	KBTBD10
contig00021	86.588	108.911	228.803	214.629	1546	0.122830089	isogroup00001	ENSG00000155657	ENST00000342992	TTN
contig00025	4.814	16.077	14.088	9.02	14670	0.507777861	isogroup00001	ENSG00000155657	ENST00000342992	TTN
contig00027	7.025	6.261	4.888	4.555	1666	0.200740212	isogroup00001	ENSG00000021574	ENST00000315285	SPAST
contig00029	24.604	45.987	56.051	57.728	1125	0.526010746	isogroup00001	ENSG00000155657	ENST00000342992	TTN
contig00031	36.139	64.655	10.605	7.786	11702	0.49669347	isogroup00001	ENSG00000155657	ENST00000460472	TTN
contig00032	6.337	17.965	10.536	2.816	6407	0.149705042	isogroup00001	ENSG00000155657	ENST00000460472	TTN
contig00033	10.317	23.663	9.897	2.901	7640	0.208151725	isogroup00001	ENSG00000155657	ENST00000460472	TTN
contig00034	197.213	309.853	26.145	101.762	7912	0.771342151	isogroup00001	ENSG00000155657	ENST00000460472	TTN
contig00038	14.261	29.399	8.909	2.845	3368	0.282417149	isogroup00001	ENSG00000155657	ENST00000460472	TTN
contig00042	27.845	16.097	26.611	17.014	1471	0.766006613	isogroup00001	ENSG00000240563	ENST00000498273	L1TD1
contig00044	1.026	1.377	1.136	1.182	1382	0.208612009	isogroup00001	ENSG00000159147	ENST00000303113	DONSON
contig00048	3213.91	1780.434	2876.17	2418.046	652	0.696184339	isogroup00001	ENSG00000239474	ENST00000284669	KBTBD10
contig00055	8.654	10.738	7.465	6.448	1170	0.27086308	isogroup00001	ENSG00000162704	ENST00000359856	ARPC5
contig00060	354.57	339.741	408.674	333.299	814	0.2497088	isogroup00001	ENSG00000213585	ENST00000395044	VDAC1
contig00063	363.047	343.328	384.894	349.216	509	0.18709138	isogroup00001	ENSG00000213585	ENST00000425992	VDAC1
contig00066	52.616	53.899	40.109	40.386	4692	0.056868565	isogroup00001	ENSG00000165410	ENST00000555765	CFL2
contig00071	46.675	48.528	46.983	59.606	6299	0.37314947	isogroup00001	ENSG00000155657	ENST00000360870	TTN
contig00073	16.489	31.11	18.919	15.963	7807	0.511300443	isogroup00001	ENSG00000155657	ENST00000460472	TTN
contig00074	3.031	2.918	3.022	2.798	672	0.059555121	isogroup00001	ENSG00000029363	ENST00000529826	BCLAF1
contig00087	18.813	29.806	24.749	19.974	1707	0.323476366	isogroup00001	ENSG00000155657	ENST00000460472	TTN
contig00089	8756.491	11393.546	8789.513	9418.372	513	0.461467649	isogroup00001	ENSG00000198886	ENST00000361381	MT-ND4
contig00098	5.592	5.112	5.547	8.936	1225	0.161569099	isogroup00001	ENSG00000104312	ENST00000220751	RIPK2
contig00100	2.813	2.363	2.314	3.5	838	0.05706583	isogroup00001	ENSG00000104312	ENST00000540020	RIPK2
contig00105	28.454	12.065	17.362	12.89	1808	0.828905839	isogroup00001	ENSG00000150938	ENST00000280527	CRIM1
contig00107	5.521	5.379	6.706	4.097	2109	0.340948373	isogroup00001	ENSG00000137098	ENST00000497810	SPAG8
contig00113	27.459	19.733	40.723	42.74	1317	0.118621778	isogroup00001	ENSG00000128272	ENST00000404241	ATF4
contig00120	12.542	12.014	14.25	10.919	3137	0.335237093	isogroup00001	ENSG00000102910	ENST00000285737	LONP2
contig00125	4.075	3.376	3.566	3.489	2926	0.15215676	isogroup00001	ENSG00000143363	ENST00000271620	PRUNE
contig00170	45.283	73.894	161.969	162.339	815	0.169337567	isogroup00001	ENSG00000221855	ENST00000408916	TAS2R41
contig00173	80.283	105.749	207.807	219.359	700	0.294196663	isogroup00001	ENSG00000155657	ENST00000448510	TTN
contig00177	1.649	3.291	3.424	3.097	1041	0.859613361	isogroup00001	ENSG00000122176	ENST00000354955	FMOD
contig00180	2.578	2.115	2.657	2.559	945	0.243631172	isogroup00001	ENSG00000171853	ENST00000382110	TRAPPC12
contig00181	2.785	2.36	2.751	3.122	839	0.03231382	isogroup00001	ENSG00000171853	ENST00000382110	TRAPPC12
contig00183	298.671	552.781	706.399	773.455	604	0.495359585	isogroup00001	ENSG00000133020	ENST00000403437	MYH8
contig00192	1959.233	2920.834	2046.186	2905.451	700	0.643655595	isogroup00001	ENSG00000198763	ENST00000361453	MT-ND2
contig00197	2145.051	1170.062	2077.601	1495.079	646	0.766213271	isogroup00001	ENSG00000163093	ENST00000554017	BBS5
contig00221	6868.419	7256.105	8954.885	7914.851	1252	0.123976103	isogroup00001	ENSG00000198786	ENST00000361567	MT-ND5
contig00222	5192.008	4983.736	5864.881	5577.423	1063	0.155096941	isogroup00001	ENSG00000198786	ENST00000361567	MT-ND5
contig00226	254.52	263.664	615.866	502.028	642	0.041716766	isogroup00001	ENSG00000155657	ENST00000342992	TTN
contig00242	4899.183	2844.824	5089.576	3449.011	703	0.667233411	isogroup00001	ENSG00000125414	ENST00000397183	MYH2
contig00253	4682.087	4824.258	5671.165	5395.074	565	0.09194409	isogroup00001	ENSG00000198888	ENST00000361390	MT-ND1
contig00254	1178.773	2923.615	3692.688	4517.056	1132	0.703464342	isogroup00001	ENSG00000109061	ENST00000379814	MYH1
contig00268	47.186	74.593	106.514	109.263	838	0.389747877	isogroup00001	ENSG00000155657	ENST00000414766	TTN
contig00301	1556.937	3726.1	5198.496	5901.003	1013	0.62687871	isogroup00001	ENSG00000109061	ENST00000226207	MYH1
contig00304	3841.129	2755.109	3863.239	3493.453	2278	0.340450515	isogroup00001	ENSG00000125414	ENST00000397183	MYH2
contig00332	2053.631	4823.054	7625.09	7800.764	1036	0.539894416	isogroup00001	ENSG00000109061	ENST00000226207	MYH1
contig00340	699.666	794.446	154.919	597.839	2600	0.752536259	isogroup00001	ENSG00000155657	ENST00000460472	TTN
contig02797	19.939	30.563	8.78	12.142	6322	0.628766814	isogroup00047	ENSG00000114796	ENST00000454652	KLHL24
isotig00435	10.498	13.644	11.859	11.76	1387	0.308399714	isogroup00047	ENSG00000127922	ENST00000248566	SHFM1
isotig00436	13.864	18.829	15.613	15.707	1280	0.367804539	isogroup00047	ENSG00000127922	ENST00000248566	SHFM1
isotig00437	36.76	50.158	41.517	41.357	487	0.371965883	isogroup00047	ENSG00000127922	ENST00000248566	SHFM1
isotig00440	259.149	594.954	472.848	896.226	894	0.926392124	isogroup00048	ENSG00000086967	ENST00000357701	MYBPC2
isotig00445	144.956	335.73	274.848	485.629	429	0.915523784	isogroup00048	ENSG00000086967	ENST00000357701	MYBPC2
isotig00447	605.118	489.275	268.323	301.327	600	0.265330277	isogroup00049	ENSG00000100097	ENST00000215909	LGALS1
contig02880	3.429	3.005	1.85	2.965	868	0.158844969	isogroup00050	ENSG00000181555	ENST00000409792	SETD2
contig02883	5.02	2.497	4.266	4.841	541	0.375535432	isogroup00050	ENSG00000110925	ENST00000228515	CSRNP2
contig02892	165.434	206.81	250.545	204.813	712	0.041444353	isogroup00051	ENSG00000122566	ENST00000360787	HNRNPA2B1
contig02932	28.313	23.236	22.084	17.872	1051	0.562335613	isogroup00052	ENSG00000173141	ENST00000309594	MRP63
isotig00480	1.551	1.659	1.092	1.441	1996	0.136817089	isogroup00052	ENSG00000101654	ENST00000543302	RNMT
contig02958	59.425	18.196	19.123	13.456	3263	0.953361013	isogroup00053	ENSG00000196091	ENST00000452455	MYBPC1
isotig00492	154.646	90.751	110.476	112.55	7130	0.565303975	isogroup00053	ENSG00000196091	ENST00000550270	MYBPC1
isotig00493	287.632	251.79	288.487	295.475	5673	0.123271586	isogroup00053	ENSG00000196091	ENST00000392934	MYBPC1
isotig00495	292.763	255.08	297.442	306.155	5604	0.123215225	isogroup00053	ENSG00000196091	ENST00000553190	MYBPC1
isotig00497	352.251	300.396	350.053	359.036	4470	0.154542722	isogroup00053	ENSG00000196091	ENST00000392934	MYBPC1
isotig00501	359.797	305.347	362.421	373.632	4401	0.138395206	isogroup00053	ENSG00000196091	ENST00000553190	MYBPC1
isotig00504	207.277	181.431	194.858	218.046	2692	0.024404449	isogroup00053	ENSG00000196091	ENST00000553190	MYBPC1
isotig00505	130.807	64.929	190.15	78.733	4471	0.926918163	isogroup00054	ENSG00000165795	ENST00000556147	NDRG2
isotig00506	119.476	59.242	171.734	71.218	4429	0.926119711	isogroup00054	ENSG00000165795	ENST00000403829	NDRG2
isotig00507	94.228	47.729	132.771	55.185	4098	0.917937927	isogroup00054	ENSG00000165795	ENST00000553593	NDRG2
isotig00508	67.651	35.831	98.307	39.621	2995	0.907708349	isogroup00054	ENSG00000165795	ENST00000553503	NDRG2
isotig00509	9.269	8.759	12.223	8.239	2461	0.429905313	isogroup00054	ENSG00000189403	ENST00000405805	HMGB1
isotig00510	11.356	10.626	15.087	10.23	2342	0.440961524	isogroup00054	ENSG00000189403	ENST00000405805	HMGB1
isotig00511	14.414	13.388	19.4	12.748	1329	0.49127339	isogroup00054	ENSG00000189403	ENST00000405805	HMGB1
isotig00512	18.959	17.458	25.65	17.045	1210	0.499436387	isogroup00054	ENSG00000189403	ENST00000405805	HMGB1
isotig00513	14.34	14.174	15.614	14.345	2238	0.099308635	isogroup00055	ENSG00000004961	ENST00000380762	HCCS
isotig00514	538.749	306.021	776.281	771.282	1016	0.658102878	isogroup00055	ENSG00000166710	ENST00000561424	B2M
isotig00516	21.903	18.56	21.993	19.651	1023	0.307450966	isogroup00055	ENSG00000004961	ENST00000380762	HCCS

isotig00517	5.139	5.11	4.596	4.585	5853	0.131340648	isogroup00056	ENSG00000120729	ENST00000239926	MYOT
isotig00518	5.6	6.003	5.105	5.337	4978	0.029937251	isogroup00056	ENSG00000120729	ENST00000239926	MYOT
isotig00519	5.693	6.101	5.191	5.423	4894	0.0314684	isogroup00056	ENSG00000120729	ENST00000239926	MYOT
isotig00521	6.024	6.085	5.437	5.57	4862	0.109547607	isogroup00056	ENSG00000120729	ENST00000239926	MYOT
isotig00522	6.126	6.187	5.531	5.662	4778	0.110552717	isogroup00056	ENSG00000120729	ENST00000239926	MYOT
isotig00523	5.476	6.042	5.158	5.453	4792	0.017143233	isogroup00056	ENSG00000120729	ENST00000239926	MYOT
isotig00525	5.571	6.145	5.249	5.544	4708	0.017143233	isogroup00056	ENSG00000120729	ENST00000239926	MYOT
isotig00527	6.702	6.782	5.577	5.906	4131	0.098782596	isogroup00056	ENSG00000120729	ENST00000239926	MYOT
isotig00528	7.828	8.595	6.619	7.411	3256	0.048282859	isogroup00056	ENSG00000120729	ENST00000239926	MYOT
isotig00529	8.566	8.818	7.19	7.847	3140	0.063209213	isogroup00056	ENSG00000120729	ENST00000239926	MYOT
isotig00530	7.77	8.813	6.794	7.717	3070	0.09109867	isogroup00056	ENSG00000120729	ENST00000239926	MYOT
isotig00531	3.691	5.067	3.241	3.422	3441	0.404655445	isogroup00056	ENSG00000133961	ENST00000555238	NUMB
isotig00532	122.521	181.198	155.135	189.574	2025	0.546253851	isogroup00056	ENSG00000120729	ENST00000515645	MYOT
isotig00533	127.815	189.027	161.845	197.764	1941	0.546422935	isogroup00056	ENSG00000120729	ENST00000515645	MYOT
isotig00535	36.024	51.97	41.809	55.269	1855	0.53646577	isogroup00056	ENSG00000120729	ENST00000515645	MYOT
isotig00536	37.28	53.745	43.406	57.163	1781	0.536822725	isogroup00056	ENSG00000120729	ENST00000515645	MYOT
isotig00537	37.723	54.422	43.788	57.875	1771	0.537085744	isogroup00056	ENSG00000120729	ENST00000515645	MYOT
isotig00539	39.115	56.392	45.55	59.977	1697	0.537461486	isogroup00056	ENSG00000120729	ENST00000515645	MYOT
isotig00541	62.635	90.469	75.337	97.173	1455	0.542017359	isogroup00056	ENSG00000120729	ENST00000515645	MYOT
isotig00542	66.461	95.996	79.947	103.108	1371	0.54246825	isogroup00056	ENSG00000120729	ENST00000515645	MYOT
isotig00571	3.987	2.853	3.707	4.027	7008	0.131678816	isogroup00058	ENSG00000126777	ENST00000459737	KTN1
isotig00575	4.052	2.904	3.768	4.096	6924	0.131678816	isogroup00058	ENSG00000126777	ENST00000459737	KTN1
isotig00576	3.937	2.807	3.665	3.986	6921	0.131678816	isogroup00058	ENSG00000126777	ENST00000459737	KTN1
isotig00580	4.002	2.857	3.727	4.055	6837	0.131678816	isogroup00058	ENSG00000126777	ENST00000438792	KTN1
isotig00582	3.923	2.867	3.628	4.057	6652	0.09609604	isogroup00058	ENSG00000126777	ENST00000459737	KTN1
isotig00583	3.991	2.921	3.692	4.13	6568	0.09609604	isogroup00058	ENSG00000126777	ENST00000459737	KTN1
isotig00584	3.87	2.818	3.584	4.013	6565	0.09609604	isogroup00058	ENSG00000126777	ENST00000459737	KTN1
isotig00585	3.938	2.872	3.648	4.087	6481	0.09609604	isogroup00058	ENSG00000126777	ENST00000438792	KTN1
isotig00586	7.653	7.964	9.219	13.88	5938	0.491752461	isogroup00058	ENSG00000107263	ENST00000372190	RAPGEF1
isotig00587	7.676	7.803	9.317	13.89	5824	0.470823627	isogroup00058	ENSG00000107263	ENST00000337036	RAPGEF1
isotig00588	5.342	5.94	7.062	10.125	3501	0.440858195	isogroup00058	ENSG00000107263	ENST00000337036	RAPGEF1
isotig00589	467.227	310.952	307.885	228.365	750	0.679238371	isogroup00059	ENSG00000241343	ENST00000427805	RPL36A
isotig00591	595.394	390.938	390.626	289.523	436	0.694268054	isogroup00059	ENSG00000241343	ENST00000427805	RPL36A
isotig00592	111.991	102.954	65.371	53.621	5706	0.262042534	isogroup00060	ENSG00000174437	ENST00000308664	ATP2A2
isotig00593	118.934	109.239	69.283	56.865	5357	0.262267979	isogroup00060	ENSG00000174437	ENST00000308664	ATP2A2
isotig00594	30.329	23.297	17.261	12.211	4316	0.550988202	isogroup00060	ENSG00000103365	ENST00000309859	GGA2
isotig00595	141.636	111.676	67.935	56.945	5166	0.466840761	isogroup00060	ENSG00000174437	ENST00000539276	ATP2A2
isotig00596	32.52	24.776	18.312	12.948	3967	0.562561058	isogroup00060	ENSG00000103365	ENST00000309859	GGA2
isotig00597	160.732	147.249	92.869	76.065	3879	0.263254302	isogroup00060	ENSG00000174437	ENST00000308664	ATP2A2
isotig00598	166.013	151.97	95.923	78.276	3806	0.278020966	isogroup00060	ENSG00000174437	ENST00000308664	ATP2A2
isotig00599	9.076	11.22	9.104	8.091	3088	0.220269407	isogroup00060	ENSG00000009954	ENST00000404251	BAZ1B
isotig00600	46.346	33.858	24.802	16.793	2489	0.629039227	isogroup00060	ENSG00000103365	ENST00000309859	GGA2
isotig00601	8.977	11.358	9.156	8.024	2913	0.233194935	isogroup00060	ENSG00000168827	ENST00000312756	GFM1
isotig00602	51.209	37.868	27.557	18.485	2416	0.615221312	isogroup00060	ENSG00000103365	ENST00000309859	GGA2
isotig00603	650.599	579.006	413.12	268.482	2676	0.516767491	isogroup00061	ENSG00000143549	ENST00000368530	TPM3
isotig00604	53.483	35.073	30.466	18.617	2436	0.727013978	isogroup00061	ENSG00000143549	ENST00000368533	TPM3
isotig00605	746.286	641.401	461.568	292.539	1702	0.570010145	isogroup00061	ENSG00000143549	ENST00000368530	TPM3
isotig00606	66.047	45.918	38.544	23.836	1857	0.693309912	isogroup00061	ENSG00000143549	ENST00000368533	TPM3
isotig00607	2573.651	2181.435	2594.269	3504.7	1262	0.250638762	isogroup00061	ENSG00000140416	ENST00000403994	TPM1
isotig00608	16.624	14.77	33.268	11.156	1790	0.737271737	isogroup00061	ENSG00000140416	ENST00000559397	TPM1
isotig00609	3076.765	2603.901	3106.97	4188.475	1042	0.250657549	isogroup00061	ENSG00000140416	ENST00000403994	TPM1
isotig00610	11.691	4.085	5.662	3.662	755	0.858937026	isogroup00061	ENSG00000143549	ENST00000368533	TPM3
isotig00611	18.687	25.049	19.867	18.916	1874	0.321437965	isogroup00062	ENSG00000230989	ENST00000570259	HSBP1
isotig00612	36.224	46.46	37.715	33.325	572	0.196823101	isogroup00062	ENSG00000230989	ENST00000570259	HSBP1
contig03187	155.01	221.627	260.628	159.052	592	0.058099121	isogroup00063	ENSG00000026025	ENST00000545533	VIM
isotig00613	7.936	7.419	9.549	6.135	3796	0.376653265	isogroup00063	ENSG00000077380	ENST00000409197	DYNC1I2
isotig00614	7.599	5.744	6.936	5.204	3082	0.472166905	isogroup00063	ENSG00000077380	ENST00000409197	DYNC1I2
isotig00616	9.795	9.124	11.557	7.584	3000	0.375037574	isogroup00063	ENSG00000077380	ENST00000409197	DYNC1I2
isotig00617	5.859	6.935	9.624	5.136	2057	0.275099572	isogroup00063	ENSG00000077380	ENST00000534253	DYNC1I2
isotig00618	3.981	2.835	3.666	2.47	1343	0.472749305	isogroup00063	ENSG00000077380	ENST00000534253	DYNC1I2
isotig00620	8.971	10.687	14.448	7.952	1261	0.251127226	isogroup00063	ENSG00000077380	ENST00000534253	DYNC1I2
isotig00621	8.54	9.876	8.444	6.538	878	0.048282859	isogroup00064	ENSG00000243147	ENST00000296102	MRPL33
isotig00624	11.564	13.641	11.307	8.987	629	0.008040881	isogroup00064	ENSG00000243147	ENST00000296102	MRPL33
isotig00629	13.283	15.701	12.96	10.286	545	0.008040881	isogroup00064	ENSG00000243147	ENST00000296102	MRPL33
isotig00635	9.155	9.133	8.948	6.938	497	0.189965807	isogroup00064	ENSG00000243147	ENST00000379666	MRPL33
isotig00638	10.932	10.932	10.649	8.235	413	0.194455925	isogroup00064	ENSG00000243147	ENST00000379666	MRPL33
isotig00639	129.899	197.888	133.927	131.864	565	0.535413692	isogroup00065	ENSG00000173436	ENST00000322753	MINOS1
isotig00642	166.391	102.289	105.398	88.633	596	0.66046066	isogroup00066	ENSG00000071082	ENST00000409733	RPL31
isotig00643	196.032	120.601	124.343	104.323	504	0.661371834	isogroup00066	ENSG00000071082	ENST00000409733	RPL31
isotig00644	203.082	124.792	128.763	106.754	451	0.66905576	isogroup00066	ENSG00000071082	ENST00000409733	RPL31
isotig00660	6.517	10.366	6.193	6.235	3861	0.235439994	isogroup00068	ENSG00000164122	ENST00000512254	ASB5
isotig01192	142.886	56.501	113.818	72.632	782	0.909587059	isogroup00100	ENSG00000124701	ENST00000426505	APOBEC2
isotig01193	176.971	70.979	140.893	85.384	682	0.911090028	isogroup00100	ENSG00000124701	ENST00000244669	APOBEC2
isotig01194	156.494	61.895	124.658	79.549	714	0.910272789	isogroup00100	ENSG00000124701	ENST00000426505	APOBEC2
isotig01195	196.57	78.855	156.497	94.84	614	0.911822725	isogroup00100	ENSG00000124701	ENST00000244669	APOBEC2
isotig01196	16.977	11.977	18.086	13.895	4650	0.567614789	isogroup00101	ENSG00000128989	ENST00000561971	ARPP19
isotig01199	17.202	12.059	18.278	14.064	4591	0.568394454	isogroup00101	ENSG00000128989	ENST00000569281	ARPP19
isotig01200	234.796	200.088	201.344	157.945	543	0.420079657	isogroup00102	ENSG00000112695	ENST00000370081	COX7A2
isotig01248	1.551	1.513	1.871	1.603	5749	0.128945292	isogroup00105	ENSG00000165525	ENST00000545773	NEMF
isotig01249	1.093	1.06	1.264	1.02	5710	0.087519726	isogroup00105	ENSG00000165525	ENST00000545773	NEMF
isotig01250	1.602	1.582	1.928	1.675	5476	0.121392876	isogroup00105	ENSG00000165525	ENST00000545773	NEMF
isotig01251	1.209	1.145	1.403	1.15	5447	0.100069512	isogroup00105	ENSG00000165525	ENST00000545773	NEMF

isotig01252	1.121	1.107	1.29	1.064	5437	0.081160292	isogroup00105	ENSG00000165525	ENST00000545773	NEMF
isotig01253	1.245	1.198	1.438	1.202	5174	0.087209739	isogroup00105	ENSG00000165525	ENST00000545773	NEMF
isotig01258	1.923	1.805	2.434	2.048	4199	0.192051176	isogroup00105	ENSG00000165516	ENST00000298307	KLHDC2
isotig01259	1.298	1.186	1.605	1.253	4160	0.148934771	isogroup00105	ENSG00000165516	ENST00000298307	KLHDC2
isotig01260	2.026	1.901	2.567	2.16	3978	0.199706921	isogroup00105	ENSG00000165516	ENST00000298307	KLHDC2
isotig01261	1.367	1.248	1.693	1.321	3939	0.154899677	isogroup00105	ENSG00000165516	ENST00000298307	KLHDC2
isotig01262	2.019	1.922	2.552	2.18	3926	0.183258811	isogroup00105	ENSG00000165516	ENST00000298307	KLHDC2
isotig01263	1.474	1.313	1.823	1.45	3897	0.159737356	isogroup00105	ENSG00000165516	ENST00000298307	KLHDC2
isotig01264	1.351	1.26	1.666	1.33	3887	0.136817089	isogroup00105	ENSG00000165516	ENST00000298307	KLHDC2
isotig01265	2.135	2.032	2.701	2.307	3705	0.190473059	isogroup00105	ENSG00000165516	ENST00000298307	KLHDC2
isotig01266	1.558	1.387	1.931	1.534	3676	0.170136019	isogroup00105	ENSG00000165516	ENST00000298307	KLHDC2
isotig01267	1.429	1.331	1.764	1.408	3666	0.142425039	isogroup00105	ENSG00000165516	ENST00000298307	KLHDC2
isotig01268	1.545	1.401	1.905	1.547	3624	0.152936424	isogroup00105	ENSG00000165516	ENST00000298307	KLHDC2
isotig01269	1.64	1.488	2.026	1.645	3403	0.162705719	isogroup00105	ENSG00000165516	ENST00000298307	KLHDC2
isotig01307	1.392	1.396	1.352	1.077	3003	0.080728188	isogroup00106	ENSG00000047410	ENST00000367478	TPR
isotig01308	1.438	1.435	1.389	1.107	2889	0.080728188	isogroup00106	ENSG00000047410	ENST00000367478	TPR
isotig01315	1.471	1.305	1.353	1.1	3065	0.080728188	isogroup00107	ENSG00000130254	ENST00000252542	SAFB2
isotig01316	1.535	1.281	1.45	1.083	2999	0.160827008	isogroup00107	ENSG00000160633	ENST00000292123	SAFB
isotig01317	1.307	1.339	1.28	1.131	3013	0.022976629	isogroup00107	ENSG00000130254	ENST00000252542	SAFB2
isotig01318	1.369	1.316	1.378	1.115	2947	0.068140828	isogroup00107	ENSG00000160633	ENST00000538656	SAFB
isotig01339	2.264	1.874	1.578	1.253	1036	0.155397535	isogroup00107	ENSG00000160633	ENST00000292123	SAFB
isotig01344	16.8	12.311	13.724	8.854	3660	0.594593071	isogroup00108	ENSG00000135486	ENST00000552591	HNRNPA1
isotig01347	17.451	12.874	14.329	9.29	3439	0.588487262	isogroup00108	ENSG00000135486	ENST00000552591	HNRNPA1
isotig01348	12.041	8.916	10.261	6.139	3116	0.606795296	isogroup00108	ENSG00000135486	ENST00000547276	HNRNPA1
isotig01349	12.451	9.325	10.715	6.451	2895	0.594912452	isogroup00108	ENSG00000135486	ENST00000547276	HNRNPA1
isotig01352	25.947	19.062	20.237	14.235	1285	0.569108364	isogroup00108	ENSG00000135486	ENST00000552591	HNRNPA1
isotig01355	28.336	20.561	21.694	15.396	1171	0.579986098	isogroup00108	ENSG00000135486	ENST00000552591	HNRNPA1
isotig01358	30.475	22.29	23.373	16.671	1077	0.573504546	isogroup00108	ENSG00000135486	ENST00000552591	HNRNPA1
isotig01359	12.649	9.737	10.457	6.771	741	0.564608852	isogroup00108	ENSG00000135486	ENST00000547276	HNRNPA1
isotig01360	14.694	10.842	11.4	7.581	627	0.601713384	isogroup00108	ENSG00000135486	ENST00000547276	HNRNPA1
isotig01361	16.61	12.621	12.976	8.78	533	0.576698354	isogroup00108	ENSG00000135486	ENST00000547276	HNRNPA1
contig04160	38.004	37.23	49.703	33.03	1567	0.447621553	isogroup00109	ENSG00000164039	ENST00000296424	BDH2
contig04161	3.365	3.61	3.721	2.862	1181	0.172428045	isogroup00109	ENSG00000221837	ENST00000397911	KRTAP10-9
isotig01366	3.29	3.198	3.44	2.763	1542	0.247510709	isogroup00109	ENSG00000164039	ENST00000504285	BDH2
isotig01368	3.467	3.367	3.615	2.94	1449	0.243912978	isogroup00109	ENSG00000164039	ENST00000504285	BDH2
isotig01370	6.917	7.5	7.37	5.782	1393	0.234463065	isogroup00109	ENSG00000164039	ENST00000504285	BDH2
isotig01372	7.125	6.909	7.171	5.752	1344	0.353206959	isogroup00109	ENSG00000164039	ENST00000504285	BDH2
isotig01374	7.374	7.995	7.846	6.195	1300	0.225464041	isogroup00109	ENSG00000164039	ENST00000504285	BDH2
isotig01376	7.615	7.38	7.652	6.179	1251	0.346603292	isogroup00109	ENSG00000164039	ENST00000504285	BDH2
isotig01380	2.228	2.571	4.6	4.614	3520	0.051664537	isogroup00110	ENSG00000115592	ENST00000529249	PRKAG3
isotig01381	2.465	2.796	5.037	5.007	3341	0.0314684	isogroup00110	ENSG00000115592	ENST00000529249	PRKAG3
isotig01382	2.395	2.761	4.932	4.937	3335	0.051664537	isogroup00110	ENSG00000115592	ENST00000529249	PRKAG3
isotig01385	2.655	3.01	5.413	5.371	3156	0.029937251	isogroup00110	ENSG00000115592	ENST00000529249	PRKAG3
isotig01392	1.176	1.254	2.46	2.439	2317	0.034718569	isogroup00110	ENSG00000115592	ENST00000529249	PRKAG3
isotig01394	1.457	1.495	2.964	2.871	2138	0.074039979	isogroup00110	ENSG00000115592	ENST00000529249	PRKAG3
isotig01395	1.345	1.438	2.793	2.754	2132	0.05275419	isogroup00110	ENSG00000115592	ENST00000529249	PRKAG3
isotig01396	1.005	1.346	2.104	2.188	2045	0.122444954	isogroup00110	ENSG00000115592	ENST00000529249	PRKAG3
isotig01397	1.669	1.718	3.375	3.256	1953	0.091530773	isogroup00110	ENSG00000115592	ENST00000529249	PRKAG3
isotig01399	1.15	1.302	2.322	2.2	1388	0.034718569	isogroup00110	ENSG00000115592	ENST00000529249	PRKAG3
isotig01412	5.338	3.854	5.172	5.235	7047	0.274836552	isogroup00111	ENSG00000110768	ENST00000453096	GTF2H1
isotig01413	2.709	2.1	2.4	1.78	2547	0.364413467	isogroup00111	ENSG00000110768	ENST00000453096	GTF2H1
isotig01414	1154.414	639.957	787.174	610.095	507	0.735439994	isogroup00112	ENSG00000112306	ENST00000230050	RPS12
isotig01415	2.245	2.042	2.618	2.257	1601	0.123215225	isogroup00113	ENSG00000134809	ENST00000525158	TIMM10
isotig01416	2.497	2.395	2.928	2.529	1462	0.102323965	isogroup00113	ENSG00000134809	ENST00000525158	TIMM10
isotig01417	2.044	1.927	2.334	2.05	1536	0.091699857	isogroup00113	ENSG00000134809	ENST00000525158	TIMM10
isotig01418	2.288	2.286	2.631	2.314	1397	0.071109191	isogroup00113	ENSG00000134809	ENST00000525158	TIMM10
isotig01419	2.765	2.592	3.239	2.797	1116	0.138291876	isogroup00113	ENSG00000134809	ENST00000525158	TIMM10
isotig01420	1.33	1.179	1.488	1.248	984	0.099740738	isogroup00113	ENSG00000134809	ENST00000525158	TIMM10
isotig01421	1.617	1.649	1.839	1.553	845	0.060513264	isogroup00113	ENSG00000134809	ENST00000525158	TIMM10
isotig01423	1.168	1.39	1.214	1.086	780	0.02329601	isogroup00113	ENSG00000134809	ENST00000525158	TIMM10
isotig01424	1.605	1.571	1.78	1.475	499	0.123468851	isogroup00113	ENSG00000134809	ENST00000525158	TIMM10
isotig01425	185.077	198.969	167.153	149.675	1399	0.041378598	isogroup00114	ENSG00000165629	ENST00000356708	ATP5C1
isotig01426	190.726	204.834	172.697	153.952	1345	0.041472533	isogroup00114	ENSG00000165629	ENST00000356708	ATP5C1
isotig01427	73.212	63.076	68.308	57.815	642	0.333790486	isogroup00115	ENSG00000123349	ENST00000551018	PFDN5
isotig01429	68.486	58.112	62.227	52.871	481	0.361689336	isogroup00115	ENSG00000123349	ENST00000551018	PFDN5
isotig01432	48.261	44.269	41.389	37.045	617	0.272910874	isogroup00116	ENSG00000214265	ENST00000551312	SNURF
isotig01437	62.123	56.983	53.273	47.718	479	0.27475201	isogroup00116	ENSG00000214265	ENST00000551312	SNURF
isotig01440	11.655	9.563	7.314	4.322	5358	0.574340573	isogroup00117	ENSG00000175155	ENST00000312655	YPEL2
isotig01441	176.604	130.544	134.169	115.12	1146	0.502536259	isogroup00117	ENSG00000122406	ENST00000370321	RPL5
isotig01443	189.483	140.024	143.91	123.507	1068	0.502883821	isogroup00117	ENSG00000122406	ENST00000370321	RPL5
isotig01444	131.562	92.035	100.471	85.425	1011	0.543849102	isogroup00117	ENSG00000122406	ENST00000370321	RPL5
isotig01446	142.539	99.668	108.805	92.542	933	0.544375141	isogroup00117	ENSG00000122406	ENST00000370321	RPL5
isotig01447	75.949	53.508	58.409	48.163	792	0.518054407	isogroup00117	ENSG00000122406	ENST00000432788	RPL5
contig04329	40.168	36.101	39.047	34.476	1711	0.301279402	isogroup00118	ENSG00000113141	ENST00000417647	IK
isotig01448	119.298	111.567	107.778	94.635	594	0.262803412	isogroup00118	ENSG00000131495	ENST00000252102	NDUFA2
isotig01452	80.131	74.472	71.597	61.327	566	0.276114075	isogroup00118	ENSG00000131495	ENST00000252102	NDUFA2
isotig01453	15.254	28.568	18.696	16.444	599	0.530284813	isogroup00119	ENSG00000109390	ENST00000544855	NDUFC1
isotig01459	102.796	72.789	96.738	88.056	2832	0.429679868	isogroup00120	ENSG00000058056	ENST00000263966	USP13
isotig01460	3.881	2.967	4.26	3.164	1205	0.414180507	isogroup00120	ENSG00000058056	ENST00000496897	USP13
isotig01461	161.6	285.993	62.008	71.722	1208	0.351084016	isogroup00121	ENSG00000172399	ENST00000307128	MYOZ2
isotig01468	152.745	91.491	145.498	148.179	5554	0.418680018	isogroup00122	ENSG00000164309	ENST00000446378	CMYA5
isotig01469	231.611	139.339	220.905	226.201	3610	0.420126625	isogroup00122	ENSG00000164309	ENST00000446378	CMYA5

isotig01470	236.157	142.079	225.242	230.632	3540	0.42019238	isogroup00122	ENSG00000164309	ENST00000446378	CMYA5
isotig01471	6.924	5.72	8.861	8.898	3369	0.276499211	isogroup00122	ENSG00000129744	ENST00000250693	ART1
isotig01472	6.551	5.379	8.365	8.384	3335	0.296685955	isogroup00122	ENSG00000129744	ENST00000250693	ART1
isotig01473	4.671	4.007	6.042	6.144	2589	0.169685128	isogroup00122	ENSG00000129749	ENST00000250699	CHRNA10
isotig01474	4.155	3.539	5.357	5.437	2555	0.188096491	isogroup00122	ENSG00000129749	ENST00000250699	CHRNA10
isotig01475	104.853	101.859	96.553	82.981	991	0.247163147	isogroup00123	ENSG00000178127	ENST00000318388	NDUFV2
isotig01476	7.911	5.34	6.162	4.241	833	0.624783948	isogroup00123	ENSG00000178127	ENST00000400033	NDUFV2
isotig01477	45.157	44.984	42.426	34.681	383	0.287827835	isogroup00123	ENSG00000178127	ENST00000400033	NDUFV2
isotig01764	5.461	7.554	6.459	7.165	3538	0.33980236	isogroup00131	ENSG00000163827	ENST00000395905	LRRC2
isotig01765	4.88	6.742	5.854	6.411	3509	0.322762456	isogroup00131	ENSG00000163827	ENST00000395905	LRRC2
isotig01766	4.827	6.75	5.841	6.368	3456	0.333039002	isogroup00131	ENSG00000163827	ENST00000395905	LRRC2
isotig01767	5.012	6.942	6.031	6.609	3393	0.325721425	isogroup00131	ENSG00000163827	ENST00000395905	LRRC2
isotig01768	5.332	7.327	6.329	6.996	3392	0.327524987	isogroup00131	ENSG00000163827	ENST00000395905	LRRC2
isotig01769	4.667	6.482	5.681	6.159	3310	0.319850455	isogroup00131	ENSG00000163827	ENST00000395905	LRRC2
isotig01770	4.857	6.677	5.877	6.407	3247	0.312467123	isogroup00131	ENSG00000163827	ENST00000395905	LRRC2
isotig01771	4.889	6.774	6.035	6.438	2776	0.311076877	isogroup00131	ENSG00000163827	ENST00000395905	LRRC2
isotig01772	3.521	4.939	3.765	4.564	1485	0.362450214	isogroup00131	ENSG00000163827	ENST00000395905	LRRC2
isotig01773	4.524	6.236	4.765	5.856	1368	0.36854663	isogroup00131	ENSG00000163827	ENST00000395905	LRRC2
isotig01774	2.983	4.079	3.143	3.851	1339	0.322630946	isogroup00131	ENSG00000163827	ENST00000395905	LRRC2
isotig01775	2.761	3.991	2.996	3.629	1286	0.361764485	isogroup00131	ENSG00000163827	ENST00000395905	LRRC2
isotig01776	3.168	4.381	3.378	4.158	1223	0.358730367	isogroup00131	ENSG00000163827	ENST00000395905	LRRC2
isotig01777	4.055	5.449	4.202	5.23	1222	0.337491546	isogroup00131	ENSG00000163827	ENST00000395905	LRRC2
isotig01778	2.032	2.859	2.167	2.673	1140	0.296347787	isogroup00131	ENSG00000163827	ENST00000395905	LRRC2
isotig01779	2.451	3.237	2.552	3.217	1077	0.297888329	isogroup00131	ENSG00000163827	ENST00000395905	LRRC2
contig04574	1.507	2.488	1.717	1.992	742	0.425199143	isogroup00132	ENSG00000196712	ENST00000456735	NF1
contig04575	17.346	27.821	24.751	31.193	1172	0.536005486	isogroup00132	ENSG00000179094	ENST00000354903	PER1
contig04580	10.1	16.128	19.01	18.857	609	0.314853085	isogroup00132	ENSG00000179094	ENST00000317276	PER1
contig04582	22.966	36.743	39.785	36.765	847	0.356090779	isogroup00132	ENSG00000179094	ENST00000317276	PER1
contig04584	14.412	20.964	21.107	25.813	503	0.373356128	isogroup00132	ENSG00000179094	ENST00000354903	PER1
contig04590	3.995	2.592	3.594	3.146	925	0.490202525	isogroup00132	ENSG00000164692	ENST00000545487	COL1A2
contig04594	36.287	34.341	41.464	30.346	1246	0.242344255	isogroup00133	ENSG00000128342	ENST00000403987	LIF
contig04600	104.431	79.095	101.428	90.325	1152	0.511093785	isogroup00133	ENSG00000108654	ENST00000540698	DDX5
contig04602	113.339	91.236	141.356	102.636	508	0.611106936	isogroup00133	ENSG00000108654	ENST00000540698	DDX5
isotig01781	3.46	3.338	3.868	4.91	3080	0.20295709	isogroup00134	ENSG00000085377	ENST00000369110	PREP
isotig01782	6.34	6.173	6.553	8.622	2666	0.274075674	isogroup00134	ENSG00000085377	ENST00000369110	PREP
isotig01784	2.3	2.288	2.834	3.413	2387	0.152945818	isogroup00134	ENSG00000085377	ENST00000369110	PREP
isotig01785	5.154	4.738	5.123	6.983	2219	0.237487788	isogroup00134	ENSG00000085377	ENST00000369110	PREP
isotig01787	5.947	5.899	6.245	8.115	1973	0.271924551	isogroup00134	ENSG00000085377	ENST00000369110	PREP
isotig01789	4.977	5.093	5.871	7.05	1634	0.214342076	isogroup00134	ENSG00000085377	ENST00000369110	PREP
isotig01791	4.108	3.731	4.077	5.582	1526	0.211833997	isogroup00134	ENSG00000085377	ENST00000369110	PREP
isotig01792	2.247	2.003	2.944	3.393	1187	0.006660029	isogroup00134	ENSG00000085377	ENST00000369110	PREP
isotig01796	2.842	6.933	4.477	4.668	7728	0.596265124	isogroup00135	ENSG00000198796	ENST00000361673	ALPK2
isotig01797	2.955	7.252	4.639	4.865	7267	0.60728376	isogroup00135	ENSG00000198796	ENST00000361673	ALPK2
isotig01810	851.883	717.45	639.37	500.277	573	0.460490719	isogroup00136	ENSG00000138326	ENST00000440692	RPS24
isotig01812	754.917	565.098	451.664	481.141	703	0.339727211	isogroup00137	ENSG00000163682	ENST00000449470	RPL9
isotig01813	127.629	107.643	145.63	117.495	1556	0.431323739	isogroup00138	ENSG00000090565	ENST00000457159	RAB11FIP3
isotig01814	138.68	116.952	158.066	127.586	1431	0.418698805	isogroup00138	ENSG00000090565	ENST00000457159	RAB11FIP3
isotig01815	7.166	5.723	6.765	5.597	928	0.317755692	isogroup00138	ENSG00000139233	ENST00000446587	LLPH
isotig01822	26.961	17.66	26.12	17.409	2982	0.679191403	isogroup00140	ENSG00000165119	ENST00000376268	HNRNPK
isotig01826	27.021	17.601	26.278	17.366	2905	0.68608627	isogroup00140	ENSG00000165119	ENST00000376268	HNRNPK
isotig01830	24.13	16.743	21.732	16.561	1564	0.594095213	isogroup00140	ENSG00000165119	ENST00000376268	HNRNPK
isotig01831	24.101	16.58	21.814	16.432	1487	0.602727888	isogroup00140	ENSG00000165119	ENST00000376268	HNRNPK
isotig01832	5.391	6.609	6.63	6.569	4216	0.245171714	isogroup00141	ENSG00000151414	ENST00000538004	NEK7
isotig01833	5.374	6.485	6.664	6.508	4075	0.220053355	isogroup00141	ENSG00000151414	ENST00000538004	NEK7
isotig01834	3.543	4.451	3.471	4.284	2370	0.347777486	isogroup00141	ENSG00000151414	ENST00000538004	NEK7
isotig01835	531.643	488.518	779.056	892.021	1371	0.058343353	isogroup00141	ENSG00000060138	ENST00000279550	CSDA
isotig01836	3.396	4.088	3.334	4.027	2229	0.306145262	isogroup00141	ENSG00000151414	ENST00000538004	NEK7
isotig01837	551.766	507.008	808.539	925.779	1321	0.058343353	isogroup00141	ENSG00000060138	ENST00000279550	CSDA
isotig01838	187.64	170.478	284.738	312.176	1383	0.02446081	isogroup00141	ENSG00000060138	ENST00000279550	CSDA
isotig01839	194.678	176.872	295.413	323.881	1333	0.024470204	isogroup00141	ENSG00000060138	ENST00000279550	CSDA
isotig01840	326.692	281.777	471.179	538.545	1188	0.041660404	isogroup00141	ENSG00000060138	ENST00000228251	CSDA
isotig01841	341.046	294.157	491.876	562.2	1138	0.041660404	isogroup00141	ENSG00000060138	ENST00000228251	CSDA
isotig01842	14.109	9.147	12.576	11.518	3110	0.491837003	isogroup00142	ENSG00000196531	ENST00000454682	NACA
isotig01843	12.785	8.12	11.709	11.047	3058	0.477521229	isogroup00142	ENSG00000196531	ENST00000454682	NACA
isotig01844	17.01	10.453	14.779	13.381	3020	0.537527241	isogroup00142	ENSG00000196531	ENST00000454682	NACA
isotig01845	12.977	8.336	11.951	11.256	3006	0.469367626	isogroup00142	ENSG00000196531	ENST00000454682	NACA
isotig01846	18.966	13.136	21.106	11.992	2146	0.718447058	isogroup00142	ENSG00000196531	ENST00000550952	NACA
isotig01847	41.742	27.828	47.812	30.328	1855	0.716352296	isogroup00142	ENSG00000196531	ENST00000454682	NACA
isotig01848	20.956	14.509	23.345	13.257	1937	0.722072969	isogroup00142	ENSG00000196531	ENST00000550952	NACA
isotig01849	46.976	31.309	53.838	34.145	1646	0.71837191	isogroup00142	ENSG00000196531	ENST00000454682	NACA
isotig01850	72.065	108.604	49.812	73.633	2428	0.706705118	isogroup00143	ENSG00000109971	ENST00000532636	HSPA8
isotig01851	78.289	118.823	54.096	80.069	2291	0.707578718	isogroup00143	ENSG00000109971	ENST00000532636	HSPA8
isotig01854	55.559	81.928	36.075	55.324	2291	0.697048546	isogroup00143	ENSG00000109971	ENST00000532636	HSPA8
isotig01855	61.127	91.1	39.759	61.005	2154	0.704957917	isogroup00143	ENSG00000109971	ENST00000532636	HSPA8
isotig01856	1139.341	615.824	768.421	586.959	580	0.781675058	isogroup00144	ENSG00000143947	ENST00000402285	RPS27A
isotig01857	4.568	3.483	2.702	2.508	6133	0.337115804	isogroup00145	ENSG00000107854	ENST00000371627	TNKS2
isotig01858	4.465	3.472	2.747	2.569	5944	0.325439618	isogroup00145	ENSG00000107854	ENST00000371627	TNKS2
isotig01859	3.526	3.16	3.218	2.672	4324	0.275249868	isogroup00145	ENSG00000107854	ENST00000371627	TNKS2
isotig01860	4.437	2.973	1.837	2.165	4198	0.347044788	isogroup00145	ENSG00000107854	ENST00000371627	TNKS2
isotig01861	3.515	3.232	2.895	2.317	3783	0.240493725	isogroup00145	ENSG00000173273	ENST00000518281	TNKS
isotig01862	4.278	2.932	1.864	2.239	4009	0.323279101	isogroup00145	ENSG00000107854	ENST00000371627	TNKS2
isotig01863	5.407	2.222	1.734	1.661	3655	0.662499061	isogroup00145	ENSG00000107854	ENST00000371627	TNKS2

isotig01864	14.698	21.753	19.412	19.074	2632	0.384365372	isogroup00145	ENSG00000165775	ENST00000369498	FUNDC2
isotig01865	2.217	1.936	1.201	1.379	1848	0.084682874	isogroup00145	ENSG00000173273	ENST00000518281	TNKS
isotig01866	31.297	48.501	41.677	41.096	1050	0.447593372	isogroup00145	ENSG00000165775	ENST00000369498	FUNDC2
isotig01873	21.732	12.084	17.007	11.164	760	0.743828436	isogroup00147	ENSG00000105258	ENST00000221859	POLR2I
isotig01874	30.697	16.748	24.02	15.785	512	0.755683099	isogroup00147	ENSG00000105258	ENST00000221859	POLR2I
isotig01878	174.474	157.357	181.339	147.157	865	0.366282783	isogroup00148	ENSG00000119421	ENST00000373768	NDUFA8
isotig01879	247.544	167.514	150.82	144.801	3750	0.524845946	isogroup00149	ENSG00000137154	ENST00000380394	RPS6
isotig01881	26.126	36.149	21.159	21.193	610	0.475435861	isogroup00150	ENSG00000115541	ENST00000233893	HSPE1
isotig01882	28.159	38.925	22.887	23.052	522	0.476797926	isogroup00150	ENSG00000115541	ENST00000233893	HSPE1
isotig01947	8.073	3.648	11.647	6.382	1443	0.79334185	isogroup00151	ENSG00000138606	ENST00000290894	SHF
isotig01965	12.336	5.554	10.429	8.616	6660	0.757082738	isogroup00152	ENSG00000081189	ENST00000510942	MEF2C
isotig01969	12.506	5.617	10.574	8.724	6615	0.758097242	isogroup00152	ENSG00000081189	ENST00000510942	MEF2C
isotig01989	13.06	5.853	10.913	9.192	6098	0.75214173	isogroup00152	ENSG00000081189	ENST00000510942	MEF2C
isotig01993	13.251	5.925	11.075	9.314	6053	0.756923048	isogroup00152	ENSG00000081189	ENST00000510942	MEF2C
isotig01996	4.933	6.437	5.9	7.405	4120	0.431464643	isogroup00153	ENSG00000092529	ENST00000397163	CAPN3
isotig01998	5.229	6.86	6.266	7.861	3940	0.444559255	isogroup00153	ENSG00000092529	ENST00000397163	CAPN3
isotig02000	4.843	6.302	5.781	7.329	3994	0.430403171	isogroup00153	ENSG00000092529	ENST00000397163	CAPN3
isotig02002	5.145	6.732	6.154	7.797	3814	0.443657474	isogroup00153	ENSG00000092529	ENST00000397163	CAPN3
isotig02004	5.517	7.305	6.65	8.38	3659	0.458893815	isogroup00153	ENSG00000092529	ENST00000397163	CAPN3
isotig02006	5.883	7.828	7.103	8.947	3479	0.465055986	isogroup00153	ENSG00000092529	ENST00000397163	CAPN3
isotig02008	5.436	7.183	6.542	8.329	3533	0.458367776	isogroup00153	ENSG00000092529	ENST00000397163	CAPN3
isotig02010	5.811	7.72	7.007	8.915	3353	0.464708424	isogroup00153	ENSG00000092529	ENST00000397163	CAPN3
isotig02012	3.131	3.925	3.693	4.296	2388	0.346424814	isogroup00153	ENSG00000092529	ENST00000397163	CAPN3
isotig02014	3.512	4.475	4.166	4.856	2208	0.376756594	isogroup00153	ENSG00000092529	ENST00000397163	CAPN3
isotig02016	2.872	3.547	3.36	3.989	2262	0.335913429	isogroup00153	ENSG00000092529	ENST00000397163	CAPN3
isotig02018	2.87	3.575	3.361	3.818	2181	0.326012625	isogroup00153	ENSG00000092529	ENST00000565274	CAPN3
isotig02019	5.828	7.726	6.97	9.069	2509	0.463581198	isogroup00153	ENSG00000092529	ENST00000397163	CAPN3
isotig02021	3.254	4.096	3.833	4.557	2082	0.368264823	isogroup00153	ENSG00000092529	ENST00000397163	CAPN3
isotig02023	3.267	4.148	3.854	4.394	2001	0.359998497	isogroup00153	ENSG00000092529	ENST00000569136	CAPN3
isotig02024	3.809	4.972	4.588	5.403	1927	0.406120839	isogroup00153	ENSG00000092529	ENST00000397200	CAPN3
isotig02026	2.569	3.136	2.974	3.452	2055	0.309113624	isogroup00153	ENSG00000092529	ENST00000565274	CAPN3
isotig02027	6.74	8.139	7.464	9.105	2341	0.3277974	isogroup00153	ENSG00000092529	ENST00000397163	CAPN3
isotig02028	4.361	5.774	5.279	6.226	1747	0.430008642	isogroup00153	ENSG00000092529	ENST00000397163	CAPN3
isotig02030	2.964	3.706	3.462	4.031	1875	0.347664763	isogroup00153	ENSG00000092529	ENST00000569136	CAPN3
isotig02031	3.531	4.57	4.233	5.095	1801	0.3978733	isogroup00153	ENSG00000092529	ENST00000397200	CAPN3
isotig02033	3.559	4.653	4.276	4.931	1720	0.390987826	isogroup00153	ENSG00000092529	ENST00000397163	CAPN3
isotig02034	4.095	5.389	4.938	5.948	1621	0.425668821	isogroup00153	ENSG00000092529	ENST00000397163	CAPN3
isotig02036	4.156	5.525	5.022	5.808	1540	0.424964305	isogroup00153	ENSG00000092529	ENST00000397163	CAPN3
isotig02037	3.226	4.173	3.849	4.546	1594	0.386572856	isogroup00153	ENSG00000092529	ENST00000397163	CAPN3
isotig02038	3.834	5.062	4.607	5.453	1414	0.41860487	isogroup00153	ENSG00000092529	ENST00000397163	CAPN3
isotig02039	2.284	2.879	2.57	3.223	777	0.301176073	isogroup00153	ENSG00000092529	ENST00000397200	CAPN3
isotig02075	3.696	1.619	1.726	1.266	391	0.602596378	isogroup00154	ENSG00000145284	ENST00000319540	SCD5
isotig02076	2.035	2.232	2.197	1.847	5662	0.028237018	isogroup00155	ENSG00000080503	ENST00000382203	SMARCA2
isotig02077	2.018	2.22	2.182	1.836	5608	0.028237018	isogroup00155	ENSG00000080503	ENST00000382203	SMARCA2
isotig02078	1.888	2.081	2.02	1.725	5626	0.041209514	isogroup00155	ENSG00000080503	ENST00000382203	SMARCA2
isotig02079	1.87	2.067	2.003	1.713	5572	0.041209514	isogroup00155	ENSG00000080503	ENST00000382203	SMARCA2
isotig02080	1.514	1.678	1.486	1.387	4799	0.079507026	isogroup00155	ENSG00000080503	ENST00000382203	SMARCA2
isotig02081	1.491	1.661	1.462	1.37	4745	0.079507026	isogroup00155	ENSG00000080503	ENST00000382194	SMARCA2
isotig02082	1.554	1.721	1.524	1.423	4671	0.079507026	isogroup00155	ENSG00000080503	ENST00000382203	SMARCA2
isotig02083	1.53	1.704	1.5	1.406	4617	0.079507026	isogroup00155	ENSG00000080503	ENST00000382194	SMARCA2
isotig02084	1.708	1.864	1.637	1.539	4371	0.071428571	isogroup00155	ENSG00000080503	ENST00000382203	SMARCA2
isotig02085	1.575	1.729	1.464	1.493	4102	0.098350492	isogroup00155	ENSG00000080503	ENST00000382194	SMARCA2
isotig02086	1.795	1.972	2.17	1.664	3139	0.039415345	isogroup00155	ENSG00000080503	ENST00000382203	SMARCA2
isotig02087	1.76	1.945	2.142	1.641	3085	0.041209514	isogroup00155	ENSG00000080503	ENST00000382194	SMARCA2
isotig02088	1.525	1.695	1.848	1.442	3103	0.016354174	isogroup00155	ENSG00000080503	ENST00000382203	SMARCA2
isotig02089	1.485	1.663	1.813	1.415	3049	0.016354174	isogroup00155	ENSG00000080503	ENST00000382194	SMARCA2
isotig02090	2.449	2.63	2.984	2.133	1888	0.091530773	isogroup00155	ENSG00000080503	ENST00000382186	SMARCA2
isotig02091	2.009	2.179	2.459	1.769	1852	0.063566168	isogroup00155	ENSG00000080503	ENST00000382186	SMARCA2
isotig02100	13.876	8.387	14.103	11.339	1231	0.598500789	isogroup00156	ENSG00000159352	ENST00000368881	PSMD4
isotig02101	30.477	16.24	31.59	19.13	1150	0.79318216	isogroup00156	ENSG00000159352	ENST00000445776	PSMD4
isotig02102	8.492	4.826	8.04	5.982	1089	0.651649508	isogroup00156	ENSG00000159352	ENST00000437736	PSMD4
isotig02103	16.103	10.314	16.73	14.071	1062	0.529439393	isogroup00156	ENSG00000159352	ENST00000368881	PSMD4
isotig02104	14.091	8.78	14.737	12.177	1029	0.559479973	isogroup00156	ENSG00000159352	ENST00000368881	PSMD4
isotig02105	10.074	6.396	9.959	8.151	920	0.531646878	isogroup00156	ENSG00000159352	ENST00000437736	PSMD4
isotig02106	7.515	4.471	7.395	5.735	887	0.586927933	isogroup00156	ENSG00000159352	ENST00000437736	PSMD4
isotig02115	45.671	29.79	50	36.728	563	0.618386939	isogroup00156	ENSG00000159352	ENST00000445776	PSMD4
isotig02120	23.683	15.622	27.367	19.81	463	0.60598745	isogroup00156	ENSG00000159352	ENST00000368881	PSMD4
isotig02140	20.949	21.894	21.591	17.472	3738	0.14586308	isogroup00158	ENSG00000151726	ENST00000515030	ACSL1
isotig02141	19.965	20.015	20.404	16.004	3065	0.219358233	isogroup00158	ENSG00000151726	ENST00000515030	ACSL1
isotig02142	20.986	21.282	21.845	17.038	2742	0.21982791	isogroup00158	ENSG00000151726	ENST00000515030	ACSL1
isotig02143	24.006	23.97	24.938	19.326	2236	0.237816563	isogroup00158	ENSG00000151726	ENST00000515030	ACSL1
isotig02144	10.555	11.289	10.003	8.801	2582	0.003503795	isogroup00158	ENSG00000151726	ENST00000515030	ACSL1
isotig02145	11.145	12.342	11.031	9.694	2507	0.021999699	isogroup00158	ENSG00000151726	ENST00000515030	ACSL1
isotig02146	7.332	5.65	7.15	5.678	1991	0.434235741	isogroup00158	ENSG00000047249	ENST00000396774	ATP6V1H
isotig02147	5.31	4.533	4.011	3.387	1909	0.246374089	isogroup00158	ENSG00000151726	ENST00000515030	ACSL1
isotig02148	5.902	5.697	5.171	4.386	1834	0.182469753	isogroup00158	ENSG00000151726	ENST00000515030	ACSL1
isotig02149	12.988	14.498	12.816	11.377	1730	0.067614789	isogroup00158	ENSG00000151726	ENST00000515030	ACSL1
isotig02150	4.091	3.57	3.165	2.606	1586	0.226102803	isogroup00158	ENSG00000151726	ENST00000515030	ACSL1
isotig02151	4.749	4.935	4.53	3.78	1511	0.139710303	isogroup00158	ENSG00000151726	ENST00000515030	ACSL1
isotig02152	13.726	14.383	12.817	11.377	1407	0.037874803	isogroup00158	ENSG00000151726	ENST00000515030	ACSL1
isotig02153	5.066	4.341	3.784	3.239	1057	0.23971406	isogroup00158	ENSG00000151726	ENST00000515030	ACSL1
isotig02154	3.294	2.687	2.694	2.194	1005	0.340037198	isogroup00158	ENSG00000151726	ENST00000515030	ACSL1

isotig02155	2.324	2.176	1.856	1.486	734	0.151724656	isogroup00158	ENSG00000151726	ENST00000515030	ACSL1
isotig02156	715.04	555.385	461.002	397.628	449	0.472026001	isogroup00159	ENSG00000109475	ENST00000506397	RPL34
isotig02158	395.506	331.71	269.701	248.965	1082	0.338881792	isogroup00161	ENSG00000089009	ENST00000424576	RPL6
isotig02159	4.054	3.039	3.528	4.812	335	0.014428496	isogroup00162	ENSG00000139637	ENST00000545214	C12orf10
isotig02165	6.707	5.353	8.092	5.021	3652	0.573748779	isogroup00163	ENSG00000093167	ENST00000421307	LRRFIP2
isotig02166	7.385	5.867	8.847	5.636	3343	0.575045089	isogroup00163	ENSG00000093167	ENST00000421307	LRRFIP2
isotig02167	8.09	6.193	9.588	5.663	2654	0.635116856	isogroup00163	ENSG00000093167	ENST00000421307	LRRFIP2
isotig02168	4.559	3.569	5.651	3.631	2297	0.530275419	isogroup00163	ENSG00000093167	ENST00000421307	LRRFIP2
isotig02169	4.968	4.084	5.458	3.516	1997	0.485336665	isogroup00163	ENSG00000093167	ENST00000440230	LRRFIP2
isotig02170	5.365	4.155	6.541	4.449	1988	0.524357481	isogroup00163	ENSG00000093167	ENST00000421307	LRRFIP2
isotig02171	2.26	1.189	2.553	2.195	1869	0.383125423	isogroup00163	ENSG00000093167	ENST00000452742	LRRFIP2
isotig02172	8.634	6.756	9.951	5.936	1621	0.620284437	isogroup00163	ENSG00000093167	ENST00000396428	LRRFIP2
isotig02173	6.804	5.67	7.982	4.959	1578	0.545849929	isogroup00163	ENSG00000093167	ENST00000440230	LRRFIP2
isotig02174	2.833	1.464	3.073	2.953	1560	0.377667769	isogroup00163	ENSG00000093167	ENST00000452742	LRRFIP2
isotig02175	3.036	2.182	3.459	2.124	1663	0.498590967	isogroup00163	ENSG00000093167	ENST00000421307	LRRFIP2
isotig02176	3.873	2.725	4.265	2.98	1354	0.497313444	isogroup00163	ENSG00000093167	ENST00000421307	LRRFIP2
isotig02177	5.734	3.915	6.832	3.875	1299	0.669910573	isogroup00163	ENSG00000093167	ENST00000421307	LRRFIP2
isotig02178	109.544	128.37	118.647	95.433	487	0.041078004	isogroup00164	ENSG00000125356	ENST00000371437	NDUFA1
isotig02179	114.4	134.455	124.828	99.693	437	0.041124972	isogroup00164	ENSG00000125356	ENST00000371437	NDUFA1
isotig02180	123.671	144.935	133.852	107.726	431	0.041124972	isogroup00164	ENSG00000125356	ENST00000371437	NDUFA1
contig05167	2.688	1.49	2.635	1.625	1226	0.536118208	isogroup00165	ENSG00000234545	ENST00000445716	FAM133B
isotig02187	3.718	4.868	4.893	4.38	2560	0.067426918	isogroup00165	ENSG00000215712	ENST00000400788	TMEM242
isotig02188	3.82	5.013	5.024	4.508	2482	0.069775306	isogroup00165	ENSG00000215712	ENST00000400788	TMEM242
isotig02192	3.375	4.201	4.322	3.855	2371	0.054877132	isogroup00165	ENSG00000215712	ENST00000400788	TMEM242
isotig02195	5.971	3.43	6.26	3.784	2776	0.729719321	isogroup00166	ENSG00000161265	ENST00000412391	U2AF1L4
isotig02196	5.443	3.126	5.682	3.41	2770	0.714605095	isogroup00166	ENSG00000161265	ENST00000412391	U2AF1L4
isotig02197	5.658	3.25	5.953	3.583	2707	0.723078079	isogroup00166	ENSG00000161265	ENST00000412391	U2AF1L4
isotig02198	5.069	2.723	5.387	3.147	2587	0.733279477	isogroup00166	ENSG00000126246	ENST00000246532	IGFLR1
isotig02199	5.969	5.112	6.851	4.058	1083	0.516776884	isogroup00166	ENSG00000126246	ENST00000246532	IGFLR1
isotig02200	5.48	2.402	6.269	3.635	783	0.782201097	isogroup00166	ENSG00000126246	ENST00000246532	IGFLR1
isotig02201	7.41	5.021	7.209	4.623	608	0.682234914	isogroup00166	ENSG00000161265	ENST00000412391	U2AF1L4
isotig02202	4.995	3.64	4.558	2.914	602	0.5858101	isogroup00166	ENSG00000161265	ENST00000412391	U2AF1L4
isotig02203	6.024	4.323	5.785	3.72	539	0.631124596	isogroup00166	ENSG00000161265	ENST00000412391	U2AF1L4
isotig02205	3.064	6.64	4.131	5.585	1723	0.677763583	isogroup00167	ENSG00000132423	ENST00000254759	COQ3
isotig02206	486.655	358.589	381.11	352.497	984	0.447057939	isogroup00167	ENSG00000137818	ENST00000260379	RPLP1
isotig02207	505.944	371.38	395.198	365.191	951	0.447161269	isogroup00167	ENSG00000137818	ENST00000260379	RPLP1
isotig02208	2.821	6.786	3.517	5.058	1352	0.717930413	isogroup00167	ENSG00000132423	ENST00000254759	COQ3
isotig02209	746.398	549.048	584.201	540.268	641	0.447612159	isogroup00167	ENSG00000137818	ENST00000260379	RPLP1
isotig02213	507.652	374.053	397.608	367.752	943	0.447161269	isogroup00167	ENSG00000137818	ENST00000260379	RPLP1
isotig02216	690.047	485.756	503.323	480.888	872	0.460265274	isogroup00168	ENSG00000149273	ENST00000278572	RPS3
isotig02218	7.194	4.675	4.05	3.525	4261	0.430168332	isogroup00169	ENSG00000138592	ENST00000433963	USP8
isotig02220	1.957	1.352	1.812	1.979	2907	0.186011122	isogroup00169	ENSG00000164877	ENST00000405088	MICALL2
isotig02222	2.238	1.612	1.631	1.038	2335	0.339699031	isogroup00169	ENSG00000143549	ENST00000368531	TPM3
isotig02225	2.171	1.879	1.798	1.06	2097	0.258454197	isogroup00169	ENSG00000142676	ENST00000458455	RPL11
isotig02226	3.397	2.063	2.731	1.829	1803	0.496824979	isogroup00169	ENSG00000138592	ENST00000433963	USP8
contig05253	3.16	3.874	3.627	2.748	611	0.061837755	isogroup00170	ENSG00000100567	ENST00000553677	PSMA3
isotig02229	19.192	13.065	18.837	14.605	2034	0.633736004	isogroup00170	ENSG00000100567	ENST00000216455	PSMA3
isotig02231	35.941	22.563	35.31	26.599	1015	0.705296085	isogroup00170	ENSG00000100567	ENST00000216455	PSMA3
isotig02233	14.064	9.24	13.989	10.651	550	0.672371684	isogroup00170	ENSG00000100567	ENST00000412908	PSMA3
isotig02235	9.026	4.935	8.567	5.143	1409	0.740813106	isogroup00171	ENSG00000225312	ENST00000454191	HSD17B8
isotig02236	10.246	5.816	9.736	5.996	1311	0.733899451	isogroup00171	ENSG00000225312	ENST00000454191	HSD17B8
isotig02237	9.578	5.621	9.31	5.484	1218	0.729024198	isogroup00171	ENSG00000225312	ENST00000454191	HSD17B8
isotig02238	11.054	6.712	10.742	6.513	1120	0.721800556	isogroup00171	ENSG00000225312	ENST00000454191	HSD17B8
isotig02239	8.722	4.636	8.11	4.605	880	0.754189524	isogroup00171	ENSG00000225312	ENST00000454191	HSD17B8
isotig02240	9.614	5.765	9.295	5.058	689	0.733777335	isogroup00171	ENSG00000225312	ENST00000454191	HSD17B8
isotig02252	2.789	1.356	2.813	1.962	550	0.53062298	isogroup00173	ENSG00000115657	ENST00000427013	ABCB6
isotig02254	53.702	62.639	58.81	52.245	746	0.151546179	isogroup00174	ENSG00000129559	ENST00000250495	NEDD8
isotig02255	7.592	10.695	8.381	9.766	4783	0.477586984	isogroup00175	ENSG00000152601	ENST00000324210	MBNL1
isotig02256	7.664	10.863	8.458	9.859	4747	0.479465695	isogroup00175	ENSG00000152601	ENST00000355460	MBNL1
isotig02257	7.996	11.454	8.762	10.338	4672	0.500450891	isogroup00175	ENSG00000152601	ENST00000324210	MBNL1
isotig02258	8.073	11.633	8.844	10.438	4636	0.501737807	isogroup00175	ENSG00000152601	ENST00000355460	MBNL1
isotig02259	2.128	1.571	1.841	1.207	3287	0.290044713	isogroup00176	ENSG00000155755	ENST00000409444	TMEM237
isotig02262	2.032	1.632	2.581	1.646	1683	0.389691516	isogroup00176	ENSG00000105327	ENST00000449228	BBC3
isotig02265	1.345	1.326	2.593	2.909	1210	0.128719847	isogroup00176	ENSG00000197603	ENST00000508244	C5orf42
isotig02268	566.182	769.443	110.467	475.402	1768	0.775080785	isogroup00177	ENSG00000164879	ENST00000285381	CA3
isotig02269	584.256	794.015	113.992	490.601	1713	0.775146539	isogroup00177	ENSG00000164879	ENST00000285381	CA3
isotig02270	351.645	460.092	60.562	286.335	555	0.750901781	isogroup00177	ENSG00000164879	ENST00000285381	CA3
isotig02271	894.771	589.953	635.664	592.279	876	0.559996618	isogroup00178	ENSG00000188846	ENST00000396203	RPL14
isotig02273	32.135	18.991	30.028	24.201	3146	0.626192981	isogroup00179	ENSG00000175166	ENST00000538096	PSMD2
isotig02274	30.628	18.189	28.78	23.261	3098	0.625122116	isogroup00179	ENSG00000175166	ENST00000538096	PSMD2
isotig02275	34.948	20.477	32.507	26.236	2992	0.628259563	isogroup00179	ENSG00000175166	ENST00000310118	PSMD2
isotig02276	33.408	19.657	31.234	25.28	2944	0.627057188	isogroup00179	ENSG00000175166	ENST00000310118	PSMD2
isotig02278	114.005	101.821	134.963	111.578	2141	0.293351244	isogroup00180	ENSG00000160014	ENST00000391918	CALM3
contig05438	32.547	8.499	31.984	9.249	748	0.96483054	isogroup00181	ENSG00000186635	ENST00000455638	ARAP1
isotig02279	72.61	58.214	63.917	28.414	1066	0.690407304	isogroup00181	ENSG00000092841	ENST00000550697	MYL6
isotig02280	76.388	61.076	67.093	29.967	1007	0.691036672	isogroup00181	ENSG00000092841	ENST00000550697	MYL6
isotig02281	56.11	35.138	42.97	16.826	1083	0.856372586	isogroup00181	ENSG00000092841	ENST00000551589	MYL6
isotig02283	207.253	218.17	214.966	163.749	736	0.186499587	isogroup00182	ENSG00000167863	ENST00000301587	ATP5H
isotig02284	209.243	220.239	216.955	165.368	729	0.18650898	isogroup00182	ENSG00000167863	ENST00000301587	ATP5H
isotig02285	2.497	2.968	2.607	2.453	680	0.046479297	isogroup00182	ENSG00000145911	ENST00000274605	N4BP3
isotig02298	1.281	1.076	1.354	1.018	5132	0.167674908	isogroup00183	ENSG00000130653	ENST00000406427	PNPLA7
isotig02396	2.151	2.02	2.828	3.007	6763	0.071109191	isogroup00186	ENSG00000079393	ENST00000372702	DUSP13

isotig02397	2.167	2.035	2.85	3.031	6711	0.071109191	isogroup00186	ENSG00000079393	ENST00000372702	DUSP13
isotig02398	2.283	2.186	2.993	3.209	6062	0.094423987	isogroup00186	ENSG00000079393	ENST00000491677	DUSP13
isotig02399	2.449	2.284	3.231	3.466	6009	0.074481476	isogroup00186	ENSG00000079393	ENST00000372702	DUSP13
isotig02400	2.303	2.204	3.019	3.237	6010	0.094423987	isogroup00186	ENSG00000079393	ENST00000491677	DUSP13
isotig02401	2.471	2.304	3.26	3.496	5957	0.074481476	isogroup00186	ENSG00000079393	ENST00000372702	DUSP13
isotig02402	2.295	2.103	3.073	3.253	5797	0.05706583	isogroup00186	ENSG00000079393	ENST00000491677	DUSP13
isotig02403	2.315	2.122	3.101	3.283	5745	0.05706583	isogroup00186	ENSG00000079393	ENST00000491677	DUSP13
isotig02404	2.384	2.203	3.244	3.442	5569	0.06991621	isogroup00186	ENSG00000079393	ENST00000491677	DUSP13
isotig02405	2.407	2.224	3.274	3.475	5517	0.06991621	isogroup00186	ENSG00000079393	ENST00000491677	DUSP13
isotig02406	2.64	2.508	3.474	3.757	5308	0.099938003	isogroup00186	ENSG00000079393	ENST00000491677	DUSP13
isotig02407	2.666	2.533	3.508	3.794	5256	0.102370933	isogroup00186	ENSG00000079393	ENST00000491677	DUSP13
isotig02408	2.472	2.312	3.304	3.527	5096	0.087162771	isogroup00186	ENSG00000079393	ENST00000491677	DUSP13
isotig02409	2.498	2.335	3.338	3.563	5044	0.089605095	isogroup00186	ENSG00000079393	ENST00000491677	DUSP13
isotig02410	2.583	2.436	3.51	3.757	4868	0.099938003	isogroup00186	ENSG00000079393	ENST00000491677	DUSP13
isotig02411	2.611	2.462	3.548	3.797	4816	0.099938003	isogroup00186	ENSG00000079393	ENST00000491677	DUSP13
isotig02415	2.205	2.292	2.34	3.025	1918	0.230771399	isogroup00186	ENSG00000079393	ENST00000372702	DUSP13
isotig02422	1.888	1.99	2.122	2.804	1478	0.271248215	isogroup00186	ENSG00000079393	ENST00000491677	DUSP13
isotig02423	2.479	2.861	2.651	3.428	987	0.263686406	isogroup00186	ENSG00000079393	ENST00000372702	DUSP13
isotig02424	2.594	2.822	2.736	3.136	764	0.156205381	isogroup00186	ENSG00000079393	ENST00000372702	DUSP13
isotig02427	1.841	2.503	2.313	3.157	547	0.382636958	isogroup00186	ENSG00000079393	ENST00000491677	DUSP13
isotig02428	3.776	2.729	3.734	3.392	4112	0.249727587	isogroup00187	ENSG00000148660	ENST00000433289	CAMK2G
isotig02429	3.75	2.717	3.704	3.362	4076	0.245885624	isogroup00187	ENSG00000148660	ENST00000322635	CAMK2G
isotig02430	3.754	2.709	3.7	3.379	4028	0.247820696	isogroup00187	ENSG00000148660	ENST00000433289	CAMK2G
isotig02431	3.727	2.697	3.669	3.348	3992	0.245885624	isogroup00187	ENSG00000148660	ENST00000322635	CAMK2G
isotig02432	4.16	3.06	4.115	3.784	3679	0.236942962	isogroup00187	ENSG00000148660	ENST00000433289	CAMK2G
isotig02433	4.134	3.05	4.084	3.754	3643	0.233645826	isogroup00187	ENSG00000148660	ENST00000322635	CAMK2G
isotig02434	4.144	3.045	4.085	3.779	3595	0.235317878	isogroup00187	ENSG00000148660	ENST00000433289	CAMK2G
isotig02465	1.905	2.137	2.097	1.605	1541	0.039415345	isogroup00188	ENSG00000010810	ENST00000368667	FYN
isotig02501	2.862	2.458	3.163	2.474	5688	0.332202976	isogroup00190	ENSG00000157119	ENST00000287777	KBTBD5
isotig02502	2.953	2.514	3.272	2.568	5487	0.336843391	isogroup00190	ENSG00000157119	ENST00000287777	KBTBD5
isotig02503	2.872	2.466	3.174	2.534	5275	0.315839408	isogroup00190	ENSG00000157119	ENST00000287777	KBTBD5
isotig02504	2.972	2.528	3.293	2.638	5074	0.323279101	isogroup00190	ENSG00000157119	ENST00000287777	KBTBD5
isotig02505	2.995	2.807	3.325	2.704	4730	0.282849252	isogroup00190	ENSG00000157119	ENST00000287777	KBTBD5
isotig02506	4.065	3.645	3.971	4.375	4835	0.040580146	isogroup00190	ENSG00000157119	ENST00000287777	KBTBD5
isotig02507	3.453	3.119	3.4	3.604	4723	0.079835801	isogroup00190	ENSG00000157119	ENST00000287777	KBTBD5
isotig02508	3.112	2.891	3.466	2.828	4529	0.287414519	isogroup00190	ENSG00000157119	ENST00000287777	KBTBD5
isotig02509	4.225	3.763	4.136	4.569	4634	0.040580146	isogroup00190	ENSG00000157119	ENST00000287777	KBTBD5
isotig02510	3.591	3.217	3.543	3.768	4522	0.076068986	isogroup00190	ENSG00000157119	ENST00000287777	KBTBD5
isotig02511	3.021	2.851	3.354	2.798	4317	0.252226272	isogroup00190	ENSG00000157119	ENST00000287777	KBTBD5
isotig02512	4.19	3.765	4.06	4.623	4422	0.014428496	isogroup00190	ENSG00000157119	ENST00000287777	KBTBD5
isotig02513	3.523	3.193	3.436	3.784	4310	0.029937251	isogroup00190	ENSG00000157119	ENST00000287777	KBTBD5
isotig02514	3.151	2.945	3.51	2.939	4116	0.256838506	isogroup00190	ENSG00000157119	ENST00000287777	KBTBD5
isotig02515	4.372	3.901	4.245	4.848	4221	0.014428496	isogroup00190	ENSG00000157119	ENST00000287777	KBTBD5
isotig02516	2.685	2.426	2.857	2.55	4190	0.20724055	isogroup00190	ENSG00000157119	ENST00000287777	KBTBD5
isotig02517	3.678	3.304	3.596	3.974	4109	0.029937251	isogroup00190	ENSG00000157119	ENST00000287777	KBTBD5
isotig02518	2.802	2.501	2.992	2.683	3989	0.210312242	isogroup00190	ENSG00000157119	ENST00000287777	KBTBD5
isotig02519	2.68	2.433	2.838	2.641	3777	0.160639137	isogroup00190	ENSG00000157119	ENST00000287777	KBTBD5
isotig02520	2.811	2.519	2.989	2.794	3576	0.166453746	isogroup00190	ENSG00000157119	ENST00000287777	KBTBD5
isotig02521	5.653	4.217	5.105	6.211	2072	0.032651988	isogroup00190	ENSG00000010282	ENST00000441594	HHATL
isotig02522	4.269	2.983	3.792	4.458	1960	0.110299091	isogroup00190	ENSG00000010282	ENST00000441594	HHATL
isotig03067	3.326	3.589	3.026	2.972	1912	0.006660029	isogroup00221	ENSG00000089159	ENST00000267257	PXN
isotig03070	2.156	1.444	1.885	1.613	1428	0.314543098	isogroup00221	ENSG00000089159	ENST00000267257	PXN
isotig03101	3.555	3.423	7.131	3.53	6558	0.450721425	isogroup00223	ENSG00000196924	ENST00000422373	FLNA
isotig03103	3.626	3.495	7.277	3.604	6460	0.4530886	isogroup00223	ENSG00000196924	ENST00000369850	FLNA
isotig03105	2.305	2.148	5.743	2.276	6098	0.547456226	isogroup00223	ENSG00000196924	ENST00000422373	FLNA
isotig03107	2.361	2.205	5.878	2.336	6000	0.551147892	isogroup00223	ENSG00000196924	ENST00000369850	FLNA
isotig03109	3.342	3.219	6.126	3.352	5761	0.368293004	isogroup00223	ENSG00000196924	ENST00000422373	FLNA
isotig03111	2.462	2.29	6.136	2.423	5680	0.563425265	isogroup00223	ENSG00000196924	ENST00000369850	FLNA
isotig03112	3.419	3.297	6.276	3.434	5663	0.370566243	isogroup00223	ENSG00000196924	ENST00000369850	FLNA
isotig03115	2.525	2.354	6.288	2.49	5582	0.56663786	isogroup00223	ENSG00000196924	ENST00000369850	FLNA
isotig03117	1.885	1.734	4.442	1.894	5301	0.473463215	isogroup00223	ENSG00000196924	ENST00000422373	FLNA
isotig03119	1.942	1.792	4.574	1.956	5203	0.479352972	isogroup00223	ENSG00000196924	ENST00000369850	FLNA
isotig03121	2.033	1.864	4.788	2.032	4883	0.493640565	isogroup00223	ENSG00000196924	ENST00000369850	FLNA
isotig03123	2.097	1.929	4.937	2.103	4785	0.499173367	isogroup00223	ENSG00000196924	ENST00000369850	FLNA
isotig03125	3.005	2.755	8.376	2.893	1657	0.670098444	isogroup00223	ENSG00000196924	ENST00000422373	FLNA
isotig03126	3.038	2.763	8.108	2.666	1544	0.671225671	isogroup00223	ENSG00000196924	ENST00000422373	FLNA
isotig03140	2.224	3.065	1.842	1.759	2906	0.256209138	isogroup00224	ENSG00000124831	ENST00000391999	LRRFIP1
isotig03141	2.296	3.159	1.897	1.822	2864	0.259590817	isogroup00224	ENSG00000124831	ENST00000391999	LRRFIP1
isotig03142	2.163	2.973	1.807	1.722	2834	0.246129856	isogroup00224	ENSG00000124831	ENST00000391999	LRRFIP1
isotig03143	2.236	3.067	1.862	1.786	2792	0.253485008	isogroup00224	ENSG00000124831	ENST00000391999	LRRFIP1
isotig03144	2.348	3.263	1.942	1.875	2768	0.275710153	isogroup00224	ENSG00000124831	ENST00000308482	LRRFIP1
isotig03145	2.425	3.364	2.001	1.942	2726	0.282032013	isogroup00224	ENSG00000124831	ENST00000308482	LRRFIP1
isotig03146	2.287	3.17	1.907	1.839	2696	0.268928008	isogroup00224	ENSG00000124831	ENST00000308482	LRRFIP1
isotig03147	2.366	3.273	1.967	1.908	2654	0.275710153	isogroup00224	ENSG00000124831	ENST00000308482	LRRFIP1
isotig03148	2.326	3.143	1.961	1.811	2393	0.237074472	isogroup00224	ENSG00000124831	ENST00000391999	LRRFIP1
isotig03149	2.255	3.033	1.92	1.767	2321	0.224045615	isogroup00224	ENSG00000124831	ENST00000391999	LRRFIP1
isotig03150	2.485	3.39	2.09	1.955	2255	0.259590817	isogroup00224	ENSG00000124831	ENST00000391999	LRRFIP1
isotig03151	2.414	3.28	2.051	1.914	2183	0.253503795	isogroup00224	ENSG00000124831	ENST00000391999	LRRFIP1
isotig03153	1.768	2.672	1.221	1.221	734	0.254236492	isogroup00224	ENSG00000124831	ENST00000308482	LRRFIP1
isotig03155	2.338	2.678	2.492	2.389	6499	0.119636282	isogroup00225	ENSG00000160679	ENST00000368687	CHTOP
isotig03156	2.47	2.899	2.713	2.619	5884	0.142387465	isogroup00225	ENSG00000160679	ENST00000368687	CHTOP
isotig03157	2.649	2.79	2.78	2.546	5168	0.025447133	isogroup00225	ENSG00000160679	ENST00000368687	CHTOP

isotig03158	2.862	3.09	3.104	2.865	4553	0.05706583	isogroup00225	ENSG00000160679	ENST00000368687	CHTOP
isotig03161	1.111	1.289	1.004	1.112	4552	0.106776509	isogroup00225	ENSG00000160679	ENST00000368687	CHTOP
isotig03162	3.317	4.197	3.897	3.804	3926	0.235317878	isogroup00225	ENSG00000160679	ENST00000368694	CHTOP
isotig03166	5.01	5.233	5.501	4.454	2931	0.145693996	isogroup00225	ENSG00000160679	ENST00000403433	CHTOP
isotig03169	4.439	5.2	5.19	4.844	2595	0.145693996	isogroup00225	ENSG00000160679	ENST00000368694	CHTOP
isotig03172	1.367	2.039	1.505	1.768	2594	0.290739836	isogroup00225	ENSG00000160679	ENST00000368694	CHTOP
isotig03174	1.316	2.105	1.478	1.845	2090	0.34768355	isogroup00225	ENSG00000160679	ENST00000368694	CHTOP
isotig03176	1.618	1.823	1.643	1.759	1263	0.084682874	isogroup00225	ENSG00000160679	ENST00000368694	CHTOP
isotig03178	1.644	1.862	1.66	1.967	759	0.13859247	isogroup00225	ENSG00000160679	ENST00000368694	CHTOP
isotig03181	2.327	1.618	2.508	2.768	2620	0.163504171	isogroup00226	ENSG00000100644	ENST00000539097	HIF1A
isotig03182	2.551	1.809	2.886	3.218	2405	0.167580972	isogroup00226	ENSG00000100644	ENST00000539097	HIF1A
isotig03183	1.841	1.229	2.066	1.555	1396	0.306389494	isogroup00226	ENSG00000100644	ENST00000539097	HIF1A
isotig03184	1.766	1.257	1.754	1.254	1362	0.261892237	isogroup00226	ENSG00000100644	ENST00000539097	HIF1A
isotig03185	1.98	1.456	2.257	1.741	1276	0.292477643	isogroup00226	ENSG00000100644	ENST00000539097	HIF1A
isotig03186	1.902	1.494	1.92	1.416	1242	0.243687533	isogroup00226	ENSG00000100644	ENST00000539097	HIF1A
isotig03189	2.159	1.208	1.957	1.787	1204	0.322057939	isogroup00226	ENSG00000100644	ENST00000539097	HIF1A
isotig03190	2.13	1.591	2.405	1.913	1147	0.288203577	isogroup00226	ENSG00000100644	ENST00000539097	HIF1A
isotig03193	2.082	1.241	1.591	1.443	1170	0.271783648	isogroup00226	ENSG00000100644	ENST00000539097	HIF1A
isotig03194	2.337	1.916	2.683	2.186	1027	0.268928008	isogroup00226	ENSG00000100644	ENST00000539097	HIF1A
isotig03197	2.591	1.638	2.336	2.276	955	0.302791764	isogroup00226	ENSG00000100644	ENST00000539097	HIF1A
isotig03215	3.928	8.381	4.523	13.291	6064	0.901827985	isogroup00228	ENSG00000170525	ENST00000379782	PFKFB3
isotig03216	2.072	4.73	2.075	6.941	6021	0.853432404	isogroup00228	ENSG00000170525	ENST00000379782	PFKFB3
isotig03217	2.932	6.342	3.593	10.06	5797	0.876803562	isogroup00228	ENSG00000170525	ENST00000540253	PFKFB3
isotig03219	3.471	7.593	4.326	12.068	4632	0.891767491	isogroup00228	ENSG00000170525	ENST00000540253	PFKFB3
isotig03220	5.03	10.644	5.811	17.048	4592	0.915796197	isogroup00228	ENSG00000170525	ENST00000358499	PFKFB3
isotig03222	2.562	5.765	2.541	8.57	4549	0.877104156	isogroup00228	ENSG00000170525	ENST00000379782	PFKFB3
isotig03223	3.764	8.05	4.645	12.949	4325	0.895693996	isogroup00228	ENSG00000170525	ENST00000358499	PFKFB3
isotig03225	5.743	14.582	7.346	22.249	4004	0.93333396	isogroup00228	ENSG00000196531	ENST00000454682	NACA
isotig03226	4.86	10.513	6.106	16.959	3160	0.911785151	isogroup00228	ENSG00000170525	ENST00000358499	PFKFB3
isotig03228	3.827	7.762	3.712	12.167	2101	0.901527392	isogroup00228	ENSG00000170525	ENST00000490474	PFKFB3
isotig03230	4.535	9.328	4.312	14.568	1569	0.913776584	isogroup00228	ENSG00000170525	ENST00000490474	PFKFB3
isotig03231	2.879	5.904	2.835	9.949	846	0.88486323	isogroup00228	ENSG00000170525	ENST00000490474	PFKFB3
isotig03238	3.575	2.851	3.35	3.347	3097	0.241818216	isogroup00229	ENSG00000095319	ENST00000372577	NUP188
isotig03249	18.996	27.102	16.007	17.98	4324	0.521407906	isogroup00230	ENSG00000154415	ENST00000284601	PPP1R3A
isotig03251	17.164	23.855	12.458	15.098	3526	0.545229954	isogroup00230	ENSG00000154415	ENST00000284601	PPP1R3A
isotig03257	11.712	18.225	17.113	16.913	2985	0.405641767	isogroup00230	ENSG00000155657	ENST00000342175	TTN
isotig03259	11.099	11.778	6.665	8.053	1245	0.247257083	isogroup00230	ENSG00000154415	ENST00000284601	PPP1R3A
isotig03276	1.731	1.665	1.405	1.1	3727	0.048414368	isogroup00232	ENSG00000130844	ENST00000509047	ZNF331
isotig03278	1.683	1.612	1.345	1.086	3651	0.042270985	isogroup00232	ENSG00000130844	ENST00000514022	ZNF331
isotig03279	7.053	5.795	7.742	6.116	1388	0.33136695	isogroup00232	ENSG00000130844	ENST00000511154	ZNF331
isotig03280	1.366	1.469	1.277	1.093	983	0.029392425	isogroup00232	ENSG00000130844	ENST00000509047	ZNF331
isotig03284	1.14	1.238	1.022	1.037	907	0.068525964	isogroup00232	ENSG00000130844	ENST00000514022	ZNF331
contig06241	45.166	30.369	57.121	36.412	1743	0.724393177	isogroup00233	ENSG00000067064	ENST00000381344	IDI1
contig06242	21.381	17.642	25.986	20.919	793	0.531844142	isogroup00233	ENSG00000067064	ENST00000381344	IDI1
contig06256	261.742	303.483	345.922	304.442	1215	0.075185992	isogroup00234	ENSG00000158864	ENST00000392179	NDUFS2
isotig03289	3.513	5.775	5.393	3.488	1981	0.65218494	isogroup00235	ENSG00000013441	ENST00000357369	CLK1
isotig03291	3.811	7.405	6.051	4.473	1766	0.748844593	isogroup00235	ENSG00000013441	ENST00000434813	CLK1
isotig03293	3.232	5.266	4.822	3.098	1626	0.641279026	isogroup00235	ENSG00000013441	ENST00000357369	CLK1
isotig03294	3.562	7.228	5.559	4.271	1411	0.768674382	isogroup00235	ENSG00000013441	ENST00000434813	CLK1
isotig03295	5.322	8.824	6.371	4.176	1167	0.723078079	isogroup00235	ENSG00000013441	ENST00000432425	CLK1
isotig03296	2.565	4.438	4.03	2.671	803	0.583518073	isogroup00235	ENSG00000013441	ENST00000434813	CLK1
isotig03298	3.157	4.285	4.031	2.47	734	0.426044563	isogroup00235	ENSG00000013441	ENST00000434813	CLK1
contig06295	2.185	1.776	3.101	1.308	804	0.309977831	isogroup00236	ENSG00000203667	ENST00000411948	FAM36A
contig06296	6.189	5.032	5.269	3.43	855	0.414969565	isogroup00236	ENSG00000203667	ENST00000411948	FAM36A
isotig03301	606.632	602.68	740.116	679.231	1912	0.20295709	isogroup00236	ENSG00000198888	ENST00000361390	MT-ND1
isotig03303	3.259	7.231	3.795	4.696	4975	0.686292929	isogroup00238	ENSG00000130695	ENST00000476272	CEP85
isotig03304	4.026	8.486	4.777	5.793	3864	0.67392162	isogroup00238	ENSG00000130695	ENST00000451429	CEP85
isotig03305	4.039	9.81	4.42	5.916	3753	0.790655294	isogroup00238	ENSG00000130695	ENST00000451429	CEP85
isotig03306	1.628	4.608	1.514	2.341	3494	0.726666416	isogroup00238	ENSG00000130695	ENST00000476272	CEP85
isotig03307	1.557	3.826	1.551	2.144	3446	0.624426993	isogroup00238	ENSG00000130695	ENST00000476272	CEP85
isotig03309	4.685	11.069	5.754	7.054	2597	0.748374915	isogroup00238	ENSG00000130695	ENST00000451429	CEP85
isotig03310	2.11	5.42	2.043	3.021	2383	0.731616818	isogroup00238	ENSG00000130695	ENST00000476272	CEP85
isotig03311	2.015	4.282	2.11	2.745	2335	0.584598332	isogroup00238	ENSG00000130695	ENST00000476272	CEP85
isotig03313	2.001	4.121	2.028	2.653	1906	0.575204779	isogroup00238	ENSG00000130695	ENST00000476272	CEP85
isotig03314	2.832	2.77	1.972	2.597	543	0.102323965	isogroup00238	ENSG00000130695	ENST00000476272	CEP85
isotig03315	4.445	18.607	7.913	12.286	5950	0.895693996	isogroup00239	ENSG00000107372	ENST00000376962	ZFAND5
isotig03316	4.475	18.722	7.982	12.366	5868	0.895834899	isogroup00239	ENSG00000107372	ENST00000376962	ZFAND5
isotig03317	4.521	18.925	8.074	12.52	5799	0.896360938	isogroup00239	ENSG00000107372	ENST00000376962	ZFAND5
isotig03319	4.554	19.047	8.146	12.605	5717	0.896501841	isogroup00239	ENSG00000107372	ENST00000376962	ZFAND5
isotig03321	4.765	20.705	8.777	13.629	5518	0.905162696	isogroup00239	ENSG00000107372	ENST00000376962	ZFAND5
isotig03322	4.857	21.108	8.975	13.919	5367	0.90604569	isogroup00239	ENSG00000107372	ENST00000376962	ZFAND5
isotig03324	19.349	64.518	28.617	66.276	3631	0.952590742	isogroup00240	ENSG00000135821	ENST00000417584	GLUL
isotig03326	21.154	70.657	31.222	72.484	3451	0.953821297	isogroup00240	ENSG00000135821	ENST00000417584	GLUL
isotig03328	23.16	78.647	34.144	80.415	3079	0.957033892	isogroup00240	ENSG00000135821	ENST00000417584	GLUL
isotig03330	25.546	86.833	37.587	88.684	2899	0.958161118	isogroup00240	ENSG00000135821	ENST00000417584	GLUL
isotig03332	47.124	34.443	37.326	40.918	6122	0.134478094	isogroup00241	ENSG00000130779	ENST00000540338	CLIP1
isotig03335	8.821	6.392	5.281	7.15	2106	0.035300969	isogroup00241	ENSG00000130779	ENST00000540338	CLIP1
isotig03336	9.316	6.746	5.573	7.549	1994	0.035761253	isogroup00241	ENSG00000130779	ENST00000540338	CLIP1
isotig03342	3.623	9.416	7.486	3.949	1218	0.540993462	isogroup00242	ENSG00000072080	ENST00000373368	SPP2
isotig03343	4.014	9.707	8.152	4.123	1123	0.470908169	isogroup00242	ENSG00000072080	ENST00000373368	SPP2
isotig03344	4.868	11.752	9.88	4.991	926	0.483241903	isogroup00242	ENSG00000072080	ENST00000373368	SPP2
isotig03347	2.092	4.963	4.068	1.744	618	0.369936875	isogroup00242	ENSG00000072080	ENST00000373368	SPP2

isotig03349	1.673	3.955	3.398	1.532	532	0.32766589	isogroup00242	ENSG00000072080	ENST00000425558	SPP2
isotig03350	3.071	7.24	5.957	2.542	421	0.403199444	isogroup00242	ENSG00000072080	ENST00000373368	SPP2
isotig03351	9.317	3.669	13.434	7.684	3729	0.833640189	isogroup00243	ENSG00000111275	ENST00000261733	ALDH2
isotig03352	10.408	4.12	15.117	8.728	3597	0.837735778	isogroup00243	ENSG00000111275	ENST00000261733	ALDH2
isotig03353	9.982	3.918	14.415	8.312	3561	0.837670023	isogroup00243	ENSG00000111275	ENST00000261733	ALDH2
isotig03354	10.719	4.313	16.118	9.278	3160	0.84080747	isogroup00243	ENSG00000111275	ENST00000261733	ALDH2
isotig03355	12.076	4.877	18.234	10.588	3028	0.844743368	isogroup00243	ENSG00000111275	ENST00000261733	ALDH2
isotig03356	11.589	4.646	17.436	10.115	2992	0.84509093	isogroup00243	ENSG00000111275	ENST00000261733	ALDH2
isotig03357	5.501	2.146	8.556	4.479	1531	0.81605546	isogroup00243	ENSG00000111275	ENST00000552234	ALDH2
isotig03358	4.475	1.806	5.348	3.234	953	0.733373412	isogroup00243	ENSG00000111275	ENST00000553044	ALDH2
isotig03362	1.781	1.38	1.445	1.057	4048	0.231288044	isogroup00244	ENSG00000101928	ENST00000370783	MOSPD1
isotig03366	2.899	1.756	2.493	1.571	1498	0.548517697	isogroup00244	ENSG00000186166	ENST00000334418	CCDC84
isotig03367	4.308	2.355	3.326	1.927	1427	0.652438566	isogroup00244	ENSG00000186166	ENST00000532132	CCDC84
isotig03369	3.177	1.895	2.571	1.298	857	0.602239423	isogroup00244	ENSG00000186166	ENST00000532132	CCDC84
isotig03372	4.222	2.492	2.863	2.376	4197	0.471960246	isogroup00245	ENSG00000070413	ENST00000263196	DGCR2
isotig03373	4.167	2.342	2.702	2.091	3339	0.510304727	isogroup00245	ENSG00000095261	ENST00000210313	PSMD5
isotig03374	3.082	2.316	2.496	2.55	2251	0.19021004	isogroup00245	ENSG00000070413	ENST00000263196	DGCR2
isotig03375	3.106	2.22	2.418	2.504	1568	0.217178928	isogroup00245	ENSG00000070413	ENST00000263196	DGCR2
isotig03376	3.623	1.655	2.164	1.991	1253	0.511366198	isogroup00245	ENSG00000155542	ENST00000285947	C5orf35
isotig03377	2.246	1.849	1.884	1.974	1393	0.076266251	isogroup00245	ENSG00000070413	ENST00000537045	DGCR2
isotig03378	3.526	1.31	1.838	1.766	1195	0.552895093	isogroup00245	ENSG00000155542	ENST00000285947	C5orf35
isotig03379	2.994	1.245	1.673	1.483	1146	0.485083039	isogroup00245	ENSG00000070413	ENST00000545799	DGCR2
isotig03381	1.496	1.189	1.123	1.32	710	0.016354174	isogroup00245	ENSG00000070413	ENST00000537045	DGCR2
isotig03382	304.314	263.698	254.727	190.614	513	0.470729691	isogroup00246	ENSG00000116251	ENST00000234875	RPL22
isotig03386	268.524	260.282	215.564	168.134	433	0.279505148	isogroup00247	ENSG00000173915	ENST00000369815	USMG5
isotig03389	7.782	4.235	5.828	9.053	6711	0.046836252	isogroup00249	ENSG00000197905	ENST00000359864	TEAD4
isotig03390	7.634	4.196	5.686	8.889	6677	0.048837078	isogroup00249	ENSG00000197905	ENST00000359864	TEAD4
isotig03392	5.211	2.31	4.359	5.953	5521	0.153819418	isogroup00249	ENSG00000007866	ENST00000402886	TEAD3
isotig03393	5.015	2.25	4.177	5.734	5487	0.149658075	isogroup00249	ENSG00000007866	ENST00000402886	TEAD3
isotig03395	8.081	9.49	4.834	6.197	2372	0.32137221	isogroup00249	ENSG00000074219	ENST00000377214	TEAD2
isotig03396	1.872	1.655	2.242	3.489	624	0.063566168	isogroup00249	ENSG00000167978	ENST00000426305	SRRM2
isotig03397	1.691	2.762	2.995	6.91	2822	0.68563538	isogroup00250	ENSG00000182326	ENST00000406697	C1S
isotig03398	1.739	2.825	3.101	7.071	2756	0.684977831	isogroup00250	ENSG00000182326	ENST00000406697	C1S
isotig03399	2.198	2.227	3.088	3.881	2318	0.135605321	isogroup00250	ENSG00000159714	ENST00000348579	ZDHHC1
isotig03400	2.1	2.128	2.956	3.783	2261	0.132946945	isogroup00250	ENSG00000182326	ENST00000406697	C1S
isotig03401	3.595	3.861	5.21	4.665	1988	0.089520553	isogroup00250	ENSG00000159714	ENST00000348579	ZDHHC1
isotig03402	3.521	3.793	5.118	4.575	1931	0.099505899	isogroup00250	ENSG00000159714	ENST00000348579	ZDHHC1
isotig03403	3.241	3.237	4.502	4.032	1792	0.119354475	isogroup00250	ENSG00000159714	ENST00000348579	ZDHHC1
isotig03404	3.148	3.141	4.376	3.911	1735	0.131678816	isogroup00250	ENSG00000159714	ENST00000348579	ZDHHC1
isotig03405	1.117	1.207	1.539	1.571	401	0.029392425	isogroup00250	ENSG00000206427	ENST00000552505	PRRC2A
isotig03411	2.321	2.215	4.576	16.137	1963	0.715901405	isogroup00251	ENSG00000128335	ENST00000451256	APOL2
isotig03413	2.551	2.939	4.403	11.676	1927	0.6567596	isogroup00251	ENSG00000128284	ENST00000349314	APOL3
isotig03414	24.532	22.048	18.347	17.811	2106	0.176448486	isogroup00252	ENSG00000122477	ENST00000370137	LRRC39
isotig03415	23.609	20.811	17.46	16.519	1902	0.205145788	isogroup00252	ENSG00000122477	ENST00000370137	LRRC39
isotig03416	12.779	9.597	25.355	12.944	6848	0.790448636	isogroup00253	ENSG00000138162	ENST00000369005	TACC2
isotig03417	11.251	8.563	22.733	11.141	6415	0.789706545	isogroup00253	ENSG00000138162	ENST00000369005	TACC2
isotig03418	14.123	10.701	28.942	14.892	5968	0.79032652	isogroup00253	ENSG00000138162	ENST00000369005	TACC2
isotig03419	12.457	9.589	26.184	12.954	5535	0.791021643	isogroup00253	ENSG00000138162	ENST00000369005	TACC2
isotig03708	2.183	3.408	2.371	1.906	701	0.077975877	isogroup00271	ENSG00000113240	ENST00000522556	CLK4
isotig03709	4.81	4.153	2.098	2.553	732	0.247510709	isogroup00271	ENSG00000113240	ENST00000521621	CLK4
isotig03712	2.315	1.716	3.332	2.31	2839	0.421573232	isogroup00272	ENSG00000162777	ENST00000357640	DENND2D
isotig03713	1.802	1.25	2.604	1.788	2712	0.396746073	isogroup00272	ENSG00000162777	ENST00000369752	DENND2D
isotig03714	2.078	1.319	2.844	1.865	2667	0.458875028	isogroup00272	ENSG00000162777	ENST00000357640	DENND2D
isotig03715	2.039	1.31	2.882	1.907	2593	0.455033065	isogroup00272	ENSG00000162777	ENST00000357640	DENND2D
isotig03716	2.015	1.23	2.703	1.714	2546	0.469076426	isogroup00272	ENSG00000162777	ENST00000369752	DENND2D
isotig03717	2.593	1.942	3.774	2.648	2520	0.43323063	isogroup00272	ENSG00000162777	ENST00000357640	DENND2D
isotig03718	1.973	1.218	2.739	1.753	2472	0.465948373	isogroup00272	ENSG00000162777	ENST00000369752	DENND2D
isotig03720	2.568	1.951	3.843	2.715	2446	0.428383558	isogroup00272	ENSG00000162777	ENST00000357640	DENND2D
isotig03721	2.026	1.426	2.972	2.074	2393	0.410009769	isogroup00272	ENSG00000162777	ENST00000369752	DENND2D
isotig03722	1.981	1.42	3.019	2.126	2319	0.405087548	isogroup00272	ENSG00000162777	ENST00000369752	DENND2D
isotig03723	2.308	1.504	3.309	2.225	2274	0.472026001	isogroup00272	ENSG00000162777	ENST00000357640	DENND2D
isotig03724	2.247	1.408	3.169	2.066	2153	0.486942962	isogroup00272	ENSG00000162777	ENST00000369752	DENND2D
isotig03726	2.935	2.254	4.443	3.176	2127	0.435729315	isogroup00272	ENSG00000162777	ENST00000357640	DENND2D
isotig03728	2.278	1.656	3.526	2.522	2000	0.417609153	isogroup00272	ENSG00000162777	ENST00000369752	DENND2D
isotig03729	1.803	1.299	2.497	1.6	1741	0.40718231	isogroup00272	ENSG00000162777	ENST00000369752	DENND2D
isotig03732	2.219	1.75	3.041	2.229	1266	0.360834523	isogroup00272	ENSG00000162777	ENST00000357640	DENND2D
isotig03734	16.261	15.069	17.715	16.647	2771	0.143552266	isogroup00273	ENSG00000185482	ENST00000332782	STAC3
isotig03736	17.185	15.86	18.691	17.523	2692	0.14472646	isogroup00273	ENSG00000185482	ENST00000332782	STAC3
isotig03738	11.425	10.912	12.582	11.878	2530	0.082888705	isogroup00273	ENSG00000185482	ENST00000332782	STAC3
isotig03740	17.679	16.794	19.571	18.532	2499	0.11500526	isogroup00273	ENSG00000185482	ENST00000332782	STAC3
isotig03742	12.284	11.646	13.489	12.686	2451	0.097927782	isogroup00273	ENSG00000185482	ENST00000332782	STAC3
isotig03744	18.753	17.73	20.718	19.568	2420	0.116029158	isogroup00273	ENSG00000185482	ENST00000332782	STAC3
isotig03746	12.411	12.32	14.018	13.389	2258	0.0383163	isogroup00273	ENSG00000185482	ENST00000332782	STAC3
isotig03748	13.414	13.197	15.091	14.353	2179	0.053580822	isogroup00273	ENSG00000185482	ENST00000332782	STAC3
isotig03750	6.663	6.061	5.741	5.832	1882	0.073692418	isogroup00273	ENSG00000185482	ENST00000554578	STAC3
isotig03752	7.242	7.216	6.6	6.929	1610	0.048282859	isogroup00273	ENSG00000185482	ENST00000553489	STAC3
isotig03754	20.691	18.788	22.356	21.057	971	0.176758473	isogroup00273	ENSG00000185482	ENST00000554578	STAC3
isotig03755	23.872	21.502	25.715	24.091	892	0.192605396	isogroup00273	ENSG00000185482	ENST00000332782	STAC3
isotig03756	5.395	5.607	6.1	5.982	730	0.059639663	isogroup00273	ENSG00000185482	ENST00000554578	STAC3
isotig03757	7.897	7.727	8.728	8.31	651	0.048837078	isogroup00273	ENSG00000185482	ENST00000332782	STAC3
isotig03758	1.924	1.621	1.891	1.329	2215	0.26946344	isogroup00274	ENSG00000126226	ENST00000375479	PCID2
isotig03764	2.085	1.795	2.036	1.414	1845	0.282520478	isogroup00274	ENSG00000126226	ENST00000375479	PCID2

isotig03768	2.315	1.957	2.284	1.546	1634	0.316515744	isogroup00274	ENSG00000126226	ENST00000375479	PCID2
isotig03770	1.878	1.13	1.752	1.004	1655	0.394538589	isogroup00274	ENSG00000126226	ENST00000375457	PCID2
isotig03776	3.994	1.458	2.744	2.157	1023	0.656703239	isogroup00274	ENSG00000126226	ENST00000246505	PCID2
isotig03777	2.724	2.007	2.41	1.72	997	0.389776058	isogroup00274	ENSG00000126226	ENST00000375479	PCID2
isotig03779	1.916	1.319	1.884	1.673	3485	0.259271436	isogroup00275	ENSG00000115657	ENST00000439002	ABCB6
isotig03780	1.989	1.422	1.973	1.789	3361	0.2416961	isogroup00275	ENSG00000115657	ENST00000439002	ABCB6
isotig03781	2.01	1.394	1.975	1.77	3333	0.252376569	isogroup00275	ENSG00000115657	ENST00000265316	ABCB6
isotig03782	2.076	1.479	2.024	1.844	3241	0.256472158	isogroup00275	ENSG00000115657	ENST00000439002	ABCB6
isotig03783	2.09	1.505	2.072	1.896	3209	0.235092433	isogroup00275	ENSG00000115657	ENST00000265316	ABCB6
isotig03784	2.16	1.597	2.126	1.977	3117	0.237591117	isogroup00275	ENSG00000115657	ENST00000439002	ABCB6
isotig03785	2.185	1.568	2.129	1.958	3089	0.24857218	isogroup00275	ENSG00000115657	ENST00000265316	ABCB6
isotig03786	2.279	1.696	2.24	2.102	2965	0.229437514	isogroup00275	ENSG00000115657	ENST00000265316	ABCB6
isotig03787	2.304	1.434	2.176	1.74	2389	0.360609078	isogroup00275	ENSG00000115657	ENST00000439002	ABCB6
isotig03788	2.433	1.593	2.324	1.917	2265	0.343174645	isogroup00275	ENSG00000115657	ENST00000439002	ABCB6
isotig03789	2.589	1.688	2.421	2.008	2145	0.361229052	isogroup00275	ENSG00000115657	ENST00000439002	ABCB6
isotig03790	2.751	1.883	2.602	2.222	2021	0.34310889	isogroup00275	ENSG00000115657	ENST00000439002	ABCB6
isotig03792	1.59	1.109	1.457	1.47	1535	0.166726159	isogroup00275	ENSG00000115657	ENST00000265316	ABCB6
isotig03799	87.599	64.139	56.406	52.325	3560	0.452393477	isogroup00276	ENSG00000198755	ENST00000322203	RPL10A
isotig03801	88.031	64.488	56.738	52.572	3531	0.452449838	isogroup00276	ENSG00000198755	ENST00000322203	RPL10A
isotig03803	7.096	7.163	6.111	5.89	4185	0.089144811	isogroup00276	ENSG00000138814	ENST00000394854	PPP3CA
isotig03804	6.902	7.062	6.041	5.776	4156	0.090431728	isogroup00276	ENSG00000138814	ENST00000394854	PPP3CA
isotig03818	1.242	1.875	1.741	1.809	2745	0.227962726	isogroup00278	ENSG00000136718	ENST00000428740	IMP4
isotig03819	1.46	2.207	2.019	2.126	2658	0.263780341	isogroup00278	ENSG00000136718	ENST00000259239	IMP4
isotig03820	1.376	2.135	1.911	2.025	2662	0.270543699	isogroup00278	ENSG00000136718	ENST00000452955	IMP4
isotig03821	1.607	2.487	2.204	2.359	2575	0.302791764	isogroup00278	ENSG00000136718	ENST00000452955	IMP4
isotig03822	1.152	1.69	1.562	1.644	2422	0.195780416	isogroup00278	ENSG00000136718	ENST00000428740	IMP4
isotig03823	1.303	1.979	1.749	1.883	2339	0.24801796	isogroup00278	ENSG00000136718	ENST00000428740	IMP4
isotig03824	1.63	2.68	2.434	2.65	2025	0.353779965	isogroup00278	ENSG00000136718	ENST00000259239	IMP4
isotig03825	1.948	3.171	2.847	3.123	1938	0.386525889	isogroup00278	ENSG00000136718	ENST00000259239	IMP4
isotig03826	1.832	3.071	2.697	2.982	1942	0.395543699	isogroup00278	ENSG00000136718	ENST00000259239	IMP4
isotig03827	2.174	3.602	3.141	3.491	1855	0.423442549	isogroup00278	ENSG00000136718	ENST00000259239	IMP4
isotig03828	1.577	2.568	2.312	2.575	1702	0.348115653	isogroup00278	ENSG00000136718	ENST00000409935	IMP4
isotig03829	1.817	3.031	2.62	2.968	1619	0.397976629	isogroup00278	ENSG00000136718	ENST00000409935	IMP4
isotig03830	30.023	23.568	32.358	22.918	1300	0.593343729	isogroup00279	ENSG00000256646	ENST00000442788	RP11-111K18.1.1
isotig03834	20.497	16.186	23.468	15.851	1095	0.609331555	isogroup00279	ENSG00000106588	ENST00000445517	PSMA2
isotig03838	39.374	30.081	41.121	29.763	481	0.572546404	isogroup00279	ENSG00000256646	ENST00000442788	RP11-111K18.1.1
isotig03839	3.941	3.884	4.076	3.616	3212	0.099505899	isogroup00280	ENSG00000197912	ENST00000268704	SPG7
isotig03840	4.766	4.895	5.053	4.465	3197	0.079262794	isogroup00280	ENSG00000197912	ENST00000268704	SPG7
isotig03841	4.738	4.734	4.844	4.334	2652	0.092404374	isogroup00280	ENSG00000197912	ENST00000268704	SPG7
isotig03842	5.742	5.965	6.033	5.368	2637	0.061292929	isogroup00280	ENSG00000197912	ENST00000268704	SPG7
isotig03843	4.569	4.702	4.571	4.174	2047	0.053402345	isogroup00280	ENSG00000197912	ENST00000268704	SPG7
isotig03845	2.943	2.996	3.255	2.846	1463	0.06991621	isogroup00280	ENSG00000197912	ENST00000268704	SPG7
isotig03846	1.78	1.403	1.584	1.504	918	0.128945292	isogroup00280	ENSG00000197912	ENST00000268704	SPG7
isotig03847	4.663	4.944	5.002	4.478	903	0.0314684	isogroup00280	ENSG00000197912	ENST00000268704	SPG7
isotig03848	2.754	2.193	1.749	2.62	887	0.091699857	isogroup00280	ENSG00000222033	ENST00000409786	AC007405.2.1
isotig03849	5.261	1.478	2.759	1.378	582	0.792590366	isogroup00280	ENSG00000080573	ENST00000264828	COL5A3
isotig03855	135.075	90.229	116.079	84.545	1875	0.681765236	isogroup00281	ENSG00000154473	ENST00000368858	BUB3
isotig03856	129.967	87.091	111.673	81.445	1883	0.674109491	isogroup00281	ENSG00000118181	ENST00000527673	RPS25
isotig03859	11.924	8.68	9.976	7.738	1205	0.545342677	isogroup00281	ENSG00000154473	ENST00000368865	BUB3
isotig03860	2.338	1.797	3.364	2.482	4496	0.405453896	isogroup00282	ENSG00000095637	ENST00000371227	SORBS1
isotig03861	2.342	1.818	3.398	2.522	4433	0.403227625	isogroup00282	ENSG00000095637	ENST00000371227	SORBS1
isotig03862	2.33	1.777	3.366	2.477	4364	0.403227625	isogroup00282	ENSG00000095637	ENST00000371247	SORBS1
isotig03863	2.334	1.799	3.401	2.518	4301	0.400588036	isogroup00282	ENSG00000095637	ENST00000371227	SORBS1
isotig03864	2.254	1.807	3.262	2.394	3686	0.395139776	isogroup00282	ENSG00000095637	ENST00000371227	SORBS1
isotig03865	2.241	1.783	3.261	2.385	3554	0.392396859	isogroup00282	ENSG00000095637	ENST00000371227	SORBS1
isotig03866	2.194	1.803	3.124	2.229	3533	0.373374915	isogroup00282	ENSG00000095637	ENST00000393949	SORBS1
isotig03867	2.197	1.83	3.163	2.275	3470	0.366649132	isogroup00282	ENSG00000095637	ENST00000393949	SORBS1
isotig03868	2.037	1.818	2.915	2.034	2723	0.343512813	isogroup00282	ENSG00000095637	ENST00000393949	SORBS1
isotig03869	3.37	3.086	3.088	2.318	4595	0.268580446	isogroup00283	ENSG00000177156	ENST00000319006	TALDO1
isotig03870	3.608	3.387	3.329	2.521	4297	0.258980236	isogroup00283	ENSG00000177156	ENST00000319006	TALDO1
isotig03871	4.442	4.18	3.975	3.257	3779	0.22826332	isogroup00283	ENSG00000177156	ENST00000319006	TALDO1
isotig03872	4.828	4.645	4.349	3.588	3481	0.211833997	isogroup00283	ENSG00000177156	ENST00000319006	TALDO1
isotig03873	4.825	4.748	4.421	3.546	2397	0.20023296	isogroup00283	ENSG00000177156	ENST00000319006	TALDO1
isotig03874	5.52	5.601	5.103	4.134	2099	0.171638987	isogroup00283	ENSG00000177156	ENST00000319006	TALDO1
isotig03875	8.14	8.223	7.231	6.423	1581	0.130194634	isogroup00283	ENSG00000177156	ENST00000319006	TALDO1
isotig03876	10.046	10.424	9	8.055	1283	0.095062749	isogroup00283	ENSG00000177156	ENST00000319006	TALDO1
isotig03877	3.065	2.432	3.557	3.409	2576	0.245246863	isogroup00284	ENSG00000184343	ENST00000393786	SRPK3
isotig03878	3.211	2.614	3.763	3.68	2507	0.229841437	isogroup00284	ENSG00000184343	ENST00000393786	SRPK3
isotig03879	3.286	2.639	3.776	3.736	2496	0.231926805	isogroup00284	ENSG00000184343	ENST00000393786	SRPK3
isotig03880	3.443	2.833	3.996	4.025	2427	0.208076576	isogroup00284	ENSG00000184343	ENST00000393786	SRPK3
isotig03881	3.466	2.892	3.999	4.067	2203	0.174457053	isogroup00284	ENSG00000184343	ENST00000393786	SRPK3
isotig03882	3.651	3.121	4.256	4.407	2134	0.153725483	isogroup00284	ENSG00000184343	ENST00000393786	SRPK3
isotig03883	3.74	3.154	4.274	4.476	2123	0.155266025	isogroup00284	ENSG00000184343	ENST00000393786	SRPK3
isotig03884	3.942	3.4	4.55	4.843	2054	0.135426843	isogroup00284	ENSG00000184343	ENST00000393786	SRPK3
isotig03885	40.094	80.555	125.415	97.429	1154	0.184818141	isogroup00285	ENSG00000134184	ENST00000309851	GSTM1
isotig03887	36.332	73.358	109.942	87.451	1124	0.230207785	isogroup00285	ENSG00000134184	ENST00000309851	GSTM1
isotig03891	22.29	40.852	67.666	49.153	1148	0.040223191	isogroup00285	ENSG00000134184	ENST00000309851	GSTM1
contig07020	17.332	33.139	11.113	13.471	560	0.71025588	isogroup00286	ENSG00000139209	ENST00000447411	SLC38A4
contig07024	7.063	15.124	3.794	6.112	1066	0.811302322	isogroup00286	ENSG00000139209	ENST00000447411	SLC38A4
isotig03895	12.517	21.051	10.917	9.254	3266	0.42233411	isogroup00286	ENSG00000139209	ENST00000447411	SLC38A4
isotig03901	23.271	13.351	24.432	14.509	844	0.740756745	isogroup00287	ENSG00000163156	ENST00000368905	SCNM1
isotig03902	20.69	11.369	22.394	12.779	833	0.763583077	isogroup00287	ENSG00000163156	ENST00000368905	SCNM1

isotig03903	16.232	9.83	15.639	10.405	518	0.646915157	isogroup00287	ENSG00000163156	ENST00000368905	SCNM1
isotig03904	11.836	6.495	12.101	7.473	507	0.691806944	isogroup00287	ENSG00000163156	ENST00000368905	SCNM1
isotig03905	5.404	8.961	5.747	8.782	4608	0.6864808	isogroup00288	ENSG00000198492	ENST00000542507	YTHDF2
isotig03909	5.576	9.236	5.869	8.926	4476	0.683409108	isogroup00288	ENSG00000198492	ENST00000542507	YTHDF2
isotig03911	3.071	12.008	4.577	11.242	1917	0.917956715	isogroup00288	ENSG00000173868	ENST00000514112	PHOSPHO1
isotig03913	7.17	5.998	6.712	4.855	3784	0.413241151	isogroup00289	ENSG00000154310	ENST00000475336	TNIK
isotig03914	12.574	13.876	14.895	9.583	2202	0.26930375	isogroup00289	ENSG00000141503	ENST00000355280	MINK1
isotig03916	2.761	2.573	2.486	1.701	1818	0.23384309	isogroup00289	ENSG00000141503	ENST00000355280	MINK1
isotig03917	1.765	1.38	1.335	1.141	1679	0.178731119	isogroup00289	ENSG00000141503	ENST00000355280	MINK1
isotig03918	2.013	1.594	1.648	1.173	1442	0.234200045	isogroup00289	ENSG00000141503	ENST00000355280	MINK1
isotig03919	22.524	26.713	29.175	17.588	1087	0.253005937	isogroup00289	ENSG00000164687	ENST00000297258	FABP5
isotig03921	3.135	2.036	2.396	1.663	4340	0.465084166	isogroup00290	ENSG00000127980	ENST00000248633	PEX1
isotig03922	3.102	2.047	2.303	1.739	3601	0.433822424	isogroup00290	ENSG00000127980	ENST00000248633	PEX1
isotig03923	3.022	2.032	2.244	1.739	3425	0.416951604	isogroup00290	ENSG00000127980	ENST00000248633	PEX1
isotig03924	2.718	1.805	2.247	1.21	1505	0.480893515	isogroup00290	ENSG00000127980	ENST00000428214	PEX1
isotig03925	4.237	2.239	3.384	1.885	1326	0.633548133	isogroup00290	ENSG00000127980	ENST00000428214	PEX1
isotig03926	2.164	1.632	1.666	1.131	766	0.340272037	isogroup00290	ENSG00000127980	ENST00000428214	PEX1
isotig03928	3.372	3.356	4.102	3.595	3221	0.182713985	isogroup00291	ENSG00000126214	ENST00000557450	KLC1
isotig03929	3.46	3.492	4.26	3.743	3145	0.174457053	isogroup00291	ENSG00000126214	ENST00000334553	KLC1
isotig03930	5.369	6.603	5.882	5.489	2914	0.188772826	isogroup00291	ENSG00000126214	ENST00000554228	KLC1
isotig03931	5.61	6.873	6.151	5.738	2887	0.177848125	isogroup00291	ENSG00000126214	ENST00000380038	KLC1
isotig03934	8.764	11.232	9.363	8.342	2604	0.233523709	isogroup00291	ENSG00000126214	ENST00000452929	KLC1
isotig03937	6.693	16.249	14.28	15.262	4977	0.666294056	isogroup00292	ENSG00000072422	ENST00000357917	RHOBTB1
isotig03938	4.333	12.979	9.618	11.051	3489	0.761563463	isogroup00292	ENSG00000072422	ENST00000357917	RHOBTB1
isotig03939	4.424	12.706	9.756	11.02	3422	0.742729391	isogroup00292	ENSG00000072422	ENST00000357917	RHOBTB1
isotig03940	7.675	16.002	15.436	15.69	2528	0.554351093	isogroup00292	ENSG00000072422	ENST00000357917	RHOBTB1
isotig03942	5.845	4.15	9.123	4.251	3061	0.704056136	isogroup00293	ENSG00000090905	ENST00000395799	TNRC6A
isotig03943	8.567	5.658	14.264	6.184	1838	0.80135455	isogroup00293	ENSG00000084072	ENST00000324379	PPIE
isotig03944	3.819	5.003	5.401	4.804	1843	0.103761178	isogroup00293	ENSG00000084072	ENST00000324379	PPIE
isotig03945	4.26	5.229	5.682	4.516	1807	0.003503795	isogroup00293	ENSG00000084072	ENST00000324379	PPIE
isotig03946	2.317	2.135	1.602	1.546	1810	0.104597204	isogroup00293	ENSG00000090905	ENST00000395799	TNRC6A
isotig03947	2.467	2.29	1.868	1.099	876	0.239244383	isogroup00293	ENSG00000084072	ENST00000470213	PPIE
isotig03948	2.739	2.237	1.557	1.659	587	0.057310062	isogroup00293	ENSG00000090905	ENST00000395799	TNRC6A
isotig03951	42.109	36.771	36.131	27.882	3654	0.421432329	isogroup00294	ENSG00000104852	ENST00000401730	SNRNP70
isotig03952	298.841	292.835	305.945	275.424	1633	0.186941084	isogroup00294	ENSG00000087460	ENST00000371085	GNAS
isotig03955	21.896	19.883	19.864	16.036	897	0.306041933	isogroup00294	ENSG00000104852	ENST00000221448	SNRNP70
isotig03956	38.611	35.492	38.948	34.505	750	0.329056136	isogroup00294	ENSG00000087460	ENST00000371081	GNAS
isotig03957	15.92	14.234	14.6	10.601	700	0.456629969	isogroup00294	ENSG00000104852	ENST00000544278	SNRNP70
isotig03958	1.862	2.891	8.878	15.092	1125	0.38002555	isogroup00295	ENSG00000231021	ENST00000457451	HLA-DRB4
isotig03959	2.461	4.051	12.548	22.094	985	0.463008191	isogroup00295	ENSG00000231021	ENST00000457451	HLA-DRB4
isotig03961	1.372	1.892	5.078	9.641	1025	0.421075374	isogroup00295	ENSG00000231021	ENST00000457451	HLA-DRB4
isotig03963	1.96	3.024	8.563	16.573	885	0.52147366	isogroup00295	ENSG00000231021	ENST00000457451	HLA-DRB4
contig07162	27.858	31.086	32.355	27.732	1780	0.023681145	isogroup00296	ENSG00000138297	ENST00000260867	TIMM23
isotig03964	194.283	119.526	151.789	122.491	3522	0.669328173	isogroup00296	ENSG00000138293	ENST00000443446	NCOA4
isotig03965	61.02	38.762	55.189	38.705	1660	0.681333133	isogroup00296	ENSG00000074181	ENST00000539383	NOTCH3
isotig03966	79.67	48.485	88.116	85.152	1960	0.51988615	isogroup00297	ENSG00000163754	ENST00000345003	GYG1
isotig03967	85.422	51.985	94.482	91.273	1824	0.520412189	isogroup00297	ENSG00000163754	ENST00000345003	GYG1
isotig03968	38.403	24.181	44.331	40.307	1411	0.523192681	isogroup00297	ENSG00000163754	ENST00000484197	GYG1
isotig03969	42.231	26.597	48.768	44.279	1275	0.524573533	isogroup00297	ENSG00000163754	ENST00000484197	GYG1
isotig03970	8.508	6.988	8.522	8.832	1716	0.148690539	isogroup00297	ENSG00000146574	ENST00000316731	CCZ1B
isotig03983	102.103	114.012	201.895	183.316	493	0.106898625	isogroup00299	ENSG00000104904	ENST00000322297	OAZ1
isotig03985	822.336	581.055	615.604	508.184	934	0.616611558	isogroup00300	ENSG00000142534	ENST00000270625	RPS11
isotig03989	156.084	157.498	227.033	206.654	4522	0.201745322	isogroup00301	ENSG00000172403	ENST00000429713	SYNPO2
isotig03991	77.666	92.923	78.373	60.184	782	0.023775081	isogroup00302	ENSG00000186010	ENST00000252576	NDUFA13
isotig03992	111.652	135.791	114.185	87.475	531	0.008538739	isogroup00302	ENSG00000186010	ENST00000252576	NDUFA13
isotig03993	83.406	103.834	85.528	68.902	379	0.106194108	isogroup00302	ENSG00000186010	ENST00000507754	NDUFA13
isotig03994	355.245	441.066	344.344	297.619	604	0.138348238	isogroup00303	ENSG00000159199	ENST00000393366	ATP5G1
isotig03996	78.156	97.562	74.334	60.549	601	0.184508154	isogroup00303	ENSG00000135390	ENST00000549164	ATP5G2
isotig03997	69.796	34.123	44.577	32.2	619	0.79217705	isogroup00304	ENSG00000241343	ENST00000427805	RPL36A
isotig03999	1.657	1.685	1.876	1.807	2371	0.028237018	isogroup00305	ENSG00000169696	ENST00000306739	ASPSCR1
isotig04000	1.818	1.855	2.082	1.976	2294	0.034718569	isogroup00305	ENSG00000169696	ENST00000306739	ASPSCR1
isotig04001	1.576	1.608	1.779	1.72	2275	0.016354174	isogroup00305	ENSG00000169696	ENST00000306739	ASPSCR1
isotig04002	1.578	1.61	1.828	1.736	2252	0.041209514	isogroup00305	ENSG00000169696	ENST00000306739	ASPSCR1
isotig04003	1.529	1.536	1.742	1.654	2239	0.029392425	isogroup00305	ENSG00000169696	ENST00000306739	ASPSCR1
isotig04004	1.742	1.783	1.99	1.894	2198	0.028237018	isogroup00305	ENSG00000169696	ENST00000306739	ASPSCR1
isotig04005	1.872	1.872	2.227	2.112	2176	0.076266251	isogroup00305	ENSG00000169696	ENST00000306739	ASPSCR1
isotig04006	1.745	1.788	2.042	1.913	2175	0.05275419	isogroup00305	ENSG00000169696	ENST00000306739	ASPSCR1
isotig04007	1.695	1.711	1.955	1.828	2162	0.041209514	isogroup00305	ENSG00000169696	ENST00000306739	ASPSCR1
isotig04008	1.489	1.526	1.723	1.641	2156	0.034718569	isogroup00305	ENSG00000169696	ENST00000306739	ASPSCR1
isotig04009	1.438	1.448	1.633	1.554	2143	0.021652138	isogroup00305	ENSG00000169696	ENST00000306739	ASPSCR1
isotig04010	1.605	1.61	1.893	1.773	2119	0.060513264	isogroup00305	ENSG00000169696	ENST00000306739	ASPSCR1
isotig04011	1.438	1.448	1.683	1.57	2120	0.048414368	isogroup00305	ENSG00000169696	ENST00000306739	ASPSCR1
isotig04012	2.056	2.066	2.465	2.309	2099	0.084617119	isogroup00305	ENSG00000169696	ENST00000306739	ASPSCR1
isotig04013	1.794	1.797	2.138	2.031	2080	0.065266401	isogroup00305	ENSG00000169696	ENST00000306739	ASPSCR1
isotig04014	1.66	1.709	1.943	1.823	2079	0.041209514	isogroup00305	ENSG00000169696	ENST00000306739	ASPSCR1
isotig04015	1.608	1.628	1.851	1.733	2066	0.029392425	isogroup00305	ENSG00000169696	ENST00000306739	ASPSCR1
isotig04016	1.746	1.721	2.103	1.964	2044	0.083029608	isogroup00305	ENSG00000169696	ENST00000306739	ASPSCR1
isotig04017	1.785	1.799	2.124	1.962	2042	0.069775306	isogroup00305	ENSG00000169696	ENST00000306739	ASPSCR1
isotig04018	1.61	1.631	1.906	1.752	2043	0.059301495	isogroup00305	ENSG00000169696	ENST00000306739	ASPSCR1
isotig04019	1.512	1.52	1.784	1.674	2023	0.048414368	isogroup00305	ENSG00000169696	ENST00000306739	ASPSCR1
isotig04020	1.336	1.351	1.564	1.461	2024	0.035733073	isogroup00305	ENSG00000169696	ENST00000306739	ASPSCR1
isotig04021	1.983	1.997	2.382	2.234	2003	0.074039979	isogroup00305	ENSG00000169696	ENST00000306739	ASPSCR1

isotig04022	1.457	1.437	1.743	1.599	1987	0.066496956	isogroup00305	ENSG00000169696	ENST00000306739	ASPSCR1
isotig04023	1.501	1.551	1.676	1.661	1987	0.010060494	isogroup00305	ENSG00000169696	ENST00000306739	ASPSCR1
isotig04024	1.936	1.922	2.351	2.168	1967	0.091699857	isogroup00305	ENSG00000169696	ENST00000306739	ASPSCR1
isotig04025	1.696	1.715	2.022	1.868	1946	0.059301495	isogroup00305	ENSG00000169696	ENST00000306739	ASPSCR1
isotig04026	1.656	1.633	2.001	1.871	1948	0.071428571	isogroup00305	ENSG00000169696	ENST00000306739	ASPSCR1
isotig04027	1.513	1.539	1.793	1.648	1947	0.047521981	isogroup00305	ENSG00000169696	ENST00000306739	ASPSCR1
isotig04028	1.643	1.632	1.983	1.794	1910	0.078182536	isogroup00305	ENSG00000169696	ENST00000306739	ASPSCR1
isotig04029	1.35	1.332	1.619	1.483	1891	0.054895919	isogroup00305	ENSG00000169696	ENST00000306739	ASPSCR1
isotig04030	1.396	1.452	1.549	1.548	1891	0.010060494	isogroup00305	ENSG00000169696	ENST00000306739	ASPSCR1
isotig04031	1.852	1.841	2.257	2.081	1871	0.087435184	isogroup00305	ENSG00000169696	ENST00000306739	ASPSCR1
isotig04032	1.395	1.452	1.604	1.566	1868	0.029392425	isogroup00305	ENSG00000169696	ENST00000306739	ASPSCR1
isotig04033	1.335	1.361	1.5	1.465	1855	0.016354174	isogroup00305	ENSG00000169696	ENST00000306739	ASPSCR1
isotig04034	1.541	1.534	1.867	1.684	1814	0.066496956	isogroup00305	ENSG00000169696	ENST00000306739	ASPSCR1
isotig04035	1.744	1.764	2.081	2.016	1792	0.069775306	isogroup00305	ENSG00000169696	ENST00000306739	ASPSCR1
isotig04036	1.277	1.341	1.465	1.442	1772	0.016354174	isogroup00305	ENSG00000169696	ENST00000306739	ASPSCR1
isotig04037	1.213	1.244	1.354	1.334	1759	0.010060494	isogroup00305	ENSG00000169696	ENST00000306739	ASPSCR1
isotig04038	1.415	1.44	1.667	1.599	1735	0.048414368	isogroup00305	ENSG00000169696	ENST00000306739	ASPSCR1
isotig04039	1.21	1.243	1.411	1.351	1736	0.029805741	isogroup00305	ENSG00000169696	ENST00000306739	ASPSCR1
isotig04040	1.641	1.666	1.962	1.911	1696	0.059301495	isogroup00305	ENSG00000169696	ENST00000306739	ASPSCR1
isotig04041	1.578	1.569	1.916	1.826	1660	0.078182536	isogroup00305	ENSG00000169696	ENST00000306739	ASPSCR1
isotig04042	1.289	1.319	1.52	1.466	1639	0.035733073	isogroup00305	ENSG00000169696	ENST00000306739	ASPSCR1
isotig04043	1.071	1.11	1.248	1.204	1640	0.016560833	isogroup00305	ENSG00000169696	ENST00000306739	ASPSCR1
isotig04044	1.216	1.211	1.463	1.368	1603	0.055318629	isogroup00305	ENSG00000169696	ENST00000306739	ASPSCR1
isotig04045	1.457	1.451	1.777	1.701	1564	0.066496956	isogroup00305	ENSG00000169696	ENST00000306739	ASPSCR1
isotig04194	1.479	1.753	1.94	1.613	3985	0.078182536	isogroup00310	ENSG00000108439	ENST00000544840	PNPO
isotig04196	1.567	1.896	2.088	1.745	3757	0.066496956	isogroup00310	ENSG00000108439	ENST00000544840	PNPO
isotig04198	1.523	1.838	2.037	1.689	3710	0.071428571	isogroup00310	ENSG00000108439	ENST00000225573	PNPO
isotig04200	1.607	1.929	2.103	1.754	3677	0.071428571	isogroup00310	ENSG00000108439	ENST00000534893	PNPO
isotig04202	1.621	1.998	2.203	1.837	3482	0.059301495	isogroup00310	ENSG00000108439	ENST00000225573	PNPO
isotig04204	1.71	2.097	2.274	1.908	3449	0.063566168	isogroup00310	ENSG00000108439	ENST00000534893	PNPO
isotig04206	1.665	2.036	2.222	1.848	3402	0.063566168	isogroup00310	ENSG00000108439	ENST00000534893	PNPO
isotig04208	1.782	2.226	2.417	2.022	3174	0.05275419	isogroup00310	ENSG00000108439	ENST00000225573	PNPO
isotig04212	1.347	1.426	1.542	1.329	2840	0.106053205	isogroup00310	ENSG00000108439	ENST00000544840	PNPO
isotig04214	1.285	1.342	1.466	1.247	2793	0.112431427	isogroup00310	ENSG00000108439	ENST00000225573	PNPO
isotig04216	1.393	1.458	1.546	1.329	2760	0.112036898	isogroup00310	ENSG00000108439	ENST00000534893	PNPO
isotig04218	1.396	1.515	1.641	1.409	2565	0.099327422	isogroup00310	ENSG00000108439	ENST00000225573	PNPO
isotig04220	1.515	1.643	1.729	1.5	2532	0.104832043	isogroup00310	ENSG00000108439	ENST00000534893	PNPO
isotig04222	1.449	1.552	1.648	1.411	2485	0.105508379	isogroup00310	ENSG00000108439	ENST00000534893	PNPO
isotig04224	1.592	1.77	1.864	1.611	2257	0.090788683	isogroup00310	ENSG00000108439	ENST00000225573	PNPO
isotig04250	3.395	4.774	3.148	2.576	2936	0.281036297	isogroup00312	ENSG00000112759	ENST00000313248	SLC29A1
isotig04251	3.535	5.022	3.273	2.709	2846	0.296732923	isogroup00312	ENSG00000112759	ENST00000313248	SLC29A1
isotig04252	3.76	5.349	3.501	2.909	2691	0.311631096	isogroup00312	ENSG00000112759	ENST00000427851	SLC29A1
isotig04253	3.792	5.387	3.525	2.916	2640	0.304867739	isogroup00312	ENSG00000112759	ENST00000313248	SLC29A1
isotig04254	3.926	5.641	3.649	3.066	2601	0.327712858	isogroup00312	ENSG00000112759	ENST00000427851	SLC29A1
isotig04255	3.963	5.685	3.677	3.077	2550	0.328755542	isogroup00312	ENSG00000112759	ENST00000313248	SLC29A1
isotig04256	4.243	6.096	3.959	3.324	2395	0.335443751	isogroup00312	ENSG00000112759	ENST00000313248	SLC29A1
isotig04257	4.449	6.454	4.145	3.518	2305	0.359068535	isogroup00312	ENSG00000112759	ENST00000313248	SLC29A1
isotig04260	1.515	2.085	1.314	1.148	2126	0.17693695	isogroup00312	ENSG00000112759	ENST00000427851	SLC29A1
isotig04261	1.501	2.053	1.291	1.114	2075	0.170981438	isogroup00312	ENSG00000112759	ENST00000313248	SLC29A1
isotig04262	1.628	2.313	1.407	1.271	2036	0.221950853	isogroup00312	ENSG00000112759	ENST00000427851	SLC29A1
isotig04263	1.617	2.285	1.386	1.239	1985	0.21596716	isogroup00312	ENSG00000112759	ENST00000313248	SLC29A1
isotig04266	1.784	2.534	1.561	1.407	1830	0.240503119	isogroup00312	ENSG00000112759	ENST00000313248	SLC29A1
isotig04267	1.93	2.825	1.683	1.564	1740	0.286531525	isogroup00312	ENSG00000112759	ENST00000313248	SLC29A1
isotig04269	1.699	1.977	1.388	1.127	1636	0.042270985	isogroup00312	ENSG00000112759	ENST00000313248	SLC29A1
isotig04270	1.524	2.047	1.32	1.107	1624	0.147779364	isogroup00312	ENSG00000112759	ENST00000313248	SLC29A1
isotig04271	1.858	2.272	1.515	1.288	1546	0.102314571	isogroup00312	ENSG00000112759	ENST00000313248	SLC29A1
isotig04272	1.71	2.293	1.506	1.259	1418	0.167637334	isogroup00312	ENSG00000112759	ENST00000313248	SLC29A1
isotig04273	1.897	2.658	1.662	1.455	1328	0.231588638	isogroup00312	ENSG00000112759	ENST00000313248	SLC29A1
isotig04278	1.465	1.02	1.297	1.435	4073	0.106428947	isogroup00313	ENSG00000196781	ENST00000376499	TLE1
isotig04279	1.47	1.038	1.298	1.449	4043	0.09326858	isogroup00313	ENSG00000196781	ENST00000376499	TLE1
isotig04282	1.539	1.072	1.362	1.508	3872	0.106053205	isogroup00313	ENSG00000196781	ENST00000376499	TLE1
isotig04283	1.546	1.092	1.363	1.523	3842	0.099327422	isogroup00313	ENSG00000196781	ENST00000376499	TLE1
isotig04284	1.438	1.015	1.298	1.419	3771	0.093559781	isogroup00313	ENSG00000196781	ENST00000376499	TLE1
isotig04285	1.444	1.036	1.298	1.434	3741	0.086824604	isogroup00313	ENSG00000196781	ENST00000376499	TLE1
isotig04287	2.584	1.451	2.424	2.741	2297	0.265612084	isogroup00313	ENSG00000196781	ENST00000418319	TLE1
isotig04288	2.609	1.491	2.439	2.783	2267	0.250018787	isogroup00313	ENSG00000196781	ENST00000418319	TLE1
isotig04289	2.458	1.372	2.362	2.645	2196	0.258472984	isogroup00313	ENSG00000196781	ENST00000376463	TLE1
isotig04290	2.483	1.411	2.377	2.688	2166	0.240164951	isogroup00313	ENSG00000196781	ENST00000376463	TLE1
isotig04291	1.595	1.247	1.293	1.31	1764	0.074321786	isogroup00313	ENSG00000106829	ENST00000376544	TLE4
isotig04292	1.796	1.405	1.453	1.475	1563	0.079507026	isogroup00313	ENSG00000106829	ENST00000376544	TLE4
isotig04294	1.324	1.172	1.256	1.051	1069	0.048827685	isogroup00313	ENSG00000196781	ENST00000376499	TLE1
isotig04295	2.024	4.71	2.274	2.048	8100	0.760436237	isogroup00314	ENSG00000115414	ENST00000265313	FN1
isotig04297	1.995	4.646	2.238	2.021	8025	0.758134816	isogroup00314	ENSG00000115414	ENST00000265313	FN1
isotig04299	2.103	4.882	2.367	2.12	7827	0.765480574	isogroup00314	ENSG00000115414	ENST00000356005	FN1
isotig04301	2.073	4.817	2.33	2.092	7752	0.764419103	isogroup00314	ENSG00000115414	ENST00000356005	FN1
isotig04303	1.964	4.585	2.202	1.981	7740	0.755964906	isogroup00314	ENSG00000115414	ENST00000432072	FN1
isotig04305	2.045	4.761	2.296	2.054	7467	0.762577966	isogroup00314	ENSG00000115414	ENST00000357867	FN1
isotig04307	1.389	3.039	1.269	1.313	7369	0.70036447	isogroup00314	ENSG00000115414	ENST00000432072	FN1
isotig04308	1.349	2.951	1.219	1.275	7294	0.694380777	isogroup00314	ENSG00000115414	ENST00000432072	FN1
isotig04309	1.451	3.164	1.332	1.364	7096	0.709983467	isogroup00314	ENSG00000115414	ENST00000356005	FN1
isotig04310	1.411	3.075	1.281	1.325	7021	0.703755542	isogroup00314	ENSG00000115414	ENST00000356005	FN1
isotig04312	1.352	2.938	1.199	1.25	6736	0.694803487	isogroup00314	ENSG00000115414	ENST00000357867	FN1

isotig04353	2.368	1.981	2.265	2.156	4819	0.140236342	isogroup00317	ENSG00000130985	ENST00000377351	UBA1
isotig04354	2.32	1.891	2.216	2.097	4645	0.155763884	isogroup00317	ENSG00000130985	ENST00000377351	UBA1
isotig04355	2.58	2.14	2.466	2.349	4578	0.153913354	isogroup00317	ENSG00000130985	ENST00000377351	UBA1
isotig04356	2.515	2.091	2.409	2.284	4563	0.150381378	isogroup00317	ENSG00000130985	ENST00000377351	UBA1
isotig04357	2.538	2.052	2.423	2.295	4404	0.174006162	isogroup00317	ENSG00000130985	ENST00000377351	UBA1
isotig04358	2.47	2.001	2.364	2.227	4389	0.167487037	isogroup00317	ENSG00000130985	ENST00000377351	UBA1
isotig04359	2.748	2.266	2.63	2.496	4322	0.164330803	isogroup00317	ENSG00000130985	ENST00000377351	UBA1
isotig04360	2.711	2.177	2.592	2.444	4148	0.18420756	isogroup00317	ENSG00000130985	ENST00000377351	UBA1
isotig04361	4.424	3.354	3.3	3.018	3360	0.40805591	isogroup00317	ENSG00000111605	ENST00000435070	CPSF6
isotig04362	3.34	2.789	3.192	3.021	3340	0.173912227	isogroup00317	ENSG00000130985	ENST00000377351	UBA1
isotig04363	3.323	2.701	3.173	2.982	3166	0.196062223	isogroup00317	ENSG00000130985	ENST00000377351	UBA1
isotig04364	3.729	3.087	3.562	3.373	3099	0.192417525	isogroup00317	ENSG00000130985	ENST00000377351	UBA1
isotig04365	3.638	3.019	3.483	3.282	3084	0.182713985	isogroup00317	ENSG00000130985	ENST00000377351	UBA1
isotig04366	3.734	3.01	3.563	3.352	2925	0.214323288	isogroup00317	ENSG00000130985	ENST00000377351	UBA1
isotig04367	3.638	2.938	3.479	3.256	2910	0.210002254	isogroup00317	ENSG00000130985	ENST00000377351	UBA1
isotig04368	4.088	3.363	3.91	3.689	2843	0.19978207	isogroup00317	ENSG00000130985	ENST00000377351	UBA1
isotig04369	4.118	3.297	3.934	3.686	2669	0.22511648	isogroup00317	ENSG00000130985	ENST00000377351	UBA1
isotig04370	11.146	19.818	15.932	20.67	879	0.677265725	isogroup00317	ENSG00000248713	ENST00000511828	RP11-766F14.2.1
isotig04438	1.742	1.108	1.725	1.543	9618	0.212247313	isogroup00322	ENSG00000058272	ENST00000546369	PPP1R12A
isotig04439	1.752	1.117	1.75	1.566	9448	0.212247313	isogroup00322	ENSG00000058272	ENST00000546369	PPP1R12A
isotig04440	1.751	1.091	1.738	1.558	9441	0.218024348	isogroup00322	ENSG00000058272	ENST00000312727	PPP1R12A
isotig04441	3.041	2.361	2.965	2.863	9376	0.135605321	isogroup00322	ENSG00000133030	ENST00000395804	MPRIP
isotig04442	1.761	1.1	1.765	1.581	9271	0.22268355	isogroup00322	ENSG00000058272	ENST00000312727	PPP1R12A
isotig04443	3.062	2.328	2.981	2.862	9314	0.150869843	isogroup00322	ENSG00000133030	ENST00000395811	MPRIP
isotig04444	3.085	2.392	3.019	2.901	9153	0.138291876	isogroup00322	ENSG00000133030	ENST00000395804	MPRIP
isotig04445	3.107	2.358	3.036	2.9	9091	0.158844969	isogroup00322	ENSG00000133030	ENST00000395811	MPRIP
isotig04449	2.988	2.87	2.69	2.707	5009	0.059555121	isogroup00322	ENSG00000133030	ENST00000395804	MPRIP
isotig04451	3.027	2.814	2.716	2.702	4947	0.027992786	isogroup00322	ENSG00000133030	ENST00000395811	MPRIP
isotig04452	3.07	2.953	2.78	2.771	4786	0.059555121	isogroup00322	ENSG00000133030	ENST00000395804	MPRIP
isotig04453	3.112	2.895	2.808	2.767	4724	0.025447133	isogroup00322	ENSG00000133030	ENST00000395811	MPRIP
isotig04454	2.096	1.722	1.807	1.651	7792	0.159821898	isogroup00323	ENSG00000160294	ENST00000397708	MCM3AP
isotig04455	2.166	1.779	1.859	1.709	7583	0.159821898	isogroup00323	ENSG00000160294	ENST00000397708	MCM3AP
isotig04456	2.321	1.979	1.966	1.835	6647	0.139334561	isogroup00323	ENSG00000160294	ENST00000397708	MCM3AP
isotig04457	2.411	2.054	2.032	1.909	6438	0.139334561	isogroup00323	ENSG00000160294	ENST00000397708	MCM3AP
isotig04458	2.02	1.662	1.832	1.592	6028	0.180158939	isogroup00323	ENSG00000160294	ENST00000397708	MCM3AP
isotig04459	2.108	1.734	1.901	1.665	5819	0.183258811	isogroup00323	ENSG00000160294	ENST00000397708	MCM3AP
isotig04460	2.309	1.997	2.054	1.828	4883	0.155763884	isogroup00323	ENSG00000160294	ENST00000397708	MCM3AP
isotig04461	2.432	2.102	2.149	1.93	4674	0.15977493	isogroup00323	ENSG00000160294	ENST00000397708	MCM3AP
isotig04462	1.453	1.026	1.455	1.12	3575	0.237591117	isogroup00323	ENSG00000160294	ENST00000539647	MCM3AP
isotig04463	1.571	1.11	1.55	1.219	3366	0.242738784	isogroup00323	ENSG00000160294	ENST00000397708	MCM3AP
isotig04464	1.767	1.4	1.723	1.373	2430	0.218024348	isogroup00323	ENSG00000160294	ENST00000539647	MCM3AP
isotig04465	1.976	1.563	1.892	1.547	2221	0.227117307	isogroup00323	ENSG00000160294	ENST00000397708	MCM3AP
isotig04466	2.171	1.957	1.581	1.711	2187	0.022976629	isogroup00323	ENSG00000160294	ENST00000397708	MCM3AP
isotig04467	2.925	4.194	3.778	3.258	5123	0.231851657	isogroup00324	ENSG00000186628	ENST00000334574	FSD2
isotig04468	2.568	3.609	3.113	2.854	5047	0.237403246	isogroup00324	ENSG00000186628	ENST00000334574	FSD2
isotig04469	2.774	3.993	3.596	3.062	4893	0.214323288	isogroup00324	ENSG00000186628	ENST00000334574	FSD2
isotig04470	2.605	3.667	3.16	2.895	4978	0.237403246	isogroup00324	ENSG00000186628	ENST00000334574	FSD2
isotig04471	2.398	3.377	2.896	2.634	4817	0.21826858	isogroup00324	ENSG00000186628	ENST00000334574	FSD2
isotig04472	2.488	4.318	3.264	2.793	4673	0.378794995	isogroup00324	ENSG00000186628	ENST00000334574	FSD2
isotig04473	2.433	3.435	2.943	2.675	4748	0.220598181	isogroup00324	ENSG00000186628	ENST00000334574	FSD2
isotig04474	2.09	3.678	2.526	2.341	4597	0.394942511	isogroup00324	ENSG00000186628	ENST00000334574	FSD2
isotig04475	2.123	3.743	2.569	2.379	4528	0.397009093	isogroup00324	ENSG00000186628	ENST00000334574	FSD2
isotig04476	2.62	3.423	3.302	2.706	4392	0.110749981	isogroup00324	ENSG00000230230	ENST00000455000	TRIM26
isotig04477	2.198	2.725	2.517	2.222	4316	0.09882017	isogroup00324	ENSG00000230230	ENST00000455000	TRIM26
isotig04478	2.235	2.779	2.562	2.261	4247	0.09882017	isogroup00324	ENSG00000230230	ENST00000455000	TRIM26
isotig04479	2.738	4.176	3.089	3.024	1600	0.380514015	isogroup00324	ENSG00000186628	ENST00000541889	FSD2
isotig04480	2.166	3.454	2.323	2.282	1370	0.362159014	isogroup00324	ENSG00000186628	ENST00000541889	FSD2
contig07564	1144.138	523.718	1173.904	657.018	4066	0.889090328	isogroup00326	ENSG00000197893	ENST00000369358	NRAP
isotig04496	6.341	8.741	9.164	9.051	4195	0.345166078	isogroup00326	ENSG00000141279	ENST00000530173	NPEPPS
isotig04497	6.413	8.836	9.268	9.153	4148	0.34567333	isogroup00326	ENSG00000141279	ENST00000530173	NPEPPS
isotig04499	55.8	27.18	72.736	32.541	1901	0.906815962	isogroup00326	ENSG00000197893	ENST00000369350	NRAP
isotig04500	57.074	27.831	74.378	33.302	1854	0.904768167	isogroup00326	ENSG00000197893	ENST00000369350	NRAP
isotig04502	79.265	41.946	110.187	51.005	1323	0.884656572	isogroup00326	ENSG00000197893	ENST00000369350	NRAP
isotig04503	81.981	43.437	113.951	52.79	1276	0.884957165	isogroup00326	ENSG00000197893	ENST00000369350	NRAP
isotig04505	184.509	99.068	261.228	117.105	381	0.887277373	isogroup00326	ENSG00000197893	ENST00000369350	NRAP
isotig04506	209.695	112.799	296.865	133.225	334	0.887794018	isogroup00326	ENSG00000197893	ENST00000369350	NRAP
isotig04520	3.106	2.559	1.959	2.023	8071	0.095447885	isogroup00328	ENSG00000143382	ENST00000369038	ADAMTSL4
isotig04521	4.04	3.398	3.601	3.567	8112	0.09609604	isogroup00328	ENSG00000143382	ENST00000369038	ADAMTSL4
isotig04896	2.349	1.313	1.342	1.031	2112	0.385671075	isogroup00371	ENSG00000108821	ENST00000225964	COL1A1
isotig04899	2.535	1.412	1.455	1.112	1988	0.405228451	isogroup00371	ENSG00000172889	ENST00000406555	EGFL7
isotig04901	2.545	1.411	1.425	1.116	1923	0.409634027	isogroup00371	ENSG00000172889	ENST00000406555	EGFL7
isotig04906	2.764	1.528	1.555	1.211	1799	0.431727662	isogroup00371	ENSG00000172889	ENST00000406555	EGFL7
isotig04911	2.875	2.163	1.498	1.03	528	0.338994514	isogroup00371	ENSG00000214655	ENST00000492395	KIAA0913
isotig04935	9.319	8.612	10.869	11.359	4268	0.019651311	isogroup00373	ENSG00000181035	ENST00000318596	SLC25A42
isotig04937	9.489	8.709	11.165	11.601	4098	0.007420906	isogroup00373	ENSG00000181035	ENST00000318596	SLC25A42
isotig04939	14.431	14.299	15.65	18.62	3951	0.2025062	isogroup00373	ENSG00000104369	ENST00000342232	JPH1
isotig04943	14.845	14.66	16.185	19.208	3781	0.202731645	isogroup00373	ENSG00000104369	ENST00000342232	JPH1
isotig04947	6.997	7.289	8.6	9.144	2499	0.161127602	isogroup00373	ENSG00000181035	ENST00000318596	SLC25A42
isotig04949	15.916	17.395	16.926	21.97	2182	0.38317239	isogroup00373	ENSG00000104369	ENST00000342232	JPH1
isotig04953	3.206	2.975	4.252	3.799	1705	0.161428196	isogroup00373	ENSG00000181035	ENST00000318596	SLC25A42
isotig04954	16.362	17.877	16.349	22.741	1388	0.408807395	isogroup00373	ENSG00000104369	ENST00000342232	JPH1
isotig04956	9.974	10.764	9.652	13.768	1201	0.403678515	isogroup00373	ENSG00000104369	ENST00000342232	JPH1

isotig05540	6.35	3.364	5.077	5.517	2799	0.488652589	isogroup00434	ENSG00000179912	ENST00000403821	R3HDM2
isotig05541	6.267	3.33	5	5.448	2766	0.486416923	isogroup00434	ENSG00000179912	ENST00000403821	R3HDM2
isotig05542	5.899	3.141	4.718	5.143	2760	0.47897723	isogroup00434	ENSG00000179912	ENST00000403821	R3HDM2
isotig05543	5.809	3.104	4.635	5.07	2727	0.471349666	isogroup00434	ENSG00000179912	ENST00000403821	R3HDM2
isotig05544	6.266	3.28	4.988	5.425	2703	0.494251146	isogroup00434	ENSG00000179912	ENST00000403821	R3HDM2
isotig05545	6.296	3.278	5.062	5.417	2697	0.495951379	isogroup00434	ENSG00000179912	ENST00000402412	R3HDM2
isotig05546	6.178	3.243	4.906	5.353	2670	0.49211881	isogroup00434	ENSG00000179912	ENST00000403821	R3HDM2
isotig05547	5.797	3.047	4.614	5.037	2664	0.484547607	isogroup00434	ENSG00000179912	ENST00000403821	R3HDM2
isotig05548	6.208	3.242	4.981	5.345	2664	0.493668746	isogroup00434	ENSG00000179912	ENST00000402412	R3HDM2
isotig05549	5.826	3.045	4.688	5.028	2658	0.486257233	isogroup00434	ENSG00000179912	ENST00000402412	R3HDM2
isotig05550	5.702	3.008	4.527	4.959	2631	0.476271887	isogroup00434	ENSG00000179912	ENST00000403821	R3HDM2
isotig05551	5.731	3.005	4.602	4.95	2625	0.482819193	isogroup00434	ENSG00000179912	ENST00000402412	R3HDM2
isotig05552	6.206	3.187	4.968	5.318	2601	0.507486661	isogroup00434	ENSG00000179912	ENST00000403821	R3HDM2
isotig05553	6.114	3.148	4.883	5.242	2568	0.4997088	isogroup00434	ENSG00000179912	ENST00000403821	R3HDM2
isotig05554	5.717	2.944	4.58	4.913	2562	0.491508229	isogroup00434	ENSG00000179912	ENST00000403821	R3HDM2
isotig05555	5.617	2.901	4.488	4.831	2529	0.488887428	isogroup00434	ENSG00000179912	ENST00000403821	R3HDM2
isotig05556	4.249	2.262	3.477	3.523	1380	0.450552341	isogroup00434	ENSG00000179912	ENST00000402412	R3HDM2
isotig05557	4.025	2.164	3.278	3.334	1347	0.433869392	isogroup00434	ENSG00000179912	ENST00000402412	R3HDM2
isotig05558	3.914	2.001	3.17	3.182	1284	0.462576088	isogroup00434	ENSG00000179912	ENST00000403821	R3HDM2
isotig05559	3.663	1.889	2.947	2.969	1251	0.4459495	isogroup00434	ENSG00000179912	ENST00000403821	R3HDM2
contig08908	4.612	6.127	4.332	6.403	2451	0.587491546	isogroup00435	ENSG00000167702	ENST00000301332	KIFC2
isotig05560	6.031	6.791	8.052	8.07	3605	0.144989479	isogroup00435	ENSG00000143164	ENST00000367840	DCAF6
isotig05561	6.094	6.873	8.157	8.17	3563	0.144407079	isogroup00435	ENSG00000143164	ENST00000367840	DCAF6
isotig05562	6.194	6.973	8.274	8.287	3506	0.145524912	isogroup00435	ENSG00000143164	ENST00000367840	DCAF6
isotig05563	6.261	7.059	8.385	8.393	3464	0.144989479	isogroup00435	ENSG00000143164	ENST00000367840	DCAF6
isotig05564	5.864	6.57	7.816	7.744	3434	0.130654918	isogroup00435	ENSG00000143164	ENST00000367843	DCAF6
isotig05565	5.929	6.653	7.923	7.845	3392	0.130194634	isogroup00435	ENSG00000143164	ENST00000367843	DCAF6
isotig05566	6.031	6.754	8.043	7.963	3335	0.131171564	isogroup00435	ENSG00000143164	ENST00000367843	DCAF6
isotig05567	6.1	6.842	8.156	8.07	3293	0.130654918	isogroup00435	ENSG00000143164	ENST00000367843	DCAF6
isotig05568	5.405	6.327	7.827	7.509	2637	0.128071692	isogroup00435	ENSG00000143164	ENST00000432587	DCAF6
isotig05569	5.481	6.432	7.968	7.637	2595	0.116423687	isogroup00435	ENSG00000143164	ENST00000432587	DCAF6
isotig05570	5.606	6.56	8.126	7.788	2538	0.128597731	isogroup00435	ENSG00000143164	ENST00000432587	DCAF6
isotig05571	5.689	6.673	8.277	7.925	2496	0.116893364	isogroup00435	ENSG00000143164	ENST00000432587	DCAF6
isotig05572	5.129	5.987	7.484	7.016	2466	0.102474262	isogroup00435	ENSG00000143164	ENST00000432587	DCAF6
isotig05573	5.207	6.094	7.628	7.144	2424	0.101976403	isogroup00435	ENSG00000143164	ENST00000367843	DCAF6
isotig05574	5.333	6.223	7.789	7.294	2367	0.10313181	isogroup00435	ENSG00000143164	ENST00000432587	DCAF6
isotig05575	5.418	6.338	7.945	7.434	2325	0.102474262	isogroup00435	ENSG00000143164	ENST00000367843	DCAF6
isotig05576	13.041	8.043	6.988	5.606	1865	0.592667393	isogroup00436	ENSG00000135424	ENST00000394230	ITGA7
isotig05578	19.633	9.959	10.79	7.289	1794	0.757524235	isogroup00436	ENSG00000135424	ENST00000557555	ITGA7
isotig05580	11.425	7.003	6.353	4.93	1756	0.59710115	isogroup00436	ENSG00000135424	ENST00000394230	ITGA7
isotig05581	12.989	8.322	7.068	5.752	1752	0.563904336	isogroup00436	ENSG00000135424	ENST00000553804	ITGA7
isotig05584	11.257	7.229	6.394	5.038	1643	0.568695048	isogroup00436	ENSG00000135424	ENST00000553804	ITGA7
isotig05586	31.526	10.873	16.484	8.939	1595	0.911193357	isogroup00436	ENSG00000135424	ENST00000557555	ITGA7
isotig05588	9.765	5.731	5.523	4.081	1320	0.616752461	isogroup00436	ENSG00000135424	ENST00000557555	ITGA7
isotig05590	9.382	5.921	5.503	4.149	1207	0.56809386	isogroup00436	ENSG00000135424	ENST00000553804	ITGA7
isotig05592	13.203	8.007	6.196	5.428	897	0.576548057	isogroup00436	ENSG00000135424	ENST00000553804	ITGA7
isotig05593	9.622	5.684	4.671	3.897	788	0.583039002	isogroup00436	ENSG00000135424	ENST00000553804	ITGA7
isotig05610	3.209	2.346	4.102	3.635	5157	0.335293455	isogroup00438	ENSG00000100239	ENST00000216061	PPP6R2
isotig05612	3.267	2.389	4.171	3.694	5097	0.337115804	isogroup00438	ENSG00000100239	ENST00000216061	PPP6R2
isotig05613	3.052	2.268	3.991	3.502	5076	0.331601789	isogroup00438	ENSG00000100239	ENST00000359139	PPP6R2
isotig05616	3.108	2.311	4.059	3.56	5016	0.333442925	isogroup00438	ENSG00000100239	ENST00000359139	PPP6R2
isotig05618	2.979	2.216	3.558	3.343	3845	0.270957015	isogroup00438	ENSG00000100239	ENST00000216061	PPP6R2
isotig05620	3.052	2.272	3.643	3.418	3785	0.273220861	isogroup00438	ENSG00000100239	ENST00000216061	PPP6R2
isotig05621	2.762	2.108	3.396	3.157	3764	0.263686406	isogroup00438	ENSG00000100239	ENST00000359139	PPP6R2
isotig05624	2.833	2.164	3.479	3.231	3704	0.266185091	isogroup00438	ENSG00000100239	ENST00000359139	PPP6R2
isotig05642	10.003	7.112	10.855	10.564	4743	0.248694296	isogroup00440	ENSG00000092607	ENST00000369429	TBX15
isotig05643	10.245	7.241	11.127	10.795	4356	0.24957729	isogroup00440	ENSG00000092607	ENST00000369429	TBX15
isotig05644	10.603	7.493	11.513	11.131	4174	0.263273089	isogroup00440	ENSG00000092607	ENST00000369429	TBX15
isotig05645	11.509	8.152	12.48	12.142	4121	0.265743594	isogroup00440	ENSG00000092607	ENST00000369429	TBX15
isotig05646	11.948	8.41	12.966	12.575	3734	0.26673931	isogroup00440	ENSG00000092607	ENST00000369429	TBX15
isotig05647	12.456	8.765	13.514	13.062	3552	0.268345608	isogroup00440	ENSG00000092607	ENST00000369429	TBX15
isotig05648	2.319	1.724	2.522	2.671	3236	0.106053205	isogroup00440	ENSG00000092607	ENST00000369429	TBX15
isotig05649	1.645	1.19	1.807	1.951	2849	0.060513264	isogroup00440	ENSG00000092607	ENST00000369429	TBX15
isotig05650	1.619	1.171	1.774	1.874	2667	0.07293154	isogroup00440	ENSG00000092607	ENST00000369429	TBX15
isotig05651	2.914	2.241	3.283	3.514	2559	0.097401743	isogroup00440	ENSG00000092607	ENST00000369429	TBX15
isotig05652	2.137	1.632	2.48	2.72	2172	0.039415345	isogroup00440	ENSG00000092607	ENST00000369429	TBX15
isotig05653	2.148	1.646	2.498	2.688	1990	0.057310062	isogroup00440	ENSG00000092607	ENST00000369429	TBX15
isotig05654	3.773	2.688	4.158	4.439	1958	0.165260765	isogroup00440	ENSG00000092607	ENST00000369429	TBX15
isotig05655	2.91	1.957	3.264	3.568	1571	0.128428647	isogroup00440	ENSG00000092607	ENST00000369429	TBX15
isotig05656	3.027	2.021	3.392	3.634	1389	0.153283986	isogroup00440	ENSG00000092607	ENST00000369429	TBX15
isotig05658	2.464	2.193	2.814	2.486	2586	0.18122041	isogroup00441	ENSG00000140983	ENST00000315082	RHOT2
isotig05659	1.789	1.733	2.13	1.807	2546	0.117710603	isogroup00441	ENSG00000140983	ENST00000315082	RHOT2
isotig05660	2.525	2.24	2.88	2.531	2508	0.18420756	isogroup00441	ENSG00000140983	ENST00000315082	RHOT2
isotig05661	2.478	2.23	2.844	2.541	2502	0.167580972	isogroup00441	ENSG00000140983	ENST00000315082	RHOT2
isotig05662	1.829	1.766	2.175	1.831	2468	0.119880514	isogroup00441	ENSG00000140983	ENST00000315082	RHOT2
isotig05663	1.78	1.754	2.136	1.839	2462	0.102314571	isogroup00441	ENSG00000140983	ENST00000315082	RHOT2
isotig05664	2.541	2.28	2.913	2.589	2424	0.17064327	isogroup00441	ENSG00000140983	ENST00000315082	RHOT2
isotig05665	1.821	1.789	2.183	1.865	2384	0.106814083	isogroup00441	ENSG00000140983	ENST00000315082	RHOT2
isotig05666	1.553	1.45	1.8	1.552	1998	0.1161137	isogroup00441	ENSG00000140983	ENST00000315082	RHOT2
isotig05667	1.595	1.481	1.845	1.572	1920	0.1161137	isogroup00441	ENSG00000140983	ENST00000315082	RHOT2
isotig05668	1.53	1.466	1.794	1.582	1914	0.097420531	isogroup00441	ENSG00000140983	ENST00000315082	RHOT2
isotig05669	1.472	1.269	1.694	1.397	1904	0.166303449	isogroup00441	ENSG00000140983	ENST00000315082	RHOT2

isotig05670	1.574	1.499	1.84	1.605	1836	0.098350492	isogroup00441	ENSG00000140983	ENST00000315082	RHOT2
isotig05671	1.513	1.295	1.737	1.412	1826	0.171817464	isogroup00441	ENSG00000140983	ENST00000315082	RHOT2
isotig05672	1.445	1.277	1.683	1.421	1820	0.148446306	isogroup00441	ENSG00000140983	ENST00000315082	RHOT2
isotig05673	1.487	1.304	1.727	1.439	1742	0.154392425	isogroup00441	ENSG00000140983	ENST00000315082	RHOT2
isotig05680	1.299	3.069	2.429	2.082	1787	0.388028857	isogroup00442	ENSG00000142910	ENST00000271064	TINAGL1
isotig05681	1.319	3.156	2.487	2.058	1782	0.383069061	isogroup00442	ENSG00000142910	ENST00000271064	TINAGL1
isotig05684	1.408	3.41	2.693	2.208	1698	0.403227625	isogroup00442	ENSG00000142910	ENST00000271064	TINAGL1
isotig05689	14.82	10.974	6.845	5.826	1932	0.560813857	isogroup00443	ENSG00000079308	ENST00000419504	TNS1
isotig05692	14.558	10.213	6.657	5.701	1867	0.603601488	isogroup00443	ENSG00000079308	ENST00000419504	TNS1
isotig05694	30.591	23.173	13.106	11.706	855	0.567398738	isogroup00443	ENSG00000165055	ENST00000419443	METTL2B
isotig05697	31.271	22.377	13.176	11.895	790	0.617212745	isogroup00443	ENSG00000165055	ENST00000419443	METTL2B
isotig05699	46.267	35.081	20.625	18.542	603	0.577966484	isogroup00443	ENSG00000167775	ENST00000537716	CD320
isotig05701	4.883	3.124	2.798	2.36	564	0.547794394	isogroup00443	ENSG00000167775	ENST00000537716	CD320
isotig05702	49.16	35.351	21.636	19.645	538	0.62113925	isogroup00443	ENSG00000167775	ENST00000537716	CD320
isotig05704	1.674	1.275	1.679	1.531	4662	0.189054633	isogroup00444	ENSG00000172046	ENST00000417901	USP19
isotig05705	1.456	1.179	1.534	1.416	4655	0.148934771	isogroup00444	ENSG00000172046	ENST00000417901	USP19
isotig05706	1.812	1.495	1.935	1.768	4637	0.169140302	isogroup00444	ENSG00000172046	ENST00000417901	USP19
isotig05707	1.671	1.268	1.666	1.513	4646	0.189740362	isogroup00444	ENSG00000172046	ENST00000417901	USP19
isotig05708	1.453	1.172	1.521	1.398	4639	0.148934771	isogroup00444	ENSG00000172046	ENST00000417901	USP19
isotig05709	1.81	1.489	1.923	1.751	4621	0.169140302	isogroup00444	ENSG00000172046	ENST00000417901	USP19
isotig05710	1.754	1.332	1.756	1.604	4570	0.194380777	isogroup00444	ENSG00000172046	ENST00000398892	USP19
isotig05711	1.533	1.234	1.609	1.488	4563	0.154392425	isogroup00444	ENSG00000172046	ENST00000417901	USP19
isotig05712	1.896	1.557	2.018	1.847	4545	0.178778087	isogroup00444	ENSG00000172046	ENST00000417901	USP19
isotig05713	1.753	1.324	1.744	1.586	4554	0.20052416	isogroup00444	ENSG00000172046	ENST00000398892	USP19
isotig05714	1.53	1.227	1.596	1.469	4547	0.154392425	isogroup00444	ENSG00000172046	ENST00000417901	USP19
isotig05715	1.894	1.55	2.006	1.83	4529	0.178778087	isogroup00444	ENSG00000172046	ENST00000417901	USP19
isotig05718	2.152	2.234	2.201	1.918	2551	0.021652138	isogroup00445	ENSG00000166333	ENST00000420936	ILK
isotig05719	2.403	2.691	2.575	2.251	2446	0.043172766	isogroup00445	ENSG00000166333	ENST00000420936	ILK
isotig05720	2.265	2.398	2.321	2.033	2446	0.003503795	isogroup00445	ENSG00000166333	ENST00000420936	ILK
isotig05721	2.273	2.395	2.359	2.061	2410	0.006660029	isogroup00445	ENSG00000166333	ENST00000420936	ILK
isotig05722	2.532	2.882	2.717	2.386	2341	0.064064026	isogroup00445	ENSG00000166333	ENST00000420936	ILK
isotig05723	2.544	2.886	2.763	2.421	2305	0.064064026	isogroup00445	ENSG00000166333	ENST00000420936	ILK
isotig05724	2.398	2.576	2.493	2.188	2305	0.021652138	isogroup00445	ENSG00000166333	ENST00000420936	ILK
isotig05725	2.688	3.099	2.923	2.572	2200	0.079835801	isogroup00445	ENSG00000166333	ENST00000420936	ILK
isotig05726	2.243	2.192	2.224	1.98	1917	0.063566168	isogroup00445	ENSG00000166333	ENST00000537806	ILK
isotig05727	2.401	2.411	2.387	2.139	1812	0.043172766	isogroup00445	ENSG00000166333	ENST00000537806	ILK
isotig05728	2.414	2.407	2.441	2.178	1776	0.043172766	isogroup00445	ENSG00000166333	ENST00000537806	ILK
isotig05729	2.595	2.657	2.631	2.363	1671	0.021652138	isogroup00445	ENSG00000166333	ENST00000537806	ILK
isotig05731	2.286	2.804	2.537	2.073	490	0.112901105	isogroup00445	ENSG00000166333	ENST00000528995	ILK
isotig05732	13.516	15.498	19.894	8.377	4896	0.757486661	isogroup00446	ENSG00000154153	ENST00000399793	FAM134B
isotig05738	13.99	16.513	20.627	8.835	4850	0.743950552	isogroup00446	ENSG00000154153	ENST00000399793	FAM134B
isotig05757	3.224	2.893	2.886	2.372	4670	0.263686406	isogroup00448	ENSG00000015479	ENST00000502929	MATR3
isotig05758	3.251	2.939	2.932	2.411	4644	0.260952882	isogroup00448	ENSG00000015479	ENST00000502929	MATR3
isotig05759	3.348	2.983	3.01	2.481	4526	0.275907417	isogroup00448	ENSG00000015479	ENST00000509990	MATR3
isotig05761	3.376	3.031	3.058	2.522	4500	0.268580446	isogroup00448	ENSG00000015479	ENST00000509990	MATR3
isotig05763	3.506	3.245	3.149	2.639	4187	0.235176975	isogroup00448	ENSG00000015479	ENST00000502929	MATR3
isotig05764	3.538	3.298	3.202	2.685	4161	0.229841437	isogroup00448	ENSG00000015479	ENST00000502929	MATR3
isotig05765	3.655	3.358	3.298	2.77	4043	0.241818216	isogroup00448	ENSG00000015479	ENST00000509990	MATR3
isotig05767	3.689	3.414	3.353	2.819	4017	0.239676486	isogroup00448	ENSG00000015479	ENST00000509990	MATR3
isotig05769	3.453	3.207	2.394	1.852	4169	0.077224393	isogroup00449	ENSG00000173210	ENST00000326685	ABLIM3
isotig05770	3.399	3.197	2.368	1.839	4121	0.067041783	isogroup00449	ENSG00000173210	ENST00000326685	ABLIM3
isotig05771	3.503	3.257	2.434	1.872	4052	0.077224393	isogroup00449	ENSG00000173210	ENST00000326685	ABLIM3
isotig05772	3.448	3.247	2.407	1.859	4004	0.067041783	isogroup00449	ENSG00000173210	ENST00000326685	ABLIM3
isotig05773	3.272	3.203	2.359	1.772	3301	0.03231382	isogroup00449	ENSG00000173210	ENST00000326685	ABLIM3
isotig05774	3.2	3.19	2.325	1.755	3253	0.017143233	isogroup00449	ENSG00000173210	ENST00000326685	ABLIM3
isotig05775	3.595	3.999	2.791	1.983	2645	0.038353874	isogroup00449	ENSG00000173210	ENST00000536903	ABLIM3
isotig05776	2.67	1.946	1.707	1.243	2509	0.278697302	isogroup00449	ENSG00000173210	ENST00000326685	ABLIM3
isotig05777	2.564	1.904	1.649	1.21	2461	0.265114226	isogroup00449	ENSG00000173210	ENST00000504238	ABLIM3
isotig05778	2.718	1.97	1.74	1.247	2392	0.284117382	isogroup00449	ENSG00000173210	ENST00000326685	ABLIM3
isotig05779	2.607	1.926	1.68	1.212	2344	0.270543699	isogroup00449	ENSG00000173210	ENST00000504238	ABLIM3
isotig05782	7.49	6.849	8.782	8.523	3101	0.172738033	isogroup00450	ENSG00000183020	ENST00000448903	AP2A2
isotig05783	10.218	8.816	11.376	11.855	3100	0.194662584	isogroup00450	ENSG00000183020	ENST00000448903	AP2A2
isotig05784	9.246	7.885	10.471	10.375	2099	0.205023672	isogroup00450	ENSG00000183020	ENST00000448903	AP2A2
isotig05785	13.279	10.792	14.304	15.299	2098	0.228197565	isogroup00450	ENSG00000183020	ENST00000448903	AP2A2
isotig05806	2.19	1.68	1.935	1.463	7333	0.288119035	isogroup00452	ENSG00000134313	ENST00000256707	KIDINS220
isotig05807	2.181	1.69	1.94	1.477	7297	0.282520478	isogroup00452	ENSG00000134313	ENST00000256707	KIDINS220
isotig05808	2.171	1.697	1.93	1.461	7207	0.278283986	isogroup00452	ENSG00000134313	ENST00000473731	KIDINS220
isotig05809	1.765	1.336	1.396	1.216	4920	0.213506049	isogroup00452	ENSG00000134313	ENST00000256707	KIDINS220
isotig05811	1.749	1.347	1.398	1.234	4884	0.202459232	isogroup00452	ENSG00000134313	ENST00000256707	KIDINS220
isotig05813	1.726	1.352	1.374	1.206	4794	0.196456752	isogroup00452	ENSG00000134313	ENST00000473731	KIDINS220
isotig05817	2.924	2.627	2.775	2.479	4033	0.19021004	isogroup00453	ENSG00000188021	ENST00000338222	UBQLN2
isotig05818	2.955	2.626	2.788	2.476	4028	0.203191929	isogroup00453	ENSG00000188021	ENST00000338222	UBQLN2
isotig05819	3.037	1.982	2.241	1.848	3632	0.396238822	isogroup00453	ENSG00000135018	ENST00000376395	UBQLN1
isotig05820	3.072	1.98	2.255	1.843	3627	0.40831893	isogroup00453	ENSG00000135018	ENST00000376395	UBQLN1
isotig05821	2.978	1.844	2.17	1.784	3548	0.406909897	isogroup00453	ENSG00000135018	ENST00000376395	UBQLN1
isotig05822	3.014	1.842	2.184	1.779	3543	0.418726986	isogroup00453	ENSG00000135018	ENST00000376395	UBQLN1
isotig05823	2.743	2.816	2.819	2.609	3356	0.077224393	isogroup00453	ENSG00000188021	ENST00000338222	UBQLN2
isotig05824	2.747	2.8	2.794	2.585	3351	0.087162771	isogroup00453	ENSG00000188021	ENST00000338222	UBQLN2
isotig05825	2.857	2.049	2.168	1.851	2955	0.333658976	isogroup00453	ENSG00000135018	ENST00000376395	UBQLN1
isotig05826	2.862	2.029	2.139	1.822	2950	0.343512813	isogroup00453	ENSG00000135018	ENST00000376395	UBQLN1
isotig05827	2.779	1.88	2.078	1.772	2871	0.35027617	isogroup00453	ENSG00000135018	ENST00000376395	UBQLN1
isotig05828	2.784	1.86	2.048	1.742	2866	0.359791839	isogroup00453	ENSG00000135018	ENST00000376395	UBQLN1

isotig05829	1.361	2.199	2.667	2.741	3317	0.196813707	isogroup00454	ENSG00000131196	ENST00000545796	NFATC1
isotig05830	1.465	2.381	2.879	3.045	3185	0.224674983	isogroup00454	ENSG00000131196	ENST00000545796	NFATC1
isotig05841	5.039	3.503	3.86	4.357	2497	0.274658075	isogroup00455	ENSG00000185761	ENST00000413997	ADAMTSL5
isotig05842	5.485	3.443	4.131	4.585	2451	0.358983993	isogroup00455	ENSG00000185761	ENST00000413997	ADAMTSL5
isotig05843	5.221	3.702	4.056	4.693	2358	0.234744871	isogroup00455	ENSG00000185761	ENST00000413997	ADAMTSL5
isotig05844	4.523	2.946	3.469	3.639	2338	0.339454798	isogroup00455	ENSG00000185761	ENST00000413997	ADAMTSL5
isotig05845	5.697	3.642	4.347	4.941	2312	0.325167205	isogroup00455	ENSG00000185761	ENST00000413997	ADAMTSL5
isotig05846	4.685	3.124	3.655	3.954	2199	0.299654317	isogroup00455	ENSG00000185761	ENST00000413997	ADAMTSL5
isotig05847	5.958	3.618	4.429	5.002	2175	0.375742091	isogroup00455	ENSG00000185761	ENST00000413997	ADAMTSL5
isotig05848	6.231	3.856	4.694	5.435	2036	0.342188322	isogroup00455	ENSG00000185761	ENST00000413997	ADAMTSL5
isotig05849	2.79	2.51	2.106	2.371	1279	0.028237018	isogroup00455	ENSG00000185761	ENST00000413997	ADAMTSL5
isotig05850	3.593	2.354	2.578	2.749	1233	0.280350567	isogroup00455	ENSG00000185761	ENST00000413997	ADAMTSL5
isotig05851	4.121	2.437	2.808	3.168	957	0.319390171	isogroup00455	ENSG00000185761	ENST00000413997	ADAMTSL5
isotig05852	4.897	5.063	4.045	4.346	783	0.089520553	isogroup00455	ENSG00000152475	ENST00000427624	ZNF837
contig09138	3.161	3.025	4.015	3.097	3058	0.17035207	isogroup00456	ENSG00000142599	ENST00000476556	RERE
isotig05856	1.509	1.711	2.151	1.756	810	0.034718569	isogroup00456	ENSG00000110756	ENST00000537258	HPS5
isotig05857	1.296	1.136	1.323	1.101	770	0.080934846	isogroup00456	ENSG00000110756	ENST00000537258	HPS5
isotig05858	1.504	1.163	1.255	1.12	557	0.125300594	isogroup00456	ENSG00000110756	ENST00000537258	HPS5
isotig05859	2.814	3.025	4.064	3.181	393	0.125638762	isogroup00456	ENSG00000112297	ENST00000285105	AIM1
isotig05860	3.586	3.122	3.819	2.873	353	0.226102803	isogroup00456	ENSG00000154473	ENST00000538238	BUB3
isotig06097	2.724	2.107	3.074	2.534	5484	0.332682047	isogroup00487	ENSG00000175662	ENST00000379504	TOM1L2
isotig06098	2.768	2.098	3.127	2.539	5357	0.348585331	isogroup00487	ENSG00000175662	ENST00000379504	TOM1L2
isotig06099	2.621	2.031	3.016	2.456	5325	0.326886225	isogroup00487	ENSG00000175662	ENST00000535933	TOM1L2
isotig06100	2.664	2.02	3.069	2.459	5198	0.345973923	isogroup00487	ENSG00000175662	ENST00000535933	TOM1L2
isotig06101	2.598	2.035	3.076	2.49	4294	0.33795183	isogroup00487	ENSG00000175662	ENST00000542206	TOM1L2
isotig06102	2.684	2.051	2.497	2.134	1621	0.287423912	isogroup00487	ENSG00000175662	ENST00000379504	TOM1L2
isotig06103	2.304	1.771	2.223	1.807	1462	0.271680319	isogroup00487	ENSG00000175662	ENST00000535933	TOM1L2
isotig06104	2.994	1.773	4.081	2.128	1294	0.61270384	isogroup00487	ENSG00000126562	ENST00000246914	WNK4
isotig06105	5.771	4.446	5.688	6.33	8281	0.247839483	isogroup00488	ENSG00000164307	ENST00000443439	ERAP1
isotig06106	5.822	4.617	6.038	6.597	8079	0.238859247	isogroup00488	ENSG00000164307	ENST00000443439	ERAP1
isotig06107	5.337	4.019	5.398	5.9	7488	0.277927031	isogroup00488	ENSG00000164307	ENST00000443439	ERAP1
isotig06108	11.391	9.618	10.452	11.842	2259	0.138028857	isogroup00488	ENSG00000153113	ENST00000510756	CAST
isotig06109	12.146	10.799	12.296	13.435	2057	0.124849703	isogroup00488	ENSG00000153113	ENST00000511049	CAST
isotig06110	12.215	10.238	11.55	12.629	1466	0.182103404	isogroup00488	ENSG00000153113	ENST00000515663	CAST
isotig06111	1.588	1.387	1.496	1.282	4393	0.142885324	isogroup00489	ENSG00000117625	ENST00000419091	RCOR3
isotig06112	1.65	1.446	1.548	1.359	4260	0.141805065	isogroup00489	ENSG00000117625	ENST00000419091	RCOR3
isotig06113	1.567	1.297	1.432	1.254	4018	0.155312993	isogroup00489	ENSG00000117625	ENST00000367006	RCOR3
isotig06115	1.419	2.256	1.668	1.786	1909	0.289265048	isogroup00489	ENSG00000117625	ENST00000419091	RCOR3
isotig06116	1.556	2.462	1.806	2.007	1776	0.317840234	isogroup00489	ENSG00000117625	ENST00000419091	RCOR3
isotig06117	1.398	1.826	1.441	1.66	1423	0.211730668	isogroup00489	ENSG00000117625	ENST00000367006	RCOR3
isotig06118	1.229	1.868	1.31	1.273	1155	0.166726159	isogroup00489	ENSG00000117625	ENST00000452621	RCOR3
isotig06119	2.736	2.251	2.025	2.256	4530	0.06751146	isogroup00490	ENSG00000040341	ENST00000519961	STAU2
isotig06120	2.774	2.254	2.067	2.27	4398	0.084617119	isogroup00490	ENSG00000040341	ENST00000519961	STAU2
isotig06121	3.046	4.584	2.296	3.199	3223	0.516269633	isogroup00490	ENSG00000040341	ENST00000524300	STAU2
isotig06123	3.114	4.688	2.367	3.259	3091	0.514268806	isogroup00490	ENSG00000040341	ENST00000524300	STAU2
isotig06125	11.237	6.415	6.904	6.295	3999	0.563331329	isogroup00491	ENSG00000144746	ENST00000273258	ARL6IP5
isotig06126	9.102	5.064	5.641	4.955	3955	0.558850605	isogroup00491	ENSG00000144746	ENST00000273258	ARL6IP5
isotig06127	15.838	8.529	8.889	7.959	2709	0.633773578	isogroup00491	ENSG00000144746	ENST00000273258	ARL6IP5
isotig06128	12.746	6.559	7.048	5.999	2665	0.649892914	isogroup00491	ENSG00000144746	ENST00000273258	ARL6IP5
isotig06132	5.079	2.746	3.051	2.8	3453	0.492419403	isogroup00492	ENSG00000068615	ENST00000165698	REEP1
isotig06133	5.201	2.714	2.98	2.598	3303	0.534718569	isogroup00492	ENSG00000170836	ENST00000544712	PPM1D
isotig06134	5.21	2.795	3.166	2.88	3297	0.502348388	isogroup00492	ENSG00000068615	ENST00000165698	REEP1
isotig06136	3.506	1.95	1.974	2.337	1255	0.329300368	isogroup00492	ENSG00000068615	ENST00000165698	REEP1
isotig06137	3.657	1.746	1.615	1.671	1105	0.482913128	isogroup00492	ENSG00000170836	ENST00000544712	PPM1D
isotig06138	3.677	1.985	2.166	2.51	1099	0.359650936	isogroup00492	ENSG00000068615	ENST00000165698	REEP1
isotig06139	4.071	2.757	4.121	3.295	2189	0.452299542	isogroup00493	ENSG00000182952	ENST00000377575	HMGN4
isotig06140	4.124	2.877	4.305	3.516	2134	0.441224543	isogroup00493	ENSG00000196872	ENST00000397899	C2orf55
isotig06141	4.062	2.789	4.18	3.274	2123	0.453868265	isogroup00493	ENSG00000182952	ENST00000377575	HMGN4
isotig06142	4.117	2.914	4.371	3.502	2068	0.441224543	isogroup00493	ENSG00000182952	ENST00000377575	HMGN4
isotig06143	4.508	2.953	4.623	3.617	2031	0.496674682	isogroup00493	ENSG00000182952	ENST00000377575	HMGN4
isotig06144	4.513	2.994	4.704	3.606	1965	0.497623431	isogroup00493	ENSG00000182952	ENST00000377575	HMGN4
isotig06145	4.357	3.02	4.586	3.571	1863	0.457381453	isogroup00493	ENSG00000182952	ENST00000377575	HMGN4
isotig06146	4.553	6.01	5.772	5.337	2593	0.250150297	isogroup00494	ENSG00000146476	ENST00000367294	C6orf211
isotig06147	4.731	6.45	6.134	5.723	2375	0.28818479	isogroup00494	ENSG00000146476	ENST00000367294	C6orf211
isotig06148	4.863	7.355	9.874	7.352	1626	0.062654994	isogroup00494	ENSG00000159082	ENST00000433931	SYNJ1
isotig06150	5.255	8.335	11.161	8.3	1408	0.034840685	isogroup00494	ENSG00000159082	ENST00000433931	SYNJ1
isotig06152	4.408	3.553	4.439	3.824	882	0.231062599	isogroup00494	ENSG00000146476	ENST00000367294	C6orf211
isotig06153	51.697	72.319	77.747	38.785	2099	0.438829188	isogroup00495	ENSG00000138029	ENST00000317799	HADHB
isotig06154	48.304	67.519	72.646	36.278	2054	0.438228	isogroup00495	ENSG00000138029	ENST00000317799	HADHB
isotig06159	50.514	63.372	66.684	53.622	2605	0.040645901	isogroup00496	ENSG00000165678	ENST00000372134	GHITM
isotig06161	51.786	70.027	72.377	60.403	1391	0.121251973	isogroup00496	ENSG00000165678	ENST00000372134	GHITM
isotig06163	35.016	46.65	49.316	42.281	488	0.08866574	isogroup00496	ENSG00000165678	ENST00000436406	GHITM
isotig06165	119.042	97.952	106.352	133.168	2752	0.090732321	isogroup00497	ENSG00000105220	ENST00000415930	GPI
isotig06166	143.417	118.168	128.147	160.838	2336	0.106776509	isogroup00497	ENSG00000105220	ENST00000356487	GPI
isotig06167	59.345	48.489	53.089	64.853	2181	0.089426618	isogroup00497	ENSG00000105220	ENST00000415930	GPI
isotig06168	77.536	63.587	69.381	85.374	1765	0.105912302	isogroup00497	ENSG00000105220	ENST00000415930	GPI
isotig06169	1.817	1.876	1.739	1.458	1401	0.065266401	isogroup00498	ENSG00000147669	ENST00000353107	POLR2K
isotig06170	2.158	2.191	2.137	1.803	1369	0.108908845	isogroup00498	ENSG00000147669	ENST00000353107	POLR2K
isotig06173	3.879	3.4	3.683	3.206	480	0.228122417	isogroup00498	ENSG00000147669	ENST00000353107	POLR2K
isotig06176	4.935	4.341	4.942	4.242	463	0.303176899	isogroup00498	ENSG00000147669	ENST00000353107	POLR2K
isotig06177	15.358	28.871	21.965	20.931	2115	0.552632073	isogroup00499	ENSG00000175376	ENST00000533544	EIF1AD
isotig06179	36.329	73.41	52.986	51.573	782	0.630279176	isogroup00499	ENSG00000175334	ENST00000533166	BANF1

isotig06181	5.666	17.749	16.867	20.256	578	0.768336214	isogroup00499	ENSG00000175315	ENST00000312134	CST6	
isotig06182	2.894	1.86	2.187	1.534	1277	0.467798903	isogroup00500	ENSG00000196663	ENST00000380088	TECPR2	
isotig06183	42.217	32.467	27.204	23.562	998	0.444315022	isogroup00500	ENSG00000233927	ENST00000449223	RPS28	
isotig06184	123.75	103.517	81.376	76.518	743	0.247228902	isogroup00500	ENSG00000233927	ENST00000449223	RPS28	
isotig06185	129.624	108.098	85.175	80.042	691	0.247379199	isogroup00500	ENSG00000233927	ENST00000449223	RPS28	
isotig06186	293.937	243.448	185.497	182.192	636	0.279505148	isogroup00500	ENSG00000233927	ENST00000417088	RPS28	
isotig06187	2.898	2.179	2.187	1.946	627	0.187579845	isogroup00500	ENSG00000186994	ENST00000330915	KANK3	
isotig06188	621.409	523.213	397.081	391.633	381	0.233504922	isogroup00500	ENSG00000233927	ENST00000417088	RPS28	
isotig06189	1.462	2.246	2.26	2.23	793	0.208067183	isogroup00501	ENSG00000214413	ENST00000448814	BBIP1	
isotig06191	1.665	2.251	2.517	2.335	690	0.167487037	isogroup00501	ENSG00000214413	ENST00000448814	BBIP1	
isotig06192	1.269	1.68	1.878	1.81	696	0.162705719	isogroup00501	ENSG00000214413	ENST00000448814	BBIP1	
isotig06193	1.797	2.529	2.701	2.501	595	0.197396107	isogroup00501	ENSG00000214413	ENST00000448814	BBIP1	
isotig06194	1.441	1.804	2.134	2.043	606	0.153706696	isogroup00501	ENSG00000214413	ENST00000448814	BBIP1	
isotig06195	36.471	34.037	45.626	39.113	6751	0.257909371	isogroup00502	ENSG00000091436	ENST00000539448	AC013461.1.1	
isotig06196	36.911	34.473	46.266	39.609	6593	0.257956339	isogroup00502	ENSG00000091436	ENST00000539448	AC013461.1.1	
isotig06197	10.584	7.926	10.228	9.267	918	0.469301871	isogroup00502	ENSG00000091436	ENST00000539448	AC013461.1.1	
isotig06198	9.013	6.28	8.425	7.364	760	0.513150973	isogroup00502	ENSG00000091436	ENST00000539448	AC013461.1.1	
isotig06199	7.443	4.411	5.866	4.974	4723	0.53490644	isogroup00503	ENSG00000196998	ENST00000322995	WDR45	
isotig06200	17.917	15.232	23.147	17.318	1451	0.497200721	isogroup00503	ENSG00000196998	ENST00000485908	WDR45	
isotig06201	3.583	2.364	3.133	2.301	1370	0.512897347	isogroup00503	ENSG00000196998	ENST00000471338	WDR45	
isotig06202	4.58	3.538	4.14	3.265	1281	0.441121214	isogroup00503	ENSG00000196998	ENST00000471338	WDR45	
isotig06203	10.252	8.469	12.415	9.432	960	0.520186744	isogroup00503	ENSG00000196998	ENST00000322995	WDR45	
isotig06204	3.436	4.138	4.449	4.409	2238	0.090957767	isogroup00504	ENSG00000114388	ENST00000232501	NPRL2	
isotig06205	2.561	2.64	15.126	23.705	1694	0.362562937	isogroup00504	ENSG00000230034	ENST00000546794	PSMB8	
isotig06206	3.001	3.075	17.882	27.449	1622	0.344273691	isogroup00504	ENSG00000230034	ENST00000546794	PSMB8	
isotig06207	5.013	5.911	5.25	4.376	1486	0.0314684	isogroup00504	ENSG00000114388	ENST00000232501	NPRL2	
isotig06208	1.268	1.29	2.526	3.162	1347	0.028237018	isogroup00504	ENSG00000114388	ENST00000232501	NPRL2	
isotig06209	2.467	2.119	2.094	1.504	595	0.292158263	isogroup00504	ENSG00000114388	ENST00000232501	NPRL2	
isotig06210	644.86	499.987	429.586	333.367	607	0.495190501	isogroup00505	ENSG00000161970	ENST00000293842	RPL26	
isotig06211	9.532	4.74	9.542	5.481	1170	0.772676035	isogroup00505	ENSG00000179010	ENST00000507420	MRFAP1	
isotig06212	9.493	4.644	9.526	5.258	1141	0.784089201	isogroup00505	ENSG00000178988	ENST00000320848	MRFAP1L1	
isotig06214	4.141	1.941	3.879	1.972	611	0.738699557	isogroup00505	ENSG00000179010	ENST00000507420	MRFAP1	
contig09688	5.84	7.868	6.749	5.21	921	0.140752987	isogroup00506	ENSG00000060762	ENST00000360961	BRP44L	
isotig06215	76.456	108.93	78.88	71.476	973	0.306830991	isogroup00506	ENSG00000060762	ENST00000360961	BRP44L	
isotig06216	83.213	118.556	85.85	77.792	894	0.307159766	isogroup00506	ENSG00000060762	ENST00000360961	BRP44L	
isotig06217	335.283	225.16	242.038	191.552	1235	0.614939506	isogroup00507	ENSG00000147604	ENST00000352983	RPL7	
isotig06218	470.822	312.958	337.873	267.372	860	0.62457729	isogroup00507	ENSG00000147604	ENST00000352983	RPL7	
isotig06219	40.027	36.232	30.987	22.907	710	0.456169685	isogroup00508	ENSG00000143198	ENST00000404549	MGST3	
isotig06220	31.674	28.418	24.469	17.643	715	0.474355602	isogroup00508	ENSG00000143198	ENST00000367889	MGST3	
isotig06222	219.905	307.174	202.595	175.011	556	0.264606974	isogroup00509	ENSG00000131174	ENST00000481445	COX7B	
isotig06223	263.877	369.011	243.438	210.015	463	0.264804238	isogroup00509	ENSG00000131174	ENST00000481445	COX7B	
isotig06224	188.549	260.302	176.549	149.121	463	0.248947922	isogroup00509	ENSG00000131174	ENST00000481445	COX7B	
isotig06225	1.689	1.026	1.783	3.207	3446	0.085528293	isogroup00510	ENSG00000182179	ENST00000333486	UBA7	
isotig06226	1.698	1.047	1.8	3.276	3363	0.071428571	isogroup00510	ENSG00000182179	ENST00000333486	UBA7	
isotig06230	28.898	12.62	29.574	23.899	3127	0.6947847	isogroup00511	ENSG00000141905	ENST00000395111	NFIC	
isotig06231	28.708	12.527	29.275	23.647	3041	0.694700158	isogroup00511	ENSG00000141905	ENST00000341919	NFIC	
isotig06232	13.368	5.83	14.659	11.139	1968	0.706770872	isogroup00511	ENSG00000141905	ENST00000343825	NFIC	
isotig06233	12.798	5.588	13.952	10.697	1882	0.704460059	isogroup00511	ENSG00000142279	ENST00000270288	WTIP	
isotig06234	34.72	34.559	23.465	19.837	2744	0.209476216	isogroup00512	ENSG00000116171	ENST00000371514	SCP2	
isotig06235	35.299	35.135	23.856	20.168	2699	0.209551364	isogroup00512	ENSG00000116171	ENST00000371514	SCP2	
isotig06237	87.007	97.923	95.154	84.143	2077	0.008482378	isogroup00513	ENSG00000146729	ENST00000322090	GBAS	
isotig06238	90.087	101.37	98.512	87.066	2006	0.008491771	isogroup00513	ENSG00000146729	ENST00000322090	GBAS	
isotig06239	30.844	32.998	30.467	29.517	1459	0.055675584	isogroup00513	ENSG00000146729	ENST00000322090	GBAS	
isotig06240	405.981	295.033	605.185	607.041	1485	0.138545502	isogroup00514	ENSG00000196218	ENST00000359596	RYR1	
isotig06242	429.346	311.905	639.955	641.807	1404	0.138554896	isogroup00514	ENSG00000196218	ENST00000359596	RYR1	
isotig06243	74.582	51.584	113.254	102.779	646	0.349590441	isogroup00514	ENSG00000196218	ENST00000359596	RYR1	
isotig06249	18.663	21.775	25.945	23.743	2344	0.023070564	isogroup00516	ENSG00000082641	ENST00000362042	NFE2L1	
isotig06250	33.4	36.356	45.485	40.065	1267	0.133942662	isogroup00516	ENSG00000082641	ENST00000362042	NFE2L1	
isotig06251	41.162	47.178	58.205	52.991	933	0.056727662	isogroup00516	ENSG00000082641	ENST00000362042	NFE2L1	
isotig06252	40.463	43.363	58.527	50.603	843	0.212980011	isogroup00516	ENSG00000082641	ENST00000536222	NFE2L1	
isotig06253	10.505	11.88	14.806	11.094	1345	0.139682122	isogroup00517	ENSG00000125971	ENST00000374846	DYNLRB1	
isotig06254	12.464	13.912	17.684	12.637	1124	0.158431653	isogroup00517	ENSG00000125971	ENST00000357156	DYNLRB1	
isotig06255	137.691	68.32	102.734	105.177	640	0.65948373	isogroup00518	ENSG00000221857	ENST00000455515	FXYD1	
isotig06256	241.717	118.543	184.483	189.336	580	0.67019238	isogroup00518	ENSG00000221857	ENST00000455515	FXYD1	
isotig06257	159.952	79.333	120.648	122.415	540	0.668041256	isogroup00518	ENSG00000221857	ENST00000455515	FXYD1	
isotig06258	69.292	76.738	66.868	62.758	824	0.121421057	isogroup00519	ENSG00000122873	ENST00000333926	CISD1	
isotig06260	1.739	1.272	1.711	1.229	3974	0.235458781	isogroup00520	ENSG00000151164	ENST00000358071	RAD9B	
isotig06261	12.151	9.048	10.361	9.252	1826	0.443629293	isogroup00520	ENSG00000120860	ENST00000240079	CCDC53	
isotig06262	13.847	20.523	21.641	25.698	1592	0.489958293	isogroup00521	ENSG00000132429	ENST00000254765	POPDC3	
isotig06263	15.953	24.143	25.105	29.292	1522	0.503597731	isogroup00521	ENSG00000132429	ENST00000254765	POPDC3	
isotig06264	16.335	20.022	23.709	28.062	1470	0.349674983	isogroup00521	ENSG00000132429	ENST00000254765	POPDC3	
isotig06269	146.681	128.442	118.249	100.195	615	0.308286992	isogroup00523	ENSG00000169567	ENST00000304043	HINT1	
isotig06271	140.826	124.89	112.609	96.924	563	0.278020966	isogroup00523	ENSG00000169567	ENST00000304043	HINT1	
isotig06272	3.942	3.031	4.39	2.025	1473	0.67804539	isogroup00524	ENSG00000164104	ENST00000446922	HMGB2	
isotig06278	4.358	3.349	4.999	2.313	1401	0.697903359	isogroup00524	ENSG00000164104	ENST00000446922	HMGB2	
isotig06284	4.679	3.558	5.211	2.404	1241	0.710716164	isogroup00524	ENSG00000164104	ENST00000446922	HMGB2	
isotig06290	5.223	3.973	5.991	2.772	1169	0.729803863	isogroup00524	ENSG00000164104	ENST00000446922	HMGB2	
isotig06296	6.337	6.669	4.008	5.038	9936	0.237487788	isogroup00525	ENSG00000103994	ENST00000263805	ZFP106	
isotig06297	6.435	6.775	4.066	5.119	9891	0.238192305	isogroup00525	ENSG00000103994	ENST00000263805	ZFP106	
isotig06298	6.382	6.716	4.036	5.074	9866	0.237487788	isogroup00525	ENSG00000103994	ENST00000263805	ZFP106	
isotig06299	6.175	6.468	3.915	4.904	9816	0.224975577	isogroup00525	ENSG00000103994	ENST00000263805	ZFP106	
isotig06300	6.481	6.823	4.095	5.156	9821	0.238859247	isogroup00525	ENSG00000103994	ENST00000263805	ZFP106	

isotig06301	6.274	6.574	3.974	4.985	9771	0.23582513	isogroup00525	ENSG00000103994	ENST00000263805	ZFP106
isotig06302	6.22	6.514	3.944	4.939	9746	0.224975577	isogroup00525	ENSG00000103994	ENST00000263805	ZFP106
isotig06303	6.319	6.621	4.003	5.021	9701	0.23582513	isogroup00525	ENSG00000103994	ENST00000263805	ZFP106
isotig06304	6.232	6.586	4.013	5.054	9712	0.247050425	isogroup00525	ENSG00000103994	ENST00000263805	ZFP106
isotig06305	6.332	6.694	4.073	5.137	9667	0.248637935	isogroup00525	ENSG00000103994	ENST00000263805	ZFP106
isotig06306	6.277	6.633	4.042	5.091	9642	0.247839483	isogroup00525	ENSG00000103994	ENST00000263805	ZFP106
isotig06307	6.066	6.379	3.918	4.917	9592	0.234876381	isogroup00525	ENSG00000103994	ENST00000263805	ZFP106
isotig06308	6.378	6.742	4.103	5.175	9597	0.248637935	isogroup00525	ENSG00000103994	ENST00000263805	ZFP106
isotig06309	6.166	6.487	3.979	5	9547	0.236614188	isogroup00525	ENSG00000103994	ENST00000263805	ZFP106
isotig06310	6.11	6.425	3.947	4.953	9522	0.23582513	isogroup00525	ENSG00000103994	ENST00000263805	ZFP106
isotig06311	6.212	6.535	4.008	5.037	9477	0.236614188	isogroup00525	ENSG00000103994	ENST00000263805	ZFP106
isotig06312	5.322	5.392	3.885	4.336	5659	0.154984219	isogroup00525	ENSG00000103994	ENST00000565611	ZFP106
isotig06313	5.487	5.568	3.987	4.472	5614	0.157961975	isogroup00525	ENSG00000103994	ENST00000565611	ZFP106
isotig06314	5.389	5.459	3.933	4.39	5589	0.154984219	isogroup00525	ENSG00000103994	ENST00000565611	ZFP106
isotig06315	5.556	5.638	4.037	4.529	5544	0.157961975	isogroup00525	ENSG00000103994	ENST00000565611	ZFP106
isotig06336	3.486	4.274	3.405	2.834	4834	0.106607425	isogroup00527	ENSG00000100201	ENST00000403230	DDX17
isotig06337	3.409	4.203	3.338	2.756	4805	0.106607425	isogroup00527	ENSG00000100201	ENST00000403230	DDX17
isotig06338	3.515	4.319	3.434	2.856	4777	0.108561284	isogroup00527	ENSG00000100201	ENST00000403230	DDX17
isotig06339	3.438	4.246	3.366	2.778	4748	0.108561284	isogroup00527	ENSG00000100201	ENST00000403230	DDX17
isotig06340	3.311	3.99	3.603	3.04	3990	0.072142481	isogroup00527	ENSG00000100201	ENST00000381633	DDX17
isotig06341	3.217	3.901	3.523	2.947	3961	0.064082814	isogroup00527	ENSG00000100201	ENST00000381633	DDX17
isotig06342	3.344	4.04	3.642	3.07	3933	0.072142481	isogroup00527	ENSG00000100201	ENST00000381633	DDX17
isotig06343	3.248	3.95	3.561	2.976	3904	0.06991621	isogroup00527	ENSG00000100201	ENST00000381633	DDX17
isotig06344	2.885	3.16	3.307	2.513	3593	0.110749981	isogroup00527	ENSG00000100201	ENST00000444597	DDX17
isotig06345	2.777	3.055	3.216	2.406	3564	0.118715714	isogroup00527	ENSG00000100201	ENST00000444597	DDX17
isotig06346	2.915	3.202	3.346	2.538	3536	0.110749981	isogroup00527	ENSG00000100201	ENST00000444597	DDX17
isotig06347	2.805	3.096	3.253	2.429	3507	0.118715714	isogroup00527	ENSG00000100201	ENST00000444597	DDX17
isotig06348	8.097	11.373	4.364	5.387	3107	0.527288269	isogroup00527	ENSG00000100201	ENST00000404499	DDX17
isotig06349	4.2	5.278	2.842	2.815	1954	0.288879913	isogroup00527	ENSG00000100201	ENST00000404499	DDX17
isotig06350	4.114	5.019	3.127	3.541	1110	0.291923424	isogroup00527	ENSG00000100201	ENST00000381633	DDX17
isotig06351	3.304	2.552	3.389	3.244	4466	0.292064327	isogroup00528	ENSG00000142453	ENST00000327064	CARM1
isotig06352	3.196	2.438	3.276	3.132	4397	0.293783347	isogroup00528	ENSG00000142453	ENST00000344150	CARM1
isotig06353	3.359	2.603	3.45	3.298	4386	0.294299992	isogroup00528	ENSG00000142453	ENST00000327064	CARM1
isotig06355	3.25	2.488	3.335	3.185	4317	0.296319606	isogroup00528	ENSG00000142453	ENST00000344150	CARM1
isotig06357	3.29	2.556	3.378	3.234	4101	0.300650034	isogroup00528	ENSG00000142453	ENST00000327064	CARM1
isotig06358	3.173	2.431	3.254	3.111	4032	0.302979635	isogroup00528	ENSG00000142453	ENST00000344150	CARM1
isotig06359	3.35	2.611	3.444	3.292	4021	0.302462989	isogroup00528	ENSG00000142453	ENST00000327064	CARM1
isotig06361	3.231	2.485	3.319	3.169	3952	0.304989855	isogroup00528	ENSG00000142453	ENST00000344150	CARM1
isotig06363	3.919	2.981	3.857	3.742	1777	0.368246036	isogroup00528	ENSG00000142453	ENST00000327064	CARM1
isotig06376	2.781	1.742	1.662	1.77	1080	0.306098294	isogroup00529	ENSG00000073169	ENST00000380903	RP3-402G11.5.1
isotig06381	3.846	2.965	1.014	1.089	3065	0.327196213	isogroup00530	ENSG00000162409	ENST00000371244	PRKAA2
isotig06390	2.734	3.671	4.176	3.174	1371	0.072142481	isogroup00531	ENSG00000124596	ENST00000479950	C6orf130
isotig06391	1.469	1.575	1.762	1.334	1538	0.11153904	isogroup00531	ENSG00000124596	ENST00000479950	C6orf130
isotig06392	2.462	3.484	3.751	2.828	1226	0.113323815	isogroup00531	ENSG00000124596	ENST00000486443	C6orf130
isotig06393	2.956	3.947	4.636	3.393	1180	0.040580146	isogroup00531	ENSG00000124596	ENST00000479950	C6orf130
isotig06396	1.485	1.519	1.823	1.265	1347	0.170981438	isogroup00531	ENSG00000124596	ENST00000479950	C6orf130
isotig06398	2.666	3.763	4.198	3.014	1035	0.086157661	isogroup00531	ENSG00000124596	ENST00000486443	C6orf130
isotig06402	2.631	3.811	4.168	3.008	841	0.141889607	isogroup00531	ENSG00000124596	ENST00000486443	C6orf130
isotig06403	2.316	2.923	1.775	1.76	8277	0.233570677	isogroup00532	ENSG00000069122	ENST00000452370	GPR116
isotig06404	2.27	2.861	1.734	1.723	8239	0.230433231	isogroup00532	ENSG00000069122	ENST00000452370	GPR116
isotig06405	2.341	2.934	1.796	1.788	8157	0.227483655	isogroup00532	ENSG00000069122	ENST00000452370	GPR116
isotig06406	2.294	2.87	1.754	1.751	8119	0.224045615	isogroup00532	ENSG00000069122	ENST00000452370	GPR116
isotig06407	2.401	3.015	1.845	1.833	8044	0.237074472	isogroup00532	ENSG00000069122	ENST00000452370	GPR116
isotig06408	2.354	2.951	1.802	1.795	8006	0.233570677	isogroup00532	ENSG00000069122	ENST00000452370	GPR116
isotig06409	2.427	3.027	1.867	1.863	7924	0.230489592	isogroup00532	ENSG00000069122	ENST00000452370	GPR116
isotig06410	2.379	2.962	1.824	1.824	7886	0.227483655	isogroup00532	ENSG00000069122	ENST00000452370	GPR116
isotig06423	1.637	1.04	1.626	1.137	3836	0.310146915	isogroup00533	ENSG00000120549	ENST00000450158	KIAA1217
isotig06424	1.641	1.043	1.623	1.14	3800	0.305393778	isogroup00533	ENSG00000120549	ENST00000450158	KIAA1217
isotig06427	4.864	3.612	3.998	3.396	3210	0.368264823	isogroup00534	ENSG00000244005	ENST00000374092	NFS1
isotig06428	6.125	4.35	4.861	4.165	3179	0.417787631	isogroup00534	ENSG00000244005	ENST00000374092	NFS1
isotig06429	4.956	3.677	4.074	3.456	3137	0.370876231	isogroup00534	ENSG00000244005	ENST00000374092	NFS1
isotig06430	6.248	4.432	4.958	4.244	3106	0.420446006	isogroup00534	ENSG00000244005	ENST00000374092	NFS1
isotig06431	5.464	4.165	4.565	3.884	2786	0.355968663	isogroup00534	ENSG00000244005	ENST00000374092	NFS1
isotig06432	6.926	5.023	5.567	4.777	2755	0.412395732	isogroup00534	ENSG00000244005	ENST00000374092	NFS1
isotig06433	5.588	4.255	4.669	3.967	2713	0.359068535	isogroup00534	ENSG00000244005	ENST00000374092	NFS1
isotig06434	7.091	5.137	5.699	4.886	2682	0.414302623	isogroup00534	ENSG00000244005	ENST00000374092	NFS1
isotig06435	1.364	1.386	1.715	1.584	1085	0.029392425	isogroup00534	ENSG00000244005	ENST00000540053	NFS1
isotig06436	9.518	5.331	6.051	4.944	571	0.585904035	isogroup00534	ENSG00000125995	ENST00000397416	ROMO1
isotig06437	10.785	5.99	6.833	5.552	498	0.603075449	isogroup00534	ENSG00000125995	ENST00000397416	ROMO1
isotig06443	1.893	1.143	1.57	1.004	1555	0.369476591	isogroup00535	ENSG00000175575	ENST00000310571	PAAF1
isotig06450	13.687	8.976	7.708	7.543	7985	0.470372736	isogroup00536	ENSG00000112531	ENST00000453779	QKI
isotig06452	14.572	9.221	8.384	8.078	7193	0.503588337	isogroup00536	ENSG00000112531	ENST00000361752	QKI
isotig06454	8.121	6.724	2.201	3.657	2241	0.032651988	isogroup00536	ENSG00000112531	ENST00000453779	QKI
isotig06456	8.018	6.942	1.994	3.484	1631	0.045878109	isogroup00536	ENSG00000112531	ENST00000275262	QKI
isotig06458	9.473	6.708	2.551	4.188	1449	0.125544826	isogroup00536	ENSG00000112531	ENST00000361752	QKI
isotig06460	6.512	4.823	1.982	3.027	598	0.101177951	isogroup00536	ENSG00000112531	ENST00000453779	QKI
isotig06461	3.27	2	3.117	2.361	3843	0.464501766	isogroup00537	ENSG00000133226	ENST00000447431	SRRM1
isotig06463	3.177	1.972	3.032	2.307	3753	0.453182536	isogroup00537	ENSG00000133226	ENST00000537199	SRRM1
isotig06464	3.315	2.031	3.249	2.45	3713	0.470213046	isogroup00537	ENSG00000133226	ENST00000447431	SRRM1
isotig06467	3.219	2.003	3.164	2.396	3623	0.459823777	isogroup00537	ENSG00000133226	ENST00000447431	SRRM1
isotig06469	2.64	3.909	3.007	3.802	4597	0.37513151	isogroup00538	ENSG00000198276	ENST00000369908	UCKL1
isotig06470	2.732	4.065	3.128	3.93	4512	0.381998196	isogroup00538	ENSG00000198276	ENST00000369908	UCKL1

isotig06471	3.046	4.389	3.579	4.53	3575	0.378663485	isogroup00538	ENSG00000198276	ENST00000369908	UCKL1
isotig06472	3.174	4.603	3.75	4.713	3490	0.385304727	isogroup00538	ENSG00000198276	ENST00000369908	UCKL1
isotig06473	3.345	5.02	4.233	5.475	3250	0.434686631	isogroup00538	ENSG00000237149	ENST00000425916	ZNF503-AS2
isotig06474	2.774	3.821	2.5	2.951	2494	0.313021342	isogroup00538	ENSG00000198276	ENST00000369908	UCKL1
isotig06475	2.951	4.11	2.709	3.16	2409	0.323429398	isogroup00538	ENSG00000198276	ENST00000369908	UCKL1
isotig06476	1.969	3.406	1.647	1.936	1661	0.397929661	isogroup00538	ENSG00000100441	ENST00000553935	KHNYN
isotig06477	3.852	4.927	3.539	4.128	1472	0.283666491	isogroup00538	ENSG00000198276	ENST00000369908	UCKL1
isotig06478	4.224	5.496	3.966	4.562	1387	0.303176899	isogroup00538	ENSG00000198276	ENST00000369908	UCKL1
isotig06479	4.928	6.865	5.382	6.69	1147	0.42762268	isogroup00538	ENSG00000100399	ENST00000417999	CHADL
isotig06480	4.951	4.153	6.149	4.264	3272	0.446381604	isogroup00539	ENSG00000012822	ENST00000550804	CALCOCO1
isotig06481	5.335	4.447	6.655	4.605	3047	0.461486436	isogroup00539	ENSG00000012822	ENST00000550804	CALCOCO1
isotig06482	4.999	4.073	6.157	4.246	3031	0.463346359	isogroup00539	ENSG00000012822	ENST00000430117	CALCOCO1
isotig06483	3.49	2.962	4.462	3.122	2920	0.418257308	isogroup00539	ENSG00000012822	ENST00000550804	CALCOCO1
isotig06484	5.42	4.386	6.707	4.615	2806	0.479287217	isogroup00539	ENSG00000012822	ENST00000430117	CALCOCO1
isotig06485	3.802	3.195	4.893	3.412	2695	0.433042759	isogroup00539	ENSG00000012822	ENST00000342760	CALCOCO1
isotig06486	3.412	2.765	4.319	2.999	2679	0.436715638	isogroup00539	ENSG00000012822	ENST00000430117	CALCOCO1
isotig06487	3.748	3.003	4.779	3.306	2454	0.457945067	isogroup00539	ENSG00000012822	ENST00000430117	CALCOCO1
isotig06488	2.833	2.586	3.557	2.585	2349	0.319324416	isogroup00539	ENSG00000012822	ENST00000550804	CALCOCO1
isotig06489	3.16	2.842	4.008	2.896	2124	0.347627189	isogroup00539	ENSG00000012822	ENST00000342760	CALCOCO1
isotig06490	2.658	2.506	3.613	2.361	1387	0.366151274	isogroup00539	ENSG00000012822	ENST00000546443	CALCOCO1
isotig06491	1.84	1.459	2.174	1.659	2203	0.278283986	isogroup00540	ENSG00000231618	ENST00000551222	PPT2
isotig06492	1.882	1.526	2.353	1.759	2048	0.286691215	isogroup00540	ENSG00000231618	ENST00000551222	PPT2
isotig06493	2.034	1.688	2.44	1.885	2025	0.273202074	isogroup00540	ENSG00000231618	ENST00000551222	PPT2
isotig06494	1.787	1.113	1.678	1.1	1915	0.337557301	isogroup00540	ENSG00000231618	ENST00000551222	PPT2
isotig06495	1.755	1.397	2.149	1.565	1887	0.26946344	isogroup00540	ENSG00000231618	ENST00000551222	PPT2
isotig06496	2.096	1.781	2.659	2.013	1870	0.284671601	isogroup00540	ENSG00000231618	ENST00000551222	PPT2
isotig06497	2.007	1.345	1.937	1.306	1737	0.339210566	isogroup00540	ENSG00000231618	ENST00000551222	PPT2
isotig06498	1.975	1.663	2.462	1.823	1709	0.263780341	isogroup00540	ENSG00000231618	ENST00000551222	PPT2
isotig06499	1.579	1.175	1.501	1.329	1488	0.19679492	isogroup00540	ENSG00000231618	ENST00000551222	PPT2
isotig06500	1.612	1.245	1.699	1.444	1333	0.207071466	isogroup00540	ENSG00000231618	ENST00000551222	PPT2
isotig06502	2.269	3.476	1.684	3.06	6350	0.557601262	isogroup00541	ENSG00000206560	ENST00000383777	ANKRD28
isotig06503	2.286	3.501	1.7	3.084	6245	0.559263921	isogroup00541	ENSG00000206560	ENST00000412318	ANKRD28
isotig06504	2.049	3.172	1.443	2.847	5988	0.552829338	isogroup00541	ENSG00000206560	ENST00000383777	ANKRD28
isotig06505	2.064	3.193	1.456	2.869	5883	0.552829338	isogroup00541	ENSG00000206560	ENST00000412318	ANKRD28
isotig06506	2.104	3.259	1.439	2.987	5222	0.571259487	isogroup00541	ENSG00000206560	ENST00000383777	ANKRD28
isotig06507	2.122	3.286	1.454	3.015	5117	0.573185166	isogroup00541	ENSG00000206560	ENST00000412318	ANKRD28
isotig06512	1.287	1.099	1.322	1.304	5004	0.068319306	isogroup00542	ENSG00000162885	ENST00000366600	B3GALNT2
isotig06513	1.279	1.13	1.332	1.328	4920	0.055318629	isogroup00542	ENSG00000162885	ENST00000366600	B3GALNT2
isotig06514	1.258	1.055	1.249	1.22	4829	0.080934846	isogroup00542	ENSG00000162885	ENST00000366600	B3GALNT2
isotig06515	1.25	1.086	1.258	1.244	4745	0.062260464	isogroup00542	ENSG00000162885	ENST00000366600	B3GALNT2
isotig06522	3.479	2.743	2.435	1.402	3938	0.34297738	isogroup00543	ENSG00000145730	ENST00000348126	PAM
isotig06523	3.436	2.747	2.437	1.403	3884	0.332682047	isogroup00543	ENSG00000145730	ENST00000348126	PAM
isotig06524	3.455	2.704	2.381	1.373	3737	0.33525588	isogroup00543	ENSG00000145730	ENST00000348126	PAM
isotig06526	3.391	2.725	2.367	1.395	3680	0.316421808	isogroup00543	ENSG00000145730	ENST00000348126	PAM
isotig06527	2.728	2.174	1.957	1.051	3128	0.301176073	isogroup00543	ENSG00000145730	ENST00000438793	PAM
isotig06528	2.66	2.169	1.952	1.046	3074	0.282567446	isogroup00543	ENSG00000145730	ENST00000438793	PAM
isotig06531	2.547	2.1	1.826	1.01	2870	0.24857218	isogroup00543	ENSG00000145730	ENST00000346918	PAM
isotig06536	1.094	1.071	1.216	1.07	2578	0.074753889	isogroup00544	ENSG00000177239	ENST00000371589	MAN1B1
isotig06542	1.518	1.283	1.256	1.201	3080	0.118668746	isogroup00545	ENSG00000136161	ENST00000430805	RCBTB2
isotig06543	1.545	1.311	1.281	1.237	3014	0.112036898	isogroup00545	ENSG00000136161	ENST00000430805	RCBTB2
isotig06544	1.544	1.294	1.265	1.216	3014	0.118668746	isogroup00545	ENSG00000136161	ENST00000452987	RCBTB2
isotig06545	1.573	1.323	1.291	1.253	2948	0.112431427	isogroup00545	ENSG00000136161	ENST00000452987	RCBTB2
isotig06546	2.158	1.737	1.558	1.575	2489	0.180158939	isogroup00545	ENSG00000136144	ENST00000378302	RCBTB1
isotig06552	2.358	1.949	2.106	1.672	3142	0.269087698	isogroup00546	ENSG00000171824	ENST00000376936	EXOSC10
isotig06553	2.609	2.194	2.368	1.858	2938	0.271248215	isogroup00546	ENSG00000171824	ENST00000376936	EXOSC10
isotig06554	2.479	2.094	2.245	1.78	2577	0.269782821	isogroup00546	ENSG00000171824	ENST00000376936	EXOSC10
isotig06555	1.862	1.611	1.568	1.233	2354	0.17693695	isogroup00546	ENSG00000171824	ENST00000376936	EXOSC10
isotig06556	2.158	1.913	1.874	1.447	2150	0.172136845	isogroup00546	ENSG00000171824	ENST00000376936	EXOSC10
isotig06557	1.881	1.713	1.598	1.252	1789	0.151978282	isogroup00546	ENSG00000171824	ENST00000376936	EXOSC10
isotig06558	2.693	2.586	2.304	2.133	1763	0.088308785	isogroup00546	ENSG00000171824	ENST00000544779	EXOSC10
isotig06560	1.887	1.259	1.744	1.14	1275	0.346716014	isogroup00546	ENSG00000171824	ENST00000376936	EXOSC10
isotig06561	3	1.419	2.366	1.485	396	0.527992786	isogroup00546	ENSG00000171824	ENST00000376936	EXOSC10
isotig06562	9.409	8.991	9.938	5.91	1274	0.510774404	isogroup00547	ENSG00000105379	ENST00000309244	ETFB
isotig06563	18.893	15.987	17.343	10.132	1183	0.6169873	isogroup00547	ENSG00000105379	ENST00000309244	ETFB
isotig06564	14.42	13.208	13.646	7.99	1151	0.541434959	isogroup00547	ENSG00000105379	ENST00000309244	ETFB
isotig06565	2.373	2.532	2.83	2.213	1115	0.16059217	isogroup00547	ENSG00000188155	ENST00000400368	KRTAP10-6
isotig06566	12.705	10.04	10.753	6.763	1024	0.595729691	isogroup00547	ENSG00000105379	ENST00000354232	ETFB
isotig06567	7.315	6.623	6.251	4.168	992	0.401085895	isogroup00547	ENSG00000105379	ENST00000354232	ETFB
isotig06568	30.551	27.862	28.725	17.06	927	0.566487563	isogroup00547	ENSG00000105379	ENST00000309244	ETFB
isotig06569	37.252	33.34	35.025	21.134	886	0.579638536	isogroup00547	ENSG00000105379	ENST00000309244	ETFB
isotig06570	24.715	22.391	22.296	14.001	768	0.522149996	isogroup00547	ENSG00000105379	ENST00000354232	ETFB
isotig06571	32.55	28.758	29.611	18.794	727	0.557601262	isogroup00547	ENSG00000105379	ENST00000354232	ETFB
isotig06577	12.158	8.462	9.643	8.069	925	0.527945818	isogroup00548	ENSG00000232119	ENST00000371315	MCTS1
isotig06578	12.452	8.673	9.877	8.328	928	0.529073044	isogroup00548	ENSG00000232119	ENST00000371317	MCTS1
isotig06580	13.071	9.098	10.362	8.734	884	0.531909897	isogroup00548	ENSG00000232119	ENST00000371317	MCTS1
isotig06581	12.003	8.641	9.288	7.674	337	0.535235215	isogroup00548	ENSG00000232119	ENST00000371317	MCTS1
isotig06591	129.404	121.582	135.329	103.63	581	0.378419253	isogroup00550	ENSG00000130770	ENST00000335514	ATPIF1
isotig06594	147.213	137.742	153.213	117.728	508	0.378945292	isogroup00550	ENSG00000130770	ENST00000335514	ATPIF1
isotig06596	160.534	149.783	167.13	128.009	461	0.392481401	isogroup00550	ENSG00000130770	ENST00000335514	ATPIF1
contig10148	28.201	25.244	27.475	20.017	2201	0.473998647	isogroup00551	ENSG00000112245	ENST00000370651	PTP4A1
isotig06599	2081.099	2336.207	1989.948	1986.397	1148	0.171516871	isogroup00552	ENSG00000022267	ENST00000370683	FHL1
contig10168	5.515	4.718	7.237	5.154	1416	0.310325393	isogroup00553	ENSG00000146063	ENST00000438174	TRIM41

isotig06609	4.484	7.166	7.033	7.718	4192	0.436415045	isogroup00556	ENSG00000121671	ENST00000443527	CRY2
isotig06610	5.392	8.816	8.493	9.505	3981	0.478996017	isogroup00556	ENSG00000121671	ENST00000443527	CRY2
isotig06611	4.948	8.061	7.865	8.715	3833	0.464708424	isogroup00556	ENSG00000121671	ENST00000443527	CRY2
isotig06612	4.722	7.584	7.54	8.161	3794	0.440858195	isogroup00556	ENSG00000121671	ENST00000443527	CRY2
isotig06613	4.845	7.96	7.808	8.553	3600	0.462519726	isogroup00556	ENSG00000121671	ENST00000443527	CRY2
isotig06614	1.181	1.201	1.273	1.274	2014	0.010060494	isogroup00556	ENSG00000121671	ENST00000443527	CRY2
isotig06615	2.797	4.145	3.822	4.465	1803	0.386347411	isogroup00556	ENSG00000121671	ENST00000443527	CRY2
isotig06616	1.539	1.979	1.949	2.185	1655	0.212021868	isogroup00556	ENSG00000121671	ENST00000443527	CRY2
isotig06618	2.744	2.092	2.541	2.037	4135	0.281637484	isogroup00557	ENSG00000151923	ENST00000369092	TIAL1
isotig06619	2.774	2.133	2.577	2.093	4084	0.275710153	isogroup00557	ENSG00000151923	ENST00000369092	TIAL1
isotig06620	2.755	2.066	2.534	2.02	4039	0.290598933	isogroup00557	ENSG00000151923	ENST00000369092	TIAL1
isotig06621	2.786	2.108	2.57	2.077	3988	0.284821898	isogroup00557	ENSG00000151923	ENST00000369092	TIAL1
isotig06622	2.423	2.004	2.299	1.811	1298	0.254724957	isogroup00557	ENSG00000151923	ENST00000369092	TIAL1
isotig06623	2.51	2.135	2.404	1.984	1247	0.236510859	isogroup00557	ENSG00000151923	ENST00000369092	TIAL1
isotig06624	2.434	1.91	2.256	1.737	1202	0.284671601	isogroup00557	ENSG00000151923	ENST00000369092	TIAL1
isotig06625	2.529	2.049	2.368	1.921	1151	0.267781994	isogroup00557	ENSG00000151923	ENST00000369092	TIAL1
isotig06626	2.059	2.086	2.193	1.723	3301	0.111369956	isogroup00558	ENSG00000086475	ENST00000327347	SEPHS1
isotig06627	2.05	2.067	2.192	1.743	3292	0.108908845	isogroup00558	ENSG00000086475	ENST00000327347	SEPHS1
isotig06628	2.22	2.216	2.358	1.837	3193	0.125901781	isogroup00558	ENSG00000086475	ENST00000327347	SEPHS1
isotig06629	2.21	2.197	2.357	1.858	3184	0.119720824	isogroup00558	ENSG00000086475	ENST00000327347	SEPHS1
isotig06630	2.482	2.234	2.452	1.914	1962	0.211307958	isogroup00558	ENSG00000086475	ENST00000327347	SEPHS1
isotig06631	2.468	2.203	2.451	1.949	1953	0.20750357	isogroup00558	ENSG00000086475	ENST00000327347	SEPHS1
isotig06632	2.783	2.467	2.751	2.122	1854	0.230771399	isogroup00558	ENSG00000086475	ENST00000327347	SEPHS1
isotig06633	2.77	2.435	2.752	2.16	1845	0.230771399	isogroup00558	ENSG00000086475	ENST00000327347	SEPHS1
isotig06643	5.504	4.519	6.266	5.134	3015	0.358608251	isogroup00560	ENSG00000149182	ENST00000524782	ARFGAP2
isotig06644	5.695	4.768	6.584	5.419	2935	0.342385587	isogroup00560	ENSG00000149182	ENST00000524782	ARFGAP2
isotig06645	5.969	4.98	6.787	5.712	2855	0.323927256	isogroup00560	ENSG00000149182	ENST00000524782	ARFGAP2
isotig06646	6.184	5.256	7.138	6.03	2775	0.314289472	isogroup00560	ENSG00000149182	ENST00000524782	ARFGAP2
isotig06647	5.694	4.156	6.167	4.626	2236	0.492353648	isogroup00560	ENSG00000149182	ENST00000524782	ARFGAP2
isotig06648	6.347	4.76	6.875	5.381	2076	0.463036372	isogroup00560	ENSG00000149182	ENST00000524782	ARFGAP2
isotig06649	4.972	3.521	5.014	3.943	1730	0.473651086	isogroup00560	ENSG00000149182	ENST00000524782	ARFGAP2
isotig06650	5.286	3.915	5.519	4.392	1650	0.457729015	isogroup00560	ENSG00000149182	ENST00000524782	ARFGAP2
isotig06651	2.551	1.853	2.56	2.194	577	0.280585406	isogroup00560	ENSG00000149182	ENST00000524586	ARFGAP2
isotig06661	7.634	5.132	5.441	5.231	2361	0.443535357	isogroup00562	ENSG00000129245	ENST00000250113	FXR2
isotig06662	8.404	5.687	6.062	5.811	2232	0.444005035	isogroup00562	ENSG00000129245	ENST00000250113	FXR2
isotig06663	8.864	6.101	6.369	6.202	2066	0.420295709	isogroup00562	ENSG00000129245	ENST00000250113	FXR2
isotig06664	9.832	6.805	7.146	6.936	1937	0.427838731	isogroup00562	ENSG00000129245	ENST00000250113	FXR2
isotig06665	6.007	3.956	4.437	4.079	1667	0.454234613	isogroup00562	ENSG00000129245	ENST00000250113	FXR2
isotig06666	6.986	4.664	5.254	4.825	1538	0.464285714	isogroup00562	ENSG00000129245	ENST00000250113	FXR2
isotig06667	7.507	5.163	5.619	5.295	1372	0.426410912	isogroup00562	ENSG00000129245	ENST00000250113	FXR2
isotig06668	8.874	6.163	6.751	6.344	1243	0.439261291	isogroup00562	ENSG00000129245	ENST00000250113	FXR2
isotig06669	5.552	3.706	3.889	3.713	943	0.409136169	isogroup00562	ENSG00000129245	ENST00000250113	FXR2
isotig06670	8.934	9.74	9.647	5.739	2922	0.42317953	isogroup00563	ENSG00000212857	ENST00000391545	AL356585.1
isotig06671	4.537	7.441	4.971	3.935	1653	0.369476591	isogroup00563	ENSG00000148120	ENST00000478473	C9orf3
isotig06672	7.179	10.847	7.405	6.31	1566	0.366076125	isogroup00563	ENSG00000148120	ENST00000478473	C9orf3
isotig06673	1.722	2.149	1.723	1.018	1662	0.127789885	isogroup00563	ENSG00000148120	ENST00000478473	C9orf3
isotig06674	4.194	5.244	3.964	3.218	1575	0.065830014	isogroup00563	ENSG00000148120	ENST00000478473	C9orf3
isotig06675	5.353	8.242	5.781	4.593	1456	0.324612986	isogroup00563	ENSG00000148120	ENST00000478473	C9orf3
isotig06676	6.941	10.049	7.189	6.013	1421	0.302528744	isogroup00563	ENSG00000148120	ENST00000478473	C9orf3
isotig06677	2.155	2.233	2.091	1.28	1465	0.259694146	isogroup00563	ENSG00000148120	ENST00000478473	C9orf3
isotig06679	6.184	3.141	5.714	5.174	1849	0.627263846	isogroup00564	ENSG00000105808	ENST00000449970	RASA4
isotig06680	6.775	3.452	6.278	5.747	1786	0.627658375	isogroup00564	ENSG00000105808	ENST00000449970	RASA4
isotig06681	6.569	3.325	6.102	5.497	1698	0.635051101	isogroup00564	ENSG00000105808	ENST00000262940	RASA4
isotig06682	7.23	3.672	6.733	6.136	1635	0.635755617	isogroup00564	ENSG00000105808	ENST00000262940	RASA4
isotig06683	6.852	3.334	6.298	5.51	1294	0.664706545	isogroup00564	ENSG00000105808	ENST00000541884	RASA4
isotig06684	7.744	3.796	7.145	6.359	1231	0.663447809	isogroup00564	ENSG00000105808	ENST00000541884	RASA4
isotig06685	4.271	1.445	3.137	2.357	1072	0.717592245	isogroup00564	ENSG00000170667	ENST00000541662	RASA4B
isotig06686	4.668	1.506	3.431	2.493	921	0.744241752	isogroup00564	ENSG00000170667	ENST00000465829	RASA4B
isotig06688	33.345	6.231	33.203	19.955	1479	0.942689938	isogroup00565	ENSG00000102317	ENST00000376759	RBM3
isotig06689	25.119	5.704	25.626	15.532	1480	0.921019013	isogroup00565	ENSG00000102317	ENST00000430348	RBM3
isotig06692	36.335	6.819	36.344	21.87	1339	0.943798377	isogroup00565	ENSG00000102317	ENST00000376759	RBM3
isotig06694	16.294	3.125	15.441	9.709	1209	0.915251371	isogroup00565	ENSG00000102317	ENST00000376759	RBM3
isotig06695	6.247	2.483	6.188	4.307	1210	0.720814233	isogroup00565	ENSG00000102317	ENST00000430348	RBM3
isotig06696	17.807	3.455	17.049	10.766	1069	0.919271812	isogroup00565	ENSG00000102317	ENST00000376759	RBM3
isotig06705	8.191	6.203	8.122	7.833	7624	0.25171902	isogroup00567	ENSG00000070808	ENST00000398376	CAMK2A
isotig06707	7.908	5.929	7.759	7.554	7584	0.250779665	isogroup00567	ENSG00000070808	ENST00000398376	CAMK2A
isotig06708	5.665	4.383	5.151	5.452	5487	0.146642744	isogroup00567	ENSG00000070808	ENST00000398376	CAMK2A
isotig06709	11.645	9.448	12.918	12.309	3841	0.183859998	isogroup00567	ENSG00000070808	ENST00000398376	CAMK2A
isotig06711	11.116	8.936	12.245	11.8	3801	0.182986398	isogroup00567	ENSG00000070808	ENST00000398376	CAMK2A
isotig06712	7.843	7.657	9.366	10.257	1704	0.150804088	isogroup00567	ENSG00000070808	ENST00000398376	CAMK2A
isotig06713	22.218	17.696	16.715	21.306	14216	0.054595326	isogroup00568	ENSG00000197102	ENST00000360184	DYNC1H1
isotig06715	4.287	3.007	1.429	2.478	1861	0.017143233	isogroup00568	ENSG00000197102	ENST00000360184	DYNC1H1
isotig06716	10.336	8.775	11.596	9.97	1082	0.26287856	isogroup00568	ENSG00000197102	ENST00000360184	DYNC1H1
isotig06717	13.88	11.176	14.487	12.776	1052	0.297230781	isogroup00568	ENSG00000197102	ENST00000360184	DYNC1H1
isotig06721	1.623	1.862	1.983	2.315	5334	0.18119223	isogroup00569	ENSG00000260968	ENST00000567584	SCRIB.1
isotig06722	1.612	2.003	2.062	2.476	5100	0.250629368	isogroup00569	ENSG00000260968	ENST00000567584	SCRIB.1
isotig06723	1.535	1.698	1.892	2.206	5010	0.159821898	isogroup00569	ENSG00000260968	ENST00000567584	SCRIB.1
isotig06724	1.488	1.847	1.867	2.317	4893	0.249943639	isogroup00569	ENSG00000260968	ENST00000567584	SCRIB.1
isotig06725	1.52	1.84	1.972	2.372	4776	0.233533103	isogroup00569	ENSG00000260968	ENST00000567584	SCRIB.1
isotig06726	1.383	1.666	1.759	2.197	4569	0.23156985	isogroup00569	ENSG00000260968	ENST00000567584	SCRIB.1
isotig06727	2.805	1.422	2.106	1.634	1336	0.480893515	isogroup00569	ENSG00000155846	ENST00000394320	PPARGC1B
isotig06728	2.887	1.817	2.301	2.075	1102	0.339868115	isogroup00569	ENSG00000155846	ENST00000394320	PPARGC1B

isotig06729	10.232	24.928	12.407	18.605	5228	0.758416623	isogroup00570	ENSG00000136383	ENST00000258888	ALPK3
isotig06730	17.375	34.337	21.563	27.706	4712	0.646097918	isogroup00570	ENSG00000136383	ENST00000258888	ALPK3
isotig06731	18.849	37.3	23.664	30.27	4318	0.648220861	isogroup00570	ENSG00000136383	ENST00000258888	ALPK3
isotig06732	5.972	16.618	5.979	9.733	3556	0.787536635	isogroup00570	ENSG00000136383	ENST00000258888	ALPK3
isotig06733	11.735	25.322	15.531	21.776	3242	0.709156835	isogroup00570	ENSG00000136383	ENST00000258888	ALPK3
isotig06734	6.564	18.457	6.905	10.996	3162	0.796131735	isogroup00570	ENSG00000136383	ENST00000258888	ALPK3
isotig06735	5.569	12.564	6.012	8.462	2505	0.659784324	isogroup00570	ENSG00000136383	ENST00000258888	ALPK3
isotig06736	6.382	14.561	7.405	10.115	2111	0.673217104	isogroup00570	ENSG00000136383	ENST00000258888	ALPK3
isotig06737	2.968	2.348	3.497	3.855	4894	0.038353874	isogroup00571	ENSG00000188037	ENST00000343257	CLCN1
isotig06738	2.989	2.369	3.56	3.917	4815	0.038353874	isogroup00571	ENSG00000188037	ENST00000343257	CLCN1
isotig06739	3.136	2.407	3.849	4.089	3749	0.014428496	isogroup00571	ENSG00000188037	ENST00000343257	CLCN1
isotig06740	1.71	1.508	3.199	3.263	2928	0.014428496	isogroup00571	ENSG00000127585	ENST00000397621	FBXL16
isotig06745	2.589	2.768	2.782	2.247	3441	0.025447133	isogroup00572	ENSG00000164985	ENST00000380733	PSIP1
isotig06746	1.967	1.827	1.513	1.4	3596	0.016354174	isogroup00572	ENSG00000164985	ENST00000380733	PSIP1
isotig06749	2.17	2.104	1.751	1.595	2159	0.003503795	isogroup00572	ENSG00000164985	ENST00000380733	PSIP1
isotig06751	1.483	1.276	1.089	1.048	1698	0.055553468	isogroup00572	ENSG00000164985	ENST00000397519	PSIP1
isotig06753	6.765	4.352	5.118	3.855	2661	0.537433306	isogroup00573	ENSG00000198791	ENST00000361272	CNOT7
isotig06754	6.796	4.359	5.11	3.837	2618	0.543388818	isogroup00573	ENSG00000198791	ENST00000361272	CNOT7
isotig06755	2.396	1.965	2.158	1.701	2961	0.229437514	isogroup00573	ENSG00000149480	ENST00000278823	MTA2
isotig06756	2.359	1.935	2.108	1.653	2918	0.225341925	isogroup00573	ENSG00000149480	ENST00000278823	MTA2
isotig06757	6.54	4.398	4.965	3.842	2361	0.496749831	isogroup00573	ENSG00000198791	ENST00000361272	CNOT7
isotig06758	1.775	1.768	1.741	1.527	2661	0.034718569	isogroup00573	ENSG00000149480	ENST00000278823	MTA2
isotig06759	7.015	5.751	5.414	5.061	3387	0.126841136	isogroup00574	ENSG00000102401	ENST00000537169	ARMCX3
isotig06760	2.543	1.707	2.18	1.663	3285	0.264052754	isogroup00574	ENSG00000126947	ENST00000372829	ARMCX1
isotig06761	2.823	1.978	2.468	1.931	2750	0.250385136	isogroup00574	ENSG00000102401	ENST00000537169	ARMCX3
isotig06763	1.812	1.668	1.863	1.298	1536	0.187316826	isogroup00574	ENSG00000126947	ENST00000372829	ARMCX1
isotig06765	2.221	2.457	2.579	1.886	1001	0.112769595	isogroup00574	ENSG00000170632	ENST00000441711	ARMC10
isotig06768	4.937	9.593	6.317	7.773	1899	0.637822199	isogroup00575	ENSG00000196333	ENST00000358740	AL158821.1
isotig06771	3.955	4.986	4.048	4.658	1328	0.344001278	isogroup00575	ENSG00000169592	ENST00000563197	INO80E
isotig06773	4.408	5.478	4.667	5.296	1221	0.332851131	isogroup00575	ENSG00000186399	ENST00000327271	RP11-382B18.2.1
isotig06775	9.88	10.808	10.477	8.498	1416	0.095560607	isogroup00576	ENSG00000109270	ENST00000499666	LAMTOR3
isotig06778	8.649	9.982	8.748	7.211	1352	0.019651311	isogroup00576	ENSG00000109270	ENST00000499666	LAMTOR3
isotig06798	3.719	4.11	3.393	2.794	2019	0.064082814	isogroup00580	ENSG00000164934	ENST00000297579	DCAF13
isotig07247	1.353	1.52	1.503	1.335	4223	0.029392425	isogroup00655	ENSG00000164347	ENST00000509430	GFM2
isotig07248	2.237	2.339	2.572	2.521	3048	0.03231382	isogroup00655	ENSG00000164347	ENST00000509430	GFM2
isotig07253	11.238	8.957	12.436	10.047	3784	0.414377771	isogroup00656	ENSG00000132388	ENST00000396981	UBE2G1
isotig07254	10.911	8.627	11.93	9.599	3764	0.411991809	isogroup00656	ENSG00000132388	ENST00000396981	UBE2G1
isotig07255	11.346	9.049	12.566	10.149	3742	0.414913204	isogroup00656	ENSG00000132388	ENST00000396981	UBE2G1
isotig07256	11.015	8.715	12.055	9.696	3722	0.412405125	isogroup00656	ENSG00000132388	ENST00000396981	UBE2G1
isotig07257	9.483	7.27	10.284	8.255	3605	0.42661757	isogroup00656	ENSG00000132388	ENST00000396981	UBE2G1
isotig07258	9.13	6.914	9.742	7.775	3585	0.42347073	isogroup00656	ENSG00000132388	ENST00000396981	UBE2G1
isotig07259	9.576	7.347	10.395	8.342	3563	0.427246938	isogroup00656	ENSG00000132388	ENST00000396981	UBE2G1
isotig07260	9.218	6.987	9.847	7.856	3543	0.424165853	isogroup00656	ENSG00000132388	ENST00000396981	UBE2G1
isotig07261	1.22	1.301	1.261	1.144	3796	0.036221538	isogroup00657	ENSG00000149930	ENST00000279394	TAOK2
isotig07262	1.166	1.244	1.237	1.116	3734	0.036221538	isogroup00657	ENSG00000149930	ENST00000279394	TAOK2
isotig07263	1.126	1.186	1.23	1.086	3265	0.022976629	isogroup00657	ENSG00000149930	ENST00000279394	TAOK2
isotig07270	2.524	1.784	2.725	2.149	4007	0.393063801	isogroup00658	ENSG00000215041	ENST00000399464	NEURL4
isotig07271	2.526	1.688	2.753	2.143	3632	0.399150823	isogroup00658	ENSG00000215041	ENST00000399464	NEURL4
isotig07272	2.254	1.533	2.204	1.783	3146	0.370209288	isogroup00658	ENSG00000215041	ENST00000399464	NEURL4
isotig07273	2.163	1.598	2.204	1.848	3115	0.325185992	isogroup00658	ENSG00000215041	ENST00000399464	NEURL4
isotig07274	2.22	1.374	2.17	1.725	2771	0.375807845	isogroup00658	ENSG00000215041	ENST00000399464	NEURL4
isotig07275	2.116	1.446	2.17	1.798	2740	0.321259487	isogroup00658	ENSG00000215041	ENST00000399464	NEURL4
isotig07276	2.961	1.875	3.549	2.391	1352	0.521285789	isogroup00658	ENSG00000215041	ENST00000399464	NEURL4
isotig07277	3.233	1.93	3.814	2.535	1203	0.573833321	isogroup00658	ENSG00000215041	ENST00000399464	NEURL4
isotig07279	3.476	3.025	2.897	2.092	3097	0.275907417	isogroup00659	ENSG00000096746	ENST00000354695	HNRNPH3
isotig07280	3.781	3.389	3.152	2.365	3024	0.237403246	isogroup00659	ENSG00000096746	ENST00000354695	HNRNPH3
isotig07281	3.154	2.634	2.644	1.812	2958	0.310701135	isogroup00659	ENSG00000096746	ENST00000441000	HNRNPH3
isotig07282	3.466	3.006	2.906	2.091	2885	0.275907417	isogroup00659	ENSG00000096746	ENST00000441000	HNRNPH3
isotig07283	3.915	3.546	3.319	2.419	2646	0.243912978	isogroup00659	ENSG00000096746	ENST00000354695	HNRNPH3
isotig07284	4.286	3.988	3.631	2.749	2573	0.19978207	isogroup00659	ENSG00000096746	ENST00000354695	HNRNPH3
isotig07285	3.561	3.114	3.045	2.106	2507	0.282623807	isogroup00659	ENSG00000096746	ENST00000441000	HNRNPH3
isotig07286	3.942	3.569	3.366	2.447	2434	0.236088149	isogroup00659	ENSG00000096746	ENST00000441000	HNRNPH3
isotig07287	4.016	8.243	4.174	5.039	2824	0.624201548	isogroup00660	ENSG00000017483	ENST00000376876	SLC38A5
isotig07288	4.191	8.573	4.367	5.279	2749	0.628738634	isogroup00660	ENSG00000017483	ENST00000376876	SLC38A5
isotig07289	4.122	8.43	4.337	5.322	2543	0.62658751	isogroup00660	ENSG00000017483	ENST00000376876	SLC38A5
isotig07290	4.32	8.803	4.557	5.599	2468	0.627808672	isogroup00660	ENSG00000017483	ENST00000376876	SLC38A5
isotig07291	3.211	6.585	2.997	3.871	1764	0.628221988	isogroup00660	ENSG00000017483	ENST00000376876	SLC38A5
isotig07292	3.46	7.048	3.258	4.21	1689	0.634478094	isogroup00660	ENSG00000017483	ENST00000376876	SLC38A5
isotig07293	3.241	6.591	3.052	4.136	1483	0.630607951	isogroup00660	ENSG00000139209	ENST00000447411	SLC38A4
isotig07294	3.54	7.146	3.369	4.558	1408	0.638235515	isogroup00660	ENSG00000139209	ENST00000447411	SLC38A4
isotig07295	3.616	6.979	3.97	4.287	1319	0.529956038	isogroup00660	ENSG00000161681	ENST00000391813	SHANK1
isotig07296	2.216	1.856	1.432	1.659	2902	0.090788683	isogroup00661	ENSG00000148337	ENST00000537314	CIZ1
isotig07297	2.112	1.638	1.414	1.48	2812	0.175941234	isogroup00661	ENSG00000101150	ENST00000346249	TPD52L2
isotig07298	2.007	1.587	1.273	1.431	2752	0.135689862	isogroup00661	ENSG00000101150	ENST00000351424	TPD52L2
isotig07299	4.409	2.706	4.142	2.527	1525	0.588205456	isogroup00661	ENSG00000101150	ENST00000352482	TPD52L2
isotig07300	4.439	2.753	4.244	2.553	1483	0.595260014	isogroup00661	ENSG00000101150	ENST00000352482	TPD52L2
isotig07301	4.306	2.654	3.99	2.477	1465	0.577647103	isogroup00661	ENSG00000101150	ENST00000217121	TPD52L2
isotig07302	2.776	2.084	2.125	1.625	1445	0.363023221	isogroup00661	ENSG00000101150	ENST00000346249	TPD52L2
isotig07303	4.335	2.702	4.092	2.503	1423	0.582362666	isogroup00661	ENSG00000101150	ENST00000351424	TPD52L2
isotig07304	2.597	2.003	1.877	1.534	1385	0.314439769	isogroup00661	ENSG00000101150	ENST00000351424	TPD52L2
isotig07305	5.775	4.533	6.254	5.342	2037	0.371195611	isogroup00662	ENSG00000101158	ENST00000460601	TH1L
isotig07306	5.671	4.404	6.149	5.258	1948	0.380457654	isogroup00662	ENSG00000101158	ENST00000460601	TH1L

isotig07307	5.375	4.131	5.696	5	1721	0.352868791	isogroup00662	ENSG00000101158	ENST00000460601	TH1L
isotig07308	4.266	3.409	4.616	3.57	1357	0.370876231	isogroup00662	ENSG00000101158	ENST00000460601	TH1L
isotig07309	3.888	3.151	4.224	3.263	1287	0.360327271	isogroup00662	ENSG00000101158	ENST00000460601	TH1L
isotig07310	4.002	3.133	4.341	3.317	1268	0.395430976	isogroup00662	ENSG00000101158	ENST00000460601	TH1L
isotig07311	3.579	2.84	3.903	2.973	1198	0.386469527	isogroup00662	ENSG00000101158	ENST00000460601	TH1L
isotig07312	3.147	2.405	3.197	2.467	1041	0.325552341	isogroup00662	ENSG00000101158	ENST00000460601	TH1L
isotig07313	2.565	1.991	2.575	1.981	971	0.284098595	isogroup00662	ENSG00000101158	ENST00000460601	TH1L
isotig07314	7.409	6.071	7.92	6.211	2012	0.357189825	isogroup00663	ENSG00000168259	ENST00000457167	DNAJC7
isotig07315	8.041	6.877	8.74	6.856	1813	0.327421658	isogroup00663	ENSG00000168259	ENST00000457167	DNAJC7
isotig07316	6.01	4.783	6.296	5.175	1828	0.331639363	isogroup00663	ENSG00000168259	ENST00000457167	DNAJC7
isotig07317	5.94	4.752	6.253	5.134	1756	0.330324265	isogroup00663	ENSG00000168259	ENST00000457167	DNAJC7
isotig07318	6.542	5.524	7.01	5.766	1629	0.293398211	isogroup00663	ENSG00000168259	ENST00000457167	DNAJC7
isotig07319	6.487	5.522	6.995	5.747	1557	0.291481927	isogroup00663	ENSG00000168259	ENST00000457167	DNAJC7
isotig07320	7.864	6.679	8.449	5.978	810	0.393533479	isogroup00663	ENSG00000168259	ENST00000457167	DNAJC7
isotig07321	3.912	3.097	3.864	2.883	626	0.344499136	isogroup00663	ENSG00000168259	ENST00000457167	DNAJC7
isotig07322	3.417	2.78	3.412	2.457	554	0.321390997	isogroup00663	ENSG00000168259	ENST00000457167	DNAJC7
contig11249	48.562	38.57	52.337	41.657	1214	0.483307658	isogroup00664	ENSG00000013275	ENST00000157812	PSMC4
isotig07331	10.238	6.531	9.412	8.303	3729	0.445244984	isogroup00669	ENSG00000108349	ENST00000394114	CASC3
isotig07332	29.573	35.14	32.619	38.313	2890	0.328267077	isogroup00669	ENSG00000139644	ENST00000552699	TMBIM6
isotig07334	16.279	18.099	18.37	20.12	2962	0.204140678	isogroup00669	ENSG00000157782	ENST00000316803	CABP1
isotig07335	25.631	31.396	27.431	33.964	2116	0.405782671	isogroup00669	ENSG00000139644	ENST00000552699	TMBIM6
isotig07337	6.288	3.909	6.04	4.485	2286	0.520506125	isogroup00669	ENSG00000108349	ENST00000394114	CASC3
isotig07338	7.765	8.449	8.313	9.477	2188	0.202618922	isogroup00669	ENSG00000108349	ENST00000418132	CASC3
isotig07339	11.723	18.322	11.018	14.471	6184	0.67032389	isogroup00670	ENSG00000205339	ENST00000379719	IPO7
isotig07341	12.98	20.989	11.893	16.352	4962	0.705662433	isogroup00670	ENSG00000205339	ENST00000379719	IPO7
isotig07342	28.029	54.343	57.796	61.656	3083	0.577750432	isogroup00670	ENSG00000116871	ENST00000373151	MAP7D1
isotig07343	3.818	3.622	3.469	2.847	3424	0.09609604	isogroup00671	ENSG00000120948	ENST00000240185	TARDBP
isotig07344	3.96	3.779	3.596	2.96	3280	0.09609604	isogroup00671	ENSG00000120948	ENST00000240185	TARDBP
isotig07345	2.158	2.405	2.32	1.638	2405	0.05275419	isogroup00671	ENSG00000120948	ENST00000473869	TARDBP
isotig07346	2.258	2.554	2.431	1.725	2261	0.043172766	isogroup00671	ENSG00000120948	ENST00000473869	TARDBP
isotig07347	2.425	1.994	1.807	1.704	1737	0.104597204	isogroup00671	ENSG00000120948	ENST00000473869	TARDBP
isotig07348	2.591	2.168	1.918	1.834	1593	0.091699857	isogroup00671	ENSG00000120948	ENST00000473869	TARDBP
isotig07351	1.898	5.813	5.542	6.246	2694	0.623590967	isogroup00672	ENSG00000161896	ENST00000451316	IP6K3
isotig07352	2.828	5.176	7.21	7.158	2687	0.294215451	isogroup00672	ENSG00000161896	ENST00000451316	IP6K3
isotig07353	2.038	6.358	6.123	6.847	2611	0.634496881	isogroup00672	ENSG00000161896	ENST00000451316	IP6K3
isotig07354	1.716	5.212	5.196	5.536	2489	0.590149921	isogroup00672	ENSG00000161896	ENST00000451316	IP6K3
isotig07355	1.86	5.782	5.814	6.163	2406	0.603601488	isogroup00672	ENSG00000161896	ENST00000451316	IP6K3
isotig07356	1.669	4.711	5.018	5	2140	0.507740287	isogroup00672	ENSG00000161896	ENST00000451316	IP6K3
isotig07357	1.837	5.358	5.734	5.713	2057	0.530688735	isogroup00672	ENSG00000161896	ENST00000451316	IP6K3
isotig07359	1.897	1.264	1.807	1.213	3979	0.368894191	isogroup00673	ENSG00000130023	ENST00000392095	C6orf70
isotig07360	1.893	1.258	1.872	1.26	3540	0.3592658	isogroup00673	ENSG00000130023	ENST00000392095	C6orf70
isotig07361	2.029	1.413	2.109	1.359	2476	0.383040881	isogroup00673	ENSG00000130023	ENST00000392095	C6orf70
isotig07367	3.273	5.41	3.945	4.173	2334	0.371965883	isogroup00674	ENSG00000167705	ENST00000301336	RILP
isotig07368	3.191	5.258	3.872	4.024	2291	0.359998497	isogroup00674	ENSG00000167705	ENST00000301336	RILP
isotig07369	3.598	6.117	4.505	4.78	2062	0.385924701	isogroup00674	ENSG00000167705	ENST00000301336	RILP
isotig07370	3.512	5.96	4.434	4.624	2019	0.381321861	isogroup00674	ENSG00000167705	ENST00000301336	RILP
isotig07371	2.841	4.51	2.755	3.116	1797	0.472505073	isogroup00674	ENSG00000086544	ENST00000263370	ITPKC
isotig07372	2.252	3.093	2.68	2.194	1661	0.119636282	isogroup00674	ENSG00000167705	ENST00000301336	RILP
isotig07373	3.203	5.305	3.301	3.749	1525	0.502648982	isogroup00674	ENSG00000167705	ENST00000301336	RILP
isotig07374	2.535	3.688	3.265	2.708	1389	0.138291876	isogroup00674	ENSG00000167705	ENST00000301336	RILP
contig11352	4.84	4.224	5.466	4.223	2645	0.356973773	isogroup00675	ENSG00000005469	ENST00000419147	CROT
isotig07375	8.962	6.171	7.171	5.573	6497	0.505927331	isogroup00675	ENSG00000135316	ENST00000369622	SYNCRIP
isotig07376	4.487	4.594	2.472	3.111	2637	0.208076576	isogroup00675	ENSG00000135316	ENST00000369622	SYNCRIP
isotig07377	4.54	2.592	5.966	6.017	2184	0.369401443	isogroup00676	ENSG00000067365	ENST00000381920	METTL22
isotig07379	5.106	2.913	6.638	6.698	2113	0.379715563	isogroup00676	ENSG00000067365	ENST00000561758	METTL22
isotig07381	7.338	4.133	9.668	10.138	992	0.386788908	isogroup00676	ENSG00000067365	ENST00000381920	METTL22
isotig07382	8.85	4.989	11.496	12.017	921	0.406618697	isogroup00676	ENSG00000067365	ENST00000561758	METTL22
isotig07383	9.137	5.207	12.163	12.817	847	0.387484031	isogroup00676	ENSG00000067365	ENST00000381920	METTL22
isotig07384	11.097	6.32	14.561	15.293	776	0.405782671	isogroup00676	ENSG00000067365	ENST00000561758	METTL22
isotig07393	60.01	72.288	77.484	63.294	2779	0.073683024	isogroup00678	ENSG00000064726	ENST00000261721	BTBD1
isotig07395	20.605	14.308	17.921	13.52	866	0.626897498	isogroup00679	ENSG00000113732	ENST00000519374	ATP6V0E1
isotig07399	14.096	10.552	12.406	9.064	736	0.571409784	isogroup00679	ENSG00000113732	ENST00000519911	ATP6V0E1
isotig07417	1.024	1.55	1.407	2.725	4936	0.304708048	isogroup00682	ENSG00000137959	ENST00000370751	IFI44L
isotig07418	1.011	1.441	1.333	2.536	4760	0.273991132	isogroup00682	ENSG00000137959	ENST00000370751	IFI44L
isotig07420	1.06	1.574	1.479	2.854	4438	0.304163222	isogroup00682	ENSG00000137959	ENST00000370751	IFI44L
isotig07421	1.048	1.454	1.4	2.648	4262	0.268195311	isogroup00682	ENSG00000137959	ENST00000370751	IFI44L
isotig07424	2.032	1.762	2.045	1.772	4640	0.205822124	isogroup00683	ENSG00000172995	ENST00000458225	ARPP21
isotig07425	2.049	1.789	2.056	1.794	4538	0.193929887	isogroup00683	ENSG00000172995	ENST00000458225	ARPP21
isotig07426	2.616	2.194	2.586	2.172	3452	0.263057038	isogroup00683	ENSG00000172995	ENST00000458225	ARPP21
isotig07427	1.664	1.693	1.711	1.606	3458	0.079507026	isogroup00683	ENSG00000172995	ENST00000458225	ARPP21
isotig07428	2.657	2.244	2.618	2.215	3350	0.250385136	isogroup00683	ENSG00000172995	ENST00000458225	ARPP21
isotig07429	2.359	2.313	2.36	2.127	2270	0.125901781	isogroup00683	ENSG00000172995	ENST00000458225	ARPP21
isotig07430	1.818	1.428	1.882	1.392	997	0.259966559	isogroup00683	ENSG00000172995	ENST00000458225	ARPP21
isotig07431	6.287	6.646	5.865	4.837	4051	0.072048546	isogroup00684	ENSG00000168036	ENST00000405570	CTNNB1
isotig07432	7.71	8.154	7.388	5.881	3633	0.103732998	isogroup00684	ENSG00000168036	ENST00000405570	CTNNB1
isotig07433	6.186	6.428	5.589	4.677	3749	0.086899752	isogroup00684	ENSG00000168036	ENST00000405570	CTNNB1
isotig07434	7.726	8.044	7.215	5.795	3331	0.129058015	isogroup00684	ENSG00000168036	ENST00000405570	CTNNB1
isotig07435	6.035	6.386	5.367	4.593	3590	0.046836252	isogroup00684	ENSG00000168036	ENST00000405570	CTNNB1
isotig07436	7.631	8.078	7.046	5.756	3172	0.090431728	isogroup00684	ENSG00000168036	ENST00000405570	CTNNB1
isotig07437	3.028	2.946	3.514	5.291	4248	0.137606147	isogroup00685	ENSG00000138496	ENST00000477522	PARP9
isotig07440	6.924	8.757	7.486	7.819	2342	0.225464041	isogroup00685	ENSG00000164751	ENST00000522527	PEX2
isotig07443	1.204	1.425	1.409	1.703	5098	0.172390471	isogroup00686	ENSG00000091490	ENST00000399878	SEL1L3

isotig07444	1.236	1.441	1.438	1.745	4943	0.166303449	isogroup00686	ENSG00000091490	ENST00000399878	SEL1L3
isotig07445	2.616	3.195	3.486	4.01	4282	0.292609153	isogroup00686	ENSG00000091490	ENST00000399878	SEL1L3
isotig07448	3.989	5.229	5.504	6.268	1688	0.388940032	isogroup00686	ENSG00000091490	ENST00000502949	SEL1L3
isotig07449	7.233	6.359	8.001	7.117	3465	0.274075674	isogroup00687	ENSG00000129353	ENST00000407327	SLC44A2
isotig07450	7.599	6.706	8.476	7.472	3371	0.265837529	isogroup00687	ENSG00000129353	ENST00000335757	SLC44A2
isotig07451	7.212	6.256	7.991	6.964	3353	0.287405125	isogroup00687	ENSG00000129353	ENST00000407327	SLC44A2
isotig07452	7.591	6.612	8.481	7.327	3259	0.289199294	isogroup00687	ENSG00000129353	ENST00000407327	SLC44A2
isotig07453	5.663	4.998	6.556	5.494	2620	0.330324265	isogroup00687	ENSG00000129353	ENST00000335757	SLC44A2
isotig07454	6.093	5.41	7.135	5.909	2526	0.325721425	isogroup00687	ENSG00000129353	ENST00000407327	SLC44A2
isotig07455	8.058	7.717	10.389	9.166	1680	0.204375517	isogroup00687	ENSG00000212747	ENST00000538706	FAM127C
isotig07456	1.574	1.406	1.811	1.675	3811	0.1161137	isogroup00688	ENSG00000158941	ENST00000389279	KIAA1967
isotig07457	1.633	1.469	1.884	1.748	3709	0.121392876	isogroup00688	ENSG00000158941	ENST00000389279	KIAA1967
isotig07458	1.614	1.452	1.889	1.742	3703	0.114948899	isogroup00688	ENSG00000158941	ENST00000389279	KIAA1967
isotig07459	1.676	1.518	1.966	1.819	3601	0.114948899	isogroup00688	ENSG00000158941	ENST00000389279	KIAA1967
isotig07460	1.525	1.294	1.756	1.536	1773	0.171817464	isogroup00688	ENSG00000158941	ENST00000520738	KIAA1967
isotig07461	1.654	1.425	1.916	1.688	1671	0.181267378	isogroup00688	ENSG00000158941	ENST00000520738	KIAA1967
isotig07462	1.552	1.247	1.686	1.426	1649	0.129903434	isogroup00688	ENSG00000158941	ENST00000389279	KIAA1967
isotig07463	3.219	3.228	3.123	2.468	3391	0.173912227	isogroup00689	ENSG00000203710	ENST00000529814	CR1
isotig07464	2.297	1.63	1.852	1.283	3298	0.328097994	isogroup00689	ENSG00000131788	ENST00000393045	PIAS3
isotig07465	3.363	3.385	3.308	2.628	3119	0.168144586	isogroup00689	ENSG00000203710	ENST00000529814	CR1
isotig07466	2.363	1.649	1.929	1.341	3026	0.335049222	isogroup00689	ENSG00000131788	ENST00000393045	PIAS3
isotig07467	4.457	2.092	3.758	2.061	2784	0.670652664	isogroup00689	ENSG00000131788	ENST00000393045	PIAS3
isotig07468	2.179	2.118	1.613	1.453	1982	0.084682874	isogroup00689	ENSG00000203710	ENST00000529814	CR1
isotig07469	2.277	2.228	1.712	1.584	1710	0.059301495	isogroup00689	ENSG00000203710	ENST00000529814	CR1
isotig07470	2.89	2.819	2.619	2.529	4713	0.084401067	isogroup00690	ENSG00000078304	ENST00000557268	PPP2R5C
isotig07471	3.035	2.908	2.646	2.474	4574	0.112901105	isogroup00690	ENSG00000078304	ENST00000334743	PPP2R5C
isotig07472	2.894	2.91	2.723	2.545	3162	0.074481476	isogroup00690	ENSG00000078304	ENST00000557268	PPP2R5C
isotig07473	2.279	2.019	1.773	1.836	2264	0.100435861	isogroup00690	ENSG00000078304	ENST00000328724	PPP2R5C
isotig07474	2.552	2.158	1.774	1.672	2125	0.168539115	isogroup00690	ENSG00000078304	ENST00000334743	PPP2R5C
isotig07475	3.364	1.341	3.546	2.306	1670	0.65115165	isogroup00690	ENSG00000078304	ENST00000556260	PPP2R5C
isotig07477	4.632	2.626	3.599	2.672	3180	0.535873976	isogroup00691	ENSG00000126858	ENST00000358365	RHOT1
isotig07479	4.615	2.624	3.551	2.675	3109	0.527250695	isogroup00691	ENSG00000126858	ENST00000354266	RHOT1
isotig07481	2.392	1.772	2.254	1.668	1436	0.301176073	isogroup00691	ENSG00000126858	ENST00000333942	RHOT1
isotig07483	4.214	3.04	5.534	4.408	3522	0.499267303	isogroup00692	ENSG00000162302	ENST00000334205	RPS6KA4
isotig07490	3.059	4.122	3.781	4.599	2187	0.455333659	isogroup00693	ENSG00000087274	ENST00000264758	ADD1
isotig07491	3.099	4.156	3.773	4.602	2094	0.456667543	isogroup00693	ENSG00000087274	ENST00000264758	ADD1
isotig07492	2.717	3.671	3.355	3.994	1996	0.430130758	isogroup00693	ENSG00000087274	ENST00000264758	ADD1
isotig07493	2.745	3.687	3.325	3.967	1903	0.431577365	isogroup00693	ENSG00000087274	ENST00000446856	ADD1
isotig07494	1.768	2.331	1.913	2.267	981	0.295887503	isogroup00693	ENSG00000087274	ENST00000514940	ADD1
isotig07495	1.727	2.225	1.698	2.029	888	0.274770797	isogroup00693	ENSG00000087274	ENST00000446856	ADD1
isotig07496	2.628	3.297	3.008	3.738	489	0.36770121	isogroup00693	ENSG00000087274	ENST00000510101	ADD1
isotig07497	11.588	19.102	13.86	15.114	1867	0.575571128	isogroup00694	ENSG00000135469	ENST00000308197	COQ10A
isotig07498	11.695	19.331	13.681	14.928	1774	0.584495003	isogroup00694	ENSG00000135469	ENST00000433805	COQ10A
isotig07499	11.212	18.417	13.418	14.535	1646	0.582804163	isogroup00694	ENSG00000135469	ENST00000553234	COQ10A
isotig07500	13.62	22.933	16.442	17.864	1634	0.599224093	isogroup00694	ENSG00000135469	ENST00000308197	COQ10A
isotig07501	13.865	23.428	16.393	17.817	1541	0.607715864	isogroup00694	ENSG00000135469	ENST00000433805	COQ10A
isotig07502	13.499	22.734	16.332	17.621	1413	0.607133464	isogroup00694	ENSG00000135469	ENST00000546544	COQ10A
isotig07516	3.016	3.422	2.459	2.187	1086	0.025447133	isogroup00697	ENSG00000105327	ENST00000449228	BBC3
isotig07517	3.039	3.962	2.434	2.151	986	0.158844969	isogroup00697	ENSG00000235878	ENST00000424538	AP001468.1
isotig07518	7.188	7.865	6.904	5.554	944	0.089144811	isogroup00697	ENSG00000105327	ENST00000449228	BBC3
isotig07519	7.707	9.023	7.4	5.911	844	0.006660029	isogroup00697	ENSG00000221961	ENST00000486799	PRR21
isotig07520	5.492	6.345	4.388	3.484	829	0.032651988	isogroup00697	ENSG00000162783	ENST00000367577	IER5
isotig07521	6.877	9.614	7.594	5.983	747	0.198053656	isogroup00697	ENSG00000099308	ENST00000262811	MAST3
isotig07522	489.349	463.845	537.787	523.735	656	0.138554896	isogroup00698	ENSG00000135390	ENST00000394349	ATP5G2
isotig07526	1.902	1.698	1.891	1.669	5545	0.126484181	isogroup00699	ENSG00000165819	ENST00000298717	METTL3
isotig07527	1.907	1.658	1.883	1.642	5357	0.155397535	isogroup00699	ENSG00000092203	ENST00000545559	TOX4
isotig07528	2.026	1.857	1.969	1.78	5105	0.106814083	isogroup00699	ENSG00000165819	ENST00000298717	METTL3
isotig07529	2.033	1.812	1.963	1.755	4917	0.136967386	isogroup00699	ENSG00000092203	ENST00000545559	TOX4
isotig07530	2.142	1.784	2.032	1.797	3893	0.164396558	isogroup00699	ENSG00000092203	ENST00000545559	TOX4
isotig07531	2.269	1.891	2.163	1.919	3622	0.172869542	isogroup00699	ENSG00000092203	ENST00000545559	TOX4
isotig07532	5.177	4.62	4.43	3.929	5805	0.117710603	isogroup00700	ENSG00000129116	ENST00000505667	PALLD
isotig07533	4.987	4.522	4.376	3.802	5759	0.116282783	isogroup00700	ENSG00000129116	ENST00000505667	PALLD
isotig07534	5.517	5.099	5.439	4.252	3792	0.170699632	isogroup00700	ENSG00000129116	ENST00000335742	PALLD
isotig07535	5.229	4.953	5.369	4.061	3746	0.169685128	isogroup00700	ENSG00000129116	ENST00000507735	PALLD
isotig07536	3.943	3.393	2.547	3.032	2818	0.025447133	isogroup00700	ENSG00000129116	ENST00000505667	PALLD
isotig07537	2.498	6.524	2.558	3.787	5663	0.776950101	isogroup00701	ENSG00000114770	ENST00000334444	ABCC5
isotig07538	2.496	6.485	2.54	3.728	5602	0.772807545	isogroup00701	ENSG00000114770	ENST00000334444	ABCC5
isotig07539	5.049	5.334	4.012	3.853	3479	0.143448937	isogroup00701	ENSG00000054219	ENST00000553424	LY75
isotig07541	3.574	3.669	2.684	2.823	908	0.072142481	isogroup00701	ENSG00000054219	ENST00000553424	LY75
contig11596	46.067	35.686	47.442	35.461	815	0.503804389	isogroup00702	ENSG00000174231	ENST00000304992	PRPF8
isotig07542	13.365	10.236	10.311	10.525	7328	0.304351093	isogroup00702	ENSG00000174231	ENST00000304992	PRPF8
isotig07543	13.768	10.616	10.66	10.94	7138	0.293078831	isogroup00702	ENSG00000174231	ENST00000304992	PRPF8
isotig07544	2.884	5.431	2.201	3.486	3172	0.662809048	isogroup00702	ENSG00000173588	ENST00000397809	CCDC41
isotig07545	3.951	3.858	5.094	4.466	3865	0.090957767	isogroup00703	ENSG00000236149	ENST00000457111	ABCF1
isotig07546	4.195	4.078	5.349	4.76	3779	0.092404374	isogroup00703	ENSG00000236149	ENST00000457111	ABCF1
isotig07547	4.129	4.088	5.341	4.67	3691	0.081930563	isogroup00703	ENSG00000236149	ENST00000457111	ABCF1
isotig07548	4.389	4.324	5.613	4.983	3605	0.083020215	isogroup00703	ENSG00000236149	ENST00000457111	ABCF1
isotig07549	3.741	3.558	4.388	3.995	1392	0.0314684	isogroup00703	ENSG00000236149	ENST00000457111	ABCF1
isotig07550	3.022	2.866	3.946	3.255	1315	0.155500864	isogroup00703	ENSG00000236149	ENST00000547965	ABCF1
isotig07555	1.446	1.115	4.687	2.967	1298	0.480095063	isogroup00704	ENSG00000094963	ENST00000441535	FMO2
isotig07556	2.099	1.61	6.467	3.914	875	0.552425415	isogroup00704	ENSG00000094963	ENST00000441535	FMO2
isotig07562	3.596	2.539	3.758	3.654	2916	0.226102803	isogroup00706	ENSG00000172006	ENST00000317243	ZNF554

isotig07563	1.759	1.417	1.906	1.169	2763	0.359866987	isogroup00706	ENSG00000167967	ENST00000564139	E4F1
isotig07564	1.782	1.43	1.878	1.18	2632	0.341493199	isogroup00706	ENSG00000167967	ENST00000564139	E4F1
isotig07565	1.777	1.473	1.877	1.2	2550	0.341493199	isogroup00706	ENSG00000167967	ENST00000564139	E4F1
isotig07566	1.803	1.489	1.845	1.215	2419	0.318187796	isogroup00706	ENSG00000167967	ENST00000301727	E4F1
isotig07567	1.875	1.469	1.772	1.565	2060	0.193441422	isogroup00706	ENSG00000167967	ENST00000565090	E4F1
isotig07568	4.921	2.865	3.455	2.363	2917	0.565191253	isogroup00707	ENSG00000132485	ENST00000370920	ZRANB2
isotig07569	4.727	2.735	3.318	2.24	2843	0.561941084	isogroup00707	ENSG00000132485	ENST00000370920	ZRANB2
isotig07571	4.731	2.782	2.963	2.13	2062	0.543773954	isogroup00707	ENSG00000132485	ENST00000370920	ZRANB2
isotig07572	4.446	2.593	2.747	1.945	1988	0.53189111	isogroup00707	ENSG00000132485	ENST00000370920	ZRANB2
isotig07574	2.319	1.823	2.407	1.959	3448	0.264052754	isogroup00708	ENSG00000087087	ENST00000347433	SRRT
isotig07575	2.391	1.894	2.536	2.068	3288	0.26200496	isogroup00708	ENSG00000087087	ENST00000347433	SRRT
isotig07576	2.383	1.991	2.574	2.135	3142	0.227483655	isogroup00708	ENSG00000087087	ENST00000347433	SRRT
isotig07577	2.466	2.079	2.725	2.265	2982	0.221302698	isogroup00708	ENSG00000087087	ENST00000347433	SRRT
isotig07578	1.943	1.535	1.615	1.415	1261	0.146079131	isogroup00708	ENSG00000087087	ENST00000457580	SRRT
isotig07579	1.561	1.252	1.475	1.275	659	0.202712858	isogroup00708	ENSG00000076201	ENST00000456408	PTPN23
isotig07580	3.367	1.385	2.965	1.206	3600	0.70909108	isogroup00709	ENSG00000136404	ENST00000565774	TM6SF1
isotig07581	3.256	2.105	3.548	2.335	2744	0.508472984	isogroup00709	ENSG00000136404	ENST00000322019	TM6SF1
isotig07586	40.245	580.29	173.187	158.675	3679	0.98442549	isogroup00710	ENSG00000004799	ENST00000005178	PDK4
isotig07590	14.46	16.052	18.533	13.637	2383	0.099571654	isogroup00711	ENSG00000002586	ENST00000381192	CD99
isotig07591	10.719	11.371	17.525	15.503	2889	0.053223867	isogroup00711	ENSG00000135213	ENST00000257665	POM121C
isotig07592	9.85	11.687	12.17	7.923	1981	0.067239047	isogroup00711	ENSG00000165837	ENST00000298738	FAM194B
isotig07593	21.208	20.477	32.974	27.398	2299	0.193948674	isogroup00711	ENSG00000002586	ENST00000449611	CD99
isotig07594	17.601	16.631	28.672	23.457	1929	0.236933569	isogroup00711	ENSG00000135213	ENST00000257665	POM121C
isotig07595	7.603	7.792	9.926	8.844	1048	0.133961449	isogroup00711	ENSG00000002586	ENST00000449611	CD99
isotig07596	7.515	2.985	5.494	4.163	2888	0.700467799	isogroup00712	ENSG00000136881	ENST00000395051	BAAT
isotig07597	10.054	3.157	6.968	5.149	2113	0.790880739	isogroup00712	ENSG00000105321	ENST00000504556	CCDC9
isotig07598	12.29	12.582	13.907	13.303	1470	0.08237206	isogroup00712	ENSG00000119673	ENST00000238651	ACOT2
isotig07599	4.781	2.569	3.145	2.806	1593	0.511103179	isogroup00712	ENSG00000205669	ENST00000381139	ACOT6
isotig07600	12.23	5.288	9.703	7.779	1224	0.702111671	isogroup00712	ENSG00000177465	ENST00000326303	ACOT4
isotig07601	18.891	19.943	22.083	21.022	894	0.054999249	isogroup00712	ENSG00000119673	ENST00000238651	ACOT2
isotig07602	39.226	30.93	35.739	30.258	3116	0.395637634	isogroup00713	ENSG00000112186	ENST00000229922	CAP2
isotig07603	39.873	31.444	36.342	30.782	3066	0.395787931	isogroup00713	ENSG00000112186	ENST00000229922	CAP2
isotig07604	41.2	32.384	36.633	31.391	2619	0.408704066	isogroup00713	ENSG00000112186	ENST00000229922	CAP2
isotig07605	7.804	6.312	6.924	6.074	634	0.387803412	isogroup00713	ENSG00000080573	ENST00000264828	COL5A3
isotig07606	16.233	33.251	23.032	25.219	2184	0.731814083	isogroup00714	ENSG00000124702	ENST00000432243	KLHDC3
isotig07607	17.503	36.147	24.944	27.405	2058	0.739779815	isogroup00714	ENSG00000124702	ENST00000432243	KLHDC3
isotig07608	10.319	20.755	14.57	16.375	1738	0.705549711	isogroup00714	ENSG00000124702	ENST00000432243	KLHDC3
isotig07609	11.478	23.475	16.35	18.474	1612	0.721716014	isogroup00714	ENSG00000124702	ENST00000432243	KLHDC3
isotig07610	11.006	20.115	14.306	16.166	1243	0.674879763	isogroup00714	ENSG00000124702	ENST00000394096	KLHDC3
isotig07611	19.541	37.361	25.566	26.863	897	0.698551514	isogroup00714	ENSG00000169436	ENST00000303045	COL22A1
isotig07612	5.166	3.686	5.325	3.729	1779	0.522347261	isogroup00715	ENSG00000078328	ENST00000355637	RBFOX1
isotig07613	4.746	3.43	4.874	3.409	1726	0.501709627	isogroup00715	ENSG00000078328	ENST00000311745	RBFOX1
isotig07614	6.197	5.384	6.577	5.3	1609	0.367306681	isogroup00715	ENSG00000078328	ENST00000355637	RBFOX1
isotig07615	4.318	3.46	4.218	3.228	1571	0.407548659	isogroup00715	ENSG00000078328	ENST00000436368	RBFOX1
isotig07616	5.766	5.157	6.12	4.999	1556	0.327975877	isogroup00715	ENSG00000078328	ENST00000311745	RBFOX1
isotig07617	2.938	3.591	3.657	3.245	832	0.208076576	isogroup00715	ENSG00000078328	ENST00000553186	RBFOX1
isotig07618	2.567	5.789	4.242	3.616	2858	0.531383858	isogroup00716	ENSG00000147573	ENST00000315962	TRIM55
isotig07619	2.147	4.638	3.411	2.898	2570	0.485036071	isogroup00716	ENSG00000147573	ENST00000353317	TRIM55
isotig07620	1.875	3.355	2.82	2.17	1303	0.315839408	isogroup00716	ENSG00000147573	ENST00000353317	TRIM55
isotig07621	1.42	3.951	2.302	2.447	872	0.60083039	isogroup00716	ENSG00000147573	ENST00000353317	TRIM55
isotig07629	2.275	6.132	4.059	3.639	2812	0.536850906	isogroup00718	ENSG00000005981	ENST00000325885	ASB4
isotig07630	1.196	2.579	1.606	1.303	1603	0.260811979	isogroup00718	ENSG00000005981	ENST00000325885	ASB4
isotig07631	1.244	2.704	1.67	1.363	1525	0.264898174	isogroup00718	ENSG00000005981	ENST00000325885	ASB4
isotig07637	1.858	3.903	1.402	1.94	1180	0.519191027	isogroup00719	ENSG00000095794	ENST00000462058	CREM
isotig07638	1.289	1.259	1.329	1.266	1255	0.048827685	isogroup00719	ENSG00000095794	ENST00000395895	CREM
isotig07646	12.702	14.307	8.685	10.624	1542	0.383350868	isogroup00721	ENSG00000197321	ENST00000375398	SVIL
isotig07647	9.039	10.719	6.431	9.082	1376	0.455784549	isogroup00721	ENSG00000197321	ENST00000375398	SVIL
isotig07649	9.577	11.102	6.361	8.899	1188	0.467282257	isogroup00721	ENSG00000197321	ENST00000375398	SVIL
isotig07650	9.875	11.714	6.665	9.43	1127	0.491094912	isogroup00721	ENSG00000197321	ENST00000375398	SVIL
isotig07651	6.699	6.637	10.122	11.786	1552	0.164114752	isogroup00722	ENSG00000179526	ENST00000398712	SHARPIN
isotig07652	6.031	5.911	9.198	10.483	1493	0.146032164	isogroup00722	ENSG00000179526	ENST00000398712	SHARPIN
isotig07653	6.018	5.987	9.158	10.383	1420	0.145524912	isogroup00722	ENSG00000179526	ENST00000398712	SHARPIN
isotig07654	5.255	5.162	8.103	8.892	1361	0.102474262	isogroup00722	ENSG00000179526	ENST00000398712	SHARPIN
isotig07655	5.59	5.456	8.044	9.497	388	0.129640415	isogroup00722	ENSG00000179526	ENST00000532536	SHARPIN
isotig07656	3.795	3.231	4.392	3.066	1739	0.406581123	isogroup00723	ENSG00000148803	ENST00000368552	C10orf125
isotig07657	3.297	2.519	3.578	2.532	1481	0.39647366	isogroup00723	ENSG00000183248	ENST00000539422	AC010336.1
isotig07658	2.283	1.674	2.521	1.888	820	0.327196213	isogroup00723	ENSG00000148803	ENST00000368552	C10orf125
isotig07659	2.289	1.755	2.468	1.72	744	0.378804389	isogroup00723	ENSG00000148803	ENST00000368552	C10orf125
isotig07660	2.485	1.605	2.505	1.878	721	0.356635605	isogroup00723	ENSG00000148803	ENST00000368552	C10orf125
isotig07661	2.516	1.691	2.442	1.684	645	0.418745773	isogroup00723	ENSG00000148803	ENST00000368552	C10orf125
isotig07662	2.652	2.259	1.325	1.207	1386	0.230508379	isogroup00724	ENSG00000092208	ENST00000308317	GEMIN2
isotig07663	2.734	2.309	1.367	1.228	1344	0.240503119	isogroup00724	ENSG00000092208	ENST00000308317	GEMIN2
isotig07664	3.138	2.846	1.645	1.418	1271	0.25259262	isogroup00724	ENSG00000092208	ENST00000308317	GEMIN2
isotig07665	3.246	2.919	1.701	1.448	1229	0.265612084	isogroup00724	ENSG00000092208	ENST00000308317	GEMIN2
isotig07666	9.335	8.731	11.323	8.039	1320	0.34969377	isogroup00725	ENSG00000168894	ENST00000306368	RNF181
isotig07668	8.066	7.814	9.907	7.16	1285	0.305703765	isogroup00725	ENSG00000168894	ENST00000443647	RNF181
isotig07670	1.426	1.957	2.291	1.934	1435	0.135248366	isogroup00726	ENSG00000181610	ENST00000313608	MRPS23
isotig07671	1.499	2.387	2.256	1.944	1232	0.235449388	isogroup00726	ENSG00000181610	ENST00000313608	MRPS23
isotig07672	1.805	2.617	2.853	2.524	1029	0.236773878	isogroup00726	ENSG00000181610	ENST00000313608	MRPS23
isotig07673	2.007	3.423	2.939	2.683	826	0.365089802	isogroup00726	ENSG00000181610	ENST00000313608	MRPS23
isotig07675	1.355	1.564	1.486	1.285	932	0.048414368	isogroup00727	ENSG00000153391	ENST00000441607	INO80C
isotig07677	1.114	1.332	1.027	1.112	668	0.055619223	isogroup00727	ENSG00000153391	ENST00000441607	INO80C

isotig07683	1.507	1.029	2.029	1.121	513	0.304482603	isogroup00728	ENSG00000196531	ENST00000454682	NACA
isotig07686	2.154	2.711	3.201	3.034	1664	0.112901105	isogroup00729	ENSG00000145414	ENST00000422287	NAF1
isotig07687	3.604	2.312	2.987	2.545	1051	0.375732697	isogroup00730	ENSG00000102181	ENST00000320893	CD99L2
isotig07688	3.57	2.612	3.127	2.56	927	0.349731344	isogroup00730	ENSG00000102181	ENST00000320893	CD99L2
isotig07689	3.427	2.568	3.053	2.403	750	0.356898625	isogroup00730	ENSG00000258643	ENST00000553781	BCL2L2-PABPN1
isotig07690	28.672	27.193	30.227	21.549	512	0.378691666	isogroup00731	ENSG00000134825	ENST00000537328	C11orf10
isotig07693	18.656	15.108	21.022	15.527	509	0.507045164	isogroup00732	ENSG00000102309	ENST00000373669	PIN4
isotig07695	16.909	15.418	17.46	12.811	450	0.460951003	isogroup00732	ENSG00000102309	ENST00000373669	PIN4
isotig07696	6.048	5.994	6.561	6.021	7991	0.006660029	isogroup00733	ENSG00000137497	ENST00000393695	NUMA1
isotig07697	6.337	6.361	6.78	6.294	7352	0.033563162	isogroup00733	ENSG00000137497	ENST00000393695	NUMA1
isotig07698	4.703	4.502	7.5	4.758	1465	0.488474111	isogroup00733	ENSG00000102032	ENST00000393700	RENBP
isotig07699	3.531	3.338	4.379	3.324	1165	0.211570978	isogroup00733	ENSG00000019144	ENST00000543207	PHLDB1
isotig07700	1.719	2.1	2.947	2.344	662	0.119720824	isogroup00733	ENSG00000102032	ENST00000412763	RENBP
isotig07701	10.577	12.059	7.712	8.335	7011	0.234256406	isogroup00734	ENSG00000141367	ENST00000269122	CLTC
isotig07706	4.901	3.905	5.961	5.313	3785	0.233382806	isogroup00735	ENSG00000168710	ENST00000369799	AHCYL1
isotig07707	5.502	4.32	6.708	5.661	3655	0.319033216	isogroup00735	ENSG00000168710	ENST00000369799	AHCYL1
isotig07708	3.405	2.694	4.357	3.513	2879	0.288560532	isogroup00735	ENSG00000168710	ENST00000393614	AHCYL1
isotig07709	3.24	2.577	4.102	3.388	2872	0.264935748	isogroup00735	ENSG00000168710	ENST00000393614	AHCYL1
isotig07710	3.403	2.734	4.398	3.553	2754	0.292073721	isogroup00735	ENSG00000168710	ENST00000393614	AHCYL1
isotig07711	29.186	15.128	26.332	28.415	4940	0.494936875	isogroup00736	ENSG00000198771	ENST00000367854	RCSD1
isotig07713	10.586	4.454	7.997	8.673	1248	0.620284437	isogroup00736	ENSG00000198771	ENST00000367854	RCSD1
isotig07714	2.845	3.801	4.024	4.137	1970	0.186330503	isogroup00737	ENSG00000164002	ENST00000372703	DEM1
isotig07719	96.141	110.197	113.549	98.297	1947	0.041078004	isogroup00738	ENSG00000108953	ENST00000264335	YWHAE
isotig07722	8.93	6.386	8.399	4.945	1669	0.64573157	isogroup00739	ENSG00000131876	ENST00000254193	SNRPA1
isotig07723	10.081	6.985	9.643	4.793	1364	0.718963703	isogroup00739	ENSG00000131876	ENST00000254193	SNRPA1
isotig07724	8.062	6.107	7.883	5.195	1175	0.560419328	isogroup00739	ENSG00000131876	ENST00000559309	SNRPA1
isotig07725	8.527	5.307	8.225	3.983	920	0.765076651	isogroup00739	ENSG00000131876	ENST00000559309	SNRPA1
isotig07726	44.639	55.109	47.891	41.324	2836	0.135192004	isogroup00740	ENSG00000143252	ENST00000367975	SDHC
isotig07727	88.469	110.57	96.205	81.95	1337	0.136770121	isogroup00740	ENSG00000143252	ENST00000367975	SDHC
isotig07729	60.977	99.217	89.797	91.642	1853	0.542853385	isogroup00741	ENSG00000120053	ENST00000370508	GOT1
isotig07730	37.113	61.006	56.696	55.581	1431	0.516917788	isogroup00741	ENSG00000120053	ENST00000370508	GOT1
isotig07731	17.527	26.369	24.428	25.47	1042	0.450270534	isogroup00741	ENSG00000120053	ENST00000370508	GOT1
isotig07732	37.792	26.915	33.892	26.318	2164	0.61354926	isogroup00742	ENSG00000136888	ENST00000374050	ATP6V1G1
isotig07734	21.539	11.45	32.09	8.297	1282	0.940153679	isogroup00743	ENSG00000116717	ENST00000370986	GADD45A
isotig07735	6.137	3.864	9.962	2.776	1203	0.863906215	isogroup00743	ENSG00000116717	ENST00000370985	GADD45A
isotig07737	4.608	2.302	7.712	1.918	510	0.855865334	isogroup00743	ENSG00000066933	ENST00000564571	MYO9A
isotig07742	73.806	64.357	76.08	72.794	1081	0.334213196	isogroup00745	ENSG00000100902	ENST00000261479	PSMA6
contig11992	7.577	1.379	3.612	1.322	593	0.867729391	isogroup00746	ENSG00000135451	ENST00000547923	TROAP
isotig07744	129.11	153.4	142.46	113.851	1150	0.041012249	isogroup00746	ENSG00000241837	ENST00000290299	ATP5O
isotig07745	183.008	212.958	200.256	160.905	792	0.074829037	isogroup00746	ENSG00000241837	ENST00000290299	ATP5O
isotig07746	80.953	90.795	84.764	71.661	635	0.057450966	isogroup00746	ENSG00000241837	ENST00000290299	ATP5O
isotig07747	11.773	11.004	10.337	9.504	830	0.181154655	isogroup00747	ENSG00000162961	ENST00000342166	DPY30
isotig07748	13.743	12.671	12.007	11.07	690	0.18492147	isogroup00747	ENSG00000162961	ENST00000342166	DPY30
isotig07749	12.719	11.92	10.89	10.564	598	0.168586083	isogroup00747	ENSG00000162961	ENST00000342166	DPY30
isotig07750	32.486	25.494	29.614	29.187	1983	0.298959194	isogroup00748	ENSG00000244687	ENST00000371674	UBE2V1
isotig07751	6.55	4.59	9.726	6.072	5383	0.687504697	isogroup00749	ENSG00000144791	ENST00000273317	LIMD1
isotig07753	3.172	2.31	4.216	2.97	2043	0.502949575	isogroup00749	ENSG00000144791	ENST00000273317	LIMD1
isotig07754	10.882	7.372	10.698	7.561	2601	0.604719321	isogroup00750	ENSG00000162384	ENST00000294360	C1orf123
isotig07755	9.675	6.445	9.602	6.781	2519	0.590243857	isogroup00750	ENSG00000162384	ENST00000294360	C1orf123
isotig07756	74.788	81.147	47.669	40.658	1746	0.198927256	isogroup00751	ENSG00000071282	ENST00000157600	LMCD1
isotig07757	74.6	80.852	47.586	40.395	1718	0.198946043	isogroup00751	ENSG00000071282	ENST00000157600	LMCD1
isotig07758	19.572	36.993	28.406	25.992	1602	0.665392275	isogroup00752	ENSG00000164111	ENST00000296511	ANXA5
isotig07759	9.024	15.638	11.842	11.889	821	0.615634628	isogroup00752	ENSG00000164111	ENST00000506395	ANXA5
isotig07760	85.165	41.637	61.054	50.424	832	0.763742767	isogroup00753	ENSG00000180879	ENST00000370086	SSR4
isotig07761	2.833	2.43	4.681	3.671	5513	0.266758097	isogroup00754	ENSG00000104774	ENST00000456935	MAN2B1
isotig07762	2.857	2.449	4.72	3.701	5467	0.266758097	isogroup00754	ENSG00000104774	ENST00000456935	MAN2B1
isotig07763	2.47	2.324	3.909	3.13	5019	0.173912227	isogroup00754	ENSG00000104774	ENST00000456935	MAN2B1
isotig07764	2.514	2.276	4.009	3.137	5023	0.223341099	isogroup00754	ENSG00000104774	ENST00000456935	MAN2B1
isotig07765	2.493	2.344	3.945	3.158	4973	0.173912227	isogroup00754	ENSG00000104774	ENST00000456935	MAN2B1
isotig07766	2.537	2.296	4.045	3.165	4977	0.22553919	isogroup00754	ENSG00000104774	ENST00000456935	MAN2B1
isotig07767	2.077	2.142	3.08	2.478	4529	0.09882017	isogroup00754	ENSG00000104774	ENST00000456935	MAN2B1
isotig07768	2.098	2.162	3.111	2.503	4483	0.102323965	isogroup00754	ENSG00000104774	ENST00000456935	MAN2B1
isotig07769	4.063	2.865	7.799	6.268	2854	0.381265499	isogroup00754	ENSG00000185271	ENST00000344581	KLHL33
isotig07770	4.13	2.91	7.926	6.37	2808	0.382618171	isogroup00754	ENSG00000185271	ENST00000344581	KLHL33
isotig07771	3.548	2.731	6.809	5.66	2360	0.297531374	isogroup00754	ENSG00000185271	ENST00000344581	KLHL33
isotig07772	3.64	2.63	7.016	5.67	2364	0.362027504	isogroup00754	ENSG00000185271	ENST00000344581	KLHL33
isotig07773	3.619	2.782	6.943	5.771	2314	0.29918464	isogroup00754	ENSG00000185271	ENST00000344581	KLHL33
isotig07774	3.713	2.679	7.154	5.782	2318	0.371167431	isogroup00754	ENSG00000185271	ENST00000344581	KLHL33
isotig07775	2.879	2.398	5.56	4.745	1870	0.226713384	isogroup00754	ENSG00000185271	ENST00000344581	KLHL33
isotig07776	2.951	2.455	5.7	4.864	1824	0.22826332	isogroup00754	ENSG00000185271	ENST00000344581	KLHL33
isotig07777	5.7	5.104	6.383	5.714	3813	0.285282182	isogroup00755	ENSG00000075785	ENST00000464496	RAB7A
isotig07779	5.195	4.626	5.888	5.253	3760	0.281524761	isogroup00755	ENSG00000075785	ENST00000464496	RAB7A
isotig07781	5.817	5.33	6.543	5.895	3710	0.263413993	isogroup00755	ENSG00000075785	ENST00000464496	RAB7A
isotig07783	5.299	4.842	6.037	5.424	3657	0.259092959	isogroup00755	ENSG00000075785	ENST00000464496	RAB7A
isotig07785	8.644	8.277	10.024	9.305	2270	0.191205756	isogroup00755	ENSG00000075785	ENST00000265062	RAB7A
isotig07787	7.858	7.543	9.272	8.61	2217	0.188209213	isogroup00755	ENSG00000075785	ENST00000265062	RAB7A
isotig07789	8.984	8.816	10.472	9.786	2167	0.165373488	isogroup00755	ENSG00000075785	ENST00000265062	RAB7A
isotig07791	8.168	8.06	9.694	9.069	2114	0.162001202	isogroup00755	ENSG00000075785	ENST00000265062	RAB7A
isotig07796	2.346	1.019	2.004	1.33	3642	0.509478094	isogroup00756	ENSG00000183605	ENST00000392875	SFXN4
isotig07800	2.509	1.041	2.079	1.387	3090	0.531750207	isogroup00756	ENSG00000183605	ENST00000355697	SFXN4
isotig07801	2.515	1.065	2.091	1.412	3032	0.525221688	isogroup00756	ENSG00000183605	ENST00000355697	SFXN4
isotig07802	2.686	1.129	2.244	1.501	2987	0.553101751	isogroup00756	ENSG00000183605	ENST00000355697	SFXN4

isotig07803	2.367	1.005	1.964	1.366	2966	0.499079432	isogroup00756	ENSG00000183605	ENST00000355697	SFXN4
isotig07804	2.54	1.069	2.118	1.456	2921	0.527804915	isogroup00756	ENSG00000183605	ENST00000355697	SFXN4
isotig07805	2.002	1.627	2.759	1.729	3954	0.378804389	isogroup00757	ENSG00000181038	ENST00000341249	METTL23
isotig07806	1.946	1.599	2.737	1.739	3676	0.367306681	isogroup00757	ENSG00000181038	ENST00000341249	METTL23
isotig07807	1.861	1.477	2.43	1.513	3450	0.358655219	isogroup00757	ENSG00000181038	ENST00000341249	METTL23
isotig07808	1.921	1.565	2.627	1.711	3310	0.360609078	isogroup00757	ENSG00000181038	ENST00000341249	METTL23
isotig07809	1.864	1.514	2.564	1.679	3206	0.348087473	isogroup00757	ENSG00000181038	ENST00000341249	METTL23
isotig07810	1.783	1.431	2.376	1.505	3172	0.342113174	isogroup00757	ENSG00000181038	ENST00000341249	METTL23
isotig07811	1.846	1.526	2.589	1.721	3032	0.347110543	isogroup00757	ENSG00000181038	ENST00000341249	METTL23
isotig07812	1.926	1.639	2.754	1.862	2928	0.343174645	isogroup00757	ENSG00000181038	ENST00000341249	METTL23
isotig07813	1.733	1.369	2.2	1.441	2806	0.330249117	isogroup00757	ENSG00000181038	ENST00000341249	METTL23
isotig07814	1.657	1.301	2.108	1.393	2702	0.317417525	isogroup00757	ENSG00000181038	ENST00000341249	METTL23
isotig07815	1.621	1.3	2.107	1.424	2528	0.307760953	isogroup00757	ENSG00000181038	ENST00000341249	METTL23
isotig07816	1.709	1.427	2.285	1.582	2424	0.304961674	isogroup00757	ENSG00000181038	ENST00000341249	METTL23
isotig07817	1.148	2.001	1.037	1.12	2259	0.27446081	isogroup00758	ENSG00000131373	ENST00000321169	HACL1
isotig07820	1.154	2.138	1.056	1.164	2005	0.305393778	isogroup00758	ENSG00000131373	ENST00000321169	HACL1
isotig07829	6.917	8.477	10.945	12.7	1632	0.313312542	isogroup00759	ENSG00000160408	ENST00000373146	ST6GALNAC6
isotig07831	4.904	6.136	7.83	9.176	1535	0.311161419	isogroup00759	ENSG00000160408	ENST00000373146	ST6GALNAC6
isotig07833	8.752	11.112	13.923	16.458	1156	0.359951529	isogroup00759	ENSG00000160408	ENST00000373146	ST6GALNAC6
isotig07834	9.226	11.722	14.549	17.377	1144	0.374117006	isogroup00759	ENSG00000160408	ENST00000373146	ST6GALNAC6
isotig07837	6.002	7.959	9.68	11.694	1059	0.381904261	isogroup00759	ENSG00000160408	ENST00000373146	ST6GALNAC6
isotig07838	6.488	8.59	10.315	12.644	1047	0.39686819	isogroup00759	ENSG00000160408	ENST00000373146	ST6GALNAC6
isotig07851	2.347	4.976	1.572	2.052	5547	0.638179154	isogroup00761	ENSG00000138798	ENST00000265171	EGF
isotig07852	2.323	4.918	1.544	2.009	5480	0.634121139	isogroup00761	ENSG00000138798	ENST00000503392	EGF
isotig07853	2.132	3.619	1.371	1.73	3480	0.47223266	isogroup00761	ENSG00000138798	ENST00000503392	EGF
isotig07854	2.088	3.499	1.322	1.655	3413	0.453567671	isogroup00761	ENSG00000138798	ENST00000503392	EGF
isotig07855	2.06	3.606	1.309	1.705	3409	0.485759375	isogroup00761	ENSG00000138798	ENST00000503392	EGF
isotig07856	2.014	3.484	1.259	1.627	3342	0.469348839	isogroup00761	ENSG00000138798	ENST00000503392	EGF
isotig07857	2.438	6.118	1.733	2.256	2591	0.729615992	isogroup00761	ENSG00000138798	ENST00000509793	EGF
isotig07861	6.293	5.551	5.468	4.834	2931	0.261882844	isogroup00762	ENSG00000109332	ENST00000343106	UBE2D3
isotig07862	6.092	5.322	5.261	4.713	2881	0.270562486	isogroup00762	ENSG00000109332	ENST00000453744	UBE2D3
isotig07863	6.718	5.919	5.818	5.155	2838	0.265358458	isogroup00762	ENSG00000109332	ENST00000343106	UBE2D3
isotig07864	5.654	4.883	4.914	4.205	2824	0.276771624	isogroup00762	ENSG00000109332	ENST00000343106	UBE2D3
isotig07865	6.519	5.689	5.609	5.035	2788	0.274282333	isogroup00762	ENSG00000109332	ENST00000453744	UBE2D3
isotig07866	5.434	4.633	4.688	4.067	2774	0.286259112	isogroup00762	ENSG00000109332	ENST00000453744	UBE2D3
isotig07867	6.075	5.242	5.258	4.517	2731	0.281524761	isogroup00762	ENSG00000109332	ENST00000343106	UBE2D3
isotig07868	5.855	4.99	5.031	4.38	2681	0.290176223	isogroup00762	ENSG00000109332	ENST00000453744	UBE2D3
isotig07869	4.983	4.367	4.497	3.609	2379	0.288588713	isogroup00762	ENSG00000109332	ENST00000343106	UBE2D3
isotig07870	4.707	4.059	4.219	3.432	2329	0.295699632	isogroup00762	ENSG00000109332	ENST00000507845	UBE2D3
isotig07871	6.638	3.526	4.717	4.906	2808	0.502254453	isogroup00763	ENSG00000137817	ENST00000565443	PARP6
isotig07873	6.888	3.703	4.863	5.167	2748	0.492353648	isogroup00763	ENSG00000137817	ENST00000260376	PARP6
isotig07875	6.972	3.783	5.042	5.268	2685	0.493912978	isogroup00763	ENSG00000137817	ENST00000565443	PARP6
isotig07877	7.242	3.974	5.203	5.55	2625	0.483232509	isogroup00763	ENSG00000137817	ENST00000260376	PARP6
isotig07879	3.722	1.976	2.01	2.354	1672	0.382636958	isogroup00763	ENSG00000094975	ENST00000367723	C1orf9
isotig07889	1.244	1.288	1.144	1.045	1621	0.010060494	isogroup00764	ENSG00000175395	ENST00000302609	ZNF25
isotig07891	5.796	6.096	7.559	6.05	873	0.229907192	isogroup00765	ENSG00000116586	ENST00000368302	LAMTOR2
isotig07892	4.647	4.855	5.952	4.648	832	0.19637221	isogroup00765	ENSG00000116586	ENST00000368302	LAMTOR2
isotig07893	5.65	5.743	7.055	5.688	779	0.242560307	isogroup00765	ENSG00000116586	ENST00000368302	LAMTOR2
isotig07894	4.346	4.325	5.215	4.087	738	0.215572631	isogroup00765	ENSG00000116586	ENST00000368302	LAMTOR2
isotig07895	8.385	9.167	10.855	8.937	663	0.190895769	isogroup00765	ENSG00000116586	ENST00000368305	LAMTOR2
isotig07896	7.018	7.709	8.923	7.251	622	0.146952732	isogroup00765	ENSG00000116586	ENST00000368305	LAMTOR2
isotig07897	8.612	9.193	10.708	8.919	569	0.204018562	isogroup00765	ENSG00000116586	ENST00000368305	LAMTOR2
isotig07898	7.019	7.477	8.42	6.932	528	0.159643421	isogroup00765	ENSG00000116586	ENST00000368305	LAMTOR2
isotig07899	5.253	5.296	6.654	5.942	538	0.189533704	isogroup00765	ENSG00000116586	ENST00000368305	LAMTOR2
isotig07900	3.284	3.153	3.889	3.586	497	0.115456151	isogroup00765	ENSG00000116586	ENST00000368304	LAMTOR2
isotig07901	4.572	2.712	4.421	4.453	8437	0.327712858	isogroup00766	ENSG00000196535	ENST00000527372	MYO18A
isotig07902	4.569	2.654	4.375	4.34	8427	0.352887578	isogroup00766	ENSG00000196535	ENST00000527372	MYO18A
isotig07903	4.543	2.678	4.397	4.412	8359	0.333677764	isogroup00766	ENSG00000196535	ENST00000527372	MYO18A
isotig07904	4.539	2.62	4.351	4.299	8349	0.352887578	isogroup00766	ENSG00000196535	ENST00000527372	MYO18A
isotig07905	6.257	3.617	6.403	6.528	7360	0.366743068	isogroup00766	ENSG00000196535	ENST00000527372	MYO18A
isotig07906	6.256	3.552	6.354	6.402	7350	0.394266176	isogroup00766	ENSG00000196535	ENST00000527372	MYO18A
isotig07907	6.242	3.588	6.398	6.503	7282	0.374690013	isogroup00766	ENSG00000196535	ENST00000527372	MYO18A
isotig07908	6.24	3.522	6.347	6.376	7272	0.394839182	isogroup00766	ENSG00000196535	ENST00000527372	MYO18A
isotig07909	8.64	6.051	10.53	7.949	2071	0.582296911	isogroup00766	ENSG00000196535	ENST00000546105	MYO18A
isotig07910	1.971	1.906	1.503	1.482	5285	0.016354174	isogroup00767	ENSG00000085832	ENST00000371733	EPS15
isotig07911	1.895	1.831	1.455	1.428	5174	0.021652138	isogroup00767	ENSG00000085832	ENST00000371733	EPS15
isotig07912	1.891	1.767	1.432	1.39	5091	0.029392425	isogroup00767	ENSG00000085832	ENST00000371733	EPS15
isotig07913	1.697	1.648	1.263	1.281	5155	0.016354174	isogroup00767	ENSG00000085832	ENST00000371733	EPS15
isotig07914	1.81	1.686	1.38	1.331	4980	0.034718569	isogroup00767	ENSG00000085832	ENST00000371733	EPS15
isotig07915	1.613	1.565	1.209	1.22	5044	0.016560833	isogroup00767	ENSG00000085832	ENST00000371733	EPS15
isotig07916	1.605	1.494	1.181	1.177	4961	0.029392425	isogroup00767	ENSG00000085832	ENST00000371733	EPS15
isotig07917	1.515	1.405	1.122	1.112	4850	0.029805741	isogroup00767	ENSG00000085832	ENST00000371733	EPS15
isotig07918	2.494	2.208	2.541	2.64	3387	0.119636282	isogroup00767	ENSG00000138375	ENST00000357276	SMARCAL1
isotig07919	22.775	18.431	46.689	31.47	4046	0.587500939	isogroup00768	ENSG00000166123	ENST00000340124	GPT2
isotig07920	16.585	13.297	31.675	22.399	3934	0.570151048	isogroup00768	ENSG00000166123	ENST00000340124	GPT2
isotig07922	13.189	10.649	29.062	18.099	3215	0.607715864	isogroup00768	ENSG00000166123	ENST00000562801	GPT2
isotig07923	12.809	10.208	28.509	17.423	2908	0.622191328	isogroup00768	ENSG00000181222	ENST00000322644	POLR2A
isotig07924	4.996	3.859	9.391	6.116	3103	0.543736379	isogroup00768	ENSG00000166123	ENST00000562801	GPT2
isotig07926	3.701	2.655	6.656	4.097	2796	0.563256181	isogroup00768	ENSG00000166123	ENST00000569193	GPT2
isotig07928	9.703	5.507	8.919	8.895	3822	0.379490118	isogroup00769	ENSG00000179456	ENST00000358704	ZNF238
isotig07930	9.752	5.601	8.947	8.989	3710	0.368274217	isogroup00769	ENSG00000179456	ENST00000358704	ZNF238
isotig07932	8.39	4.833	8.445	7.625	2545	0.417064327	isogroup00769	ENSG00000179456	ENST00000358704	ZNF238

isotig07934	9.444	5.334	7.621	8.864	1621	0.279852709	isogroup00769	ENSG00000179456	ENST00000358704	ZNF238
isotig07935	9.545	5.553	7.592	9.091	1509	0.241489442	isogroup00769	ENSG00000179456	ENST00000358704	ZNF238
isotig07937	2.52	5.624	9.85	12.406	4521	0.61870632	isogroup00770	ENSG00000096060	ENST00000539068	FKBP5
isotig07938	2.757	6.42	11.003	14.006	3934	0.641438716	isogroup00770	ENSG00000096060	ENST00000539068	FKBP5
isotig07939	2.796	6.582	11.268	14.271	3758	0.642669272	isogroup00770	ENSG00000096060	ENST00000539068	FKBP5
isotig07940	1.151	2.206	3.619	4.879	2159	0.51488878	isogroup00770	ENSG00000096060	ENST00000539068	FKBP5
isotig07941	1.174	2.153	3.689	4.969	2088	0.513827309	isogroup00770	ENSG00000096060	ENST00000539068	FKBP5
isotig07942	1.233	2.92	4.176	6.074	1572	0.632195461	isogroup00770	ENSG00000096060	ENST00000539068	FKBP5
isotig07943	1.27	2.881	4.299	6.256	1501	0.634590817	isogroup00770	ENSG00000096060	ENST00000539068	FKBP5
isotig07944	1.146	2.915	4.03	5.787	1396	0.629875254	isogroup00770	ENSG00000096060	ENST00000539068	FKBP5
isotig07945	1.183	2.87	4.162	5.978	1325	0.634008417	isogroup00770	ENSG00000096060	ENST00000539068	FKBP5
isotig07946	4.503	4.441	4.985	4.933	2641	0.054877132	isogroup00771	ENSG00000168872	ENST00000302243	DDX19A
isotig07949	4.077	4.015	4.499	4.364	2606	0.065830014	isogroup00771	ENSG00000157349	ENST00000563206	DDX19B
isotig07952	4.505	4.286	4.671	5.107	1773	0.007420906	isogroup00771	ENSG00000168872	ENST00000302243	DDX19A
isotig07954	3.867	3.644	3.936	4.257	1738	0.006660029	isogroup00771	ENSG00000157349	ENST00000563206	DDX19B
isotig07963	17.016	62.579	31.239	29.692	3090	0.854578417	isogroup00773	ENSG00000117289	ENST00000369317	TXNIP
isotig07964	18.141	66.361	33.161	31.502	2994	0.855686857	isogroup00773	ENSG00000117289	ENST00000369317	TXNIP
isotig07965	18.502	68.157	33.925	32.337	2947	0.856241076	isogroup00773	ENSG00000117289	ENST00000369317	TXNIP
isotig07966	19.732	72.317	36.033	34.327	2851	0.857001954	isogroup00773	ENSG00000117289	ENST00000369317	TXNIP
isotig07967	12.145	44.772	21.706	21.581	2176	0.853507552	isogroup00773	ENSG00000117289	ENST00000369317	TXNIP
isotig07968	13.538	49.395	24.032	23.812	2080	0.856071992	isogroup00773	ENSG00000117289	ENST00000369317	TXNIP
isotig07969	13.956	51.606	24.928	24.844	2033	0.85725558	isogroup00773	ENSG00000117289	ENST00000369317	TXNIP
isotig07970	15.542	56.909	27.585	27.402	1937	0.859416097	isogroup00773	ENSG00000117289	ENST00000369317	TXNIP
isotig07971	8.351	29.611	13.82	14.521	963	0.846819343	isogroup00773	ENSG00000117289	ENST00000369317	TXNIP
isotig07972	20.368	30.73	16.193	16.689	2389	0.249549109	isogroup00774	ENSG00000158022	ENST00000374272	TRIM63
isotig07973	25.227	39.476	24.535	22.728	1937	0.20821748	isogroup00774	ENSG00000158022	ENST00000374272	TRIM63
isotig07975	43.258	71.231	50.95	45.088	1789	0.198156985	isogroup00774	ENSG00000158022	ENST00000374272	TRIM63
isotig07976	28.269	44.412	29.598	26.79	1804	0.164434132	isogroup00774	ENSG00000158022	ENST00000374272	TRIM63
isotig07979	30.295	48.425	33.162	29.378	1594	0.165166829	isogroup00774	ENSG00000158022	ENST00000374272	TRIM63
isotig07981	5.424	4.22	4.591	4.608	2181	0.292064327	isogroup00775	ENSG00000224552	ENST00000546528	ABHD16A
isotig07982	5.767	4.469	4.894	4.886	2097	0.29918464	isogroup00775	ENSG00000224552	ENST00000546528	ABHD16A
isotig07983	6.175	4.826	5.2	5.236	1996	0.29347336	isogroup00775	ENSG00000224552	ENST00000546528	ABHD16A
isotig07984	6.585	5.125	5.559	5.569	1912	0.306624333	isogroup00775	ENSG00000224552	ENST00000546528	ABHD16A
isotig07985	3.568	2.577	2.967	2.602	1411	0.313021342	isogroup00775	ENSG00000236063	ENST00000549853	ABHD16A
isotig07986	3.994	2.865	3.343	2.914	1327	0.337115804	isogroup00775	ENSG00000236063	ENST00000549853	ABHD16A
isotig07987	4.512	3.315	3.715	3.323	1226	0.323607876	isogroup00775	ENSG00000236063	ENST00000549853	ABHD16A
isotig07988	5.076	3.704	4.206	3.739	1142	0.347091756	isogroup00775	ENSG00000236063	ENST00000549853	ABHD16A
isotig07989	5.938	4.611	4.849	5.191	1268	0.300133388	isogroup00775	ENSG00000224552	ENST00000546528	ABHD16A
isotig07990	8.791	7.412	9.267	8.79	2226	0.231344405	isogroup00776	ENSG00000164896	ENST00000297532	FASTK
isotig07991	8.05	6.718	8.491	8	2181	0.253005937	isogroup00776	ENSG00000164896	ENST00000297532	FASTK
isotig07992	7.727	6.512	8.352	7.629	1735	0.214107237	isogroup00776	ENSG00000164896	ENST00000353841	FASTK
isotig07993	6.744	5.593	7.325	6.578	1690	0.23497971	isogroup00776	ENSG00000164896	ENST00000353841	FASTK
isotig07994	5.32	4.394	5.301	5.285	1607	0.253785602	isogroup00776	ENSG00000164896	ENST00000483105	FASTK
isotig08397	6.53	4.609	6.504	4.61	1049	0.525644398	isogroup00835	ENSG00000099901	ENST00000402752	RANBP1
isotig08399	2.888	1.599	2.116	1.485	2058	0.517021117	isogroup00836	ENSG00000187778	ENST00000550165	MCRS1
isotig08400	2.851	1.557	2.068	1.415	1975	0.521689712	isogroup00836	ENSG00000187778	ENST00000357123	MCRS1
isotig08401	3.136	1.71	2.302	1.619	1943	0.540420455	isogroup00836	ENSG00000187778	ENST00000550165	MCRS1
isotig08402	3.108	1.67	2.259	1.551	1860	0.5460378	isogroup00836	ENSG00000187778	ENST00000550165	MCRS1
isotig08403	2.896	1.571	2.082	1.465	1694	0.527231908	isogroup00836	ENSG00000187778	ENST00000357123	MCRS1
isotig08404	3.202	1.706	2.308	1.628	1579	0.556079507	isogroup00836	ENSG00000187778	ENST00000550165	MCRS1
isotig08406	11.462	16.452	13.146	21.642	2466	0.704910949	isogroup00837	ENSG00000078061	ENST00000377045	ARAF
isotig08407	11.772	16.886	13.561	22.258	2426	0.700336289	isogroup00837	ENSG00000078061	ENST00000377045	ARAF
isotig08408	8.761	12.272	9.968	15.422	1882	0.653340347	isogroup00837	ENSG00000157764	ENST00000288602	BRAF
isotig08409	7.527	10.737	8.386	14.418	1658	0.688584955	isogroup00837	ENSG00000078061	ENST00000377045	ARAF
isotig08410	7.896	11.247	8.89	15.162	1618	0.68521267	isogroup00837	ENSG00000078061	ENST00000377045	ARAF
isotig08412	7.698	4.232	4.694	6.294	2811	0.36471406	isogroup00838	ENSG00000081052	ENST00000329662	COL4A4
isotig08413	5.077	7.367	5.112	6.62	2047	0.53318742	isogroup00838	ENSG00000063854	ENST00000564445	HAGH
isotig08414	9.575	15.568	10.5	14.747	1643	0.663861126	isogroup00838	ENSG00000063854	ENST00000564445	HAGH
isotig08415	4.908	8.72	6.144	8.195	1360	0.629537086	isogroup00838	ENSG00000063854	ENST00000564445	HAGH
isotig08416	4.518	7.713	5.324	7.149	1298	0.612487788	isogroup00838	ENSG00000063854	ENST00000564445	HAGH
isotig08417	12.567	23.385	15.839	22.827	956	0.728592094	isogroup00838	ENSG00000063854	ENST00000564445	HAGH
isotig08418	12.531	22.94	15.319	22.324	894	0.733035245	isogroup00838	ENSG00000063854	ENST00000564445	HAGH
isotig08426	8.09	5.109	3.999	3.839	1931	0.495021417	isogroup00840	ENSG00000135424	ENST00000555728	ITGA7
isotig08427	7.293	4.539	3.656	3.38	1869	0.4997088	isogroup00840	ENSG00000135424	ENST00000555728	ITGA7
isotig08428	9.027	5.75	4.282	4.319	1667	0.472138724	isogroup00840	ENSG00000135424	ENST00000555728	ITGA7
isotig08429	8.136	5.11	3.893	3.802	1605	0.478451191	isogroup00840	ENSG00000135424	ENST00000553804	ITGA7
isotig08430	11.185	6.76	5.082	5.214	1554	0.507233035	isogroup00840	ENSG00000135424	ENST00000555728	ITGA7
isotig08431	10.316	6.114	4.696	4.695	1492	0.516945968	isogroup00840	ENSG00000135424	ENST00000257879	ITGA7
isotig08432	2.863	2.63	1.404	1.547	386	0.028237018	isogroup00840	ENSG00000135424	ENST00000553804	ITGA7
isotig08440	17.955	15.094	19.638	18.72	1752	0.304398061	isogroup00842	ENSG00000105700	ENST00000539106	KXD1
isotig08442	18.829	16.068	19.256	18.403	1326	0.290364094	isogroup00842	ENSG00000105700	ENST00000539106	KXD1
isotig08446	18.377	14.962	25.25	22.72	1665	0.250882994	isogroup00843	ENSG00000116688	ENST00000444836	MFN2
isotig08447	19.35	15.78	26.495	23.901	1615	0.251080259	isogroup00843	ENSG00000116688	ENST00000444836	MFN2
isotig08448	17.798	14.706	24.034	22.348	1580	0.205305478	isogroup00843	ENSG00000116688	ENST00000444836	MFN2
isotig08449	18.806	15.562	25.308	23.583	1530	0.191741189	isogroup00843	ENSG00000116688	ENST00000444836	MFN2
isotig08458	2.267	2.359	2.421	2.91	1504	0.18420756	isogroup00845	ENSG00000169249	ENST00000307771	ZRSR2
isotig08459	2.394	2.56	2.67	3.143	1458	0.180356204	isogroup00845	ENSG00000169249	ENST00000307771	ZRSR2
isotig08460	1.379	1.679	1.595	1.977	691	0.243687533	isogroup00845	ENSG00000169249	ENST00000380308	ZRSR2
isotig08461	1.602	2.084	2.099	2.436	645	0.247031637	isogroup00845	ENSG00000169249	ENST00000307771	ZRSR2
isotig08470	4.311	2.814	4.439	3.52	7081	0.462829714	isogroup00847	ENSG00000020577	ENST00000554335	SAMD4A
isotig08471	5.569	2.844	5.104	3.464	7050	0.665570752	isogroup00847	ENSG00000020577	ENST00000554335	SAMD4A
isotig08472	4.174	2.787	4.407	3.492	6817	0.452487413	isogroup00847	ENSG00000020577	ENST00000305831	SAMD4A

isotig08473	5.481	2.818	5.097	3.434	6786	0.66789096	isogroup00847	ENSG00000020577	ENST00000305831	SAMD4A
isotig08474	4.039	3.024	4.337	2.279	6332	0.612290524	isogroup00847	ENSG00000020577	ENST00000554335	SAMD4A
isotig08475	3.873	3.003	4.297	2.194	6068	0.608758548	isogroup00847	ENSG00000020577	ENST00000305831	SAMD4A
isotig08476	1.514	1.129	1.509	1.323	9050	0.190764259	isogroup00848	ENSG00000163125	ENST00000369068	RPRD2
isotig08477	1.486	1.118	1.493	1.317	8994	0.185222064	isogroup00848	ENSG00000163125	ENST00000369068	RPRD2
isotig08478	1.497	1.085	1.471	1.277	8910	0.197001578	isogroup00848	ENSG00000163125	ENST00000369068	RPRD2
isotig08479	1.467	1.073	1.454	1.271	8854	0.191064853	isogroup00848	ENSG00000163125	ENST00000369068	RPRD2
isotig08480	3.8	3.119	3.985	3.609	1963	0.187927407	isogroup00848	ENSG00000178188	ENST00000545570	SH2B1
isotig08481	3.732	3.124	3.98	3.649	1907	0.165260765	isogroup00848	ENSG00000178188	ENST00000395532	SH2B1
contig12821	6.053	3.736	4.237	4.504	815	0.146642744	isogroup00849	ENSG00000167548	ENST00000301067	MLL2
contig12823	4.531	2.964	3.548	2.868	1124	0.465159315	isogroup00849	ENSG00000070061	ENST00000537196	IKBKAP
isotig08482	9.785	5.274	6.604	6.563	12401	0.583743518	isogroup00849	ENSG00000176542	ENST00000478658	KIAA2018
isotig08483	7.719	3.844	5.651	4.743	8002	0.635755617	isogroup00849	ENSG00000176542	ENST00000478658	KIAA2018
isotig08484	5.072	4.544	6.535	6.079	4528	0.234876381	isogroup00850	ENSG00000167323	ENST00000527651	STIM1
isotig08485	5.23	4.692	6.732	6.252	4435	0.237487788	isogroup00850	ENSG00000167323	ENST00000527651	STIM1
isotig08486	4.93	4.575	6.57	6.14	4207	0.188143458	isogroup00850	ENSG00000167323	ENST00000300737	STIM1
isotig08487	5.097	4.735	6.784	6.328	4114	0.199922973	isogroup00850	ENSG00000167323	ENST00000300737	STIM1
isotig08488	4.941	4.405	6.61	5.899	2288	0.272225145	isogroup00850	ENSG00000167323	ENST00000533977	STIM1
isotig08489	4.116	3.831	5.115	4.95	1912	0.105001127	isogroup00850	ENSG00000167323	ENST00000527651	STIM1
isotig08490	18.083	28.828	33.983	38.898	4113	0.659643421	isogroup00851	ENSG00000164591	ENST00000517768	MYOZ3
isotig08492	19.947	32.619	38.256	44.222	3961	0.675969415	isogroup00851	ENSG00000164591	ENST00000517768	MYOZ3
isotig08494	8.03	13.105	15.046	17.61	2170	0.634487488	isogroup00851	ENSG00000168542	ENST00000304636	COL3A1
isotig08495	10.931	19.362	22.006	26.458	2018	0.676805441	isogroup00851	ENSG00000164591	ENST00000517768	MYOZ3
isotig08496	5.477	5.535	6.14	5.963	3748	0.060381754	isogroup00852	ENSG00000143337	ENST00000271583	TOR1AIP1
isotig08497	5.565	5.494	6.242	6.036	3703	0.086899752	isogroup00852	ENSG00000143337	ENST00000435319	TOR1AIP1
isotig08498	4.262	4.496	4.973	4.774	3147	0.003503795	isogroup00852	ENSG00000169905	ENST00000367612	TOR1AIP2
isotig08499	3.522	3.609	3.589	3.863	2409	0.053402345	isogroup00852	ENSG00000143337	ENST00000271583	TOR1AIP1
isotig08500	3.623	3.509	3.701	3.937	2364	0.006660029	isogroup00852	ENSG00000143337	ENST00000435319	TOR1AIP1
isotig08502	1.217	4.144	1.438	2.91	4239	0.74743556	isogroup00853	ENSG00000229619	ENST00000445466	AC106722.1
isotig08503	1.164	3.066	1.278	2.166	3941	0.630993086	isogroup00853	ENSG00000229619	ENST00000445466	AC106722.1
isotig08504	1.285	3.352	1.303	2.253	3617	0.650033817	isogroup00853	ENSG00000229619	ENST00000445466	AC106722.1
isotig08508	9.136	14.51	11.29	10.32	4762	0.194296235	isogroup00854	ENSG00000176171	ENST00000368636	BNIP3
isotig08509	11.551	16.071	15.2	14.064	3429	0.053580822	isogroup00854	ENSG00000176171	ENST00000368636	BNIP3
isotig08510	9.314	16.389	11.929	10.77	3289	0.305825881	isogroup00854	ENSG00000184216	ENST00000429936	IRAK1
isotig08511	5.968	12.245	5.468	5.383	2657	0.484791839	isogroup00854	ENSG00000176171	ENST00000540159	BNIP3
isotig08512	9.035	14.007	9.732	10.11	1324	0.248243406	isogroup00854	ENSG00000176171	ENST00000368636	BNIP3
isotig08513	7.35	14.927	6.475	5.312	1014	0.375140903	isogroup00854	ENSG00000145536	ENST00000274181	ADAMTS16
isotig08514	3.93	3.111	4.209	3.605	3546	0.341042309	isogroup00855	ENSG00000132676	ENST00000368336	DAP3
isotig08515	3.775	3.037	4.071	3.491	3444	0.329290975	isogroup00855	ENSG00000132676	ENST00000421487	DAP3
isotig08516	3.696	2.906	3.84	3.134	3427	0.355583527	isogroup00855	ENSG00000132676	ENST00000535183	DAP3
isotig08517	1.896	1.439	2.011	1.736	2538	0.20753175	isogroup00855	ENSG00000132676	ENST00000535183	DAP3
isotig08518	4.476	2.948	4.527	3.721	928	0.47268355	isogroup00855	ENSG00000132676	ENST00000368336	DAP3
isotig08519	3.898	2.621	3.99	3.259	826	0.474055009	isogroup00855	ENSG00000132676	ENST00000368336	DAP3
isotig08520	4.574	3.789	5.747	4.398	3708	0.42006087	isogroup00856	ENSG00000168309	ENST00000474531	FAM107A
isotig08521	4.128	4.491	6.452	5.025	3072	0.218587961	isogroup00856	ENSG00000099625	ENST00000382477	C19orf26
isotig08522	4.618	4.355	6.711	5.258	3005	0.32393665	isogroup00856	ENSG00000168309	ENST00000447756	FAM107A
isotig08523	4.603	4.488	6.915	5.336	2982	0.314251897	isogroup00856	ENSG00000168309	ENST00000474531	FAM107A
isotig08524	2.69	1.876	2.462	1.951	898	0.43164312	isogroup00856	ENSG00000168309	ENST00000447756	FAM107A
isotig08525	2.591	2.263	3.045	2.13	875	0.392819569	isogroup00856	ENSG00000168309	ENST00000474531	FAM107A
isotig08526	12.303	17.284	13.782	9.496	3338	0.156440219	isogroup00857	ENSG00000198805	ENST00000361505	PNP
isotig08527	10.431	14.434	11.619	7.777	3319	0.111097543	isogroup00857	ENSG00000198805	ENST00000361505	PNP
isotig08528	13.223	18.707	14.745	10.232	3135	0.173282859	isogroup00857	ENSG00000198805	ENST00000361505	PNP
isotig08529	11.236	15.68	12.447	8.406	3116	0.155021793	isogroup00857	ENSG00000198805	ENST00000361505	PNP
isotig08530	10.8	13.023	10.966	7.302	1255	0.153067934	isogroup00857	ENSG00000198805	ENST00000553418	PNP
isotig08531	1.463	1.819	1.645	1.561	3421	0.127789885	isogroup00858	ENSG00000127419	ENST00000264771	TMEM175
isotig08532	1.444	1.858	1.626	1.573	3273	0.157933794	isogroup00858	ENSG00000127419	ENST00000264771	TMEM175
isotig08533	1.711	1.855	1.875	1.637	2771	0.003503795	isogroup00858	ENSG00000127419	ENST00000515740	TMEM175
isotig08534	3.893	3.161	3.176	2.846	1641	0.331601789	isogroup00858	ENSG00000213121	ENST00000392385	AL590867.1
isotig08537	6.777	4.063	6.373	4.661	3271	0.611445104	isogroup00859	ENSG00000160953	ENST00000415183	MUM1
isotig08538	7.263	4.637	7.175	5.486	2626	0.570414068	isogroup00859	ENSG00000160953	ENST00000344663	MUM1
isotig08539	6.277	3.445	5.593	3.782	2277	0.652147366	isogroup00859	ENSG00000160953	ENST00000344663	MUM1
isotig08540	7.71	3.698	6.512	3.942	1940	0.733410987	isogroup00859	ENSG00000160953	ENST00000344663	MUM1
isotig08541	6.861	4.125	6.577	4.762	1632	0.596471782	isogroup00859	ENSG00000160953	ENST00000344663	MUM1
isotig08542	6.039	3.939	5.371	4.27	1648	0.539387165	isogroup00859	ENSG00000160953	ENST00000415183	MUM1
isotig08543	3.043	6.15	5.566	4.23	3466	0.340441121	isogroup00860	ENSG00000196549	ENST00000493237	MME
isotig08544	2.863	5.773	5.187	4.003	3420	0.333226873	isogroup00860	ENSG00000196549	ENST00000493237	MME
isotig08545	2.023	3.88	3.254	2.55	2646	0.313021342	isogroup00860	ENSG00000196549	ENST00000493237	MME
isotig08546	1.768	3.344	2.715	2.222	2600	0.287865409	isogroup00860	ENSG00000196549	ENST00000493237	MME
isotig08547	1.901	3.122	3.401	2.013	760	0.13943789	isogroup00860	ENSG00000196549	ENST00000493237	MME
isotig08548	38.043	21.45	32.183	28.311	2515	0.61299504	isogroup00861	ENSG00000185624	ENST00000537205	P4HB
isotig08550	69.676	38.292	57.208	52.023	2395	0.61370895	isogroup00861	ENSG00000185624	ENST00000331483	P4HB
isotig08552	26.301	14.989	24.024	20.031	1178	0.643261066	isogroup00861	ENSG00000165238	ENST00000448251	WNK2
isotig08554	5.636	4.585	4.907	3.665	2783	0.308108514	isogroup00862	ENSG00000166913	ENST00000372839	YWHAB
isotig08555	5.761	4.725	4.973	3.709	2636	0.301072744	isogroup00862	ENSG00000166913	ENST00000372839	YWHAB
isotig08556	4.882	4.156	4.504	3.151	2345	0.289753513	isogroup00862	ENSG00000166913	ENST00000372839	YWHAB
isotig08557	4.981	4.295	4.557	3.167	2198	0.288588713	isogroup00862	ENSG00000166913	ENST00000372839	YWHAB
isotig08558	3.957	2.903	2.653	2.426	1058	0.315416698	isogroup00862	ENSG00000166913	ENST00000428262	YWHAB
isotig08559	2.379	2.666	1.663	1.824	3075	0.183258811	isogroup00863	ENSG00000072609	ENST00000432561	CHFR
isotig08560	2.269	2.536	1.618	1.797	2573	0.166209514	isogroup00863	ENSG00000072609	ENST00000432561	CHFR
isotig08561	3.114	2.934	1.833	2.083	1865	0.074481476	isogroup00863	ENSG00000072609	ENST00000432561	CHFR
isotig08562	2.161	2.608	1.687	1.754	1710	0.219226723	isogroup00863	ENSG00000072609	ENST00000537522	CHFR
isotig08563	1.699	2.109	1.366	1.207	1196	0.1161137	isogroup00863	ENSG00000072609	ENST00000432561	CHFR

isotig08564	1.835	2.305	1.601	1.668	1208	0.195461036	isogroup00863	ENSG00000072609	ENST00000537522	CHFR
isotig08565	6.652	6.819	6.193	6.538	3236	0.060381754	isogroup00864	ENSG00000106615	ENST00000262187	RHEB
isotig08566	8.447	8.935	7.832	8.382	2981	0.078163748	isogroup00864	ENSG00000106615	ENST00000262187	RHEB
isotig08567	13.792	12.437	13.101	14.078	2187	0.052660254	isogroup00864	ENSG00000102178	ENST00000369660	UBL4A
isotig08568	8.461	8.746	7.45	7.634	1624	0.019651311	isogroup00864	ENSG00000106615	ENST00000262187	RHEB
isotig08569	12.706	13.713	11.252	11.852	1369	0.036869693	isogroup00864	ENSG00000106615	ENST00000262187	RHEB
isotig08570	19.723	14.403	18.168	13.187	2321	0.54434696	isogroup00865	ENSG00000108788	ENST00000435881	MLX
isotig08571	20.631	14.824	18.861	13.673	2214	0.563528594	isogroup00865	ENSG00000108788	ENST00000346833	MLX
isotig08572	3.983	4.564	3.843	4.59	2130	0.2660254	isogroup00865	ENSG00000108788	ENST00000435881	MLX
isotig08573	4.144	4.505	3.843	4.667	2023	0.242466371	isogroup00865	ENSG00000108788	ENST00000346833	MLX
isotig08574	55.905	36.031	51.427	30.744	973	0.72524799	isogroup00865	ENSG00000169436	ENST00000545577	COL22A1
isotig08575	58.568	37.701	53.846	32.219	921	0.72599008	isogroup00865	ENSG00000169436	ENST00000545577	COL22A1
isotig08581	52.911	95.658	69.719	100.692	3809	0.76700233	isogroup00867	ENSG00000116748	ENST00000520113	AMPD1
isotig08583	23.47	39.569	27.982	43.01	939	0.738051402	isogroup00867	ENSG00000116748	ENST00000520113	AMPD1
isotig08585	9.918	8.365	9.483	8.768	2321	0.218409484	isogroup00868	ENSG00000115866	ENST00000264161	DARS
isotig08586	10.254	8.49	9.73	8.939	2279	0.246355302	isogroup00868	ENSG00000115866	ENST00000264161	DARS
isotig08587	10.768	9.076	10.332	9.59	2199	0.220945743	isogroup00868	ENSG00000115866	ENST00000264161	DARS
isotig08588	11.14	9.222	10.61	9.788	2157	0.248694296	isogroup00868	ENSG00000115866	ENST00000264161	DARS
isotig08589	4.707	3.886	4.628	3.349	1445	0.382364545	isogroup00868	ENSG00000115866	ENST00000422708	DARS
isotig08590	6.69	6.203	8.681	9.726	2589	0.006660029	isogroup00869	ENSG00000182087	ENST00000356663	C19orf6
isotig08591	7.099	6.555	9.234	10.308	2502	0.017143233	isogroup00869	ENSG00000182087	ENST00000356663	C19orf6
isotig08592	2.818	2.554	3.357	3.836	1421	0.038353874	isogroup00869	ENSG00000182087	ENST00000356663	C19orf6
isotig08593	4.702	4.222	5.748	6.452	1387	0.032651988	isogroup00869	ENSG00000182087	ENST00000356663	C19orf6
isotig08594	3.334	2.978	4.046	4.545	1334	0.061837755	isogroup00869	ENSG00000182087	ENST00000356663	C19orf6
isotig08595	5.356	4.768	6.615	7.355	1300	0.046836252	isogroup00869	ENSG00000182087	ENST00000356663	C19orf6
isotig08596	6.664	3.697	4.679	3.265	2127	0.665091681	isogroup00870	ENSG00000011304	ENST00000356948	PTBP1
isotig08597	6.469	3.515	4.529	3.053	2049	0.673705568	isogroup00870	ENSG00000011304	ENST00000394601	PTBP1
isotig08598	6.068	3.305	3.754	2.76	1853	0.642894717	isogroup00870	ENSG00000011304	ENST00000356948	PTBP1
isotig08599	5.817	3.078	3.54	2.494	1775	0.648605997	isogroup00870	ENSG00000011304	ENST00000394601	PTBP1
isotig08600	7.16	3.714	5.446	3.584	1391	0.7182404	isogroup00870	ENSG00000011304	ENST00000350092	PTBP1
isotig08601	8.186	4.178	5.783	3.992	1329	0.726394003	isogroup00870	ENSG00000011304	ENST00000394601	PTBP1
isotig08603	11.361	18.862	14.501	13.936	1769	0.540476817	isogroup00871	ENSG00000151967	ENST00000482804	SCHIP1
isotig08604	11.783	19.326	14.966	14.124	1533	0.531853536	isogroup00871	ENSG00000151967	ENST00000445224	SCHIP1
isotig08605	6.591	10.837	8.739	8.19	1329	0.506227925	isogroup00871	ENSG00000250588	ENST00000473061	IQCJ-SCHIP1
isotig08606	6.153	9.756	8.148	7.214	1093	0.461167055	isogroup00871	ENSG00000250588	ENST00000473061	IQCJ-SCHIP1
isotig08607	2.09	3.309	2.845	2.907	978	0.344818517	isogroup00871	ENSG00000143536	ENST00000271835	CRNN
isotig08608	23.919	15.058	25.003	21.506	2008	0.570987074	isogroup00872	ENSG00000125037	ENST00000245046	TMEM111
isotig08610	17.558	11.743	19.295	15.944	1779	0.533638311	isogroup00872	ENSG00000125037	ENST00000245046	TMEM111
isotig08612	11.92	13.197	11.869	11.404	2726	0.124849703	isogroup00873	ENSG00000225073	ENST00000547039	DDX39B
isotig08613	12.682	11.563	16.012	13.182	1733	0.298395581	isogroup00873	ENSG00000225073	ENST00000547039	DDX39B
isotig08614	9.865	8.828	12.031	10.415	1485	0.289180507	isogroup00873	ENSG00000230624	ENST00000456666	DDX39B
isotig08615	10.493	10.428	13.741	10.303	1239	0.266100549	isogroup00873	ENSG00000237889	ENST00000552820	DDX39B
isotig08616	5.724	6.045	7.206	5.439	991	0.167083114	isogroup00873	ENSG00000237889	ENST00000552820	DDX39B
isotig08617	6.321	6.288	7.923	7.525	1803	0.155078154	isogroup00874	ENSG00000175634	ENST00000312629	RPS6KB2
isotig08618	6.941	6.798	8.693	8.309	1773	0.15861013	isogroup00874	ENSG00000175634	ENST00000312629	RPS6KB2
isotig08619	6.938	7.102	8.823	8.433	1719	0.132636958	isogroup00874	ENSG00000175634	ENST00000312629	RPS6KB2
isotig08620	7.599	7.651	9.647	9.272	1689	0.147337867	isogroup00874	ENSG00000175634	ENST00000312629	RPS6KB2
isotig08621	4.083	3.552	4.577	3.948	636	0.194465319	isogroup00874	ENSG00000175634	ENST00000312629	RPS6KB2
isotig08622	4.766	4.146	5.25	4.576	632	0.239394679	isogroup00874	ENSG00000175634	ENST00000312629	RPS6KB2
isotig08623	13.517	10.118	11.306	9.277	2060	0.448833321	isogroup00875	ENSG00000123562	ENST00000441076	MORF4L2
isotig08624	13.874	10.606	11.752	9.751	1888	0.439017059	isogroup00875	ENSG00000123562	ENST00000441076	MORF4L2
isotig08625	14.254	10.975	12.09	10.019	1847	0.440069137	isogroup00875	ENSG00000123562	ENST00000441076	MORF4L2
isotig08628	1.286	2.522	3	3.576	2617	0.256772751	isogroup00876	ENSG00000182732	ENST00000556437	RGS6
isotig08629	1.22	2.425	2.911	3.466	2577	0.268928008	isogroup00876	ENSG00000182732	ENST00000556437	RGS6
isotig08635	6.102	4.6	3.513	10.865	1633	0.689129781	isogroup00877	ENSG00000180228	ENST00000325748	PRKRA
isotig08638	6.046	5.577	3.219	12.126	588	0.782332607	isogroup00877	ENSG00000180228	ENST00000424699	PRKRA
isotig08640	6.501	6.334	7.041	6.714	1268	0.113539866	isogroup00878	ENSG00000143575	ENST00000483970	HAX1
isotig08641	6.336	6.093	6.884	6.518	1148	0.101976403	isogroup00878	ENSG00000143575	ENST00000483970	HAX1
isotig08642	7.96	8.034	8.626	8.331	1097	0.077149245	isogroup00878	ENSG00000143575	ENST00000483970	HAX1
isotig08643	7.947	7.958	8.637	8.301	977	0.063209213	isogroup00878	ENSG00000143575	ENST00000483970	HAX1
isotig08644	5.131	4.069	6.654	5.191	905	0.460753739	isogroup00879	ENSG00000148362	ENST00000371620	C9orf142
isotig08645	4.064	3.387	5.194	4.097	831	0.375535432	isogroup00879	ENSG00000148362	ENST00000371620	C9orf142
isotig08646	6.064	4.802	7.971	6.088	825	0.502442324	isogroup00879	ENSG00000148362	ENST00000371620	C9orf142
isotig08647	4.976	4.123	6.483	4.967	751	0.433080334	isogroup00879	ENSG00000148362	ENST00000371620	C9orf142
isotig08648	4.971	4.313	6.699	4.892	584	0.434583302	isogroup00879	ENSG00000148362	ENST00000371620	C9orf142
isotig08649	4.199	3.743	5.356	4.349	522	0.320808597	isogroup00879	ENSG00000148362	ENST00000371620	C9orf142
isotig08650	260.558	278.493	261.305	218.489	1003	0.123234012	isogroup00880	ENSG00000117118	ENST00000375499	SDHB
isotig08654	14.807	10.267	11.123	8.548	1079	0.519641918	isogroup00881	ENSG00000258315	ENST00000439424	C17orf49.1
isotig08656	17.809	13.2	13.635	10.976	822	0.458001428	isogroup00881	ENSG00000258315	ENST00000439424	C17orf49.1
contig13085	26.176	22.91	17.067	17.477	1149	0.116508229	isogroup00882	ENSG00000198736	ENST00000361871	SEPX1
contig13095	2.16	2.059	2.589	2.242	749	0.060757496	isogroup00883	ENSG00000221944	ENST00000408957	TIGD1
isotig08660	226.031	142.148	156.537	151.672	827	0.54646051	isogroup00883	ENSG00000143158	ENST00000367846	BRP44
isotig08662	189.748	186.777	210.472	173.437	1073	0.232377696	isogroup00884	ENSG00000179091	ENST00000318911	CYC1
isotig08663	201.087	198.054	223.104	183.802	1012	0.232509206	isogroup00884	ENSG00000179091	ENST00000318911	CYC1
isotig08673	42.859	39.454	42.38	34.593	661	0.384318404	isogroup00887	ENSG00000183172	ENST00000331479	C22orf32
isotig08674	34.272	25.327	31.016	25.018	611	0.558700308	isogroup00887	ENSG00000183172	ENST00000422252	C22orf32
isotig08675	27.769	22.779	26.006	19.722	493	0.515273916	isogroup00887	ENSG00000183172	ENST00000331479	C22orf32
isotig08676	12.866	10.631	13.894	9.736	1137	0.452252574	isogroup00888	ENSG00000132432	ENST00000450622	SEC61G
isotig08678	836.967	544.38	531.956	428.25	540	0.634205681	isogroup00889	ENSG00000131469	ENST00000253788	RPL27
isotig08684	3.062	2.994	4.018	4.069	6708	0.051664537	isogroup00891	ENSG00000100241	ENST00000380817	SBF1
isotig08685	3.15	3.073	4.115	4.184	6639	0.051664537	isogroup00891	ENSG00000100241	ENST00000380817	SBF1
isotig08686	3.144	3.089	4.144	4.202	6623	0.049513414	isogroup00891	ENSG00000100241	ENST00000380817	SBF1

isotig08687	3.234	3.169	4.243	4.319	6554	0.049513414	isogroup00891	ENSG00000100241	ENST00000380817	SBF1
isotig08688	11.296	18.419	11.089	14.191	4498	0.66960998	isogroup00892	ENSG00000123124	ENST00000517970	WWP1
isotig08689	10.266	16.746	10.041	12.887	4393	0.664734726	isogroup00892	ENSG00000123124	ENST00000517970	WWP1
isotig08690	10.556	17.079	10.648	13.198	4313	0.652945818	isogroup00892	ENSG00000123124	ENST00000517970	WWP1
isotig08691	9.462	15.299	9.544	11.812	4208	0.64718757	isogroup00892	ENSG00000123124	ENST00000517970	WWP1
isotig08693	1.814	3.404	3.346	2.667	3811	0.285103705	isogroup00893	ENSG00000112394	ENST00000368851	SLC16A10
isotig08697	53.19	54.907	53.284	51.971	4059	0.135473811	isogroup00894	ENSG00000080824	ENST00000334701	HSP90AA1
isotig08698	51.192	52.956	51.698	50.455	4031	0.135276546	isogroup00894	ENSG00000080824	ENST00000334701	HSP90AA1
isotig08700	62.754	53.156	84.161	90.426	1045	0.023962952	isogroup00894	ENSG00000133401	ENST00000438447	PDZD2
isotig08701	1.461	1.639	1.696	1.168	3398	0.136272263	isogroup00895	ENSG00000107521	ENST00000407891	HPS1
isotig08705	1.393	1.86	1.708	1.084	880	0.13057977	isogroup00895	ENSG00000107521	ENST00000407891	HPS1
isotig08706	34.98	37.364	55.878	41.554	4212	0.134534456	isogroup00896	ENSG00000124942	ENST00000378024	AHNAK
isotig08707	35.386	37.767	56.391	41.992	4144	0.134562636	isogroup00896	ENSG00000124942	ENST00000378024	AHNAK
isotig08708	39.476	40.325	41.88	37.128	1520	0.227267604	isogroup00896	ENSG00000124942	ENST00000378024	AHNAK
isotig08709	17.851	20.103	10.82	10.562	1353	0.354550237	isogroup00896	ENSG00000124942	ENST00000378024	AHNAK
isotig08710	16.148	18.054	9.537	9.639	1320	0.389635154	isogroup00896	ENSG00000124942	ENST00000378024	AHNAK
isotig08711	9.231	7.344	7.31	6.145	4390	0.415204404	isogroup00897	ENSG00000033627	ENST00000537728	ATP6V0A1
isotig08712	9.04	7.348	7.643	6.381	4084	0.395224318	isogroup00897	ENSG00000033627	ENST00000393829	ATP6V0A1
isotig08713	6.379	5.189	5.662	4.456	1877	0.40088863	isogroup00897	ENSG00000090006	ENST00000396819	LTBP4
isotig08714	6.831	5.121	3.832	3.711	1472	0.362769595	isogroup00897	ENSG00000033627	ENST00000264649	ATP6V0A1
isotig08715	4.64	3.716	4.312	3.197	849	0.342291651	isogroup00897	ENSG00000198933	ENST00000361722	TBKBP1
isotig08716	13.438	11.185	13.876	12.04	3058	0.297230781	isogroup00898	ENSG00000180104	ENST00000512944	EXOC3
isotig08717	6.91	6.767	7.546	6.72	2766	0.09154956	isogroup00898	ENSG00000180104	ENST00000512944	EXOC3
isotig08718	10.32	8.141	9.978	8.635	2360	0.336843391	isogroup00898	ENSG00000180104	ENST00000512944	EXOC3
isotig08719	10.138	8.138	10.076	8.339	1986	0.372125573	isogroup00898	ENSG00000180104	ENST00000512944	EXOC3
isotig08720	11.463	9.882	12.034	10.202	1313	0.304454422	isogroup00898	ENSG00000180104	ENST00000512944	EXOC3
isotig08721	25.309	27.546	25.67	14.032	3811	0.449819644	isogroup00899	ENSG00000135218	ENST00000447544	CD36
isotig08722	25.935	27.968	26.614	14.84	3682	0.450336289	isogroup00899	ENSG00000135218	ENST00000447544	CD36
isotig08723	25.296	27.481	25.867	14.292	3687	0.449631773	isogroup00899	ENSG00000135218	ENST00000447544	CD36
isotig08724	4.234	3.119	4.644	3.226	2853	0.443582325	isogroup00900	ENSG00000179222	ENST00000375722	MAGED1
isotig08725	4.246	3.117	4.621	3.188	2855	0.442323589	isogroup00900	ENSG00000179222	ENST00000375722	MAGED1
isotig08726	4.942	3.647	5.36	3.721	2651	0.453314045	isogroup00900	ENSG00000179222	ENST00000375722	MAGED1
isotig08727	4.953	3.645	5.335	3.68	2653	0.44692643	isogroup00900	ENSG00000179222	ENST00000375722	MAGED1
isotig08728	5.905	15.185	8.394	8.457	3456	0.738229879	isogroup00901	ENSG00000196177	ENST00000358776	ACADSB
isotig08729	5.213	14.252	7.181	7.37	2137	0.758181784	isogroup00901	ENSG00000196177	ENST00000358776	ACADSB
isotig08730	3.929	10.289	5.105	5.554	1570	0.735186368	isogroup00901	ENSG00000196177	ENST00000358776	ACADSB
isotig08731	3.56	9.051	4.414	4.881	1559	0.722185692	isogroup00901	ENSG00000196177	ENST00000358776	ACADSB
isotig08732	3.551	8.004	4.455	5.072	1553	0.656985045	isogroup00901	ENSG00000196177	ENST00000358776	ACADSB
isotig08733	1.605	1.705	1.735	1.266	2719	0.147018487	isogroup00902	ENSG00000258890	ENST00000556440	CEP95
isotig08734	1.516	1.622	1.645	1.226	2588	0.129903434	isogroup00902	ENSG00000258890	ENST00000556440	CEP95
isotig08735	1.615	1.582	1.704	1.288	2281	0.183315172	isogroup00902	ENSG00000258890	ENST00000556440	CEP95
isotig08736	1.509	1.475	1.593	1.242	2150	0.166303449	isogroup00902	ENSG00000258890	ENST00000556440	CEP95
contig13264	2.327	1.733	2.685	1.168	901	0.534690389	isogroup00904	ENSG00000069696	ENST00000176183	DRD4
isotig08742	15.814	9.868	17.551	12.925	3194	0.681295559	isogroup00904	ENSG00000055130	ENST00000409469	CUL1
isotig08743	14.291	8.538	15.371	11.326	2196	0.692755692	isogroup00904	ENSG00000055130	ENST00000409469	CUL1
isotig08744	13.963	9.133	16.1	10.872	1394	0.694155332	isogroup00904	ENSG00000055130	ENST00000433865	CUL1
isotig08745	12.785	8.516	15.374	10.824	1183	0.672371684	isogroup00904	ENSG00000055130	ENST00000433865	CUL1
isotig08751	4.996	7.172	6.708	6.42	1925	0.218973097	isogroup00906	ENSG00000070540	ENST00000262139	WIPI1
isotig08752	4.932	6.771	6.544	6.273	1859	0.151574359	isogroup00906	ENSG00000070540	ENST00000262139	WIPI1
isotig08753	4.275	6.1	5.684	5.824	1985	0.247050425	isogroup00906	ENSG00000070540	ENST00000262139	WIPI1
isotig08754	2.848	3.163	3.957	2.939	1635	0.157802285	isogroup00906	ENSG00000197647	ENST00000550507	ZNF433
isotig08755	3.28	4.266	4.666	4.714	647	0.13700496	isogroup00906	ENSG00000070540	ENST00000262139	WIPI1
isotig08756	6.214	6.134	7.126	5.537	1615	0.302866912	isogroup00907	ENSG00000105364	ENST00000307422	MRPL4
isotig08757	5.557	5.556	6.464	4.901	1562	0.305816488	isogroup00907	ENSG00000105364	ENST00000307422	MRPL4
isotig08758	5.41	5.336	6.223	4.809	1508	0.294919967	isogroup00907	ENSG00000105364	ENST00000307422	MRPL4
isotig08759	4.676	4.686	5.48	4.1	1455	0.295530548	isogroup00907	ENSG00000105364	ENST00000307422	MRPL4
isotig08760	4.605	4.481	5.073	3.739	1031	0.324209063	isogroup00907	ENSG00000105364	ENST00000307422	MRPL4
isotig08763	9.647	9.846	6.952	11.036	1378	0.384412339	isogroup00908	ENSG00000196776	ENST00000361309	CD47
isotig08765	16.082	9.003	15.28	18.257	4857	0.300443376	isogroup00909	ENSG00000164061	ENST00000296452	BSN
isotig08769	66.596	64.822	58.394	59.709	2779	0.024028707	isogroup00910	ENSG00000213639	ENST00000395366	PPP1CB
isotig08770	68.959	67.138	60.503	61.89	2680	0.024056887	isogroup00910	ENSG00000213639	ENST00000395366	PPP1CB
isotig08771	1.676	1.813	1.429	1.402	1588	0.07293154	isogroup00911	ENSG00000257727	ENST00000273308	CNPY2
isotig08772	1.74	1.865	1.477	1.451	1518	0.066496956	isogroup00911	ENSG00000257727	ENST00000273308	CNPY2
isotig08773	2.179	2.238	1.731	1.607	1482	0.016354174	isogroup00911	ENSG00000257727	ENST00000273308	CNPY2
isotig08774	1.122	1.352	1.283	1.1	1277	0.068140828	isogroup00911	ENSG00000127586	ENST00000317063	CHTF18
isotig08775	8.065	7.909	9.732	8.136	2265	0.175819118	isogroup00912	ENSG00000224877	ENST00000431388	C17orf89
isotig08776	18.157	13.943	16.599	14.288	1061	0.44023822	isogroup00912	ENSG00000160075	ENST00000291386	SSU72
isotig08777	8.683	10.869	10.904	9.922	1009	0.122247689	isogroup00912	ENSG00000224877	ENST00000431388	C17orf89
isotig08778	6.428	3.65	5.359	4.383	1249	0.575185992	isogroup00912	ENSG00000160075	ENST00000359060	SSU72
isotig08789	9.125	7.069	10.66	10.603	1213	0.219687007	isogroup00915	ENSG00000157045	ENST00000287706	NTAN1
isotig08790	8.759	6.759	10.558	10.528	1111	0.203717968	isogroup00915	ENSG00000157045	ENST00000287706	NTAN1
isotig08791	7.614	5.755	8.592	8.465	1118	0.276921921	isogroup00915	ENSG00000157045	ENST00000287706	NTAN1
isotig08792	7.062	5.283	8.274	8.169	1016	0.251324491	isogroup00915	ENSG00000157045	ENST00000287706	NTAN1
isotig08793	6.542	4.949	7.824	5.911	1173	0.435306606	isogroup00916	ENSG00000260692	ENST00000564539	SURF1.1
isotig08794	7.54	5.537	8.865	6.775	1131	0.444718945	isogroup00916	ENSG00000260692	ENST00000564539	SURF1.1
isotig08795	7.482	5.667	8.882	6.757	1095	0.43404787	isogroup00916	ENSG00000260692	ENST00000564539	SURF1.1
isotig08796	8.592	6.327	10.044	7.717	1053	0.44277448	isogroup00916	ENSG00000260692	ENST00000564539	SURF1.1
isotig08797	51.828	36.681	50.738	34.041	1166	0.635492598	isogroup00917	ENSG00000041357	ENST00000559082	PSMA4
isotig08800	2.594	1.313	2.257	1.595	1540	0.474214699	isogroup00918	ENSG00000114956	ENST00000264093	DGUOK
isotig08801	3.652	1.878	3.054	2.189	1102	0.545680845	isogroup00918	ENSG00000114956	ENST00000264093	DGUOK
isotig08804	4.441	2.738	4.347	4.253	1111	0.385304727	isogroup00919	ENSG00000188549	ENST00000559313	C15orf52
isotig08805	4.059	2.367	3.71	3.586	1068	0.400681972	isogroup00919	ENSG00000188549	ENST00000559313	C15orf52

isotig08806	3.77	2.256	3.585	3.301	1028	0.392387465	isogroup00919	ENSG00000188549	ENST00000559313	C15orf52
isotig08807	3.632	2.241	3.468	3.044	752	0.38729616	isogroup00919	ENSG00000188549	ENST00000559313	C15orf52
isotig08808	2.786	2.938	2.565	1.852	1179	0.043172766	isogroup00920	ENSG00000113734	ENST00000231668	BNIP1
isotig08810	1.814	1.537	1.885	1.199	644	0.239122266	isogroup00920	ENSG00000113734	ENST00000231668	BNIP1
isotig08813	453.584	306.221	335.374	254.089	933	0.641570226	isogroup00921	ENSG00000197756	ENST00000491306	RPL37A
isotig08816	5.916	7.151	5.25	4.21	881	0.006660029	isogroup00922	ENSG00000166347	ENST00000340533	CYB5A
isotig08817	6.94	7.95	5.824	4.568	856	0.075402044	isogroup00922	ENSG00000166347	ENST00000340533	CYB5A
isotig08819	3.695	4.278	3.949	2.532	554	0.119354475	isogroup00922	ENSG00000166347	ENST00000397914	CYB5A
isotig08820	11.09	3.373	11.541	7.047	1428	0.848519576	isogroup00923	ENSG00000154277	ENST00000284440	UCHL1
isotig08821	35.423	14.046	36.131	20.44	1202	0.872069212	isogroup00923	ENSG00000154277	ENST00000284440	UCHL1
isotig08822	5.081	6.366	5.864	6.145	1280	0.287414519	isogroup00924	ENSG00000164172	ENST00000527216	MOCS2
isotig08823	4.78	6.194	5.422	5.962	1248	0.35342301	isogroup00924	ENSG00000164172	ENST00000527216	MOCS2
isotig08970	2.657	1.527	1.843	1.549	10697	0.347110543	isogroup00959	ENSG00000060237	ENST00000315939	WNK1
isotig08971	2.653	1.517	1.854	1.553	10583	0.350886751	isogroup00959	ENSG00000060237	ENST00000315939	WNK1
isotig08972	2.566	1.473	1.804	1.496	10445	0.343672503	isogroup00959	ENSG00000060237	ENST00000315939	WNK1
isotig08973	2.561	1.463	1.815	1.499	10331	0.348087473	isogroup00959	ENSG00000060237	ENST00000315939	WNK1
isotig08974	2.623	1.516	1.872	1.544	9956	0.351938829	isogroup00959	ENSG00000060237	ENST00000535572	WNK1
isotig08975	2.619	1.506	1.884	1.548	9842	0.355705644	isogroup00959	ENSG00000060237	ENST00000535572	WNK1
isotig08976	2.524	1.458	1.831	1.487	9704	0.348087473	isogroup00959	ENSG00000060237	ENST00000535572	WNK1
isotig08977	2.519	1.447	1.843	1.49	9590	0.349158338	isogroup00959	ENSG00000060237	ENST00000535572	WNK1
isotig08978	2.699	2.223	2.033	1.984	10857	0.17677726	isogroup00960	ENSG00000131018	ENST00000448038	SYNE1
isotig08979	2.712	2.237	2.048	1.998	10788	0.174006162	isogroup00960	ENSG00000131018	ENST00000367255	SYNE1
isotig08980	2.626	2.158	1.976	1.931	10710	0.17677726	isogroup00960	ENSG00000131018	ENST00000448038	SYNE1
isotig08981	2.639	2.171	1.99	1.946	10641	0.172869542	isogroup00960	ENSG00000131018	ENST00000367255	SYNE1
isotig08982	3.376	2.415	3	2.455	3816	0.368649959	isogroup00960	ENSG00000131018	ENST00000354674	SYNE1
isotig08983	3.426	2.459	3.06	2.506	3747	0.367635455	isogroup00960	ENSG00000131018	ENST00000354674	SYNE1
isotig08984	3.19	2.233	2.871	2.321	3669	0.380485835	isogroup00960	ENSG00000131018	ENST00000367247	SYNE1
isotig08985	3.239	2.274	2.931	2.372	3600	0.37743293	isogroup00960	ENSG00000131018	ENST00000354674	SYNE1
isotig08986	2.894	1.659	5.197	2.286	7342	0.731241076	isogroup00961	ENSG00000099204	ENST00000277895	ABLIM1
isotig08987	2.891	1.673	5.162	2.273	7258	0.727361539	isogroup00961	ENSG00000099204	ENST00000277895	ABLIM1
isotig08988	2.884	1.657	5.228	2.295	7195	0.732095889	isogroup00961	ENSG00000099204	ENST00000277895	ABLIM1
isotig08989	2.945	1.699	5.272	2.303	7117	0.734209439	isogroup00961	ENSG00000099204	ENST00000369267	ABLIM1
isotig08990	2.881	1.671	5.192	2.282	7111	0.728206959	isogroup00961	ENSG00000099204	ENST00000277895	ABLIM1
isotig08991	2.943	1.714	5.236	2.29	7033	0.729991734	isogroup00961	ENSG00000099204	ENST00000369256	ABLIM1
isotig08992	2.936	1.697	5.305	2.312	6970	0.735054858	isogroup00961	ENSG00000099204	ENST00000369267	ABLIM1
isotig08993	2.933	1.713	5.269	2.299	6886	0.730846547	isogroup00961	ENSG00000099204	ENST00000369260	ABLIM1
isotig08994	3.577	2.476	3.98	2.881	4708	0.479052378	isogroup00962	ENSG00000176986	ENST00000339365	SEC24C
isotig08996	3.637	2.532	4.073	2.941	4639	0.483636432	isogroup00962	ENSG00000176986	ENST00000339365	SEC24C
isotig08998	3.718	2.569	4.035	2.97	4564	0.477474262	isogroup00962	ENSG00000176986	ENST00000339365	SEC24C
isotig09000	3.782	2.628	4.131	3.033	4495	0.480329902	isogroup00962	ENSG00000176986	ENST00000339365	SEC24C
isotig09002	5.513	5.696	5.805	8.49	4861	0.518279853	isogroup00963	ENSG00000198952	ENST00000361813	SMG5
isotig09004	5.69	5.92	5.976	8.794	4793	0.527945818	isogroup00963	ENSG00000198952	ENST00000361813	SMG5
isotig09006	5.882	6.252	6.325	9.369	4458	0.55132637	isogroup00963	ENSG00000198952	ENST00000361813	SMG5
isotig09008	6.08	6.505	6.519	9.715	4390	0.561875329	isogroup00963	ENSG00000198952	ENST00000361813	SMG5
isotig09010	1.763	1.846	1.476	1.51	4461	0.054135042	isogroup00964	ENSG00000198836	ENST00000361510	OPA1
isotig09011	1.234	1.52	1.066	1.161	4502	0.131678816	isogroup00964	ENSG00000198836	ENST00000361510	OPA1
isotig09012	1.794	1.877	1.487	1.537	4407	0.059301495	isogroup00964	ENSG00000198836	ENST00000361908	OPA1
isotig09013	1.258	1.547	1.073	1.183	4448	0.131678816	isogroup00964	ENSG00000198836	ENST00000361908	OPA1
isotig09014	1.793	1.888	1.52	1.559	4350	0.060513264	isogroup00964	ENSG00000198836	ENST00000361510	OPA1
isotig09015	1.25	1.553	1.1	1.201	4391	0.137502818	isogroup00964	ENSG00000198836	ENST00000361510	OPA1
isotig09016	1.825	1.92	1.532	1.587	4296	0.065266401	isogroup00964	ENSG00000198836	ENST00000361828	OPA1
isotig09017	1.275	1.582	1.107	1.223	4337	0.143251672	isogroup00964	ENSG00000198836	ENST00000361828	OPA1
isotig09018	2.968	2.988	2.889	2.066	4282	0.193300519	isogroup00965	ENSG00000073921	ENST00000447890	PICALM
isotig09020	3.036	3.087	2.956	2.12	4179	0.188754039	isogroup00965	ENSG00000073921	ENST00000447890	PICALM
isotig09022	2.995	3.101	2.963	2.12	4132	0.173724356	isogroup00965	ENSG00000073921	ENST00000532317	PICALM
isotig09024	3.067	3.207	3.034	2.177	4029	0.169271812	isogroup00965	ENSG00000073921	ENST00000532317	PICALM
isotig09034	1.227	1.249	1.233	1.123	6635	0.029805741	isogroup00967	ENSG00000119760	ENST00000464789	SUPT7L
isotig09035	1.253	1.274	1.252	1.143	6608	0.029805741	isogroup00967	ENSG00000119760	ENST00000464789	SUPT7L
isotig09036	1.236	1.26	1.245	1.133	6574	0.029805741	isogroup00967	ENSG00000119760	ENST00000464789	SUPT7L
isotig09037	1.263	1.285	1.263	1.153	6547	0.035733073	isogroup00967	ENSG00000119760	ENST00000464789	SUPT7L
isotig09038	1.01	1.174	1.116	1.013	945	0.029805741	isogroup00967	ENSG00000198522	ENST00000264718	GPN1
isotig09039	1.194	1.353	1.245	1.153	918	0.02329601	isogroup00967	ENSG00000198522	ENST00000264718	GPN1
isotig09040	1.068	1.249	1.193	1.083	884	0.029805741	isogroup00967	ENSG00000198522	ENST00000264718	GPN1
isotig09041	1.267	1.443	1.334	1.235	857	0.022976629	isogroup00967	ENSG00000198522	ENST00000264718	GPN1
isotig09042	1.135	1.515	1.608	1.278	4634	0.010060494	isogroup00968	ENSG00000138002	ENST00000260570	IFT172
isotig09043	1.185	1.578	1.691	1.322	4467	0.003503795	isogroup00968	ENSG00000138002	ENST00000260570	IFT172
isotig09044	1.168	1.572	1.675	1.32	4459	0.010060494	isogroup00968	ENSG00000138002	ENST00000260570	IFT172
isotig09045	1.222	1.64	1.764	1.368	4292	0.010060494	isogroup00968	ENSG00000138002	ENST00000260570	IFT172
isotig09537	17.981	21.562	20.112	19.276	482	0.145957015	isogroup01050	ENSG00000213920	ENST00000396833	MDP1
isotig09538	3.357	2.677	3.759	3.597	10636	0.200054483	isogroup01051	ENSG00000080603	ENST00000395059	SRCAP
isotig09540	3.063	2.317	3.381	3.236	9866	0.21337454	isogroup01051	ENSG00000080603	ENST00000395059	SRCAP
isotig09542	3.838	2.376	4.967	3.532	3845	0.55262268	isogroup01051	ENSG00000149929	ENST00000279392	HIRIP3
isotig09543	4.597	5.643	4.332	4.22	10190	0.140912678	isogroup01052	ENSG00000092148	ENST00000553700	HECTD1
isotig09544	4.658	5.746	4.392	4.285	10007	0.143448937	isogroup01052	ENSG00000092148	ENST00000553700	HECTD1
isotig09545	4.221	5.248	4.343	4.114	9353	0.117710603	isogroup01052	ENSG00000092148	ENST00000553700	HECTD1
isotig09546	4.28	5.353	4.41	4.183	9170	0.129086195	isogroup01052	ENSG00000092148	ENST00000553700	HECTD1
isotig09547	8.517	4.868	5.16	3.9	7846	0.592479522	isogroup01053	ENSG00000064042	ENST00000503057	LIMCH1
isotig09548	8.639	4.897	5.213	3.96	7819	0.598942286	isogroup01053	ENSG00000064042	ENST00000503057	LIMCH1
isotig09549	8.227	4.848	5.101	3.848	7603	0.575758999	isogroup01053	ENSG00000064042	ENST00000503057	LIMCH1
isotig09550	8.352	4.878	5.156	3.91	7576	0.577863155	isogroup01053	ENSG00000064042	ENST00000503057	LIMCH1
isotig09551	8.677	5.106	5.551	4.032	6187	0.596490569	isogroup01053	ENSG00000064042	ENST00000313860	LIMCH1
isotig09552	5.325	3.962	4.702	4.1	8653	0.426993312	isogroup01054	ENSG00000125107	ENST00000317147	CNOT1

isotig09554	5.386	4.032	4.8	4.14	8618	0.435860825	isogroup01054	ENSG00000125107	ENST00000317147	CNOT1
isotig09556	3.661	2.528	3.443	2.26	2404	0.525024423	isogroup01054	ENSG00000125107	ENST00000569240	CNOT1
isotig09557	5.15	3.377	4.711	4.024	7186	0.486097543	isogroup01055	ENSG00000074054	ENST00000263710	CLASP1
isotig09559	5.203	3.407	4.772	4.113	7078	0.486097543	isogroup01055	ENSG00000074054	ENST00000541377	CLASP1
isotig09561	5.824	5.044	5.075	4.438	5397	0.301871196	isogroup01055	ENSG00000163539	ENST00000480013	CLASP2
isotig09562	3.119	3.348	2.868	3.507	6504	0.263132186	isogroup01056	ENSG00000103479	ENST00000262133	RBL2
isotig09563	3.248	3.474	2.985	3.709	6494	0.276208011	isogroup01056	ENSG00000103479	ENST00000262133	RBL2
isotig09564	3.132	3.308	2.861	3.493	6358	0.258980236	isogroup01056	ENSG00000103479	ENST00000262133	RBL2
isotig09565	3.264	3.437	2.981	3.699	6348	0.268580446	isogroup01056	ENSG00000103479	ENST00000262133	RBL2
isotig09567	4.665	4.265	4.63	3.796	6574	0.307798527	isogroup01057	ENSG00000075568	ENST00000186436	TMEM131
isotig09568	4.781	4.405	4.735	3.892	6488	0.300884873	isogroup01057	ENSG00000075568	ENST00000186436	TMEM131
isotig09569	3.212	2.996	2.868	2.582	5710	0.188754039	isogroup01057	ENSG00000075568	ENST00000186436	TMEM131
isotig09570	3.324	3.138	2.962	2.673	5624	0.181304952	isogroup01057	ENSG00000075568	ENST00000186436	TMEM131
isotig09571	7.46	6.094	8.379	5.715	1666	0.544656947	isogroup01057	ENSG00000075568	ENST00000186436	TMEM131
isotig09572	1.948	2.133	2.137	1.962	6648	0.034718569	isogroup01058	ENSG00000110713	ENST00000324932	NUP98
isotig09573	1.963	2.154	2.164	1.975	6597	0.034718569	isogroup01058	ENSG00000110713	ENST00000324932	NUP98
isotig09574	1.47	1.885	1.821	1.88	4087	0.078182536	isogroup01058	ENSG00000110713	ENST00000397007	NUP98
isotig09575	1.488	1.916	1.862	1.901	4036	0.078182536	isogroup01058	ENSG00000110713	ENST00000397004	NUP98
isotig09576	3.096	1.854	2.276	1.762	2939	0.433822424	isogroup01058	ENSG00000110713	ENST00000429801	NUP98
isotig09577	1.98	2.25	1.691	1.872	5421	0.197875178	isogroup01059	ENSG00000019144	ENST00000361417	PHLDB1
isotig09578	2.008	2.271	1.715	1.885	5376	0.192051176	isogroup01059	ENSG00000019144	ENST00000361417	PHLDB1
isotig09579	1.992	2.287	1.708	1.905	5280	0.214736605	isogroup01059	ENSG00000019144	ENST00000356063	PHLDB1
isotig09580	2.02	2.308	1.733	1.919	5235	0.208067183	isogroup01059	ENSG00000019144	ENST00000356063	PHLDB1
isotig09581	8.486	22.189	7.609	7.966	2979	0.760905914	isogroup01059	ENSG00000163931	ENST00000423516	TKT
isotig09582	7.336	6.777	10.517	11.077	5697	0.063810401	isogroup01060	ENSG00000070371	ENST00000427926	CLTCL1
isotig09583	7.159	6.604	10.225	10.746	5640	0.065144285	isogroup01060	ENSG00000070371	ENST00000427926	CLTCL1
isotig09584	7.083	6.6	10.216	10.717	5535	0.050321259	isogroup01060	ENSG00000070371	ENST00000427926	CLTCL1
isotig09585	6.898	6.42	9.913	10.373	5478	0.063810401	isogroup01060	ENSG00000070371	ENST00000427926	CLTCL1
isotig09586	4.159	3.55	5.766	5.441	1069	0.168586083	isogroup01060	ENSG00000179270	ENST00000331664	C2orf71
isotig09587	10.405	7.471	11.34	10.351	5842	0.388160367	isogroup01061	ENSG00000007314	ENST00000435607	SCN4A
isotig09588	10.912	7.876	11.944	10.957	5329	0.378315924	isogroup01061	ENSG00000007314	ENST00000435607	SCN4A
isotig09589	10.599	8.058	11.874	11.118	5292	0.315482453	isogroup01061	ENSG00000007314	ENST00000435607	SCN4A
isotig09590	11.185	8.573	12.606	11.875	4779	0.292900353	isogroup01061	ENSG00000007314	ENST00000435607	SCN4A
isotig09591	7.598	5.5	7.489	7.342	2088	0.286052454	isogroup01061	ENSG00000007314	ENST00000435607	SCN4A
isotig09592	10.029	7.611	2.721	2.655	7975	0.523568423	isogroup01062	ENSG00000132953	ENST00000400602	XPO4
isotig09593	9.753	7.43	2.684	2.605	7797	0.51359247	isogroup01062	ENSG00000132953	ENST00000400602	XPO4
isotig09594	12.416	8.221	2.448	2.496	2681	0.647629067	isogroup01062	ENSG00000132953	ENST00000400602	XPO4
isotig09595	11.727	7.699	2.316	2.33	2503	0.641720523	isogroup01062	ENSG00000132953	ENST00000400602	XPO4
isotig09596	6.092	4.334	1.05	1.319	1055	0.480132637	isogroup01062	ENSG00000132953	ENST00000456108	XPO4
isotig09597	4.597	5.499	5.103	4.281	5532	0.056474036	isogroup01063	ENSG00000138641	ENST00000402738	HERC3
isotig09598	2.588	2.878	3.312	2.596	4777	0.125807845	isogroup01063	ENSG00000138641	ENST00000402738	HERC3
isotig09599	2.625	2.92	3.36	2.634	4709	0.125807845	isogroup01063	ENSG00000138641	ENST00000402738	HERC3
isotig09600	28.202	34.345	21.711	24.308	3916	0.325336289	isogroup01063	ENSG00000152642	ENST00000282541	GPD1L
isotig09601	1.914	2.147	2.183	1.9	2810	0.010060494	isogroup01063	ENSG00000138641	ENST00000402738	HERC3
isotig09602	6.015	3.336	3.007	2.169	6034	0.601722777	isogroup01064	ENSG00000004399	ENST00000393239	PLXND1
isotig09603	6.082	3.41	3.068	2.229	5887	0.594837304	isogroup01064	ENSG00000004399	ENST00000393239	PLXND1
isotig09604	3.069	1.681	1.387	1.12	3459	0.409389795	isogroup01064	ENSG00000004399	ENST00000393239	PLXND1
isotig09605	3.057	1.739	1.423	1.179	3312	0.383022094	isogroup01064	ENSG00000004399	ENST00000393239	PLXND1
isotig09606	5.881	3.38	3.373	2.13	1699	0.630138273	isogroup01064	ENSG00000004399	ENST00000324093	PLXND1
isotig09607	2.405	2.448	3.04	2.845	4302	0.054116255	isogroup01065	ENSG00000175224	ENST00000528494	ATG13
isotig09608	2.438	2.45	3.08	2.887	4216	0.05706583	isogroup01065	ENSG00000175224	ENST00000528494	ATG13
isotig09609	2.37	2.369	3.036	2.824	4092	0.071109191	isogroup01065	ENSG00000175224	ENST00000529655	ATG13
isotig09610	2.403	2.369	3.078	2.868	4006	0.077224393	isogroup01065	ENSG00000175224	ENST00000529655	ATG13
isotig09611	2.309	2.394	2.945	2.703	3134	0.054116255	isogroup01065	ENSG00000175224	ENST00000528494	ATG13
isotig09612	3.139	3.107	2.765	2.732	4601	0.03231382	isogroup01066	ENSG00000134644	ENST00000373741	PUM1
isotig09613	3.043	2.978	2.661	2.626	4364	0.035704892	isogroup01066	ENSG00000134644	ENST00000373741	PUM1
isotig09614	2.966	3.003	2.676	2.652	4313	0.014428496	isogroup01066	ENSG00000134644	ENST00000373742	PUM1
isotig09615	2.854	2.859	2.559	2.534	4076	0.017143233	isogroup01066	ENSG00000134644	ENST00000373742	PUM1
isotig09617	3.515	4.477	5.674	4.964	6674	0.040580146	isogroup01067	ENSG00000148384	ENST00000371712	INPP5E
isotig09618	3.109	3.907	5.109	4.359	6410	0.010060494	isogroup01067	ENSG00000148384	ENST00000371712	INPP5E
isotig09619	5.268	6.207	6.702	6.522	2069	0.140010897	isogroup01067	ENSG00000165688	ENST00000371717	PMPCA
isotig09620	1.21	1.411	1.835	1.781	1848	0.003503795	isogroup01067	ENSG00000148384	ENST00000371712	INPP5E
isotig09621	4.08	4.437	4.847	4.604	1805	0.056474036	isogroup01067	ENSG00000165688	ENST00000371717	PMPCA
isotig09622	3.944	4.1	4.29	3.033	4637	0.284511911	isogroup01068	ENSG00000254996	ENST00000532219	ANKHD1-EIF4EBP3
isotig09623	3.977	4.123	4.293	3.049	4586	0.286024273	isogroup01068	ENSG00000254996	ENST00000532219	ANKHD1-EIF4EBP3
isotig09624	1.463	1.513	1.5	1.352	3720	0.042270985	isogroup01068	ENSG00000131503	ENST00000431508	ANKHD1
isotig09625	1.47	1.506	1.466	1.348	3669	0.042270985	isogroup01068	ENSG00000131503	ENST00000360839	ANKHD1
isotig09626	15.354	16.183	16.746	9.986	1511	0.528490644	isogroup01068	ENSG00000243056	ENST00000310331	EIF4EBP3
isotig09627	5.115	4.249	4.998	4.146	4057	0.314439769	isogroup01069	ENSG00000135108	ENST00000330622	FBXO21
isotig09629	4.938	3.86	4.803	3.975	3899	0.370566243	isogroup01069	ENSG00000135108	ENST00000330622	FBXO21
isotig09631	3.853	2.467	2.601	2.469	1517	0.365089802	isogroup01069	ENSG00000135108	ENST00000427718	FBXO21
isotig09632	1.514	1.571	1.512	1.56	3717	0.010060494	isogroup01070	ENSG00000105655	ENST00000338128	ISYNA1
isotig09633	1.526	1.61	1.534	1.601	3651	0.016354174	isogroup01070	ENSG00000105655	ENST00000338128	ISYNA1
isotig09634	1.559	1.635	1.582	1.584	3556	0.014428496	isogroup01070	ENSG00000105655	ENST00000338128	ISYNA1
isotig09635	1.572	1.677	1.606	1.626	3490	0.021652138	isogroup01070	ENSG00000105655	ENST00000338128	ISYNA1
isotig09636	1.111	1.113	1.098	1.059	2617	0.02329601	isogroup01070	ENSG00000105655	ENST00000338128	ISYNA1
isotig09637	3.178	3.219	2.944	2.185	5078	0.188754039	isogroup01071	ENSG00000119707	ENST00000527432	RBM25
isotig09638	3.246	3.175	2.985	1.99	4979	0.275907417	isogroup01071	ENSG00000119707	ENST00000527432	RBM25
isotig09639	3.886	3.94	3.138	2.514	2431	0.150146539	isogroup01071	ENSG00000119707	ENST00000540173	RBM25
isotig09640	4.061	3.877	3.235	2.112	2332	0.327186819	isogroup01071	ENSG00000119707	ENST00000527432	RBM25
isotig09641	3.794	3.872	1.998	2.149	1365	0.021652138	isogroup01071	ENSG00000119707	ENST00000540173	RBM25
isotig09642	5.49	3.96	4.615	4.714	4474	0.262887954	isogroup01072	ENSG00000162923	ENST00000414423	WDR26

isotig09643	5.532	3.986	4.661	4.763	4426	0.262887954	isogroup01072	ENSG00000162923	ENST00000414423	WDR26
isotig09644	3.824	2.592	3.597	3.383	2698	0.330728188	isogroup01072	ENSG00000116560	ENST00000357214	SFPQ
isotig09645	4.723	3.701	3.598	4.1	2263	0.126258736	isogroup01072	ENSG00000162923	ENST00000414423	WDR26
isotig09646	4.79	3.747	3.668	4.184	2215	0.124520929	isogroup01072	ENSG00000162923	ENST00000414423	WDR26
isotig09647	7.625	7.044	8.947	11.867	4792	0.348021718	isogroup01073	ENSG00000187715	ENST00000405256	KBTBD12
isotig09648	7.858	7.199	9.211	12.211	4693	0.348021718	isogroup01073	ENSG00000187715	ENST00000405256	KBTBD12
isotig09649	10.116	6.487	10.851	12.738	2522	0.137577966	isogroup01073	ENSG00000187715	ENST00000405256	KBTBD12
isotig09650	10.669	6.764	11.439	13.439	2423	0.153067934	isogroup01073	ENSG00000187715	ENST00000405256	KBTBD12
isotig09651	1.65	1.081	1.78	1.848	3406	0.252094762	isogroup01074	ENSG00000115282	ENST00000233623	TTC31
isotig09652	1.657	1.103	1.772	1.859	3286	0.25155933	isogroup01074	ENSG00000115282	ENST00000233623	TTC31
isotig09653	1.564	1.019	1.673	1.723	3231	0.242344255	isogroup01074	ENSG00000115282	ENST00000233623	TTC31
isotig10065	17.262	12.583	20.812	14.854	1278	0.562701961	isogroup01177	ENSG00000107581	ENST00000369144	EIF3A
isotig10066	4.152	4.322	14.153	51.719	1873	0.820968287	isogroup01178	ENSG00000115415	ENST00000409465	STAT1
isotig10067	4.668	4.926	15.986	58.929	1709	0.832898099	isogroup01178	ENSG00000115415	ENST00000409465	STAT1
isotig10068	4.831	4.671	16.368	53.733	1556	0.765480574	isogroup01178	ENSG00000115415	ENST00000409465	STAT1
isotig10069	4.385	4.529	15.37	51.402	1183	0.781271135	isogroup01178	ENSG00000115415	ENST00000409465	STAT1
isotig10070	1.528	2.095	1.411	1.483	2006	0.20052416	isogroup01179	ENSG00000197885	ENST00000443659	NKIRAS1
isotig10071	10.316	6.634	9.599	6.052	1647	0.678674758	isogroup01179	ENSG00000170142	ENST00000306627	UBE2E1
isotig10072	4.217	3.021	3.66	2.931	1442	0.446437965	isogroup01179	ENSG00000197885	ENST00000443659	NKIRAS1
isotig10074	1.901	2.314	1.686	1.6	1684	0.227370933	isogroup01180	ENSG00000163072	ENST00000317647	NOSTRIN
isotig10075	1.837	2.071	1.609	1.431	1569	0.113154731	isogroup01180	ENSG00000163072	ENST00000317647	NOSTRIN
isotig10076	2.973	2.107	3.518	1.416	1518	0.589210566	isogroup01180	ENSG00000163072	ENST00000458381	NOSTRIN
isotig10078	6.674	3.564	6.107	3.595	1901	0.739075299	isogroup01181	ENSG00000231115	ENST00000427374	RING1
isotig10079	6.443	3.866	5.758	3.783	1718	0.654401819	isogroup01181	ENSG00000231115	ENST00000427374	RING1
isotig10080	3.979	2.114	2.97	1.974	1251	0.597955963	isogroup01181	ENSG00000231115	ENST00000427374	RING1
isotig10081	4.212	2.207	2.793	2.292	537	0.559592696	isogroup01181	ENSG00000231115	ENST00000427374	RING1
isotig10083	49.611	39.084	38.988	37.51	1368	0.35871158	isogroup01182	ENSG00000091164	ENST00000217515	TXNL1
isotig10085	4.191	2.615	4.944	4.335	1695	0.318582325	isogroup01183	ENSG00000172775	ENST00000569266	FAM192A
isotig10086	4.094	2.739	5.021	4.271	1691	0.327712858	isogroup01183	ENSG00000172775	ENST00000569266	FAM192A
isotig10087	4.296	2.711	5.149	4.511	1579	0.299494627	isogroup01183	ENSG00000172775	ENST00000569266	FAM192A
isotig10088	1.874	2.456	2.362	2.132	1764	0.06751146	isogroup01184	ENSG00000148688	ENST00000371703	RPP30
isotig10089	2.706	3.392	3.352	3.043	1199	0.059555121	isogroup01184	ENSG00000148688	ENST00000371703	RPP30
isotig10090	6.013	3.698	6.758	4.304	827	0.625197265	isogroup01184	ENSG00000148688	ENST00000413330	RPP30
isotig10092	12.323	13.401	14.253	15.077	1620	0.126615691	isogroup01185	ENSG00000168904	ENST00000301981	LRRC28
isotig10093	11.303	12.505	13.091	13.738	1579	0.140640265	isogroup01185	ENSG00000168904	ENST00000301981	LRRC28
isotig10094	10.96	12.493	13.131	13.527	1468	0.155886	isogroup01185	ENSG00000168904	ENST00000301981	LRRC28
isotig10097	1.326	1.31	1.074	1.213	1047	0.061922297	isogroup01186	ENSG00000150977	ENST00000280571	RILPL2
isotig10099	18.814	14.053	18.998	12.344	1686	0.578276471	isogroup01187	ENSG00000100345	ENST00000216181	MYH9
isotig10100	7.563	6.883	5.726	3.388	1197	0.409944014	isogroup01187	ENSG00000133026	ENST00000396239	MYH10
isotig10101	30.298	23.17	31.433	19.603	1044	0.610195762	isogroup01187	ENSG00000100345	ENST00000216181	MYH9
isotig10102	28.423	17.012	41.31	31.845	645	0.636225295	isogroup01187	ENSG00000105357	ENST00000440075	MYH14
isotig10103	35.958	33.856	31.774	34.01	1250	0.088064552	isogroup01188	ENSG00000077522	ENST00000542672	ACTN2
isotig10105	777.8	706.74	747.406	738.667	974	0.171329	isogroup01188	ENSG00000077522	ENST00000366578	ACTN2
isotig10106	992.609	935.944	1119.901	956.286	962	0.265978432	isogroup01188	ENSG00000077522	ENST00000366578	ACTN2
isotig10107	4.094	2.343	2.486	1.471	1491	0.49857218	isogroup01189	ENSG00000154305	ENST00000340535	MIA3
isotig10108	4.004	2.487	2.442	1.53	1420	0.444052003	isogroup01189	ENSG00000154305	ENST00000340535	MIA3
isotig10110	3.283	2.932	2.498	1.778	428	0.252648982	isogroup01189	ENSG00000221972	ENST00000408895	C3orf36
isotig10111	11.751	14.783	14.748	12.755	1414	0.098378673	isogroup01190	ENSG00000204316	ENST00000309352	MRPL38
isotig10112	10.672	12.824	13.313	11.344	1368	0.022356654	isogroup01190	ENSG00000204316	ENST00000309352	MRPL38
isotig10113	5.497	7.11	7.325	5.562	999	0.006660029	isogroup01190	ENSG00000204316	ENST00000409963	MRPL38
isotig10114	10.378	15.228	10.579	11.141	1147	0.417693695	isogroup01191	ENSG00000112110	ENST00000367034	MRPL18
isotig10115	9.956	14.527	9.931	10.771	1105	0.437476516	isogroup01191	ENSG00000112110	ENST00000367034	MRPL18
isotig10116	9.798	12.511	9.773	9.536	961	0.263273089	isogroup01191	ENSG00000112110	ENST00000367034	MRPL18
isotig10121	6.515	9.933	9.77	10.792	1638	0.395590667	isogroup01193	ENSG00000157557	ENST00000360938	ETS2
isotig10122	7.392	12.917	11.751	14.256	1295	0.56306831	isogroup01193	ENSG00000157557	ENST00000360938	ETS2
isotig10123	2.876	3.507	4.084	3.667	523	0.051664537	isogroup01193	ENSG00000157557	ENST00000456966	ETS2
isotig10125	16.047	12.246	25.07	14.359	917	0.719442774	isogroup01194	ENSG00000160271	ENST00000372062	RALGDS
isotig10126	12.102	9.113	18.661	10.152	706	0.706695724	isogroup01194	ENSG00000160271	ENST00000372062	RALGDS
isotig10127	4.041	2.321	4.334	2.183	607	0.593672503	isogroup01194	ENSG00000128159	ENST00000425018	TUBGCP6
isotig10128	47.21	39.973	49.25	31.737	1166	0.555872849	isogroup01195	ENSG00000160014	ENST00000391918	CALM3
isotig10132	2.777	2.458	3.287	2.171	1320	0.326566845	isogroup01197	ENSG00000183580	ENST00000504595	FBXL7
isotig10135	52.72	46.122	47.538	38.352	1255	0.436405651	isogroup01198	ENSG00000129084	ENST00000396393	PSMA1
isotig10144	6.354	8.363	10.671	10.8	1765	0.298094988	isogroup01201	ENSG00000157514	ENST00000506081	TSC22D3
isotig10145	14.91	22.428	19.137	25.445	659	0.66434959	isogroup01201	ENSG00000157514	ENST00000506081	TSC22D3
isotig10150	19.22	13.523	18.32	14.873	1530	0.56219471	isogroup01203	ENSG00000126067	ENST00000373237	PSMB2
isotig10151	27.666	22.662	27.524	23.585	850	0.427979635	isogroup01203	ENSG00000126067	ENST00000373237	PSMB2
isotig10152	114.049	113.383	113.699	120.2	775	0.008350868	isogroup01204	ENSG00000101439	ENST00000398411	CST3
isotig10153	86.992	83.805	83.693	89.826	921	0.040739836	isogroup01204	ENSG00000101439	ENST00000398411	CST3
isotig10154	62.337	61.566	60.385	63.803	629	0.023681145	isogroup01204	ENSG00000101439	ENST00000398411	CST3
isotig10155	72.583	41.704	58.155	42.776	796	0.71567596	isogroup01205	ENSG00000108294	ENST00000225426	PSMB3
isotig10157	40.521	38.371	46.112	37.209	726	0.358401593	isogroup01206	ENSG00000105583	ENST00000222190	WDR83OS
isotig10159	29.093	28.334	31.809	26.1	613	0.298329826	isogroup01206	ENSG00000105583	ENST00000222190	WDR83OS
isotig10160	75.684	36.875	54.431	33.916	901	0.816647253	isogroup01207	ENSG00000135404	ENST00000552692	CD63
isotig10161	80.272	39.179	57.768	36.009	848	0.817445705	isogroup01207	ENSG00000135404	ENST00000552692	CD63
isotig10162	127.576	128.054	107.395	113.749	925	0.023962952	isogroup01208	ENSG00000136930	ENST00000536392	PSMB7
isotig10164	35.269	61.781	52.792	51.018	722	0.412245435	isogroup01209	ENSG00000231555	ENST00000551428	HSPA1B
isotig10165	51.409	82.659	79.349	74.979	607	0.334213196	isogroup01209	ENSG00000231555	ENST00000551428	HSPA1B
isotig10167	54.386	59.051	71.681	63.324	1348	0.089332682	isogroup01210	ENSG00000099341	ENST00000215071	PSMD8
contig15478	29.871	37.888	38.107	31.572	580	0.023605997	isogroup01214	ENSG00000140990	ENST00000543683	NDUFB10
isotig10180	241.258	291.652	301.198	258.41	814	0.058136695	isogroup01214	ENSG00000140990	ENST00000268668	NDUFB10
isotig10181	120.273	75.994	95.327	68.75	535	0.658751033	isogroup01215	ENSG00000163584	ENST00000466674	RPL22L1
isotig10183	64.471	68.386	60.215	58.706	609	0.199115127	isogroup01216	ENSG00000100142	ENST00000442738	POLR2F

isotig10185	2.582	2.136	2.652	2.13	1114	0.287423912	isogroup01217	ENSG00000186687	ENST00000379380	LYRM7
isotig10189	4.504	4.08	3.729	2.781	3095	0.296732923	isogroup01219	ENSG00000136518	ENST00000429709	ACTL6A
isotig10190	4.199	3.596	3.321	2.483	3025	0.317389344	isogroup01219	ENSG00000136518	ENST00000429709	ACTL6A
isotig10192	2.769	6.531	3.197	5.058	2983	0.885079282	isogroup01220	ENSG00000123999	ENST00000243786	INHA
isotig10195	134.831	121.822	128.399	118.151	2056	0.293548508	isogroup01221	ENSG00000065427	ENST00000302445	KARS
isotig10196	137.965	124.696	131.349	120.886	2008	0.293642444	isogroup01221	ENSG00000065427	ENST00000302445	KARS
isotig10197	100.05	90.079	93.439	86.474	1231	0.30706583	isogroup01221	ENSG00000065427	ENST00000302445	KARS
contig15536	5.177	3.733	5.683	3.978	1030	0.475492222	isogroup01222	ENSG00000116690	ENST00000367484	PRG4
isotig10198	8.017	7.495	8.584	7.943	2080	0.118612384	isogroup01222	ENSG00000106246	ENST00000555673	PTCD1
isotig10200	29.637	23.825	26.841	21.249	3719	0.3527091	isogroup01223	ENSG00000084234	ENST00000263574	APLP2
isotig10201	45.404	70.8	51.644	44.9	730	0.485308484	isogroup01224	ENSG00000197747	ENST00000368811	S100A10
isotig10202	50.78	80.589	59.163	50.256	676	0.486670549	isogroup01224	ENSG00000197747	ENST00000368811	S100A10
isotig10204	49.014	41.867	57.167	44.934	1323	0.437081987	isogroup01225	ENSG00000111639	ENST00000229238	MRPL51
isotig10205	84.854	61.939	83.711	67.727	671	0.552012099	isogroup01225	ENSG00000111639	ENST00000229238	MRPL51
isotig10206	60.837	63.335	101.572	75.414	594	0.415448636	isogroup01226	ENSG00000134248	ENST00000256644	HBXIP
isotig10207	34.56	35.312	54.638	42.429	525	0.344790336	isogroup01226	ENSG00000134248	ENST00000483260	HBXIP
isotig10209	93.677	25.54	70.34	56.391	668	0.914697152	isogroup01227	ENSG00000171680	ENST00000544978	PLEKHG5
isotig10210	90.215	114.712	103.006	92.698	2191	0.200665064	isogroup01228	ENSG00000136143	ENST00000378654	SUCLA2
isotig10211	92.041	116.967	104.827	94.383	2120	0.200712031	isogroup01228	ENSG00000136143	ENST00000378654	SUCLA2
isotig10212	2.952	2.75	2.877	2.572	6969	0.229813256	isogroup01229	ENSG00000109756	ENST00000264431	RAPGEF2
isotig10214	2.97	2.773	2.886	2.582	6903	0.222739911	isogroup01229	ENSG00000109756	ENST00000264431	RAPGEF2
isotig10215	2.981	2.754	2.909	2.597	6894	0.232884948	isogroup01229	ENSG00000109756	ENST00000264431	RAPGEF2
isotig10218	2.999	2.777	2.918	2.607	6828	0.232884948	isogroup01229	ENSG00000109756	ENST00000264431	RAPGEF2
isotig10220	5.207	8.529	6.352	7.3	4664	0.528753663	isogroup01230	ENSG00000087299	ENST00000421284	L2HGDH
isotig10222	5.381	8.788	6.543	7.575	4552	0.539002029	isogroup01230	ENSG00000087299	ENST00000421284	L2HGDH
isotig10224	4.044	5.51	4.523	5.046	3027	0.329197039	isogroup01230	ENSG00000087299	ENST00000421284	L2HGDH
isotig10226	4.272	5.8	4.751	5.389	2915	0.344057639	isogroup01230	ENSG00000087299	ENST00000421284	L2HGDH
isotig10228	1.751	1.256	2.476	2.272	3682	0.277288269	isogroup01231	ENSG00000049759	ENST00000400345	NEDD4L
isotig10230	1.711	1.246	2.449	2.26	3628	0.262230405	isogroup01231	ENSG00000049759	ENST00000400345	NEDD4L
isotig10232	1.679	1.34	2.552	2.44	3232	0.216728038	isogroup01231	ENSG00000049759	ENST00000256830	NEDD4L
isotig10234	1.632	1.33	2.523	2.429	3178	0.199603592	isogroup01231	ENSG00000049759	ENST00000256830	NEDD4L
isotig10236	3.876	2.695	2.906	2.59	3447	0.302462989	isogroup01232	ENSG00000137776	ENST00000380516	SLTM
isotig10238	3.63	2.487	2.77	2.438	3276	0.310738709	isogroup01232	ENSG00000137776	ENST00000380516	SLTM
isotig10240	3.693	2.574	2.813	2.466	3266	0.304989855	isogroup01232	ENSG00000137776	ENST00000380516	SLTM
isotig10242	3.423	2.348	2.664	2.298	3095	0.312241677	isogroup01232	ENSG00000137776	ENST00000380516	SLTM
isotig10244	1.871	1.485	1.286	1.096	3252	0.123468851	isogroup01233	ENSG00000182606	ENST00000327628	TRAK1
isotig10246	1.921	1.505	1.317	1.093	3189	0.141805065	isogroup01233	ENSG00000182606	ENST00000327628	TRAK1
isotig10248	1.812	1.495	1.291	1.103	3161	0.098350492	isogroup01233	ENSG00000182606	ENST00000396175	TRAK1
isotig10250	1.862	1.516	1.323	1.1	3098	0.116996693	isogroup01233	ENSG00000182606	ENST00000396175	TRAK1
isotig10252	9.784	6.792	11.474	11.036	3312	0.386554069	isogroup01234	ENSG00000135766	ENST00000366641	EGLN1
isotig10254	9.312	6.314	10.82	10.299	3244	0.416143759	isogroup01234	ENSG00000135766	ENST00000366641	EGLN1
isotig10256	8.624	5.958	10.19	9.576	3175	0.402466747	isogroup01234	ENSG00000135766	ENST00000366641	EGLN1
isotig10258	6.88	4.958	8.606	7.54	2819	0.430224694	isogroup01234	ENSG00000135766	ENST00000366641	EGLN1
isotig10260	1.276	1.416	1.335	1.36	3513	0.09326858	isogroup01235	ENSG00000162604	ENST00000371180	TM2D1
isotig10261	1.194	1.323	1.238	1.27	3405	0.093559781	isogroup01235	ENSG00000162604	ENST00000371180	TM2D1
isotig10262	1.254	1.402	1.341	1.329	3063	0.086824604	isogroup01235	ENSG00000162604	ENST00000371180	TM2D1
isotig10263	1.16	1.294	1.229	1.224	2955	0.087209739	isogroup01235	ENSG00000162604	ENST00000371180	TM2D1
isotig10264	1.269	1.476	1.271	1.391	2851	0.136817089	isogroup01235	ENSG00000162604	ENST00000371180	TM2D1
isotig10265	1.168	1.363	1.148	1.28	2743	0.137502818	isogroup01235	ENSG00000162604	ENST00000371180	TM2D1
isotig10266	1.24	1.469	1.267	1.358	2401	0.131406403	isogroup01235	ENSG00000162604	ENST00000371180	TM2D1
isotig10267	1.118	1.333	1.119	1.224	2293	0.137709476	isogroup01235	ENSG00000162604	ENST00000371180	TM2D1
isotig10268	1.762	1.341	1.31	1.083	2952	0.172860149	isogroup01236	ENSG00000156256	ENST00000399976	USP16
isotig10270	1.752	1.312	1.276	1.06	2850	0.173245284	isogroup01236	ENSG00000156256	ENST00000399976	USP16
isotig10276	9.628	7.117	12.157	9.638	3335	0.500695123	isogroup01237	ENSG00000130821	ENST00000253122	SLC6A8
isotig10278	9.189	6.808	11.608	9.176	3302	0.49828098	isogroup01237	ENSG00000130821	ENST00000253122	SLC6A8
isotig10280	7.124	5.234	9.344	6.707	2193	0.515668445	isogroup01237	ENSG00000130821	ENST00000328897	SLC6A8
isotig10282	6.414	4.732	8.461	5.957	2160	0.516185091	isogroup01237	ENSG00000130821	ENST00000328897	SLC6A8
isotig10284	9.939	12.422	11.567	10.589	2564	0.051805441	isogroup01238	ENSG00000107745	ENST00000361114	MICU1
isotig10286	10.808	13.141	12.598	11.333	2281	0.095560607	isogroup01238	ENSG00000107745	ENST00000361114	MICU1
isotig10288	4.693	6.22	5.12	5.116	1898	0.029937251	isogroup01238	ENSG00000107745	ENST00000361114	MICU1
isotig10290	5.002	6.149	5.445	5.209	1615	0.071052829	isogroup01238	ENSG00000107745	ENST00000361114	MICU1
isotig10292	6.124	3.274	5.457	3.618	1663	0.667534005	isogroup01239	ENSG00000175193	ENST00000317096	PARL
isotig10294	6.66	3.561	5.83	4.001	1578	0.663325693	isogroup01239	ENSG00000175193	ENST00000317096	PARL
isotig10296	6.865	3.941	6.435	4.44	1485	0.646379725	isogroup01239	ENSG00000175193	ENST00000317096	PARL
isotig10298	7.514	4.306	6.914	4.921	1400	0.644651311	isogroup01239	ENSG00000175193	ENST00000317096	PARL
isotig10300	12.373	9.884	13.33	16.75	1331	0.037621177	isogroup01240	ENSG00000101400	ENST00000217381	SNTA1
isotig10301	12.252	9.774	13.085	16.646	1303	0.052209363	isogroup01240	ENSG00000101400	ENST00000217381	SNTA1
isotig10302	19.002	15.049	20.45	25.747	1250	0.022563313	isogroup01240	ENSG00000101400	ENST00000217381	SNTA1
isotig10303	14.267	11.399	15.433	19.446	1219	0.052660254	isogroup01240	ENSG00000101400	ENST00000217381	SNTA1
isotig10304	13.701	10.961	14.759	18.583	1194	0.038522958	isogroup01240	ENSG00000101400	ENST00000217381	SNTA1
isotig10305	13.597	10.866	14.52	18.511	1166	0.053468099	isogroup01240	ENSG00000101400	ENST00000217381	SNTA1
isotig10306	21.243	16.841	22.859	28.82	1113	0.023070564	isogroup01240	ENSG00000101400	ENST00000217381	SNTA1
isotig10307	15.972	12.78	17.276	21.81	1082	0.053580822	isogroup01240	ENSG00000101400	ENST00000217381	SNTA1
isotig10308	10.881	13.022	10.368	10.298	1545	0.181154655	isogroup01241	ENSG00000156219	ENST00000355810	ART3
isotig10310	10.603	12.784	10.063	10.161	1512	0.194455925	isogroup01241	ENSG00000156219	ENST00000355810	ART3
isotig10316	2.485	4.049	2.374	1.912	1979	0.243067558	isogroup01242	ENSG00000109445	ENST00000421169	ZNF330
isotig10317	3.675	4.752	3.387	2.765	1858	0.040580146	isogroup01242	ENSG00000109445	ENST00000262990	ZNF330
isotig10319	2.732	4.47	2.627	2.117	1782	0.256622454	isogroup01242	ENSG00000109445	ENST00000421169	ZNF330
isotig10320	4.081	5.287	3.778	3.087	1661	0.042524611	isogroup01242	ENSG00000109445	ENST00000262990	ZNF330
isotig10321	3.982	4.884	3.342	3.235	886	0.131678816	isogroup01242	ENSG00000109445	ENST00000512809	ZNF330
isotig10323	19.624	15.173	12.449	13.431	1326	0.245162321	isogroup01243	ENSG00000205707	ENST00000381356	LYRM5
isotig10324	20.081	15.849	12.921	13.88	1279	0.216615315	isogroup01243	ENSG00000205707	ENST00000381356	LYRM5

isotig10330	3.138	3.681	3.186	2.752	1193	0.035704892	isogroup01244	ENSG00000128891	ENST00000558750	C15orf57
isotig10331	3.226	3.769	3.268	2.821	1148	0.035704892	isogroup01244	ENSG00000128891	ENST00000558750	C15orf57
isotig10332	2.746	3.205	2.8	2.331	906	0.035704892	isogroup01244	ENSG00000128891	ENST00000559153	C15orf57
isotig10333	2.843	3.3	2.89	2.401	861	0.038353874	isogroup01244	ENSG00000128891	ENST00000559153	C15orf57
isotig10334	2.487	2.852	2.482	2.194	797	0.014428496	isogroup01244	ENSG00000128891	ENST00000558750	C15orf57
isotig10335	2.582	2.939	2.565	2.266	752	0.010060494	isogroup01244	ENSG00000128891	ENST00000558750	C15orf57
isotig10336	2.145	2.473	2.114	1.953	674	0.05275419	isogroup01244	ENSG00000128891	ENST00000558113	C15orf57
isotig10337	75.508	66.808	72.324	54.93	712	0.440116104	isogroup01245	ENSG00000184983	ENST00000498737	NDUFA6
isotig10343	14.601	10.15	8.515	8.747	11913	0.390884497	isogroup01246	ENSG00000103657	ENST00000443617	HERC1
isotig10347	6.075	4.103	5.081	3.548	777	0.561668671	isogroup01246	ENSG00000103657	ENST00000443617	HERC1
isotig10348	6.393	4.355	5.369	3.751	732	0.567680544	isogroup01246	ENSG00000103657	ENST00000443617	HERC1
isotig10349	2.483	2.697	2.296	2.129	6888	0.126700233	isogroup01247	ENSG00000104067	ENST00000356107	TJP1
isotig10350	2.503	2.717	2.316	2.141	6849	0.126700233	isogroup01247	ENSG00000104067	ENST00000356107	TJP1
isotig10352	2.467	2.7	2.318	2.113	6647	0.119636282	isogroup01247	ENSG00000104067	ENST00000545208	TJP1
isotig10353	2.488	2.721	2.339	2.126	6608	0.119636282	isogroup01247	ENSG00000104067	ENST00000545208	TJP1
isotig10355	1.173	2.48	1.393	1.575	5682	0.444268054	isogroup01248	ENSG00000198853	ENST00000455600	RUSC2
isotig10356	1.203	2.53	1.417	1.601	5588	0.450777786	isogroup01248	ENSG00000198853	ENST00000455600	RUSC2
isotig10357	1.211	2.557	1.45	1.646	5306	0.453558278	isogroup01248	ENSG00000198853	ENST00000455600	RUSC2
isotig10358	1.235	2.597	1.468	1.677	5273	0.45678966	isogroup01248	ENSG00000198853	ENST00000455600	RUSC2
isotig10359	1.243	2.612	1.477	1.676	5212	0.460124371	isogroup01248	ENSG00000198853	ENST00000455600	RUSC2
isotig10360	1.269	2.654	1.495	1.707	5179	0.463261817	isogroup01248	ENSG00000198853	ENST00000455600	RUSC2
isotig10361	8.289	6.319	9.673	7.48	5115	0.409831292	isogroup01249	ENSG00000123983	ENST00000392066	ACSL3
isotig10363	10.204	7.841	11.04	9.461	4975	0.31436462	isogroup01249	ENSG00000068366	ENST00000469796	ACSL4
isotig10365	3.282	2.908	3.066	1.84	2945	0.323279101	isogroup01249	ENSG00000123983	ENST00000392066	ACSL3
isotig10366	6.315	5.313	4.99	4.952	2805	0.085274667	isogroup01249	ENSG00000068366	ENST00000469796	ACSL4
isotig10367	5.042	4.431	3.064	3.528	6349	0.029937251	isogroup01250	ENSG00000113742	ENST00000265085	CPEB4
isotig10371	4.822	4.198	1.442	2.754	3707	0.145975802	isogroup01250	ENSG00000113742	ENST00000265085	CPEB4
isotig10379	9.78	11.405	8.867	9.356	4628	0.179266551	isogroup01252	ENSG00000139793	ENST00000345429	MBNL2
isotig10381	9.784	11.608	8.923	9.453	4533	0.206583001	isogroup01252	ENSG00000139793	ENST00000376673	MBNL2
isotig10383	7.916	8.605	8.095	6.89	3027	0.116893364	isogroup01252	ENSG00000139793	ENST00000445661	MBNL2
isotig10385	3.111	3.366	2.207	2.341	3271	0.064064026	isogroup01253	ENSG00000152904	ENST00000488594	GGPS1
isotig10387	3.292	3.499	2.328	2.441	3109	0.035704892	isogroup01253	ENSG00000152904	ENST00000488594	GGPS1
isotig10391	9.272	10.836	8.098	8.86	3340	0.191205756	isogroup01254	ENSG00000177354	ENST00000374144	C10orf71
isotig10392	8.39	9.722	7.319	7.875	3299	0.162433306	isogroup01254	ENSG00000177354	ENST00000374144	C10orf71
isotig10394	10.304	12.074	9.052	9.886	2988	0.193864132	isogroup01254	ENSG00000177354	ENST00000374144	C10orf71
isotig10395	9.332	10.845	8.194	8.797	2947	0.164725333	isogroup01254	ENSG00000177354	ENST00000374144	C10orf71
isotig10399	5.428	4.012	5.637	3.01	3931	0.540072894	isogroup01255	ENSG00000119280	ENST00000366663	C1orf198
isotig10403	6.864	9.175	9.594	7.409	3693	0.078708574	isogroup01256	ENSG00000132305	ENST00000410111	IMMT
isotig10404	6.986	9.348	9.773	7.553	3657	0.079666717	isogroup01256	ENSG00000132305	ENST00000442664	IMMT
isotig10405	7.031	9.643	9.959	7.736	3555	0.107396483	isogroup01256	ENSG00000132305	ENST00000409051	IMMT
isotig10406	5.194	6.581	6.751	5.593	2253	0.113539866	isogroup01256	ENSG00000132305	ENST00000410111	IMMT
isotig10407	5.368	6.825	7	5.802	2217	0.126841136	isogroup01256	ENSG00000132305	ENST00000442664	IMMT
isotig10408	5.365	7.199	7.178	6.025	2115	0.182300669	isogroup01256	ENSG00000132305	ENST00000409051	IMMT
isotig10409	2.442	3.197	3.734	2.862	4226	0.010060494	isogroup01257	ENSG00000147274	ENST00000562646	RBMX
isotig10410	2.836	3.665	4.266	3.306	3601	0.017143233	isogroup01257	ENSG00000147274	ENST00000320676	RBMX
isotig10411	2.609	3.281	3.67	2.841	2984	0.046479297	isogroup01257	ENSG00000147274	ENST00000562646	RBMX
isotig10412	2.575	3.5	3.849	3.174	2370	0.094226723	isogroup01257	ENSG00000147274	ENST00000562646	RBMX
isotig10413	3.254	4.017	4.465	3.514	2359	0.064082814	isogroup01257	ENSG00000147274	ENST00000320676	RBMX
isotig10421	4.058	3.064	4.191	3.69	4748	0.315163072	isogroup01259	ENSG00000112078	ENST00000373731	KCTD20
isotig10422	4.147	3.118	4.352	3.83	4474	0.321344029	isogroup01259	ENSG00000112078	ENST00000536244	KCTD20
isotig10423	4.215	3.165	4.452	3.914	4269	0.329892162	isogroup01259	ENSG00000112078	ENST00000373731	KCTD20
isotig10424	1.797	1.357	1.473	1.335	1294	0.123468851	isogroup01259	ENSG00000112078	ENST00000373731	KCTD20
isotig10425	1.579	1.138	1.453	1.321	1020	0.154899677	isogroup01259	ENSG00000112078	ENST00000536244	KCTD20
isotig10427	3.251	1.887	2.237	1.739	5462	0.420596303	isogroup01260	ENSG00000168137	ENST00000402198	SETD5
isotig10428	3.471	2.007	2.388	1.845	4981	0.437307432	isogroup01260	ENSG00000168137	ENST00000402198	SETD5
isotig10429	2.051	1.259	1.656	1.063	1573	0.359557	isogroup01260	ENSG00000168137	ENST00000402466	SETD5
isotig10431	2.528	1.529	2.092	1.251	1092	0.435879612	isogroup01260	ENSG00000168137	ENST00000402466	SETD5
isotig10433	6.972	7.452	6.264	7.951	2673	0.195198016	isogroup01261	ENSG00000136709	ENST00000322313	WDR33
isotig10435	2.335	2.161	1.765	1.858	2546	0.100435861	isogroup01261	ENSG00000136709	ENST00000409658	WDR33
isotig10437	5.964	6.141	5.13	6.644	2562	0.164048997	isogroup01261	ENSG00000136709	ENST00000322313	WDR33
isotig10439	3.922	3.366	5.252	4.162	2804	0.275944991	isogroup01262	ENSG00000139613	ENST00000550164	SMARCC2
isotig10440	3.82	3.341	5.133	4.063	2713	0.263395206	isogroup01262	ENSG00000139613	ENST00000550164	SMARCC2
isotig10441	3.991	3.288	5.281	4.098	2545	0.336054332	isogroup01262	ENSG00000139613	ENST00000550164	SMARCC2
isotig10442	3.881	3.258	5.151	3.987	2454	0.326623206	isogroup01262	ENSG00000139613	ENST00000550164	SMARCC2
isotig10443	3.3	2.749	4.293	3.571	2423	0.238079582	isogroup01262	ENSG00000139613	ENST00000550164	SMARCC2
isotig10444	3.157	2.696	4.117	3.434	2332	0.222110543	isogroup01262	ENSG00000139613	ENST00000550164	SMARCC2
isotig10445	3.952	5.107	4.993	4.23	3016	0.081930563	isogroup01263	ENSG00000136450	ENST00000258962	SRSF1
isotig10446	3.941	4.842	4.939	4.283	2817	0.042524611	isogroup01263	ENSG00000136450	ENST00000258962	SRSF1
isotig10447	3.391	4.011	4.204	3.675	2255	0.003503795	isogroup01263	ENSG00000136450	ENST00000258962	SRSF1
isotig10448	5.008	6.23	6.074	4.947	2077	0.0314684	isogroup01263	ENSG00000136450	ENST00000258962	SRSF1
isotig10449	5.103	5.953	6.108	5.101	1878	0.045004509	isogroup01263	ENSG00000136450	ENST00000258962	SRSF1
isotig10450	4.657	5.002	5.346	4.409	1316	0.143448937	isogroup01263	ENSG00000136450	ENST00000258962	SRSF1
isotig10451	8.308	7.66	9.616	9.07	2467	0.020365221	isogroup01264	ENSG00000187642	ENST00000433179	C1orf170
isotig10452	9.245	8.729	10.802	10.49	2212	0.052049673	isogroup01264	ENSG00000187642	ENST00000433179	C1orf170
isotig10453	8.988	8.634	10.941	10.502	2080	0.050715789	isogroup01264	ENSG00000187642	ENST00000433179	C1orf170
isotig10454	10.494	10.31	12.926	12.755	1896	0.096302698	isogroup01264	ENSG00000187642	ENST00000433179	C1orf170
isotig10455	10.218	10.066	12.564	12.423	1825	0.111313594	isogroup01264	ENSG00000187642	ENST00000433179	C1orf170
isotig10456	12.096	12.164	15.04	15.242	1641	0.157041407	isogroup01264	ENSG00000187642	ENST00000433179	C1orf170
isotig10463	23.022	13.591	20.117	15.306	2716	0.67320771	isogroup01266	ENSG00000177951	ENST00000382762	BET1L
isotig10465	1.656	2.528	5.688	3.934	1399	0.226102803	isogroup01266	ENSG00000177951	ENST00000382762	BET1L
isotig10467	1.769	2.645	5.696	4.05	1287	0.196165552	isogroup01266	ENSG00000177951	ENST00000382762	BET1L
isotig10469	1.344	1.926	1.848	2.003	2345	0.297972871	isogroup01267	ENSG00000154079	ENST00000370474	C6orf57

isotig10471	4.404	3.168	3.938	3.585	2115	0.335866461	isogroup01267	ENSG00000154079	ENST00000370474	C6orf57
isotig10481	2.328	2.561	2.712	2.439	2253	0.014428496	isogroup01269	ENSG00000088766	ENST00000378863	CRLS1
isotig10482	2.043	2.251	2.408	2.029	2123	0.050227324	isogroup01269	ENSG00000088766	ENST00000378863	CRLS1
isotig10483	2.69	2.967	3.152	2.826	1923	0.014428496	isogroup01269	ENSG00000088766	ENST00000378863	CRLS1
isotig10484	2.378	2.63	2.824	2.369	1793	0.054116255	isogroup01269	ENSG00000088766	ENST00000378863	CRLS1
isotig10487	49.858	28.24	40.971	33.178	2168	0.697865785	isogroup01270	ENSG00000143761	ENST00000540651	ARF1
isotig10488	73.445	41.798	60.646	49.308	1743	0.703717968	isogroup01270	ENSG00000143761	ENST00000540651	ARF1
isotig10489	6.386	3.681	4.536	4.099	1559	0.558775457	isogroup01270	ENSG00000165527	ENST00000298316	ARF6
isotig10491	25.739	14.973	20.732	17.698	1134	0.682338243	isogroup01270	ENSG00000165527	ENST00000298316	ARF6
isotig10499	5.058	1.697	3.073	1.932	2015	0.771915157	isogroup01272	ENSG00000214114	ENST00000397572	MYCBP
isotig10500	6.123	1.818	3.608	2.19	1646	0.818864132	isogroup01272	ENSG00000214114	ENST00000397572	MYCBP
isotig10501	6.273	1.82	3.668	2.161	1581	0.826294432	isogroup01272	ENSG00000214114	ENST00000397572	MYCBP
isotig10505	8.883	17.967	7.061	6.931	2147	0.662996919	isogroup01273	ENSG00000120915	ENST00000521400	EPHX2
isotig10507	4.343	6.177	2.554	2.499	1099	0.286024273	isogroup01273	ENSG00000120915	ENST00000518379	EPHX2
isotig10509	4.104	6.19	2.237	2.338	897	0.383379049	isogroup01273	ENSG00000120915	ENST00000518379	EPHX2
isotig10511	8.129	4.464	7.003	8.351	1553	0.399263546	isogroup01274	ENSG00000104870	ENST00000426395	FCGRT
isotig10512	8.909	5.005	7.713	9.051	1444	0.385173217	isogroup01274	ENSG00000104870	ENST00000426395	FCGRT
isotig10513	7.229	3.909	6.221	7.377	1448	0.41048884	isogroup01274	ENSG00000104870	ENST00000426395	FCGRT
isotig10514	8.43	4.757	7.365	8.81	1429	0.381650635	isogroup01274	ENSG00000104870	ENST00000426395	FCGRT
isotig10515	7.997	4.447	6.922	8.053	1339	0.398784474	isogroup01274	ENSG00000104870	ENST00000426395	FCGRT
isotig10516	7.469	4.174	6.539	7.781	1324	0.393571053	isogroup01274	ENSG00000104870	ENST00000426395	FCGRT
isotig10517	214.79	1408.693	937.088	210.383	1311	0.44766852	isogroup01275	ENSG00000166452	ENST00000534147	AKIP1
isotig10519	192.548	1245.026	835.535	185.881	1248	0.422136845	isogroup01275	ENSG00000166452	ENST00000534147	AKIP1
isotig10520	15.092	11.342	15.698	12.476	1097	0.527015856	isogroup01275	ENSG00000166452	ENST00000534147	AKIP1
isotig10522	440.839	2958.899	1969.421	434.772	601	0.448100624	isogroup01275	ENSG00000254827	ENST00000533594	SLC22A18AS
isotig10523	3.411	4.068	4.016	3.864	1577	0.131678816	isogroup01276	ENSG00000173085	ENST00000311469	COQ2
isotig10524	3.608	4.23	4.223	4.103	1537	0.124520929	isogroup01276	ENSG00000173085	ENST00000439031	COQ2
isotig10525	3.547	4.152	4.401	4.072	1370	0.090957767	isogroup01276	ENSG00000173085	ENST00000439031	COQ2
isotig10526	2.265	2.341	2.189	2.306	1296	0.057310062	isogroup01276	ENSG00000173085	ENST00000311469	COQ2
isotig10527	2.47	2.484	2.384	2.549	1256	0.050227324	isogroup01276	ENSG00000173085	ENST00000439031	COQ2
isotig10528	2.219	2.118	2.326	2.272	1089	0.025447133	isogroup01276	ENSG00000173085	ENST00000503915	COQ2
isotig10535	4.386	3.735	5.014	3.193	2009	0.410244608	isogroup01278	ENSG00000124535	ENST00000380773	WRNIP1
isotig10536	4.421	3.523	4.837	3.064	1929	0.456667543	isogroup01278	ENSG00000124535	ENST00000380773	WRNIP1
isotig10537	3.214	2.605	3.615	2.373	1640	0.391213271	isogroup01278	ENSG00000124535	ENST00000380773	WRNIP1
isotig10538	4.314	3.842	5.029	3.056	917	0.415852559	isogroup01278	ENSG00000124535	ENST00000380764	WRNIP1
isotig10546	1.585	1.413	2.206	1.482	1796	0.312213497	isogroup01280	ENSG00000100263	ENST00000414672	RHBDD3
isotig10547	2.128	2.041	2.852	2.231	1281	0.271248215	isogroup01280	ENSG00000100263	ENST00000414672	RHBDD3
isotig10548	1.714	1.67	2.285	1.64	1241	0.267650485	isogroup01280	ENSG00000100263	ENST00000414672	RHBDD3
isotig10549	1.478	1.477	1.835	1.373	1173	0.195780416	isogroup01280	ENSG00000100263	ENST00000414672	RHBDD3
isotig10550	2.453	2.748	2.801	2.745	658	0.021652138	isogroup01280	ENSG00000100263	ENST00000414672	RHBDD3
isotig10551	1.642	2.049	1.66	1.591	618	0.041209514	isogroup01280	ENSG00000100263	ENST00000414672	RHBDD3
isotig10564	3.261	4.155	4.392	4.25	1013	0.242466371	isogroup01283	ENSG00000170873	ENST00000325064	MTSS1
isotig10565	3.389	4.475	4.69	4.528	959	0.299494627	isogroup01283	ENSG00000170873	ENST00000325064	MTSS1
isotig10570	4.577	3.177	2.123	2.854	1306	0.176260615	isogroup01284	ENSG00000197343	ENST00000320583	ZNF655
isotig10574	2.718	1.1	1.155	1.205	458	0.434517547	isogroup01284	ENSG00000197343	ENST00000454654	ZNF655
isotig10576	6.662	7.129	10.001	12.839	769	0.203717968	isogroup01285	ENSG00000124104	ENST00000491381	SNX21
isotig10579	7.794	8.642	11.93	15.569	729	0.290683475	isogroup01285	ENSG00000124104	ENST00000462307	SNX21
isotig10594	20.601	17.39	25.146	14.732	631	0.57708349	isogroup01288	ENSG00000133142	ENST00000372629	TCEAL4
isotig10596	17.591	14.205	18.952	10.395	565	0.609960923	isogroup01288	ENSG00000133142	ENST00000372629	TCEAL4
contig15945	10.541	7.433	8.327	5.23	612	0.652053431	isogroup01290	ENSG00000138398	ENST00000530133	PPIG
contig15950	10.842	7.475	8.278	4.826	1925	0.635793192	isogroup01290	ENSG00000138398	ENST00000448752	PPIG
contig15952	31.34	39.862	49.11	42.333	1284	0.056521004	isogroup01291	ENSG00000122643	ENST00000405342	NT5C3
contig15957	32.753	34.45	44.406	39.25	689	0.119006914	isogroup01292	ENSG00000108671	ENST00000261712	PSMD11
contig15967	10.281	8.827	10.449	10.953	744	0.166350417	isogroup01293	ENSG00000143771	ENST00000465271	CNIH4
contig15972	905.835	547.779	608.916	537.204	569	0.658290749	isogroup01294	ENSG00000140988	ENST00000343262	RPS2
contig15975	1.202	1.56	1.236	1.946	598	0.289180507	isogroup01295	ENSG00000117707	ENST00000541470	PROX1
isotig10605	5.398	5.277	4.241	3.302	7676	0.351788532	isogroup01297	ENSG00000068878	ENST00000404125	PSME4
isotig10606	5.435	5.313	4.271	3.324	7624	0.352990907	isogroup01297	ENSG00000068878	ENST00000404125	PSME4
isotig10607	5.536	5.444	4.393	3.478	7171	0.336561584	isogroup01297	ENSG00000068878	ENST00000404125	PSME4
isotig10608	5.577	5.483	4.426	3.503	7119	0.336561584	isogroup01297	ENSG00000068878	ENST00000404125	PSME4
isotig10609	3.541	3.607	1.959	2.11	4830	0.109575787	isogroup01297	ENSG00000068878	ENST00000404125	PSME4
isotig10610	8.328	6.697	9.527	9.536	7313	0.229897798	isogroup01298	ENSG00000099968	ENST00000317582	BCL2L13
isotig10612	8.243	6.552	9.333	9.372	7219	0.229634779	isogroup01298	ENSG00000099968	ENST00000317582	BCL2L13
isotig10614	10.438	8.497	9.6	10.547	5504	0.136986173	isogroup01299	ENSG00000198961	ENST00000361557	PJA2
isotig10694	2.453	1.327	2.445	2.018	1431	0.411343654	isogroup01315	ENSG00000168427	ENST00000409223	KLHL30
isotig10695	3.171	2.091	2.666	2.944	1247	0.304463816	isogroup01315	ENSG00000168427	ENST00000409223	KLHL30
isotig10696	3.655	3.438	4.711	3.563	3406	0.296732923	isogroup01316	ENSG00000127483	ENST00000312239	HP1BP3
isotig10697	3.667	3.349	4.637	3.55	3207	0.307591869	isogroup01316	ENSG00000127483	ENST00000312239	HP1BP3
isotig10698	2.659	2.507	3.241	2.599	1831	0.229813256	isogroup01316	ENSG00000127483	ENST00000312239	HP1BP3
isotig10699	2.561	2.218	2.918	2.456	1632	0.246740437	isogroup01316	ENSG00000127483	ENST00000312239	HP1BP3
isotig10701	26.422	30.914	33.153	18.452	3153	0.429360487	isogroup01317	ENSG00000084754	ENST00000380649	HADHA
isotig10702	30.978	36.297	39.078	21.753	2709	0.432056436	isogroup01317	ENSG00000084754	ENST00000380649	HADHA
isotig10703	27.716	32.38	34.375	19.315	2099	0.418266702	isogroup01317	ENSG00000084754	ENST00000380649	HADHA
isotig10704	15.325	17.666	19.463	10.554	1540	0.45123807	isogroup01317	ENSG00000084754	ENST00000380649	HADHA
isotig10705	22.089	25.603	28.56	15.514	1096	0.460481326	isogroup01317	ENSG00000084754	ENST00000380649	HADHA
isotig10706	12.279	9.605	11.706	8.689	2506	0.467122567	isogroup01318	ENSG00000163902	ENST00000296255	RPN1
isotig10707	13.72	10.786	13.148	9.751	2317	0.471847524	isogroup01318	ENSG00000163902	ENST00000296255	RPN1
isotig10708	8.801	6.805	8.199	5.974	2179	0.465760502	isogroup01318	ENSG00000163902	ENST00000296255	RPN1
isotig10709	10.149	7.915	9.546	6.952	1990	0.465478695	isogroup01318	ENSG00000163902	ENST00000296255	RPN1
isotig10710	11.106	7.667	10.443	7.156	1266	0.611679943	isogroup01318	ENSG00000163902	ENST00000545956	RPN1
isotig10711	2.855	2.842	3.683	3.573	3156	0.006660029	isogroup01319	ENSG00000204852	ENST00000551590	TCTN1
isotig10712	3.397	3.405	4.304	4.311	2468	0.018711956	isogroup01319	ENSG00000204852	ENST00000551590	TCTN1

isotig10713	1.226	1.283	1.865	1.103	1842	0.124257909	isogroup01319	ENSG00000122986	ENST00000356742	HVCN1
isotig10715	1.415	1.559	2.108	1.208	1154	0.133698429	isogroup01319	ENSG00000204852	ENST00000551590	TCTN1
isotig10716	1.668	1.722	1.607	1.124	2647	0.147018487	isogroup01320	ENSG00000083544	ENST00000535286	TDRD3
isotig10717	1.702	1.767	1.647	1.151	2594	0.141016007	isogroup01320	ENSG00000083544	ENST00000535286	TDRD3
isotig10718	1.765	1.837	1.744	1.302	2508	0.128945292	isogroup01320	ENSG00000083544	ENST00000535286	TDRD3
isotig10725	9.764	11.299	7.302	7.801	2542	0.347777486	isogroup01322	ENSG00000167978	ENST00000382301	SRRM2
isotig10726	9.926	11.655	7.391	8.089	2457	0.374483355	isogroup01322	ENSG00000167978	ENST00000382301	SRRM2
isotig10727	9.886	10.563	7.155	8.097	2247	0.285648531	isogroup01322	ENSG00000167978	ENST00000382301	SRRM2
isotig10728	10.075	10.939	7.251	8.437	2162	0.324866612	isogroup01322	ENSG00000167978	ENST00000382301	SRRM2
isotig10729	16.082	18.638	19.059	15.007	1944	0.100229203	isogroup01323	ENSG00000167978	ENST00000544933	SRRM2
isotig10731	17.706	20.879	21.481	16.853	1852	0.086082513	isogroup01323	ENSG00000142279	ENST00000270288	WTIP
isotig10733	13.028	16.962	12.822	10.207	1188	0.140969039	isogroup01323	ENSG00000167978	ENST00000544933	SRRM2
isotig10734	4.699	3.866	5.594	4.451	2470	0.339586308	isogroup01324	ENSG00000160741	ENST00000368633	CRTC2
isotig10735	3.602	2.862	3.862	3.324	1947	0.315801834	isogroup01324	ENSG00000160741	ENST00000368633	CRTC2
isotig10736	3.793	3.196	3.802	3.254	1894	0.313970091	isogroup01324	ENSG00000160741	ENST00000368633	CRTC2
isotig10737	3.995	3.301	5.122	3.745	1662	0.34482791	isogroup01324	ENSG00000160741	ENST00000368633	CRTC2
isotig10738	1.797	1.324	1.946	1.493	1139	0.259966559	isogroup01324	ENSG00000160741	ENST00000461638	CRTC2
isotig10739	3.638	2.634	2.91	2.264	2026	0.388470354	isogroup01325	ENSG00000146457	ENST00000358372	WTAP
isotig10740	3.949	2.894	3.21	2.49	2008	0.389109116	isogroup01325	ENSG00000146457	ENST00000358372	WTAP
isotig10741	3.738	3.82	3.554	3.381	1652	0.064082814	isogroup01325	ENSG00000146457	ENST00000337387	WTAP
isotig10742	4.122	4.153	3.93	3.672	1634	0.077571955	isogroup01325	ENSG00000146457	ENST00000337387	WTAP
isotig10743	3.517	2.24	2.772	1.777	1541	0.509243255	isogroup01325	ENSG00000146457	ENST00000358372	WTAP
isotig10744	4.716	3.705	5.474	5.363	1987	0.169685128	isogroup01326	ENSG00000186187	ENST00000567962	ZNRF1
isotig10745	12.121	8.847	13.868	13.656	1854	0.212087623	isogroup01326	ENSG00000186187	ENST00000567962	ZNRF1
isotig10747	8.267	6.372	9.682	9.356	1560	0.162433306	isogroup01326	ENSG00000186187	ENST00000567962	ZNRF1
isotig10748	6.178	4.41	7.445	7.036	1462	0.281496581	isogroup01326	ENSG00000186187	ENST00000567962	ZNRF1
isotig10749	356.298	312.257	398.599	263.198	1684	0.548489517	isogroup01327	ENSG00000169714	ENST00000422453	CNBP
isotig10757	1.538	2.269	1.766	1.033	2223	0.155397535	isogroup01329	ENSG00000169902	ENST00000304842	TPST1
isotig10758	6.001	4.44	11.976	7.902	1596	0.623778838	isogroup01329	ENSG00000168734	ENST00000372889	PKIG
isotig10759	4.997	3.825	10.11	6.712	1517	0.595494852	isogroup01329	ENSG00000168734	ENST00000372889	PKIG
isotig10760	7.156	5.278	14.287	9.426	1336	0.637286766	isogroup01329	ENSG00000168734	ENST00000372889	PKIG
isotig10761	6.018	4.588	12.181	8.086	1257	0.612271737	isogroup01329	ENSG00000168734	ENST00000372889	PKIG
isotig10764	1.707	1.349	2.018	1.805	1249	0.2497088	isogroup01330	ENSG00000102390	ENST00000373358	CXorf26
isotig10765	2.442	1.896	2.777	2.607	1024	0.309977831	isogroup01330	ENSG00000102390	ENST00000373358	CXorf26
isotig10767	7.317	5.768	6.876	6.141	2212	0.328877658	isogroup01331	ENSG00000175203	ENST00000537439	DCTN2
isotig10768	8.47	6.741	7.92	7.185	1987	0.30320508	isogroup01331	ENSG00000175203	ENST00000434715	DCTN2
isotig10769	10.084	7.883	9.364	8.512	1931	0.33451379	isogroup01331	ENSG00000175203	ENST00000434715	DCTN2
isotig10770	11.791	9.297	10.907	10.042	1706	0.316356053	isogroup01331	ENSG00000175203	ENST00000434715	DCTN2
isotig10776	6.45	5.885	6.749	4.699	2179	0.378353498	isogroup01333	ENSG00000159840	ENST00000322764	ZYX
isotig10777	5.306	4.841	5.656	3.894	2058	0.354709927	isogroup01333	ENSG00000159840	ENST00000322764	ZYX
isotig10778	4.893	4.337	5.175	3.374	2004	0.382186067	isogroup01333	ENSG00000159840	ENST00000322764	ZYX
isotig10779	5.485	4.622	5.147	3.652	873	0.414809875	isogroup01333	ENSG00000159840	ENST00000449423	ZYX
isotig10780	4.092	3.502	3.888	3.009	765	0.303017209	isogroup01333	ENSG00000146067	ENST00000514747	FAM193B
isotig10781	3.995	7.25	3.941	6.062	2011	0.650090178	isogroup01334	ENSG00000162413	ENST00000377663	KLHL21
isotig10782	4.84	10.148	5.458	8.421	1840	0.746881341	isogroup01334	ENSG00000162413	ENST00000377658	KLHL21
isotig10783	2.499	3.678	2.247	2.843	1744	0.379790712	isogroup01334	ENSG00000162413	ENST00000377663	KLHL21
isotig10784	3.325	6.68	3.837	5.254	1573	0.661446983	isogroup01334	ENSG00000162413	ENST00000377658	KLHL21
isotig10785	4.23	8.626	5.862	6.25	609	0.563171639	isogroup01334	ENSG00000162413	ENST00000377658	KLHL21
isotig10786	1.908	1.136	2.141	1.697	2441	0.361642369	isogroup01335	ENSG00000169220	ENST00000511890	RGS14
isotig10788	1.683	1.096	1.8	1.776	1506	0.230001127	isogroup01335	ENSG00000169220	ENST00000511890	RGS14
isotig10791	3.857	2.695	3.751	3.032	2260	0.435362967	isogroup01336	ENSG00000177042	ENST00000526170	TMEM80
isotig10792	2.573	1.45	2.197	1.909	1733	0.370754114	isogroup01336	ENSG00000177042	ENST00000526170	TMEM80
isotig10793	3.736	3.059	3.887	2.98	1537	0.405031187	isogroup01336	ENSG00000177042	ENST00000526170	TMEM80
isotig10794	4.644	3.485	4.747	3.609	1032	0.471819343	isogroup01336	ENSG00000103404	ENST00000564896	USP31
isotig10796	1.73	1.833	1.512	1.526	2113	0.021652138	isogroup01337	ENSG00000163322	ENST00000321945	FAM175A
isotig10803	105.442	104.199	132.361	124.034	1830	0.041078004	isogroup01338	ENSG00000166411	ENST00000299518	IDH3A
isotig10804	115.364	114.474	145.629	136.037	1737	0.041078004	isogroup01338	ENSG00000166411	ENST00000299518	IDH3A
isotig10805	4.944	5.16	8.005	5.186	1826	0.416425565	isogroup01339	ENSG00000116990	ENST00000429311	MYCL1
isotig10806	4.254	3.467	5.053	3.923	1629	0.440548208	isogroup01339	ENSG00000167965	ENST00000565250	MLST8
isotig11276	318.885	274.446	397.795	292.533	1369	0.516062974	isogroup01462	ENSG00000163541	ENST00000393868	SUCLG1
isotig11278	34.06	23.728	50.235	36.058	1095	0.69663523	isogroup01463	ENSG00000153531	ENST00000375418	ADPRHL1
isotig11279	17.848	12.227	26.019	17.868	858	0.704760652	isogroup01463	ENSG00000153531	ENST00000375418	ADPRHL1
isotig11280	24.471	17.031	35.577	26.467	833	0.675884873	isogroup01463	ENSG00000153531	ENST00000375418	ADPRHL1
isotig11282	379.988	369.781	360.68	428.233	1331	0.138413993	isogroup01464	ENSG00000122678	ENST00000414235	POLM
isotig11288	6.14	5.89	4.225	4.04	1488	0.0314684	isogroup01466	ENSG00000169139	ENST00000523111	UBE2V2
isotig11289	4.758	4.381	3.328	3.032	1362	0.054877132	isogroup01466	ENSG00000169139	ENST00000523111	UBE2V2
isotig11290	9.254	4.136	8.46	3.436	1597	0.871129856	isogroup01467	ENSG00000226976	ENST00000423336	COX6A1P2
isotig11291	29.873	14.203	23.234	11.262	715	0.892622304	isogroup01467	ENSG00000226976	ENST00000423336	COX6A1P2
isotig11292	36.925	19.623	27.807	14.582	533	0.86011122	isogroup01467	ENSG00000226976	ENST00000423336	COX6A1P2
isotig11293	8.229	5.155	7.501	4.378	864	0.674287969	isogroup01468	ENSG00000147789	ENST00000544249	ZNF7
isotig11297	3.431	4.753	5.627	4.742	845	0.144688886	isogroup01469	ENSG00000124357	ENST00000455662	NAGK
isotig11298	1.998	2.908	3.452	3.282	805	0.21826858	isogroup01469	ENSG00000124357	ENST00000524537	NAGK
isotig11299	3.115	4.303	4.56	4.711	575	0.296958368	isogroup01469	ENSG00000124357	ENST00000455662	NAGK
isotig11300	2.773	3.504	3.864	3.254	516	0.09210378	isogroup01469	ENSG00000124357	ENST00000455662	NAGK
isotig11305	6.835	5.935	5.848	5.778	772	0.126202375	isogroup01471	ENSG00000141385	ENST00000537174	AFG3L2
isotig11306	7.782	7.52	7.267	7.531	648	0.033563162	isogroup01471	ENSG00000141385	ENST00000537174	AFG3L2
isotig11307	4.93	4.287	3.964	4.121	645	0.079262794	isogroup01471	ENSG00000141385	ENST00000537174	AFG3L2
isotig11308	5.655	5.864	5.28	5.908	521	0.151574359	isogroup01471	ENSG00000141385	ENST00000537174	AFG3L2
isotig11317	15.893	19.035	16.659	17.053	1043	0.217601638	isogroup01474	ENSG00000106603	ENST00000446330	C7orf44
isotig11318	18.501	22.968	20.457	21.021	992	0.23556211	isogroup01474	ENSG00000106603	ENST00000446330	C7orf44
isotig11323	8.247	7.361	7.147	5.147	762	0.390198768	isogroup01476	ENSG00000187145	ENST00000369084	MRPS21
isotig11324	10.558	9.938	9.248	6.991	529	0.323495153	isogroup01476	ENSG00000187145	ENST00000369084	MRPS21

isotig11325	11.597	10.335	9.694	7.233	516	0.397234538	isogroup01476	ENSG00000187145	ENST00000369084	MRPS21
isotig11326	2.744	1.875	2.172	1.328	472	0.415711656	isogroup01476	ENSG00000187145	ENST00000369084	MRPS21
isotig11337	206.812	540.692	287.249	294.626	672	0.884083565	isogroup01480	ENSG00000148180	ENST00000545652	GSN
isotig11345	16.485	12.981	16.06	14.634	719	0.414368378	isogroup01483	ENSG00000205155	ENST00000222266	PSENEN
isotig11346	20.692	16.731	20.192	18.048	650	0.421535658	isogroup01483	ENSG00000205155	ENST00000222266	PSENEN
isotig11347	10.341	8.751	9.937	10.406	7255	0.021802435	isogroup01484	ENSG00000070182	ENST00000389723	SPTB
isotig11348	10.617	9.134	10.277	10.852	7139	0.065529421	isogroup01484	ENSG00000070182	ENST00000389723	SPTB
isotig11349	9.276	7.998	8.558	9.177	6876	0.080568498	isogroup01484	ENSG00000070182	ENST00000542895	SPTB
isotig11350	2.902	2.961	2.017	3.07	8161	0.286700609	isogroup01485	ENSG00000100731	ENST00000304743	PCNX
isotig11351	2.884	3.015	2.021	3.122	8110	0.303937777	isogroup01485	ENSG00000100731	ENST00000304743	PCNX
isotig11352	9.732	6.847	8.825	6.174	5649	0.587012475	isogroup01486	ENSG00000167615	ENST00000431846	LENG8
isotig11353	9.88	7.147	8.869	6.4	5254	0.558409108	isogroup01486	ENSG00000167615	ENST00000431846	LENG8
isotig11354	9.769	6.987	8.784	6.22	5052	0.572537011	isogroup01486	ENSG00000167615	ENST00000431846	LENG8
isotig11355	6.239	5.548	7.419	3.996	8982	0.585781919	isogroup01487	ENSG00000149091	ENST00000527911	DGKZ
isotig11356	7.158	5.606	8.614	4.617	4447	0.669563012	isogroup01487	ENSG00000149091	ENST00000527911	DGKZ
isotig11357	6.262	4.373	7.55	3.336	1548	0.745923198	isogroup01487	ENSG00000149091	ENST00000527911	DGKZ
isotig11358	2.399	1.765	2.261	2.028	6617	0.187185316	isogroup01488	ENSG00000090975	ENST00000280562	PITPNM2
isotig11359	2.431	1.78	2.301	2.049	6467	0.199706921	isogroup01488	ENSG00000090975	ENST00000280562	PITPNM2
isotig11360	2.151	1.837	1.789	1.889	1758	0.045840535	isogroup01488	ENSG00000090975	ENST00000542749	PITPNM2
isotig11361	2.736	2.409	2.548	2.547	5240	0.149996243	isogroup01489	ENSG00000168781	ENST00000381878	PPIP5K1
isotig11362	2.809	2.443	2.601	2.635	5090	0.149996243	isogroup01489	ENSG00000168781	ENST00000381878	PPIP5K1
isotig11363	2.343	2.155	2.48	2.342	1861	0.123215225	isogroup01489	ENSG00000168781	ENST00000420765	PPIP5K1
isotig11364	5.01	4.234	4.62	4.053	7078	0.153819418	isogroup01490	ENSG00000154305	ENST00000344922	MIA3
isotig11365	3.602	3.11	3.867	3.066	3939	0.247820696	isogroup01490	ENSG00000154305	ENST00000340535	MIA3
isotig11366	5.615	3.831	8.119	7	4976	0.458414744	isogroup01491	ENSG00000177728	ENST00000314256	KIAA0195
isotig11367	5.58	3.786	8.014	6.866	4910	0.465375366	isogroup01491	ENSG00000177728	ENST00000314256	KIAA0195
isotig11368	3.811	3.587	3.9	3.561	3503	0.119354475	isogroup01492	ENSG00000161533	ENST00000301608	ACOX1
isotig11369	3.885	3.516	3.976	3.535	3503	0.176147892	isogroup01492	ENSG00000161533	ENST00000539791	ACOX1
isotig11370	3.54	3.036	3.691	2.939	2443	0.241818216	isogroup01492	ENSG00000161533	ENST00000537812	ACOX1
isotig11371	2.421	2.173	2.823	2.262	3726	0.224674983	isogroup01493	ENSG00000167491	ENST00000404158	GATAD2A
isotig11372	2.402	2.22	2.813	2.302	3651	0.197396107	isogroup01493	ENSG00000167491	ENST00000404158	GATAD2A
isotig11373	1.842	1.636	2.252	1.616	1953	0.245725934	isogroup01493	ENSG00000167491	ENST00000404158	GATAD2A
isotig11374	3.324	2.095	2.495	1.949	4230	0.448899076	isogroup01494	ENSG00000133114	ENST00000497558	KIAA1704
isotig11375	2.519	1.612	2.337	1.816	3702	0.390461787	isogroup01494	ENSG00000133114	ENST00000497558	KIAA1704
isotig11376	2.184	1.312	1.463	1.46	1262	0.250385136	isogroup01494	ENSG00000133114	ENST00000497558	KIAA1704
isotig11377	3.63	6.498	5.331	5.245	3092	0.444164725	isogroup01495	ENSG00000177646	ENST00000308982	ACAD9
isotig11379	4.651	8.495	7.17	6.135	2310	0.408403472	isogroup01495	ENSG00000177646	ENST00000308982	ACAD9
isotig11383	7.92	6.3	8.186	7.975	3131	0.239507402	isogroup01497	ENSG00000135924	ENST00000336576	DNAJB2
isotig11384	7.823	6.484	8.433	8.038	2922	0.213365146	isogroup01497	ENSG00000135924	ENST00000336576	DNAJB2
isotig11385	9.368	8.828	10.044	10.086	2001	0.021407906	isogroup01497	ENSG00000135924	ENST00000392086	DNAJB2
isotig11386	10.455	11.309	10.532	8.509	3994	0.094151574	isogroup01498	ENSG00000008952	ENST00000480708	SEC62
isotig11388	16.42	16.78	24.932	20.442	3943	0.161475163	isogroup01499	ENSG00000108604	ENST00000448276	SMARCD2
isotig11389	15.042	15.236	22.882	18.258	3934	0.190388517	isogroup01499	ENSG00000108604	ENST00000448276	SMARCD2
isotig11390	12.896	7.287	12.382	9.329	3697	0.695263771	isogroup01500	ENSG00000063660	ENST00000264039	GPC1
isotig11391	13.492	7.702	13.012	9.878	3604	0.692004208	isogroup01500	ENSG00000063660	ENST00000264039	GPC1
isotig11393	17.627	16.895	23.293	16.818	3025	0.455606072	isogroup01501	ENSG00000109861	ENST00000227266	CTSC
isotig11395	5.071	3.297	5.997	3.294	1016	0.602154881	isogroup01501	ENSG00000109861	ENST00000529974	CTSC
isotig11398	8.53	6.745	7.675	5.551	3360	0.453764936	isogroup01503	ENSG00000257103	ENST00000433627	LSM14A
isotig11399	7.863	5.846	6.948	5.016	3183	0.498872774	isogroup01503	ENSG00000257103	ENST00000433627	LSM14A
isotig11400	14.373	12.168	12.635	12.837	3942	0.140969039	isogroup01504	ENSG00000102606	ENST00000375737	ARHGEF7
isotig11401	12.493	8.898	9.676	10.814	2537	0.314937627	isogroup01504	ENSG00000102606	ENST00000375741	ARHGEF7
isotig11402	13.291	26.975	19.175	23.585	3121	0.738305027	isogroup01505	ENSG00000112425	ENST00000367519	EPM2A
isotig11404	10.982	17.493	27.952	17.782	2987	0.070226197	isogroup01506	ENSG00000168509	ENST00000357836	HFE2
isotig11405	12.232	19.075	30.057	18.918	2881	0.101647629	isogroup01506	ENSG00000168509	ENST00000336751	HFE2
isotig11406	8.826	5.758	9.262	8.137	3084	0.450317502	isogroup01507	ENSG00000134046	ENST00000256429	MBD2
isotig11407	2.175	1.895	3.416	2.948	1492	0.122942812	isogroup01507	ENSG00000166828	ENST00000300061	SCNN1G
isotig11408	1.988	1.653	2.933	2.646	1341	0.106053205	isogroup01507	ENSG00000185252	ENST00000400451	ZNF74
isotig11412	16.936	11.915	15.578	11.149	5508	0.589745998	isogroup01509	ENSG00000116209	ENST00000234831	TMEM59
isotig11413	87.454	80.57	91.323	63.901	3424	0.306802811	isogroup01510	ENSG00000177469	ENST00000357037	PTRF
isotig11414	17.136	17.156	18.212	20.322	2011	0.218907342	isogroup01510	ENSG00000177469	ENST00000357037	PTRF
isotig11415	4.654	2.561	3.913	2.913	1985	0.582362666	isogroup01511	ENSG00000016864	ENST00000491606	GLT8D1
isotig11416	6.083	3.631	5.124	3.933	1678	0.598538363	isogroup01511	ENSG00000016864	ENST00000491606	GLT8D1
isotig11417	3.149	1.72	2.18	2.112	1196	0.459185015	isogroup01511	ENSG00000016864	ENST00000491606	GLT8D1
isotig11421	11.912	10.879	9.571	9.458	1721	0.096039678	isogroup01513	ENSG00000170035	ENST00000410062	UBE2E3
isotig11422	14.037	13.166	11.251	11.355	1464	0.052829338	isogroup01513	ENSG00000170035	ENST00000410062	UBE2E3
isotig11423	14.933	13.606	11.604	11.603	1453	0.098059292	isogroup01513	ENSG00000170035	ENST00000410062	UBE2E3
isotig11424	20.821	32.486	19.646	18.452	2196	0.471856917	isogroup01514	ENSG00000198721	ENST00000380118	ECI2
isotig11425	24.623	37.027	22.521	20.403	2037	0.416641617	isogroup01514	ENSG00000198721	ENST00000380118	ECI2
isotig11426	27.563	24.758	27.972	28.769	2115	0.132157887	isogroup01515	ENSG00000169733	ENST00000429557	RFNG
isotig11427	31.373	28.312	31.496	32.398	1988	0.118424513	isogroup01515	ENSG00000169733	ENST00000429557	RFNG
isotig11428	46.306	62.206	63.54	60.065	1727	0.290523784	isogroup01516	ENSG00000105953	ENST00000444676	OGDH
isotig11429	37.977	51.296	52.89	49.357	1598	0.259158713	isogroup01516	ENSG00000105953	ENST00000222673	OGDH
isotig11430	59.09	81.658	93.253	77.687	655	0.057131585	isogroup01516	ENSG00000204099	ENST00000415936	NEU4
isotig11431	4431.109	3638.013	3892.378	4197.996	2042	0.058474863	isogroup01517	ENSG00000198467	ENST00000360958	TPM2
isotig11432	3157.223	2806.589	2950.801	3142.012	999	0.008623281	isogroup01517	ENSG00000198467	ENST00000378292	TPM2
isotig11433	757.224	670.258	672.789	759.42	880	0.008613887	isogroup01517	ENSG00000198467	ENST00000378300	TPM2
isotig11434	6.512	8.116	7.473	6.949	1352	0.089144811	isogroup01518	ENSG00000185267	ENST00000465530	CDNF
isotig11435	5.593	6.918	6.402	5.797	1333	0.073692418	isogroup01518	ENSG00000185267	ENST00000465530	CDNF
isotig11436	4.944	6.421	6.257	5.713	1082	0.086167055	isogroup01518	ENSG00000185267	ENST00000465530	CDNF
isotig11437	3.344	4.213	3.7	3.641	1731	0.209814383	isogroup01519	ENSG00000186994	ENST00000330915	KANK3
isotig11842	1.885	1.334	1.515	1.346	3136	0.235458781	isogroup01631	ENSG00000134283	ENST00000552761	PPHLN1
isotig11843	3.889	4.348	2.705	2.991	5164	0.230001127	isogroup01632	ENSG00000197601	ENST00000354817	FAR1

isotig11845	3.931	4.457	2.124	2.674	3269	0.294497257	isogroup01632	ENSG00000197601	ENST00000354817	FAR1
isotig11847	6.354	7.344	5.327	4.846	5515	0.072048546	isogroup01633	ENSG00000185722	ENST00000535427	ANKFY1
isotig11848	6.138	7.189	5.503	4.873	4216	0.059639663	isogroup01633	ENSG00000185722	ENST00000535427	ANKFY1
isotig11849	4.884	5.841	3.37	3.482	2207	0.164171113	isogroup01633	ENSG00000185722	ENST00000535427	ANKFY1
isotig11850	1.776	2.971	1.388	1.659	908	0.329863981	isogroup01633	ENSG00000185722	ENST00000535427	ANKFY1
isotig11851	4.114	6.867	5.309	5.219	4302	0.533563162	isogroup01634	ENSG00000143374	ENST00000369064	TARS2
isotig11852	4.134	6.798	5.287	5.144	4245	0.518561659	isogroup01634	ENSG00000143374	ENST00000369064	TARS2
isotig11853	3.019	9.866	4.806	6.243	2019	0.871637108	isogroup01634	ENSG00000143369	ENST00000369047	ECM1
isotig11854	3.031	9.803	4.743	6.11	1962	0.870913805	isogroup01634	ENSG00000143369	ENST00000369047	ECM1
isotig11855	4.326	3.324	2.202	3.561	3145	0.084044112	isogroup01635	ENSG00000134775	ENST00000445677	FHOD3
isotig11856	4.17	3.265	2.138	3.503	3109	0.113323815	isogroup01635	ENSG00000134775	ENST00000445677	FHOD3
isotig11857	4.328	3.345	2.214	3.563	3077	0.09210378	isogroup01635	ENSG00000134775	ENST00000359247	FHOD3
isotig11858	4.169	3.284	2.15	3.504	3041	0.115456151	isogroup01635	ENSG00000134775	ENST00000359247	FHOD3
isotig11859	8.04	5.755	5.46	5.717	3267	0.100473435	isogroup01636	ENSG00000172037	ENST00000418109	LAMB2
isotig11860	8.109	6.091	5.474	6.036	3147	0.017143233	isogroup01636	ENSG00000172037	ENST00000418109	LAMB2
isotig11861	8.221	6.424	5.674	6.189	2994	0.048837078	isogroup01636	ENSG00000172037	ENST00000418109	LAMB2
isotig11862	8.304	6.82	5.699	6.559	2874	0.15818742	isogroup01636	ENSG00000172037	ENST00000418109	LAMB2
isotig11863	1.444	1.497	3.205	2.346	5358	0.260389269	isogroup01637	ENSG00000243244	ENST00000404752	STON1
isotig11864	1.406	1.48	3.382	2.145	3658	0.323617269	isogroup01637	ENSG00000243244	ENST00000404752	STON1
isotig11865	1.207	1.26	2.196	2.079	2342	0.10352634	isogroup01637	ENSG00000068781	ENST00000402114	STON1-GTF2A1L
isotig11867	3.186	4.029	3.123	3.091	3098	0.192417525	isogroup01638	ENSG00000085760	ENST00000403721	MTIF2
isotig11868	3.014	3.692	2.905	2.797	3007	0.140846923	isogroup01638	ENSG00000085760	ENST00000403721	MTIF2
isotig11869	3.004	3.673	2.996	2.742	2937	0.110749981	isogroup01638	ENSG00000085760	ENST00000403721	MTIF2
isotig11870	2.893	3.453	2.851	2.57	2846	0.06991621	isogroup01638	ENSG00000085760	ENST00000403721	MTIF2
isotig11871	1.705	3.055	3.68	3.453	4873	0.145271286	isogroup01639	ENSG00000189369	ENST00000502175	GSPT2
isotig11872	2.812	5.558	7.137	6.897	2500	0.245312617	isogroup01639	ENSG00000186207	ENST00000334269	LCE5A
isotig11873	1.717	3.049	3.888	3.46	2385	0.138291876	isogroup01639	ENSG00000186207	ENST00000334269	LCE5A
isotig11874	1.617	2.932	3.645	3.361	2149	0.145271286	isogroup01639	ENSG00000186207	ENST00000334269	LCE5A
isotig11879	6.008	2.197	4.825	4.858	4449	0.693103254	isogroup01641	ENSG00000142661	ENST00000374434	MYOM3
isotig11880	4.458	1.996	3.898	4.325	3266	0.525137146	isogroup01641	ENSG00000142661	ENST00000374434	MYOM3
isotig11881	28.346	9.736	24.671	24.724	2605	0.819427745	isogroup01641	ENSG00000142661	ENST00000330966	MYOM3
isotig11882	43.368	15.547	39.053	40.027	1422	0.804153829	isogroup01641	ENSG00000142661	ENST00000330966	MYOM3
isotig11883	3.174	2.951	4.745	4.597	3011	0.157003833	isogroup01642	ENSG00000143365	ENST00000356728	RORC
isotig11884	3.353	3.085	5.077	4.864	2972	0.179491997	isogroup01642	ENSG00000143365	ENST00000318247	RORC
isotig11885	2.927	2.667	4.306	4.247	2878	0.148117532	isogroup01642	ENSG00000143365	ENST00000356728	RORC
isotig11886	3.111	2.804	4.649	4.522	2839	0.165260765	isogroup01642	ENSG00000143365	ENST00000318247	RORC
isotig11887	7.462	6.312	8.949	7.442	2966	0.327421658	isogroup01643	ENSG00000132383	ENST00000254719	RPA1
isotig11888	7.713	6.481	9.236	7.672	2915	0.339727211	isogroup01643	ENSG00000132383	ENST00000254719	RPA1
isotig11889	7.071	6.105	8.725	7.173	2873	0.324951154	isogroup01643	ENSG00000132383	ENST00000254719	RPA1
isotig11890	7.323	6.276	9.016	7.405	2822	0.337256707	isogroup01643	ENSG00000132383	ENST00000254719	RPA1
isotig11891	6.132	5.751	6.398	5.17	2993	0.276987676	isogroup01644	ENSG00000141027	ENST00000395857	NCOR1
isotig11892	6.25	5.678	6.495	5.195	2954	0.322020365	isogroup01644	ENSG00000141027	ENST00000395857	NCOR1
isotig11893	5.632	5.494	6.095	4.972	2813	0.237487788	isogroup01644	ENSG00000141027	ENST00000395849	NCOR1
isotig11894	5.751	5.414	6.194	4.995	2774	0.284568272	isogroup01644	ENSG00000141027	ENST00000395849	NCOR1
isotig11895	3.655	2.931	3.068	2.254	2922	0.372003457	isogroup01645	ENSG00000117859	ENST00000447887	OSBPL9
isotig11896	3.652	2.958	3.113	2.308	2883	0.366038551	isogroup01645	ENSG00000117859	ENST00000447887	OSBPL9
isotig11897	3.687	2.932	3.09	2.262	2853	0.379856467	isogroup01645	ENSG00000117859	ENST00000371710	OSBPL9
isotig11898	3.685	2.959	3.136	2.317	2814	0.373985496	isogroup01645	ENSG00000117859	ENST00000337809	OSBPL9
isotig11899	2.927	3.219	4.046	3.61	2973	0.082137221	isogroup01646	ENSG00000031823	ENST00000340578	RANBP3
isotig11900	3.074	3.302	4.236	3.782	2886	0.094226723	isogroup01646	ENSG00000031823	ENST00000034275	RANBP3
isotig11901	2.777	3.027	3.917	3.504	2853	0.10439994	isogroup01646	ENSG00000031823	ENST00000340578	RANBP3
isotig11902	2.926	3.109	4.111	3.679	2766	0.117494552	isogroup01646	ENSG00000031823	ENST00000034275	RANBP3
isotig11903	3.457	2.675	3.708	3.032	2988	0.283553769	isogroup01647	ENSG00000158526	ENST00000375151	TSR2
isotig11904	3.122	2.401	3.414	2.632	2949	0.313021342	isogroup01647	ENSG00000102302	ENST00000375135	FGD1
isotig11905	3.545	2.832	3.949	3.203	2759	0.259562636	isogroup01647	ENSG00000158526	ENST00000375151	TSR2
isotig11906	3.183	2.537	3.633	2.772	2720	0.292064327	isogroup01647	ENSG00000158526	ENST00000375151	TSR2
isotig11907	6.341	5.017	5.072	4.257	2846	0.367776358	isogroup01648	ENSG00000125375	ENST00000311459	ATP5S
isotig11909	4.448	3.567	3.472	2.873	2674	0.329290975	isogroup01648	ENSG00000125375	ENST00000245448	ATP5S
isotig11911	4.827	1.31	5.102	3.149	3668	0.770233712	isogroup01649	ENSG00000187486	ENST00000339994	KCNJ11
isotig11912	4.433	1.423	4.544	2.871	3131	0.73725295	isogroup01649	ENSG00000187486	ENST00000339994	KCNJ11
isotig11913	4.249	2.436	3.278	2.707	2543	0.499690013	isogroup01649	ENSG00000082701	ENST00000316626	GSK3B
isotig12359	6.531	7.051	7.398	6.112	1532	0.062654994	isogroup01762	ENSG00000156642	ENST00000351217	NPTN
isotig12360	2.514	2.97	3.73	2.426	1474	0.123543999	isogroup01762	ENSG00000156642	ENST00000542234	NPTN
isotig12361	7.346	7.983	7.602	6.789	1328	0.185748103	isogroup01762	ENSG00000156642	ENST00000351217	NPTN
isotig12362	2.72	3.29	3.355	2.543	1270	0.099938003	isogroup01762	ENSG00000156642	ENST00000542234	NPTN
isotig12363	7.745	9.909	4.956	6.118	1578	0.518824679	isogroup01763	ENSG00000147166	ENST00000373829	ITGB1BP2
isotig12364	9.451	12.723	6.122	7.901	1309	0.592366799	isogroup01763	ENSG00000147166	ENST00000373829	ITGB1BP2
isotig12365	5.656	6.026	3.632	4.094	1393	0.25285564	isogroup01763	ENSG00000147166	ENST00000373829	ITGB1BP2
isotig12366	7.142	8.374	4.673	5.686	1124	0.418820921	isogroup01763	ENSG00000147166	ENST00000373829	ITGB1BP2
isotig12367	126.061	89.257	130.647	120.82	1305	0.456141504	isogroup01764	ENSG00000204219	ENST00000450454	TCEA3
isotig12369	115.163	79.539	119.129	109.419	1242	0.479033591	isogroup01764	ENSG00000204219	ENST00000450454	TCEA3
isotig12371	37.222	36.694	38.033	38.532	1406	0.039969565	isogroup01765	ENSG00000112335	ENST00000230085	SNX3
isotig12373	31.638	30.951	33.079	31.822	1146	0.022356654	isogroup01765	ENSG00000112335	ENST00000230085	SNX3
isotig12375	25.035	39.404	30.29	32.076	1818	0.443817164	isogroup01766	ENSG00000099875	ENST00000541165	MKNK2
isotig12376	29.468	48.168	36.744	39.185	1522	0.480978057	isogroup01766	ENSG00000099875	ENST00000250896	MKNK2
isotig12377	10.367	12.96	11.239	10.667	1139	0.081423311	isogroup01766	ENSG00000099875	ENST00000309340	MKNK2
isotig12378	13.219	19.497	16.203	15.987	843	0.315247614	isogroup01766	ENSG00000099875	ENST00000309340	MKNK2
isotig12379	3.69	3.638	3.334	2.868	1385	0.115456151	isogroup01767	ENSG00000249915	ENST00000264933	PDCD6
isotig12380	3.123	2.974	2.89	2.52	1323	0.147910874	isogroup01767	ENSG00000249915	ENST00000264933	PDCD6
isotig12381	4.424	4.429	3.818	3.374	1277	0.089520553	isogroup01767	ENSG00000249915	ENST00000264933	PDCD6
isotig12382	3.689	3.551	3.19	2.918	1214	0.125638762	isogroup01767	ENSG00000249915	ENST00000502359	PDCD6
isotig12383	4.044	3.804	4.75	3.687	1410	0.252451717	isogroup01768	ENSG00000158636	ENST00000533248	C11orf30

isotig12384	4.103	3.872	4.833	3.751	1380	0.253691666	isogroup01768	ENSG00000158636	ENST00000533248	C11orf30
isotig12397	1.9	1.459	1.092	1.028	1230	0.048414368	isogroup01771	ENSG00000147526	ENST00000276520	TACC1
isotig12398	1.847	1.526	1.116	1.012	1195	0.029392425	isogroup01771	ENSG00000147526	ENST00000518415	TACC1
isotig12399	2.858	2.432	2.64	2.3	1385	0.194089577	isogroup01772	ENSG00000173992	ENST00000530961	CCS
isotig12400	2.861	2.622	2.705	2.526	1314	0.105376869	isogroup01772	ENSG00000173992	ENST00000530961	CCS
isotig12401	2.801	2.828	2.894	2.668	1198	0.014428496	isogroup01772	ENSG00000173992	ENST00000533244	CCS
isotig12402	2.799	3.074	2.986	2.955	1127	0.102370933	isogroup01772	ENSG00000173992	ENST00000533244	CCS
isotig12403	326.712	246.153	210.014	208.475	1458	0.38057977	isogroup01773	ENSG00000174444	ENST00000432669	RPL4
isotig12404	339.306	255.647	218.132	216.557	1404	0.380645525	isogroup01773	ENSG00000174444	ENST00000432669	RPL4
isotig12406	3.859	5.78	4.69	4.144	1379	0.295530548	isogroup01774	ENSG00000138777	ENST00000341695	PPA2
isotig12407	4.429	6.836	5.394	4.626	1223	0.338844217	isogroup01774	ENSG00000138777	ENST00000357415	PPA2
isotig12408	3.188	4.743	3.966	3.45	1215	0.279570903	isogroup01774	ENSG00000138777	ENST00000357415	PPA2
isotig12409	3.748	5.81	4.673	3.906	1059	0.328126174	isogroup01774	ENSG00000138777	ENST00000357415	PPA2
isotig12410	14.83	12.184	14.694	14.052	1349	0.33840272	isogroup01775	ENSG00000105698	ENST00000379134	USF2
isotig12411	16.933	14.34	16.965	16.688	1262	0.301157286	isogroup01775	ENSG00000105698	ENST00000379134	USF2
isotig12412	18.88	15.601	18.726	18.306	1167	0.331507853	isogroup01775	ENSG00000105698	ENST00000222305	USF2
isotig12413	21.663	18.395	21.705	21.729	1080	0.293586083	isogroup01775	ENSG00000105698	ENST00000222305	USF2
isotig12418	34.739	11.06	19.075	15.092	1967	0.878560156	isogroup01777	ENSG00000175262	ENST00000418570	C1orf127
isotig12894	2.912	2.287	2.249	2.718	832	0.281637484	isogroup01923	ENSG00000257382	ENST00000551838	AC217772.1
isotig12895	5.668	7.316	6.644	5.36	3451	0.061292929	isogroup01924	ENSG00000107959	ENST00000380980	PITRM1
isotig12896	4.648	5.938	5.26	4.459	2638	0.080662433	isogroup01924	ENSG00000107959	ENST00000380980	PITRM1
isotig12897	6.016	6.555	6.834	4.854	1180	0.228667243	isogroup01924	ENSG00000107959	ENST00000451104	PITRM1
isotig12898	3.102	2.423	2.635	1.993	2676	0.357133464	isogroup01925	ENSG00000185515	ENST00000369462	BRCC3
isotig12899	3.069	2.449	2.661	2.024	2601	0.3515443	isogroup01925	ENSG00000185515	ENST00000340647	BRCC3
isotig12900	2.412	2.133	2.379	1.683	1951	0.294065154	isogroup01925	ENSG00000213121	ENST00000392385	AL590867.1
isotig12901	2.347	1.415	2.147	1.745	2516	0.37843804	isogroup01926	ENSG00000197562	ENST00000538492	RAB40C
isotig12902	2.323	1.348	2.139	1.623	2430	0.413776584	isogroup01926	ENSG00000197562	ENST00000538492	RAB40C
isotig12903	2.69	1.779	2.326	1.936	2273	0.379039227	isogroup01926	ENSG00000197562	ENST00000538492	RAB40C
isotig12904	1.984	1.194	1.831	1.366	2917	0.312213497	isogroup01927	ENSG00000106009	ENST00000340611	BRAT1
isotig12905	1.841	1.116	1.686	1.289	2797	0.293623657	isogroup01927	ENSG00000106009	ENST00000340611	BRAT1
isotig12906	2	1.129	1.825	1.282	1501	0.401752837	isogroup01927	ENSG00000106009	ENST00000340611	BRAT1
isotig12907	7.753	5.31	7.934	7.087	3234	0.382101526	isogroup01928	ENSG00000169221	ENST00000409939	TBC1D10B
isotig12908	7.803	5.518	8.505	6.76	2384	0.462519726	isogroup01928	ENSG00000169221	ENST00000409939	TBC1D10B
isotig12909	4.499	2.818	4.46	4.015	1567	0.394200421	isogroup01928	ENSG00000169221	ENST00000409939	TBC1D10B
isotig12910	4.42	3.882	3.976	3.198	2890	0.216634102	isogroup01929	ENSG00000157933	ENST00000378536	SKI
isotig12911	4.151	3.614	3.715	2.945	2830	0.221716014	isogroup01929	ENSG00000157933	ENST00000378536	SKI
isotig12912	3.043	2.419	2.321	1.69	1435	0.236773878	isogroup01929	ENSG00000157933	ENST00000378536	SKI
isotig12913	15.047	20.72	13.498	15.609	2600	0.416744946	isogroup01930	ENSG00000165192	ENST00000537676	ASB11
isotig12914	13.285	18.947	12.063	14.001	2542	0.447612159	isogroup01930	ENSG00000165192	ENST00000480796	ASB11
isotig12916	5.992	6.065	7.188	5.752	3573	0.290288946	isogroup01931	ENSG00000171298	ENST00000390015	GAA
isotig12917	4.22	4.361	4.81	4.204	2021	0.186828361	isogroup01931	ENSG00000171298	ENST00000390015	GAA
isotig12918	6.939	6.725	8.084	5.807	1544	0.398277223	isogroup01931	ENSG00000171298	ENST00000390015	GAA
isotig12919	9.392	1.056	5.6	2.945	2910	0.8981645	isogroup01932	ENSG00000240038	ENST00000361355	AMY2B
isotig12920	11.625	1.213	6.955	3.659	2205	0.916341024	isogroup01932	ENSG00000240038	ENST00000361355	AMY2B
isotig12922	4.054	4.036	3.42	3.142	3203	0.027992786	isogroup01933	ENSG00000035681	ENST00000038176	NSMAF
isotig12923	2.619	2.71	2.593	1.954	2045	0.074481476	isogroup01933	ENSG00000035681	ENST00000427130	NSMAF
isotig12924	2.97	2.855	2.839	2.003	1598	0.20052416	isogroup01933	ENSG00000035681	ENST00000427130	NSMAF
isotig12926	1.928	1.99	1.005	1.555	2066	0.259271436	isogroup01934	ENSG00000038219	ENST00000040738	BOD1L
isotig12928	4.483	6.814	6.215	5.764	2480	0.250150297	isogroup01935	ENSG00000149150	ENST00000528450	SLC43A1
isotig12929	4.207	6.194	5.542	5.22	2258	0.258350868	isogroup01935	ENSG00000149150	ENST00000528450	SLC43A1
isotig12930	4.259	6.087	5.409	5.167	2071	0.270562486	isogroup01935	ENSG00000149150	ENST00000528450	SLC43A1
isotig12934	7.091	9.176	8.004	5.991	2845	0.186274141	isogroup01937	ENSG00000130592	ENST00000381775	LSP1
isotig12935	7.648	9.982	8.56	6.38	2703	0.189590065	isogroup01937	ENSG00000130592	ENST00000311604	LSP1
isotig12936	7.616	11.631	7.913	10.631	1180	0.636112572	isogroup01937	ENSG00000117399	ENST00000437896	CDC20
isotig12940	12.335	11.898	17.308	15.558	3957	0.25758999	isogroup01939	ENSG00000092098	ENST00000324103	RNF31
isotig12941	5.686	5.4	7.687	6.794	1995	0.219470955	isogroup01939	ENSG00000092098	ENST00000324103	RNF31
isotig12942	8.87	8.413	12.576	10.402	707	0.327355903	isogroup01939	ENSG00000092098	ENST00000559275	RNF31
isotig12943	5.957	6.145	6.963	6.86	2945	0.045004509	isogroup01940	ENSG00000006695	ENST00000261643	COX10
isotig12944	5.814	5.986	7.003	6.476	2712	0.034305253	isogroup01940	ENSG00000006695	ENST00000261643	COX10
isotig12945	3.617	2.904	3.49	3.527	933	0.159624634	isogroup01940	ENSG00000006695	ENST00000261643	COX10
isotig12946	5.51	4.686	5.684	4.632	2295	0.291726159	isogroup01941	ENSG00000138385	ENST00000409333	SSB
isotig12947	4.94	4.366	5.317	4.134	2160	0.306239197	isogroup01941	ENSG00000138385	ENST00000409333	SSB
isotig12948	3.503	3.118	4.055	2.715	2097	0.358983993	isogroup01941	ENSG00000138385	ENST00000409333	SSB
isotig12949	39.737	24.006	41.044	34.21	2841	0.623525212	isogroup01942	ENSG00000177731	ENST00000327031	FLII
isotig12950	21.141	13.671	19.61	16.949	2250	0.537329977	isogroup01942	ENSG00000177731	ENST00000327031	FLII
isotig12951	13.442	8.107	12.905	11.04	1458	0.555337416	isogroup01942	ENSG00000177731	ENST00000327031	FLII
isotig12952	3.121	1.994	3.778	5.474	2245	0.247510709	isogroup01943	ENSG00000213928	ENST00000560275	IRF9
isotig12953	3.074	2	3.928	5.818	2143	0.190520027	isogroup01943	ENSG00000213928	ENST00000560275	IRF9
isotig12954	3.138	1.948	4.072	5.613	2058	0.301955738	isogroup01943	ENSG00000213928	ENST00000396864	IRF9
isotig12955	16.598	13.425	15.151	14.601	2627	0.313049523	isogroup01944	ENSG00000125826	ENST00000356286	RBCK1
isotig12956	6.898	6.346	6.837	6.393	2540	0.142622304	isogroup01944	ENSG00000125826	ENST00000356286	RBCK1
isotig12957	8.738	7.526	8.851	7.782	1235	0.292580972	isogroup01944	ENSG00000125826	ENST00000382181	RBCK1
isotig12961	27.576	33.4	36.203	35.456	4272	0.149122642	isogroup01946	ENSG00000101892	ENST00000218008	ATP1B4
isotig12962	7.789	10.926	9.211	9.749	1279	0.361388743	isogroup01946	ENSG00000101892	ENST00000218008	ATP1B4
isotig12963	5.501	7.843	4.719	6.281	720	0.455352446	isogroup01946	ENSG00000101892	ENST00000218008	ATP1B4
isotig12964	21.52	13.104	22.5	17.6	3265	0.653387315	isogroup01947	ENSG00000084463	ENST00000261167	WBP11
isotig12965	1.282	2.41	2.526	2.87	1703	0.297634704	isogroup01947	ENSG00000120055	ENST00000239125	C10orf95
isotig12966	9.488	5.627	9.518	7.419	1291	0.641457504	isogroup01947	ENSG00000084463	ENST00000261167	WBP11
isotig12967	8.122	6.544	6.021	5.772	3186	0.294008792	isogroup01948	ENSG00000196636	ENST00000432641	ACN9
isotig12970	49.693	38.674	32.343	46.935	3066	0.212378823	isogroup01949	ENSG00000123240	ENST00000378748	OPTN
isotig12971	52.647	41.027	34.205	49.843	2992	0.212867288	isogroup01949	ENSG00000123240	ENST00000378748	OPTN
isotig12972	20.656	14.757	20.742	25.113	4606	0.08594161	isogroup01950	ENSG00000141456	ENST00000269230	AC091153.1

isotig12973	19.849	15.838	7.513	17.324	1477	0.340102953	isogroup01950	ENSG00000141456	ENST00000269230	AC091153.1
isotig12974	2.925	2.788	3.276	2.795	2283	0.133238145	isogroup01951	ENSG00000021355	ENST00000380739	SERPINB1
isotig12975	3.772	3.62	3.878	3.263	1945	0.143646201	isogroup01951	ENSG00000021355	ENST00000380739	SERPINB1
isotig12976	3.835	3.482	3.832	3.264	1814	0.177904486	isogroup01951	ENSG00000021355	ENST00000380739	SERPINB1
isotig12977	5.807	27.846	8.099	21.708	3317	0.959062899	isogroup01952	ENSG00000080546	ENST00000436639	SESN1
isotig12978	5.849	30.629	8.457	23.774	2607	0.963590591	isogroup01952	ENSG00000080546	ENST00000356644	SESN1
isotig12979	2.945	2.881	2.676	1.758	2842	0.19021004	isogroup01953	ENSG00000188529	ENST00000344989	SRSF10
isotig12980	6.032	4.726	4.86	3.083	2659	0.526170437	isogroup01953	ENSG00000188529	ENST00000492112	SRSF10
isotig12981	2.598	2.475	1.276	1.378	482	0.071428571	isogroup01953	ENSG00000188529	ENST00000492112	SRSF10
isotig12982	71.845	52.504	77.999	32.019	2103	0.83596979	isogroup01954	ENSG00000167315	ENST00000285093	ACAA2
isotig12983	39.546	27.346	39.971	16.3	2061	0.837228526	isogroup01954	ENSG00000167315	ENST00000285093	ACAA2
isotig12984	45.23	31.39	45.872	18.662	1787	0.836664913	isogroup01954	ENSG00000167315	ENST00000285093	ACAA2
isotig12985	167.4	201.798	308.478	336.022	4006	0.310090554	isogroup01955	ENSG00000196296	ENST00000357084	ATP2A1
isotig12986	546.598	665.946	1009.389	1094.706	1201	0.311527767	isogroup01955	ENSG00000196296	ENST00000357084	ATP2A1
isotig12989	4.128	3.036	3.863	2.689	1995	0.372867664	isogroup01956	ENSG00000214063	ENST00000409531	TSPAN4
isotig12990	4.198	3.171	4.045	2.872	1814	0.361679943	isogroup01956	ENSG00000214063	ENST00000409531	TSPAN4
isotig12991	20.467	12.904	25.974	20.729	2700	0.618358759	isogroup01957	ENSG00000124422	ENST00000455117	USP22
isotig12992	16.184	10.234	18.958	15.413	1817	0.615935222	isogroup01957	ENSG00000124422	ENST00000455117	USP22
isotig12993	13.87	9.143	19.678	15.14	1347	0.58253175	isogroup01957	ENSG00000124422	ENST00000537526	USP22
isotig12994	7.958	10.114	12.975	10.516	2709	0.052049673	isogroup01958	ENSG00000112234	ENST00000369244	FBXL4
isotig12995	1.971	19.712	10.89	13.978	2275	0.925020666	isogroup01958	ENSG00000135744	ENST00000366667	AGT
isotig12996	1.956	2.374	3.346	2.427	858	0.112901105	isogroup01958	ENSG00000112234	ENST00000369244	FBXL4
contig19559	202.806	199.738	217.58	169.483	545	0.279336064	isogroup01959	ENSG00000169021	ENST00000304863	UQCRFS1
isotig12997	133.222	129.002	140.929	113.341	2641	0.278237018	isogroup01959	ENSG00000169021	ENST00000304863	UQCRFS1
isotig12999	4.231	3.735	3.845	4.553	2524	0.111839633	isogroup01960	ENSG00000146733	ENST00000437355	PSPH
isotig13000	5.208	4.263	5.885	4.96	1640	0.373788232	isogroup01960	ENSG00000261657	ENST00000566782	SLC25A26.1
isotig13001	6.636	5.774	7.11	6.112	1534	0.38187608	isogroup01960	ENSG00000261657	ENST00000566782	SLC25A26.1
isotig13002	7.924	5.096	6.725	5.194	2614	0.538090854	isogroup01961	ENSG00000126012	ENST00000375383	KDM5C
isotig13003	7.714	5.202	6.564	5.328	2460	0.479756895	isogroup01961	ENSG00000126012	ENST00000375383	KDM5C
isotig13004	3.482	2.116	2.955	1.774	606	0.594292478	isogroup01961	ENSG00000126012	ENST00000452825	KDM5C
isotig13005	2.929	3.159	2.969	1.659	2533	0.281440219	isogroup01962	ENSG00000177000	ENST00000376590	MTHFR
isotig13006	3.102	3.578	3.148	1.783	2231	0.203492523	isogroup01962	ENSG00000177000	ENST00000376590	MTHFR
isotig13008	9.768	4.898	8.164	4.805	2362	0.792505824	isogroup01963	ENSG00000041880	ENST00000431474	PARP3
isotig13009	11.055	5.55	8.948	5.454	2153	0.791237694	isogroup01963	ENSG00000041880	ENST00000398755	PARP3
isotig13010	8.303	3.968	7.036	3.923	1124	0.791124972	isogroup01963	ENSG00000041880	ENST00000431474	PARP3
isotig13011	22.781	24.422	24.152	21.873	3855	0.023211468	isogroup01964	ENSG00000100347	ENST00000350028	SAMM50
isotig13012	6.34	7.319	6.427	6.221	860	0.195695874	isogroup01964	ENSG00000100347	ENST00000350028	SAMM50
isotig13013	17.517	17.563	17.824	16.167	826	0.007815435	isogroup01964	ENSG00000100347	ENST00000350028	SAMM50
isotig13014	4.121	3.806	1.528	5.069	2702	0.597890208	isogroup01965	ENSG00000176463	ENST00000424469	SLCO3A1
isotig13015	4.134	3.338	1.569	4.798	2220	0.483119787	isogroup01965	ENSG00000176463	ENST00000318445	SLCO3A1
isotig13016	5.25	1.626	1.24	2.273	601	0.589210566	isogroup01965	ENSG00000176463	ENST00000424469	SLCO3A1
isotig13019	1.831	3.08	2.864	2.638	1341	0.37743293	isogroup01966	ENSG00000134255	ENST00000545121	CEPT1
isotig13020	4.482	5.28	3.361	1.633	3122	0.25301533	isogroup01967	ENSG00000186205	ENST00000407981	1-Mar
isotig13023	11.625	8.012	8.183	8.064	3105	0.436790787	isogroup01968	ENSG00000137216	ENST00000323267	TMEM63B
isotig13024	6.827	3.883	4.837	4.257	1483	0.567041783	isogroup01968	ENSG00000137216	ENST00000371893	TMEM63B
isotig13025	4.025	2.224	2.538	1.884	826	0.49857218	isogroup01968	ENSG00000137216	ENST00000532634	TMEM63B
isotig13029	10.528	5.768	7.204	5.451	4310	0.629959796	isogroup01970	ENSG00000186812	ENST00000330501	ZNF397
isotig13030	8.724	3.758	5.078	4.533	689	0.698983618	isogroup01970	ENSG00000186812	ENST00000355632	ZNF397
isotig13031	3.801	3.246	3.925	5.367	2853	0.053402345	isogroup01971	ENSG00000172432	ENST00000307126	GTPBP2
isotig13032	3.223	2.868	3.321	4.216	1540	0.027992786	isogroup01971	ENSG00000172432	ENST00000393882	GTPBP2
isotig13033	2.106	1.845	2.284	2.972	934	0.010060494	isogroup01971	ENSG00000172432	ENST00000307126	GTPBP2
isotig13034	8.432	18.076	10.066	15.755	3099	0.855874728	isogroup01972	ENSG00000155792	ENST00000286234	DEPTOR
isotig13035	4.257	9.796	6.06	9.437	1982	0.826463515	isogroup01972	ENSG00000155792	ENST00000523492	DEPTOR
isotig13036	1.116	1.272	1.047	1.239	2078	0.119006914	isogroup01973	ENSG00000069943	ENST00000164305	PIGB
isotig13037	1.201	1.359	1.168	1.368	1832	0.131406403	isogroup01973	ENSG00000069943	ENST00000164305	PIGB
isotig13039	10.495	8.21	11.256	9.245	2687	0.481804689	isogroup01974	ENSG00000129910	ENST00000289746	CDH15
isotig13040	3.712	3.151	4.154	3.06	1329	0.42447584	isogroup01974	ENSG00000129910	ENST00000289746	CDH15
isotig13041	5.064	4.162	5.37	4.109	1262	0.470494852	isogroup01974	ENSG00000129910	ENST00000289746	CDH15
isotig13044	35.178	23.112	24.219	19.477	985	0.441487563	isogroup01975	ENSG00000114353	ENST00000540560	GNAI2
isotig13048	3.328	3.18	3.252	13.723	2058	0.631256106	isogroup01977	ENSG00000125347	ENST00000405885	IRF1
isotig13049	2.904	2.548	2.827	11.123	1754	0.555741339	isogroup01977	ENSG00000125347	ENST00000405885	IRF1
isotig13050	1.907	1.7	1.849	7.127	1288	0.457052679	isogroup01977	ENSG00000125347	ENST00000405885	IRF1
isotig13051	1.604	1.437	3	3.817	1868	0.025447133	isogroup01978	ENSG00000105642	ENST00000222249	KCNN1
isotig13054	57.392	65.963	45.872	60.364	1899	0.474768919	isogroup01979	ENSG00000198848	ENST00000360526	CES1
isotig13055	28.054	32.291	22.51	28.572	1578	0.442342376	isogroup01979	ENSG00000198848	ENST00000426667	CES1
isotig13056	34.306	39.785	27.705	35.843	1558	0.468869768	isogroup01979	ENSG00000198848	ENST00000422046	CES1
isotig13057	4.864	3.802	4.681	3.082	2408	0.445113474	isogroup01980	ENSG00000164548	ENST00000538367	TRA2A
isotig13058	4.957	3.881	4.771	3.146	2359	0.447367927	isogroup01980	ENSG00000164548	ENST00000538367	TRA2A
isotig13059	3.848	3.383	5.391	4.871	2307	0.233570677	isogroup01981	ENSG00000169016	ENST00000381525	E2F6
isotig13060	3.116	2.952	4.885	4.014	1612	0.223463215	isogroup01981	ENSG00000169016	ENST00000546212	E2F6
isotig13061	3.527	2.703	3.863	4.404	1042	0.139954535	isogroup01981	ENSG00000169016	ENST00000381525	E2F6
isotig13062	30.671	27.681	48.345	40.81	2601	0.286907267	isogroup01982	ENSG00000180891	ENST00000407144	CUEDC1
isotig13063	10.533	9.012	16.33	13.973	1695	0.322405501	isogroup01982	ENSG00000196739	ENST00000494090	COL27A1
isotig13064	17.305	16.193	26.559	23.731	633	0.235421207	isogroup01982	ENSG00000180891	ENST00000407144	CUEDC1
isotig13065	8.968	7.244	7.42	6.576	2257	0.326040806	isogroup01983	ENSG00000182979	ENST00000331320	MTA1
isotig13066	4.347	3.467	3.554	2.616	1647	0.444906816	isogroup01983	ENSG00000182979	ENST00000405646	MTA1
isotig13067	3.206	3.16	3.102	2.54	1009	0.247332231	isogroup01983	ENSG00000182979	ENST00000438610	MTA1
isotig13068	19.999	18.655	30.55	26.607	2663	0.295990832	isogroup01984	ENSG00000161664	ENST00000293414	ASB16
isotig13069	12.514	11.772	19.68	16.068	1945	0.273643571	isogroup01984	ENSG00000161664	ENST00000293414	ASB16
isotig13071	7.277	5.722	8.177	8.731	3255	0.172155632	isogroup01985	ENSG00000185105	ENST00000409745	MYADML2
isotig13072	11.843	7.952	11.522	12.334	1629	0.304351093	isogroup01985	ENSG00000185105	ENST00000409745	MYADML2
isotig13073	12	15.17	16.143	15.344	2330	0.112638085	isogroup01986	ENSG00000170855	ENST00000546954	TRIAP1

isotig13075	8.759	8.966	11.616	9.422	2800	0.122538889	isogroup01987	ENSG00000110066	ENST00000405515	SUV420H1
isotig13076	5.875	5.936	6.28	5.853	2033	0.0314684	isogroup01987	ENSG00000110066	ENST00000304363	SUV420H1
contig19704	47.7	38.087	55.129	36.744	520	0.619655069	isogroup01988	ENSG00000136156	ENST00000378565	ITM2B
isotig13077	15.896	11.603	11.224	9.801	2637	0.502508078	isogroup01988	ENSG00000102471	ENST00000218652	NDFIP2
isotig13078	112.669	89.27	118.556	85.437	1645	0.574171489	isogroup01988	ENSG00000136156	ENST00000378565	ITM2B
isotig13079	6.018	5.221	6.24	5.76	2131	0.240390396	isogroup01989	ENSG00000117419	ENST00000372257	ERI3
isotig13080	4.55	4.175	5.094	4.244	1853	0.280707522	isogroup01989	ENSG00000117419	ENST00000372259	ERI3
isotig13081	5.564	5.196	6.303	5.092	828	0.285282182	isogroup01989	ENSG00000117419	ENST00000456170	ERI3
isotig13082	2.84	2.409	2.594	2.25	1758	0.253503795	isogroup01990	ENSG00000028116	ENST00000394539	VRK2
isotig13083	3.045	2.512	2.68	2.422	1633	0.243067558	isogroup01990	ENSG00000028116	ENST00000435505	VRK2
isotig13084	1.997	2.279	1.613	1.949	1420	0.168539115	isogroup01990	ENSG00000028116	ENST00000423109	VRK2
isotig13085	2.462	2.679	2.816	2.74	1659	0.003503795	isogroup01991	ENSG00000089177	ENST00000377997	KIF16B
isotig13086	8.227	6.879	9.186	11.109	1584	0.178721725	isogroup01991	ENSG00000015133	ENST00000427583	CCDC88C
isotig13088	5.666	7.038	7.62	7.117	2078	0.101976403	isogroup01992	ENSG00000172732	ENST00000437855	MUS81
isotig13089	5.579	6.463	7.248	6.7	1992	0.047550162	isogroup01992	ENSG00000172732	ENST00000533035	MUS81
isotig13090	4.768	5.249	5.861	5.146	712	0.072048546	isogroup01992	ENSG00000172732	ENST00000533035	MUS81
isotig13091	2.634	1.585	2.045	2.49	2158	0.17677726	isogroup01993	ENSG00000004866	ENST00000393449	ST7
isotig13092	2.683	1.635	2.055	2.557	2022	0.153293379	isogroup01993	ENSG00000004866	ENST00000323984	ST7
isotig13094	13.133	8.922	11.513	8.505	1997	0.576332006	isogroup01994	ENSG00000020426	ENST00000261245	MNAT1
isotig13095	96.363	41.73	82.255	63.112	1929	0.849637409	isogroup01994	ENSG00000108823	ENST00000262018	SGCA
isotig13096	4.24	3.495	3.413	2.37	836	0.366583377	isogroup01994	ENSG00000020426	ENST00000557134	MNAT1
isotig13097	26.129	18.97	22.409	18.713	2013	0.459664087	isogroup01995	ENSG00000161203	ENST00000411763	AP2M1
isotig13098	19.084	13.033	15.415	13.266	1471	0.47138724	isogroup01995	ENSG00000161203	ENST00000292807	AP2M1
isotig13099	18.857	13.002	15.303	13.489	1279	0.45010145	isogroup01995	ENSG00000161203	ENST00000292807	AP2M1
isotig13103	1.914	4.156	3.206	2.793	2450	0.439345833	isogroup01997	ENSG00000145439	ENST00000306193	CBR4
isotig13104	1.458	2.215	2.142	1.645	1710	0.029392425	isogroup01997	ENSG00000145439	ENST00000504480	CBR4
isotig13106	43.641	39.146	44.728	54.595	1925	0.023070564	isogroup01998	ENSG00000179364	ENST00000325438	PACS2
isotig13108	8.863	5.647	7.221	7.484	863	0.462059442	isogroup01998	ENSG00000175115	ENST00000320580	PACS1
isotig13109	15.581	8.25	13.446	9.966	1933	0.7253795	isogroup01999	ENSG00000174915	ENST00000308020	PTDSS2
isotig13110	16.936	9.07	14.78	11.193	1861	0.724224093	isogroup01999	ENSG00000174915	ENST00000308020	PTDSS2
isotig13111	7.646	4.196	6.566	4.9	868	0.664161719	isogroup01999	ENSG00000174915	ENST00000308020	PTDSS2
isotig13112	19.615	21.79	19.24	19.432	2383	0.039152326	isogroup02000	ENSG00000119801	ENST00000402708	YPEL5
isotig13113	20.292	22.737	20.199	20.55	2236	0.022356654	isogroup02000	ENSG00000119801	ENST00000402708	YPEL5
isotig13114	2.558	3.715	3.427	1.776	1620	0.05706583	isogroup02001	ENSG00000161649	ENST00000317310	CD300LG
isotig13115	2.59	4.012	3.454	1.754	1538	0.027992786	isogroup02001	ENSG00000161649	ENST00000317310	CD300LG
isotig13116	2.434	4.157	3.391	1.798	1436	0.099938003	isogroup02001	ENSG00000161649	ENST00000293396	CD300LG
isotig13117	4.072	8.493	2.987	5.378	2408	0.881331254	isogroup02002	ENSG00000231608	ENST00000550212	TNXB
isotig13118	2.426	5.768	2.148	3.561	1556	0.843503419	isogroup02002	ENSG00000231608	ENST00000550212	TNXB
isotig13119	3.328	6.16	2.435	4.311	618	0.846359059	isogroup02002	ENSG00000233323	ENST00000549232	TNXB
isotig13120	4.206	4.873	3.714	3.987	2170	0.172907117	isogroup02003	ENSG00000092931	ENST00000336509	MFSD11
isotig13121	2.742	4.618	2.985	2.933	1828	0.411897873	isogroup02003	ENSG00000092931	ENST00000355954	MFSD11
isotig13122	7.752	2.773	3.662	2.945	532	0.79934433	isogroup02003	ENSG00000092931	ENST00000355954	MFSD11
isotig13123	2.155	1.705	2.098	1.736	1801	0.227370933	isogroup02004	ENSG00000156873	ENST00000328273	PHKG2
isotig13124	2.008	1.727	1.933	1.83	1525	0.166247088	isogroup02004	ENSG00000156873	ENST00000328273	PHKG2
isotig13125	1.866	1.494	2.238	1.54	1202	0.378719847	isogroup02004	ENSG00000156873	ENST00000328273	PHKG2
isotig13126	10.913	9.823	13.165	12.295	2174	0.239695273	isogroup02005	ENSG00000213762	ENST00000418193	ZNF134
isotig13128	73.582	62.81	76.878	72.019	1780	0.261431953	isogroup02006	ENSG00000100804	ENST00000361611	PSMB5
isotig13129	5.588	7.35	5.247	5.224	1645	0.275033817	isogroup02006	ENSG00000163444	ENST00000367242	TMEM183A
isotig13130	3.514	4.337	2.943	2.784	1076	0.082137221	isogroup02006	ENSG00000163444	ENST00000367242	TMEM183A
isotig13134	5.113	9.149	6.266	3.947	2323	0.349026828	isogroup02008	ENSG00000213121	ENST00000392385	AL590867.1
isotig13135	3.637	4.136	5.146	4.924	1554	0.092404374	isogroup02008	ENSG00000134864	ENST00000455100	A2LD1
isotig13137	106.179	123.759	64.041	42.162	3120	0.261760727	isogroup02009	ENSG00000198523	ENST00000357525	PLN
isotig13138	91.853	119.822	57.344	42.178	871	0.023718719	isogroup02009	ENSG00000198523	ENST00000357525	PLN
isotig13139	2.98	2.781	3.954	3.183	2118	0.176260615	isogroup02010	ENSG00000197620	ENST00000450602	CXorf40A
isotig13140	2.982	2.837	3.869	3.162	1835	0.180872849	isogroup02010	ENSG00000197620	ENST00000450602	CXorf40A
isotig13142	2.768	2.079	1.558	1.286	1691	0.17921019	isogroup02011	ENSG00000179044	ENST00000314586	EXOC3L1
isotig13143	2.413	1.997	1.464	1.104	1484	0.149310513	isogroup02011	ENSG00000179044	ENST00000314586	EXOC3L1
isotig13145	5.309	5.253	5.632	3.843	1842	0.353798753	isogroup02012	ENSG00000155367	ENST00000309276	PPM1J
isotig13146	5.442	5.788	5.49	4.261	1698	0.19734914	isogroup02012	ENSG00000155367	ENST00000309276	PPM1J
isotig13148	25.166	22.255	32.168	27.416	1974	0.339097843	isogroup02013	ENSG00000141232	ENST00000499247	TOB1
isotig13149	13.976	11.461	18.145	13.242	1126	0.469217329	isogroup02013	ENSG00000141232	ENST00000499247	TOB1
isotig13150	5.929	5.389	5.901	5.829	1275	0.199922973	isogroup02013	ENSG00000164867	ENST00000461406	NOS3
isotig13151	22.391	11.193	17.783	13.379	2141	0.77916698	isogroup02014	ENSG00000177427	ENST00000395706	SMCR7
isotig13152	8.234	3.046	5.211	3.004	1334	0.837998798	isogroup02014	ENSG00000060718	ENST00000512756	COL11A1
isotig13153	12.213	5.469	10.216	5.954	910	0.832212369	isogroup02014	ENSG00000157927	ENST00000445392	RADIL
isotig13154	4.874	4.612	5.818	5.386	2020	0.219799729	isogroup02015	ENSG00000143294	ENST00000271526	PRCC
isotig13155	5.427	5.243	6.579	5.825	1472	0.261009243	isogroup02015	ENSG00000143294	ENST00000271526	PRCC
isotig13156	2.3	1.65	2.201	2.246	877	0.247031637	isogroup02015	ENSG00000084463	ENST00000261167	WBP11
isotig13157	8.293	6.352	8.381	9.507	1699	0.253456827	isogroup02016	ENSG00000136840	ENST00000335791	ST6GALNAC4
isotig13158	8.668	6.717	8.693	9.888	1638	0.241160667	isogroup02016	ENSG00000136840	ENST00000335791	ST6GALNAC4
isotig13159	6.98	5.163	6.905	7.159	1010	0.305816488	isogroup02016	ENSG00000136840	ENST00000541933	ST6GALNAC4
isotig13160	3.75	3.644	4.951	4.222	1534	0.231062599	isogroup02017	ENSG00000131196	ENST00000542384	NFATC1
isotig13161	6.198	5.93	7.807	7.494	1507	0.208151725	isogroup02017	ENSG00000131196	ENST00000427363	NFATC1
isotig13162	6.476	6.229	8.012	7.76	1291	0.196221913	isogroup02017	ENSG00000131196	ENST00000542384	NFATC1
isotig13163	8.905	7.861	7.194	6.875	2097	0.161127602	isogroup02018	ENSG00000102738	ENST00000323563	MRPS31
isotig13169	1.905	2.413	2.846	1.074	1625	0.426176073	isogroup02020	ENSG00000134324	ENST00000425416	LPIN1
isotig13170	3.187	3.396	4.818	2.051	824	0.511103179	isogroup02020	ENSG00000072364	ENST00000378595	AFF4
isotig13171	5.229	5.425	22.324	19.186	2455	0.020947622	isogroup02021	ENSG00000224982	ENST00000426426	TMEM233
isotig13172	4.493	3.402	8.508	7.765	1257	0.180468926	isogroup02021	ENSG00000224982	ENST00000426426	TMEM233
isotig13173	2.013	2.354	8.108	8.39	539	0.186443225	isogroup02021	ENSG00000224982	ENST00000426426	TMEM233
isotig13174	2.631	2.154	2.433	2.401	2793	0.193911099	isogroup02022	ENSG00000076650	ENST00000170564	GPATCH1
isotig13176	5.283	5.177	5.346	3.937	2138	0.206301195	isogroup02023	ENSG00000176444	ENST00000361168	CLK2

isotig13177	1.657	1.927	2.561	1.538	1121	0.170624483	isogroup02023	ENSG00000176444	ENST00000536801	CLK2
isotig13178	2.828	2.114	2.457	2.114	932	0.243631172	isogroup02023	ENSG00000176444	ENST00000536801	CLK2
contig19884	1.763	1.491	1.838	1.642	536	0.127789885	isogroup02024	ENSG00000105289	ENST00000541714	TJP3
isotig13179	117.742	88.107	141.918	143.814	3384	0.351149771	isogroup02024	ENSG00000170027	ENST00000307630	YWHAG
isotig13180	22.165	14.326	22.646	20.91	1716	0.530529045	isogroup02025	ENSG00000116604	ENST00000348159	MEF2D
isotig13181	10.451	6.403	9.816	9.278	1329	0.495491095	isogroup02025	ENSG00000116604	ENST00000541336	MEF2D
isotig13182	12.511	7.612	11.341	11.62	1143	0.456573608	isogroup02025	ENSG00000116604	ENST00000454816	MEF2D
isotig13183	7.333	3.515	7.077	6.592	1689	0.644266176	isogroup02026	ENSG00000155287	ENST00000370495	SLC25A28
isotig13184	8.946	4.28	8.749	8.425	1380	0.647657248	isogroup02026	ENSG00000155287	ENST00000370495	SLC25A28
isotig13185	8.381	3.758	8.19	6.583	1104	0.730884121	isogroup02026	ENSG00000155287	ENST00000434701	SLC25A28
isotig13186	8.16	7.178	8.23	6.702	2238	0.290561359	isogroup02027	ENSG00000130177	ENST00000356221	CDC16
isotig13187	3.448	2.554	3.36	2.209	1338	0.433643947	isogroup02027	ENSG00000130177	ENST00000375312	CDC16
isotig13188	4.15	3.744	4.796	3.863	539	0.252451717	isogroup02027	ENSG00000169689	ENST00000392359	STRA13
isotig13189	5.759	8.184	9.381	10.192	1561	0.305703765	isogroup02028	ENSG00000042286	ENST00000373248	AIFM2
isotig13190	5.75	8.119	9.353	10.186	1529	0.306342526	isogroup02028	ENSG00000042286	ENST00000373248	AIFM2
isotig13191	4.41	5.693	6.649	7.506	1022	0.252742917	isogroup02028	ENSG00000042286	ENST00000373248	AIFM2
isotig13192	1.916	2.322	2.403	2.207	1706	0.080728188	isogroup02029	ENSG00000103148	ENST00000399953	NPRL3
isotig13193	1.973	2.229	2.47	2.205	1667	0.025447133	isogroup02029	ENSG00000103148	ENST00000399953	NPRL3
isotig13194	1.866	1.779	2.193	1.876	724	0.104597204	isogroup02029	ENSG00000103148	ENST00000399953	NPRL3
isotig13195	4.173	3.609	4.292	4.318	1723	0.170314496	isogroup02030	ENSG00000127663	ENST00000536461	KDM4B
isotig13196	4.423	3.73	4.539	4.603	1621	0.195883745	isogroup02030	ENSG00000127663	ENST00000536461	KDM4B
isotig13197	7.624	1.725	4.243	4.099	750	0.778772451	isogroup02030	ENSG00000255251	ENST00000533250	RP11-1118M6.1.1
isotig13201	1.471	1.56	1.399	1.187	1986	0.086824604	isogroup02032	ENSG00000155729	ENST00000409157	KCTD18
isotig13202	3.697	2.844	3.157	2.912	1471	0.311386864	isogroup02032	ENSG00000155729	ENST00000409157	KCTD18
isotig13203	1.117	1.436	1.322	1.362	614	0.247632825	isogroup02032	ENSG00000155729	ENST00000409157	KCTD18
isotig13204	2.726	1.825	2.309	1.975	1419	0.295436612	isogroup02033	ENSG00000131849	ENST00000254166	ZNF132
isotig13205	6.336	4.784	6.263	3.904	1337	0.522422409	isogroup02033	ENSG00000187607	ENST00000421016	ZNF286A
isotig13206	6.139	4.466	5.968	3.764	1253	0.531947471	isogroup02033	ENSG00000184635	ENST00000343769	ZNF93
isotig13207	5.969	4.445	5.519	4.972	1835	0.405848426	isogroup02034	ENSG00000088298	ENST00000374492	EDEM2
isotig13208	3.426	2.541	2.882	2.583	1326	0.360336665	isogroup02034	ENSG00000088298	ENST00000374492	EDEM2
isotig13209	3.121	2.591	3.258	3.158	819	0.242870294	isogroup02034	ENSG00000088298	ENST00000541621	EDEM2
isotig13214	7.678	6.283	7.433	5.532	1380	0.398784474	isogroup02036	ENSG00000184047	ENST00000443649	DIABLO
isotig13215	3.248	2.486	2.745	2.541	1110	0.213036372	isogroup02036	ENSG00000184047	ENST00000443649	DIABLO
contig19949	104.419	83.671	100.281	84.083	840	0.350172841	isogroup02037	ENSG00000164237	ENST00000296658	CMBL
isotig13216	188.459	147.725	170.815	148.029	1429	0.37931164	isogroup02037	ENSG00000164237	ENST00000296658	CMBL
isotig13218	5.426	8.042	6.29	6.507	1527	0.447189449	isogroup02038	ENSG00000172660	ENST00000311979	TAF15
isotig13219	7.866	8.738	6.714	6.59	1454	0.145524912	isogroup02038	ENSG00000172660	ENST00000311979	TAF15
isotig13220	2.939	4.925	3.042	3.705	872	0.534352221	isogroup02038	ENSG00000172660	ENST00000311979	TAF15
isotig13221	6.778	4.893	6.745	4.344	1778	0.614244383	isogroup02039	ENSG00000168000	ENST00000405837	BSCL2
isotig13222	3.366	2.496	3.239	2.336	1083	0.415354701	isogroup02039	ENSG00000168000	ENST00000524862	BSCL2
isotig13223	6.811	4.408	5.972	3.863	991	0.675997595	isogroup02039	ENSG00000168000	ENST00000405837	BSCL2
isotig13224	180.643	420.821	308.981	626.162	1358	0.948363643	isogroup02040	ENSG00000086967	ENST00000357701	MYBPC2
isotig13226	96.897	217.058	172.323	326.382	1104	0.931802811	isogroup02040	ENSG00000086967	ENST00000357701	MYBPC2
isotig13229	3.179	2.128	2.484	2.07	1249	0.299081311	isogroup02041	ENSG00000116337	ENST00000528667	AMPD2
isotig13230	9.336	7.76	12.251	13.669	1614	0.095062749	isogroup02042	ENSG00000231370	ENST00000548373	PRRC2A
isotig13231	17.221	19.873	17.488	12.321	1260	0.006660029	isogroup02042	ENSG00000225748	ENST00000454306	PRRC2A
isotig13232	8.643	9.418	8.943	7.063	870	0.065529421	isogroup02042	ENSG00000225748	ENST00000454306	PRRC2A
isotig13233	6.243	4.675	6.243	5.25	2292	0.332296911	isogroup02043	ENSG00000177963	ENST00000526104	RIC8A
isotig13234	4.612	3.173	4.046	3.248	972	0.3827215	isogroup02043	ENSG00000177963	ENST00000524854	RIC8A
isotig13235	1.313	1.964	2.469	2.971	441	0.275400165	isogroup02043	ENSG00000177963	ENST00000531209	RIC8A
isotig13239	4.105	3.522	4.097	3.212	1805	0.256209138	isogroup02045	ENSG00000070610	ENST00000378094	GBA2
isotig13240	3.004	2.822	3.088	2.33	1393	0.208518073	isogroup02045	ENSG00000070610	ENST00000545786	GBA2
isotig13241	2.767	2.042	3.019	2.098	480	0.359650936	isogroup02045	ENSG00000070610	ENST00000545786	GBA2
isotig13242	8.844	5.958	8.228	5.571	1379	0.643956188	isogroup02046	ENSG00000104522	ENST00000529064	TSTA3
isotig13243	6.401	4.41	6.459	3.965	1328	0.619861727	isogroup02046	ENSG00000104522	ENST00000529064	TSTA3
isotig13244	5.094	3.576	4.939	3.025	967	0.576134741	isogroup02046	ENSG00000104522	ENST00000529064	TSTA3
isotig13245	11.512	12.867	10.239	9.747	1733	0.095062749	isogroup02047	ENSG00000119723	ENST00000334571	COQ6
isotig13246	12.752	14.23	11.317	10.792	1563	0.096302698	isogroup02047	ENSG00000119723	ENST00000334571	COQ6
isotig13247	3.388	3.445	3.473	2.69	2222	0.210002254	isogroup02048	ENSG00000076356	ENST00000367033	PLXNA2
isotig13248	1.857	1.907	1.699	1.836	1000	0.059301495	isogroup02048	ENSG00000076356	ENST00000367033	PLXNA2
isotig13249	2.078	2.324	1.965	1.957	423	0.039415345	isogroup02048	ENSG00000076356	ENST00000367033	PLXNA2
isotig13250	7.926	5.815	5.889	5.429	1571	0.33980236	isogroup02049	ENSG00000165113	ENST00000376371	GKAP1
isotig13251	8.06	5.76	5.861	5.357	1421	0.362215375	isogroup02049	ENSG00000165113	ENST00000376365	GKAP1
isotig13253	1.423	1.13	1.973	1.427	1137	0.206639363	isogroup02050	ENSG00000164306	ENST00000515774	CCDC111
isotig13255	14.234	8.012	14.99	11.418	1897	0.669065154	isogroup02051	ENSG00000172456	ENST00000303721	FGGY
isotig13256	2.624	5.493	3.024	3.687	1606	0.613943789	isogroup02051	ENSG00000172456	ENST00000430447	FGGY
isotig13260	4.446	2.906	5.194	5.304	1015	0.215572631	isogroup02053	ENSG00000006757	ENST00000444736	PNPLA4
isotig13261	4.741	3.079	5.538	5.538	952	0.231297437	isogroup02053	ENSG00000006757	ENST00000444736	PNPLA4
isotig13263	2.093	8.415	2.171	3.359	1692	0.808578192	isogroup02054	ENSG00000152782	ENST00000371775	PANK1
isotig13264	2.139	4.715	2.448	2.306	1086	0.369373262	isogroup02054	ENSG00000152782	ENST00000371775	PANK1
isotig13266	1.846	1.949	2.852	2.195	1924	0.146821222	isogroup02055	ENSG00000133816	ENST00000537344	MICAL2
isotig13269	18.113	7.095	8.039	7.876	1741	0.743781468	isogroup02056	ENSG00000106537	ENST00000262067	TSPAN13
isotig13270	12.542	5.116	5.866	5.274	1492	0.717883445	isogroup02056	ENSG00000106537	ENST00000262067	TSPAN13
isotig13274	138.724	127.281	147.999	151.695	987	0.137484031	isogroup02058	ENSG00000170296	ENST00000302386	GABARAP
isotig13277	2.561	1.857	3.213	2.54	1322	0.278678515	isogroup02059	ENSG00000253953	ENST00000519479	PCDHGB4
isotig13278	2.697	2.169	3.301	2.674	1281	0.169271812	isogroup02059	ENSG00000240184	ENST00000308177	PCDHGC3
isotig13280	25.044	17.616	22.285	17.286	1624	0.532351394	isogroup02060	ENSG00000074319	ENST00000251968	TSG101
isotig13281	27.239	19.203	25.273	19.447	1584	0.544450289	isogroup02060	ENSG00000074319	ENST00000251968	TSG101
isotig13282	7.535	11.395	9.408	8.431	2950	0.323795747	isogroup02061	ENSG00000133318	ENST00000537981	RTN3
isotig13284	3.494	7.224	3.91	5.528	1471	0.822161269	isogroup02062	ENSG00000168477	ENST00000375244	TNXB
isotig13285	4.355	9.387	4.81	7.329	1134	0.872322838	isogroup02062	ENSG00000168477	ENST00000375244	TNXB
isotig13286	19.602	43.599	27.095	37.121	738	0.899855339	isogroup02062	ENSG00000229353	ENST00000424713	TNXB

isotig13287	12.019	13.15	15.479	11.534	1611	0.22768092	isogroup02063	ENSG00000105968	ENST00000308153	H2AFV
isotig13288	7.374	7.298	9.482	6.533	1518	0.35099008	isogroup02063	ENSG00000105968	ENST00000349299	H2AFV
isotig13289	25.974	10.429	26.843	21.269	1502	0.788353874	isogroup02064	ENSG00000135063	ENST00000455972	FAM189A2
isotig13290	8.864	5.846	9.796	8.217	1381	0.490127377	isogroup02064	ENSG00000135063	ENST00000455972	FAM189A2
isotig13291	3.888	1.737	4.066	3.949	411	0.518965582	isogroup02064	ENSG00000135063	ENST00000455972	FAM189A2
contig20089	648.742	736.972	771.506	599.289	543	0.075439618	isogroup02065	ENSG00000110955	ENST00000552919	ATP5B
isotig13292	828.363	933.808	955.887	787.616	1621	0.041791914	isogroup02065	ENSG00000110955	ENST00000262030	ATP5B
isotig13293	356.711	402.047	406.737	335.922	1116	0.058249418	isogroup02065	ENSG00000110955	ENST00000262030	ATP5B
isotig13294	6.713	4.528	5.799	4.149	1481	0.492024874	isogroup02066	ENSG00000100029	ENST00000354694	PES1
isotig13295	5.552	3.733	4.451	3.541	1154	0.465929586	isogroup02066	ENSG00000100029	ENST00000354694	PES1
isotig13296	4.092	2.98	3.275	2.654	641	0.442887202	isogroup02066	ENSG00000100029	ENST00000402281	PES1
isotig13300	4.149	3.469	3.704	3.63	1964	0.155266025	isogroup02068	ENSG00000101138	ENST00000217109	CSTF1
isotig13302	9.082	6.334	7.954	6.086	1570	0.543060044	isogroup02069	ENSG00000070785	ENST00000360403	EIF2B3
isotig13303	3.373	2.426	3.257	2.585	977	0.398286616	isogroup02069	ENSG00000070785	ENST00000360403	EIF2B3
isotig13304	1.991	1.572	2.109	1.477	642	0.299814008	isogroup02069	ENSG00000070785	ENST00000372182	EIF2B3
isotig13305	8.004	7.452	7.729	6.637	1515	0.225464041	isogroup02070	ENSG00000167272	ENST00000357500	POP5
isotig13306	8.754	8.33	8.455	7.167	1481	0.216399264	isogroup02070	ENSG00000167272	ENST00000357500	POP5
isotig13307	7.898	5.321	6.268	6.073	2138	0.451604419	isogroup02071	ENSG00000142655	ENST00000356607	PEX14
isotig13310	17.258	14.29	9.103	6.103	1645	0.538701435	isogroup02072	ENSG00000123739	ENST00000243501	PLA2G12A
isotig13311	22.928	19.428	11.927	8.187	1158	0.522366048	isogroup02072	ENSG00000123739	ENST00000243501	PLA2G12A
isotig13312	25.575	25.186	27.981	28.002	1527	0.039358984	isogroup02073	ENSG00000121022	ENST00000357849	COPS5
isotig13313	18.211	18.47	19.794	19.204	1333	0.007815435	isogroup02073	ENSG00000121022	ENST00000357849	COPS5
isotig13318	1.447	1.292	4.284	14.808	1058	0.673358007	isogroup02075	ENSG00000240508	ENST00000453059	PSMB9
isotig13323	10.982	7.259	12.312	7.547	1412	0.643392575	isogroup02077	ENSG00000183520	ENST00000373014	UTP11L
isotig13324	3.611	3.747	4.736	3.2	1308	0.315163072	isogroup02077	ENSG00000183520	ENST00000373014	UTP11L
isotig13325	40.197	10.129	13.893	5.253	1098	0.961899752	isogroup02078	ENSG00000078814	ENST00000262873	MYH7B
isotig13326	32.82	7.842	11.883	4.18	973	0.957437815	isogroup02078	ENSG00000078814	ENST00000262873	MYH7B
isotig13327	19.19	4.634	7.235	2.477	946	0.937842865	isogroup02078	ENSG00000078814	ENST00000262873	MYH7B
isotig13328	1.719	1.472	2.051	1.498	1569	0.244523559	isogroup02079	ENSG00000161277	ENST00000392182	THAP8
isotig13329	1.86	1.655	1.703	1.541	843	0.169140302	isogroup02079	ENSG00000125450	ENST00000541827	NUP85
isotig13331	29.063	29.34	38.998	39.061	1062	0.119006914	isogroup02080	ENSG00000103495	ENST00000568411	MAZ
isotig13333	25.679	26.05	35.845	31.93	502	0.039866236	isogroup02080	ENSG00000103495	ENST00000568411	MAZ
isotig13334	59.99	54.763	44.163	39.654	1432	0.198185166	isogroup02081	ENSG00000139180	ENST00000266544	NDUFA9
isotig13335	65.61	61.526	48.114	44.716	1362	0.151424063	isogroup02081	ENSG00000139180	ENST00000266544	NDUFA9
isotig13336	4.651	3.406	6.296	5.129	1855	0.332062073	isogroup02082	ENSG00000214756	ENST00000532971	METTL12
isotig13337	6.804	5.054	9.917	8.098	998	0.384778688	isogroup02082	ENSG00000162194	ENST00000524958	C11orf48
isotig13338	12.404	8.29	11.134	7.711	1068	0.603009694	isogroup02083	ENSG00000173418	ENST00000334982	NAA20
isotig13339	10.121	6.692	9.182	6.397	932	0.596312091	isogroup02083	ENSG00000173418	ENST00000398602	NAA20
isotig13340	9.299	6.3	8.302	5.678	922	0.583424138	isogroup02083	ENSG00000173418	ENST00000310450	NAA20
isotig13341	19.051	14.151	16.463	11.353	1259	0.522366048	isogroup02084	ENSG00000160789	ENST00000473598	LMNA
isotig13342	4.81	4.915	4.837	3.611	1063	0.103761178	isogroup02084	ENSG00000160789	ENST00000473598	LMNA
isotig13343	31.648	19.588	26.042	16.38	602	0.712735778	isogroup02084	ENSG00000160789	ENST00000473598	LMNA
isotig13344	2.455	1.679	2.722	2.584	1291	0.322865785	isogroup02085	ENSG00000156860	ENST00000356166	FBRS
isotig13345	2.483	1.863	2.889	2.9	1204	0.272685429	isogroup02085	ENSG00000156860	ENST00000356166	FBRS
isotig13350	7.086	4.193	5.859	4.196	1228	0.620387766	isogroup02087	ENSG00000116350	ENST00000434636	SRSF4
isotig13351	7.125	5.3	7.113	5.776	896	0.414415345	isogroup02087	ENSG00000116350	ENST00000434636	SRSF4
isotig13352	6.115	4.185	5.554	3.848	737	0.533920117	isogroup02087	ENSG00000116350	ENST00000434636	SRSF4
isotig13353	2.819	3.419	4.117	2.781	1119	0.027992786	isogroup02088	ENSG00000188095	ENST00000341735	MESP2
isotig13354	4.548	3.237	5.181	3.136	1135	0.505607951	isogroup02088	ENSG00000170291	ENST00000396628	C17orf81
isotig13359	3.351	1.401	1.967	1.619	1286	0.521389119	isogroup02090	ENSG00000015532	ENST00000507602	XYLT2
isotig13360	2.256	1.037	1.456	1.279	875	0.372754941	isogroup02090	ENSG00000103489	ENST00000261381	XYLT1
isotig13362	9.072	11.59	7.121	7.974	918	0.40533178	isogroup02091	ENSG00000176956	ENST00000430474	LY6H
isotig13365	32.387	23.999	27.726	24.8	2588	0.391626588	isogroup02092	ENSG00000044574	ENST00000324460	HSPA5
isotig13366	1.386	1.775	1.256	1.426	1162	0.172390471	isogroup02093	ENSG00000221944	ENST00000408957	TIGD1
isotig13367	1.261	1.725	1.516	1.448	800	0.195198016	isogroup02093	ENSG00000213121	ENST00000392385	AL590867.1
isotig13368	1.502	2.16	1.603	1.711	745	0.252376569	isogroup02093	ENSG00000213121	ENST00000392385	AL590867.1
isotig13370	33.258	10.009	13.76	12.277	974	0.901320733	isogroup02094	ENSG00000187479	ENST00000528572	C11orf96
isotig13372	25.543	15.174	23.163	15.098	1181	0.757251822	isogroup02095	ENSG00000115944	ENST00000378669	COX7A2L
isotig13373	27.116	16.419	24.503	15.694	1139	0.759045991	isogroup02095	ENSG00000115944	ENST00000378669	COX7A2L
isotig13374	45.421	29.991	42.873	31.87	2493	0.662517848	isogroup02096	ENSG00000172053	ENST00000306125	QARS
isotig13375	2.146	1.89	1.945	3.136	940	0.193197189	isogroup02097	ENSG00000088899	ENST00000360342	RP5-1187M17.10.1
isotig13376	2.245	1.634	1.049	1.325	924	0.098350492	isogroup02097	ENSG00000088899	ENST00000329152	RP5-1187M17.10.1
isotig13377	2.315	1.659	1.187	1.332	801	0.195198016	isogroup02097	ENSG00000261539	ENST00000565084	FBXL6.1
isotig13378	7.192	3.152	7.72	3.784	926	0.826754716	isogroup02098	ENSG00000182871	ENST00000400337	COL18A1
isotig13379	23.625	18.724	31.397	28.487	899	0.377977756	isogroup02098	ENSG00000107404	ENST00000378888	DVL1
isotig13380	27.873	22.553	36.822	34.232	824	0.340694747	isogroup02098	ENSG00000107404	ENST00000263743	DVL1
isotig13381	5.753	2.407	5.158	2.388	1439	0.800960021	isogroup02099	ENSG00000059122	ENST00000416288	FLYWCH1
isotig13384	13.235	18.568	16.803	15.087	1006	0.21668107	isogroup02100	ENSG00000125656	ENST00000245816	CLPP
isotig13385	10.969	14.712	12.918	11.63	897	0.183399714	isogroup02100	ENSG00000125656	ENST00000245816	CLPP
isotig13386	7.714	10.202	9.027	8.293	737	0.175819118	isogroup02100	ENSG00000125656	ENST00000245816	CLPP
isotig13387	40.89	14.401	14.567	19.019	1253	0.771774254	isogroup02101	ENSG00000234728	ENST00000456268	C6orf48
isotig13388	17.051	6.229	6.999	8.059	1207	0.728798753	isogroup02101	ENSG00000234728	ENST00000456268	C6orf48
isotig13392	8.307	4.778	8.408	5.843	952	0.70023296	isogroup02103	ENSG00000188517	ENST00000494183	COL25A1
isotig13393	9.864	6.17	8.947	6.281	907	0.658572556	isogroup02103	ENSG00000136908	ENST00000314392	DPM2
isotig13394	6.578	4.614	6.155	5.173	753	0.468907342	isogroup02103	ENSG00000136908	ENST00000373110	DPM2
isotig13395	7.466	27.167	16.183	18.066	905	0.842178928	isogroup02104	ENSG00000164776	ENST00000297373	PHKG1
isotig13396	9.195	34.234	19.774	22.629	863	0.858955813	isogroup02104	ENSG00000164776	ENST00000297373	PHKG1
isotig13397	3.206	10.798	6.11	7.701	812	0.779495754	isogroup02104	ENSG00000164776	ENST00000452681	PHKG1
isotig13403	19.234	11.702	18.357	21.396	1002	0.395027053	isogroup02107	ENSG00000198816	ENST00000394341	ZNF358
isotig13404	20.827	12.505	20.225	23.37	790	0.397403622	isogroup02107	ENSG00000198816	ENST00000394341	ZNF358
isotig13405	27.004	16.464	26.339	30.357	743	0.377094762	isogroup02107	ENSG00000198816	ENST00000394341	ZNF358
isotig13406	12.356	21.188	16.811	14.435	1167	0.392866536	isogroup02108	ENSG00000184831	ENST00000379226	APOO

isotig13407	10.573	17.559	14.094	12.019	1071	0.363117156	isogroup02108	ENSG00000184831	ENST00000379226	APOO
isotig13408	3.962	4.005	4.859	3.359	1135	0.197283385	isogroup02109	ENSG00000130175	ENST00000412601	PRKCSH
isotig13409	5.19	5.33	6.44	4.198	951	0.294215451	isogroup02109	ENSG00000130175	ENST00000412601	PRKCSH
isotig13410	2.005	2.683	2.193	1.715	435	0.045840535	isogroup02109	ENSG00000130175	ENST00000412601	PRKCSH
isotig13414	11.348	6.983	10.864	7.124	894	0.70766326	isogroup02111	ENSG00000143256	ENST00000368010	PFDN2
isotig13415	2.971	2.969	2.829	2.699	904	0.152945818	isogroup02111	ENSG00000106078	ENST00000431948	COBL
isotig13416	18.554	11.979	17.038	11.528	634	0.707541144	isogroup02111	ENSG00000143256	ENST00000368010	PFDN2
isotig13417	7.28	9.946	9.51	5.924	1196	0.647243932	isogroup02112	ENSG00000189058	ENST00000421243	APOD
isotig13418	8.009	9.727	10.363	5.028	955	0.561189599	isogroup02112	ENSG00000189058	ENST00000421243	APOD
isotig13419	10.54	13.877	13.545	10.41	1458	0.171159916	isogroup02113	ENSG00000085871	ENST00000503816	MGST2
isotig13420	4.814	7.028	6.02	4.697	753	0.149911701	isogroup02113	ENSG00000085871	ENST00000503816	MGST2
isotig13424	322.705	221.524	194.461	177.652	794	0.536954235	isogroup02115	ENSG00000161016	ENST00000394920	RPL8
isotig13425	360.686	247.62	217.246	198.517	710	0.537254828	isogroup02115	ENSG00000161016	ENST00000394920	RPL8
isotig13427	17.253	11.716	12.942	12.472	779	0.496928308	isogroup02116	ENSG00000083457	ENST00000263087	ITGAE
isotig13428	12.512	7.866	7.919	8.901	830	0.477276997	isogroup02116	ENSG00000083457	ENST00000263087	ITGAE
isotig13429	14.497	6.578	5.466	7.462	777	0.598961073	isogroup02116	ENSG00000196531	ENST00000454682	NACA
isotig13430	9.9	8.631	10.5	10.351	1289	0.081639363	isogroup02117	ENSG00000070501	ENST00000265421	POLB
isotig13431	8.659	6.91	8.79	8.508	1084	0.189590065	isogroup02117	ENSG00000070501	ENST00000518925	POLB
isotig13435	1551.878	2474.177	2896.607	1939.016	962	0.091897122	isogroup02119	ENSG00000170290	ENST00000531293	SLN
isotig13439	58.109	37.738	43.821	40.226	576	0.483467348	isogroup02120	ENSG00000175768	ENST00000321301	TOMM5
isotig13440	5.35	6.811	6.041	4.892	871	0.189533704	isogroup02121	ENSG00000130402	ENST00000440400	ACTN4
isotig13441	7.332	8.712	8.749	6.184	765	0.020365221	isogroup02121	ENSG00000130402	ENST00000424234	ACTN4
isotig13442	12.001	11.717	14.205	9.439	672	0.254715563	isogroup02121	ENSG00000130402	ENST00000424234	ACTN4
isotig13443	55.661	35.164	54.236	35.293	2104	0.731475915	isogroup02122	ENSG00000104388	ENST00000262646	RAB2A
isotig13447	2.118	1.659	1.807	1.313	1427	0.296159916	isogroup02124	ENSG00000225614	ENST00000565624	ZNF469
isotig13448	1.285	2.533	1.686	1.555	456	0.239122266	isogroup02124	ENSG00000139626	ENST00000422257	ITGB7
isotig13449	12.724	14.443	20.958	14.867	377	0.314768543	isogroup02124	ENSG00000131069	ENST00000484354	ACSS2
isotig13450	3.406	2.394	3.119	2.848	946	0.313021342	isogroup02125	ENSG00000159322	ENST00000311669	ADPGK
isotig13451	3.342	1.652	2.955	2.103	749	0.564637033	isogroup02125	ENSG00000159322	ENST00000311669	ADPGK
isotig13455	4.021	3.168	2.915	3.46	517	0.040580146	isogroup02126	ENSG00000213121	ENST00000392385	AL590867.1
isotig13456	31.419	24.476	28.472	19.432	2144	0.557169159	isogroup02127	ENSG00000130119	ENST00000360845	GNL3L
isotig13457	5.597	3.337	4.214	2.615	986	0.590168708	isogroup02128	ENSG00000099330	ENST00000215061	OCEL1
isotig13458	5.616	3.495	4.587	3.365	620	0.536954235	isogroup02128	ENSG00000099330	ENST00000215061	OCEL1
isotig13459	5.169	2.783	3.78	2.841	604	0.564702788	isogroup02128	ENSG00000099330	ENST00000215061	OCEL1
isotig13460	10.252	12.401	10.193	11.596	1086	0.416387991	isogroup02129	ENSG00000149196	ENST00000278483	C11orf73
isotig13461	5.955	7.713	6.752	7.394	934	0.369946269	isogroup02129	ENSG00000149196	ENST00000533986	C11orf73
isotig13468	63.788	60.411	67.773	53.287	1575	0.333367776	isogroup02132	ENSG00000161057	ENST00000435765	PSMC2
isotig13473	12.557	10.564	14.767	13.71	854	0.255673706	isogroup02134	ENSG00000130304	ENST00000442725	SLC27A1
isotig13474	19.999	20.289	26.558	26.359	713	0.023211468	isogroup02134	ENSG00000130304	ENST00000442725	SLC27A1
isotig13475	11.373	10.234	13.829	14.369	504	0.140245735	isogroup02134	ENSG00000130304	ENST00000442725	SLC27A1
isotig13476	135.551	92.1	166.114	121.447	944	0.585781919	isogroup02135	ENSG00000243696	ENST00000446157	MUSTN1
isotig13477	145.552	98.91	178.398	130.428	879	0.586167055	isogroup02135	ENSG00000243696	ENST00000446157	MUSTN1
isotig13484	4.081	6.203	5.944	3.578	876	0.296451116	isogroup02138	ENSG00000122786	ENST00000436461	CALD1
isotig13485	3.767	5.771	4.441	3.386	790	0.396079131	isogroup02138	ENSG00000122786	ENST00000443197	CALD1
isotig13490	3.846	3.982	1.898	2.682	850	0.287076351	isogroup02140	ENSG00000160050	ENST00000373602	CCDC28B
contig21218	18.491	15.969	21.082	16.475	621	0.420718419	isogroup02315	ENSG00000103423	ENST00000355296	DNAJA3
isotig14083	10.303	6.816	8.378	7.423	659	0.534990982	isogroup02316	ENSG00000204822	ENST00000258105	MRPL53
isotig14085	13.096	9.725	11.69	9.774	552	0.468897948	isogroup02316	ENSG00000204822	ENST00000258105	MRPL53
contig21233	25.57	15.721	22.639	16.471	2172	0.669919967	isogroup02319	ENSG00000081154	ENST00000265260	PCNP
isotig14099	3.321	2.783	4.329	3.323	511	0.340751108	isogroup02321	ENSG00000124920	ENST00000278836	C11orf9
isotig14101	6.26	6.561	9.909	6.814	485	0.326040806	isogroup02321	ENSG00000168802	ENST00000306585	CHTF8
isotig14103	754.842	487.021	450.947	382.011	468	0.658008943	isogroup02322	ENSG00000134419	ENST00000565420	RPS15A
isotig14105	359.687	228.891	214.246	178.13	476	0.663945668	isogroup02322	ENSG00000134419	ENST00000565420	RPS15A
contig21251	15.214	17.351	14.809	12.195	995	0.053223867	isogroup02324	ENSG00000119878	ENST00000238892	CRIPT
isotig14107	5.774	4.659	5.456	5.499	5777	0.136572856	isogroup02327	ENSG00000114982	ENST00000431828	KANSL3
isotig14108	5.866	4.726	5.52	5.555	5707	0.137305553	isogroup02327	ENSG00000114982	ENST00000431828	KANSL3
isotig14109	5.796	4.776	5.551	5.602	5639	0.111435711	isogroup02327	ENSG00000114982	ENST00000431828	KANSL3
isotig14110	7.003	3.596	5.127	3.994	6485	0.578501916	isogroup02328	ENSG00000111642	ENST00000357008	CHD4
isotig14111	7.09	3.64	5.191	4.043	6405	0.580145788	isogroup02328	ENSG00000111642	ENST00000357008	CHD4
isotig14112	4.264	2.266	2.654	2.381	3422	0.435550838	isogroup02328	ENSG00000111642	ENST00000544040	CHD4
isotig14113	20.987	31.956	22.34	33.945	6661	0.728122417	isogroup02329	ENSG00000126934	ENST00000262948	MAP2K2
isotig14115	14.906	14.498	15.766	20.786	2713	0.260962275	isogroup02329	ENSG00000126934	ENST00000262948	MAP2K2
isotig14116	15.864	15.393	13.116	15.926	4707	0.113699557	isogroup02330	ENSG00000134294	ENST00000256689	SLC38A2
isotig14119	5.575	5.082	4.863	3.398	5536	0.349938003	isogroup02331	ENSG00000134884	ENST00000426600	ARGLU1
isotig14120	5.163	4.68	4.3	3.226	5109	0.328755542	isogroup02331	ENSG00000134884	ENST00000426600	ARGLU1
isotig14121	3.833	3.967	3.395	2.827	3249	0.106607425	isogroup02331	ENSG00000134884	ENST00000400198	ARGLU1
isotig14122	11.764	10.87	6.6	8.135	5608	0.108345232	isogroup02332	ENSG00000131791	ENST00000254101	PRKAB2
isotig14123	11.753	11.103	6.613	8.257	5571	0.150804088	isogroup02332	ENSG00000131791	ENST00000254101	PRKAB2
isotig14125	5.359	4.247	3.699	3.105	6234	0.304867739	isogroup02333	ENSG00000055917	ENST00000319801	PUM2
isotig14126	5.821	4.537	4.444	3.446	4522	0.368631172	isogroup02333	ENSG00000055917	ENST00000536417	PUM2
isotig14127	4.421	3.613	1.705	2.296	1965	0.003503795	isogroup02333	ENSG00000055917	ENST00000403432	PUM2
isotig14128	6.342	3.08	4.698	3.106	4264	0.716652889	isogroup02334	ENSG00000144597	ENST00000396842	EAF1
isotig14130	6.748	5.592	6.106	5.598	3741	0.278330954	isogroup02334	ENSG00000143093	ENST00000369795	FAM40A
isotig14131	5.986	7.305	5.994	6.865	6264	0.257730894	isogroup02335	ENSG00000136111	ENST00000377636	TBC1D4
isotig14132	6.078	7.375	6.284	6.98	5696	0.231851657	isogroup02335	ENSG00000136111	ENST00000377636	TBC1D4
isotig14133	10.953	193.061	9.15	6.792	4481	0.994232359	isogroup02336	ENSG00000108821	ENST00000225964	COL1A1
isotig14135	6.168	109.66	5.113	4.126	2835	0.991714887	isogroup02336	ENSG00000108821	ENST00000225964	COL1A1
isotig14136	11.846	10.771	16.292	15.071	4550	0.156909897	isogroup02337	ENSG00000142784	ENST00000319394	WDTC1
isotig14137	11.401	10.372	15.738	14.562	4492	0.155886	isogroup02337	ENSG00000142784	ENST00000447062	WDTC1
isotig14138	7.681	7.011	10.557	9.868	2770	0.176157286	isogroup02337	ENSG00000142784	ENST00000361771	WDTC1
isotig14139	7.573	11.597	8.337	9.244	4956	0.570160442	isogroup02338	ENSG00000137710	ENST00000343115	RDX
isotig14140	7.653	11.72	8.425	9.342	4904	0.570733449	isogroup02338	ENSG00000137710	ENST00000343115	RDX

isotig14141	5.239	6.135	4.032	4.907	1606	0.41561772	isogroup02338	ENSG00000137710	ENST00000528498	RDX
isotig14142	6.996	2.475	3.129	3.087	5344	0.682948824	isogroup02339	ENSG00000113389	ENST00000265074	NPR3
isotig14143	8.186	3.003	3.354	3.603	5188	0.688819794	isogroup02339	ENSG00000113389	ENST00000265074	NPR3
isotig14144	5.314	1.924	2.435	2.088	1024	0.680581649	isogroup02339	ENSG00000115266	ENST00000535453	APC2
isotig14145	11.034	8.541	8.606	6.13	3915	0.553609003	isogroup02340	ENSG00000117335	ENST00000322875	CD46
isotig14146	11.169	8.468	8.567	6.151	3825	0.5623544	isogroup02340	ENSG00000117335	ENST00000354848	CD46
isotig14147	11.409	8.591	8.73	6.277	3777	0.563697678	isogroup02340	ENSG00000117335	ENST00000357714	CD46
isotig14148	4.104	4.249	2.639	2.536	4457	0.006660029	isogroup02341	ENSG00000082898	ENST00000406957	XPO1
isotig14149	5.527	6.096	3.864	3.67	4282	0.029937251	isogroup02341	ENSG00000082898	ENST00000406957	XPO1
isotig14151	17.35	22.132	16.8	21.519	3876	0.478159991	isogroup02342	ENSG00000078295	ENST00000338316	ADCY2
isotig14152	16.708	20.999	15.953	20.481	3738	0.466775006	isogroup02342	ENSG00000078295	ENST00000338316	ADCY2
isotig14153	11.654	14.377	11.018	14.492	3068	0.432770346	isogroup02342	ENSG00000078295	ENST00000338316	ADCY2
isotig14154	11.805	9.929	8.121	9.688	6200	0.021999699	isogroup02343	ENSG00000166266	ENST00000531427	CUL5
isotig14155	4.689	3.951	4.67	4.615	3573	0.116282783	isogroup02343	ENSG00000140396	ENST00000518287	NCOA2
isotig14157	1.842	1.888	1.631	1.287	4011	0.078182536	isogroup02344	ENSG00000164944	ENST00000297591	KIAA1429
isotig14158	1.862	1.908	1.649	1.301	3969	0.078182536	isogroup02344	ENSG00000164944	ENST00000297591	KIAA1429
isotig14160	12.106	10.628	10.158	13.76	4907	0.197161269	isogroup02345	ENSG00000175727	ENST00000319080	MLXIP
isotig14161	12.102	10.987	10.642	13.974	3374	0.196973397	isogroup02345	ENSG00000175727	ENST00000538698	MLXIP
isotig14162	9.716	8.037	6.975	9.993	1967	0.217122567	isogroup02345	ENSG00000175727	ENST00000319080	MLXIP
isotig14163	5.325	3.875	3.266	3.143	5057	0.280886	isogroup02346	ENSG00000137075	ENST00000259605	RNF38
isotig14164	5.319	3.835	3.258	3.138	4951	0.290420455	isogroup02346	ENSG00000137075	ENST00000259605	RNF38
isotig14165	10.897	8.673	12.821	12.226	3434	0.209203803	isogroup02347	ENSG00000090861	ENST00000261772	AARS
isotig14166	11.225	8.932	13.199	12.601	3330	0.209654693	isogroup02347	ENSG00000090861	ENST00000261772	AARS
isotig14167	8.404	6.212	9.15	8.694	3060	0.281552942	isogroup02347	ENSG00000090861	ENST00000261772	AARS
isotig14168	8.086	6.345	7.674	6.547	3763	0.382101526	isogroup02348	ENSG00000067955	ENST00000290858	CBFB
isotig14169	3.324	2.134	4.153	2.6	3001	0.572612159	isogroup02348	ENSG00000067955	ENST00000290858	CBFB
isotig14171	4.621	3.68	4.725	3.965	3859	0.288588713	isogroup02349	ENSG00000168092	ENST00000527958	PAFAH1B2
isotig14177	3.75	2.907	3.469	3.148	3965	0.299889156	isogroup02351	ENSG00000123505	ENST00000368885	AMD1
isotig14178	4.164	3.471	3.977	3.74	3271	0.233645826	isogroup02351	ENSG00000123505	ENST00000368885	AMD1
isotig14180	7.923	4.484	6.172	5.301	3276	0.574594199	isogroup02352	ENSG00000139197	ENST00000412720	PEX5
isotig14181	7.702	4.413	6.173	5.189	3165	0.572659127	isogroup02352	ENSG00000139197	ENST00000412720	PEX5
isotig14182	7.555	4.265	6.077	5.172	3072	0.577863155	isogroup02352	ENSG00000139197	ENST00000266563	PEX5
isotig14183	3.496	5.159	4.158	2.43	4081	0.283666491	isogroup02353	ENSG00000145216	ENST00000507166	FIP1L1
isotig14186	3.35	2.235	2.658	2.572	3213	0.372106786	isogroup02354	ENSG00000082213	ENST00000325366	C5orf22
isotig14187	3.453	2.446	2.779	2.676	3143	0.342460735	isogroup02354	ENSG00000082213	ENST00000325366	C5orf22
isotig14188	3.392	2.273	2.754	2.526	2997	0.392781994	isogroup02354	ENSG00000082213	ENST00000325366	C5orf22
isotig14189	2.656	3.618	4.011	3.758	4516	0.292073721	isogroup02355	ENSG00000167601	ENST00000301178	AXL
isotig14190	2.695	3.698	4.19	3.92	4398	0.306145262	isogroup02355	ENSG00000167601	ENST00000301178	AXL
isotig14191	17.977	14.613	11.253	17.528	3875	0.187880439	isogroup02356	ENSG00000172508	ENST00000445895	CARNS1
isotig14193	4.48	4.39	2.62	4.377	1110	0.298311039	isogroup02356	ENSG00000172508	ENST00000542831	CARNS1
isotig14194	3.194	2.497	2.366	1.854	3190	0.275813482	isogroup02357	ENSG00000060339	ENST00000543225	CCAR1
isotig14195	2.817	2.099	2.215	1.636	2979	0.300837905	isogroup02357	ENSG00000060339	ENST00000543719	CCAR1
isotig14196	2.527	1.86	2.104	1.419	2763	0.316515744	isogroup02357	ENSG00000060339	ENST00000543719	CCAR1
isotig14197	3.011	1.884	2.792	2.569	4262	0.329760652	isogroup02358	ENSG00000172534	ENST00000369984	HCFC1
isotig14198	3.106	1.892	2.848	2.562	4068	0.351177951	isogroup02358	ENSG00000172534	ENST00000369984	HCFC1
isotig14200	2.57	3.074	3.024	3.797	3082	0.223341099	isogroup02359	ENSG00000137965	ENST00000370747	IFI44
isotig14201	2.143	2.863	2.612	2.894	2956	0.243067558	isogroup02359	ENSG00000137965	ENST00000370747	IFI44
isotig14202	2.347	2.988	2.787	3.1	2724	0.203191929	isogroup02359	ENSG00000137965	ENST00000370747	IFI44
isotig14203	4	2.766	3.686	3.122	4012	0.40805591	isogroup02360	ENSG00000178971	ENST00000315684	CTC1
isotig14204	4.12	2.721	3.877	3.009	3393	0.463703314	isogroup02360	ENSG00000178971	ENST00000315684	CTC1
isotig14205	2.29	1.743	1.929	1.988	1278	0.229071166	isogroup02360	ENSG00000178971	ENST00000315684	CTC1
isotig14206	3.952	2.215	3.318	2.356	4129	0.563350116	isogroup02361	ENSG00000076108	ENST00000549884	BAZ2A
isotig14207	4.075	2.203	3.164	2.177	2813	0.588289998	isogroup02361	ENSG00000076108	ENST00000549884	BAZ2A
isotig14208	3.788	1.975	3.382	2.336	1687	0.595579394	isogroup02361	ENSG00000076108	ENST00000549884	BAZ2A
isotig14209	4.473	3.555	2.91	2.85	4602	0.264935748	isogroup02362	ENSG00000058063	ENST00000323116	ATP11B
isotig14210	3.347	2.849	1.764	2.147	3752	0.088308785	isogroup02362	ENSG00000058063	ENST00000323116	ATP11B
isotig14211	8.827	7.551	9.206	9.676	3041	0.120237469	isogroup02363	ENSG00000160767	ENST00000361361	FAM189B
isotig14212	8.253	7.044	8.46	8.878	2984	0.132636958	isogroup02363	ENSG00000160767	ENST00000368368	FAM189B
isotig14213	7.912	6.951	8.034	8.363	2493	0.105527166	isogroup02363	ENSG00000160767	ENST00000361361	FAM189B
isotig14214	1.858	2.095	1.665	1.951	2794	0.205822124	isogroup02364	ENSG00000108381	ENST00000456349	ASPA
isotig14216	1.736	2.736	2.48	2.605	2703	0.272347261	isogroup02364	ENSG00000108381	ENST00000456349	ASPA
isotig14217	4.103	4.047	5.396	5.94	3716	0.080662433	isogroup02365	ENSG00000185033	ENST00000411539	SEMA4B
isotig14218	8.232	6.121	12.395	14.55	2881	0.007420906	isogroup02365	ENSG00000187764	ENST00000455551	SEMA4D
isotig14219	8.269	6.456	12.516	14.672	1850	0.020947622	isogroup02365	ENSG00000187764	ENST00000455551	SEMA4D
isotig14220	13.382	16.508	10.289	10.395	4166	0.268890434	isogroup02366	ENSG00000114166	ENST00000263754	KAT2B
isotig14221	12.863	15.942	9.934	9.91	4081	0.26758473	isogroup02366	ENSG00000114166	ENST00000263754	KAT2B
isotig14222	3.08	2.17	3.048	2.814	3592	0.295399038	isogroup02367	ENSG00000104365	ENST00000520810	IKBKB
isotig14223	3.149	1.969	2.976	2.678	2867	0.355874728	isogroup02367	ENSG00000104365	ENST00000520810	IKBKB
isotig14224	2.739	2.302	2.923	2.489	1794	0.275813482	isogroup02367	ENSG00000049759	ENST00000456986	NEDD4L
isotig14225	26.129	5.131	11.46	5.841	3934	0.946221913	isogroup02368	ENSG00000132002	ENST00000254322	DNAJB1
isotig14226	20.918	4.92	10.148	5.579	2530	0.927585106	isogroup02368	ENSG00000132002	ENST00000254322	DNAJB1
isotig14227	24.537	3.844	7.745	4.316	1800	0.945508003	isogroup02368	ENSG00000169660	ENST00000337014	HEXDC
isotig14228	11.833	7.033	12.407	9.188	3570	0.632768468	isogroup02369	ENSG00000214717	ENST00000381218	ZBED1
isotig14229	11.974	7.117	12.555	9.297	3528	0.633322687	isogroup02369	ENSG00000214717	ENST00000381218	ZBED1
isotig14230	5.259	3.812	3.572	3.123	829	0.408938904	isogroup02369	ENSG00000169084	ENST00000334651	DHRSX
isotig14231	1.785	1.401	1.251	1.243	2819	0.117795145	isogroup02370	ENSG00000159086	ENST00000331923	GCFC1
isotig14233	1.724	1.27	1.327	1.207	2421	0.160827008	isogroup02370	ENSG00000159086	ENST00000331923	GCFC1
isotig14234	11.201	7.058	18.795	17.25	3526	0.33840272	isogroup02371	ENSG00000104689	ENST00000221132	TNFRSF10A
isotig14235	6.538	3.515	7.731	8.857	2410	0.209626512	isogroup02371	ENSG00000181754	ENST00000369862	AMIGO1
isotig14236	8.273	4.512	10.675	11.663	2099	0.232922522	isogroup02371	ENSG00000181754	ENST00000369862	AMIGO1
isotig14237	4.094	3.355	4.144	2.956	2890	0.422850755	isogroup02372	ENSG00000146409	ENST00000275227	C6orf192
isotig14238	4.287	4.287	4.469	3.591	2581	0.205991208	isogroup02372	ENSG00000146409	ENST00000275227	C6orf192

isotig14239	4.17	3.571	4.032	3.013	2531	0.358730367	isogroup02372	ENSG00000146409	ENST00000275227	C6orf192
isotig14240	10.396	7.886	8.9	5.396	5665	0.529429999	isogroup02373	ENSG00000164983	ENST00000297632	TMEM65
isotig14241	5.954	5.369	3.841	2.989	1060	0.247379199	isogroup02373	ENSG00000164983	ENST00000297632	TMEM65
isotig14243	8.762	6.123	9.027	9.234	3690	0.163109642	isogroup02374	ENSG00000159082	ENST00000438952	SYNJ1
isotig14244	5.904	4.257	5.974	5.856	2743	0.111435711	isogroup02374	ENSG00000204571	ENST00000398530	KRTAP5-11
isotig14245	7.711	5.236	7.622	7.7	1554	0.143909221	isogroup02374	ENSG00000152291	ENST00000444342	TGOLN2
isotig14246	2.93	4.004	4.254	4.297	4202	0.291923424	isogroup02375	ENSG00000115159	ENST00000438166	GPD2
isotig14247	4.736	5.112	8.293	6.323	3578	0.127489291	isogroup02375	ENSG00000115159	ENST00000438166	GPD2
isotig14248	6.564	8.314	6.369	6.847	3505	0.333912602	isogroup02376	ENSG00000197969	ENST00000376636	VPS13A
isotig14249	4.6	5.448	3.539	4.204	3230	0.325167205	isogroup02376	ENSG00000197969	ENST00000357409	VPS13A
isotig14251	2.747	2.162	2.214	2.205	3799	0.09882017	isogroup02377	ENSG00000140382	ENST00000381714	HMG20A
isotig14626	4.035	2.993	4.233	2.743	1039	0.445385887	isogroup02518	ENSG00000196655	ENST00000533632	TRAPPC4
isotig14627	4.187	3.043	4.367	2.795	981	0.464943263	isogroup02518	ENSG00000196655	ENST00000533632	TRAPPC4
isotig14628	3.403	2.49	3.202	2.072	767	0.487741414	isogroup02518	ENSG00000196655	ENST00000533058	TRAPPC4
isotig14632	5.04	4.216	4.869	3.778	1280	0.304275945	isogroup02520	ENSG00000130489	ENST00000543927	SCO2
isotig14633	3.362	3.955	3.295	3.167	1191	0.129828286	isogroup02520	ENSG00000130489	ENST00000543927	SCO2
isotig14634	2	1.789	1.473	1.528	564	0.010060494	isogroup02520	ENSG00000055118	ENST00000430723	KCNH2
isotig14641	24.891	25.208	30.555	11.414	1725	0.711552191	isogroup02523	ENSG00000147872	ENST00000276914	PLIN2
isotig14643	3.002	1.584	2.662	4.123	1630	0.108279477	isogroup02524	ENSG00000128596	ENST00000494552	CCDC136
isotig14644	2.45	1.035	1.977	1.992	766	0.319023822	isogroup02524	ENSG00000128596	ENST00000494552	CCDC136
isotig14649	42.211	45.507	56.286	52.456	1326	0.056699481	isogroup02526	ENSG00000110717	ENST00000313468	NDUFS8
isotig14650	70.279	76.58	93.504	89.601	822	0.023850229	isogroup02526	ENSG00000110717	ENST00000313468	NDUFS8
isotig14651	84.388	95.028	113.893	104.62	779	0.024235365	isogroup02526	ENSG00000110717	ENST00000313468	NDUFS8
isotig14652	15.129	12.807	16.163	10.616	2326	0.546188096	isogroup02527	ENSG00000116120	ENST00000281828	FARSB
isotig14653	4.276	2.624	2.597	2.906	1429	0.405735703	isogroup02528	ENSG00000122257	ENST00000452655	RBBP6
isotig14654	3.806	2.322	2.238	2.401	1254	0.395543699	isogroup02528	ENSG00000122257	ENST00000567686	RBBP6
isotig14655	31.836	20.602	29.179	25.4	1408	0.528396708	isogroup02529	ENSG00000129055	ENST00000511751	ANAPC13
isotig14656	24.592	15.65	22.085	18.125	1316	0.53173142	isogroup02529	ENSG00000129055	ENST00000511751	ANAPC13
isotig14657	1.357	1.802	1.207	1.348	1035	0.190219433	isogroup02530	ENSG00000110315	ENST00000528665	RNF141
isotig14658	1.701	2.807	1.71	1.862	990	0.339952656	isogroup02530	ENSG00000110315	ENST00000265981	RNF141
isotig14659	1.325	1.697	1.165	1.376	819	0.241968513	isogroup02530	ENSG00000110315	ENST00000265981	RNF141
isotig14663	31.332	27.641	39.338	35.111	1219	0.196043436	isogroup02532	ENSG00000103264	ENST00000311635	FBXO31
isotig14664	27.443	23.796	32.786	29.453	1132	0.209682874	isogroup02532	ENSG00000103264	ENST00000311635	FBXO31
isotig14665	7.293	5.674	7.432	6.751	482	0.396727286	isogroup02532	ENSG00000103264	ENST00000311635	FBXO31
isotig14666	11.423	14.232	13.258	11.231	1195	0.021802435	isogroup02533	ENSG00000111843	ENST00000541412	TMEM14C
isotig14667	14.085	17.6	16.392	13.904	962	0.022760577	isogroup02533	ENSG00000111843	ENST00000541412	TMEM14C
isotig14668	76.095	67.691	136.378	50.586	1244	0.835434358	isogroup02534	ENSG00000104823	ENST00000221418	ECH1
isotig14670	5.224	4.325	3.43	3.447	944	0.259468701	isogroup02535	ENSG00000112208	ENST00000370693	BAG2
isotig14671	6.602	5.745	4.132	4.541	931	0.183540618	isogroup02535	ENSG00000112208	ENST00000370693	BAG2
isotig14672	5.773	4.763	3.577	3.846	913	0.234744871	isogroup02535	ENSG00000112208	ENST00000370693	BAG2
isotig14673	3.796	2.86	3.359	2.874	984	0.403640941	isogroup02536	ENSG00000177225	ENST00000319863	PDDC1
isotig14674	3.279	2.528	3.917	3.181	956	0.37357218	isogroup02536	ENSG00000177225	ENST00000319863	PDDC1
isotig14675	3.672	2.904	4.521	3.818	861	0.360327271	isogroup02536	ENSG00000177225	ENST00000319863	PDDC1
isotig14676	10.451	10.987	9.144	12.75	1081	0.329065529	isogroup02537	ENSG00000174099	ENST00000535664	MSRB3
isotig14677	29.089	32.508	32.312	43.077	955	0.342301045	isogroup02537	ENSG00000174099	ENST00000535664	MSRB3
isotig14678	14.797	19.918	14.505	21.951	754	0.621787405	isogroup02537	ENSG00000174099	ENST00000535664	MSRB3
isotig14682	10.942	8.432	11.063	7.312	1244	0.576914406	isogroup02539	ENSG00000138085	ENST00000380171	C2orf28
isotig14683	9.105	7.723	10.029	6.377	1177	0.520158563	isogroup02539	ENSG00000138085	ENST00000380171	C2orf28
isotig14684	9.226	8.616	8.166	6.67	1341	0.147337867	isogroup02540	ENSG00000117448	ENST00000372070	AKR1A1
isotig14685	10.198	9.82	9.044	7.356	1225	0.13615954	isogroup02540	ENSG00000117448	ENST00000372070	AKR1A1
isotig14686	1.014	1.294	1.618	1.657	1250	0.148934771	isogroup02541	ENSG00000187626	ENST00000423974	ZKSCAN4
isotig14691	4.288	2.97	3.225	2.74	1397	0.355583527	isogroup02543	ENSG00000112237	ENST00000518714	CCNC
isotig14692	4.342	4.269	5.278	4.822	1235	0.067426918	isogroup02544	ENSG00000092094	ENST00000206542	OSGEP
isotig14693	3.46	3.289	4.074	3.888	1192	0.087961223	isogroup02544	ENSG00000092094	ENST00000206542	OSGEP
isotig14694	4.785	3.87	4.438	3.587	1323	0.448241527	isogroup02545	ENSG00000140326	ENST00000356231	CDAN1
isotig14695	4.05	2.9	3.727	2.72	1070	0.482095889	isogroup02545	ENSG00000140326	ENST00000356231	CDAN1
isotig14699	64.945	46.53	55.466	49.195	1028	0.56838506	isogroup02547	ENSG00000143106	ENST00000271308	PSMA5
isotig15190	2.677	2.451	2.507	2.279	1138	0.18122041	isogroup02802	ENSG00000182004	ENST00000414487	SNRPE
isotig15191	7.57	7.505	7.79	6.56	525	0.185748103	isogroup02802	ENSG00000182004	ENST00000414487	SNRPE
isotig15192	62.948	37.505	82.544	76.042	1046	0.560851432	isogroup02803	ENSG00000171161	ENST00000306576	ZNF672
isotig15195	9.851	9.759	11.594	9.62	1085	0.235468175	isogroup02804	ENSG00000164032	ENST00000296417	H2AFZ
isotig15196	10.504	10.448	12.425	10.3	1008	0.23725295	isogroup02804	ENSG00000164032	ENST00000296417	H2AFZ
isotig15197	11.15	11.059	13.181	10.906	955	0.238831066	isogroup02804	ENSG00000164032	ENST00000296417	H2AFZ
isotig15198	73.251	89.427	84.883	77.534	708	0.121496205	isogroup02805	ENSG00000151366	ENST00000281031	NDUFC2
isotig15200	81.405	99.385	94.295	86.155	637	0.121806192	isogroup02805	ENSG00000151366	ENST00000281031	NDUFC2
isotig15204	337.513	390.13	295.677	300.458	563	0.187035019	isogroup02807	ENSG00000156885	ENST00000287490	COX6A2
isotig15205	379.603	439.552	334.417	339.38	489	0.187119561	isogroup02807	ENSG00000156885	ENST00000287490	COX6A2
isotig15206	396.748	458.876	347.739	353.328	476	0.187138348	isogroup02807	ENSG00000156885	ENST00000287490	COX6A2
contig23168	108.015	81.838	87.248	112.851	1586	0.024085068	isogroup02808	ENSG00000139718	ENST00000542440	SETD1B
contig23169	566.989	534.659	819.016	840.415	627	0.091718644	isogroup02808	ENSG00000101306	ENST00000375994	MYLK2
isotig15216	97.011	40.978	50.904	49.018	625	0.795389645	isogroup02812	ENSG00000150768	ENST00000534998	DLAT
isotig15219	1226.241	624.583	764.263	580.485	456	0.803195686	isogroup02813	ENSG00000130255	ENST00000394580	RPL36
isotig15224	375.48	471.637	380.169	315.896	433	0.13846096	isogroup02816	ENSG00000156411	ENST00000414262	C14orf2
isotig15229	172.024	224.685	142.442	153.984	378	0.366339145	isogroup02817	ENSG00000184076	ENST00000330029	UQCR10
contig23199	47.572	43.392	46.915	36.918	1348	0.345306981	isogroup02818	ENSG00000131100	ENST00000253413	ATP6V1E1
contig23202	10.072	7.522	8.64	6.534	1045	0.572790637	isogroup02819	ENSG00000260380	ENST00000564393	FRG1.1
isotig15230	1.532	2.063	1.124	1.008	378	0.172390471	isogroup02821	ENSG00000089048	ENST00000202816	ESF1
contig23211	22.63	17.82	21.257	15.587	721	0.489394679	isogroup02822	ENSG00000168002	ENST00000301788	POLR2G
isotig15231	6.992	4.471	5.121	5.632	14099	0.273671752	isogroup02823	ENSG00000086758	ENST00000342160	HUWE1
isotig15232	6.989	4.459	5.094	5.62	14054	0.272986022	isogroup02823	ENSG00000086758	ENST00000342160	HUWE1
isotig15233	6.46	4.936	4.848	5.378	12875	0.149188397	isogroup02824	ENSG00000164142	ENST00000505231	FAM160A1
isotig15234	6.477	4.909	4.825	5.4	12752	0.149188397	isogroup02824	ENSG00000164142	ENST00000505231	FAM160A1

isotig15235	4.794	4.369	3.748	4.387	11534	0.127714737	isogroup02825	ENSG00000128731	ENST00000261609	HERC2
isotig15236	4.969	4.23	3.545	3.861	10009	0.042524611	isogroup02825	ENSG00000128731	ENST00000261609	HERC2
isotig15237	14.87	10.685	10.976	7.193	8742	0.615474938	isogroup02826	ENSG00000166025	ENST00000433060	AMOTL1
isotig15238	14.579	10.622	10.927	7.184	8467	0.607791012	isogroup02826	ENSG00000166025	ENST00000317829	AMOTL1
isotig15239	5.997	11.214	6.803	5.379	8527	0.501737807	isogroup02827	ENSG00000155380	ENST00000538576	SLC16A1
isotig15240	6.057	11.222	6.806	5.418	8430	0.502057188	isogroup02827	ENSG00000155380	ENST00000538576	SLC16A1
isotig15241	8.41	7.657	9.575	8.015	8531	0.294619373	isogroup02828	ENSG00000162434	ENST00000342505	JAK1
isotig15242	8.681	8.016	9.835	8.11	8019	0.295154806	isogroup02828	ENSG00000162434	ENST00000342505	JAK1
isotig15243	3.665	3.034	4.119	3.184	8472	0.354418727	isogroup02829	ENSG00000083290	ENST00000395544	ULK2
isotig15244	3.583	2.821	3.857	3.056	7970	0.370866837	isogroup02829	ENSG00000083290	ENST00000395544	ULK2
isotig15245	5.872	4.75	5.625	4.174	8123	0.414302623	isogroup02830	ENSG00000011523	ENST00000377990	CEP68
isotig15246	5.674	4.596	5.371	4.007	8059	0.402400992	isogroup02830	ENSG00000011523	ENST00000377990	CEP68
isotig15247	3.242	4.487	2.786	2.51	8081	0.369936875	isogroup02831	ENSG00000127329	ENST00000261266	PTPRB
isotig15248	3.272	4.309	2.75	2.432	7817	0.312110168	isogroup02831	ENSG00000127329	ENST00000538708	PTPRB
isotig15249	9.409	11.747	4.231	6.233	7512	0.541979785	isogroup02832	ENSG00000153956	ENST00000356253	CACNA2D1
isotig15250	9.189	11.489	3.983	6.01	7470	0.547540768	isogroup02832	ENSG00000153956	ENST00000356253	CACNA2D1
isotig15251	8.936	9.551	7.012	6.952	7107	0.079150071	isogroup02833	ENSG00000107863	ENST00000396432	ARHGAP21
isotig15253	15.757	12.259	13.94	14.247	12007	0.256866687	isogroup02834	ENSG00000127481	ENST00000375254	UBR4
isotig15254	16.056	13.389	19.06	15.843	1288	0.343484632	isogroup02834	ENSG00000127481	ENST00000543981	UBR4
isotig15255	23.932	18.05	15.776	15.777	6635	0.27789885	isogroup02835	ENSG00000023287	ENST00000025008	RB1CC1
isotig15257	6.528	5.917	7.76	10.091	6617	0.060381754	isogroup02836	ENSG00000105835	ENST00000222553	NAMPT
isotig15258	6.411	5.908	7.819	10.106	6589	0.047550162	isogroup02836	ENSG00000105835	ENST00000222553	NAMPT
isotig15259	3.657	2.942	2.953	2.538	6509	0.282623807	isogroup02837	ENSG00000049618	ENST00000367148	ARID1B
isotig15260	3.632	2.952	2.964	2.544	6467	0.280425716	isogroup02837	ENSG00000049618	ENST00000367148	ARID1B
isotig15261	5.066	3.425	4.302	3.34	6455	0.44935936	isogroup02838	ENSG00000167522	ENST00000378330	ANKRD11
isotig15262	5.192	3.508	4.397	3.43	6291	0.454150071	isogroup02838	ENSG00000167522	ENST00000378330	ANKRD11
isotig15263	26.483	30.318	24.582	25.67	6630	0.179022319	isogroup02839	ENSG00000167978	ENST00000544933	SRRM2
isotig15264	30.094	33.591	28.08	29.089	6001	0.132909371	isogroup02839	ENSG00000167978	ENST00000544933	SRRM2
isotig15265	4.335	4.074	5.926	5	6286	0.182469753	isogroup02840	ENSG00000163113	ENST00000369135	OTUD7B
isotig15266	4.314	3.999	5.911	4.989	6152	0.20393402	isogroup02840	ENSG00000163113	ENST00000369135	OTUD7B
isotig15267	4.057	2.782	4.154	3.223	6478	0.459551364	isogroup02841	ENSG00000120733	ENST00000314358	KDM3B
isotig15268	3.982	2.701	4.148	3.041	5878	0.498431277	isogroup02841	ENSG00000120733	ENST00000545151	KDM3B
isotig15269	7.027	4.548	6.962	6.193	6190	0.39472646	isogroup02842	ENSG00000136848	ENST00000408936	DAB2IP
isotig15270	6.931	4.511	6.906	6.163	6123	0.392349891	isogroup02842	ENSG00000136848	ENST00000408936	DAB2IP
isotig15271	4.541	3.3	3.606	2.517	6083	0.34395431	isogroup02843	ENSG00000198561	ENST00000524630	CTNND1
isotig15272	4.415	3.173	3.497	2.454	6020	0.346133614	isogroup02843	ENSG00000198561	ENST00000524630	CTNND1
isotig15273	23.192	28.45	24.957	23.865	6111	0.238276847	isogroup02844	ENSG00000138095	ENST00000260665	LRPPRC
isotig15274	21.82	26.756	23.149	22.509	5968	0.252357782	isogroup02844	ENSG00000138095	ENST00000260665	LRPPRC
isotig15275	6.524	5.529	6.517	6.006	6087	0.205831517	isogroup02845	ENSG00000010404	ENST00000340855	IDS
isotig15276	6.112	5.261	6.295	5.747	5886	0.202543774	isogroup02845	ENSG00000010404	ENST00000340855	IDS
isotig15277	5.071	6.348	3.501	3.386	6096	0.338468475	isogroup02846	ENSG00000128567	ENST00000544955	PODXL
isotig15278	5.334	6.553	3.462	3.357	5841	0.331009995	isogroup02846	ENSG00000128567	ENST00000544955	PODXL
isotig15279	4.471	2.538	4.03	2.934	6142	0.540495604	isogroup02847	ENSG00000117713	ENST00000457599	ARID1A
isotig15280	3.965	2.243	3.485	2.658	5778	0.499690013	isogroup02847	ENSG00000117713	ENST00000457599	ARID1A
isotig15281	5.29	24.679	3.117	7.418	5942	0.941619073	isogroup02848	ENSG00000162390	ENST00000343744	ACOT11
isotig15283	4.195	2.237	4.293	3.387	5972	0.583424138	isogroup02849	ENSG00000079432	ENST00000160740	CIC
isotig15284	4.177	2.265	4.267	3.36	5874	0.576839258	isogroup02849	ENSG00000079432	ENST00000160740	CIC
isotig15285	8.641	6.49	6.967	6.671	6817	0.184479973	isogroup02850	ENSG00000173120	ENST00000529006	KDM2A
isotig15286	4.458	2.475	2.719	2.567	5019	0.435823251	isogroup02850	ENSG00000173120	ENST00000529006	KDM2A
isotig15287	9.872	8.416	9.218	8.632	5887	0.297484407	isogroup02851	ENSG00000214655	ENST00000398706	KIAA0913
isotig15289	2.87	2.263	2.555	2.34	5908	0.18420756	isogroup02852	ENSG00000116698	ENST00000367537	SMG7
isotig15290	2.928	2.311	2.619	2.369	5770	0.187241677	isogroup02852	ENSG00000116698	ENST00000515829	SMG7
isotig15291	16.303	11.918	17.373	14.956	6191	0.490644022	isogroup02853	ENSG00000132361	ENST00000322335	KIAA0664
isotig15292	17.307	13.393	19.123	16.886	5458	0.418698805	isogroup02853	ENSG00000132361	ENST00000322335	KIAA0664
isotig15293	4.373	4.314	3.593	3.458	5804	0.040580146	isogroup02854	ENSG00000078674	ENST00000325083	PCM1
isotig15294	4.351	4.329	3.637	3.457	5714	0.038353874	isogroup02854	ENSG00000078674	ENST00000325083	PCM1
isotig15295	4.376	3.68	4.267	3.67	5853	0.329892162	isogroup02855	ENSG00000184863	ENST00000401878	RBM33
isotig15296	4.399	3.694	4.29	3.714	5645	0.322771849	isogroup02855	ENSG00000184863	ENST00000401878	RBM33
isotig15297	5.112	3.398	4.856	3.636	5831	0.465929586	isogroup02856	ENSG00000175866	ENST00000428708	BAIAP2
isotig15298	5.049	3.303	4.834	3.521	5621	0.486097543	isogroup02856	ENSG00000175866	ENST00000428708	BAIAP2
isotig15299	5.169	5.544	5.021	3.788	5981	0.167458856	isogroup02857	ENSG00000165282	ENST00000378617	PIGO
isotig15300	5.218	5.631	5.078	3.861	5456	0.168586083	isogroup02857	ENSG00000165282	ENST00000378617	PIGO
isotig15301	3.274	2.934	2.395	1.924	5747	0.19021004	isogroup02858	ENSG00000031081	ENST00000264245	ARHGAP31
isotig15302	3.269	2.904	2.385	1.905	5664	0.210312242	isogroup02858	ENSG00000031081	ENST00000264245	ARHGAP31
isotig15303	10.265	5.732	8.028	5.774	6570	0.688087097	isogroup02859	ENSG00000129933	ENST00000392313	MAU2
isotig15304	10.418	6.077	9.107	6.697	4645	0.660704892	isogroup02859	ENSG00000129933	ENST00000392313	MAU2
isotig15305	5.987	5.353	6.062	6.349	5624	0.045004509	isogroup02860	ENSG00000107929	ENST00000316157	LARP4B
isotig15306	5.815	5.279	5.956	6.242	5591	0.0314684	isogroup02860	ENSG00000107929	ENST00000316157	LARP4B
isotig15307	4.379	4.931	6.299	7.037	8513	0.250995717	isogroup02861	ENSG00000165699	ENST00000440111	TSC1
isotig15308	2.018	2.489	2.214	2.437	2627	0.272347261	isogroup02861	ENSG00000165699	ENST00000440111	TSC1
isotig15309	7.576	24.945	7.266	14.1	8136	0.952440445	isogroup02862	ENSG00000166147	ENST00000316623	FBN1
isotig15310	4.372	8.667	8.346	5.447	2990	0.492015481	isogroup02862	ENSG00000115380	ENST00000394554	EFEMP1
isotig15311	9.751	5.122	7.153	5.759	5489	0.643063801	isogroup02863	ENSG00000173482	ENST00000400060	PTPRM
isotig15312	9.938	5.244	7.259	5.882	5450	0.638986999	isogroup02863	ENSG00000173482	ENST00000400060	PTPRM
isotig15313	5.433	4.504	4.777	4.001	5525	0.195329526	isogroup02864	ENSG00000168056	ENST00000301873	LTBP3
isotig15314	5.398	4.475	4.765	4.04	5383	0.193140828	isogroup02864	ENSG00000168056	ENST00000536982	LTBP3
isotig15315	1.53	2.56	3.228	3.215	5944	0.164330803	isogroup02865	ENSG00000131747	ENST00000357601	TOP2A
isotig15316	1.24	1.975	2.574	2.427	4728	0.104597204	isogroup02865	ENSG00000131747	ENST00000357601	TOP2A
isotig15317	74.658	114.413	87.058	88.377	5435	0.573495153	isogroup02866	ENSG00000152556	ENST00000547587	PFKM
isotig15318	79.06	120.8	92.452	93.756	5191	0.564270685	isogroup02866	ENSG00000177981	ENST00000536549	ASB8
isotig15319	5.881	1.818	4.562	2.072	5349	0.818591719	isogroup02867	ENSG00000111670	ENST00000299314	GNPTAB
isotig15320	5.856	1.805	4.556	2.071	5263	0.819465319	isogroup02867	ENSG00000111670	ENST00000299314	GNPTAB

isotig15321	6.717	9.247	8.462	8.559	6581	0.355931089	isogroup02868	ENSG00000154822	ENST00000285094	PLCL2
isotig15322	10.076	14.868	11.899	12.013	3972	0.453586458	isogroup02868	ENSG00000154822	ENST00000285094	PLCL2
isotig15323	6.24	3.842	5.106	4.929	5316	0.403199444	isogroup02869	ENSG00000108443	ENST00000225577	RPS6KB1
isotig15324	6.462	3.986	5.26	5.126	5195	0.397723003	isogroup02869	ENSG00000108443	ENST00000225577	RPS6KB1
isotig15325	12.649	7.766	8.659	7.675	6070	0.582578718	isogroup02870	ENSG00000073711	ENST00000264977	PPP2R3A
isotig15326	15.975	9.953	11.657	10.028	4436	0.597373563	isogroup02870	ENSG00000073711	ENST00000334546	PPP2R3A
isotig15327	2.803	2.961	2.879	2.62	5301	0.079835801	isogroup02871	ENSG00000205726	ENST00000381285	ITSN1
isotig15328	2.508	2.711	2.455	2.36	5080	0.129912828	isogroup02871	ENSG00000205726	ENST00000381285	ITSN1
isotig15329	6.537	6.913	5.289	3.934	5208	0.175424589	isogroup02872	ENSG00000163681	ENST00000449503	SLMAP
isotig15330	6.04	6.337	4.859	3.627	5118	0.171638987	isogroup02872	ENSG00000163681	ENST00000449503	SLMAP
isotig15331	18.068	11.043	10.15	13.097	6381	0.361642369	isogroup02873	ENSG00000182831	ENST00000327827	C16orf72
isotig15332	14.1	9.346	12.235	10.356	3902	0.460772526	isogroup02873	ENSG00000182831	ENST00000327827	C16orf72
isotig15333	4.315	4.59	3.891	3.369	5163	0.040580146	isogroup02874	ENSG00000153201	ENST00000283195	RANBP2
isotig15334	4.354	4.541	3.933	3.355	5085	0.076068986	isogroup02874	ENSG00000153201	ENST00000283195	RANBP2
isotig15335	12.435	16.673	6.015	5.184	6892	0.486463891	isogroup02875	ENSG00000248713	ENST00000511828	RP11-766F14.2.1
isotig15336	7.903	9.86	3.497	3.406	3332	0.33824303	isogroup02875	ENSG00000248713	ENST00000511828	RP11-766F14.2.1
isotig15337	4.889	3.998	4.96	5.134	5139	0.081930563	isogroup02876	ENSG00000186575	ENST00000338641	NF2
isotig15338	4.835	3.941	4.928	5.073	5064	0.083020215	isogroup02876	ENSG00000186575	ENST00000338641	NF2
isotig15339	4.814	4.038	5.273	4.609	5153	0.261760727	isogroup02877	ENSG00000136463	ENST00000258975	TACO1
isotig15340	4.016	3.251	4.407	3.717	4884	0.290420455	isogroup02877	ENSG00000136463	ENST00000258975	TACO1
isotig15341	10.627	11.106	9.038	8.547	5558	0.019651311	isogroup02878	ENSG00000136877	ENST00000373247	FPGS
isotig15342	5.89	6.137	5.416	4.647	4401	0.097862028	isogroup02878	ENSG00000136877	ENST00000373247	FPGS
isotig15343	7.531	13.554	4.287	5.254	5390	0.848040505	isogroup02879	ENSG00000142798	ENST00000374695	HSPG2
isotig15344	5.511	10.409	2.866	3.982	4481	0.850999474	isogroup02879	ENSG00000142798	ENST00000374695	HSPG2
isotig15345	6.106	3.799	5.094	3.742	4957	0.568497783	isogroup02880	ENSG00000198815	ENST00000545068	FOXJ3
isotig15346	5.987	3.719	5.049	3.636	4855	0.578661607	isogroup02880	ENSG00000198815	ENST00000545068	FOXJ3
isotig15347	3.119	2.972	2.623	2.507	4934	0.038353874	isogroup02881	ENSG00000136754	ENST00000376142	ABI1
isotig15348	3.138	2.936	2.656	2.503	4853	0.064064026	isogroup02881	ENSG00000136754	ENST00000359188	ABI1
isotig15349	2.851	2.705	2.378	1.593	4918	0.064064026	isogroup02882	ENSG00000138434	ENST00000431877	SSFA2
isotig15350	2.861	2.675	2.407	1.601	4852	0.081207259	isogroup02882	ENSG00000138434	ENST00000431877	SSFA2
isotig15351	11.618	10.051	10.131	11.49	4942	0.036531525	isogroup02883	ENSG00000048828	ENST00000277165	FAM120A
isotig15352	11.438	9.697	9.932	11.086	4804	0.06621515	isogroup02883	ENSG00000048828	ENST00000340893	FAM120A
isotig15353	2.128	1.928	1.781	1.313	4903	0.083029608	isogroup02884	ENSG00000107186	ENST00000546205	MPDZ
isotig15354	2.172	1.963	1.872	1.323	4814	0.106814083	isogroup02884	ENSG00000107186	ENST00000538841	MPDZ
isotig15355	2.891	2.194	2.54	1.844	4928	0.313040129	isogroup02885	ENSG00000100796	ENST00000554684	SMEK1
isotig15356	2.911	2.262	2.565	1.905	4772	0.293557902	isogroup02885	ENSG00000100796	ENST00000554684	SMEK1
isotig15357	10.116	9.433	8.684	8.763	4882	0.078708574	isogroup02886	ENSG00000109171	ENST00000264313	SLAIN2
isotig15358	9.838	9.127	8.464	8.495	4804	0.079666717	isogroup02886	ENSG00000109171	ENST00000264313	SLAIN2
isotig15359	6.06	4.477	5.782	4.607	4881	0.39060269	isogroup02887	ENSG00000153560	ENST00000283629	UBP1
isotig15360	5.753	4.239	5.468	4.317	4773	0.394256782	isogroup02887	ENSG00000153560	ENST00000447368	UBP1
isotig15363	2.759	1.324	3.13	2.479	4832	0.473350492	isogroup02889	ENSG00000184271	ENST00000550824	POU6F1
isotig15364	2.709	1.304	3.13	2.345	4773	0.485721801	isogroup02889	ENSG00000184271	ENST00000550824	POU6F1
isotig15365	6.111	4.1	4.828	4.771	4861	0.390987826	isogroup02890	ENSG00000141736	ENST00000269571	ERBB2
isotig15366	6.433	4.306	5.063	4.952	4584	0.397723003	isogroup02890	ENSG00000141736	ENST00000269571	ERBB2
isotig15367	5.521	9.313	5.153	6.465	7132	0.657464117	isogroup02891	ENSG00000102755	ENST00000282397	FLT1
isotig15368	5.173	5.482	3.189	3.838	2307	0.247379199	isogroup02891	ENSG00000102755	ENST00000541932	FLT1
isotig15369	5.916	6.886	4.623	5.397	5776	0.388893064	isogroup02892	ENSG00000136381	ENST00000258886	IREB2
isotig15370	47.761	47.375	46.095	45.216	3568	0.073344856	isogroup02892	ENSG00000136381	ENST00000258886	IREB2
isotig15371	6.99	8.212	5.379	3.63	5446	0.006660029	isogroup02893	ENSG00000130508	ENST00000252804	PXDN
isotig15372	5.489	6.84	4.924	3.282	3864	0.032651988	isogroup02893	ENSG00000130508	ENST00000252804	PXDN
isotig15373	6.045	3.513	5.573	4.47	4670	0.567680544	isogroup02894	ENSG00000099290	ENST00000282633	FAM21A
isotig15374	6.136	3.578	5.637	4.556	4607	0.567680544	isogroup02894	ENSG00000099290	ENST00000351071	FAM21A
isotig15375	5.48	2.596	5.236	2.899	5723	0.76843015	isogroup02895	ENSG00000077044	ENST00000409813	DGKD
isotig15376	4.023	2.078	4.278	2.129	3522	0.728188172	isogroup02895	ENSG00000077044	ENST00000409813	DGKD
isotig15377	5.444	2.531	3.54	3.544	4637	0.531515368	isogroup02896	ENSG00000175591	ENST00000393597	P2RY2
isotig15378	5.471	2.591	3.58	3.583	4552	0.526818592	isogroup02896	ENSG00000175591	ENST00000393597	P2RY2
isotig15379	4.25	3.877	5.054	4.705	7049	0.176786654	isogroup02897	ENSG00000170653	ENST00000456903	ATF7
isotig15381	1.974	9.474	1.597	3.694	4867	0.877545653	isogroup02898	ENSG00000114770	ENST00000443376	ABCC5
isotig15382	2.089	9.597	1.734	3.751	4196	0.878372285	isogroup02898	ENSG00000114770	ENST00000427120	ABCC5
isotig15383	4.094	4.26	5.039	4.693	5398	0.056474036	isogroup02899	ENSG00000141458	ENST00000269228	NPC1
isotig15384	1.884	3.006	3.225	3.053	3641	0.291472533	isogroup02899	ENSG00000141458	ENST00000269228	NPC1
isotig15385	3.095	2.499	4.061	3.015	4856	0.40176223	isogroup02900	ENSG00000236178	ENST00000553245	VARSL.5
isotig15386	2.49	1.958	3.213	2.29	4126	0.374596077	isogroup02900	ENSG00000236178	ENST00000553245	VARSL.5
isotig15387	22.759	17.518	22.528	19.129	7608	0.399845946	isogroup02901	ENSG00000186660	ENST00000316059	ZFP91
isotig15388	5.435	2.654	5.081	3.005	1341	0.712491546	isogroup02901	ENSG00000242689	ENST00000361987	CNTF
isotig15389	9.428	5.742	8.574	5.91	4515	0.631678816	isogroup02902	ENSG00000110048	ENST00000263847	OSBP
isotig15390	9.83	5.974	8.894	6.196	4419	0.634402946	isogroup02902	ENSG00000110048	ENST00000263847	OSBP
isotig15391	10.784	3.792	7.593	4.662	4478	0.8521267	isogroup02903	ENSG00000180155	ENST00000398906	LYNX1
isotig15392	10.871	3.842	7.702	4.758	4409	0.850999474	isogroup02903	ENSG00000180155	ENST00000398906	LYNX1
isotig15393	8.84	7.309	6.31	5.454	4499	0.333424138	isogroup02904	ENSG00000177189	ENST00000379565	RPS6KA3
isotig15394	9.217	7.653	6.545	5.644	4304	0.335368603	isogroup02904	ENSG00000177189	ENST00000379565	RPS6KA3
isotig15395	4.949	3.017	5.259	3.82	4413	0.585143158	isogroup02905	ENSG00000135597	ENST00000450536	REPS1
isotig15396	5.133	3.17	5.42	3.974	4332	0.589389043	isogroup02905	ENSG00000135597	ENST00000450536	REPS1
isotig15397	7.638	6.85	10.776	8.628	6793	0.271201247	isogroup02906	ENSG00000161204	ENST00000429586	ABCF3
isotig15398	4.699	5.262	5.464	5.37	1955	0.084175622	isogroup02906	ENSG00000161204	ENST00000429586	ABCF3
isotig15399	6.437	6.613	6.363	8.33	4396	0.358194935	isogroup02907	ENSG00000055208	ENST00000538427	TAB2
isotig15400	6.571	6.812	6.498	8.508	4309	0.370378372	isogroup02907	ENSG00000055208	ENST00000538427	TAB2
isotig15401	4.656	4.927	3.927	3.483	4422	0.017143233	isogroup02908	ENSG00000143437	ENST00000358595	ARNT
isotig15402	4.452	4.607	3.836	3.396	4311	0.042524611	isogroup02908	ENSG00000143437	ENST00000358595	ARNT
isotig15403	2.755	3.494	2.301	2.214	4454	0.19021004	isogroup02909	ENSG00000171988	ENST00000542921	JMJD1C
isotig15404	2.889	3.728	2.401	2.356	4261	0.22524799	isogroup02909	ENSG00000171988	ENST00000542921	JMJD1C
isotig15405	4.785	4.301	4.424	4.533	4368	0.042524611	isogroup02910	ENSG00000137073	ENST00000449054	UBAP2

isotig15406	4.877	4.411	4.552	4.618	4302	0.042524611	isogroup02910	ENSG00000137073	ENST00000449054	UBAP2
isotig15407	3.521	3.34	2.929	2.348	4753	0.181304952	isogroup02911	ENSG00000112701	ENST00000447266	SENP6
isotig15408	3.264	3.043	3.046	2.147	3916	0.282849252	isogroup02911	ENSG00000112701	ENST00000447266	SENP6
isotig15409	6.897	3.955	3.514	3.584	6640	0.505730067	isogroup02912	ENSG00000134982	ENST00000508376	APC
isotig15411	6.388	6.676	4.605	5.353	5798	0.188772826	isogroup02913	ENSG00000104885	ENST00000398665	DOT1L
isotig15412	2.952	2.948	2.575	2.268	2818	0.125807845	isogroup02913	ENSG00000079432	ENST00000160740	CIC
isotig15413	6.438	2.681	3.815	2.001	4324	0.77215939	isogroup02914	ENSG00000184916	ENST00000331782	JAG2
isotig15414	6.208	2.635	3.768	1.944	4290	0.76816713	isogroup02914	ENSG00000184916	ENST00000331782	JAG2
isotig15415	14.774	13.558	12.187	12.764	4369	0.036531525	isogroup02915	ENSG00000101558	ENST00000340541	VAPA
isotig15416	15.158	14.408	12.505	13.515	4234	0.053580822	isogroup02915	ENSG00000101558	ENST00000340541	VAPA
isotig15417	5.006	9.124	3.945	4.558	4332	0.62230405	isogroup02916	ENSG00000138757	ENST00000395719	G3BP2
isotig15418	4.982	8.834	3.911	4.495	4233	0.607903735	isogroup02916	ENSG00000138757	ENST00000395719	G3BP2
isotig15419	3.18	2.77	2.058	1.927	4346	0.010060494	isogroup02917	ENSG00000144283	ENST00000389759	PKP4
isotig15420	3.064	2.733	1.972	1.858	4217	0.050227324	isogroup02917	ENSG00000144283	ENST00000389757	PKP4
isotig15421	6.316	8.977	8.564	67.666	4309	0.94764034	isogroup02918	ENSG00000124449	ENST00000244314	IRGC
isotig15422	6.322	9.475	8.966	71.917	4193	0.951595025	isogroup02918	ENSG00000124449	ENST00000244314	IRGC
isotig15423	5.921	3.756	4.794	3.893	4221	0.523305403	isogroup02919	ENSG00000137414	ENST00000259963	FAM8A1
isotig15424	5.466	3.595	4.388	3.589	4204	0.486097543	isogroup02919	ENSG00000137414	ENST00000259963	FAM8A1
isotig15425	7.37	4.509	6.4	4.915	4489	0.547005336	isogroup02920	ENSG00000146830	ENST00000275732	GIGYF1
isotig15426	6.432	4.526	3.509	4.015	3920	0.239394679	isogroup02920	ENSG00000106771	ENST00000374586	C9orf5
isotig15427	6.717	3.921	5.204	4.925	7529	0.479287217	isogroup02921	ENSG00000148396	ENST00000313050	SEC16A
isotig15428	4.799	2.93	4.153	3.222	747	0.524545352	isogroup02921	ENSG00000148396	ENST00000537660	SEC16A
isotig15429	2.743	2.055	2.579	2.258	4932	0.313040129	isogroup02922	ENSG00000167447	ENST00000300917	SMG8
isotig15430	1.766	1.345	1.647	1.521	3319	0.170981438	isogroup02922	ENSG00000167447	ENST00000300917	SMG8
isotig15431	6.72	6.04	7.914	4.82	4700	0.550828511	isogroup02923	ENSG00000166387	ENST00000530181	PPFIBP2
isotig15432	6.33	5.872	7.953	4.987	3551	0.519003156	isogroup02923	ENSG00000166387	ENST00000299492	PPFIBP2
isotig15433	8.266	5.247	7.706	5.782	4257	0.595570001	isogroup02924	ENSG00000145191	ENST00000444495	EIF2B5
isotig15434	8.906	5.756	8.409	6.389	3958	0.594480349	isogroup02924	ENSG00000145191	ENST00000273783	EIF2B5
isotig15435	5.115	4.118	3.102	2.104	4262	0.458189299	isogroup02925	ENSG00000112851	ENST00000284037	ERBB2IP
isotig15436	4.376	3.902	2.724	1.87	3932	0.364065905	isogroup02925	ENSG00000112851	ENST00000508515	ERBB2IP
isotig15437	15.614	13.767	9.413	8.954	4089	0.068657474	isogroup02926	ENSG00000099250	ENST00000374867	NRP1
isotig15439	3.048	2.654	3.142	2.665	4128	0.185889006	isogroup02927	ENSG00000198700	ENST00000361565	IPO9
isotig15440	3.087	2.745	3.237	2.789	4018	0.181304952	isogroup02927	ENSG00000198700	ENST00000361565	IPO9
isotig15443	7.766	9.202	6.048	6.344	4134	0.238596228	isogroup02929	ENSG00000135241	ENST00000436062	PNPLA8
isotig15444	7.721	9.222	6.083	6.419	3995	0.252160517	isogroup02929	ENSG00000135241	ENST00000436062	PNPLA8
isotig15445	4.791	2.809	4.114	3.285	5244	0.498403096	isogroup02930	ENSG00000068650	ENST00000487903	ATP11A
isotig15446	2.793	1.647	2.279	1.887	2889	0.403706696	isogroup02930	ENSG00000068650	ENST00000487903	ATP11A
isotig15447	7.652	6.484	7.162	8.121	4117	0.020365221	isogroup02931	ENSG00000074964	ENST00000361221	ARHGEF10L
isotig15448	7.756	6.679	7.372	8.398	4000	0.048282859	isogroup02931	ENSG00000074964	ENST00000361221	ARHGEF10L
isotig15449	2.544	2.545	2.116	2.629	4054	0.143514692	isogroup02932	ENSG00000136643	ENST00000366960	RPS6KC1
isotig15450	2.449	2.421	2.036	2.532	4049	0.133181784	isogroup02932	ENSG00000136643	ENST00000366959	RPS6KC1
isotig15451	3.204	8.519	5.479	3.913	4051	0.649573533	isogroup02933	ENSG00000073910	ENST00000380250	FRY
isotig15453	7.691	6.298	9.006	9.38	4086	0.147769971	isogroup02934	ENSG00000100280	ENST00000356015	AP1B1
isotig15454	7.414	6.122	8.815	9.163	3980	0.146032164	isogroup02934	ENSG00000100280	ENST00000432560	AP1B1
isotig15455	20.794	19.313	28.161	33.881	4236	0.620631998	isogroup02935	ENSG00000168334	ENST00000340369	XIRP1
isotig15456	22.414	20.805	30.966	37.132	3810	0.630232209	isogroup02935	ENSG00000168334	ENST00000340369	XIRP1
isotig15457	7.594	11.311	7.985	7.284	5131	0.306342526	isogroup02936	ENSG00000047346	ENST00000546305	FAM214A
isotig15458	7.356	8.815	7.118	5.33	2913	0.170042083	isogroup02936	ENSG00000047346	ENST00000546305	FAM214A
isotig15459	2.573	2.629	2.187	2.465	4619	0.17765086	isogroup02937	ENSG00000165156	ENST00000522655	ZHX1
isotig15460	2.443	2.418	1.899	2.464	3393	0.243631172	isogroup02937	ENSG00000165156	ENST00000522655	ZHX1
isotig15461	3.673	3.124	3.373	2.513	4347	0.249755768	isogroup02938	ENSG00000134186	ENST00000370025	PRPF38B
isotig15462	3.095	2.406	3.059	1.95	3604	0.3747088	isogroup02938	ENSG00000134186	ENST00000370025	PRPF38B
isotig15463	6.98	9.498	11.536	11.656	3991	0.31465582	isogroup02939	ENSG00000078804	ENST00000374809	TP53INP2
isotig15464	6.933	9.413	11.475	11.529	3932	0.314937627	isogroup02939	ENSG00000078804	ENST00000374809	TP53INP2
isotig15465	22.484	14.198	12.628	10.284	4523	0.622905238	isogroup02940	ENSG00000121316	ENST00000240617	PLBD1
isotig15466	6.666	2.698	3.293	2.309	3290	0.727756068	isogroup02940	ENSG00000121316	ENST00000240617	PLBD1
isotig15467	2.343	3.172	4.179	3.323	3972	0.072142481	isogroup02941	ENSG00000011376	ENST00000415258	LARS2
isotig15468	2.134	2.989	3.946	3.143	3828	0.082137221	isogroup02941	ENSG00000011376	ENST00000415258	LARS2
isotig15469	16.732	36.756	19.032	17.171	4067	0.810832644	isogroup02942	ENSG00000142156	ENST00000361866	COL6A1
isotig15470	14.373	30.765	15.688	14.526	3698	0.815510634	isogroup02942	ENSG00000142156	ENST00000361866	COL6A1
isotig15471	3.93	4.323	5.288	3.098	5391	0.35553656	isogroup02943	ENSG00000174233	ENST00000307885	ADCY6
isotig15472	2.42	2.44	3.92	1.745	2360	0.43323063	isogroup02943	ENSG00000185567	ENST00000557457	AHNAK2
isotig15473	12.253	7.411	11.238	9.195	7258	0.595729691	isogroup02944	ENSG00000102921	ENST00000262384	N4BP1
isotig15474	6.486	5.32	5.619	3.708	465	0.463684527	isogroup02944	ENSG00000132139	ENST00000254466	GAS2L2
isotig15475	2.926	1.916	4.146	3.796	3877	0.315163072	isogroup02945	ENSG00000135709	ENST00000566428	KIAA0513
isotig15476	2.949	1.881	4.2	3.788	3847	0.339877508	isogroup02945	ENSG00000135709	ENST00000538274	KIAA0513
isotig15477	17.038	15.747	14.679	12.826	4780	0.230837153	isogroup02946	ENSG00000170606	ENST00000304858	HSPA4
isotig15478	5.472	6.017	5.279	4.472	2931	0.007420906	isogroup02946	ENSG00000170606	ENST00000304858	HSPA4
isotig15479	8.273	8.406	10.281	9.33	4576	0.107715864	isogroup02947	ENSG00000185963	ENST00000375512	BICD2
isotig15480	6.334	4.718	8.863	7.677	3123	0.428449312	isogroup02947	ENSG00000185963	ENST00000375512	BICD2
isotig15481	5.638	4.48	4.572	4.408	4134	0.283816788	isogroup02948	ENSG00000181555	ENST00000451092	SETD2
isotig15482	3.986	3.115	2.963	3.174	3562	0.150146539	isogroup02948	ENSG00000181555	ENST00000451092	SETD2
isotig15483	10.518	13.841	7.833	8.984	3845	0.434517547	isogroup02949	ENSG00000166200	ENST00000299259	COPS2
isotig15487	6.354	4.727	4.296	3.441	5950	0.457729015	isogroup02951	ENSG00000123066	ENST00000281928	MED13L
isotig15489	13.629	9.36	14.404	11.089	3872	0.55894454	isogroup02952	ENSG00000090565	ENST00000457159	RAB11FIP3
isotig15490	12.933	8.898	13.709	10.489	3737	0.563660104	isogroup02952	ENSG00000090565	ENST00000262305	RAB11FIP3
isotig15491	5.981	4.354	5.055	4.2	4621	0.399827159	isogroup02953	ENSG00000157350	ENST00000393640	ST3GAL2
isotig15492	4.073	2.711	3.161	2.635	2973	0.403640941	isogroup02953	ENSG00000157350	ENST00000393640	ST3GAL2
isotig15493	4.47	2.768	3.991	3.152	4854	0.502348388	isogroup02954	ENSG00000120451	ENST00000265909	SNX19
isotig15494	1.768	1.188	1.517	1.369	2739	0.212848501	isogroup02954	ENSG00000120451	ENST00000265909	SNX19
isotig15495	8.188	4.981	13.743	9.037	4188	0.787358157	isogroup02955	ENSG00000139641	ENST00000541590	ESYT1
isotig15496	6.202	3.707	9.864	6.883	3394	0.745763508	isogroup02955	ENSG00000139641	ENST00000394048	ESYT1

isotig15497	12.602	12.009	15.627	12.007	3797	0.09897986	isogroup02956	ENSG00000108946	ENST00000536854	PRKAR1A
isotig15498	12.995	12.383	15.881	12.431	3763	0.112638085	isogroup02956	ENSG00000108946	ENST00000536854	PRKAR1A
isotig15499	10.18	15.394	11.972	10.115	5129	0.397225145	isogroup02957	ENSG00000143248	ENST00000313961	RGS5
isotig15501	2.585	2.534	3.604	4.783	3789	0.198138198	isogroup02958	ENSG00000142459	ENST00000538904	EVI5L
isotig15502	2.48	2.455	3.498	4.568	3756	0.196062223	isogroup02958	ENSG00000142459	ENST00000538904	EVI5L
isotig15503	2.687	2.776	2.897	3.023	3843	0.038353874	isogroup02959	ENSG00000135974	ENST00000258457	C2orf49
isotig15504	2.577	2.716	2.812	2.78	3697	0.014428496	isogroup02959	ENSG00000135974	ENST00000437250	C2orf49
isotig15505	3.977	3.624	4.29	4.022	3957	0.124520929	isogroup02960	ENSG00000100109	ENST00000407148	TFIP11
isotig15506	3.987	3.585	4.21	3.925	3576	0.158647704	isogroup02960	ENSG00000100109	ENST00000407148	TFIP11
isotig15507	37.124	32.418	28.368	26.276	3756	0.1950759	isogroup02961	ENSG00000142864	ENST00000370995	SERBP1
isotig15508	37.762	32.498	28.794	26.45	3711	0.210246487	isogroup02961	ENSG00000142864	ENST00000361219	SERBP1
isotig15509	1.51	1.507	1.571	1.243	3802	0.086430074	isogroup02962	ENSG00000119285	ENST00000366582	HEATR1
isotig15510	1.342	1.359	1.326	1.106	3684	0.061922297	isogroup02962	ENSG00000119285	ENST00000366582	HEATR1
isotig15511	1.776	2.808	2.254	2.365	3796	0.312213497	isogroup02963	ENSG00000163785	ENST00000296084	RYK
isotig15512	1.965	3.069	2.444	2.583	3684	0.335302848	isogroup02963	ENSG00000163785	ENST00000460933	RYK
isotig15513	5.423	4.589	4.829	3.957	4594	0.156008116	isogroup02964	ENSG00000085788	ENST00000520272	DDHD2
isotig15514	3.151	2.452	2.578	2.194	2868	0.180356204	isogroup02964	ENSG00000085788	ENST00000520272	DDHD2
isotig15515	6.469	19.772	12.126	15.526	4348	0.843052529	isogroup02965	ENSG00000164776	ENST00000537360	PHKG1
isotig15516	5.929	19.985	12.472	14.549	3092	0.839454798	isogroup02965	ENSG00000164776	ENST00000537360	PHKG1
isotig15517	10.276	6.948	10.055	9.291	3783	0.335960397	isogroup02966	ENSG00000155506	ENST00000336314	LARP1
isotig15518	9.664	6.638	9.707	8.78	3576	0.334241377	isogroup02966	ENSG00000155506	ENST00000336314	LARP1
isotig15519	4.392	2.433	3.558	3.173	3708	0.522225145	isogroup02967	ENSG00000105726	ENST00000357324	ATP13A1
isotig15520	4.422	2.381	3.576	3.135	3638	0.540580146	isogroup02967	ENSG00000105726	ENST00000357324	ATP13A1
isotig15521	2.125	1.505	1.285	1.244	3680	0.1098482	isogroup02968	ENSG00000150760	ENST00000280333	DOCK1
isotig15522	2.14	1.521	1.292	1.266	3617	0.10352634	isogroup02968	ENSG00000150760	ENST00000280333	DOCK1
isotig15523	1.977	2.952	2.63	2.448	3717	0.299081311	isogroup02969	ENSG00000168763	ENST00000305510	CNNM3
isotig15524	1.746	2.469	2.311	2.03	3512	0.193197189	isogroup02969	ENSG00000168763	ENST00000305510	CNNM3
isotig15525	6.084	5.741	6.932	6.529	3657	0.059639663	isogroup02970	ENSG00000130340	ENST00000539592	SNX9
isotig15526	6.141	5.854	6.94	6.492	3564	0.048282859	isogroup02970	ENSG00000130340	ENST00000539592	SNX9
isotig15527	3.528	2.195	2.778	1.549	3699	0.486369956	isogroup02971	ENSG00000101384	ENST00000423891	JAG1
isotig15528	3.665	2.23	2.91	1.6	3512	0.502620801	isogroup02971	ENSG00000101384	ENST00000423891	JAG1
isotig15529	28.336	20.089	30.584	24.989	3617	0.527786128	isogroup02972	ENSG00000106263	ENST00000314800	EIF3B
isotig15530	28.215	19.911	30.251	24.833	3583	0.527541895	isogroup02972	ENSG00000106263	ENST00000314800	EIF3B
isotig15531	7.053	6.335	6.495	5.224	3619	0.231184715	isogroup02973	ENSG00000089597	ENST00000534779	GANAB
isotig15532	6.905	6.104	6.317	5.05	3575	0.24399752	isogroup02973	ENSG00000089597	ENST00000540933	GANAB
isotig15533	4.47	3.369	4.923	5.48	4130	0.067426918	isogroup02974	ENSG00000107021	ENST00000372648	TBC1D13
isotig15534	3.015	2.385	3.352	3.53	3057	0.06991621	isogroup02974	ENSG00000225614	ENST00000565624	ZNF469
isotig15535	3.652	3.127	3.493	3.852	4298	0.053402345	isogroup02975	ENSG00000113812	ENST00000335754	ACTR8
isotig15536	4.073	3.281	3.817	3.871	2883	0.167120688	isogroup02975	ENSG00000113812	ENST00000335754	ACTR8
isotig15537	1.068	1.269	1.29	1.579	3629	0.131678816	isogroup02976	ENSG00000137275	ENST00000380409	RIPK1
isotig15539	2.353	2.041	2.288	2.225	3717	0.045840535	isogroup02977	ENSG00000025293	ENST00000374012	PHF20
isotig15540	2.299	1.935	2.3	2.094	3448	0.112769595	isogroup02977	ENSG00000025293	ENST00000374012	PHF20
isotig15541	3.138	3.313	6.847	3.45	4280	0.432526114	isogroup02978	ENSG00000065534	ENST00000475616	MYLK
isotig15542	2.329	2.99	5.244	3	2877	0.268580446	isogroup02978	ENSG00000065534	ENST00000475616	MYLK
isotig15543	6.645	4.923	11.104	7.704	3590	0.603695423	isogroup02979	ENSG00000108292	ENST00000325718	MLLT6
isotig15544	5.9	4.443	9.853	6.941	3556	0.573044262	isogroup02979	ENSG00000108292	ENST00000325718	MLLT6
isotig15545	11.096	15.476	11.449	13.656	3723	0.420671451	isogroup02980	ENSG00000143384	ENST00000369026	MCL1
isotig15546	9.131	13.15	9.767	10.779	3400	0.413128429	isogroup02980	ENSG00000143384	ENST00000439749	MCL1
isotig15547	23.581	19.755	24.189	23.657	4450	0.237365672	isogroup02981	ENSG00000235307	ENST00000552587	BRD2
isotig15548	11.437	9.964	11.997	13.014	2662	0.169027579	isogroup02981	ENSG00000234507	ENST00000547895	BRD2
isotig15549	3.902	3.951	2.79	2.555	3938	0.079835801	isogroup02982	ENSG00000153914	ENST00000537482	SREK1
isotig15550	3.958	3.581	3.028	2.404	3171	0.239676486	isogroup02982	ENSG00000153914	ENST00000380918	SREK1
isotig15551	4.545	3.115	2.317	2.52	4842	0.266072368	isogroup02983	ENSG00000138709	ENST00000326639	LARP1B
isotig15552	3.394	2.363	1.3	1.73	2251	0.146821222	isogroup02983	ENSG00000138709	ENST00000427266	LARP1B
isotig15553	3.915	3.117	3.533	2.919	3862	0.322630946	isogroup02984	ENSG00000163945	ENST00000511216	KIAA1530
isotig15554	5.738	3.686	5.842	4.405	3230	0.520712783	isogroup02984	ENSG00000170260	ENST00000335870	ZNF212
isotig15555	13.527	8.203	16.505	13.384	3614	0.601328248	isogroup02985	ENSG00000114316	ENST00000265560	USP4
isotig15556	12.753	7.752	15.607	12.58	3473	0.598500789	isogroup02985	ENSG00000114316	ENST00000351842	USP4
isotig15557	7.482	9.594	6.324	3.429	3574	0.457447208	isogroup02986	ENSG00000154380	ENST00000366844	ENAH
isotig15558	7.372	9.311	6.279	3.319	3511	0.465375366	isogroup02986	ENSG00000154380	ENST00000538194	ENAH
isotig15559	7.377	3.697	4.184	2.808	3561	0.708001428	isogroup02987	ENSG00000134744	ENST00000371544	ZCCHC11
isotig15560	6.818	3.288	3.77	2.506	3472	0.709476216	isogroup02987	ENSG00000134744	ENST00000371544	ZCCHC11
isotig15561	10.811	12.966	11.559	7.532	3543	0.08237206	isogroup02988	ENSG00000261371	ENST00000563924	PECAM1
isotig15562	10.782	12.736	11.383	7.379	3486	0.067614789	isogroup02988	ENSG00000261371	ENST00000563924	PECAM1
isotig15563	15.361	9.058	11.548	10.527	3544	0.556304952	isogroup02989	ENSG00000165914	ENST00000357056	TTC7B
isotig15564	15.62	9.231	11.751	10.7	3493	0.557422785	isogroup02989	ENSG00000165914	ENST00000357056	TTC7B
isotig15565	3.198	2.773	4.57	3.188	3525	0.448964831	isogroup02990	ENSG00000141376	ENST00000390652	BCAS3
isotig15566	3.188	2.754	4.539	3.165	3480	0.441891486	isogroup02990	ENSG00000141376	ENST00000407086	BCAS3
isotig15567	4.298	12.068	4.819	5.431	4052	0.736924175	isogroup02991	ENSG00000173599	ENST00000393955	PC
isotig15568	2.584	6.928	2.622	3.457	2937	0.694023822	isogroup02991	ENSG00000173599	ENST00000393955	PC
isotig15569	2.443	1.868	2.945	2.103	3577	0.284821898	isogroup02992	ENSG00000154124	ENST00000284274	FAM105B
isotig15570	3.229	2.355	3.711	2.691	3411	0.327684677	isogroup02992	ENSG00000154124	ENST00000284274	FAM105B
isotig15571	2.476	5.572	4.776	5.676	3597	0.590131134	isogroup02993	ENSG00000167703	ENST00000301335	SLC43A2
isotig15572	2.349	4.845	4.456	5.215	3383	0.531383858	isogroup02993	ENSG00000167703	ENST00000301335	SLC43A2
isotig15573	7.203	4.325	7.821	7.147	3499	0.524902307	isogroup02994	ENSG00000172534	ENST00000354233	HCFC1
isotig15575	8.806	7.844	8.968	7.838	3556	0.190294582	isogroup02995	ENSG00000260150	ENST00000565311	ZMYND11.1
isotig15576	9.103	7.849	9.325	8.054	3394	0.244833546	isogroup02995	ENSG00000260150	ENST00000565311	ZMYND11.1
isotig15577	16.789	15.914	16.016	14.59	3483	0.084758022	isogroup02996	ENSG00000132824	ENST00000342374	SERINC3
isotig15579	7.391	21.834	7.98	10.64	3722	0.924231607	isogroup02997	ENSG00000142173	ENST00000409416	COL6A2
isotig15580	12.867	37.245	13.664	18.264	3223	0.937279252	isogroup02997	ENSG00000142173	ENST00000300527	COL6A2
isotig15582	1.544	1.456	1.155	4.488	3078	0.446165552	isogroup02998	ENSG00000119922	ENST00000371826	IFIT2
isotig15584	3.022	5.07	5.747	3.788	3298	0.053402345	isogroup02999	ENSG00000150995	ENST00000456211	ITPR1

isotig15585	3.141	2.244	2.48	2.426	3529	0.269331931	isogroup03000	ENSG00000139436	ENST00000355312	GIT2
isotig15586	3.147	2.356	2.519	2.511	3401	0.236736304	isogroup03000	ENSG00000139436	ENST00000355312	GIT2
isotig15587	6.044	3.639	4.757	3.642	3544	0.570254377	isogroup03001	ENSG00000120837	ENST00000551727	NFYB
isotig15588	6.241	3.697	4.786	3.753	3349	0.566938453	isogroup03001	ENSG00000120837	ENST00000551727	NFYB
isotig15589	6.361	5.007	5.351	5.169	5069	0.224289847	isogroup03002	ENSG00000109685	ENST00000508803	WHSC1
isotig15590	4.049	2.935	2.646	2.815	1793	0.227652739	isogroup03002	ENSG00000109685	ENST00000514045	WHSC1
isotig15591	2.661	2.069	2.4	1.789	3486	0.319455925	isogroup03003	ENSG00000057663	ENST00000369076	ATG5
isotig15592	2.447	1.837	2.222	1.703	3358	0.316515744	isogroup03003	ENSG00000057663	ENST00000369070	ATG5
isotig15593	1.628	2.675	1.635	1.431	3857	0.307281882	isogroup03004	ENSG00000196455	ENST00000356763	PIK3R4
isotig15595	35.096	27.648	45.237	26.201	3425	0.682056436	isogroup03005	ENSG00000115762	ENST00000409279	PLEKHB2
isotig15596	36.026	28.686	46.195	26.754	3381	0.675574885	isogroup03005	ENSG00000115762	ENST00000409279	PLEKHB2
isotig15597	2.505	2.152	1.771	2.037	3560	0.045840535	isogroup03006	ENSG00000102763	ENST00000379310	KIAA0564
isotig15598	2.441	1.966	1.54	1.911	3268	0.028237018	isogroup03006	ENSG00000102763	ENST00000379310	KIAA0564
isotig15599	4.261	5.251	4.403	5.254	4502	0.351920042	isogroup03007	ENSG00000141867	ENST00000371835	BRD4
isotig15600	4.86	4.791	4.429	5.158	2324	0.168586083	isogroup03007	ENSG00000141867	ENST00000263377	BRD4
isotig15601	9.617	7.375	9.96	8.441	3412	0.371477418	isogroup03008	ENSG00000112640	ENST00000485511	PPP2R5D
isotig15602	6.2	5.212	6.429	5.114	3382	0.323288495	isogroup03008	ENSG00000112640	ENST00000485511	PPP2R5D
isotig15603	26.3	15.109	25.262	18.833	3613	0.726769745	isogroup03009	ENSG00000134107	ENST00000256495	BHLHE40
isotig15604	31.042	17.469	29.737	21.734	3173	0.748036748	isogroup03009	ENSG00000134107	ENST00000256495	BHLHE40
isotig15605	4.203	5.949	2.429	3.678	3500	0.504828286	isogroup03010	ENSG00000196323	ENST00000527478	ZBTB44
isotig15606	3.842	5.664	2.357	3.505	3283	0.525719546	isogroup03010	ENSG00000196323	ENST00000525842	ZBTB44
isotig15607	8.21	6.509	7.825	8.492	6270	0.105160818	isogroup03011	ENSG00000109111	ENST00000314616	SUPT6H
isotig15609	6.528	5.72	7.058	9.191	4399	0.130194634	isogroup03012	ENSG00000096070	ENST00000357641	BRPF3
isotig15610	4.16	3.104	3.748	5.136	2367	0.11341775	isogroup03012	ENSG00000096070	ENST00000543502	BRPF3
isotig15611	8.61	8.265	7.43	6.513	4379	0.160244608	isogroup03013	ENSG00000135387	ENST00000532820	CAPRIN1
isotig15612	8.711	7.459	6.248	5.733	2382	0.236811453	isogroup03013	ENSG00000135387	ENST00000530820	CAPRIN1
isotig15613	3.965	3.724	3.883	3.461	3483	0.133745397	isogroup03014	ENSG00000168488	ENST00000336783	ATXN2L
isotig15614	4.061	3.632	4.143	3.555	3278	0.204507026	isogroup03014	ENSG00000168488	ENST00000340394	ATXN2L
isotig15615	11.123	10.13	13.862	14.172	3510	0.110890884	isogroup03015	ENSG00000129347	ENST00000361821	KRI1
isotig15616	9.42	8.978	12.154	12.609	3233	0.037038777	isogroup03015	ENSG00000129347	ENST00000361821	KRI1
isotig15617	4.343	3.855	3.571	3.596	5463	0.074284211	isogroup03016	ENSG00000108061	ENST00000369452	SHOC2
isotig15618	2.744	2.577	1.674	2.137	1252	0.16059217	isogroup03016	ENSG00000108061	ENST00000369452	SHOC2
isotig15619	3.606	2.805	2.633	2.782	3464	0.201200496	isogroup03017	ENSG00000127870	ENST00000381588	RNF6
isotig15620	3.545	2.642	2.6	2.756	3253	0.232884948	isogroup03017	ENSG00000127870	ENST00000381588	RNF6
isotig15621	2.954	2.996	3.301	2.931	4212	0.099938003	isogroup03018	ENSG00000119321	ENST00000446284	FKBP15
isotig15622	1.445	1.442	1.395	1.373	2502	0.010060494	isogroup03018	ENSG00000119321	ENST00000446284	FKBP15
isotig15623	4.962	4.472	4.675	4.648	3369	0.144688886	isogroup03019	ENSG00000103194	ENST00000570191	USP10
isotig15624	5.051	4.543	4.797	4.76	3337	0.145693996	isogroup03019	ENSG00000103194	ENST00000219473	USP10
isotig15625	16.06	10.023	13.891	11.694	4762	0.585500113	isogroup03020	ENSG00000133985	ENST00000256367	TTC9
isotig15626	6.831	4.938	6.046	6.352	1912	0.312467123	isogroup03020	ENSG00000062822	ENST00000440232	POLD1
isotig15627	7.077	3.962	3.624	3.172	3536	0.54259976	isogroup03021	ENSG00000143458	ENST00000446567	GABPB2
isotig15628	7.989	4.321	3.944	3.48	3081	0.581442098	isogroup03021	ENSG00000213190	ENST00000368921	MLLT11
isotig15629	3.729	3.506	3.966	3.34	3331	0.187927407	isogroup03022	ENSG00000108883	ENST00000426333	EFTUD2
isotig15630	3.537	3.345	3.71	3.217	3292	0.161428196	isogroup03022	ENSG00000108883	ENST00000426333	EFTUD2
isotig15631	6.893	7.275	4.215	4.893	3349	0.188772826	isogroup03023	ENSG00000147852	ENST00000382100	VLDLR
isotig15632	7.236	7.451	4.527	5.094	3265	0.13943789	isogroup03023	ENSG00000147852	ENST00000382099	VLDLR
isotig15633	15.824	10.761	16.18	10.999	3390	0.632993913	isogroup03024	ENSG00000079459	ENST00000220584	FDFT1
isotig15634	12.702	8.35	12.882	8.88	3213	0.628522582	isogroup03024	ENSG00000079459	ENST00000220584	FDFT1
isotig15635	4.877	4.491	5.668	6.097	3378	0.146642744	isogroup03025	ENSG00000173295	ENST00000310542	AC068020.1
isotig15636	5.535	5.454	5.975	7.362	3211	0.034305253	isogroup03025	ENSG00000173295	ENST00000310542	AC068020.1
isotig15637	7.118	6.992	5.278	4.501	3663	0.033563162	isogroup03026	ENSG00000144815	ENST00000491511	FAM55C
isotig15638	5.69	5.855	4.699	3.722	2908	0.045878109	isogroup03026	ENSG00000144815	ENST00000491511	FAM55C
isotig15639	9.872	5.441	5.935	6.724	4135	0.442229654	isogroup03027	ENSG00000100139	ENST00000215957	MICALL1
isotig15640	9.098	4.926	5.366	5.645	2419	0.488135943	isogroup03027	ENSG00000100139	ENST00000402631	MICALL1
isotig15641	12.475	12.683	18.537	12.848	3830	0.43634929	isogroup03028	ENSG00000005893	ENST00000371335	LAMP2
isotig15642	10.653	6.639	10.104	6.598	2721	0.707860525	isogroup03028	ENSG00000005893	ENST00000200639	LAMP2
isotig15643	4.902	6.995	7.152	6.862	3675	0.233335838	isogroup03029	ENSG00000100304	ENST00000216129	TTLL12
isotig15644	4.021	6.168	4.031	5.366	2856	0.515424213	isogroup03029	ENSG00000100304	ENST00000216129	TTLL12
isotig15645	8.277	5.507	7.645	6.645	3332	0.488004434	isogroup03030	ENSG00000116750	ENST00000367450	UCHL5
isotig15646	6.774	4.615	6.08	5.456	3184	0.424597956	isogroup03030	ENSG00000116750	ENST00000367450	UCHL5
isotig15647	9.444	7.923	9.305	7.125	3302	0.402710979	isogroup03031	ENSG00000113851	ENST00000231948	CRBN
isotig15648	9.907	8.24	9.816	7.474	3196	0.425631247	isogroup03031	ENSG00000113851	ENST00000231948	CRBN
isotig15649	3.959	2.796	4.172	3.005	3305	0.450646276	isogroup03032	ENSG00000129515	ENST00000355110	SNX6
isotig15650	3.995	2.83	4.29	2.984	3171	0.459551364	isogroup03032	ENSG00000129515	ENST00000362031	SNX6
isotig15651	34.284	16.553	19.007	20.209	3265	0.630297963	isogroup03033	ENSG00000135636	ENST00000410041	DYSF
isotig15652	36.397	17.588	20.1	21.286	3206	0.631697603	isogroup03033	ENSG00000135636	ENST00000429174	DYSF
isotig15653	9.558	7.857	8.507	7.818	3296	0.306915533	isogroup03034	ENSG00000122550	ENST00000545443	KLHL7
isotig15654	9.38	7.851	8.439	7.618	3165	0.294130909	isogroup03034	ENSG00000122550	ENST00000339077	KLHL7
isotig15655	9.504	7.903	10.466	9.826	5247	0.220015781	isogroup03035	ENSG00000092377	ENST00000383032	TBL1Y
isotig15656	5.287	3.725	5.227	5.212	1224	0.277927031	isogroup03035	ENSG00000092377	ENST00000383032	TBL1Y
isotig15657	1.247	1.647	1.407	1.099	3260	0.172390471	isogroup03036	ENSG00000153071	ENST00000320816	DAB2
isotig15658	1.447	2.067	1.338	1.41	3223	0.365615841	isogroup03036	ENSG00000153071	ENST00000320816	DAB2
isotig15659	5.456	4.363	5.198	3.496	3721	0.457729015	isogroup03037	ENSG00000122299	ENST00000396516	ZC3H7A
isotig15660	3.099	2.486	2.529	1.857	2749	0.335302848	isogroup03037	ENSG00000122299	ENST00000396516	ZC3H7A
isotig15661	4.425	10.288	9.734	8.821	3369	0.614770422	isogroup03038	ENSG00000148339	ENST00000373064	SLC25A25
isotig15662	4.166	9.112	8.92	7.83	3075	0.556661907	isogroup03038	ENSG00000148339	ENST00000433501	SLC25A25
isotig15663	8.531	4.463	5.873	4.949	3248	0.638864883	isogroup03039	ENSG00000166199	ENST00000302708	ALKBH3
isotig15664	7.16	3.763	5.056	4.245	3159	0.624023071	isogroup03039	ENSG00000166199	ENST00000532129	ALKBH3
isotig15665	5.088	3.473	5.552	4.215	4724	0.510755617	isogroup03040	ENSG00000130714	ENST00000423007	POMT1
isotig15666	1.755	1.325	1.778	1.672	1667	0.164002029	isogroup03040	ENSG00000130714	ENST00000423007	POMT1
isotig15667	6.8	2.061	4.234	1.931	3652	0.832869918	isogroup03041	ENSG00000123836	ENST00000367080	PFKFB2
isotig15668	4.297	2.029	2.359	1.383	2735	0.633219358	isogroup03041	ENSG00000123836	ENST00000367080	PFKFB2

isotig15669	41.969	34.139	34.272	31.512	4665	0.356644999	isogroup03042	ENSG00000065150	ENST00000490680	IPO5
isotig15670	19.941	16.161	15.008	14.874	1709	0.315520027	isogroup03042	ENSG00000065150	ENST00000490680	IPO5
isotig15671	11.423	15.838	20.11	14.978	3508	0.020947622	isogroup03043	ENSG00000168906	ENST00000306434	MAT2A
isotig15672	12.004	16.285	21.379	16.429	2839	0.020947622	isogroup03043	ENSG00000168906	ENST00000306434	MAT2A
isotig15673	3.966	4.612	5.131	5.34	3190	0.118931765	isogroup03044	ENSG00000178234	ENST00000443352	GALNT11
isotig15674	3.956	4.732	5.139	5.368	3178	0.143448937	isogroup03044	ENSG00000178234	ENST00000443352	GALNT11
isotig15675	7.549	3.896	6.612	5.343	3901	0.625253626	isogroup03045	ENSG00000125447	ENST00000245541	GGA3
isotig15676	5.913	3.04	5.298	4.08	2464	0.633548133	isogroup03045	ENSG00000125447	ENST00000538886	GGA3
isotig15679	4.991	4.409	4.225	3.425	3745	0.295699632	isogroup03047	ENSG00000148343	ENST00000358369	FAM73B
isotig15680	3.374	3.023	3.297	2.342	2610	0.321475539	isogroup03047	ENSG00000148343	ENST00000358369	FAM73B
isotig15681	4.15	3.509	3.807	3.756	4106	0.219799729	isogroup03048	ENSG00000103932	ENST00000304330	RPAP1
isotig15682	2.232	1.786	1.927	2.139	2238	0.111369956	isogroup03048	ENSG00000103932	ENST00000561603	RPAP1
isotig15683	2.733	3.503	2.164	1.738	3529	0.258256932	isogroup03049	ENSG00000070214	ENST00000470972	SLC44A1
isotig15684	3.953	2.519	1.714	1.375	2784	0.374596077	isogroup03049	ENSG00000070214	ENST00000374724	SLC44A1
isotig15685	3.078	2.52	2.672	2.501	3410	0.074481476	isogroup03050	ENSG00000092847	ENST00000373204	EIF2C1
isotig15686	2.85	2.316	2.552	2.443	2905	0.064064026	isogroup03050	ENSG00000092847	ENST00000373204	EIF2C1
isotig15687	3.137	3.766	3.779	2.927	3174	0.025447133	isogroup03051	ENSG00000127527	ENST00000455140	EPS15L1
isotig15688	3.102	3.72	3.789	2.886	3126	0.040580146	isogroup03051	ENSG00000127527	ENST00000455140	EPS15L1
isotig15689	4.606	3.194	5.198	4.768	3334	0.388958819	isogroup03052	ENSG00000197724	ENST00000359246	PHF2
isotig15690	5.501	3.509	6.205	5.616	2964	0.459260164	isogroup03052	ENSG00000197724	ENST00000359246	PHF2
isotig15691	5.243	3.755	6.224	4.859	3308	0.481861051	isogroup03053	ENSG00000125875	ENST00000246077	TBC1D20
isotig15692	5.16	3.639	5.748	4.354	3005	0.499117006	isogroup03053	ENSG00000125875	ENST00000246077	TBC1D20
isotig15693	7.073	5.43	7.089	5.981	4080	0.405453896	isogroup03054	ENSG00000114650	ENST00000265565	SCAP
isotig15694	3.279	2.522	3.144	2.825	2211	0.287414519	isogroup03054	ENSG00000114650	ENST00000265565	SCAP
isotig15695	5.051	5.556	4.791	4.336	3191	0.0314684	isogroup03055	ENSG00000197530	ENST00000505820	MIB2
isotig15696	4.986	5.289	4.736	4.167	3084	0.014428496	isogroup03055	ENSG00000197530	ENST00000505820	MIB2
isotig15697	4.353	6.594	4.463	4.711	4177	0.421122342	isogroup03056	ENSG00000244754	ENST00000505213	N4BP2L2
isotig15698	2.8	5.045	3.105	3.61	2090	0.497069212	isogroup03056	ENSG00000244754	ENST00000267068	N4BP2L2
isotig15699	2.267	3.986	1.98	2.506	3171	0.517312317	isogroup03057	ENSG00000179152	ENST00000417237	C3orf23
isotig15700	2.181	3.911	1.945	2.481	3095	0.525381378	isogroup03057	ENSG00000179152	ENST00000417237	C3orf23
isotig15701	6.724	6.676	7.795	5.707	4103	0.296685955	isogroup03058	ENSG00000159131	ENST00000381831	GART
isotig15702	4.575	3.885	4.502	3.795	2141	0.287461486	isogroup03058	ENSG00000159131	ENST00000381831	GART
isotig15703	2.822	2.669	3.576	3.494	3723	0.089605095	isogroup03059	ENSG00000179134	ENST00000314471	SAMD4B
isotig15704	2.268	2.267	2.862	2.925	2510	0.035704892	isogroup03059	ENSG00000179134	ENST00000314471	SAMD4B
isotig15705	11.359	6.571	9.206	7.185	3133	0.681145262	isogroup03060	ENSG00000136986	ENST00000259512	DERL1
isotig15706	10.788	6.061	8.731	6.684	3073	0.688303149	isogroup03060	ENSG00000136986	ENST00000405944	DERL1
isotig15707	7.342	9.586	12.004	12.052	4144	0.276395882	isogroup03061	ENSG00000185104	ENST00000396153	FAF1
isotig15708	9.4	11.982	14.99	15.373	2062	0.284004659	isogroup03061	ENSG00000185104	ENST00000396153	FAF1
isotig15709	4.608	3.407	2.383	2.11	4956	0.362140227	isogroup03062	ENSG00000114857	ENST00000232978	NKTR
isotig15711	3.342	2.913	3.252	3.096	3185	0.133238145	isogroup03063	ENSG00000137522	ENST00000361756	RNF121
isotig15712	2.391	1.715	2.081	1.942	2991	0.267650485	isogroup03063	ENSG00000137522	ENST00000545854	RNF121
isotig15713	4.649	3.199	3.792	2.374	3258	0.566412415	isogroup03064	ENSG00000170271	ENST00000326080	C5orf4
isotig15714	4.575	3.254	3.939	2.404	2892	0.555919817	isogroup03064	ENSG00000170271	ENST00000326080	C5orf4
isotig15715	12.815	13.903	13.694	14.018	4581	0.172381078	isogroup03065	ENSG00000065000	ENST00000345016	AP3D1
isotig15716	8.992	9.031	9.8	8.724	1528	0.120960773	isogroup03065	ENSG00000065000	ENST00000356926	AP3D1
isotig15717	3.262	2.374	3.997	2.871	3536	0.419891786	isogroup03066	ENSG00000148337	ENST00000393608	CIZ1
isotig15718	3.132	2.9	4.237	3.188	2560	0.236088149	isogroup03066	ENSG00000148337	ENST00000393608	CIZ1
isotig15719	11.878	13.817	15.031	9.291	3119	0.097927782	isogroup03067	ENSG00000082781	ENST00000296181	ITGB5
isotig15720	11.014	12.496	13.83	8.702	2952	0.111726911	isogroup03067	ENSG00000082781	ENST00000296181	ITGB5
isotig15721	1.957	3.002	3.55	3.607	3826	0.161343654	isogroup03068	ENSG00000017260	ENST00000533801	ATP2C1
isotig15722	1.504	2.328	2.703	3.008	2244	0.15977493	isogroup03068	ENSG00000017260	ENST00000533801	ATP2C1
isotig15723	11.33	8.089	12.882	10.628	3066	0.467122567	isogroup03069	ENSG00000162909	ENST00000295006	CAPN2
isotig15724	10.441	7.45	11.76	9.844	3002	0.443150222	isogroup03069	ENSG00000162909	ENST00000433674	CAPN2
isotig15725	4.967	4.804	7.773	6.586	3562	0.151574359	isogroup03070	ENSG00000112294	ENST00000357578	ALDH5A1
isotig15726	2.916	3.228	5.373	4.379	2500	0.065830014	isogroup03070	ENSG00000112294	ENST00000546278	ALDH5A1
isotig15727	1.376	6.893	2.212	2.873	3794	0.854127527	isogroup03071	ENSG00000173706	ENST00000311127	HEG1
isotig15729	8.174	5.472	6.713	6.605	4955	0.33136695	isogroup03072	ENSG00000156931	ENST00000437079	VPS8
isotig15730	4.504	3.729	4.53	3.869	1082	0.241151274	isogroup03072	ENSG00000156931	ENST00000446204	VPS8
isotig15731	2.689	3.841	3.682	4.131	3157	0.375864207	isogroup03073	ENSG00000123146	ENST00000242786	CD97
isotig15732	2.665	3.777	3.654	4.025	2878	0.372867664	isogroup03073	ENSG00000123146	ENST00000358600	CD97
isotig15733	31.193	35.481	40.033	53.221	5426	0.434912076	isogroup03074	ENSG00000112782	ENST00000339561	CLIC5
isotig15734	9.23	10.924	12.197	15.407	595	0.394613737	isogroup03074	ENSG00000112782	ENST00000544153	CLIC5
isotig15735	4.993	6.802	6.568	8.558	3093	0.383491771	isogroup03075	ENSG00000121858	ENST00000241261	TNFSF10
isotig15736	4.432	5.918	5.727	7.2	2899	0.340948373	isogroup03075	ENSG00000121858	ENST00000241261	TNFSF10
isotig15737	4.237	4.114	2.731	3.37	4009	0.084044112	isogroup03076	ENSG00000133059	ENST00000367162	DSTYK
isotig15738	2.504	2.743	2.076	2.342	1974	0.126700233	isogroup03076	ENSG00000133059	ENST00000367162	DSTYK
isotig15739	4.195	2.498	5.369	2.326	3494	0.697123694	isogroup03077	ENSG00000203485	ENST00000330634	INF2
isotig15740	4.058	2.053	4.475	1.805	2480	0.735806343	isogroup03077	ENSG00000203485	ENST00000330634	INF2
isotig15741	4.258	3.124	3.195	2.753	3363	0.285282182	isogroup03078	ENSG00000176390	ENST00000324238	CRLF3
isotig15742	3.202	3.653	4.255	3.74	2595	0.051664537	isogroup03078	ENSG00000176390	ENST00000324238	CRLF3
isotig15743	3.829	2.585	4.564	3.355	3380	0.485205155	isogroup03079	ENSG00000175029	ENST00000309035	CTBP2
isotig15745	13.777	15.178	18.771	18.28	5486	0.084316525	isogroup03080	ENSG00000151148	ENST00000434735	UBE3B
isotig15746	3.069	4.429	2.777	4.968	462	0.623966709	isogroup03080	ENSG00000154175	ENST00000466947	ABI3BP
isotig15747	5.915	4.551	5.604	5.37	4588	0.406834749	isogroup03081	ENSG00000083799	ENST00000540145	CYLD
isotig15748	2.09	1.978	2.146	2.033	1338	0.135248366	isogroup03081	ENSG00000083799	ENST00000566206	CYLD
isotig15749	7.554	6.832	4.211	4.137	5306	0.458367776	isogroup03082	ENSG00000123384	ENST00000243077	LRP1
isotig15750	9.904	9.271	9.426	8.073	617	0.379161344	isogroup03082	ENSG00000123384	ENST00000243077	LRP1
isotig15751	2.746	2.883	2.228	1.771	2971	0.010060494	isogroup03083	ENSG00000132670	ENST00000425918	PTPRA
isotig15752	2.751	2.916	2.286	1.825	2944	0.003503795	isogroup03083	ENSG00000132670	ENST00000356147	PTPRA
isotig15753	1.783	3.367	1.462	2.043	3796	0.487976253	isogroup03084	ENSG00000196678	ENST00000357967	ERI2
isotig15754	2.731	3.78	2.407	2.032	2110	0.254048621	isogroup03084	ENSG00000196678	ENST00000569729	ERI2
isotig15755	14.2	18.94	19.451	15.724	3014	0.084401067	isogroup03085	ENSG00000183048	ENST00000333676	MRPL12

isotig15756	16.046	21.304	21.655	17.607	2885	0.100520403	isogroup03085	ENSG00000183048	ENST00000333676	MRPL12
isotig15757	2.538	3.447	3.205	2.852	3010	0.130814609	isogroup03086	ENSG00000176715	ENST00000406948	ACSF3
isotig15758	2.338	2.931	2.802	2.706	2883	0.102323965	isogroup03086	ENSG00000176715	ENST00000406948	ACSF3
isotig15759	8.125	6.037	5.955	5.875	4096	0.352483655	isogroup03087	ENSG00000078043	ENST00000398654	PIAS2
isotig15760	9.116	5.133	6.062	5.912	1791	0.53345044	isogroup03087	ENSG00000078043	ENST00000324794	PIAS2
isotig15761	3.02	3.278	2.276	1.883	2973	0.014428496	isogroup03088	ENSG00000075945	ENST00000361580	KIFAP3
isotig15762	3.05	3.251	2.241	1.896	2904	0.03231382	isogroup03088	ENSG00000075945	ENST00000361580	KIFAP3
isotig15765	16.731	34.04	14.771	16.764	4352	0.844348839	isogroup03090	ENSG00000142798	ENST00000374695	HSPG2
isotig15766	12.435	23.926	12.242	12.522	1500	0.766880213	isogroup03090	ENSG00000142798	ENST00000374695	HSPG2
isotig15767	8.21	5.708	8.422	7.782	2963	0.374464568	isogroup03091	ENSG00000144567	ENST00000430297	FAM134A
isotig15768	7.968	5.492	8.231	7.46	2875	0.393533479	isogroup03091	ENSG00000144567	ENST00000430297	FAM134A
isotig15769	2.242	5.161	2.551	3.848	3037	0.697255204	isogroup03092	ENSG00000172765	ENST00000393238	TMCC1
isotig15770	1.876	5.335	2.22	3.663	2799	0.76401518	isogroup03092	ENSG00000172765	ENST00000393238	TMCC1
isotig15771	3.238	3.837	2.389	2.4	3130	0.254612234	isogroup03093	ENSG00000198752	ENST00000361246	CDC42BPB
isotig15772	2.474	2.989	1.799	1.82	2700	0.253485008	isogroup03093	ENSG00000198752	ENST00000361246	CDC42BPB
isotig15773	4.942	2.56	4.638	2.753	2948	0.639550612	isogroup03094	ENSG00000157554	ENST00000288319	ERG
isotig15774	4.871	2.571	4.568	2.728	2876	0.628691666	isogroup03094	ENSG00000157554	ENST00000398905	ERG
isotig15775	11.048	6.511	8.585	5.843	4866	0.612403246	isogroup03095	ENSG00000162337	ENST00000294304	LRP5
isotig15776	2.513	2.484	1.539	1.83	954	0.098416247	isogroup03095	ENSG00000070018	ENST00000543091	LRP6
isotig15777	9.904	6.697	7.888	6.826	2960	0.487309311	isogroup03096	ENSG00000089737	ENST00000555054	DDX24
isotig15778	9.367	6.098	7.404	6.219	2842	0.525804088	isogroup03096	ENSG00000089737	ENST00000555054	DDX24
isotig15779	2.578	2.207	1.923	1.777	3773	0.162226648	isogroup03097	ENSG00000180376	ENST00000394672	CCDC66
isotig15781	14.707	10.272	11.232	10.68	3227	0.428825054	isogroup03098	ENSG00000130024	ENST00000339209	PHF10
isotig15782	11.122	6.37	8.443	7.059	2583	0.611811453	isogroup03098	ENSG00000185127	ENST00000439249	C6orf120
isotig15783	5.293	5.293	3.871	4.023	5133	0.107377696	isogroup03099	ENSG00000102580	ENST00000376795	DNAJC3
isotig15784	4.057	1.884	2.775	1.549	672	0.685297212	isogroup03099	ENSG00000102580	ENST00000376795	DNAJC3
isotig15785	13.333	16.406	16.875	13.91	3675	0.114516796	isogroup03100	ENSG00000203880	ENST00000308824	PCMTD2
isotig15786	10.601	12.906	12.815	10.83	2126	0.09651875	isogroup03100	ENSG00000203880	ENST00000308824	PCMTD2
isotig15787	4.374	4.361	3.718	3.561	2920	0.017143233	isogroup03101	ENSG00000121940	ENST00000415331	CLCC1
isotig15788	4.009	3.639	3.549	3.14	2877	0.141889607	isogroup03101	ENSG00000121940	ENST00000369969	CLCC1
isotig15789	9.42	4.742	3.605	3.398	3729	0.616508229	isogroup03102	ENSG00000156453	ENST00000394536	PCDH1
isotig15790	5.005	2.909	1.815	2.016	2055	0.39647366	isogroup03102	ENSG00000156453	ENST00000394536	PCDH1
isotig15791	1.344	2.149	1.886	3.136	2927	0.401254979	isogroup03103	ENSG00000090339	ENST00000264832	ICAM1
isotig15792	1.404	2.231	1.941	3.163	2845	0.403462463	isogroup03103	ENSG00000090339	ENST00000264832	ICAM1
isotig15793	1.48	1.628	1.818	1.77	2941	0.021652138	isogroup03104	ENSG00000132773	ENST00000372090	TOE1
isotig15794	1.486	1.562	1.858	1.774	2808	0.003503795	isogroup03104	ENSG00000132773	ENST00000372090	TOE1
isotig15795	3.76	5.613	3.102	3.187	2964	0.456667543	isogroup03105	ENSG00000241978	ENST00000510514	AKAP2
isotig15796	4.059	5.85	3.371	3.396	2785	0.422925904	isogroup03105	ENSG00000241978	ENST00000555236	AKAP2
isotig15797	1.271	4.101	3.654	5.296	2908	0.719790336	isogroup03106	ENSG00000136826	ENST00000374672	KLF4
isotig15798	1.379	4.34	3.836	5.603	2809	0.729775682	isogroup03106	ENSG00000136826	ENST00000374672	KLF4
isotig15799	2.784	1.867	6.775	5.396	4286	0.460049222	isogroup03107	ENSG00000131386	ENST00000339732	GALNTL2
isotig15802	3.577	2.448	2.846	2.223	2788	0.416547682	isogroup03108	ENSG00000111785	ENST00000392839	RIC8B
isotig15803	6.917	5.296	6.289	4.312	4140	0.53490644	isogroup03109	ENSG00000135317	ENST00000314673	SNX14
isotig15805	5.937	4.04	4.98	4.656	3991	0.33451379	isogroup03110	ENSG00000184708	ENST00000397525	EIF4ENIF1
isotig15806	3.103	2.054	2.036	1.953	1696	0.331376343	isogroup03110	ENSG00000184708	ENST00000420671	EIF4ENIF1
isotig15807	54.909	37.512	53.229	32.513	4153	0.765123619	isogroup03111	ENSG00000136261	ENST00000433922	BZW2
isotig15809	1.951	1.009	2.07	1.082	2877	0.48913166	isogroup03112	ENSG00000091127	ENST00000544995	PUS7
isotig15811	7.378	7.138	8.441	7.287	4760	0.130654918	isogroup03113	ENSG00000162852	ENST00000366513	CNST
isotig15812	3.395	2.953	3.433	3.042	909	0.176260615	isogroup03113	ENSG00000162852	ENST00000366512	CNST
isotig15813	32.325	41.063	36.72	20.751	2867	0.072612159	isogroup03114	ENSG00000152583	ENST00000418378	SPARCL1
isotig15814	31.145	39.401	35.497	20.282	2771	0.072414894	isogroup03114	ENSG00000152583	ENST00000418378	SPARCL1
isotig15815	4.531	4.614	4.729	4.445	3614	0.017143233	isogroup03115	ENSG00000135457	ENST00000257915	TFCP2
isotig15816	3.871	3.042	3.399	3.573	2002	0.131678816	isogroup03115	ENSG00000135457	ENST00000257915	TFCP2
isotig15817	10.122	6.679	10.855	9.765	2924	0.489216202	isogroup03116	ENSG00000115526	ENST00000542617	CHST10
isotig15818	10.767	7.205	11.618	10.634	2721	0.472824453	isogroup03116	ENSG00000115526	ENST00000542617	CHST10
isotig15820	2.272	2.991	2.173	1.707	2787	0.043172766	isogroup03117	ENSG00000085224	ENST00000395603	ATRX
isotig15821	26.4	18.508	23.866	16.227	4372	0.649845946	isogroup03118	ENSG00000164828	ENST00000456758	SUN1
isotig15822	10.632	7.113	7.76	6.381	1235	0.557695198	isogroup03118	ENSG00000164828	ENST00000456758	SUN1
isotig15823	7.979	6.858	9.574	9.338	2842	0.255833396	isogroup03119	ENSG00000100083	ENST00000343632	GGA1
isotig15824	7.892	7.324	10.195	10.006	2752	0.176579995	isogroup03119	ENSG00000100083	ENST00000343632	GGA1
isotig15825	19.971	17.346	26.107	15.397	4431	0.531684452	isogroup03120	ENSG00000163513	ENST00000295754	TGFBR2
isotig15826	7.401	4.968	8.501	5.567	1136	0.587895469	isogroup03120	ENSG00000163513	ENST00000295754	TGFBR2
isotig15827	2.667	2.91	2.958	2.613	3418	0.067041783	isogroup03121	ENSG00000155252	ENST00000370631	PI4K2A
isotig15828	2.241	2.596	2.527	2.372	2152	0.057310062	isogroup03121	ENSG00000260117	ENST00000570246	ZNF828.1
isotig15829	21.363	17.754	21.711	20.691	3472	0.279007289	isogroup03122	ENSG00000166938	ENST00000319212	DIS3L
isotig15830	12.423	10.581	13.94	12.208	2085	0.308268205	isogroup03122	ENSG00000166938	ENST00000319194	DIS3L
isotig15833	5.819	1.879	4.937	2.716	2852	0.810128128	isogroup03124	ENSG00000099957	ENST00000413302	P2RX6
isotig15834	5.793	1.906	5.127	2.636	2701	0.819934997	isogroup03124	ENSG00000099957	ENST00000443995	P2RX6
isotig15837	7.899	10.161	9.294	7.952	2852	0.133961449	isogroup03126	ENSG00000067082	ENST00000497571	KLF6
isotig15838	4.545	4.819	5.558	4.058	2676	0.156928684	isogroup03126	ENSG00000102554	ENST00000545883	KLF5
isotig15839	4.324	2.618	3.483	2.461	3279	0.508576313	isogroup03127	ENSG00000049769	ENST00000055335	PPP1R3F
isotig15840	3.046	1.955	2.582	1.856	2249	0.429914707	isogroup03127	ENSG00000049769	ENST00000055335	PPP1R3F
isotig15841	9.701	4.973	5.277	2.876	2915	0.794243631	isogroup03128	ENSG00000100429	ENST00000216271	HDAC10
isotig15842	8.31	4.805	4.75	2.685	2581	0.730808973	isogroup03128	ENSG00000100429	ENST00000216271	HDAC10
isotig15843	6.37	5.261	6.321	6.3	3488	0.240991583	isogroup03129	ENSG00000140564	ENST00000268171	FURIN
isotig15844	4.699	3.885	4.349	4.497	2004	0.210575261	isogroup03129	ENSG00000140564	ENST00000268171	FURIN
isotig15845	3.427	3.413	2.846	3.938	2777	0.243912978	isogroup03130	ENSG00000123908	ENST00000220592	EIF2C2
isotig15846	3.168	3.072	2.694	3.6	2708	0.196062223	isogroup03130	ENSG00000123908	ENST00000220592	EIF2C2
isotig15847	13.967	7.336	11.556	7.697	3115	0.765424213	isogroup03131	ENSG00000070476	ENST00000389709	ZXDC
isotig15848	9.479	5.156	8.246	5.228	2368	0.758764184	isogroup03131	ENSG00000070476	ENST00000389709	ZXDC
isotig15849	2.538	3.191	3.588	4.774	2774	0.312110168	isogroup03132	ENSG00000068971	ENST00000527441	PPP2R5B
isotig15850	2.458	3.101	3.572	4.812	2701	0.310287818	isogroup03132	ENSG00000068971	ENST00000527441	PPP2R5B

isotig15851	26.558	17.779	22.999	17.413	4384	0.58736943	isogroup03133	ENSG00000170348	ENST00000303575	TMED10
isotig15852	7.951	4.618	6.71	5.887	1080	0.544919967	isogroup03133	ENSG00000170348	ENST00000555873	TMED10
isotig15855	6.83	4.147	6.275	4.821	4411	0.580042459	isogroup03135	ENSG00000052749	ENST00000370992	RRP12
isotig15856	3.664	2.747	3.797	2.598	1060	0.475849177	isogroup03135	ENSG00000052749	ENST00000536831	RRP12
isotig15857	1.563	1.542	1.609	1.012	2932	0.116996693	isogroup03136	ENSG00000164330	ENST00000380654	EBF1
isotig15860	15.315	9.922	13.776	9.141	2731	0.614873751	isogroup03137	ENSG00000176435	ENST00000342213	CLEC14A
isotig15861	3.191	3.476	2.971	2.541	3154	0.035704892	isogroup03138	ENSG00000260383	ENST00000564535	HEATR7A.1
isotig15862	2.572	2.633	2.318	1.98	2306	0.03231382	isogroup03138	ENSG00000260383	ENST00000568089	HEATR7A.1
isotig15863	8.504	6.947	6.95	11.099	4758	0.515264522	isogroup03139	ENSG00000015133	ENST00000389857	CCDC88C
isotig15864	1.473	1.059	1.494	1.423	692	0.086430074	isogroup03139	ENSG00000015133	ENST00000556726	CCDC88C
isotig15865	4.189	2.836	4.469	3.158	2779	0.488596228	isogroup03140	ENSG00000068394	ENST00000156109	GPKOW
isotig15866	3.671	2.568	4.109	2.749	2654	0.477474262	isogroup03140	ENSG00000068394	ENST00000156109	GPKOW
isotig15867	4.279	4.423	3.896	3.778	2877	0.068676261	isogroup03141	ENSG00000117533	ENST00000474047	VAMP4
isotig15868	7.916	4.23	5.999	4.443	2567	0.611661156	isogroup03141	ENSG00000117533	ENST00000474047	VAMP4
isotig15869	4.392	2.908	3.038	2.632	2769	0.370866837	isogroup03142	ENSG00000167522	ENST00000378330	ANKRD11
isotig15870	4.335	2.928	2.982	2.585	2672	0.359989103	isogroup03142	ENSG00000167522	ENST00000378330	ANKRD11
isotig15871	30.37	17.682	26.225	25.46	3963	0.463553017	isogroup03143	ENSG00000119862	ENST00000238875	LGALSL
isotig15872	4.069	3.905	6.14	3.91	1460	0.435860825	isogroup03143	ENSG00000100605	ENST00000556603	ITPK1
isotig15873	6.678	6.672	3.695	3.531	2726	0.056474036	isogroup03144	ENSG00000161395	ENST00000378011	PGAP3
isotig15874	5.817	5.473	3.754	3.104	2683	0.153819418	isogroup03144	ENSG00000161395	ENST00000300658	PGAP3
isotig15875	1.653	1.482	1.142	1.218	2749	0.029392425	isogroup03145	ENSG00000106144	ENST00000310447	CASP2
isotig15876	1.549	1.43	1.108	1.094	2649	0.016560833	isogroup03145	ENSG00000106144	ENST00000310447	CASP2
isotig15877	5.687	5.444	6.111	6.078	2748	0.034305253	isogroup03146	ENSG00000104866	ENST00000221462	PPP1R37
isotig15878	5.309	5.078	5.869	5.646	2635	0.07293154	isogroup03146	ENSG00000104866	ENST00000221462	PPP1R37
isotig15879	1.503	2.019	1.647	2.404	2724	0.351938829	isogroup03147	ENSG00000133687	ENST00000551659	TMTC1
isotig15880	1.829	2.399	2.097	2.801	2642	0.339868115	isogroup03147	ENSG00000133687	ENST00000551659	TMTC1
isotig15881	18.927	21.284	22.84	34.556	2707	0.461833997	isogroup03148	ENSG00000189319	ENST00000392754	FAM53B
isotig15882	17.64	20.154	21.56	32.521	2652	0.470776659	isogroup03148	ENSG00000189319	ENST00000392754	FAM53B
isotig15883	6.701	5.125	8.437	6.592	2739	0.466831367	isogroup03149	ENSG00000099821	ENST00000215591	POLRMT
isotig15884	5.371	4.016	6.704	5.194	2614	0.471406027	isogroup03149	ENSG00000099821	ENST00000215591	POLRMT
isotig15885	1.218	2.165	2.781	2.62	2739	0.149488991	isogroup03150	ENSG00000135953	ENST00000258436	MFSD9
isotig15886	1.162	2.161	2.457	2.456	2612	0.203858871	isogroup03150	ENSG00000135953	ENST00000258436	MFSD9
isotig15887	4.282	4.443	5.206	5.071	3547	0.0314684	isogroup03151	ENSG00000118246	ENST00000403094	FASTKD2
isotig15888	2.988	3.044	3.455	3.536	1801	0.074284211	isogroup03151	ENSG00000118246	ENST00000403094	FASTKD2
isotig15889	8.357	7.832	8.429	6.197	2711	0.304576539	isogroup03152	ENSG00000164024	ENST00000296411	METAP1
isotig15890	8.322	7.729	8.314	5.959	2624	0.338468475	isogroup03152	ENSG00000164024	ENST00000296411	METAP1
isotig15891	13.742	7.034	11.917	7.23	3081	0.778800631	isogroup03153	ENSG00000170876	ENST00000306077	TMEM43
isotig15892	8.165	4.672	8.807	5.153	2255	0.719687007	isogroup03153	ENSG00000170876	ENST00000306077	TMEM43
isotig15893	4.93	3.503	4.997	3.375	2690	0.519801608	isogroup03154	ENSG00000053900	ENST00000510092	ANAPC4
isotig15894	5.216	3.664	5.18	3.557	2634	0.526790411	isogroup03154	ENSG00000053900	ENST00000315368	ANAPC4
isotig15895	3.433	3.521	3.875	3.883	3214	0.014428496	isogroup03155	ENSG00000164889	ENST00000485713	SLC4A2
isotig15896	2.488	2.12	2.431	2.546	2115	0.09882017	isogroup03155	ENSG00000164889	ENST00000485713	SLC4A2
isotig15897	19.256	9.621	17.602	16.586	2738	0.646567596	isogroup03156	ENSG00000048140	ENST00000310032	TSPAN17
isotig15898	19.67	10.298	18.336	17.618	2584	0.61185842	isogroup03156	ENSG00000048140	ENST00000310032	TSPAN17
isotig15899	2.77	2.513	2.108	1.825	2711	0.174006162	isogroup03157	ENSG00000111605	ENST00000266679	CPSF6
isotig15900	2.873	2.657	2.212	1.877	2600	0.16059217	isogroup03157	ENSG00000111605	ENST00000435070	CPSF6
isotig15901	9.222	5.293	9.713	8.345	2668	0.596011498	isogroup03158	ENSG00000133884	ENST00000252268	DPF2
isotig15902	8.966	5.103	9.44	7.941	2626	0.599205305	isogroup03158	ENSG00000133884	ENST00000528416	DPF2
isotig15903	2.407	1.938	3.118	2.016	2868	0.337989404	isogroup03159	ENSG00000115687	ENST00000539818	PASK
isotig15904	1.745	1.628	2.217	1.708	2432	0.190848801	isogroup03159	ENSG00000116560	ENST00000357214	SFPQ
isotig15905	14.752	46.843	17.245	21.623	3754	0.953736755	isogroup03160	ENSG00000163430	ENST00000295633	FSTL1
isotig15906	3.516	10.765	4.892	4.727	1525	0.837623056	isogroup03160	ENSG00000156313	ENST00000378505	RPGR
isotig15907	1.838	2.585	1.301	1.4	3037	0.267650485	isogroup03161	ENSG00000085274	ENST00000356716	MYNN
isotig15908	1.598	1.922	1.078	1.068	2243	0.09326858	isogroup03161	ENSG00000085274	ENST00000356716	MYNN
isotig15909	4.017	4.078	4.316	3.764	3393	0.056474036	isogroup03162	ENSG00000231377	ENST00000458094	DHX16
isotig15910	2.944	2.857	3.098	2.483	1889	0.110749981	isogroup03162	ENSG00000231377	ENST00000458094	DHX16
isotig15911	18.373	9.472	13.004	9.774	2974	0.728939656	isogroup03163	ENSG00000112146	ENST00000323557	FBXO9
isotig15912	18.3	10.119	13.384	10.549	2295	0.678458706	isogroup03163	ENSG00000112146	ENST00000323557	FBXO9
isotig15913	44.519	62.822	45.231	30.174	5149	0.056727662	isogroup03164	ENSG00000112992	ENST00000344920	NNT
isotig15914	19.128	13.559	17.825	15.358	2642	0.463318178	isogroup03165	ENSG00000072401	ENST00000373910	UBE2D1
isotig15915	17.97	12.77	17.025	14.401	2610	0.462115804	isogroup03165	ENSG00000072401	ENST00000373910	UBE2D1
isotig15916	8.611	7.441	8.874	6.541	2709	0.317943564	isogroup03166	ENSG00000135506	ENST00000257966	OS9
isotig15917	8.196	7.018	8.412	6.27	2544	0.349674983	isogroup03166	ENSG00000135506	ENST00000257966	OS9
isotig15918	6.796	5.66	7.721	5.161	2633	0.497980386	isogroup03167	ENSG00000100916	ENST00000216807	BRMS1L
isotig15920	6.727	4.802	5.513	4.724	3282	0.446250094	isogroup03168	ENSG00000149187	ENST00000532048	CELF1
isotig15921	5.524	4.006	4.054	3.786	1972	0.376756594	isogroup03168	ENSG00000149187	ENST00000532048	CELF1
isotig15922	5.544	7.515	6.415	5.72	2671	0.330850304	isogroup03169	ENSG00000157978	ENST00000374338	LDLRAP1
isotig15923	4.867	1.605	3.73	2.079	2579	0.763094612	isogroup03169	ENSG00000157978	ENST00000374338	LDLRAP1
isotig15926	15.363	7.454	14.98	9.063	4308	0.792853385	isogroup03171	ENSG00000025796	ENST00000369002	SEC63
isotig15927	26.836	15.661	40.242	29.264	931	0.642904111	isogroup03171	ENSG00000105357	ENST00000440075	MYH14
isotig15928	2.886	2.179	1.854	2.044	3674	0.119720824	isogroup03172	ENSG00000112282	ENST00000545957	MED23
isotig15930	5.529	11.092	8.438	11.445	2628	0.708386563	isogroup03173	ENSG00000105607	ENST00000222214	GCDH
isotig15931	5.47	11.312	8.132	11.228	2592	0.724364996	isogroup03173	ENSG00000105607	ENST00000222214	GCDH
isotig15932	11.015	20.326	9.193	14.82	2667	0.777410386	isogroup03174	ENSG00000167701	ENST00000528431	GPT
isotig15933	8.05	14.442	6.663	10.359	2549	0.744683249	isogroup03174	ENSG00000167701	ENST00000528431	GPT
isotig15934	13.092	11.921	10.788	9.185	3745	0.183399714	isogroup03175	ENSG00000044115	ENST00000302763	CTNNA1
isotig15935	6.671	6.076	6.092	4.857	1452	0.264785451	isogroup03175	ENSG00000044115	ENST00000537034	CTNNA1
isotig15936	12.775	7.866	10.368	8.452	3723	0.564439769	isogroup03176	ENSG00000134970	ENST00000456936	TMED7
isotig15937	14.216	8.147	9.937	6.278	1480	0.774958668	isogroup03176	ENSG00000124145	ENST00000372733	SDC4
isotig15938	1.927	1.281	1.851	1.7	2674	0.233457954	isogroup03177	ENSG00000136875	ENST00000374199	PRPF4
isotig15939	1.828	1.21	1.705	1.643	2534	0.188293755	isogroup03177	ENSG00000136875	ENST00000374199	PRPF4
isotig15940	9.802	11.694	11.473	7.42	2999	0.193338093	isogroup03178	ENSG00000131370	ENST00000383791	SH3BP5

isotig15941	8.031	9.703	9.527	6.185	2197	0.175819118	isogroup03178	ENSG00000131370	ENST00000383791	SH3BP5
isotig15942	7.077	3.186	7.343	4.15	2805	0.83337717	isogroup03179	ENSG00000175920	ENST00000340083	DOK7
isotig15943	4.963	2.293	5.579	3.107	2378	0.799588562	isogroup03179	ENSG00000175920	ENST00000389653	DOK7
isotig15944	3.952	4.482	4.351	4.855	2610	0.247379199	isogroup03180	ENSG00000075234	ENST00000381031	TTC38
isotig15945	3.756	4.224	4.252	4.528	2571	0.195883745	isogroup03180	ENSG00000075234	ENST00000381031	TTC38
isotig15948	7.105	8.557	5.766	5.861	3044	0.403199444	isogroup03182	ENSG00000087152	ENST00000454077	ATXN7L3
isotig15949	4.89	4.593	4.127	3.612	2132	0.067426918	isogroup03182	ENSG00000087152	ENST00000454077	ATXN7L3
isotig15950	4.448	2.671	3.763	2.459	3278	0.565848801	isogroup03183	ENSG00000187800	ENST00000338302	PEAR1
isotig15951	2.507	1.623	2.054	1.494	1888	0.404251522	isogroup03183	ENSG00000187800	ENST00000338302	PEAR1
isotig15954	4.708	4.141	5.2	5.076	2615	0.144688886	isogroup03185	ENSG00000127663	ENST00000536461	KDM4B
isotig15955	4.813	4.192	5.235	5.09	2529	0.167458856	isogroup03185	ENSG00000127663	ENST00000536461	KDM4B
isotig15956	2.396	2.389	2	1.322	2709	0.17921019	isogroup03186	ENSG00000159899	ENST00000342694	NPR2
isotig15957	2.526	2.634	2.044	1.425	2435	0.129649808	isogroup03186	ENSG00000159899	ENST00000342694	NPR2
isotig15958	38.769	28.121	22.794	17.027	4837	0.507486661	isogroup03187	ENSG00000047315	ENST00000441246	POLR2B
isotig15959	6.062	4.76	4.961	5.044	2583	0.135765011	isogroup03188	ENSG00000164053	ENST00000320211	ATRIP
isotig15960	6.389	5.226	5.196	5.411	2557	0.111435711	isogroup03188	ENSG00000164053	ENST00000320211	ATRIP
isotig15961	8.099	6.559	9.268	5.447	2805	0.633416623	isogroup03189	ENSG00000189306	ENST00000323013	RRP7A
isotig15962	6.644	5.012	7.729	4.254	2334	0.655341174	isogroup03189	ENSG00000189306	ENST00000323013	RRP7A
isotig15963	13.11	17.708	21.043	16.219	3158	0.038522958	isogroup03190	ENSG00000100714	ENST00000216605	MTHFD1
isotig15964	7.293	9.872	11.01	9.175	1958	0.049870369	isogroup03190	ENSG00000100714	ENST00000545908	MTHFD1
isotig15967	2.967	3.096	3.449	2.374	3046	0.261554069	isogroup03192	ENSG00000259958	ENST00000564173	KAT2A.1
isotig15968	2.806	2.757	2.956	2.111	2068	0.272337867	isogroup03192	ENSG00000259958	ENST00000564173	KAT2A.1
isotig15969	3.555	6.358	3.403	4.824	2868	0.639550612	isogroup03193	ENSG00000172331	ENST00000418040	BPGM
isotig15970	1.992	5.17	2.247	3.693	2231	0.739817389	isogroup03193	ENSG00000172331	ENST00000418040	BPGM
isotig15971	17.381	18.011	22.483	21.964	2780	0.021999699	isogroup03194	ENSG00000183576	ENST00000331768	SETD3
isotig15972	11.494	12.15	15.39	15.08	2324	0.053580822	isogroup03194	ENSG00000183576	ENST00000331768	SETD3
isotig15973	4.002	3.365	3.653	2.754	2727	0.228122417	isogroup03195	ENSG00000116584	ENST00000361247	ARHGEF2
isotig15974	3.921	3.226	3.703	2.56	2366	0.26129105	isogroup03195	ENSG00000116584	ENST00000361247	ARHGEF2
isotig15975	1.728	1.647	1.531	2.327	3583	0.180158939	isogroup03196	ENSG00000072121	ENST00000555452	ZFYVE26
isotig15976	5.319	4.981	7.075	5.044	1498	0.310325393	isogroup03196	ENSG00000072121	ENST00000555452	ZFYVE26
isotig16210	5.717	5.016	6.737	4.893	3493	0.36471406	isogroup03314	ENSG00000149532	ENST00000439958	CPSF7
isotig16212	3.082	3.444	3.501	2.903	2252	0.006660029	isogroup03315	ENSG00000100258	ENST00000474879	LMF2
isotig16213	2.85	3.149	3.282	2.649	2155	0.046479297	isogroup03315	ENSG00000100258	ENST00000474879	LMF2
isotig16216	14.853	13.308	11.842	9.568	2197	0.184714812	isogroup03317	ENSG00000221886	ENST00000523213	C5orf54
isotig16218	39.292	22.478	34.801	34.731	2225	0.56092658	isogroup03318	ENSG00000179912	ENST00000403821	R3HDM2
isotig16219	39.451	22.409	35.055	34.936	2152	0.561076877	isogroup03318	ENSG00000179912	ENST00000403821	R3HDM2
isotig16220	16.84	18.902	16.702	20.754	3744	0.397403622	isogroup03319	ENSG00000198690	ENST00000362065	FAN1
isotig16221	4.565	5.226	4.682	4.889	641	0.290974675	isogroup03319	ENSG00000198690	ENST00000362065	FAN1
isotig16222	1.111	1.481	1.127	1.536	2237	0.220062749	isogroup03320	ENSG00000138614	ENST00000567744	C15orf44
isotig16223	1.041	1.399	1.276	1.672	2149	0.191064853	isogroup03320	ENSG00000138614	ENST00000567744	C15orf44
isotig16224	1.759	2.327	2.091	1.685	2384	0.083029608	isogroup03321	ENSG00000132549	ENST00000357162	VPS13B
isotig16225	1.384	1.667	1.585	1.096	1992	0.054895919	isogroup03321	ENSG00000132549	ENST00000357162	VPS13B
isotig16226	4.374	2.847	3.595	3.353	2260	0.374323664	isogroup03322	ENSG00000151806	ENST00000281543	GUF1
isotig16227	4.183	2.631	3.379	2.93	2120	0.403349741	isogroup03322	ENSG00000151806	ENST00000281543	GUF1
isotig16228	3.63	3.043	5.371	3.598	2355	0.458565041	isogroup03323	ENSG00000115649	ENST00000409789	CNPPD1
isotig16229	4.457	3.508	6.395	4.292	2021	0.511610431	isogroup03323	ENSG00000115649	ENST00000409789	CNPPD1
isotig16230	25.464	28.809	49.236	34.693	2514	0.395393402	isogroup03324	ENSG00000118046	ENST00000326873	STK11
isotig16231	9.164	10.581	17.834	12.385	1860	0.397403622	isogroup03324	ENSG00000118046	ENST00000326873	STK11
isotig16232	24.852	22.282	26.023	19.544	2189	0.482302548	isogroup03325	ENSG00000105568	ENST00000322088	PPP2R1A
isotig16233	33.699	29.44	35.374	25.368	2185	0.520384008	isogroup03325	ENSG00000105568	ENST00000322088	PPP2R1A
isotig16234	5.6	4.331	4.647	4.337	2261	0.228413617	isogroup03326	ENSG00000100564	ENST00000561272	PIGH
isotig16235	5.866	4.153	4.79	4.409	2097	0.308108514	isogroup03326	ENSG00000100564	ENST00000216452	PIGH
isotig16236	4.149	3.197	4.156	3.433	2188	0.386347411	isogroup03327	ENSG00000131446	ENST00000446023	MGAT1
isotig16237	3.798	3.074	3.929	3.026	2164	0.360486962	isogroup03327	ENSG00000131446	ENST00000446023	MGAT1
isotig16238	2.573	1.954	4.094	1.276	2255	0.472176298	isogroup03328	ENSG00000132470	ENST00000449880	ITGB4
isotig16239	2.635	1.966	4.185	1.191	2096	0.508472984	isogroup03328	ENSG00000132470	ENST00000449880	ITGB4
isotig16240	6.884	5.177	7.016	5.316	2708	0.459391674	isogroup03329	ENSG00000258231	ENST00000546977	RP11-362K2.2.1
isotig16241	4.776	5.815	8.888	14.404	1635	0.551279402	isogroup03329	ENSG00000169758	ENST00000388942	C15orf27
isotig16242	11.348	8.758	11.606	9.371	2960	0.412160893	isogroup03330	ENSG00000110429	ENST00000265651	FBXO3
isotig16243	10.036	8.791	9.367	8.695	1375	0.245143533	isogroup03330	ENSG00000110429	ENST00000448981	FBXO3
isotig16244	3.813	2.888	2.137	1.972	2946	0.326933193	isogroup03331	ENSG00000078403	ENST00000307729	MLLT10
isotig16246	2.051	3.361	1.913	2.763	2220	0.511150147	isogroup03332	ENSG00000166321	ENST00000357321	NUDT13
isotig16247	2.212	3.621	2.027	2.966	2113	0.523126926	isogroup03332	ENSG00000166321	ENST00000357321	NUDT13
isotig16248	6.63	8.081	6.303	4.663	2187	0.034305253	isogroup03333	ENSG00000034971	ENST00000037502	MYOC
isotig16249	6.064	7.537	5.779	4.203	2144	0.045878109	isogroup03333	ENSG00000034971	ENST00000037502	MYOC
isotig16250	11.903	7.574	11.394	8.18	2229	0.573504546	isogroup03334	ENSG00000028310	ENST00000467963	BRD9
isotig16251	10.486	6.802	10.109	7.524	2096	0.541707372	isogroup03334	ENSG00000028310	ENST00000483173	BRD9
isotig16252	3.031	5.33	6.247	5.953	3123	0.231297437	isogroup03335	ENSG00000244005	ENST00000374092	NFS1
isotig16253	1.964	3.038	3.439	3.759	1200	0.169271812	isogroup03335	ENSG00000244005	ENST00000374092	NFS1
isotig16254	16.497	12.145	15.737	14.289	2213	0.314007665	isogroup03336	ENSG00000105323	ENST00000392006	HNRNPUL1
isotig16255	15.717	11.711	14.9	13.599	2108	0.299269182	isogroup03336	ENSG00000105323	ENST00000392006	HNRNPUL1
isotig16256	4.868	1.884	3.848	1.97	3313	0.760971669	isogroup03337	ENSG00000123728	ENST00000370874	RAP2C
isotig16257	3.071	1.25	1.956	1.437	1005	0.57179492	isogroup03337	ENSG00000123728	ENST00000370874	RAP2C
isotig16258	1.884	2.299	2.351	2.514	2761	0.247153754	isogroup03338	ENSG00000070495	ENST00000445478	JMJD6
isotig16259	2.626	2.123	3.022	2.635	1555	0.135605321	isogroup03338	ENSG00000070495	ENST00000445478	JMJD6
isotig16260	6.679	5.946	7.997	6.385	2265	0.33224055	isogroup03339	ENSG00000136527	ENST00000453386	TRA2B
isotig16261	7.058	6.606	8.615	7.064	2033	0.287799654	isogroup03339	ENSG00000136527	ENST00000453386	TRA2B
isotig16262	11.086	7.923	12.275	13.24	3402	0.209466822	isogroup03340	ENSG00000100678	ENST00000381269	SLC8A3
isotig16264	3.583	2.498	2.908	2.433	3216	0.393965582	isogroup03341	ENSG00000117682	ENST00000360009	DHDDS
isotig16265	3.169	2.055	2.02	2.196	1065	0.293276095	isogroup03341	ENSG00000117682	ENST00000236342	DHDDS
isotig16266	8.812	4.785	7.151	6.121	2178	0.580446382	isogroup03342	ENSG00000160753	ENST00000368347	RUSC1
isotig16267	8.985	4.971	7.311	6.373	2085	0.547296536	isogroup03342	ENSG00000160753	ENST00000368347	RUSC1

isotig16268	1.134	2.471	1.282	1.612	2149	0.474036222	isogroup03343	ENSG00000144306	ENST00000272732	SCRN3
isotig16269	1.225	2.605	1.309	1.635	2119	0.469555497	isogroup03343	ENSG00000144306	ENST00000272732	SCRN3
isotig16270	4.343	3.686	4.765	3.427	2143	0.422230781	isogroup03344	ENSG00000124160	ENST00000290231	NCOA5
isotig16272	8.205	5.242	5.381	3.71	2148	0.659239498	isogroup03345	ENSG00000163660	ENST00000295926	CCNL1
isotig16273	6.769	4.119	5.114	3.116	2105	0.697893966	isogroup03345	ENSG00000163660	ENST00000295926	CCNL1
isotig16274	2.673	2.517	3.388	2.57	3123	0.188754039	isogroup03346	ENSG00000119596	ENST00000325680	YLPM1
isotig16275	1.361	1.566	1.863	1.198	1130	0.141805065	isogroup03346	ENSG00000119596	ENST00000552421	YLPM1
isotig16276	3.358	2.45	2.923	2.462	3146	0.302979635	isogroup03347	ENSG00000099219	ENST00000462592	ERMP1
isotig16277	2.335	1.506	2.175	1.429	1112	0.327694071	isogroup03347	ENSG00000099219	ENST00000462592	ERMP1
isotig16280	2.327	2.171	1.792	1.409	2220	0.063566168	isogroup03349	ENSG00000167196	ENST00000308275	FBXO22
isotig16281	2.374	2.061	1.775	1.417	2034	0.104597204	isogroup03349	ENSG00000167196	ENST00000308275	FBXO22
isotig16282	5.618	2.986	4.285	3.318	2355	0.590168708	isogroup03350	ENSG00000131759	ENST00000394089	RARA
isotig16283	3.185	1.952	2.38	1.782	1897	0.441431202	isogroup03350	ENSG00000131759	ENST00000394089	RARA
isotig16284	1.461	2.846	2.5	2.852	2304	0.441882092	isogroup03351	ENSG00000130669	ENST00000435673	PAK4
isotig16286	9.02	7.11	8.788	9.269	2182	0.294976328	isogroup03352	ENSG00000198355	ENST00000360612	PIM3
isotig16287	9.889	7.809	9.706	10.007	2067	0.309893289	isogroup03352	ENSG00000198355	ENST00000360612	PIM3
isotig16288	8.577	7.664	12.444	11.354	2162	0.20740024	isogroup03353	ENSG00000198837	ENST00000361217	DENND4B
isotig16289	9.212	8.072	13.337	12.211	2067	0.22339746	isogroup03353	ENSG00000198837	ENST00000361217	DENND4B
isotig16290	4.022	7.209	2.294	5.868	2371	0.740925829	isogroup03354	ENSG00000064225	ENST00000483910	ST3GAL6
isotig16291	4.265	7.507	2.465	5.792	1861	0.736097543	isogroup03354	ENSG00000064225	ENST00000483910	ST3GAL6
isotig16292	13.529	13.137	16.871	13.049	2474	0.244476591	isogroup03355	ENSG00000128245	ENST00000248975	YWHAH
isotig16293	1.284	1.841	3.005	2.236	1742	0.151724656	isogroup03355	ENSG00000204624	ENST00000304391	PTCHD2
isotig16294	14.489	40.423	32.414	21.189	2523	0.538466597	isogroup03356	ENSG00000162461	ENST00000294454	SLC25A34
isotig16296	1.955	1.601	3.822	4.855	3080	0.108279477	isogroup03357	ENSG00000079263	ENST00000392045	SP140
isotig16297	1.385	1.177	3.254	3.678	1132	0.17035207	isogroup03357	ENSG00000185404	ENST00000415673	SP140L
isotig16298	6.47	3.753	3.809	3.955	2196	0.411033666	isogroup03358	ENSG00000163697	ENST00000508593	APBB2
isotig16299	6.998	4.082	4.244	4.368	2019	0.429088074	isogroup03358	ENSG00000163697	ENST00000508593	APBB2
isotig16300	5.951	4.46	5.245	4.051	2230	0.403199444	isogroup03359	ENSG00000039523	ENST00000562597	FAM65A
isotig16301	3.36	2.934	2.809	2.237	1982	0.171638987	isogroup03359	ENSG00000039523	ENST00000562597	FAM65A
isotig16302	44.649	26.857	35.83	24.896	2342	0.726177951	isogroup03360	ENSG00000159348	ENST00000367249	CYB5R1
isotig16303	26.038	15.454	21.554	13.208	1850	0.745998347	isogroup03360	ENSG00000159348	ENST00000367249	CYB5R1
isotig16304	31.281	19.255	20.032	18.781	3793	0.571766739	isogroup03361	ENSG00000140945	ENST00000566620	CDH13
isotig16305	2.789	2.984	3.636	2.887	2105	0.121242579	isogroup03362	ENSG00000105705	ENST00000247001	SUGP1
isotig16306	3.274	3.73	4.541	3.355	2092	0.133745397	isogroup03362	ENSG00000105705	ENST00000247001	SUGP1
isotig16307	4.059	4.248	5.16	4.199	2106	0.176786654	isogroup03363	ENSG00000175482	ENST00000312419	POLD4
isotig16308	3.683	3.739	4.396	3.81	2077	0.133745397	isogroup03363	ENSG00000175482	ENST00000312419	POLD4
isotig16309	7.144	6.416	6.789	6.232	2667	0.194737732	isogroup03364	ENSG00000005156	ENST00000378526	LIG3
isotig16310	13.928	11.141	12.475	11.349	1512	0.347166905	isogroup03364	ENSG00000159111	ENST00000414011	MRPL10
isotig16311	21.361	15.69	26.33	25.141	2715	0.266185091	isogroup03365	ENSG00000055070	ENST00000492354	C1orf144
isotig16312	5.358	3.794	6.157	5.425	1465	0.378268956	isogroup03365	ENSG00000259956	ENST00000563281	RBM15B.1
isotig16313	46.802	34.287	37.94	40.45	2156	0.242738784	isogroup03366	ENSG00000090621	ENST00000372856	PABPC4
isotig16314	53.002	38.612	42.511	45.325	2018	0.2277091	isogroup03366	ENSG00000090621	ENST00000372858	PABPC4
isotig16315	3.012	3.821	3.595	3.109	2142	0.09609604	isogroup03367	ENSG00000144455	ENST00000272902	SUMF1
isotig16316	2.679	3.585	3.351	2.854	2028	0.121242579	isogroup03367	ENSG00000144455	ENST00000272902	SUMF1
isotig16317	6.719	4.525	6.255	5.06	2137	0.540082288	isogroup03368	ENSG00000072958	ENST00000444449	AP1M1
isotig16318	6.163	3.9	5.68	4.456	2035	0.575185992	isogroup03368	ENSG00000072958	ENST00000291439	AP1M1
isotig16319	41.627	22.306	37.922	32.243	2269	0.682094011	isogroup03369	ENSG00000102226	ENST00000377107	USP11
isotig16320	45.964	25.729	43.623	38.029	1902	0.648446306	isogroup03369	ENSG00000102226	ENST00000377107	USP11
isotig16321	7.988	5.908	5.188	5.064	3004	0.408065304	isogroup03370	ENSG00000058600	ENST00000299853	POLR3E
isotig16322	3.371	2.494	2.57	1.798	1164	0.403096115	isogroup03370	ENSG00000058600	ENST00000418581	POLR3E
isotig16323	7.953	6.684	6.599	5.093	3268	0.40601751	isogroup03371	ENSG00000124587	ENST00000304611	PEX6
isotig16324	6.11	4.78	5.412	3.768	894	0.475041332	isogroup03371	ENSG00000124587	ENST00000304611	PEX6
isotig16325	10.115	6.699	13.189	8.459	2317	0.58866574	isogroup03372	ENSG00000146054	ENST00000422067	TRIM7
isotig16326	9.194	6.708	12.343	8.06	1843	0.517866536	isogroup03372	ENSG00000146054	ENST00000422067	TRIM7
isotig16327	2.79	1.565	2.347	1.218	2104	0.507439693	isogroup03373	ENSG00000144677	ENST00000447745	CTDSPL
isotig16328	2.266	1.31	1.942	1.196	2049	0.424738859	isogroup03373	ENSG00000144677	ENST00000447745	CTDSPL
isotig16329	41.114	36.131	43.528	40.506	2240	0.212172165	isogroup03374	ENSG00000198729	ENST00000361131	PPP1R14C
isotig16330	40.626	34.96	41.705	37.688	1883	0.212209739	isogroup03374	ENSG00000198729	ENST00000361131	PPP1R14C
isotig16331	4.426	2.529	3.649	3.597	3003	0.470363343	isogroup03375	ENSG00000197386	ENST00000355072	HTT
isotig16332	2.306	1.169	1.515	1.818	1139	0.290739836	isogroup03375	ENSG00000197386	ENST00000355072	HTT
isotig16333	2.1	2.167	1.142	1.901	2078	0.404646051	isogroup03376	ENSG00000162733	ENST00000367922	DDR2
isotig16334	2.341	2.37	1.25	2.005	2060	0.386497708	isogroup03376	ENSG00000162733	ENST00000367922	DDR2
isotig16335	3.102	3.267	2.868	2.975	2540	0.153283986	isogroup03377	ENSG00000111641	ENST00000382421	NOP2
isotig16336	2.523	2.494	2.692	1.93	1595	0.170915683	isogroup03377	ENSG00000111641	ENST00000541778	NOP2
isotig16337	6.646	3.236	3.858	2.637	2144	0.676448486	isogroup03378	ENSG00000176834	ENST00000359236	VSIG10
isotig16714	3.301	3.66	4.067	3.464	1092	0.159624634	isogroup03567	ENSG00000151748	ENST00000324679	SAV1
isotig16715	1.806	3.984	2.808	3.077	1775	0.493640565	isogroup03568	ENSG00000130810	ENST00000253107	PPAN
isotig16716	2.062	4.2	3.357	3.262	1661	0.433869392	isogroup03568	ENSG00000130810	ENST00000253107	PPAN
isotig16718	37.199	35.289	43.864	43.355	1784	0.056239197	isogroup03570	ENSG00000114867	ENST00000441154	EIF4G1
isotig16719	42.845	41.073	50.845	50.724	1644	0.023962952	isogroup03570	ENSG00000114867	ENST00000441154	EIF4G1
isotig16720	1.114	2.313	2.689	2.588	2366	0.219226723	isogroup03571	ENSG00000114021	ENST00000394140	NIT2
isotig16721	1.133	1.72	1.983	1.98	1057	0.122444954	isogroup03571	ENSG00000114021	ENST00000394140	NIT2
isotig16722	4.467	2.421	5.002	3.147	1757	0.69163786	isogroup03572	ENSG00000141994	ENST00000309061	DUS3L
isotig16723	4.566	2.342	5.066	3.152	1662	0.709316525	isogroup03572	ENSG00000141994	ENST00000309061	DUS3L
isotig16726	1.921	2.075	1.862	1.48	2807	0.063566168	isogroup03574	ENSG00000107614	ENST00000377799	TRDMT1
isotig16728	9.725	9.221	13.223	15.706	2954	0.066844518	isogroup03575	ENSG00000142002	ENST00000357909	DPP9
isotig16729	1.742	1.302	1.528	2.002	453	0.028237018	isogroup03575	ENSG00000142002	ENST00000357909	DPP9
isotig16730	8.002	6.261	8.734	9.041	1742	0.339304501	isogroup03576	ENSG00000162517	ENST00000373703	PEF1
isotig16731	9.025	6.944	10.453	11.012	1664	0.334823777	isogroup03576	ENSG00000162517	ENST00000373703	PEF1
isotig16732	2.278	3.26	3.484	4.537	1749	0.378794995	isogroup03577	ENSG00000168961	ENST00000395473	LGALS9
isotig16733	2.519	3.587	3.787	4.991	1653	0.398962952	isogroup03577	ENSG00000168961	ENST00000395473	LGALS9
isotig16734	15.647	17.623	15.981	16.171	1970	0.189157962	isogroup03578	ENSG00000063169	ENST00000396720	GLTSCR1

isotig16735	19.46	19.581	20.485	18.595	1426	0.022356654	isogroup03578	ENSG00000120896	ENST00000520207	SORBS3
isotig16738	7.192	6.609	13.548	8.344	1705	0.557732772	isogroup03580	ENSG00000171282	ENST00000307745	BAHCC1
isotig16739	7.964	7.026	14.893	9.506	1685	0.572142481	isogroup03580	ENSG00000139613	ENST00000550164	SMARCC2
isotig16740	4.761	2.787	5.507	4.858	1871	0.536954235	isogroup03581	ENSG00000115568	ENST00000440934	ZNF142
isotig16741	3.378	1.816	3.693	3.2	1525	0.489657699	isogroup03581	ENSG00000115568	ENST00000440934	ZNF142
isotig16744	7.193	4.853	5.449	3.753	2067	0.550499737	isogroup03583	ENSG00000132323	ENST00000254654	ILKAP
isotig16745	7.565	6.755	7.03	5.367	1316	0.297258961	isogroup03583	ENSG00000132323	ENST00000254654	ILKAP
isotig16748	5.374	3.3	5.701	3.737	2633	0.612525363	isogroup03585	ENSG00000187325	ENST00000341864	TAF9B
isotig16749	3.359	1.17	2.495	1.77	740	0.571494326	isogroup03585	ENSG00000187325	ENST00000341864	TAF9B
isotig16750	6.933	4.919	6.013	5.385	1904	0.396229428	isogroup03586	ENSG00000076513	ENST00000261739	ANKRD13A
isotig16751	3.063	2.402	2.965	3.594	1470	0.079835801	isogroup03586	ENSG00000076513	ENST00000261739	ANKRD13A
isotig16752	9.214	7.862	11.367	8.268	1955	0.448626663	isogroup03587	ENSG00000105053	ENST00000316763	VRK3
isotig16753	3.848	3.109	3.721	3.349	1417	0.279608477	isogroup03587	ENSG00000105053	ENST00000316763	VRK3
isotig16756	6.433	4.715	6.478	6.195	1720	0.346744195	isogroup03589	ENSG00000139405	ENST00000552495	C12orf52
isotig16757	6.891	4.577	6.535	6.182	1626	0.409389795	isogroup03589	ENSG00000139405	ENST00000552495	C12orf52
isotig16758	1.458	1.853	1.898	1.625	2383	0.034718569	isogroup03590	ENSG00000184719	ENST00000331772	RNLS
isotig16759	1.394	1.348	1.231	1.073	960	0.048414368	isogroup03590	ENSG00000184719	ENST00000371947	RNLS
isotig16760	8.321	13.794	10.771	12.334	1698	0.554971068	isogroup03591	ENSG00000130159	ENST00000270517	ECSIT
isotig16761	8.844	14.967	11.567	13.439	1645	0.583095363	isogroup03591	ENSG00000130159	ENST00000270517	ECSIT
isotig16764	2.989	2.494	2.607	2.019	1774	0.332278124	isogroup03593	ENSG00000140694	ENST00000539279	PARN
isotig16765	2.507	2.244	2.224	1.817	1565	0.242729391	isogroup03593	ENSG00000140694	ENST00000539279	PARN
isotig16768	16.919	14.868	21.561	20.439	2120	0.233983993	isogroup03595	ENSG00000131408	ENST00000253727	NR1H2
isotig16769	10.398	8.952	12.325	11.609	1212	0.250591794	isogroup03595	ENSG00000131408	ENST00000411902	NR1H2
isotig16770	1.716	2.367	1.2	1.296	1693	0.280482077	isogroup03596	ENSG00000167740	ENST00000301391	CYB5D2
isotig16771	1.509	2.347	1.119	1.178	1634	0.335866461	isogroup03596	ENSG00000167740	ENST00000301391	CYB5D2
isotig16772	12.609	8.146	10.639	9.21	1812	0.44481288	isogroup03597	ENSG00000065989	ENST00000352831	PDE4A
isotig16773	12.626	9.712	10.548	9.606	1509	0.25171902	isogroup03597	ENSG00000065989	ENST00000440014	PDE4A
isotig16774	5.302	5.614	2.795	3.167	1919	0.233570677	isogroup03598	ENSG00000115419	ENST00000320717	GLS
isotig16775	3.976	4.776	2.033	2.488	1399	0.351187345	isogroup03598	ENSG00000115419	ENST00000338435	GLS
isotig16776	2.957	2.771	2.249	1.852	2188	0.18122041	isogroup03599	ENSG00000141425	ENST00000399022	RPRD1A
isotig16777	3.615	2.423	2.187	2.662	1127	0.166453746	isogroup03599	ENSG00000141425	ENST00000399022	RPRD1A
isotig16778	4.625	4.869	4.075	2.807	2664	0.149658075	isogroup03600	ENSG00000085449	ENST00000233055	WDFY1
isotig16780	1.385	1.274	2.254	1.844	1651	0.175011272	isogroup03601	ENSG00000008283	ENST00000542042	CYB561
isotig16781	1.217	1.252	2.189	1.792	1649	0.123468851	isogroup03601	ENSG00000008283	ENST00000542042	CYB561
isotig16782	3.045	2.656	2.308	3.497	2021	0.166453746	isogroup03602	ENSG00000143376	ENST00000458013	SNX27
isotig16783	2.162	1.879	1.789	2.451	1276	0.122106786	isogroup03602	ENSG00000143376	ENST00000458013	SNX27
isotig16784	10.044	8.839	8.611	8.214	2750	0.24542534	isogroup03603	ENSG00000108039	ENST00000502935	XPNPEP1
isotig16786	1.262	2.344	2.926	2.662	1884	0.132824829	isogroup03604	ENSG00000176155	ENST00000392343	CCDC57
isotig16787	1.018	1.684	2.202	1.822	1413	0.066496956	isogroup03604	ENSG00000130702	ENST00000252999	LAMA5
isotig16788	13.501	9.928	13.362	19.976	1785	0.186659277	isogroup03605	ENSG00000143171	ENST00000359842	RXRG
isotig16789	16.802	11.31	16.784	21.24	1508	0.06893928	isogroup03605	ENSG00000143171	ENST00000359842	RXRG
isotig16790	5.542	4.386	5.25	4.699	2337	0.234143684	isogroup03606	ENSG00000002330	ENST00000540152	BAD
isotig16791	5.274	5.68	5.857	6.704	960	0.164048997	isogroup03606	ENSG00000002330	ENST00000394532	BAD
isotig16793	8.664	7.924	3.451	2.581	1648	0.062016232	isogroup03607	ENSG00000100196	ENST00000216014	KDELR3
isotig16794	5.172	3.592	3.478	3.016	2260	0.429398061	isogroup03608	ENSG00000141141	ENST00000349699	DDX52
isotig16795	3.183	1.917	2.335	1.594	1018	0.497116179	isogroup03608	ENSG00000141141	ENST00000394367	DDX52
isotig16796	9.193	6.309	7.89	7.635	2029	0.406543549	isogroup03609	ENSG00000182362	ENST00000397701	YBEY
isotig16797	4.703	3.555	4.496	3.604	1261	0.398089351	isogroup03609	ENSG00000182362	ENST00000397701	YBEY
isotig16798	32.09	30.918	42.464	28.615	2509	0.510455024	isogroup03610	ENSG00000185236	ENST00000328024	RAB11B
isotig16799	5.147	4.832	6.384	3.54	778	0.511798302	isogroup03610	ENSG00000090924	ENST00000425673	PLEKHG2
contig25579	298.086	217.08	269.63	193.741	2797	0.640743594	isogroup03611	ENSG00000062485	ENST00000351328	CS
isotig16801	15.779	12.97	18.676	16.718	1789	0.3292534	isogroup03612	ENSG00000173020	ENST00000308595	ADRBK1
isotig16802	8.648	7.327	9.661	8.542	1486	0.231344405	isogroup03612	ENSG00000173020	ENST00000308595	ADRBK1
isotig16803	1.644	2.675	1.983	2.07	1676	0.309555121	isogroup03613	ENSG00000039650	ENST00000322344	PNKP
isotig16804	1.626	2.89	2.105	2.271	1597	0.388028857	isogroup03613	ENSG00000039650	ENST00000322344	PNKP
isotig16807	4.844	13.812	11.007	10.072	2216	0.622595251	isogroup03615	ENSG00000010932	ENST00000367750	FMO1
isotig16808	5.56	11.685	10.922	9.625	1049	0.489723454	isogroup03615	ENSG00000010932	ENST00000433267	FMO1
isotig16809	13.365	10.13	16.768	16.803	1708	0.298884046	isogroup03616	ENSG00000136807	ENST00000373265	CDK9
isotig16810	11.817	9.022	15.279	15.021	1559	0.309686631	isogroup03616	ENSG00000136807	ENST00000373265	CDK9
isotig16811	14.88	12.773	15.882	13.175	1830	0.299269182	isogroup03617	ENSG00000182768	ENST00000379095	NGRN
isotig16812	14.954	13.498	16.599	13.856	1425	0.244598707	isogroup03617	ENSG00000182768	ENST00000379095	NGRN
isotig16813	1.843	1.8	3.047	2.645	1739	0.157041407	isogroup03618	ENSG00000175309	ENST00000308158	AGXT2L2
isotig16814	1.782	1.487	2.546	2.348	1530	0.24857218	isogroup03618	ENSG00000175309	ENST00000308158	AGXT2L2
isotig16815	3.561	2.019	3.308	1.691	1820	0.643439543	isogroup03619	ENSG00000087206	ENST00000323774	UIMC1
isotig16816	2.894	1.812	2.913	1.408	1439	0.582879312	isogroup03619	ENSG00000087206	ENST00000377220	UIMC1
isotig16817	1.919	2.11	2.22	1.75	1759	0.087435184	isogroup03620	ENSG00000137857	ENST00000431588	DUOX1
isotig16818	1.87	2.224	2.434	1.851	1506	0.076266251	isogroup03620	ENSG00000137857	ENST00000431588	DUOX1
isotig16821	3.442	2.063	2.573	2.763	1758	0.347993537	isogroup03622	ENSG00000100372	ENST00000435456	SLC25A17
isotig16822	2.677	1.371	1.683	1.846	1492	0.306098294	isogroup03622	ENSG00000100372	ENST00000542412	SLC25A17
isotig16823	5.707	3.086	5.317	6.279	1804	0.353798753	isogroup03623	ENSG00000167619	ENST00000301204	TMEM145
isotig16824	4.103	2.702	4.036	4.669	1448	0.24500263	isogroup03623	ENSG00000167619	ENST00000301204	TMEM145
isotig16825	2.769	3.033	3.027	3.396	1958	0.153283986	isogroup03624	ENSG00000110719	ENST00000265686	TCIRG1
isotig16826	1.305	1.354	1.442	1.402	1293	0.042270985	isogroup03624	ENSG00000110719	ENST00000265686	TCIRG1
isotig16827	3.152	2.515	3.468	2.899	1869	0.252226272	isogroup03625	ENSG00000135801	ENST00000366676	TAF5L
isotig16828	2.198	1.221	1.978	1.632	1372	0.322057939	isogroup03625	ENSG00000135801	ENST00000366676	TAF5L
isotig16831	5.264	4.655	5.788	4.428	1734	0.256547306	isogroup03627	ENSG00000008130	ENST00000341426	NADK
isotig16832	6.146	5.543	6.862	5.156	1511	0.231184715	isogroup03627	ENSG00000008130	ENST00000341426	NADK
isotig16833	8.491	6.272	8.113	6.3	2038	0.434827534	isogroup03628	ENSG00000086589	ENST00000199814	RBM22
isotig16834	4.387	3.321	4.597	3.344	1195	0.404655445	isogroup03628	ENSG00000086589	ENST00000540000	RBM22
isotig16835	3.606	2.299	3.129	2.822	1983	0.341803186	isogroup03629	ENSG00000173327	ENST00000309100	MAP3K11
isotig16836	4.754	2.542	3.328	2.482	1254	0.524742617	isogroup03629	ENSG00000173327	ENST00000530153	MAP3K11
isotig16837	8.559	7.207	7.722	5.891	2702	0.452365296	isogroup03630	ENSG00000102900	ENST00000308159	NUP93

isotig16843	15.922	12.435	19.901	19.58	2365	0.344048245	isogroup03633	ENSG00000225748	ENST00000551278	PRRC2A
isotig16844	6.321	5.151	7.71	7.164	861	0.329413091	isogroup03633	ENSG00000206427	ENST00000552505	PRRC2A
isotig16845	13.3	15.688	15.132	14.046	1664	0.112760201	isogroup03634	ENSG00000108840	ENST00000393622	HDAC5
isotig16846	12.283	13.564	13.444	12.508	1566	0.183596979	isogroup03634	ENSG00000108840	ENST00000225983	HDAC5
isotig16847	8.177	6.413	12.327	12.508	1668	0.179266551	isogroup03635	ENSG00000196498	ENST00000404621	NCOR2
isotig16848	8.752	6.871	13.189	13.635	1554	0.152588863	isogroup03635	ENSG00000196498	ENST00000447011	NCOR2
isotig16849	7.317	5.11	10.057	8.029	1680	0.520355828	isogroup03636	ENSG00000122515	ENST00000441627	ZMIZ2
isotig16850	6.061	3.876	7.905	5.615	1537	0.586909146	isogroup03636	ENSG00000122515	ENST00000441627	ZMIZ2
isotig16853	1.952	1.758	2.013	1.472	2046	0.290044713	isogroup03638	ENSG00000197548	ENST00000354449	ATG7
isotig16855	5.55	4.64	6.403	5.833	1612	0.250995717	isogroup03639	ENSG00000173327	ENST00000530153	MAP3K11
isotig16861	6.818	5.506	8.343	6.49	1630	0.466342902	isogroup03642	ENSG00000148719	ENST00000444643	DNAJB12
isotig16862	6.868	5.489	8.195	6.612	1576	0.474120764	isogroup03642	ENSG00000148719	ENST00000444643	DNAJB12
isotig16863	7.308	4.584	4.579	2.555	1958	0.616057338	isogroup03643	ENSG00000182013	ENST00000313683	PNMAL1
isotig16864	3.836	2.364	2.485	2.047	1252	0.376953859	isogroup03643	ENSG00000182013	ENST00000438932	PNMAL1
isotig16865	6.806	9.772	9.273	8.014	1834	0.093212219	isogroup03644	ENSG00000102119	ENST00000369842	EMD
isotig16866	8.208	12.18	10.709	9.77	1376	0.221114827	isogroup03644	ENSG00000102119	ENST00000369842	EMD
isotig16867	5.35	7.595	11.752	11.636	1693	0.163429022	isogroup03645	ENSG00000157778	ENST00000404674	PSMG3
isotig16868	5.013	6.374	10.117	9.604	1514	0.078163748	isogroup03645	ENSG00000157778	ENST00000404674	PSMG3
isotig16869	16.109	10.34	15.513	13.075	1642	0.537198467	isogroup03646	ENSG00000055211	ENST00000367419	C6orf72
isotig16870	14.962	9.375	14.096	11.852	1545	0.560099947	isogroup03646	ENSG00000055211	ENST00000367419	C6orf72
isotig16871	5.949	4.419	4.682	3.449	1613	0.494946269	isogroup03647	ENSG00000104765	ENST00000380629	BNIP3L
isotig16872	5.477	4.098	4.157	3.074	1577	0.468775832	isogroup03647	ENSG00000104765	ENST00000380629	BNIP3L
isotig16873	1.289	1.675	1.148	1.268	1898	0.202712858	isogroup03648	ENSG00000120662	ENST00000379480	MTRF1
isotig16875	14.978	10.155	16.259	14.481	1716	0.467921019	isogroup03649	ENSG00000116604	ENST00000454816	MEF2D
isotig16876	18.191	12.366	19.828	17.628	1470	0.464529947	isogroup03649	ENSG00000116604	ENST00000368240	MEF2D
isotig16877	34.668	23.301	32.356	22.989	1617	0.650221688	isogroup03650	ENSG00000126457	ENST00000454376	PRMT1
isotig16878	37.112	25.081	34.419	24.829	1563	0.644125272	isogroup03650	ENSG00000126457	ENST00000391851	PRMT1
isotig16879	12.419	11.426	13.926	12.422	1904	0.295690238	isogroup03651	ENSG00000108591	ENST00000225729	DRG2
isotig16880	5.598	3.837	4.95	4.222	1270	0.433117908	isogroup03651	ENSG00000108591	ENST00000225729	DRG2
isotig16881	4.662	5.919	5.62	4.526	1600	0.006660029	isogroup03652	ENSG00000099956	ENST00000263121	SMARCB1
isotig16882	4.539	5.718	5.423	4.419	1573	0.014428496	isogroup03652	ENSG00000099956	ENST00000407422	SMARCB1
isotig16883	2.995	1.854	2.585	1.773	1704	0.429914707	isogroup03653	ENSG00000167566	ENST00000335999	NCKAP5L
isotig16884	2.556	1.529	2.256	1.6	1469	0.400090178	isogroup03653	ENSG00000167566	ENST00000335999	NCKAP5L
isotig16885	4.754	5.626	3.917	4.936	1678	0.307094011	isogroup03654	ENSG00000158092	ENST00000481752	NCK1
isotig16886	3.772	4.807	3.32	4.206	1485	0.321344029	isogroup03654	ENSG00000158092	ENST00000469404	NCK1
isotig16888	2.243	1.366	1.979	1.883	975	0.187795897	isogroup03655	ENSG00000076108	ENST00000549884	BAZ2A
isotig16889	2.059	2.321	1.713	2.315	1644	0.294065154	isogroup03656	ENSG00000162642	ENST00000471115	C1orf52
isotig16890	1.902	2.365	1.71	2.45	1517	0.363023221	isogroup03656	ENSG00000162642	ENST00000471115	C1orf52
isotig16893	6.551	5.329	6.331	5.141	2393	0.323927256	isogroup03658	ENSG00000140463	ENST00000268057	BBS4
isotig16894	2.408	1.847	2.625	1.864	763	0.259590817	isogroup03658	ENSG00000140463	ENST00000268057	BBS4
isotig16895	6.4	5.885	6.236	5.471	1596	0.113539866	isogroup03659	ENSG00000213246	ENST00000225504	SUPT4H1
isotig16896	6.807	6.408	6.954	6.058	1552	0.114169234	isogroup03659	ENSG00000213246	ENST00000225504	SUPT4H1
isotig16897	3.057	2.777	3.151	2.037	1676	0.282849252	isogroup03660	ENSG00000101331	ENST00000452892	C20orf160
isotig16898	2.607	1.862	2.669	1.717	1467	0.419525438	isogroup03660	ENSG00000101331	ENST00000452892	C20orf160
isotig16899	17.13	14.245	18.804	20.961	1852	0.085312242	isogroup03661	ENSG00000165238	ENST00000448039	WNK2
isotig16900	10.354	8.419	12.038	14.31	1300	0.096302698	isogroup03661	ENSG00000165238	ENST00000448039	WNK2
isotig16901	10.172	6.335	11.716	6.505	1929	0.750037574	isogroup03662	ENSG00000105355	ENST00000221957	PLIN3
isotig16902	6.747	4.305	7.209	4.236	1218	0.704413091	isogroup03662	ENSG00000105355	ENST00000221957	PLIN3
isotig16903	5.13	3.527	3.012	3.26	1600	0.358730367	isogroup03663	ENSG00000152242	ENST00000321319	C18orf25
isotig16904	5.411	3.89	3.06	3.346	1539	0.328473736	isogroup03663	ENSG00000152242	ENST00000321319	C18orf25
isotig16905	13.015	10.184	8.512	6.869	1628	0.471077253	isogroup03664	ENSG00000181929	ENST00000316299	PRKAG1
isotig16906	12.387	10.094	7.941	6.853	1501	0.406402645	isogroup03664	ENSG00000181929	ENST00000316299	PRKAG1
isotig16907	7.3	7.574	8.806	8.514	2532	0.007420906	isogroup03665	ENSG00000171953	ENST00000474627	ATPAF2
isotig16908	2.58	2.775	2.893	3.358	600	0.097401743	isogroup03665	ENSG00000171953	ENST00000474627	ATPAF2
isotig16909	3.512	2.108	3.312	2.257	1706	0.447292778	isogroup03666	ENSG00000213445	ENST00000534313	SIPA1
isotig16910	3.642	2.654	3.55	2.854	1420	0.327186819	isogroup03666	ENSG00000213445	ENST00000534313	SIPA1
isotig16915	3.333	1.165	2.737	1.54	1601	0.694380777	isogroup03669	ENSG00000083750	ENST00000262850	RRAGB
isotig16916	3.214	1.154	2.657	1.478	1517	0.680243481	isogroup03669	ENSG00000083750	ENST00000374941	RRAGB
isotig16917	4.366	2.195	3.858	2.478	2168	0.63012888	isogroup03670	ENSG00000176658	ENST00000318217	MYO1D
isotig16918	2.331	1.281	2.485	1.931	951	0.369410836	isogroup03670	ENSG00000176658	ENST00000394649	MYO1D
isotig16919	1.64	2.619	1.925	2.164	1650	0.379039227	isogroup03671	ENSG00000173614	ENST00000462686	NMNAT1
isotig16920	1.505	2.104	1.65	1.81	1466	0.2728733	isogroup03671	ENSG00000173614	ENST00000462686	NMNAT1
isotig16921	6.305	6.887	4.456	5.686	2439	0.28818479	isogroup03672	ENSG00000078070	ENST00000265594	MCCC1
isotig16922	2.802	2.375	2.304	2.1	677	0.303975351	isogroup03672	ENSG00000078070	ENST00000492597	MCCC1
isotig16923	9.539	14.598	13.083	11.374	1858	0.295013902	isogroup03673	ENSG00000028528	ENST00000559844	SNX1
isotig16924	6.442	9.988	8.253	7.355	1254	0.365089802	isogroup03673	ENSG00000028528	ENST00000559844	SNX1
isotig16925	5.165	4.817	5.09	3.448	1620	0.375742091	isogroup03674	ENSG00000141101	ENST00000268802	NOB1
isotig16926	4.962	4.372	4.836	3.18	1489	0.395881867	isogroup03674	ENSG00000141101	ENST00000268802	NOB1
isotig16928	1.896	10.572	2.271	2.844	1480	0.949105734	isogroup03675	ENSG00000106483	ENST00000446575	SFRP4
isotig16929	128.83	154.008	139.81	182.536	2036	0.457127827	isogroup03676	ENSG00000185100	ENST00000332972	ADSSL1
isotig16930	54.724	64.983	56.93	80.007	1070	0.452027129	isogroup03676	ENSG00000185100	ENST00000332972	ADSSL1
isotig16931	4.61	4.247	4.084	3.381	1553	0.261976779	isogroup03677	ENSG00000174173	ENST00000309922	RG9MTD1
isotig16932	4.021	3.637	3.434	2.883	1557	0.236088149	isogroup03677	ENSG00000174173	ENST00000309922	RG9MTD1
isotig16933	14.656	8.321	9.694	7.315	2061	0.698964831	isogroup03678	ENSG00000011243	ENST00000397410	AKAP8L
isotig16934	4.671	2.925	4.771	2.785	1041	0.642472007	isogroup03678	ENSG00000011243	ENST00000397410	AKAP8L
isotig16935	4.222	3.11	4.266	3.765	1781	0.406214774	isogroup03679	ENSG00000226479	ENST00000426077	TMEM185B
isotig16936	1.504	1.455	2.216	1.618	1321	0.144745247	isogroup03679	ENSG00000153551	ENST00000334983	CMTM7
isotig16939	1.981	2.663	2.583	2.411	1880	0.16059217	isogroup03681	ENSG00000103274	ENST00000433392	NUBP1
isotig16940	2.156	2.959	3.069	2.787	1209	0.091530773	isogroup03681	ENSG00000103274	ENST00000283027	NUBP1
isotig16943	3.28	1.661	2.367	2.273	1650	0.467507703	isogroup03683	ENSG00000084693	ENST00000323064	AGBL5
isotig16944	2.163	1.157	2.245	1.431	1431	0.484030961	isogroup03683	ENSG00000084693	ENST00000323064	AGBL5
isotig16945	2.704	2.332	2.858	2.181	1673	0.259590817	isogroup03684	ENSG00000136936	ENST00000375128	XPA

isotig16946	2.901	2.31	2.768	2.269	1401	0.284944014	isogroup03684	ENSG00000136936	ENST00000375128	XPA
isotig16947	1.849	2.101	2.121	1.893	2111	0.104597204	isogroup03685	ENSG00000162062	ENST00000567489	C16orf59
isotig17223	2.376	3.391	2.922	2.177	1311	0.178749906	isogroup03824	ENSG00000213398	ENST00000264005	LCAT
isotig17226	12.299	13.796	14.012	9.092	2322	0.139954535	isogroup03826	ENSG00000170540	ENST00000304414	ARL6IP1
isotig17227	5.513	5.159	6.171	3.958	1496	0.369476591	isogroup03827	ENSG00000148484	ENST00000377921	RSU1
isotig17228	4.116	3.624	4.291	3.181	1179	0.299654317	isogroup03827	ENSG00000148484	ENST00000377921	RSU1
isotig17229	6.065	7.439	12.09	13.111	1383	0.120566243	isogroup03828	ENSG00000116663	ENST00000376753	FBXO6
isotig17230	5.928	7.169	11.596	12.548	1297	0.107396483	isogroup03828	ENSG00000116663	ENST00000376753	FBXO6
isotig17231	18.589	9.048	18.11	14.422	1380	0.762023747	isogroup03829	ENSG00000105808	ENST00000262940	RASA4
isotig17232	22.576	11.19	21.416	17.241	1292	0.759243255	isogroup03829	ENSG00000105808	ENST00000541884	RASA4
isotig17233	12.389	11.098	12.396	13.122	1944	0.082719621	isogroup03830	ENSG00000184182	ENST00000272930	UBE2F
isotig17234	8.38	9.435	8.956	10.016	703	0.121280153	isogroup03830	ENSG00000184182	ENST00000434655	UBE2F
isotig17235	4.83	2.857	3.491	3.127	1346	0.512080108	isogroup03831	ENSG00000162300	ENST00000294258	ZFPL1
isotig17236	3.998	2.44	2.959	2.684	1326	0.45123807	isogroup03831	ENSG00000162300	ENST00000294258	ZFPL1
isotig17237	29.098	24.388	31.083	21.961	1895	0.517641091	isogroup03832	ENSG00000100519	ENST00000445930	PSMC6
isotig17240	4.537	4.297	4.659	3.275	1391	0.277241302	isogroup03834	ENSG00000050393	ENST00000379170	CCDC90A
isotig17241	3.475	3.058	3.56	2.676	1276	0.251906891	isogroup03834	ENSG00000050393	ENST00000379170	CCDC90A
isotig17242	4.874	5.066	5.007	4.69	1809	0.131340648	isogroup03835	ENSG00000161956	ENST00000429205	SENP3
isotig17243	3.061	3.161	3.045	2.688	858	0.06991621	isogroup03835	ENSG00000161956	ENST00000429205	SENP3
isotig17244	2.89	3.651	3.821	2.868	1358	0.049513414	isogroup03836	ENSG00000116353	ENST00000263702	MECR
isotig17245	2.672	3.045	3.53	2.727	1297	0.121242579	isogroup03836	ENSG00000116353	ENST00000263702	MECR
isotig17248	3.13	6.565	4.187	4.798	1528	0.628578943	isogroup03838	ENSG00000167797	ENST00000301488	CDK2AP2
isotig17249	1.24	2.585	1.626	1.93	1119	0.458893815	isogroup03838	ENSG00000167797	ENST00000531506	CDK2AP2
isotig17250	4.374	12.196	17.722	252.224	2160	0.98527091	isogroup03839	ENSG00000124449	ENST00000244314	IRGC
isotig17258	8.222	4.028	7.127	6.589	1342	0.597636582	isogroup03843	ENSG00000205890	ENST00000382225	AC108134.1
isotig17259	9.675	4.525	8.407	7.601	1287	0.625375742	isogroup03843	ENSG00000205890	ENST00000382225	AC108134.1
isotig17260	9.247	12.868	8.813	5.487	2244	0.313312542	isogroup03844	ENSG00000140092	ENST00000556154	FBLN5
isotig17261	11.171	8.796	11.608	9.057	2282	0.464858721	isogroup03845	ENSG00000187522	ENST00000378372	HSPA14
isotig17262	12.284	11.81	17.044	11.763	342	0.325016908	isogroup03845	ENSG00000104852	ENST00000544278	SNRNP70
isotig17263	4.952	4.736	4.384	4.746	1849	0.10625047	isogroup03846	ENSG00000157823	ENST00000558011	AP3S2
isotig17264	1.27	1.833	1.232	1.38	779	0.230931089	isogroup03846	ENSG00000157823	ENST00000560251	AP3S2
isotig17265	16.937	13.391	15.903	12.406	1366	0.378391072	isogroup03847	ENSG00000169738	ENST00000306869	DCXR
isotig17266	12.162	9.76	12.038	8.745	1257	0.403406102	isogroup03847	ENSG00000169738	ENST00000306869	DCXR
isotig17267	10.294	11.398	2.84	4.51	1973	0.424438266	isogroup03848	ENSG00000196628	ENST00000457482	TCF4
isotig17268	3.169	3.153	1.009	1.052	649	0.153293379	isogroup03848	ENSG00000196628	ENST00000566286	TCF4
isotig17269	12.29	7.82	18.496	11.404	1680	0.587294281	isogroup03849	ENSG00000173295	ENST00000310542	AC068020.1
isotig17271	3.075	2.246	2.253	2.515	1500	0.194089577	isogroup03850	ENSG00000140326	ENST00000562465	CDAN1
isotig17272	2.825	2.25	2.115	2.455	1117	0.077975877	isogroup03850	ENSG00000140326	ENST00000356231	CDAN1
isotig17273	1.745	1.707	1.656	1.531	1316	0.010060494	isogroup03851	ENSG00000234545	ENST00000445716	FAM133B
isotig17274	1.676	1.553	1.564	1.475	1275	0.010060494	isogroup03851	ENSG00000234545	ENST00000445716	FAM133B
isotig17275	4.061	3.749	3.444	2.872	1871	0.18463027	isogroup03852	ENSG00000175354	ENST00000327283	PTPN2
isotig17277	4.237	5.278	4.291	3.947	1659	0.214426618	isogroup03853	ENSG00000119333	ENST00000372715	WDR34
isotig17278	1.565	2.109	1.678	1.462	957	0.090788683	isogroup03853	ENSG00000119333	ENST00000451652	WDR34
isotig17279	20.697	19.308	25.152	21.98	1320	0.265508755	isogroup03854	ENSG00000136425	ENST00000258930	CIB2
isotig17281	8.355	11.62	9.779	9.131	1335	0.274028707	isogroup03855	ENSG00000137070	ENST00000555003	IL11RA
isotig17282	8.858	12.615	10.746	9.824	1275	0.276630721	isogroup03855	ENSG00000137070	ENST00000555003	IL11RA
isotig17283	15.718	8.656	6.819	8.506	1404	0.519942511	isogroup03856	ENSG00000106993	ENST00000381854	CDC37L1
isotig17284	14.367	7.477	6.152	7.793	1208	0.548461336	isogroup03856	ENSG00000106993	ENST00000381854	CDC37L1
isotig17285	1.512	1.885	2.519	2.395	1631	0.034718569	isogroup03857	ENSG00000145439	ENST00000306193	CBR4
isotig17287	6.628	8.469	6.322	4.38	1380	0.017143233	isogroup03858	ENSG00000107281	ENST00000371601	NPDC1
isotig17288	5.128	6.449	4.9	3.434	1219	0.029937251	isogroup03858	ENSG00000107281	ENST00000371601	NPDC1
isotig17289	2.909	4.372	2.756	2.683	1396	0.331601789	isogroup03859	ENSG00000158793	ENST00000368009	NIT1
isotig17290	2.498	3.874	2.671	2.725	1202	0.381688209	isogroup03859	ENSG00000158793	ENST00000392190	NIT1
isotig17291	4.614	2.881	5.253	4.292	1450	0.506594274	isogroup03860	ENSG00000107185	ENST00000456972	RGP1
isotig17292	3.104	2.097	4.012	3.047	1139	0.443150222	isogroup03860	ENSG00000107185	ENST00000456972	RGP1
isotig17293	6.68	6.717	7.338	6.845	1740	0.167731269	isogroup03861	ENSG00000011132	ENST00000316757	APBA3
isotig17294	3.631	3.328	3.777	3.079	858	0.255993086	isogroup03861	ENSG00000011132	ENST00000316757	APBA3
isotig17295	20.365	13.178	21.876	16.168	1521	0.691046066	isogroup03862	ENSG00000130204	ENST00000426677	TOMM40
isotig17296	16.103	10.421	16.883	12.204	1074	0.680844668	isogroup03862	ENSG00000130204	ENST00000426677	TOMM40
isotig17297	4.804	2.279	2.671	2.165	1324	0.518289246	isogroup03863	ENSG00000171823	ENST00000339235	FBXL14
isotig17298	4.784	2.471	2.397	2.174	1267	0.467244683	isogroup03863	ENSG00000171823	ENST00000339235	FBXL14
isotig17301	9.545	9.759	11.553	10.507	1337	0.051805441	isogroup03865	ENSG00000228405	ENST00000456167	RNF5
isotig17302	10.403	11.088	13.254	12.031	1242	0.021407906	isogroup03865	ENSG00000228405	ENST00000456167	RNF5
isotig17305	2.379	1.916	1.998	1.469	1326	0.28961261	isogroup03867	ENSG00000167733	ENST00000423665	HSD11B1L
isotig17306	2.18	1.841	1.919	1.384	1246	0.262230405	isogroup03867	ENSG00000167733	ENST00000339423	HSD11B1L
isotig17311	9.23	5.232	12.155	5.806	2020	0.7894811	isogroup03870	ENSG00000213337	ENST00000443120	ANKRD39
isotig17312	4.643	4.548	5.465	3.91	544	0.355968663	isogroup03870	ENSG00000213337	ENST00000443120	ANKRD39
isotig17313	19.382	14.698	21.478	22.593	1578	0.333489893	isogroup03871	ENSG00000233348	ENST00000451932	BAG6
isotig17314	22.272	15.972	24.642	24.591	985	0.436339896	isogroup03871	ENSG00000233348	ENST00000451932	BAG6
isotig17315	48.549	47.915	47.03	56.221	1500	0.198269708	isogroup03872	ENSG00000005882	ENST00000503176	PDK2
isotig17316	39.226	36.768	35.781	44.204	1061	0.166078004	isogroup03872	ENSG00000005882	ENST00000503176	PDK2
isotig17317	2.455	2.385	2.156	2.003	1285	0.166209514	isogroup03873	ENSG00000162971	ENST00000354611	TYW5
isotig17319	58.861	47.905	77.686	91.069	1339	0.04077741	isogroup03874	ENSG00000008441	ENST00000360105	NFIX
isotig17320	62.007	51.562	83.623	98.881	1216	0.073917863	isogroup03874	ENSG00000008441	ENST00000264825	NFIX
isotig17323	1.914	3.368	4.016	4.292	1283	0.310287818	isogroup03876	ENSG00000177156	ENST00000319006	TALDO1
isotig17325	4.483	2.043	2.439	3.923	1980	0.145975802	isogroup03877	ENSG00000167548	ENST00000301067	MLL2
isotig17329	14.203	10.258	12.092	7.961	1863	0.601976403	isogroup03879	ENSG00000122565	ENST00000396386	CBX3
isotig17330	8.609	6.084	7.459	5.367	682	0.537743293	isogroup03879	ENSG00000122565	ENST00000396386	CBX3
isotig17331	2.493	1.775	1.763	1.289	1346	0.302049673	isogroup03880	ENSG00000148154	ENST00000374279	UGCG
isotig17332	2.013	1.34	1.408	1.166	1200	0.241047945	isogroup03880	ENSG00000148154	ENST00000374279	UGCG
isotig17333	9.415	5.454	7.388	5.345	1977	0.612628692	isogroup03881	ENSG00000186625	ENST00000367411	KATNA1
isotig17334	2.294	1.418	1.724	1.285	557	0.323993011	isogroup03881	ENSG00000186625	ENST00000367411	KATNA1

isotig17335	5.66	6.008	5.944	4.925	1923	0.236614188	isogroup03882	ENSG00000105656	ENST00000262809	ELL
isotig17336	2.815	2.869	2.874	1.705	617	0.369767791	isogroup03882	ENSG00000105656	ENST00000262809	ELL
isotig17337	5.576	6.513	4.329	4.697	1318	0.223378673	isogroup03883	ENSG00000172171	ENST00000306049	TEFM
isotig17338	5.312	5.724	4.1	4.386	1207	0.133529345	isogroup03883	ENSG00000172171	ENST00000306049	TEFM
isotig17339	11.848	9.149	10.94	7.731	1319	0.539133539	isogroup03884	ENSG00000140395	ENST00000558311	WDR61
isotig17340	11.387	8.509	10.264	7.102	1190	0.568695048	isogroup03884	ENSG00000140395	ENST00000558311	WDR61
isotig17341	10.056	9.644	9.676	7.327	1271	0.371477418	isogroup03885	ENSG00000172062	ENST00000380707	SMN1
isotig17347	15.069	19.11	21.946	27.721	1257	0.411973022	isogroup03888	ENSG00000160539	ENST00000372264	PPAPDC3
isotig17348	15.294	19.812	22.797	28.884	1249	0.436612309	isogroup03888	ENSG00000160539	ENST00000372264	PPAPDC3
isotig17349	5.457	1.538	4.832	3.652	1689	0.721011498	isogroup03889	ENSG00000143786	ENST00000272133	CNIH3
isotig17351	1.702	1.974	3.83	2.482	1678	0.266279026	isogroup03890	ENSG00000169087	ENST00000306103	HSPBAP1
isotig17352	1.678	1.437	1.53	1.581	821	0.060513264	isogroup03890	ENSG00000169087	ENST00000383659	HSPBAP1
isotig17353	5.97	4.628	4.237	3.301	2079	0.45123807	isogroup03891	ENSG00000120798	ENST00000333003	NR2C1
isotig17355	76.011	88.801	86.01	97.106	1854	0.335152551	isogroup03892	ENSG00000182606	ENST00000449246	TRAK1
isotig17358	8.396	8.241	5.213	5.024	1916	0.006660029	isogroup03894	ENSG00000185722	ENST00000341657	ANKFY1
isotig17360	5.423	2.242	2.576	2.401	1333	0.622163147	isogroup03895	ENSG00000185946	ENST00000533099	RNPC3
isotig17361	4.534	1.749	2.096	1.92	1154	0.62344067	isogroup03895	ENSG00000185946	ENST00000533099	RNPC3
isotig17362	23.905	24.93	17.778	18.225	1264	0.086007365	isogroup03896	ENSG00000177697	ENST00000530320	CD151
isotig17363	25.471	25.358	18.767	19.767	1222	0.039152326	isogroup03896	ENSG00000177697	ENST00000530320	CD151
isotig17364	5.92	4.001	7.604	4.068	1508	0.705784549	isogroup03897	ENSG00000123191	ENST00000418097	ATP7B
isotig17365	1.07	2.335	2.427	2.493	970	0.263780341	isogroup03897	ENSG00000123191	ENST00000418097	ATP7B
isotig17368	3.743	3.591	4.564	4.06	1574	0.201397761	isogroup03899	ENSG00000100416	ENST00000290846	TRMU
isotig17369	2.99	2.34	3.301	2.264	895	0.416547682	isogroup03899	ENSG00000100416	ENST00000290846	TRMU
isotig17370	581.129	406.744	367.654	374.626	2044	0.447208236	isogroup03900	ENSG00000204628	ENST00000512805	GNB2L1
isotig17373	52.047	72.541	77.401	67.269	2054	0.229719321	isogroup03902	ENSG00000119689	ENST00000334220	DLST
isotig17374	1.663	3.575	1.39	2.519	1435	0.628409859	isogroup03903	ENSG00000214013	ENST00000566442	GANC
isotig17375	1.507	2.585	1.438	2.344	1027	0.45049598	isogroup03903	ENSG00000214013	ENST00000566442	GANC
isotig17378	9.497	6.058	8.766	7.571	1307	0.469874878	isogroup03905	ENSG00000242372	ENST00000374436	EIF6
isotig17379	9.325	5.682	8.606	7.265	1134	0.496289547	isogroup03905	ENSG00000242372	ENST00000374436	EIF6
isotig17380	13.363	7.202	10.653	8.188	1274	0.703389194	isogroup03906	ENSG00000141971	ENST00000543795	FAM125A
isotig17381	11.869	6.857	9.726	7.456	1177	0.64988352	isogroup03906	ENSG00000141971	ENST00000543795	FAM125A
isotig17386	2.414	5.062	5.485	3.459	1961	0.076068986	isogroup03909	ENSG00000107147	ENST00000488444	KCNT1
isotig17388	5.308	4.702	6.07	5.076	1676	0.404223341	isogroup03910	ENSG00000188603	ENST00000569430	CLN3
isotig17389	2.714	2.575	3.194	2.329	769	0.239451041	isogroup03910	ENSG00000188603	ENST00000569430	CLN3
isotig17390	2.121	1.404	2.133	1.126	1342	0.428853235	isogroup03911	ENSG00000170464	ENST00000302060	DNAJC18
isotig17394	3.944	3.33	3.612	3.229	1352	0.194174119	isogroup03913	ENSG00000114554	ENST00000393409	PLXNA1
isotig17395	3.234	3.074	3.015	2.595	1087	0.158844969	isogroup03913	ENSG00000076356	ENST00000367033	PLXNA2
isotig17398	411.143	296.558	370.501	518.82	1309	0.075326896	isogroup03915	ENSG00000081248	ENST00000362061	CACNA1S
isotig17399	75.025	49.77	55.353	75.355	1120	0.121054708	isogroup03915	ENSG00000081248	ENST00000362061	CACNA1S
isotig17400	48.172	33.408	31.528	24.263	1217	0.623421883	isogroup03916	ENSG00000139684	ENST00000378720	ESD
isotig17401	52.53	35.826	34.353	25.903	1197	0.647769971	isogroup03916	ENSG00000139684	ENST00000378720	ESD
isotig17402	16.783	11.283	18.679	13.056	1250	0.600971293	isogroup03917	ENSG00000119655	ENST00000434013	NPC2
isotig17403	14.05	9.738	15.906	11.578	1162	0.552942061	isogroup03917	ENSG00000119655	ENST00000434013	NPC2
isotig17404	4.596	3.954	4.773	4.421	1271	0.164171113	isogroup03918	ENSG00000106034	ENST00000450913	C7orf58
isotig17405	5.105	4.643	4.865	4.664	1152	0.094884271	isogroup03918	ENSG00000106034	ENST00000450913	C7orf58
isotig17406	1.313	1.79	1.214	1.297	1217	0.230931089	isogroup03919	ENSG00000152464	ENST00000378197	RPP38
isotig17407	1.556	1.949	1.393	1.587	1206	0.216690464	isogroup03919	ENSG00000152464	ENST00000378197	RPP38
isotig17408	15.654	12.578	20.858	11.757	1906	0.71467085	isogroup03920	ENSG00000167508	ENST00000301012	MVD
isotig17409	5.051	4.239	6.79	3.963	514	0.651038927	isogroup03920	ENSG00000167508	ENST00000568133	MVD
isotig17410	2.47	1.674	1.646	1.472	1217	0.332860525	isogroup03921	ENSG00000181513	ENST00000398322	ACBD4
isotig17411	2.034	1.595	1.71	1.576	1193	0.238295634	isogroup03921	ENSG00000181513	ENST00000398322	ACBD4
isotig17412	3.769	3.185	2.643	2.437	1240	0.193676261	isogroup03922	ENSG00000101417	ENST00000409299	PXMP4
isotig17413	3.24	2.871	2.416	2.32	1177	0.112901105	isogroup03922	ENSG00000101417	ENST00000409299	PXMP4
isotig17414	2.374	1.461	1.748	1.053	1817	0.36984294	isogroup03923	ENSG00000150048	ENST00000315330	CLEC1A
isotig17416	11.09	15.248	15.937	9.193	1220	0.255476441	isogroup03924	ENSG00000005022	ENST00000317881	SLC25A5
isotig17418	1.652	2.128	1.474	1.384	1221	0.237290524	isogroup03925	ENSG00000038002	ENST00000264595	AGA
isotig17419	1.61	2.07	1.449	1.287	1191	0.210133764	isogroup03925	ENSG00000038002	ENST00000264595	AGA
isotig17420	31.046	28.785	30.368	22.086	1265	0.429820771	isogroup03926	ENSG00000188612	ENST00000420826	SUMO2
isotig17421	20.975	17.741	20.462	14.76	1133	0.497548283	isogroup03926	ENSG00000184900	ENST00000411651	SUMO3
isotig17430	2.276	2.286	2.033	1.515	1803	0.14560006	isogroup03931	ENSG00000198919	ENST00000463306	DZIP3
isotig17434	4.013	4.001	5.004	4.913	1727	0.017143233	isogroup03933	ENSG00000174744	ENST00000359957	BRMS1
isotig17435	3.91	4.042	4.645	4.045	664	0.090957767	isogroup03933	ENSG00000135451	ENST00000547923	TROAP
isotig17436	6.935	5.524	7.908	5.39	1568	0.499229729	isogroup03934	ENSG00000143811	ENST00000343818	PYCR2
isotig17437	4.371	3.287	5.059	3.61	834	0.450580522	isogroup03934	ENSG00000143811	ENST00000343818	PYCR2
isotig17438	96.416	97.176	94.899	77.047	2108	0.21580747	isogroup03935	ENSG00000113558	ENST00000522855	SKP1
isotig17439	2.197	2.703	2.659	2.237	1236	0.109575787	isogroup03936	ENSG00000196290	ENST00000416651	NIF3L1
isotig17440	1.612	2.008	1.907	1.661	1152	0.095898775	isogroup03936	ENSG00000196290	ENST00000416651	NIF3L1
isotig17441	3.451	2.894	2.865	2.282	1447	0.300490343	isogroup03937	ENSG00000168438	ENST00000368932	CDC40
isotig17443	3.237	2.92	3.501	2.575	1382	0.349731344	isogroup03938	ENSG00000147813	ENST00000449291	NAPRT1
isotig17444	3.109	3.116	3.261	2.278	1002	0.291472533	isogroup03938	ENSG00000147813	ENST00000276844	NAPRT1
isotig17445	47.151	88.012	77.405	59.542	1453	0.375263019	isogroup03939	ENSG00000130414	ENST00000404554	NDUFA10
isotig17446	22.644	40.348	33.089	28.365	923	0.406750207	isogroup03939	ENSG00000130414	ENST00000404554	NDUFA10
isotig17447	4.41	3.6	4.146	3.664	1216	0.229747501	isogroup03940	ENSG00000177576	ENST00000318240	C18orf32
isotig17448	4.903	4.132	4.525	4.292	1163	0.17548095	isogroup03940	ENSG00000177576	ENST00000318240	C18orf32
isotig17449	3.51	2.28	2.883	2.145	1252	0.371261366	isogroup03941	ENSG00000104660	ENST00000321250	LEPROTL1
isotig17450	2.429	1.515	1.742	1.442	1118	0.260943488	isogroup03941	ENSG00000104660	ENST00000518001	LEPROTL1
isotig17451	6.071	4.493	6.021	4.141	1449	0.558700308	isogroup03942	ENSG00000110844	ENST00000380281	PRPF40B
isotig17452	2.416	2.19	2.432	1.902	930	0.236510859	isogroup03942	ENSG00000110844	ENST00000380281	PRPF40B
isotig17457	32.224	30.053	35.001	38.747	1211	0.023643571	isogroup03945	ENSG00000125970	ENST00000246194	RALY
isotig17458	29.982	28.428	33.193	36.873	1159	0.072048546	isogroup03945	ENSG00000125970	ENST00000375114	RALY
isotig17459	22.564	12.739	22.493	19.628	1217	0.646811828	isogroup03946	ENSG00000105221	ENST00000424901	AKT2
isotig17727	1.956	3.788	3.074	2.864	985	0.392509581	isogroup04081	ENSG00000088038	ENST00000471126	CNOT3

isotig17728	1.273	3.233	1.474	1.316	1710	0.515320884	isogroup04082	ENSG00000120820	ENST00000546436	GLT8D2
isotig17730	65.814	76.356	68.08	98.251	1088	0.808972721	isogroup04083	ENSG00000163395	ENST00000412892	IGFN1
isotig17731	28.428	35.989	30.71	47.186	993	0.835471932	isogroup04083	ENSG00000163395	ENST00000412892	IGFN1
isotig17738	10.298	9.622	11.391	8.823	1086	0.364432254	isogroup04087	ENSG00000125901	ENST00000380325	MRPS26
isotig17739	11.987	11.463	13.362	10.378	988	0.346133614	isogroup04087	ENSG00000125901	ENST00000380325	MRPS26
isotig17740	26.731	31.642	33.743	27.954	1130	0.055976178	isogroup04088	ENSG00000159713	ENST00000562206	TPPP3
isotig17741	17.158	21.279	23.175	19.305	937	0.023211468	isogroup04088	ENSG00000159713	ENST00000562206	TPPP3
isotig17744	5.507	2.946	4.681	3.852	1418	0.555656797	isogroup04090	ENSG00000166896	ENST00000300145	XRCC6BP1
isotig17745	2.497	1.62	2.12	2.064	640	0.282567446	isogroup04090	ENSG00000166896	ENST00000300145	XRCC6BP1
isotig17746	4.886	3.713	4.501	3.468	1120	0.368293004	isogroup04091	ENSG00000124541	ENST00000244496	RRP36
isotig17747	4.049	3.001	3.568	2.412	942	0.432169159	isogroup04091	ENSG00000124541	ENST00000244496	RRP36
isotig17750	26.342	26.31	17.181	25.109	1036	0.505016157	isogroup04093	ENSG00000143028	ENST00000369872	SYPL2
isotig17751	31.443	33.725	22.19	32.999	1021	0.530651161	isogroup04093	ENSG00000143028	ENST00000369872	SYPL2
isotig17754	34.843	31.571	59.903	87.718	1625	0.244204178	isogroup04095	ENSG00000182108	ENST00000331808	DEXI
isotig17755	5.138	6.597	10.391	14.06	414	0.26816713	isogroup04095	ENSG00000197576	ENST00000428284	HOXA4
isotig17759	55.557	62.759	73.287	56.8	935	0.228883295	isogroup04097	ENSG00000182512	ENST00000331334	GLRX5
isotig17762	10.659	14.103	15.06	12.011	1573	0.083405351	isogroup04099	ENSG00000165917	ENST00000298854	RAPSN
isotig17763	2.606	4.248	5.229	4.242	475	0.029937251	isogroup04099	ENSG00000165917	ENST00000524487	RAPSN
isotig17764	4.068	4.12	4.161	3.853	1409	0.0314684	isogroup04100	ENSG00000104976	ENST00000221573	SNAPC2
isotig17766	26.543	23.898	27.462	22.065	1217	0.324171489	isogroup04101	ENSG00000105202	ENST00000221801	FBL
isotig17767	15.027	12.774	15.775	11.587	824	0.413109642	isogroup04101	ENSG00000105202	ENST00000221801	FBL
isotig17768	2.885	3.894	2.251	4.048	1222	0.535357331	isogroup04102	ENSG00000150907	ENST00000379561	FOXO1
isotig17769	9.185	3.238	1.278	2.19	823	0.700693244	isogroup04102	ENSG00000204060	ENST00000372591	FOXO6
isotig17770	11.657	13.399	10.054	8.234	1135	0.035300969	isogroup04103	ENSG00000175110	ENST00000495075	MRPS22
isotig17771	5.279	6.87	5.319	4.534	906	0.149188397	isogroup04103	ENSG00000175110	ENST00000495075	MRPS22
isotig17776	3.166	2.714	2.555	2.898	1066	0.017143233	isogroup04106	ENSG00000175324	ENST00000311351	LSM1
isotig17777	3.845	3.109	3.046	3.611	961	0.064082814	isogroup04106	ENSG00000175324	ENST00000311351	LSM1
isotig17779	3.195	2.111	1.774	1.218	947	0.361229052	isogroup04107	ENSG00000150054	ENST00000540098	MPP7
isotig17780	766.053	6994.349	4111.406	898.476	1371	0.63544563	isogroup04108	ENSG00000257341	ENST00000477724	AL928654.7.1
isotig17781	23.524	25.144	31.958	29.79	620	0.239817389	isogroup04108	ENSG00000257341	ENST00000477724	AL928654.7.1
isotig17782	2.32	2.054	1.619	1.632	1214	0.028237018	isogroup04109	ENSG00000138386	ENST00000409641	NAB1
isotig17783	2.994	1.58	2.895	2.441	807	0.462181559	isogroup04109	ENSG00000166886	ENST00000357680	NAB2
isotig17784	2.563	2.373	2.048	2.525	1095	0.190848801	isogroup04110	ENSG00000129038	ENST00000395162	LOXL1
isotig17785	1.973	1.932	1.425	2.043	926	0.280143909	isogroup04110	ENSG00000129038	ENST00000395162	LOXL1
isotig17786	16.601	22.355	16.083	19.031	1221	0.383848726	isogroup04111	ENSG00000119138	ENST00000377126	KLF9
isotig17787	4.974	6.17	5.026	5.408	799	0.045004509	isogroup04111	ENSG00000119138	ENST00000377126	KLF9
isotig17792	6.443	3.882	7.318	4.739	1167	0.741094912	isogroup04114	ENSG00000165494	ENST00000533018	PCF11
isotig17793	4.429	2.806	4.226	3.088	841	0.5480762	isogroup04114	ENSG00000165494	ENST00000530660	PCF11
isotig17798	5.419	4.146	6.259	3.784	1056	0.450956264	isogroup04117	ENSG00000072163	ENST00000544917	LIMS2
isotig17799	5.898	4.925	6.736	4.427	943	0.354249643	isogroup04117	ENSG00000072163	ENST00000544917	LIMS2
isotig17802	5.166	9.591	7.876	10.486	1409	0.649733223	isogroup04119	ENSG00000188735	ENST00000449592	TMEM120B
isotig17803	2.088	4.014	3.532	4.321	588	0.545849929	isogroup04119	ENSG00000188735	ENST00000449592	TMEM120B
isotig17804	19.518	23.497	22.717	19.061	949	0.086223416	isogroup04120	ENSG00000070831	ENST00000400259	CDC42
isotig17805	11.83	13.276	12.933	10.81	1023	0.007815435	isogroup04120	ENSG00000070831	ENST00000315554	CDC42
isotig17806	5.516	3.671	2.68	5.334	1068	0.151104682	isogroup04121	ENSG00000177932	ENST00000315475	ZNF354C
isotig17807	3.146	2.895	2.052	3.224	927	0.213036372	isogroup04121	ENSG00000234284	ENST00000444149	ZNF879
isotig17812	1.696	2.856	3.73	3.387	1213	0.166453746	isogroup04124	ENSG00000197982	ENST00000373042	C1orf122
isotig17813	1.488	2.08	2.706	2.978	778	0.143242279	isogroup04124	ENSG00000197982	ENST00000373042	C1orf122
isotig17816	33.321	30.884	47.058	49.666	1198	0.08869392	isogroup04126	ENSG00000125818	ENST00000335877	PSMF1
isotig17817	41.628	36.871	61.231	62.996	784	0.213224243	isogroup04126	ENSG00000125818	ENST00000381899	PSMF1
isotig17818	16.718	17.364	15.681	16.853	1151	0.159652814	isogroup04127	ENSG00000101220	ENST00000399672	C20orf27
isotig17819	8.153	9.061	7.826	8.308	834	0.229183888	isogroup04127	ENSG00000101220	ENST00000399672	C20orf27
isotig17820	2.825	2.062	2.974	1.802	1043	0.453314045	isogroup04128	ENSG00000126883	ENST00000411637	NUP214
isotig17821	2.486	1.993	2.407	1.674	936	0.25259262	isogroup04128	ENSG00000106299	ENST00000223023	WASL
isotig17822	3.469	2.478	3.154	2.571	1206	0.388470354	isogroup04129	ENSG00000118894	ENST00000427587	FAM86A
isotig17823	2.329	1.374	1.999	1.745	776	0.362487788	isogroup04129	ENSG00000158483	ENST00000359244	FAM86C1
isotig17826	1.148	1.6	2.229	1.816	1226	0.060513264	isogroup04131	ENSG00000124588	ENST00000380430	NQO2
isotig17828	6.473	9.685	5.681	6.664	1248	0.419309386	isogroup04132	ENSG00000148218	ENST00000409155	ALAD
isotig17829	3.047	4.596	2.501	2.741	731	0.37357218	isogroup04132	ENSG00000148218	ENST00000448137	ALAD
isotig17830	1.222	2.56	2.944	2.647	1201	0.17035207	isogroup04133	ENSG00000085563	ENST00000543174	ABCB1
isotig17831	1.144	2.069	2.721	2.766	777	0.210180732	isogroup04133	ENSG00000204267	ENST00000556934	TAP2
isotig17832	13.632	22.783	6.488	10.573	1046	0.786935448	isogroup04134	ENSG00000261677	ENST00000564712	PTPLA.1
isotig17833	11.456	17.737	5.565	8.383	924	0.710697377	isogroup04134	ENSG00000261677	ENST00000564712	PTPLA.1
isotig17834	4.354	6.559	3.065	3.481	1434	0.606663786	isogroup04135	ENSG00000011105	ENST00000537971	TSPAN9
isotig18233	1.887	1.436	1.719	1.231	709	0.205305478	isogroup04336	ENSG00000135213	ENST00000453279	POM121C
isotig18234	5.395	5.667	2.189	2.492	1105	0.076068986	isogroup04337	ENSG00000108510	ENST00000397786	MED13
isotig18242	2.476	2.637	5.579	3.375	859	0.375864207	isogroup04341	ENSG00000158008	ENST00000374280	EXTL1
isotig18243	1.706	1.838	2.84	1.88	673	0.189618246	isogroup04341	ENSG00000123191	ENST00000542656	ATP7B
isotig18247	2.381	1.73	1.788	1.622	759	0.124586684	isogroup04343	ENSG00000185009	ENST00000372745	AP3M1
isotig18248	2.325	1.486	1.673	1.096	1082	0.357518599	isogroup04344	ENSG00000125885	ENST00000378886	MCM8
isotig18256	1.026	2.062	2.076	2.781	974	0.326031412	isogroup04348	ENSG00000121671	ENST00000443527	CRY2
isotig18262	17.645	8.818	19.545	13.41	803	0.776283159	isogroup04351	ENSG00000161671	ENST00000376920	C19orf63
isotig18263	20.613	10.367	22.719	15.821	716	0.777325844	isogroup04351	ENSG00000161671	ENST00000376920	C19orf63
isotig18266	116.639	128.154	89.545	93.106	887	0.12201285	isogroup04353	ENSG00000084207	ENST00000398606	GSTP1
isotig18267	217.304	252.296	166.555	186.058	622	0.232537386	isogroup04353	ENSG00000084207	ENST00000398606	GSTP1
isotig18270	16.235	18.945	21.622	21.053	965	0.023211468	isogroup04355	ENSG00000256053	ENST00000409074	APOPT1
isotig18271	2.478	1.226	1.412	1.274	536	0.311621703	isogroup04355	ENSG00000140798	ENST00000529084	ABCC12
isotig18272	26.32	22.06	32.401	19.032	1101	0.600745848	isogroup04356	ENSG00000205581	ENST00000361263	HMGN1
isotig18273	8.547	9.553	8.165	5.952	805	0.227558804	isogroup04357	ENSG00000198931	ENST00000378364	APRT
isotig18274	6.566	6.981	6.044	4.779	698	0.13943789	isogroup04357	ENSG00000198931	ENST00000378364	APRT
isotig18277	1.789	1.648	2.247	2.479	826	0.014428496	isogroup04359	ENSG00000144401	ENST00000458426	METTL21A
isotig18278	1.51	1.65	2.052	2.437	675	0.193929887	isogroup04359	ENSG00000144401	ENST00000448007	METTL21A

isotig18281	1.198	2.452	4.004	2.213	784	0.170915683	isogroup04361	ENSG00000111490	ENST00000542120	TBC1D30
isotig18287	34.443	26.823	37.337	31.279	997	0.456244834	isogroup04364	ENSG00000099817	ENST00000215587	POLR2E
isotig18288	6.483	4.844	6.439	5.429	499	0.416425565	isogroup04364	ENSG00000161681	ENST00000391813	SHANK1
isotig18291	29.526	32.774	37.861	40.213	1021	0.300997595	isogroup04366	ENSG00000105819	ENST00000444457	PMPCB
isotig18292	3.938	4.058	3.996	5.107	469	0.320808597	isogroup04366	ENSG00000167419	ENST00000543544	LPO
isotig18295	4.885	3.862	5.463	5.009	791	0.339586308	isogroup04368	ENSG00000121644	ENST00000302550	PPPDE1
isotig18296	3.768	3.237	4.185	4.079	697	0.213205456	isogroup04368	ENSG00000121644	ENST00000302550	PPPDE1
isotig18299	42.334	37.008	52.497	56.02	835	0.150710153	isogroup04370	ENSG00000105085	ENST00000263390	MED26
isotig18301	6.061	3.117	6.178	4.671	1191	0.660601563	isogroup04371	ENSG00000139266	ENST00000266643	9-Mar
isotig18303	15.263	4.757	16.576	11.075	917	0.887399489	isogroup04372	ENSG00000149256	ENST00000278550	ODZ4
isotig18304	18.372	7.959	20.014	12.642	564	0.866649132	isogroup04372	ENSG00000169047	ENST00000305123	IRS1
isotig18305	1.807	10.308	2.429	1.956	950	0.914650184	isogroup04373	ENSG00000204262	ENST00000374866	COL5A2
isotig18307	5.769	4.759	4.338	4.363	862	0.335443751	isogroup04374	ENSG00000185650	ENST00000557086	ZFP36L1
isotig18308	15.638	14.703	12.967	12.454	613	0.228582701	isogroup04374	ENSG00000185650	ENST00000439696	ZFP36L1
isotig18315	5.539	3.933	4.247	4.492	750	0.20393402	isogroup04378	ENSG00000066855	ENST00000262146	MTFR1
isotig18316	4.591	3.691	3.747	3.922	722	0.122933419	isogroup04378	ENSG00000066855	ENST00000262146	MTFR1
isotig18321	41.718	27.566	41.275	38.627	956	0.480996844	isogroup04381	ENSG00000130725	ENST00000253023	UBE2M
isotig18322	29.566	19.164	28.131	27.131	512	0.452609529	isogroup04381	ENSG00000130725	ENST00000253023	UBE2M
isotig18325	1.265	1.95	2.974	2.64	742	0.091699857	isogroup04383	ENSG00000158292	ENST00000377893	GPR153
isotig18326	1.542	2.218	2.891	3.103	725	0.214623882	isogroup04383	ENSG00000212857	ENST00000391545	AL356585.1
isotig18333	17.028	12.804	18.501	24.64	750	0.129790712	isogroup04387	ENSG00000171992	ENST00000522122	SYNPO
isotig18334	22.316	16.959	24.633	32.541	711	0.131744571	isogroup04387	ENSG00000171992	ENST00000522122	SYNPO
isotig18337	5.721	10.892	11.707	12.62	761	0.540608326	isogroup04389	ENSG00000183828	ENST00000392568	NUDT14
isotig18338	5.273	10.61	11.352	11.659	697	0.546225671	isogroup04389	ENSG00000183828	ENST00000392568	NUDT14
isotig18341	15.486	9.312	10.924	13.785	786	0.395684602	isogroup04391	ENSG00000137285	ENST00000259818	TUBB2B
isotig18342	11.37	10.183	8.985	8.741	671	0.122538889	isogroup04391	ENSG00000176014	ENST00000317702	TUBB6
isotig18343	96.921	92.202	114.417	75.893	987	0.41720523	isogroup04392	ENSG00000108561	ENST00000225698	C1QBP
isotig18344	4.417	7.929	2.03	2.582	737	0.748816412	isogroup04393	ENSG00000112769	ENST00000230538	LAMA4
isotig18346	5.76	6.75	6.328	5.069	839	0.086167055	isogroup04394	ENSG00000164054	ENST00000296444	SHISA5
isotig18347	7.845	9.331	7.903	7.475	611	0.202271361	isogroup04394	ENSG00000101104	ENST00000255136	PABPC1L
isotig18350	19.735	14.557	17.276	15.841	761	0.407398362	isogroup04396	ENSG00000217930	ENST00000318059	PAM16
isotig18351	27.384	18.867	24.116	22.338	683	0.482791012	isogroup04396	ENSG00000217930	ENST00000318059	PAM16
isotig18354	8.552	7.943	7.491	6.237	822	0.277419779	isogroup04398	ENSG00000161920	ENST00000293777	MED11
isotig18355	6.464	6.713	5.367	4.651	621	0.045004509	isogroup04398	ENSG00000161920	ENST00000293777	MED11
isotig18356	1.988	2.122	2.333	1.855	839	0.216728038	isogroup04399	ENSG00000184508	ENST00000394272	HDDC3
isotig18357	1.386	1.512	1.868	1.741	598	0.104832043	isogroup04399	ENSG00000184508	ENST00000394272	HDDC3
isotig18360	2.11	1.749	1.837	1.643	729	0.166247088	isogroup04401	ENSG00000105663	ENST00000420124	MLL4.1
isotig18361	1.222	1.3	1.326	1.067	703	0.042674908	isogroup04401	ENSG00000105663	ENST00000420124	MLL4.1
isotig18368	32.78	27.01	32.005	36.069	778	0.055609829	isogroup04405	ENSG00000161677	ENST00000293431	JOSD2
isotig18369	23.678	19.37	23.945	26.284	652	0.102220636	isogroup04405	ENSG00000161677	ENST00000293431	JOSD2
isotig18372	5.454	3.155	3.675	3.573	736	0.489272563	isogroup04407	ENSG00000233323	ENST00000549232	TNXB
isotig18373	5.176	2.955	3.041	3.392	683	0.422850755	isogroup04407	ENSG00000096717	ENST00000212015	SIRT1
isotig18378	6.391	7.687	3.475	4.382	728	0.259092959	isogroup04410	ENSG00000176406	ENST00000507740	RIMS2
isotig18384	4.071	4.207	4.36	4.054	991	0.161691215	isogroup04413	ENSG00000149761	ENST00000279206	NUDT22
isotig18386	1.588	1.683	2.22	1.651	786	0.121392876	isogroup04414	ENSG00000149489	ENST00000278833	ROM1
isotig18388	9.772	6.262	5.788	5.629	1032	0.549945517	isogroup04415	ENSG00000100320	ENST00000414461	RBFOX2
isotig18389	27.922	19.705	24.661	21.684	383	0.450298715	isogroup04415	ENSG00000100320	ENST00000449924	RBFOX2
isotig18393	8.706	9.26	1.072	1.556	741	0.189336439	isogroup04418	ENSG00000197696	ENST00000360476	NMB
isotig18394	11.793	14.204	1.245	2.215	652	0.075402044	isogroup04418	ENSG00000197696	ENST00000360476	NMB
isotig18395	2.39	2.671	1.533	2.133	777	0.271248215	isogroup04419	ENSG00000112576	ENST00000372991	CCND3
isotig18396	1.459	1.421	1.305	1.137	630	0.016354174	isogroup04419	ENSG00000112576	ENST00000414200	CCND3
isotig18397	4.692	3.367	3.565	3.036	896	0.367964229	isogroup04420	ENSG00000168116	ENST00000370733	KIAA1586
isotig18398	2.918	2.333	2.135	1.778	510	0.211307958	isogroup04420	ENSG00000168116	ENST00000370733	KIAA1586
isotig18401	5.256	4.518	4.967	4.369	948	0.240597054	isogroup04422	ENSG00000076928	ENST00000316079	ARHGEF1
isotig18402	2.174	2.092	2.07	1.82	455	0.03231382	isogroup04422	ENSG00000076928	ENST00000378152	ARHGEF1
isotig18407	50.439	44.893	57.883	52.309	820	0.318169009	isogroup04425	ENSG00000116786	ENST00000375799	PLEKHM2
isotig18408	79.953	70.729	92.929	84.482	579	0.320771023	isogroup04425	ENSG00000116786	ENST00000375799	PLEKHM2
isotig18411	14.16	14.744	15.036	13.139	1007	0.126615691	isogroup04427	ENSG00000214194	ENST00000441359	AC073346.2.1
isotig18416	11.927	11.748	12.691	10.403	977	0.267406252	isogroup04430	ENSG00000185800	ENST00000377735	DMWD
isotig18417	26.275	26.713	26.851	24.547	404	0.207879312	isogroup04430	ENSG00000175265	ENST00000543376	GOLGA8A
isotig18428	2.97	3.115	3.299	2.413	722	0.237929285	isogroup04436	ENSG00000239672	ENST00000336097	NME1
isotig18429	2.085	1.628	2.147	1.383	645	0.345645149	isogroup04436	ENSG00000239672	ENST00000336097	NME1
isotig18430	58.863	43.221	104.339	115.916	846	0.12172165	isogroup04437	ENSG00000154358	ENST00000570156	OBSCN
isotig18431	26.202	19.288	44.964	52.229	524	0.072884572	isogroup04437	ENSG00000124177	ENST00000373233	CHD6
isotig18432	2.8	2.692	4.845	3.861	806	0.174457053	isogroup04438	ENSG00000127528	ENST00000248071	KLF2
isotig18433	3.71	3.854	6.762	5.222	568	0.131340648	isogroup04438	ENSG00000127528	ENST00000248071	KLF2
isotig18434	31.714	30.909	50.688	31.596	767	0.533544375	isogroup04439	ENSG00000173457	ENST00000309318	PPP1R14B
isotig18435	21.499	23.04	36.73	23.3	601	0.416829488	isogroup04439	ENSG00000173457	ENST00000542235	PPP1R14B
isotig18438	35.57	55.175	65.031	39.911	686	0.213224243	isogroup04441	ENSG00000187840	ENST00000338825	EIF4EBP1
isotig18439	20.791	30.783	38.1	23.444	678	0.254809499	isogroup04441	ENSG00000187840	ENST00000338825	EIF4EBP1
isotig18443	2.811	2.216	1.658	1.005	657	0.37386338	isogroup04443	ENSG00000187231	ENST00000428443	SESTD1
isotig18444	3.139	3.834	1.678	1.91	633	0.360336665	isogroup04444	ENSG00000188243	ENST00000355801	COMMD6
isotig18445	2.614	3.313	1.531	1.808	718	0.367259713	isogroup04444	ENSG00000188243	ENST00000355801	COMMD6
isotig18448	1.766	1.126	1.694	1.652	752	0.211730668	isogroup04446	ENSG00000167232	ENST00000397082	ZNF91
isotig18452	2.1	8.706	3.544	4.224	599	0.823955437	isogroup04448	ENSG00000106853	ENST00000407693	PTGR1
isotig18453	1.689	3.273	4.174	3.218	887	0.186405651	isogroup04449	ENSG00000122783	ENST00000393114	C7orf49
isotig18461	2.358	2.887	2.144	1.938	745	0.112769595	isogroup04453	ENSG00000104129	ENST00000220496	DNAJC17
isotig18462	2.397	1.237	1.032	1.063	600	0.246327121	isogroup04453	ENSG00000104129	ENST00000220496	DNAJC17
isotig18463	2.445	1.542	2.073	1.798	784	0.235449388	isogroup04454	ENSG00000153066	ENST00000356957	TXNDC11
isotig18464	1.81	1.234	1.934	1.869	563	0.119880514	isogroup04454	ENSG00000153066	ENST00000283033	TXNDC11
isotig18467	1.874	2.456	1.651	1.362	682	0.136967386	isogroup04456	ENSG00000198399	ENST00000380868	ITSN2
isotig18468	1.477	1.264	1.086	1.401	663	0.055318629	isogroup04456	ENSG00000198399	ENST00000380868	ITSN2

isotig18475	2.681	1.786	2.327	2.025	730	0.291998572	isogroup04460	ENSG00000151353	ENST00000281017	TMEM18
isotig18476	1.836	1.36	1.727	1.507	609	0.20052416	isogroup04460	ENSG00000151353	ENST00000281017	TMEM18
isotig18479	1.293	2.117	2.021	2.244	818	0.202966484	isogroup04462	ENSG00000160972	ENST00000526564	PPP1R16A
isotig18481	3.066	1.246	2.535	2.601	710	0.467507703	isogroup04463	ENSG00000167525	ENST00000415329	PROCA1
isotig18482	2.871	1.414	2.522	2.682	626	0.406675058	isogroup04463	ENSG00000167525	ENST00000301039	PROCA1
isotig18483	11.392	26.26	17.995	13.012	1022	0.651827985	isogroup04464	ENSG00000102760	ENST00000379359	C13orf15
isotig18498	3.04	1.847	1.44	1.087	680	0.46483054	isogroup04472	ENSG00000168152	ENST00000536314	THAP9
isotig18499	1.557	2.321	1.511	1.14	648	0.151978282	isogroup04472	ENSG00000168152	ENST00000536314	THAP9
isotig18508	4.165	13.55	9.719	4.227	911	0.801551815	isogroup04477	ENSG00000049540	ENST00000458204	ELN
isotig18509	1.895	5.766	3.526	1.526	411	0.658281356	isogroup04477	ENSG00000105327	ENST00000449228	BBC3
isotig18517	6.949	5.74	9.219	6.024	589	0.51488878	isogroup04481	ENSG00000109606	ENST00000535946	DHX15
isotig18518	2.603	2.806	1.88	2.838	702	0.324218456	isogroup04482	ENSG00000104381	ENST00000434412	GDAP1
isotig18520	7.18	11.19	5.774	6	694	0.512916134	isogroup04483	ENSG00000167799	ENST00000376693	NUDT8
isotig18521	7.877	13.187	6.056	6.627	625	0.606879838	isogroup04483	ENSG00000167799	ENST00000376693	NUDT8
isotig18522	10.832	9.943	16.878	14.188	826	0.420718419	isogroup04484	ENSG00000166582	ENST00000299736	CENPV
isotig18523	2.578	1.814	4.086	3.234	488	0.377799279	isogroup04484	ENSG00000166582	ENST00000299736	CENPV
isotig18530	8.658	7.813	7.29	6.121	737	0.288729616	isogroup04488	ENSG00000183011	ENST00000335155	LSMD1
isotig18531	13.633	12.529	11.786	9.402	575	0.370472308	isogroup04488	ENSG00000183011	ENST00000335155	LSMD1
isotig18532	3.454	1.891	2.184	1.449	896	0.500657549	isogroup04489	ENSG00000158423	ENST00000457095	RIBC1
isotig18534	2.372	1.662	3.12	2.11	716	0.310419328	isogroup04490	ENSG00000154175	ENST00000383692	ABI3BP
isotig18535	1.654	1.078	1.872	1.115	592	0.283281356	isogroup04490	ENSG00000251322	ENST00000445220	SHANK3
isotig18540	1.542	2.248	3.004	2.712	673	0.106053205	isogroup04493	ENSG00000125952	ENST00000555932	MAX
isotig18554	8.152	14.034	10.184	10.825	670	0.543943037	isogroup04500	ENSG00000184924	ENST00000328379	PTRHD1
isotig18555	7.46	11.095	9.051	9.411	609	0.371073495	isogroup04500	ENSG00000184924	ENST00000328379	PTRHD1
isotig18560	1.6	1.666	1.036	1.725	811	0.189740362	isogroup04503	ENSG00000172671	ENST00000374366	ZFAND4
isotig18564	13.305	25.153	16.817	17.149	812	0.61601037	isogroup04505	ENSG00000090013	ENST00000263368	BLVRB
isotig18565	4.332	7.371	6.169	5.731	461	0.458414744	isogroup04505	ENSG00000090013	ENST00000263368	BLVRB
isotig18570	97.143	87.099	122.488	75.314	685	0.658873149	isogroup04508	ENSG00000177666	ENST00000336615	PNPLA2
isotig18571	31.187	29.741	39.205	25.51	584	0.621064102	isogroup04508	ENSG00000177666	ENST00000336615	PNPLA2
isotig18573	2.591	1.506	1.641	1.825	613	0.321259487	isogroup04509	ENSG00000171606	ENST00000326804	ZNF274
isotig18574	30.762	19.076	31.968	28.731	681	0.453858871	isogroup04510	ENSG00000126351	ENST00000546243	THRA
isotig18575	17.75	12.012	19.038	17.484	580	0.331291801	isogroup04510	ENSG00000126351	ENST00000394121	THRA
isotig18580	12.879	9.848	17.448	11.412	718	0.553965958	isogroup04513	ENSG00000183684	ENST00000505490	ALYREF
isotig18581	6.087	4.935	8.563	4.119	538	0.62298978	isogroup04513	ENSG00000183684	ENST00000505490	ALYREF
isotig18768	41.343	39.083	51.098	43.164	591	0.358890058	isogroup04609	ENSG00000116786	ENST00000375799	PLEKHM2
isotig18769	59.261	55.665	75.196	62.888	490	0.34781506	isogroup04609	ENSG00000116786	ENST00000375799	PLEKHM2
isotig18770	3.16	3.376	4.351	2.433	575	0.330728188	isogroup04610	ENSG00000167037	ENST00000403206	SGSM1
isotig18771	2.231	2.425	2.443	1.559	510	0.129649808	isogroup04610	ENSG00000060566	ENST00000381943	CREB3L3
isotig18792	11.301	9.564	10.493	7.59	615	0.544253025	isogroup04621	ENSG00000205544	ENST00000302422	C17orf61
isotig18793	22.912	19.286	25.18	15.769	455	0.669854212	isogroup04621	ENSG00000205544	ENST00000302422	C17orf61
isotig18794	5.036	2.945	1.737	1.684	585	0.339821147	isogroup04622	ENSG00000079462	ENST00000538771	PAFAH1B3
isotig18808	26.081	23.617	24.56	36.734	621	0.339323288	isogroup04629	ENSG00000070423	ENST00000340092	RNF126
isotig18809	6.505	5.828	5.972	8.112	436	0.167083114	isogroup04629	ENSG00000070423	ENST00000340092	RNF126
isotig18818	8.243	4.564	6.914	5.222	613	0.685006012	isogroup04634	ENSG00000112787	ENST00000434748	FBRSL1
isotig18819	4.425	4.325	4.552	5.114	440	0.014428496	isogroup04634	ENSG00000112787	ENST00000434748	FBRSL1
isotig18826	177.345	162.64	166.854	166.531	964	0.091145638	isogroup04638	ENSG00000103363	ENST00000409906	TCEB2
isotig18828	1.319	2.276	3.28	2.907	439	0.116480048	isogroup04639	ENSG00000197879	ENST00000545534	MYO1C
isotig18831	1.266	2.057	2.095	2.039	560	0.102314571	isogroup04641	ENSG00000131269	ENST00000535115	ABCB7
isotig18835	92.196	80.623	95.742	67.13	751	0.429416848	isogroup04643	ENSG00000196262	ENST00000468812	PPIA
isotig18842	1.705	5.767	2.46	1.471	533	0.525381378	isogroup04647	ENSG00000121335	ENST00000389362	PRB2
isotig18850	1.986	2.269	1.314	1.708	583	0.276142256	isogroup04651	ENSG00000052795	ENST00000512986	FNIP2
isotig18852	19.144	14.808	16.284	13.162	563	0.451848651	isogroup04652	ENSG00000150459	ENST00000382533	SAP18
isotig18853	16.498	13.411	14.028	10.636	462	0.435625986	isogroup04652	ENSG00000150459	ENST00000382533	SAP18
isotig18862	5.123	4.912	2.716	2.587	649	0.231851657	isogroup04657	ENSG00000171634	ENST00000335221	BPTF
isotig18863	3.858	3.573	1.997	2.148	365	0.219818517	isogroup04657	ENSG00000184206	ENST00000510439	GOLGA6L4
isotig18868	10.167	10.641	9.985	9.873	551	0.081423311	isogroup04660	ENSG00000241863	ENST00000455025	RPP21
isotig18869	2.272	2.551	2.043	2.096	470	0.003503795	isogroup04660	ENSG00000206495	ENST00000550618	TRIM39
isotig18872	3.667	2.435	2.185	2.454	520	0.340403547	isogroup04662	ENSG00000197837	ENST00000539745	HIST4H4
isotig18873	4.254	2.78	2.678	2.866	500	0.3981645	isogroup04662	ENSG00000197837	ENST00000539745	HIST4H4
isotig18880	2.727	3.179	2.902	1.627	509	0.074481476	isogroup04666	ENSG00000172380	ENST00000370982	GNG12
isotig18881	1.596	2.215	2.004	1.059	507	0.021652138	isogroup04666	ENSG00000172380	ENST00000370982	GNG12
isotig18884	8.719	9.862	11.942	12.248	552	0.05106335	isogroup04668	ENSG00000074071	ENST00000568758	MRPS34
isotig18885	10.827	12.415	14.616	14.989	463	0.052209363	isogroup04668	ENSG00000074071	ENST00000177742	MRPS34
isotig18888	18.659	14.592	19.65	16.435	662	0.304830165	isogroup04670	ENSG00000186439	ENST00000546248	TRDN
isotig18889	372.057	284.534	355.077	323.586	350	0.280115729	isogroup04670	ENSG00000186439	ENST00000543022	TRDN
isotig18890	157.304	173.509	273.306	310.74	519	0.249145187	isogroup04671	ENSG00000167702	ENST00000301332	KIFC2
isotig18904	21.427	23.033	19.811	24.292	576	0.251202375	isogroup04678	ENSG00000141644	ENST00000436910	MBD1
isotig18905	32.614	35.604	30.731	40.372	417	0.328473736	isogroup04678	ENSG00000141644	ENST00000436910	MBD1
isotig19284	5.416	4.895	4.216	3.586	3920	0.248675509	isogroup04880	ENSG00000172869	ENST00000539542	DMXL1
isotig19286	6.431	5.25	5.638	4.208	3894	0.361773878	isogroup04881	ENSG00000154845	ENST00000400556	PPP4R1
isotig19288	12.229	9.292	11.017	7.948	3890	0.454760652	isogroup04882	ENSG00000136451	ENST00000258963	VEZF1
isotig19290	3.251	4.03	5.046	4.78	3864	0.164171113	isogroup04883	ENSG00000184677	ENST00000404138	ZBTB40
isotig19292	4.892	3.713	5.439	4.471	3850	0.347110543	isogroup04884	ENSG00000100084	ENST00000263208	HIRA
isotig19294	2.232	1.859	1.938	1.62	4926	0.139334561	isogroup04885	ENSG00000107036	ENST00000545641	KIAA1432
isotig19295	2.676	2.159	2.045	1.791	2944	0.216690464	isogroup04885	ENSG00000107036	ENST00000545641	KIAA1432
isotig19296	8.746	7.777	8.839	9.071	3828	0.007420906	isogroup04886	ENSG00000017797	ENST00000383432	RALBP1
isotig19297	8.994	7.989	9.118	9.335	3705	0.006660029	isogroup04886	ENSG00000017797	ENST00000383432	RALBP1
isotig19298	11.457	2.474	3.338	2.766	3800	0.875206658	isogroup04887	ENSG00000175928	ENST00000319331	LRRN1
isotig19299	11.772	2.557	3.414	2.821	3678	0.877705343	isogroup04887	ENSG00000175928	ENST00000319331	LRRN1
isotig19300	5.178	7.88	7.436	5.626	4075	0.180468926	isogroup04888	ENSG00000168300	ENST00000522514	PCMTD1
isotig19301	5.31	8.229	7.364	5.846	3458	0.23497971	isogroup04888	ENSG00000168300	ENST00000522514	PCMTD1
isotig19302	12.591	13.643	16.222	16.273	3755	0.114188021	isogroup04889	ENSG00000167693	ENST00000336868	NXN

isotig19303	12.213	13.202	15.335	15.753	3569	0.142537762	isogroup04889	ENSG00000167693	ENST00000336868	NXN
isotig19304	8.874	15.895	7.357	13.042	3747	0.729935372	isogroup04890	ENSG00000184588	ENST00000371045	PDE4B
isotig19306	6.738	4.129	4.215	2.969	4087	0.627113549	isogroup04891	ENSG00000162819	ENST00000340934	BROX
isotig19307	5.961	4.288	4.275	3.229	3598	0.498309161	isogroup04891	ENSG00000162819	ENST00000340934	BROX
isotig19308	5.877	9.734	4.464	4.491	3694	0.543736379	isogroup04892	ENSG00000101972	ENST00000371145	STAG2
isotig19310	5.117	4.367	5.337	5.52	4143	0.10625047	isogroup04893	ENSG00000072803	ENST00000296933	FBXW11
isotig19311	4.324	3.949	4.499	4.919	3425	0.006660029	isogroup04893	ENSG00000072803	ENST00000296933	FBXW11
isotig19312	12.395	9.158	13.284	10.498	3685	0.480414444	isogroup04894	ENSG00000085721	ENST00000198767	RRN3
isotig19314	9.642	5.96	7.893	7.969	3666	0.353338468	isogroup04895	ENSG00000130338	ENST00000367097	TULP4
isotig19316	8.974	10.818	13.612	11.157	3672	0.110646652	isogroup04896	ENSG00000072952	ENST00000534266	MRVI1
isotig19318	19.418	17.254	17.437	25.432	3646	0.263658225	isogroup04897	ENSG00000073417	ENST00000394553	PDE8A
isotig19320	3.351	4.205	3.922	2.854	3625	0.014428496	isogroup04898	ENSG00000009413	ENST00000435970	REV3L
isotig19321	3.4	4.276	3.987	2.897	3559	0.014428496	isogroup04898	ENSG00000009413	ENST00000435970	REV3L
isotig19322	10.588	10.686	11.072	8.353	7196	0.067239047	isogroup04899	ENSG00000140575	ENST00000268182	IQGAP1
isotig19323	33.877	37.052	35.564	33.337	3596	0.165439243	isogroup04900	ENSG00000150768	ENST00000280346	DLAT
isotig19325	15.744	10.887	14.495	12.437	3600	0.466286541	isogroup04901	ENSG00000196235	ENST00000432763	SUPT5H
isotig19327	5.9	5.869	7.089	5.175	3571	0.301420305	isogroup04902	ENSG00000144749	ENST00000273261	LRIG1
isotig19328	5.93	5.918	7.12	5.254	3496	0.290871346	isogroup04902	ENSG00000144749	ENST00000273261	LRIG1
isotig19329	8.683	7.794	6.477	5.63	7158	0.27559743	isogroup04903	ENSG00000157106	ENST00000389467	SMG1
isotig19330	17.702	27.786	20.413	18.949	3582	0.470100323	isogroup04904	ENSG00000155189	ENST00000285518	AGPAT5
isotig19332	13.563	10.484	20.185	12.236	3553	0.628917111	isogroup04905	ENSG00000160209	ENST00000291565	PDXK
isotig19334	7.668	12.36	15.114	7.738	3576	0.066844518	isogroup04906	ENSG00000122786	ENST00000495522	CALD1
isotig19336	13.056	13.877	12.285	11.117	3532	0.020365221	isogroup04907	ENSG00000122729	ENST00000379923	ACO1
isotig19338	5.961	4.373	5.003	4.311	3521	0.363596228	isogroup04908	ENSG00000154710	ENST00000439720	RABGEF1
isotig19342	51.303	51.498	76.088	70.887	3454	0.057234914	isogroup04910	ENSG00000100380	ENST00000216218	ST13
isotig19344	6.475	5.11	6.03	5.091	3408	0.430628617	isogroup04911	ENSG00000088930	ENST00000377191	XRN2
isotig19346	3.738	5.164	5.598	5.457	6679	0.290974675	isogroup04912	ENSG00000154237	ENST00000388948	LRRK1
isotig19347	16.833	11.409	19.382	19.069	3360	0.394247389	isogroup04913	ENSG00000144043	ENST00000272438	TEX261
isotig19349	3.541	2.762	3.159	2.234	3463	0.357725257	isogroup04914	ENSG00000163811	ENST00000407426	WDR43
isotig19350	3.434	2.744	3.06	2.263	3111	0.329835801	isogroup04914	ENSG00000163811	ENST00000407426	WDR43
isotig19351	20.917	17.295	20.231	15.873	3272	0.421732923	isogroup04915	ENSG00000108588	ENST00000403162	CCDC47
isotig19353	25.509	19.604	22.557	14.886	3271	0.522506951	isogroup04916	ENSG00000168385	ENST00000391972	2-Sep
isotig19359	8.538	10.896	7.437	9.784	6411	0.508454197	isogroup04919	ENSG00000083444	ENST00000196061	PLOD1
isotig19360	20.955	17.336	16.863	17.33	3205	0.277034643	isogroup04920	ENSG00000141540	ENST00000269346	TTYH2
isotig19362	9.881	7.542	9.651	8.271	6423	0.426457879	isogroup04921	ENSG00000003393	ENST00000264276	ALS2
isotig19363	2.566	27.124	5.625	15.972	3207	0.959588938	isogroup04922	ENSG00000146477	ENST00000275300	SLC22A3
isotig19365	2.715	4.544	4.026	3.324	3192	0.26301007	isogroup04923	ENSG00000146648	ENST00000454757	EGFR
isotig19367	5.288	3.769	5.24	3.902	3161	0.534314646	isogroup04924	ENSG00000128513	ENST00000357628	POT1
isotig19369	2.525	2.418	2.223	1.495	3168	0.045840535	isogroup04925	ENSG00000152818	ENST00000367526	UTRN
isotig19370	2.583	2.48	2.268	1.529	3081	0.043172766	isogroup04925	ENSG00000152818	ENST00000367526	UTRN
isotig19371	23.27	24.299	19.135	16.936	3142	0.070808597	isogroup04926	ENSG00000135535	ENST00000310786	CD164
isotig19373	29.481	17.432	15.623	12.301	3112	0.674701285	isogroup04927	ENSG00000119326	ENST00000325551	CTNNAL1
isotig19377	6.316	6.157	7.252	5.744	6251	0.033563162	isogroup04929	ENSG00000145555	ENST00000515803	MYO10
isotig19378	16.63	11.384	15.75	8.619	3105	0.673808898	isogroup04930	ENSG00000129625	ENST00000379638	REEP5
isotig19379	17.326	11.865	16.404	8.977	2979	0.675753363	isogroup04930	ENSG00000129625	ENST00000379638	REEP5
isotig19380	12.265	32.703	20.132	25.361	3079	0.803768693	isogroup04931	ENSG00000251359	ENST00000506413	C4orf38
isotig19382	5.59	4.34	6.585	5.674	3344	0.331094537	isogroup04932	ENSG00000237441	ENST00000497454	RGL2
isotig19383	6.021	4.766	7.168	6.239	3005	0.314289472	isogroup04932	ENSG00000237441	ENST00000497454	RGL2
isotig19384	5.009	2.654	4.836	3.238	3076	0.60656985	isogroup04933	ENSG00000135392	ENST00000357606	DNAJC14
isotig19385	5.148	2.724	4.964	3.323	2991	0.610364846	isogroup04933	ENSG00000135392	ENST00000357606	DNAJC14
isotig19386	2.713	14.913	13.833	7.86	3062	0.609923349	isogroup04934	ENSG00000142207	ENST00000382751	URB1
isotig19388	1.758	1.846	1.564	1.225	3572	0.054135042	isogroup04935	ENSG00000039319	ENST00000510158	ZFYVE16
isotig19389	2.17	2.272	1.881	1.494	2719	0.076266251	isogroup04935	ENSG00000039319	ENST00000510158	ZFYVE16
isotig19390	8.525	7.837	18.203	7.86	3036	0.725266777	isogroup04936	ENSG00000122574	ENST00000409290	WIPF3
isotig19392	3.182	2.601	4.119	3.045	3044	0.310287818	isogroup04937	ENSG00000224994	ENST00000547030	TRIM39
isotig19394	3.568	3.527	3.935	3.852	3022	0.003503795	isogroup04938	ENSG00000168538	ENST00000512476	TRAPPC11
isotig19396	17.278	17.009	16.091	10.831	3026	0.20224318	isogroup04939	ENSG00000078369	ENST00000378606	GNB1
isotig19400	5.28	5.162	9.261	2.952	2935	0.617297287	isogroup04941	ENSG00000151468	ENST00000378825	CCDC3
isotig19402	2.897	2.991	2.259	1.578	2930	0.177697828	isogroup04942	ENSG00000154783	ENST00000285046	FGD5
isotig19403	2.955	3.052	2.31	1.613	2866	0.18122041	isogroup04942	ENSG00000154783	ENST00000285046	FGD5
isotig19404	2.795	2.318	3.015	2.488	2915	0.213036372	isogroup04943	ENSG00000119541	ENST00000238497	VPS4B
isotig19406	73.117	59.587	63.187	71.9	2919	0.105592921	isogroup04944	ENSG00000129244	ENST00000250111	ATP1B2
isotig19408	13.3	6.521	9.536	5.044	2916	0.833640189	isogroup04945	ENSG00000181826	ENST00000454158	RELL1
isotig19410	59.455	51.67	53.233	42.797	2922	0.318441422	isogroup04946	ENSG00000111897	ENST00000368454	SERINC1
isotig19412	5.589	3.874	4.479	2.599	2894	0.49656196	isogroup04947	ENSG00000100644	ENST00000557538	HIF1A
isotig19416	7.81	5.82	6.727	5.898	3158	0.387267979	isogroup04949	ENSG00000125304	ENST00000376387	TM9SF2
isotig19417	8.78	6.475	7.528	6.604	2800	0.404627264	isogroup04949	ENSG00000125304	ENST00000376387	TM9SF2
isotig19418	16.192	8.162	10.902	6.531	2859	0.819296235	isogroup04950	ENSG00000114480	ENST00000264326	GBE1
isotig19420	3.49	3.183	3.71	2.79	4297	0.299889156	isogroup04951	ENSG00000150457	ENST00000542899	LATS2
isotig19424	5.594	3.576	5.367	4.372	2842	0.461777636	isogroup04953	ENSG00000213079	ENST00000417268	SCAF8
isotig19428	37.726	31.495	26.177	24.057	2848	0.240709777	isogroup04955	ENSG00000166598	ENST00000299767	HSP90B1
isotig19432	3.094	15.836	11.979	14.586	2827	0.862459608	isogroup04957	ENSG00000173334	ENST00000311922	TRIB1
isotig19434	3.302	6.065	2.686	2.004	2815	0.549381904	isogroup04958	ENSG00000112769	ENST00000522006	LAMA4
isotig19435	3.434	6.326	2.805	2.084	2696	0.55447321	isogroup04958	ENSG00000112769	ENST00000522006	LAMA4
isotig19436	7.994	10.27	9.601	9.479	2808	0.260905914	isogroup04959	ENSG00000111911	ENST00000229633	HINT3
isotig19438	11.963	12.929	30.05	22.443	2778	0.29347336	isogroup04960	ENSG00000186143	ENST00000335524	C2orf53
isotig19440	23.992	14.421	21.712	13.403	2781	0.744532953	isogroup04961	ENSG00000258289	ENST00000549115	CHURC1
isotig19442	2.403	2.533	2.685	2.612	2751	0.010060494	isogroup04962	ENSG00000137944	ENST00000260508	CCBL2
isotig19443	2.415	2.577	2.697	2.613	2622	0.003503795	isogroup04962	ENSG00000137944	ENST00000260508	CCBL2
isotig19446	5.182	4.446	3.767	2.843	2740	0.261976779	isogroup04964	ENSG00000066777	ENST00000262215	ARFGEF1
isotig19448	1.897	1.849	1.5	1.323	3045	0.047521981	isogroup04965	ENSG00000164631	ENST00000399476	ZNF12
isotig19449	2.115	1.977	1.689	1.454	2617	0.078182536	isogroup04965	ENSG00000164631	ENST00000399476	ZNF12

isotig19450	8.268	10.463	8.829	6.682	2727	0.065905163	isogroup04966	ENSG00000205785	ENST00000431698	AC091435.2
isotig19452	1.717	1.972	1.897	1.595	2983	0.047521981	isogroup04967	ENSG00000157036	ENST00000287675	EXOG
isotig19453	1.666	1.857	1.729	1.564	2440	0.014428496	isogroup04967	ENSG00000157036	ENST00000287675	EXOG
isotig19456	6.145	4.884	7.414	6.405	2894	0.315557601	isogroup04969	ENSG00000186834	ENST00000332499	HEXIM1
isotig19457	7.429	5.995	8.901	7.907	2367	0.290561359	isogroup04969	ENSG00000186834	ENST00000332499	HEXIM1
isotig19464	4.561	4.405	3.177	3.562	2627	0.110299091	isogroup04973	ENSG00000108587	ENST00000451249	GOSR1
isotig19466	48.541	33.963	47.433	37.931	5323	0.565426092	isogroup04974	ENSG00000173402	ENST00000545947	DAG1
isotig19467	15.235	11.594	15.366	11.482	2640	0.413109642	isogroup04975	ENSG00000131236	ENST00000372797	CAP1
isotig19471	15.785	16.092	17.045	12.149	5176	0.188387691	isogroup04977	ENSG00000186591	ENST00000355621	UBE2H
isotig19474	5.086	4.086	4.469	4.15	2576	0.237403246	isogroup04979	ENSG00000129691	ENST00000343823	ASH2L
isotig19475	5.409	4.366	4.706	4.434	2411	0.229512663	isogroup04979	ENSG00000129691	ENST00000343823	ASH2L
isotig19476	67.263	51.167	51.783	41.208	2562	0.516692342	isogroup04980	ENSG00000104408	ENST00000220849	EIF3E
isotig19478	2.542	2.62	3.141	2.43	3386	0.035704892	isogroup04981	ENSG00000261306	ENST00000561828	SURF6.1
isotig19479	2.067	2.352	2.558	2.196	1942	0.06751146	isogroup04981	ENSG00000261306	ENST00000561828	SURF6.1
isotig19480	19.197	24.228	21.542	40.461	2580	0.714821147	isogroup04982	ENSG00000124098	ENST00000371384	FAM210B
isotig19482	3.903	6.036	4.29	3.576	2548	0.289753513	isogroup04983	ENSG00000008405	ENST00000008527	CRY1
isotig19484	5.665	6.144	6.379	5.964	2556	0.098782596	isogroup04984	ENSG00000123106	ENST00000545336	CCDC91
isotig19486	4.515	3.251	2.971	2.113	2787	0.432169159	isogroup04985	ENSG00000114520	ENST00000251775	SNX4
isotig19487	4.965	3.619	3.249	2.339	2498	0.438500413	isogroup04985	ENSG00000114520	ENST00000251775	SNX4
isotig19488	15.406	10.966	15.719	13.231	2555	0.467723754	isogroup04986	ENSG00000182552	ENST00000326397	RWDD4
isotig19490	3.034	4.685	2.414	2.663	2505	0.38729616	isogroup04987	ENSG00000128585	ENST00000352689	MKLN1
isotig19491	3.103	4.792	2.469	2.724	2449	0.390715413	isogroup04987	ENSG00000128585	ENST00000352689	MKLN1
isotig19492	23.214	16.189	22.492	17.148	3141	0.500140903	isogroup04988	ENSG00000121057	ENST00000539273	AKAP1
isotig19493	24.414	17.093	22.925	17.997	2045	0.491104306	isogroup04988	ENSG00000121057	ENST00000539273	AKAP1
isotig19494	3.726	4.444	4.6	5.521	3422	0.335443751	isogroup04989	ENSG00000171566	ENST00000499023	PLRG1
isotig19495	3.048	4.121	3.517	4.693	1746	0.430666191	isogroup04989	ENSG00000171566	ENST00000499023	PLRG1
isotig19498	3.848	4.004	3.165	2.399	2491	0.17035207	isogroup04991	ENSG00000136870	ENST00000374861	ZNF189
isotig19500	7.483	5.636	6.817	4.436	2450	0.535047344	isogroup04992	ENSG00000104763	ENST00000262097	ASAH1
isotig19501	8.111	6.053	7.396	4.794	2253	0.543294882	isogroup04992	ENSG00000104763	ENST00000262097	ASAH1
isotig19502	6.046	5.65	6.52	5.608	2461	0.228037875	isogroup04993	ENSG00000143149	ENST00000354775	ALDH9A1
isotig19503	6.151	5.835	6.729	5.713	2357	0.218428271	isogroup04993	ENSG00000143149	ENST00000354775	ALDH9A1
isotig19504	13.173	9.118	9.221	7.456	2459	0.519942511	isogroup04994	ENSG00000104320	ENST00000265433	NBN
isotig19508	9.973	8.205	9.206	6.332	2429	0.463844217	isogroup04996	ENSG00000120992	ENST00000316963	LYPLA1
isotig19510	2.981	2.403	3.166	2.688	2432	0.206009995	isogroup04997	ENSG00000163900	ENST00000421852	TMEM41A
isotig19513	2.126	3.063	1.675	1.437	2414	0.157924401	isogroup04999	ENSG00000149084	ENST00000278353	HSD17B12
isotig19514	2.222	3.211	1.811	1.545	2202	0.166209514	isogroup04999	ENSG00000149084	ENST00000278353	HSD17B12
isotig19515	14.684	14.422	39.37	4.257	2414	0.953896445	isogroup05000	ENSG00000181039	ENST00000323397	ANKRD34A
isotig19517	13.834	16.38	16.557	13.999	2410	0.007420906	isogroup05001	ENSG00000023734	ENST00000419869	STRAP
isotig19518	14.356	16.976	17.16	14.526	2321	0.007815435	isogroup05001	ENSG00000023734	ENST00000419869	STRAP
isotig19521	15.59	14.556	13.457	11.301	2393	0.142368678	isogroup05003	ENSG00000103769	ENST00000261890	RAB11A
isotig19523	10.537	10.393	5.838	4.312	2391	0.187429548	isogroup05004	ENSG00000157184	ENST00000371486	CPT2
isotig19524	10.017	9.877	5.326	4.151	2198	0.171272638	isogroup05004	ENSG00000157184	ENST00000371486	CPT2
isotig19525	82.549	80.372	92.952	70.799	2409	0.36328624	isogroup05005	ENSG00000127540	ENST00000262946	UQCR11
isotig19526	82.139	81.149	91.316	71.167	2271	0.334823777	isogroup05005	ENSG00000127540	ENST00000262946	UQCR11
isotig19527	14.259	9.549	13.172	12.409	2367	0.492898474	isogroup05006	ENSG00000176407	ENST00000409785	KCMF1
isotig19528	14.713	9.853	13.591	12.803	2294	0.494392049	isogroup05006	ENSG00000176407	ENST00000409785	KCMF1
isotig19529	2.768	2.517	2.881	2.189	2352	0.335302848	isogroup05007	ENSG00000119599	ENST00000358377	DCAF4
isotig19531	4.081	5.202	4.339	3.145	2349	0.101177951	isogroup05008	ENSG00000132669	ENST00000440354	RIN2
isotig19535	2.224	1.851	3.669	2.848	2335	0.351177951	isogroup05010	ENSG00000153391	ENST00000283410	INO80C
isotig19537	3.662	2.373	3.275	2.292	2334	0.507909371	isogroup05011	ENSG00000122484	ENST00000370343	RPAP2
isotig19541	8.508	5.618	6.456	4.447	2331	0.524075674	isogroup05013	ENSG00000169291	ENST00000304760	SHE
isotig19545	11.885	11.704	10.241	11.868	2324	0.095560607	isogroup05015	ENSG00000221914	ENST00000380737	PPP2R2A
isotig19547	4.107	4.784	3.558	3.576	4639	0.264841813	isogroup05016	ENSG00000137845	ENST00000260408	ADAM10
isotig19548	2.641	8.111	3.761	4.614	2484	0.841277147	isogroup05017	ENSG00000102802	ENST00000380482	C13orf33
isotig19549	3.216	9.829	4.579	5.551	1984	0.859181258	isogroup05017	ENSG00000102802	ENST00000380482	C13orf33
isotig19550	34.407	26.664	62.279	47.128	2320	0.555825881	isogroup05018	ENSG00000171766	ENST00000396659	GATM
isotig19552	11.506	7.63	10.758	8.722	4610	0.570160442	isogroup05019	ENSG00000144909	ENST00000296220	OSBPL11
isotig19555	16.456	13.748	15.768	14.243	2301	0.287433306	isogroup05021	ENSG00000113384	ENST00000542582	GOLPH3
isotig19557	4.343	1.43	2.174	2.124	2688	0.637521605	isogroup05022	ENSG00000197535	ENST00000553916	MYO5A
isotig19558	4.399	1.34	2.076	2.196	2059	0.635981063	isogroup05022	ENSG00000197535	ENST00000553916	MYO5A
isotig19561	3.648	4.166	4.486	2.991	2264	0.099505899	isogroup05024	ENSG00000141753	ENST00000269593	IGFBP4
isotig19562	3.959	4.521	4.869	3.246	2086	0.102577591	isogroup05024	ENSG00000141753	ENST00000269593	IGFBP4
isotig19565	4.332	2.597	4.325	1.761	2250	0.569700158	isogroup05026	ENSG00000118729	ENST00000261448	CASQ2
isotig19567	3.568	4.086	3.841	3.821	2250	0.108561284	isogroup05027	ENSG00000113552	ENST00000508177	GNPDA1
isotig19571	15.43	15.364	14.364	11.868	2256	0.213938153	isogroup05029	ENSG00000113048	ENST00000261413	MRPS27
isotig19573	12.006	8.71	9.253	6.648	4506	0.543135192	isogroup05030	ENSG00000068793	ENST00000560848	CYFIP1
isotig19574	6.001	4.362	5.153	3.783	2243	0.535545202	isogroup05031	ENSG00000141076	ENST00000314423	CIRH1A
isotig19576	2.911	3.412	2.848	2.992	2237	0.203492523	isogroup05032	ENSG00000075539	ENST00000537810	FRYL
isotig19578	8.16	4.745	7.204	3.632	4457	0.706667543	isogroup05033	ENSG00000165801	ENST00000298694	ARHGEF40
isotig19579	1.745	2.716	2.258	2.342	2404	0.295436612	isogroup05034	ENSG00000165733	ENST00000374518	BMS1
isotig19580	1.967	3.032	2.541	2.632	2122	0.299081311	isogroup05034	ENSG00000165733	ENST00000374518	BMS1
isotig19581	9.766	9.991	11.144	10.585	2227	0.108645826	isogroup05035	ENSG00000185028	ENST00000328278	LRRC14B
isotig19583	5.804	4.092	4.498	3.145	2221	0.519792215	isogroup05036	ENSG00000126653	ENST00000247026	NSRP1
isotig19584	5.79	4.087	4.492	3.139	2047	0.525099572	isogroup05036	ENSG00000126653	ENST00000247026	NSRP1
isotig19585	5.756	4.654	6.455	5.614	2217	0.402692192	isogroup05037	ENSG00000179918	ENST00000478753	SEPHS2
isotig19589	79.496	404.296	70.246	46.676	2213	0.990296461	isogroup05039	ENSG00000113140	ENST00000231061	SPARC
isotig19591	12.197	6.902	7.154	4.469	4388	0.717704967	isogroup05040	ENSG00000182670	ENST00000399017	TTC3
isotig19592	41.032	40.406	40.389	25.787	2199	0.45824566	isogroup05041	ENSG00000012211	ENST00000417014	PRICKLE3
isotig19594	8.452	6.388	6.824	4.961	2180	0.492851507	isogroup05042	ENSG00000144744	ENST00000361055	UBA3
isotig19595	8.666	6.549	6.996	5.065	2126	0.495462914	isogroup05042	ENSG00000144744	ENST00000361055	UBA3
isotig19596	4.949	4.956	5.246	3.731	2193	0.263761554	isogroup05043	ENSG00000196975	ENST00000394295	ANXA4
isotig19597	5.144	5.142	5.452	3.878	2110	0.266711129	isogroup05043	ENSG00000196975	ENST00000394295	ANXA4

isotig19602	1.391	1.467	1.586	1.466	2154	0.010060494	isogroup05046	ENSG00000100461	ENST00000399922	RBM23
isotig19603	1.419	1.508	1.622	1.516	2083	0.016354174	isogroup05046	ENSG00000100461	ENST00000399922	RBM23
isotig19604	4.303	3.561	4.322	4.459	2149	0.014428496	isogroup05047	ENSG00000115525	ENST00000393808	ST3GAL5
isotig19606	2.979	3.105	3.493	3.36	2149	0.127818066	isogroup05048	ENSG00000185442	ENST00000327355	FAM174B
isotig19607	3.074	3.205	3.579	3.468	2082	0.137606147	isogroup05048	ENSG00000185442	ENST00000327355	FAM174B
isotig19608	2.856	3.557	3.212	3.768	2587	0.251906891	isogroup05049	ENSG00000168310	ENST00000393593	IRF2
isotig19609	3.568	4.486	3.943	4.88	1920	0.304867739	isogroup05049	ENSG00000168310	ENST00000393593	IRF2
isotig19610	6.962	10.519	8.569	8.593	2157	0.496064102	isogroup05050	ENSG00000108468	ENST00000393408	CBX1
isotig19612	9.344	8.25	6.267	5.138	2148	0.105160818	isogroup05051	ENSG00000144118	ENST00000474855	RALB
isotig19614	49.345	25.216	42.746	40.553	2143	0.648840836	isogroup05052	ENSG00000184203	ENST00000328432	PPP1R2
isotig19616	17.563	12.221	16.083	11.58	2140	0.635652288	isogroup05053	ENSG00000100603	ENST00000261531	SNW1
isotig19618	3.589	3.974	3.571	2.83	2112	0.074284211	isogroup05054	ENSG00000080822	ENST00000437922	CLDND1
isotig19619	3.701	4.095	3.7	2.908	1994	0.072142481	isogroup05054	ENSG00000080822	ENST00000437922	CLDND1
isotig19620	7.188	4.04	3.06	2.2	2106	0.573570301	isogroup05055	ENSG00000147853	ENST00000381809	AK3
isotig19622	3.375	2.422	3.168	2.276	2346	0.375732697	isogroup05056	ENSG00000174891	ENST00000480820	RSRC1
isotig19623	4.36	3.132	4.234	3.036	1729	0.41617194	isogroup05056	ENSG00000174891	ENST00000480820	RSRC1
isotig19624	14.884	12.374	11.156	9.445	2112	0.359030961	isogroup05057	ENSG00000183291	ENST00000331835	RP4-604K5.1.1
isotig19626	4.384	3.665	5.787	5.671	2114	0.272798151	isogroup05058	ENSG00000177354	ENST00000374144	C10orf71
isotig19627	4.498	3.768	5.941	5.82	2056	0.274836552	isogroup05058	ENSG00000177354	ENST00000374144	C10orf71
isotig19628	16.688	6.769	8.776	9.86	2123	0.684019689	isogroup05059	ENSG00000030110	ENST00000374467	BAK1
isotig19630	41.693	39.138	30.06	42.411	4255	0.422719246	isogroup05060	ENSG00000164951	ENST00000396200	PDP1
isotig19631	6.079	5.799	5.722	5.824	4205	0.014428496	isogroup05061	ENSG00000067248	ENST00000251636	DHX29
isotig19632	1.55	5.928	4	3.369	2100	0.692671151	isogroup05062	ENSG00000172901	ENST00000379578	LVRN.1
isotig19634	71.203	22.641	25.106	26.13	2234	0.915335913	isogroup05063	ENSG00000233822	ENST00000449538	HIST1H2BN
isotig19635	82.366	26.183	29.036	30.218	1931	0.917843992	isogroup05063	ENSG00000233822	ENST00000449538	HIST1H2BN
isotig19638	2.92	2.801	3.15	1.968	2093	0.295399038	isogroup05065	ENSG00000076944	ENST00000320400	STXBP2
isotig19640	11.384	7.838	9.025	7.212	4184	0.540345307	isogroup05066	ENSG00000069956	ENST00000261845	MAPK6
isotig19641	12.619	8.292	12.525	8.959	2089	0.614009544	isogroup05067	ENSG00000133612	ENST00000397238	AGAP3
isotig19643	1.821	1.652	1.363	1.046	2419	0.142425039	isogroup05068	ENSG00000164654	ENST00000405785	MIOS
isotig19644	2.086	1.954	1.589	1.261	1900	0.126484181	isogroup05068	ENSG00000164654	ENST00000405785	MIOS
isotig19645	9.351	7.842	9.989	9.43	2063	0.310532051	isogroup05069	ENSG00000205250	ENST00000379378	E2F4
isotig19647	2.387	3.565	2.919	2.162	2050	0.368048771	isogroup05070	ENSG00000197635	ENST00000360534	DPP4
isotig19655	2.973	2.675	2.414	2.39	2042	0.074481476	isogroup05074	ENSG00000162607	ENST00000371146	USP1
isotig19657	12.973	10.042	12.07	11.562	2331	0.357001954	isogroup05075	ENSG00000131508	ENST00000398734	UBE2D2
isotig19658	18.536	13.876	17.004	16.307	1584	0.405557226	isogroup05075	ENSG00000131508	ENST00000398734	UBE2D2
isotig19661	8.493	5.275	6.678	5.816	2194	0.582898099	isogroup05077	ENSG00000128590	ENST00000249356	DNAJB9
isotig19662	10.092	6.281	7.964	6.975	1791	0.598604118	isogroup05077	ENSG00000128590	ENST00000249356	DNAJB9
isotig19663	5.248	3.015	4.137	4.137	4053	0.442830841	isogroup05078	ENSG00000197775	ENST00000354854	AL136419.1
isotig19664	8.495	4.425	4.772	4.339	2043	0.550123995	isogroup05079	ENSG00000174695	ENST00000502346	TMEM167A
isotig19666	15.534	11.151	14.847	8.096	2050	0.63100248	isogroup05080	ENSG00000167460	ENST00000300933	TPM4
isotig19667	15.916	11.424	15.201	8.273	1997	0.632261216	isogroup05080	ENSG00000167460	ENST00000300933	TPM4
isotig19670	2.059	2.595	2.481	1.698	2017	0.060757496	isogroup05082	ENSG00000121957	ENST00000406462	GPSM2
isotig19672	3.187	1.808	2.038	1.451	2206	0.453567671	isogroup05083	ENSG00000145817	ENST00000448443	YIPF5
isotig19673	3.994	2.111	2.555	1.809	1745	0.546122342	isogroup05083	ENSG00000145817	ENST00000448443	YIPF5
isotig19674	1.073	1.312	3.698	1.357	2467	0.441553318	isogroup05084	ENSG00000213121	ENST00000392385	AL590867.1
contig29754	24.728	14.879	14.288	13.158	3413	0.580173969	isogroup05085	ENSG00000061676	ENST00000361354	NCKAP1
isotig19676	4.785	3.576	4.925	3.542	1991	0.413663861	isogroup05086	ENSG00000159495	ENST00000452443	TGM7
isotig19678	2.236	11.298	6.015	4.087	1983	0.80651161	isogroup05087	ENSG00000077274	ENST00000541758	CAPN6
isotig19680	4.878	4.724	6.841	3.676	2911	0.388226122	isogroup05088	ENSG00000131477	ENST00000253796	RAMP2
isotig19681	8.553	9.781	10.207	7.157	976	0.035761253	isogroup05088	ENSG00000131477	ENST00000253796	RAMP2
isotig19682	12.695	37.031	31.753	11.714	2001	0.584448035	isogroup05089	ENSG00000109472	ENST00000402744	CPE
isotig19684	4.375	3.513	4.753	3.601	2232	0.347091756	isogroup05090	ENSG00000157593	ENST00000393812	SLC35B2
isotig19685	3.061	2.843	3.464	2.916	1908	0.183859998	isogroup05090	ENSG00000157593	ENST00000393812	SLC35B2
isotig19686	2.309	2.857	2.416	2.486	1972	0.249671226	isogroup05091	ENSG00000083828	ENST00000449441	ZNF586
isotig19688	22.667	16.397	18.549	15.426	1978	0.444718945	isogroup05092	ENSG00000100353	ENST00000405442	EIF3D
isotig19689	23.4	16.928	19.139	15.925	1916	0.445376494	isogroup05092	ENSG00000100353	ENST00000405442	EIF3D
isotig19690	17.016	10.281	13.688	12.206	3947	0.545784174	isogroup05093	ENSG00000166889	ENST00000300146	PATL1
isotig19693	38.61	16.574	33.865	21.68	1934	0.869514165	isogroup05095	ENSG00000183283	ENST00000412716	DAZAP2
isotig19695	2.794	2.781	2.669	1.624	1930	0.17064327	isogroup05096	ENSG00000133657	ENST00000310773	ATP13A3
isotig19697	13.977	12.85	11.7	16.723	1919	0.27102277	isogroup05097	ENSG00000143499	ENST00000366957	SMYD2
isotig19698	15.292	14.069	12.746	18.249	1750	0.273389945	isogroup05097	ENSG00000143499	ENST00000366957	SMYD2
isotig19699	5.17	3.935	5.733	4.954	3855	0.391861426	isogroup05098	ENSG00000101413	ENST00000373433	RPRD1B
isotig19700	16.379	11.116	13.426	10.366	1935	0.561076877	isogroup05099	ENSG00000134077	ENST00000515662	THUMPD3
isotig19706	6.018	2.404	6.947	3.716	3753	0.817445705	isogroup05102	ENSG00000047617	ENST00000541277	ANO2
isotig19707	1.392	1.709	1.531	1.17	1894	0.022976629	isogroup05103	ENSG00000166130	ENST00000299157	IKBIP
isotig19709	6.915	5.621	6.716	5.971	1886	0.327524987	isogroup05104	ENSG00000100225	ENST00000266087	FBXO7
isotig19711	4.654	4.314	5.472	4.648	1871	0.240597054	isogroup05105	ENSG00000114491	ENST00000232607	UMPS
isotig19712	5.01	4.643	5.911	5.039	1726	0.244467198	isogroup05105	ENSG00000114491	ENST00000232607	UMPS
isotig19713	26.494	23.253	26.952	23.474	1895	0.310269031	isogroup05106	ENSG00000113648	ENST00000312469	H2AFY
isotig19715	1.798	3.326	2.184	3.077	1877	0.561020516	isogroup05107	ENSG00000182481	ENST00000537025	KPNA2
isotig19717	3.502	5.411	3.784	7.87	3718	0.741132487	isogroup05108	ENSG00000178726	ENST00000377103	THBD
isotig19720	4.733	5.368	5.332	6.036	3709	0.247050425	isogroup05110	ENSG00000198900	ENST00000361337	TOP1
isotig19721	1.739	1.631	1.992	1.558	1834	0.199566018	isogroup05111	ENSG00000261283	ENST00000564985	PRKRIP1.1
isotig19722	1.733	1.668	2.031	1.58	1786	0.198420005	isogroup05111	ENSG00000261283	ENST00000564985	PRKRIP1.1
isotig19725	4.125	3.708	3.044	3.629	1835	0.006660029	isogroup05113	ENSG00000197798	ENST00000360194	FAM118B
isotig19726	4.38	3.968	3.239	3.834	1696	0.006660029	isogroup05113	ENSG00000197798	ENST00000360194	FAM118B
isotig19729	3.383	3.016	2.338	2.454	1824	0.17064327	isogroup05115	ENSG00000166133	ENST00000315616	RPUSD2
isotig19731	14.682	41.132	15.673	17.883	1839	0.846791163	isogroup05116	ENSG00000114054	ENST00000251654	PCCB
isotig19733	4.948	4.219	5.693	4.328	1800	0.442295408	isogroup05117	ENSG00000035141	ENST00000037869	FAM136A
isotig19735	13.145	15.976	11.643	11.776	3669	0.255476441	isogroup05118	ENSG00000105887	ENST00000393085	MTPN
isotig19736	9.202	7.639	8.574	8.06	3633	0.242372436	isogroup05119	ENSG00000112276	ENST00000446408	BVES
contig29824	3.963	3.272	4.137	3.364	3281	0.286024273	isogroup05120	ENSG00000088205	ENST00000263239	DDX18

isotig19737	4.36	2.365	3.896	2.434	1775	0.631218532	isogroup05121	ENSG00000136146	ENST00000258648	MED4
isotig19738	4.894	2.614	4.243	2.611	1569	0.653932141	isogroup05121	ENSG00000136146	ENST00000258648	MED4
isotig19739	17.65	11.91	19.834	11.811	1794	0.69864545	isogroup05122	ENSG00000143162	ENST00000370509	CREG1
isotig19740	17.618	11.678	19.177	11.707	1634	0.69192906	isogroup05122	ENSG00000143162	ENST00000370509	CREG1
isotig19741	3.668	2.014	3.784	2.344	3572	0.567370557	isogroup05123	ENSG00000135074	ENST00000394020	ADAM19
isotig19744	5096.099	4749.737	3114.328	5148.872	1784	0.409634027	isogroup05125	ENSG00000159251	ENST00000290378	ACTC1
isotig19974	11.459	15.337	20.523	11.345	1328	0.08409108	isogroup05256	ENSG00000010278	ENST00000382518	CD9
isotig19976	24.854	39.755	33.794	31.777	1366	0.270581273	isogroup05257	ENSG00000219607	ENST00000405617	PPP1R3G
isotig19978	5.726	4.504	5.726	5.964	1327	0.149188397	isogroup05258	ENSG00000064995	ENST00000361288	TAF11
isotig19979	6.124	4.809	6.023	6.301	1231	0.152297663	isogroup05258	ENSG00000064995	ENST00000361288	TAF11
isotig19980	16.542	13.908	18.228	12.904	1322	0.472148118	isogroup05259	ENSG00000100823	ENST00000555414	APEX1
isotig19984	5.011	2.467	3.789	2.327	1323	0.666829488	isogroup05261	ENSG00000176340	ENST00000314133	COX8A
isotig19986	2.861	4.424	2.359	1.887	2646	0.300490343	isogroup05262	ENSG00000122970	ENST00000552912	IFT81
isotig19989	12.279	9.202	14.029	10.3	1328	0.570817991	isogroup05264	ENSG00000129518	ENST00000250454	EAPP
isotig19990	12.998	9.737	14.796	10.916	1253	0.566243331	isogroup05264	ENSG00000129518	ENST00000250454	EAPP
isotig19991	8.551	6.69	9.098	7.854	1441	0.365662809	isogroup05265	ENSG00000103275	ENST00000566587	UBE2I
isotig19992	10.29	8.491	10.659	8.726	1048	0.361388743	isogroup05265	ENSG00000103275	ENST00000566587	UBE2I
isotig19993	35.948	35.232	40.121	38.598	1325	0.11942023	isogroup05266	ENSG00000145337	ENST00000273968	PIGY
isotig19994	37.742	36.975	42.124	40.507	1262	0.119673856	isogroup05266	ENSG00000145337	ENST00000273968	PIGY
isotig19999	8.515	6.13	7.844	6.85	1304	0.444718945	isogroup05269	ENSG00000179168	ENST00000334928	GGN
isotig20001	5.478	6.671	5.781	6.133	1431	0.33882543	isogroup05270	ENSG00000130349	ENST00000443043	C6orf203
isotig20002	6.548	7.843	6.948	7.349	1188	0.337651236	isogroup05270	ENSG00000130349	ENST00000443043	C6orf203
isotig20005	6.97	5.08	8.253	6.691	2593	0.448260314	isogroup05272	ENSG00000166987	ENST00000547545	MBD6
isotig20007	19.31	14.547	24.383	18.903	1298	0.509290223	isogroup05274	ENSG00000115685	ENST00000407025	PPP1R7
isotig20011	2.997	3.656	3.561	2.281	1286	0.22553919	isogroup05276	ENSG00000133119	ENST00000380071	RFC3
isotig20015	32.751	21.217	24.281	16.448	1308	0.638986999	isogroup05278	ENSG00000100612	ENST00000557185	DHRS7
isotig20017	5.61	5.57	5.173	3.45	2563	0.352868791	isogroup05279	ENSG00000069020	ENST00000443808	MAST4
isotig20018	5.728	7.304	11.48	6.578	1290	0.292261592	isogroup05280	ENSG00000176907	ENST00000315792	C8orf4
isotig20020	1.888	2.136	2.534	1.702	1756	0.155763884	isogroup05281	ENSG00000186838	ENST00000423711	SELV.1
isotig20021	1.564	1.759	2.417	1.422	779	0.183258811	isogroup05281	ENSG00000186838	ENST00000423711	SELV.1
isotig20022	2.71	3.052	3.539	2.968	2557	0.027992786	isogroup05283	ENSG00000102805	ENST00000541907	CLN5
isotig20023	3.922	2.871	2.988	1.829	1273	0.524056887	isogroup05284	ENSG00000214700	ENST00000429849	C12orf71
isotig20029	7.816	3.391	5.546	3.058	1252	0.78490644	isogroup05287	ENSG00000113460	ENST00000336767	BRIX1
isotig20031	24.318	18.866	21.698	20.57	1245	0.335594048	isogroup05288	ENSG00000233164	ENST00000447505	C6orf136
isotig20033	1.434	1.653	1.416	1.077	1330	0.080145788	isogroup05289	ENSG00000197498	ENST00000441448	RPF2
isotig20034	1.746	2.011	1.55	1.358	989	0.041209514	isogroup05289	ENSG00000197498	ENST00000441448	RPF2
isotig20035	12.887	10.144	20.895	23.105	1246	0.128964079	isogroup05290	ENSG00000148450	ENST00000376510	MSRB2
isotig20037	3.616	3.685	2.745	2.131	2469	0.089605095	isogroup05291	ENSG00000119139	ENST00000539225	TJP2
isotig20039	28.989	14.044	25.281	21.658	2487	0.722599008	isogroup05293	ENSG00000173039	ENST00000545816	RELA
isotig20040	1197.873	1860.193	1949.615	2347.353	1697	0.635238972	isogroup05294	ENSG00000101470	ENST00000372555	TNNC2
isotig20041	3802.904	5906.579	6191.056	7456.457	532	0.635774404	isogroup05294	ENSG00000101470	ENST00000372555	TNNC2
isotig20042	5.9	5.915	6.348	6.651	2420	0.233335838	isogroup05295	ENSG00000077092	ENST00000538226	RARB
isotig20043	4.937	3.466	5.292	4.921	2411	0.262887954	isogroup05296	ENSG00000117280	ENST00000414729	RAB7L1
contig30179	3.006	3.168	3.571	2.306	631	0.049513414	isogroup05297	ENSG00000147316	ENST00000519480	MCPH1
isotig20044	12.464	7.788	9.874	9.16	2405	0.546648381	isogroup05298	ENSG00000185658	ENST00000341322	BRWD1
isotig20045	22.926	24.737	25.952	25.524	2433	0.021999699	isogroup05299	ENSG00000163399	ENST00000537345	ATP1A1
isotig20046	3.617	2.782	4.012	2.591	1201	0.498290373	isogroup05300	ENSG00000162972	ENST00000392290	C2orf47
isotig20047	3.886	2.988	4.277	2.784	1118	0.509534456	isogroup05300	ENSG00000162972	ENST00000392290	C2orf47
isotig20048	131.365	75.354	97.172	71.933	1215	0.751578117	isogroup05301	ENSG00000147677	ENST00000521861	EIF3H
isotig20050	3.946	3.681	3.478	2.877	2389	0.18463027	isogroup05302	ENSG00000065183	ENST00000349139	WDR3
isotig20051	11.106	8.481	7.995	6.806	1194	0.322499436	isogroup05303	ENSG00000143183	ENST00000367881	TMCO1
isotig20054	1.384	10.421	4.754	7.392	1169	0.89187082	isogroup05305	ENSG00000090659	ENST00000394173	CD209
isotig20057	6.923	4.086	5.599	4.5	1174	0.556474036	isogroup05307	ENSG00000185798	ENST00000332629	WDR53
isotig20059	3.116	4.036	2.637	2.326	2339	0.163955061	isogroup05308	ENSG00000158006	ENST00000374282	PAFAH2
isotig20060	7.118	7.156	6.416	6.942	2354	0.116423687	isogroup05309	ENSG00000116489	ENST00000263168	CAPZA1
isotig20061	2.135	2.547	2.532	2.689	1920	0.17765086	isogroup05310	ENSG00000156253	ENST00000493196	RWDD2B
isotig20065	3.694	3.178	4.137	3.228	2330	0.2416961	isogroup05312	ENSG00000139323	ENST00000549035	POC1B
isotig20067	2.81	2.411	2.905	1.719	2345	0.359650936	isogroup05314	ENSG00000168564	ENST00000504169	CDKN2AIP
isotig20068	2.388	3.143	1.878	1.972	1175	0.382636958	isogroup05315	ENSG00000089248	ENST00000261735	ERP29
isotig20069	2.483	3.291	1.967	2.005	1122	0.388057038	isogroup05315	ENSG00000089248	ENST00000261735	ERP29
isotig20072	28.913	25.066	48.275	29.143	1158	0.713656346	isogroup05317	ENSG00000136897	ENST00000374865	MRPL50
isotig20073	6.157	7.929	6.884	6.51	1085	0.155078154	isogroup05317	ENSG00000136897	ENST00000374865	MRPL50
isotig20076	44.557	42.369	48.796	51.235	1164	0.182610656	isogroup05319	ENSG00000092010	ENST00000206451	PSME1
isotig20078	15.491	14.091	8.173	8.132	1159	0.007420906	isogroup05320	ENSG00000230685	ENST00000457485	CLIC1
isotig20080	11.181	6.384	8.826	6.523	1136	0.673433155	isogroup05321	ENSG00000129460	ENST00000408901	NGDN
isotig20082	6.369	4.505	5.654	4.75	2286	0.454535207	isogroup05322	ENSG00000167173	ENST00000567617	C15orf39
isotig20083	15.551	13.084	18.143	11.846	2300	0.549353724	isogroup05323	ENSG00000122557	ENST00000396081	HERPUD2
isotig20091	2.512	2.632	1.07	1.798	1121	0.216728038	isogroup05328	ENSG00000243943	ENST00000355467	ZNF512
isotig20093	1.993	2.053	2	1.676	2244	0.069775306	isogroup05329	ENSG00000155329	ENST00000324170	ZCCHC10
isotig20094	13.146	8.55	11.004	9.065	2262	0.542280379	isogroup05330	ENSG00000139726	ENST00000280557	DENR
isotig20097	4.499	7.157	9.185	6.461	395	0.020365221	isogroup05332	ENSG00000198933	ENST00000361722	TBKBP1
isotig20100	10.087	13.027	11.565	9.183	1106	0.06621515	isogroup05334	ENSG00000156928	ENST00000466681	C7orf30
isotig20101	10.737	13.88	12.316	9.745	1036	0.066543924	isogroup05334	ENSG00000156928	ENST00000466681	C7orf30
isotig20104	16.48	15.31	15.086	10.058	1104	0.413692042	isogroup05336	ENSG00000106591	ENST00000223324	MRPL32
isotig20106	13.349	10.091	10.658	9.742	1211	0.379715563	isogroup05337	ENSG00000114850	ENST00000265044	SSR3
isotig20107	16.311	11.899	12.638	12.03	843	0.386939205	isogroup05337	ENSG00000114850	ENST00000265044	SSR3
isotig20108	73.191	64.656	88.709	52.157	1134	0.705587285	isogroup05338	ENSG00000151929	ENST00000369085	BAG3
isotig20110	12.216	11.729	13.258	11.173	1110	0.140245735	isogroup05339	ENSG00000120509	ENST00000374454	PDZD11
isotig20112	4.53	9.303	5.401	11.062	1111	0.845607575	isogroup05340	ENSG00000123838	ENST00000367070	C4BPA
isotig20114	4.83	3.02	3.706	2.48	2212	0.545849929	isogroup05341	ENSG00000164329	ENST00000453514	PAPD4
isotig20115	3.029	3.395	3.354	2.84	2136	0.079835801	isogroup05342	ENSG00000131043	ENST00000373932	C20orf4
isotig20116	3770.865	5562.207	5696.58	7265.282	1112	0.61812392	isogroup05343	ENSG00000108515	ENST00000518175	ENO3

isotig20120	6.066	5.906	3.547	2.605	1083	0.0314684	isogroup05345	ENSG00000249751	ENST00000515823	ECSCR
isotig20122	13.543	9.915	10.925	9.048	1075	0.40432667	isogroup05346	ENSG00000188643	ENST00000368705	S100A16
isotig20123	13.636	9.417	10.784	8.477	952	0.45782295	isogroup05346	ENSG00000188643	ENST00000368705	S100A16
isotig20124	5.189	6.65	7.679	5.986	1195	0.047550162	isogroup05347	ENSG00000116752	ENST00000369541	BCAS2
isotig20125	6.376	8.02	9.175	7.241	960	0.049870369	isogroup05347	ENSG00000116752	ENST00000369541	BCAS2
isotig20126	7.142	7.891	9.715	8.518	1071	0.161503344	isogroup05348	ENSG00000100027	ENST00000339468	YPEL1
isotig20127	7.073	7.388	9.161	8.014	912	0.119016307	isogroup05348	ENSG00000100027	ENST00000339468	YPEL1
isotig20128	4.136	4.754	5.728	5.182	2134	0.029937251	isogroup05349	ENSG00000129071	ENST00000249910	MBD4
isotig20129	36.06	19.335	30.746	23.907	1075	0.72223266	isogroup05350	ENSG00000134153	ENST00000256545	C15orf24
isotig20131	23.04	12.636	22.005	14.218	2154	0.792214624	isogroup05351	ENSG00000166848	ENST00000300086	TERF2IP
isotig20136	10.232	6.59	9.073	8.399	2143	0.435654167	isogroup05354	ENSG00000168883	ENST00000409470	USP39
isotig20137	1.496	2.566	2.161	2.579	1068	0.412686932	isogroup05355	ENSG00000123843	ENST00000367076	C4BPB
isotig20139	3.207	1.528	2.956	1.124	2130	0.626972646	isogroup05356	ENSG00000188554	ENST00000542611	NBR1
isotig20142	13.508	16.125	13.012	10.581	1060	0.020947622	isogroup05358	ENSG00000172172	ENST00000306185	MRPL13
isotig20144	5.615	4.621	6.917	5.042	2109	0.40361276	isogroup05359	ENSG00000142230	ENST00000270225	SAE1
isotig20145	11.39	10.287	11.901	6.966	1038	0.504161344	isogroup05360	ENSG00000161249	ENST00000440396	DMKN
isotig20147	6.9	7.4	8.988	6.078	2081	0.213740888	isogroup05361	ENSG00000165060	ENST00000377270	FXN
isotig20148	12.361	11.699	10.033	8.659	1038	0.182216127	isogroup05362	ENSG00000183726	ENST00000374358	TMEM50A
isotig20149	13.255	12.565	10.753	9.268	965	0.183399714	isogroup05362	ENSG00000183726	ENST00000374358	TMEM50A
isotig20150	8.462	8.48	10.274	10.058	1055	0.135069888	isogroup05363	ENSG00000135211	ENST00000257663	TMEM60
isotig20152	11.001	9.448	8.285	7.974	2055	0.323156985	isogroup05364	ENSG00000126062	ENST00000266025	TMEM115
isotig20153	28.021	19.182	29.182	33.58	2064	0.283760427	isogroup05365	ENSG00000099942	ENST00000411769	CRKL
isotig20154	4.322	3.755	8.625	4.185	1019	0.55336477	isogroup05366	ENSG00000243955	ENST00000334575	GSTA1
isotig20155	4.62	4.032	9.203	4.47	949	0.559461186	isogroup05366	ENSG00000243955	ENST00000334575	GSTA1
isotig20156	65.061	53.71	54.985	36.184	993	0.495819869	isogroup05367	ENSG00000166794	ENST00000300026	PPIB
isotig20161	2.38	3.9	2.378	2.225	1972	0.52644285	isogroup05370	ENSG00000115170	ENST00000434821	ACVR1
isotig20162	7.929	7.078	7.331	5.894	995	0.400061997	isogroup05371	ENSG00000198818	ENST00000361731	SFT2D1
isotig20164	2.142	2.465	2.223	2.321	1977	0.224674983	isogroup05372	ENSG00000030066	ENST00000378460	NUP160
isotig20165	32.217	25.92	27.604	24.585	998	0.441900879	isogroup05373	ENSG00000124562	ENST00000374017	SNRPC
isotig20167	3.073	3.412	2.569	2.641	1969	0.161343654	isogroup05374	ENSG00000131115	ENST00000313040	ZNF227
contig30334	7.547	7.635	5.471	6.183	1593	0.221781769	isogroup05375	ENSG00000113583	ENST00000231512	C5orf15
isotig20170	3.27	2.382	3.489	1.867	971	0.445414068	isogroup05377	ENSG00000196531	ENST00000454682	NACA
isotig20171	3.908	2.885	4.05	2.147	782	0.468860374	isogroup05377	ENSG00000196531	ENST00000454682	NACA
isotig20172	2.903	2.708	3.777	2.604	1926	0.292064327	isogroup05378	ENSG00000172992	ENST00000452796	DCAKD
isotig20173	14.392	9.556	13.928	14.418	1958	0.361773878	isogroup05379	ENSG00000117262	ENST00000313835	GPR89A
isotig20174	11.064	10.978	13.064	20.05	1935	0.125347561	isogroup05380	ENSG00000013374	ENST00000568733	NUB1
isotig20179	17.748	14.854	19.276	16.122	979	0.275869843	isogroup05383	ENSG00000123144	ENST00000242784	C19orf43
isotig20181	4.425	3.364	5.391	2.521	959	0.638254302	isogroup05384	ENSG00000162591	ENST00000356575	MEGF6
isotig20182	4.628	3.497	5.598	2.637	917	0.641466897	isogroup05384	ENSG00000162591	ENST00000356575	MEGF6
isotig20185	22.616	17.045	23.122	18.441	1895	0.500234839	isogroup05386	ENSG00000185721	ENST00000331457	DRG1
isotig20186	10.39	9.626	10.67	8.474	1926	0.386093785	isogroup05387	ENSG00000114354	ENST00000443578	TFG
isotig20189	170.867	83.349	161.954	126.066	941	0.736322988	isogroup05389	ENSG00000122367	ENST00000539402	LDB3
isotig20190	153.594	75.152	145.149	112.716	744	0.729738108	isogroup05389	ENSG00000122367	ENST00000539402	LDB3
isotig20191	4.652	2.868	4.557	2.535	936	0.585161945	isogroup05390	ENSG00000132661	ENST00000254998	NXT1
isotig20193	2.065	2.658	3.031	1.814	1876	0.136507102	isogroup05391	ENSG00000069712	ENST00000409154	KIAA1107
isotig20194	3.563	12.555	5.924	7.774	1889	0.579845194	isogroup05392	ENSG00000162772	ENST00000366987	ATF3
contig30370	5.148	1.629	4.381	1.986	1905	0.807037649	isogroup05393	ENSG00000110756	ENST00000537258	HPS5
isotig20195	3.24	2.986	2.276	1.804	1874	0.054116255	isogroup05394	ENSG00000166130	ENST00000342502	IKBIP
isotig20196	1.93	2.168	2.618	1.731	931	0.189618246	isogroup05395	ENSG00000096092	ENST00000211314	TMEM14A
isotig20198	6.212	7.44	5.784	4.618	1842	0.087726385	isogroup05396	ENSG00000196456	ENST00000329630	ZNF775
isotig20199	2.269	4.364	2.557	2.76	1448	0.531205381	isogroup05397	ENSG00000163064	ENST00000295206	EN1
isotig20202	5.212	5.416	4.661	4.115	1866	0.014428496	isogroup05399	ENSG00000058729	ENST00000283109	RIOK2
isotig20205	1.933	2.608	2.35	1.741	1839	0.034718569	isogroup05401	ENSG00000146918	ENST00000409423	NCAPG2
isotig20206	12.71	10.917	8.459	7.406	931	0.206583001	isogroup05402	ENSG00000005243	ENST00000006101	COPZ2
isotig20207	13.033	11.253	8.743	7.563	888	0.195244984	isogroup05402	ENSG00000005243	ENST00000006101	COPZ2
isotig20208	6.185	8.365	1.657	3.025	1850	0.611163298	isogroup05403	ENSG00000108582	ENST00000543464	CPD
isotig20211	5.98	4.643	4.788	5.67	1820	0.006660029	isogroup05405	ENSG00000171067	ENST00000304271	C11orf24
isotig20212	10.191	10.85	11.106	7.997	921	0.275963778	isogroup05406	ENSG00000171204	ENST00000358867	TMEM126B
isotig20214	2.148	3.196	1.763	2.04	1822	0.446353423	isogroup05407	ENSG00000157800	ENST00000326232	SLC37A3
isotig20215	9.564	6.735	9.866	7.81	1823	0.536954235	isogroup05408	ENSG00000163113	ENST00000543330	OTUD7B
isotig20224	6.639	5.232	5.188	6.624	1808	0.061292929	isogroup05414	ENSG00000172594	ENST00000368440	SMPDL3A
isotig20225	5.179	14.956	8.035	14.327	1797	0.871637108	isogroup05415	ENSG00000206422	ENST00000383467	LRRC30
isotig20226	35.721	25.09	23.661	14.816	899	0.654401819	isogroup05416	ENSG00000127920	ENST00000248564	GNG11
isotig20233	3.833	2.666	3.774	2.563	878	0.47427106	isogroup05420	ENSG00000129691	ENST00000517719	ASH2L
isotig20234	4.191	2.915	4.105	2.802	803	0.487403246	isogroup05420	ENSG00000129691	ENST00000517719	ASH2L
isotig20237	12.655	17.929	13.129	14.364	879	0.376549936	isogroup05423	ENSG00000181991	ENST00000325844	MRPS11
isotig20239	20.921	12.675	35.779	20.257	883	0.790561359	isogroup05424	ENSG00000143369	ENST00000369049	ECM1
isotig20241	2.032	3.73	3.081	2.72	1720	0.443150222	isogroup05425	ENSG00000175938	ENST00000318663	ORAI3
isotig20243	2.836	1.751	2.107	1.511	859	0.422794394	isogroup05427	ENSG00000198105	ENST00000395867	ZNF248
isotig20245	2.853	4.078	3.234	2.528	868	0.155500864	isogroup05428	ENSG00000164241	ENST00000296662	C5orf63
isotig20247	2.772	2.878	5.824	1.758	1693	0.634900804	isogroup05429	ENSG00000117525	ENST00000334047	F3
isotig20249	8.045	6.255	14.463	11.339	1698	0.473059292	isogroup05431	ENSG00000204352	ENST00000375419	C9orf129
isotig20254	4.406	4.313	3.689	3.591	1677	0.054877132	isogroup05435	ENSG00000109084	ENST00000226230	TMEM97
isotig20256	24.196	22.328	22.304	18.055	832	0.322076727	isogroup05437	ENSG00000169976	ENST00000367569	SF3B5
isotig20259	3.764	2.245	3.323	2.327	1645	0.494561133	isogroup05440	ENSG00000128739	ENST00000554227	SNRPN
isotig20260	14.606	10.868	12.543	9.521	817	0.472476892	isogroup05441	ENSG00000189171	ENST00000440685	S100A13
isotig20261	14.351	10.617	11.851	9.507	684	0.418792741	isogroup05441	ENSG00000189171	ENST00000440685	S100A13
isotig20262	4.087	2.099	2.534	1.92	816	0.487741414	isogroup05442	ENSG00000134202	ENST00000540225	GSTM3
isotig20264	5.569	4.08	7.486	4.689	1628	0.560654167	isogroup05443	ENSG00000212710	ENST00000391403	CTAGE1
isotig20269	28.259	18.796	29.544	23.03	800	0.566243331	isogroup05446	ENSG00000244057	ENST00000333881	LCE3C
isotig20271	4.813	1.79	3.047	1.073	1606	0.767227775	isogroup05447	ENSG00000204977	ENST00000457662	TRIM13
isotig20272	6.144	6.273	6.247	4.629	1596	0.085274667	isogroup05448	ENSG00000198860	ENST00000361641	TSEN15

isotig20274	51.194	45.076	54.981	56.413	804	0.089219959	isogroup05450	ENSG00000161981	ENST00000383018	SNRNP25
isotig20276	1.927	5.105	4.998	3.9	1596	0.581742692	isogroup05451	ENSG00000168542	ENST00000317840	COL3A1
isotig20277	36.943	34.918	58.069	44.219	809	0.331019388	isogroup05452	ENSG00000174705	ENST00000311601	SH3PXD2B
isotig20278	40.036	37.791	62.859	47.928	745	0.331705118	isogroup05452	ENSG00000174705	ENST00000311601	SH3PXD2B
isotig20279	28.307	23.22	21.309	17.882	805	0.252780491	isogroup05453	ENSG00000167397	ENST00000394975	VKORC1
isotig20282	343.514	322.684	319.572	309.625	827	0.041557075	isogroup05455	ENSG00000213619	ENST00000263774	NDUFS3
isotig20284	2.835	3.426	4.213	4.455	789	0.141889607	isogroup05456	ENSG00000028277	ENST00000531773	POU2F2
isotig20288	1.955	2.197	1.598	1.152	797	0.187795897	isogroup05458	ENSG00000182575	ENST00000513748	NXPH3
isotig20292	6.027	4.041	5.1	3.321	782	0.534793718	isogroup05460	ENSG00000179304	ENST00000509613	FAM156B
isotig20294	257.305	202.751	217.79	258.122	780	0.024319907	isogroup05461	ENSG00000136717	ENST00000409400	BIN1
isotig20295	252.074	200.924	214.015	252.593	680	0.00856692	isogroup05461	ENSG00000136717	ENST00000346226	BIN1
isotig20296	11.935	21.847	12.253	17.785	917	0.7663166	isogroup05462	ENSG00000150779	ENST00000541231	TIMM8B
isotig20297	18.355	39.815	21.815	32.763	507	0.839135417	isogroup05462	ENSG00000150779	ENST00000541231	TIMM8B
isotig20298	2.181	2.881	1.993	1.444	1566	0.17035207	isogroup05463	ENSG00000162627	ENST00000306121	SNX7
isotig20299	4.597	3.843	6.678	4.547	795	0.493668746	isogroup05464	ENSG00000164941	ENST00000523808	INTS8
isotig20300	4.662	4.162	6.493	4.793	647	0.41075186	isogroup05464	ENSG00000164941	ENST00000523808	INTS8
isotig20301	4.305	4.418	4.663	2.741	784	0.221105433	isogroup05465	ENSG00000173207	ENST00000308987	CKS1B
contig30519	1.287	2.299	1.494	1.863	853	0.378043511	isogroup05467	ENSG00000133111	ENST00000255476	RFXAP
isotig20304	7.572	7.596	9.078	5.812	778	0.465055986	isogroup05468	ENSG00000106399	ENST00000396682	RPA3
isotig20305	7.734	7.911	9.232	6.162	723	0.437279252	isogroup05468	ENSG00000106399	ENST00000396682	RPA3
isotig20306	12.01	5.767	15.509	10.837	784	0.732321335	isogroup05469	ENSG00000139219	ENST00000395281	COL2A1
isotig20307	12.518	6.076	16.185	11.344	741	0.734594574	isogroup05469	ENSG00000139219	ENST00000395281	COL2A1
isotig20308	2.998	3.056	3.616	2.704	1532	0.22553919	isogroup05471	ENSG00000157212	ENST00000404141	PAXIP1
isotig20309	2.652	2.29	2.828	2.178	1521	0.20724055	isogroup05472	ENSG00000122687	ENST00000242257	FTSJ2
isotig20315	23.576	24.777	25.86	21.295	759	0.192896596	isogroup05476	ENSG00000141741	ENST00000394231	MIEN1
isotig20317	16.235	27.385	24.449	19.821	759	0.308606373	isogroup05477	ENSG00000183978	ENST00000328434	CCDC56
isotig20319	22.298	17.906	21.956	17.498	1502	0.435175096	isogroup05478	ENSG00000166946	ENST00000300213	CCNDBP1
isotig20320	8.175	3.508	6.84	4.168	1042	0.788419629	isogroup05479	ENSG00000141504	ENST00000380466	SAT2
isotig20321	12.268	5.439	10.329	6.395	665	0.817539641	isogroup05479	ENSG00000141504	ENST00000380466	SAT2
isotig20324	17.855	10.689	13.855	12.098	1510	0.571569475	isogroup05481	ENSG00000183624	ENST00000502878	C3orf37
isotig20325	4.475	7.599	3.966	5.164	1513	0.559874502	isogroup05482	ENSG00000105793	ENST00000222511	GTPBP10
isotig20326	3.117	2.547	2.695	2.027	1480	0.310701135	isogroup05483	ENSG00000137767	ENST00000568606	SQRDL
contig30552	44.399	29.224	53.84	30.332	820	0.768618021	isogroup05484	ENSG00000054793	ENST00000402822	ATP9A
isotig20328	25.483	19.644	22.503	16.625	750	0.561048696	isogroup05486	ENSG00000170043	ENST00000540486	TRAPPC1
isotig20330	15.936	11.986	9.37	8.743	1480	0.358429774	isogroup05487	ENSG00000115661	ENST00000409260	STK16
isotig20331	1.016	1.382	2.957	3.341	1474	0.05275419	isogroup05488	ENSG00000067066	ENST00000431952	SP100
isotig20333	2.654	2.794	2.839	2.059	647	0.230771399	isogroup05489	ENSG00000103264	ENST00000563113	FBXO31
isotig20334	13.986	9.498	12.604	9.092	1465	0.61741001	isogroup05490	ENSG00000071994	ENST00000541970	PDCD2
isotig20337	26.491	25.875	20.453	23.324	729	0.070808597	isogroup05493	ENSG00000127774	ENST00000397133	TMEM93
isotig20338	28.894	28.109	22.354	25.424	667	0.055224694	isogroup05493	ENSG00000127774	ENST00000397133	TMEM93
isotig20339	14.958	11.932	18.815	10.115	1474	0.705136394	isogroup05494	ENSG00000240857	ENST00000381249	RDH14
isotig20342	634.985	474.542	434.498	412.505	753	0.435090554	isogroup05496	ENSG00000083845	ENST00000196551	RPS5
isotig20345	2.947	20.987	11.674	2.578	393	0.433117908	isogroup05497	ENSG00000099256	ENST00000376376	PRTFDC1
isotig20350	9.215	5.753	6.099	6.013	1426	0.54849891	isogroup05500	ENSG00000242259	ENST00000399562	C22orf39
isotig20351	40.237	51.124	43.733	38.568	1490	0.134816262	isogroup05501	ENSG00000197894	ENST00000296412	ADH5
isotig20587	9.108	14.503	10.994	8.9	2138	0.392368678	isogroup05678	ENSG00000172000	ENST00000307635	ZNF556
isotig20588	4.518	10.705	6.122	9.796	1282	0.76943526	isogroup05679	ENSG00000173372	ENST00000402322	C1QA
isotig20589	6.129	6.093	3.322	3.81	1134	0.071052829	isogroup05680	ENSG00000060339	ENST00000543225	CCAR1
isotig20591	54.272	40.83	77.758	101.112	393	0.199340573	isogroup05682	ENSG00000198400	ENST00000497019	NTRK1
isotig20595	4.856	8.122	10.956	9.998	13925	0.146952732	isogroup05686	ENSG00000112651	ENST00000388752	MRPL2
isotig20596	2.438	4.092	5.23	4.846	11959	0.138536109	isogroup05687	ENSG00000181418	ENST00000421952	DDN
isotig20597	6.041	4.143	5.613	5.009	10360	0.304238371	isogroup05688	ENSG00000065526	ENST00000375759	SPEN
isotig20598	9.718	8.51	6.959	9.288	10101	0.079150071	isogroup05689	ENSG00000151320	ENST00000280979	AKAP6
isotig20599	5.738	7.427	4.676	6.502	9895	0.511112572	isogroup05690	ENSG00000010818	ENST00000367604	HIVEP2
isotig20600	28.883	25.929	18.646	21.847	9663	0.008228752	isogroup05691	ENSG00000144357	ENST00000418381	UBR3
isotig20601	16.029	14.64	17.419	15.547	9550	0.301129105	isogroup05692	ENSG00000170624	ENST00000435422	SGCD
isotig20602	20.509	17.477	24.338	17.66	9202	0.411747576	isogroup05693	ENSG00000137076	ENST00000314888	TLN1
isotig20603	27.254	13.744	13.172	10.81	8764	0.707193582	isogroup05694	ENSG00000197081	ENST00000356956	IGF2R
isotig20604	15.936	16.734	20.15	22.925	8647	0.144914331	isogroup05695	ENSG00000198793	ENST00000361445	MTOR
isotig20605	16.1	17.195	12.364	16.121	8611	0.327910122	isogroup05696	ENSG00000124486	ENST00000378308	USP9X
isotig20606	5.097	6.007	3.278	4.559	8446	0.407013226	isogroup05697	ENSG00000166748	ENST00000441037	AGBL1
isotig20607	7.431	5.72	7.018	6.521	8425	0.271924551	isogroup05698	ENSG00000170471	ENST00000397042	RALGAPB
isotig20608	10.454	6.878	9.071	8.544	8136	0.39414406	isogroup05699	ENSG00000116539	ENST00000392403	ASH1L
isotig20609	4.475	4.638	4.181	5.23	8056	0.202806793	isogroup05700	ENSG00000164715	ENST00000297293	LMTK2
isotig20610	4.951	7.009	4.603	4.33	8055	0.324678741	isogroup05701	ENSG00000144535	ENST00000409307	DIS3L2
isotig20611	16.782	11.476	12.403	9.313	7935	0.465469302	isogroup05702	ENSG00000090863	ENST00000422840	GLG1
isotig20612	8.801	9.467	9.93	8.113	7817	0.078708574	isogroup05703	ENSG00000135945	ENST00000258428	REV1
isotig20613	5.938	3.575	5.303	4.024	7806	0.556661907	isogroup05704	ENSG00000224470	ENST00000427980	ATXN1L
isotig20614	12.139	15.371	9.593	10.177	7767	0.543097618	isogroup05705	ENSG00000197694	ENST00000358161	SPTAN1
isotig20615	28.275	26.023	18.823	18.495	7691	0.022356654	isogroup05706	ENSG00000144676	ENST00000356847	GOLGA4
isotig20616	19.1	15.392	19.387	15.341	7568	0.443197189	isogroup05707	ENSG00000067704	ENST00000302637	IARS2
isotig20617	2.297	3.94	5.022	4.637	7493	0.170314496	isogroup05708	ENSG00000260117	ENST00000570246	ZNF828.1
isotig20618	18.577	17.418	16.299	17.848	7453	0.084758022	isogroup05709	ENSG00000110422	ENST00000303296	HIPK3
isotig20619	7.409	4.141	7.06	7.081	7038	0.505110092	isogroup05710	ENSG00000107290	ENST00000224140	SETX
isotig20620	8.416	5.249	6.827	6.314	7024	0.518758924	isogroup05711	ENSG00000065060	ENST00000192788	UHRF1BP1
isotig20621	14.501	9.502	13.253	9.626	6933	0.590243857	isogroup05712	ENSG00000134243	ENST00000256637	SORT1
isotig20622	10.641	9.348	12.353	12.771	6931	0.110646652	isogroup05713	ENSG00000164880	ENST00000404767	INTS1
isotig20623	7.61	6.185	10	6.98	6915	0.513545502	isogroup05714	ENSG00000164916	ENST00000328914	FOXK1
isotig20624	6.37	3.769	6.093	6.965	6904	0.385802585	isogroup05715	ENSG00000124201	ENST00000396105	ZNFX1
isotig20625	8.51	5.468	6.867	6.699	6857	0.429679868	isogroup05716	ENSG00000160218	ENST00000291574	TRAPPC10
isotig20626	19.379	13.578	17.478	15.084	6848	0.463158488	isogroup05717	ENSG00000144028	ENST00000323853	SNRNP200
isotig20627	7.137	7.828	5.408	4.929	6808	0.060381754	isogroup05718	ENSG00000127914	ENST00000356239	AKAP9

isotig20628	12.04	19.75	7.882	10.5	6784	0.649761404	isogroup05719	ENSG00000171714	ENST00000324559	ANO5
isotig20629	11.875	7.464	8.938	7.37	6722	0.609472458	isogroup05720	ENSG00000074603	ENST00000559233	DPP8
isotig20630	9.692	8.731	7.239	8.67	6693	0.033563162	isogroup05721	ENSG00000180628	ENST00000543648	PCGF5
isotig20631	6.692	8.217	6.248	5.813	6685	0.221781769	isogroup05722	ENSG00000175216	ENST00000415402	CKAP5
isotig20632	13.978	10.148	10.027	7.794	6655	0.596828737	isogroup05723	ENSG00000113580	ENST00000504572	NR3C1
isotig20633	5.825	5.538	5.325	4.128	6644	0.173574059	isogroup05724	ENSG00000139645	ENST00000417002	ANKRD52
isotig20634	7.801	6.612	5.85	4.615	6641	0.350172841	isogroup05725	ENSG00000104517	ENST00000521922	UBR5
isotig20635	8.786	4.57	5.468	4.24	6626	0.689280078	isogroup05726	ENSG00000115548	ENST00000409556	KDM3A
isotig20637	11.775	8.758	8.872	7.763	6535	0.385586533	isogroup05728	ENSG00000196914	ENST00000532993	ARHGEF12
isotig20638	4.599	3.661	2.746	2.984	6535	0.029937251	isogroup05729	ENSG00000117523	ENST00000451306	PRRC2C
isotig20639	6.64	3.254	5.732	4.537	6533	0.65160254	isogroup05730	ENSG00000197136	ENST00000355703	PCNXL3
isotig20640	11.672	7.67	9.181	6.334	6514	0.609209439	isogroup05731	ENSG00000137177	ENST00000562486	KIF13A
isotig20641	11.623	8.771	8.695	7.448	6354	0.447997295	isogroup05732	ENSG00000172466	ENST00000399061	ZNF24
isotig20642	2.319	1.596	2.928	6.987	6357	0.278274592	isogroup05733	ENSG00000173821	ENST00000508628	RNF213
isotig20643	16.979	14.958	13.032	13.304	6322	0.0692023	isogroup05734	ENSG00000132463	ENST00000540657	GRSF1
isotig20644	3.946	7.189	9.208	8.26	6331	0.2102277	isogroup05735	ENSG00000168827	ENST00000486715	GFM1
isotig20645	4.014	8.033	7.712	5.405	6321	0.436762606	isogroup05736	ENSG00000069702	ENST00000465892	TGFBR3
isotig20646	6.5	6.871	5.816	5.961	6302	0.126202375	isogroup05737	ENSG00000107779	ENST00000372037	BMPR1A
isotig20647	8.403	4.639	7.218	6.65	6265	0.585904035	isogroup05738	ENSG00000110046	ENST00000421419	ATG2A
isotig20648	13.705	8.547	16.443	8.948	6247	0.747632825	isogroup05739	ENSG00000069275	ENST00000367142	NUCKS1
isotig20649	5.332	3.122	4.046	4.208	6225	0.299494627	isogroup05740	ENSG00000089250	ENST00000338101	NOS1
isotig20650	14.871	12.9	7.132	9.781	6175	0.197593372	isogroup05741	ENSG00000170759	ENST00000302418	KIF5B
isotig20651	7.972	6.633	8.269	9.176	6170	0.131171564	isogroup05742	ENSG00000103184	ENST00000251170	SEC14L5
isotig20652	4.665	5.648	4.221	4.264	6164	0.273803261	isogroup05743	ENSG00000072501	ENST00000322213	SMC1A
isotig20653	12.58	8.126	8.322	6.276	6128	0.602117307	isogroup05744	ENSG00000166398	ENST00000299505	KIAA0355
isotig20654	6.769	5.659	5.348	4.981	6122	0.176298189	isogroup05745	ENSG00000123200	ENST00000378921	ZC3H13
isotig20655	9.258	5.105	7.205	5.363	6107	0.628541369	isogroup05746	ENSG00000093100	ENST00000441493	XXbac-B461K10.4.1
isotig20656	3.739	6.856	4.993	8.387	6083	0.764127903	isogroup05747	ENSG00000064651	ENST00000262461	SLC12A2
isotig20657	2.542	4.405	8.298	9.032	6097	0.530848426	isogroup05748	ENSG00000118898	ENST00000345988	PPL
isotig20658	6.593	5.726	5.91	4.753	6067	0.190219433	isogroup05749	ENSG00000090905	ENST00000450465	TNRC6A
isotig20659	5.355	4.21	4.243	3.39	6049	0.311424438	isogroup05750	ENSG00000151240	ENST00000280886	DIP2C
isotig20660	38.902	23.871	30.155	29.019	6029	0.488906215	isogroup05751	ENSG00000179295	ENST00000351677	PTPN11
isotig20661	14.451	10.125	7.962	15.934	5995	0.281374465	isogroup05752	ENSG00000169047	ENST00000305123	IRS1
isotig20662	19.723	16.652	13.991	14.12	5935	0.15916435	isogroup05753	ENSG00000140299	ENST00000267859	BNIP2
isotig20663	6.56	5.412	4.765	4.475	5933	0.17450402	isogroup05754	ENSG00000055609	ENST00000355193	MLL3
isotig20664	8.376	9.021	7.024	6.231	5925	0.077675284	isogroup05755	ENSG00000077147	ENST00000371142	TM9SF3
isotig20665	5.198	9.829	12.288	11.499	5928	0.260060494	isogroup05756	ENSG00000182199	ENST00000553474	SHMT2
isotig20666	9.006	7.031	6.959	6.528	5861	0.301767867	isogroup05757	ENSG00000151893	ENST00000369151	C10orf46
isotig20667	43.426	31.415	15.617	21.864	5830	0.556962501	isogroup05758	ENSG00000146147	ENST00000514433	MLIP
isotig20668	12.996	17.266	13.941	14.98	5812	0.471067859	isogroup05759	ENSG00000108424	ENST00000290158	KPNB1
isotig20669	8.192	8.787	8.384	8.281	5812	0.134299617	isogroup05760	ENSG00000204120	ENST00000535418	GIGYF2
isotig20670	3.451	3	8.025	5.762	5805	0.474205305	isogroup05761	ENSG00000132326	ENST00000254657	PER2
isotig20671	2.325	14.025	4.049	4.901	5764	0.941093034	isogroup05762	ENSG00000187955	ENST00000297848	COL14A1
isotig20672	3.918	2.505	3.88	3.968	5744	0.167120688	isogroup05763	ENSG00000110274	ENST00000278935	CEP164
isotig20673	11.119	8.884	12.862	13.178	5701	0.123919741	isogroup05764	ENSG00000139990	ENST00000341516	DCAF5
isotig20674	11.998	12.195	9.727	9.368	5661	0.006660029	isogroup05765	ENSG00000100030	ENST00000415911	MAPK1
isotig20675	2.309	4.356	8.211	11.356	5630	0.823607876	isogroup05766	ENSG00000137801	ENST00000260356	THBS1
isotig20676	6.249	4.223	5.758	3.974	5634	0.582832344	isogroup05767	ENSG00000198604	ENST00000382422	BAZ1A
isotig20677	29.979	25.723	31.475	29.655	5609	0.284577666	isogroup05768	ENSG00000152700	ENST00000439578	SAR1B
isotig20678	5.536	10.671	18.944	11.317	5624	0.124849703	isogroup05769	ENSG00000157150	ENST00000287814	TIMP4
isotig20679	8.485	8.009	10.301	9.563	5616	0.079995491	isogroup05770	ENSG00000119638	ENST00000238616	NEK9
isotig20680	11.133	8.101	13.636	8.31	5606	0.62013414	isogroup05771	ENSG00000124181	ENST00000373272	PLCG1
isotig20681	9.081	6.971	4.709	6.254	5584	0.127489291	isogroup05772	ENSG00000170185	ENST00000307017	USP38
isotig20682	10.072	4.443	4.353	6.202	5552	0.49844067	isogroup05773	ENSG00000225614	ENST00000565624	ZNF469
isotig20683	24.55	21.631	22.034	22.638	5535	0.162151499	isogroup05774	ENSG00000124374	ENST00000244221	PAIP2B
isotig20684	13.053	4.085	8.923	7.02	5517	0.836589765	isogroup05775	ENSG00000163781	ENST00000260810	TOPBP1
isotig20685	5.571	6.175	6.91	5.279	5521	0.0314684	isogroup05776	ENSG00000072736	ENST00000346183	NFATC3
isotig20686	6.717	5.383	5.088	4.32	5509	0.388940032	isogroup05777	ENSG00000115760	ENST00000421745	BIRC6
isotig20687	6.415	5.508	4.372	4.697	5495	0.109547607	isogroup05778	ENSG00000152413	ENST00000334082	HOMER1
isotig20688	18.734	19.107	18.048	22.554	5491	0.347627189	isogroup05779	ENSG00000066629	ENST00000262233	EML1
isotig20689	3.974	3.43	5.047	4.129	5433	0.264841813	isogroup05780	ENSG00000126883	ENST00000540899	NUP214
isotig20690	5.023	3.173	6.265	3.938	5423	0.592000451	isogroup05781	ENSG00000029534	ENST00000265709	ANK1
isotig20691	5.2	188.975	2.79	10.12	5400	0.990099196	isogroup05782	ENSG00000099194	ENST00000370355	SCD
isotig20692	5.006	4.514	2.76	3.72	5398	0.065830014	isogroup05783	ENSG00000139697	ENST00000420886	SBNO1
isotig20693	14.412	15.407	13.442	13.468	5389	0.113239273	isogroup05784	ENSG00000007168	ENST00000397195	PAFAH1B1
isotig20694	5.056	2.314	4.004	8.671	5396	0.151104682	isogroup05785	ENSG00000244731	ENST00000428956	C4A
isotig20695	7.837	3.717	6.033	5.635	5387	0.585781919	isogroup05786	ENSG00000048707	ENST00000358136	VPS13D
isotig20696	10.156	3.304	6.246	5.283	5386	0.7431521	isogroup05787	ENSG00000008513	ENST00000521180	ST3GAL1
isotig20697	6.54	6.787	4.825	4.121	5362	0.072048546	isogroup05788	ENSG00000103335	ENST00000301015	PIEZO1
isotig20698	5.445	3.568	5.007	5.087	5351	0.376634478	isogroup05789	ENSG00000257339	ENST00000547603	AC217778.1
isotig20699	6.034	4.292	4.7	5.867	5311	0.003503795	isogroup05790	ENSG00000170153	ENST00000515673	RNF150
isotig20700	9.811	6.863	8.542	9.269	5311	0.163109642	isogroup05791	ENSG00000181222	ENST00000322644	POLR2A
isotig20701	16.279	13.631	14.168	10.316	5295	0.456742692	isogroup05792	ENSG00000117868	ENST00000421679	ESYT2
isotig20702	29.429	36.851	40.472	39.246	5285	0.257383332	isogroup05793	ENSG00000116016	ENST00000263734	EPAS1
isotig20703	19.445	9.699	25.72	18.533	5281	0.739075299	isogroup05794	ENSG00000225556	ENST00000454109	C2CD4D
isotig20704	7.775	12.36	5.407	6.958	5273	0.631256106	isogroup05795	ENSG00000080200	ENST00000182096	CRYBG3
isotig20705	5.705	7.727	10.532	11.547	5279	0.118161494	isogroup05796	ENSG00000100647	ENST00000342745	KIAA0247
isotig20706	10.397	7.641	12.138	10.669	5271	0.277457353	isogroup05797	ENSG00000188636	ENST00000341255	LDOC1L
isotig20707	3.524	6.22	8.174	7.706	5270	0.191788157	isogroup05798	ENSG00000108821	ENST00000225964	COL1A1
isotig20708	24.002	16.728	15.016	10.889	5257	0.588336966	isogroup05799	ENSG00000198300	ENST00000560429	ZIM2
isotig20709	8.49	6.949	7.968	7.988	5248	0.079150071	isogroup05800	ENSG00000151461	ENST00000397053	UPF2
isotig20710	6.857	9.024	7.415	6.323	5254	0.19764034	isogroup05801	ENSG00000168175	ENST00000395468	MAPK1IP1L

isotig20712	8.887	6.112	11.961	8.262	5238	0.542402495	isogroup05803	ENSG00000159842	ENST00000291107	ABR
isotig20713	10.537	10.816	5.328	6.474	5216	0.257646352	isogroup05804	ENSG00000134352	ENST00000381298	IL6ST
isotig20714	9.669	8.372	9.936	8.665	5211	0.246073495	isogroup05805	ENSG00000115365	ENST00000441020	LANCL1
isotig20715	7.995	6.794	7.697	6.071	5203	0.459363493	isogroup05806	ENSG00000129003	ENST00000395898	VPS13C
isotig20716	5.46	6.585	7.092	6.569	5194	0.181802811	isogroup05807	ENSG00000119125	ENST00000238018	GDA
isotig20717	11.577	6.476	5.07	4.642	5194	0.66905576	isogroup05808	ENSG00000141298	ENST00000540801	SSH2
isotig20718	15.653	64.904	4.299	16.807	5180	0.975520403	isogroup05809	ENSG00000072274	ENST00000392396	TFRC
isotig20719	6.193	4.109	5.594	4.076	5179	0.549597956	isogroup05810	ENSG00000260623	ENST00000566027	VPRBP.1
isotig20720	11.253	4.937	7.109	7.042	5182	0.600069512	isogroup05811	ENSG00000160007	ENST00000404338	ARHGAP35
isotig20721	10.409	6.28	7.632	6.176	5180	0.597645976	isogroup05812	ENSG00000181704	ENST00000462683	YIPF6
isotig20722	9.114	10.332	7.431	7.94	5171	0.20195198	isogroup05813	ENSG00000169018	ENST00000306917	FEM1B
isotig20723	8.335	9.379	6.497	7.748	5161	0.213740888	isogroup05814	ENSG00000153339	ENST00000283351	TRAPPC8
isotig20724	10.47	5.013	10.679	9.622	5156	0.624079432	isogroup05815	ENSG00000221843	ENST00000408964	C2orf16
isotig20725	5.966	3.792	3.884	3.911	5135	0.313500413	isogroup05816	ENSG00000125686	ENST00000300651	MED1
isotig20726	8.59	5.801	5.231	5.166	5122	0.372322838	isogroup05817	ENSG00000014824	ENST00000264451	SLC30A9
isotig20727	31.444	23.765	23.989	29.071	5114	0.118095739	isogroup05818	ENSG00000036257	ENST00000264414	CUL3
isotig20728	8.034	4.48	7.439	5.374	5116	0.618048771	isogroup05819	ENSG00000134575	ENST00000256997	ACP2
isotig20729	13.47	9.07	3.605	7.511	5106	0.079150071	isogroup05820	ENSG00000075223	ENST00000544525	SEMA3C
isotig20730	6.254	3.42	3.409	3.036	5103	0.475266777	isogroup05821	ENSG00000090615	ENST00000450791	GOLGA3
isotig20731	12.137	8.334	9.527	7.425	5086	0.518063801	isogroup05822	ENSG00000145833	ENST00000452510	DDX46
isotig20732	11.154	22.473	15.818	17.243	5092	0.512577966	isogroup05823	ENSG00000108821	ENST00000225964	COL1A1
isotig20733	4.958	3.301	5.015	4.988	5063	0.372773728	isogroup05824	ENSG00000135966	ENST00000393359	TGFBRAP1
isotig20734	15.411	16.982	16.455	17.841	5063	0.143965582	isogroup05825	ENSG00000155313	ENST00000400183	USP25
isotig20735	13.021	8.607	7.911	7.605	5060	0.489723454	isogroup05826	ENSG00000164164	ENST00000454497	OTUD4
isotig20736	2.29	4.307	2.297	1.575	5054	0.414443526	isogroup05827	ENSG00000248458	ENST00000502413	AL139147.1
isotig20737	18.121	11.227	11.583	10.559	5042	0.568469602	isogroup05828	ENSG00000139505	ENST00000381801	MTMR6
isotig20738	5.754	4.985	5.184	4.695	5040	0.198326069	isogroup05829	ENSG00000177303	ENST00000321617	CASKIN2
isotig20739	7.209	9.453	6.175	10.731	5036	0.665091681	isogroup05830	ENSG00000172493	ENST00000544085	AFF1
isotig20740	3.97	4.927	3.141	3.603	5026	0.351628842	isogroup05831	ENSG00000109458	ENST00000262995	GAB1
isotig20741	5.624	5.447	4.837	4.372	5028	0.144688886	isogroup05832	ENSG00000164134	ENST00000296543	NAA15
isotig20742	6.094	4.454	2.996	2.976	5017	0.35553656	isogroup05833	ENSG00000100852	ENST00000539826	ARHGAP5
isotig20743	9.397	9.896	10.707	10.114	5020	0.034840685	isogroup05834	ENSG00000175931	ENST00000319380	UBE2O
isotig20744	14.487	14.127	14.684	14.997	5011	0.053674758	isogroup05835	ENSG00000114030	ENST00000344337	KPNA1
isotig20745	6.994	3.72	5.358	4.313	5014	0.625929962	isogroup05836	ENSG00000166912	ENST00000435680	MTMR10
isotig20746	7.045	5.727	8.577	6.378	5012	0.49455174	isogroup05837	ENSG00000127511	ENST00000248054	SIN3B
isotig20747	6.866	3.127	3.178	3.07	5009	0.59252649	isogroup05838	ENSG00000038382	ENST00000537187	TRIO
isotig20748	6.885	7.152	5.741	6.352	5001	0.0314684	isogroup05839	ENSG00000171105	ENST00000302850	INSR
isotig20749	14.407	12.037	11.773	9.808	4978	0.226995191	isogroup05840	ENSG00000169504	ENST00000488683	CLIC4
isotig20751	3.11	5.763	8.652	6.344	4951	0.124671226	isogroup05842	ENSG00000051341	ENST00000543272	POLQ
isotig20752	7.381	6.188	6.783	4.934	4946	0.318920493	isogroup05843	ENSG00000126261	ENST00000246548	UBA2
isotig20753	5.395	8.589	3.475	6.742	4931	0.753531976	isogroup05844	ENSG00000180530	ENST00000400202	NRIP1
isotig20754	15.332	6.597	11.525	9.837	4925	0.750685729	isogroup05845	ENSG00000158467	ENST00000325006	AHCYL2
isotig20755	10.173	3.805	6.302	4.954	4908	0.784727963	isogroup05846	ENSG00000128578	ENST00000249344	FAM40B
isotig20756	11.562	19.748	20.869	9.316	4907	0.099656196	isogroup05847	ENSG00000115461	ENST00000233813	IGFBP5
isotig20757	16.656	12.28	10.145	10.86	4898	0.34723266	isogroup05848	ENSG00000149658	ENST00000370339	YTHDF1
isotig20758	4.024	2.496	4.342	4.972	4903	0.11341775	isogroup05849	ENSG00000155034	ENST00000382368	FBXL18
isotig20759	8.199	4.272	7.456	4.997	4901	0.698908469	isogroup05850	ENSG00000169375	ENST00000394949	SIN3A
isotig20760	7.212	6.158	6.452	5.5	4868	0.246167431	isogroup05851	ENSG00000084652	ENST00000373609	TXLNA
isotig20761	4.28	4.247	3.866	3.438	4858	0.040580146	isogroup05852	ENSG00000138246	ENST00000260818	DNAJC13
isotig20762	7.477	8.015	7.223	6.263	4848	0.049250395	isogroup05853	ENSG00000043093	ENST00000292782	DCUN1D1
isotig20763	18.044	8.782	18.564	13.722	4829	0.780021793	isogroup05854	ENSG00000176871	ENST00000535496	WSB2
isotig20764	4.005	2.522	3.783	3.073	4837	0.402081611	isogroup05855	ENSG00000124782	ENST00000379938	RREB1
isotig20765	5.766	4.483	6.126	4.638	4832	0.452393477	isogroup05856	ENSG00000101191	ENST00000370371	DIDO1
isotig20766	8.988	7.806	7.656	4.72	4833	0.454563388	isogroup05857	ENSG00000115221	ENST00000409872	ITGB6
isotig21087	4.85	5.045	4.093	5.263	3735	0.263761554	isogroup06178	ENSG00000196588	ENST00000407029	MKL1
isotig21088	5.512	4.544	5.69	5.603	3732	0.183540618	isogroup06179	ENSG00000148498	ENST00000374789	PARD3
isotig21089	15.849	13.938	18.89	24.757	3731	0.175377621	isogroup06180	ENSG00000177169	ENST00000321867	ULK1
isotig21090	4.426	4.106	2.76	3.063	3727	0.035704892	isogroup06181	ENSG00000164603	ENST00000297145	C7orf60
isotig21091	3.409	2.615	3.067	2.834	3720	0.270957015	isogroup06182	ENSG00000124275	ENST00000264668	MTRR
isotig21092	6.517	3.858	16.876	5.326	3720	0.906956865	isogroup06183	ENSG00000101280	ENST00000381922	ANGPT4
isotig21093	7.722	4.802	4.569	3.713	3714	0.580145788	isogroup06184	ENSG00000036054	ENST00000394144	TBC1D23
isotig21094	5.906	7.252	5.401	5.219	3706	0.253447434	isogroup06185	ENSG00000117500	ENST00000370282	TMED5
isotig21095	10.002	6.238	6.942	5.063	3692	0.612976253	isogroup06186	ENSG00000177150	ENST00000402563	FAM210A
isotig21096	11.066	25.093	12.149	17.338	3707	0.834485609	isogroup06187	ENSG00000204262	ENST00000374866	COL5A2
isotig21097	7.844	3.496	5.162	3.786	3702	0.705465169	isogroup06188	ENSG00000084112	ENST00000326495	SSH1
isotig21098	8.402	6.269	8.591	6.769	3701	0.405679342	isogroup06189	ENSG00000130723	ENST00000357304	PRRC2B
isotig21099	5.443	5.852	3.953	4.168	3695	0.120115353	isogroup06190	ENSG00000162928	ENST00000295030	PEX13
isotig21100	3.041	3.609	3.456	2.483	3697	0.06991621	isogroup06191	ENSG00000020181	ENST00000412232	GPR124
isotig21101	59.02	61.07	38.005	43.973	3690	0.289631397	isogroup06192	ENSG00000197043	ENST00000354546	ANXA6
isotig21102	8.807	9.764	10.877	10.139	3694	0.021802435	isogroup06193	ENSG00000140718	ENST00000471389	FTO
isotig21103	17.663	11.956	16.291	12.988	3687	0.501465394	isogroup06194	ENSG00000106609	ENST00000341567	C7orf42
isotig21105	7.881	4.889	7.227	6.734	3687	0.379386789	isogroup06196	ENSG00000198911	ENST00000361204	SREBF2
isotig21106	13.759	9.059	8.026	6.064	3681	0.55190877	isogroup06197	ENSG00000148516	ENST00000446923	ZEB1
isotig21107	6.345	5.908	6.073	6.595	3676	0.073692418	isogroup06198	ENSG00000170852	ENST00000304056	KBTBD2
isotig21108	7.971	5.33	5.939	4.681	3678	0.45879988	isogroup06199	ENSG00000213064	ENST00000271375	SFT2D2
isotig21110	4.747	3.757	3.509	4.791	3673	0.006660029	isogroup06201	ENSG00000168040	ENST00000301838	FADD
isotig21111	4.624	3.645	5.055	4.183	3671	0.292064327	isogroup06202	ENSG00000110851	ENST00000228437	PRDM4
isotig21112	18.773	16.412	14.032	12.689	3661	0.114188021	isogroup06203	ENSG00000147065	ENST00000360270	MSN
isotig21113	4.829	7.46	3.709	4.014	3664	0.497444954	isogroup06204	ENSG00000205213	ENST00000389858	LGR4
isotig21114	4.21	5.019	5.116	4.914	3667	0.194296235	isogroup06205	ENSG00000109320	ENST00000226574	NFKB1
isotig21115	3.749	2.711	1.99	2.193	3665	0.219818517	isogroup06206	ENSG00000104219	ENST00000262096	ZDHHC2
isotig21116	6.319	4.77	5.253	3.27	3663	0.433925753	isogroup06207	ENSG00000165494	ENST00000298281	PCF11

isotig21121	3.146	2.169	2.028	2.214	3650	0.18122041	isogroup06212	ENSG00000100888	ENST00000557364	CHD8
isotig21123	6.358	5.259	5.773	5.331	3645	0.250150297	isogroup06214	ENSG00000113597	ENST00000438419	C5orf44
isotig21124	4.076	3.765	3.804	4.091	3645	0.029937251	isogroup06215	ENSG00000102763	ENST00000379310	KIAA0564
isotig21125	11.531	14.631	13.681	11.728	3644	0.242560307	isogroup06216	ENSG00000166340	ENST00000299427	TPP1
isotig21126	3.233	6.032	3.779	4.674	3645	0.490071015	isogroup06217	ENSG00000084774	ENST00000264705	CAD
isotig21127	8.095	6.971	9.708	5.638	3629	0.399179004	isogroup06218	ENSG00000113262	ENST00000319065	GRM6
isotig21128	3.638	3.603	2.974	2.69	3645	0.089605095	isogroup06219	ENSG00000082516	ENST00000285873	GEMIN5
isotig21129	4.871	4.313	4.818	4.175	3641	0.238445931	isogroup06220	ENSG00000073009	ENST00000455588	IKBKG
isotig21130	1.974	2.342	1.525	1.559	3633	0.159821898	isogroup06221	ENSG00000013375	ENST00000506587	PGM3
isotig21131	3.285	3.084	6.162	3.59	3623	0.613934395	isogroup06222	ENSG00000133943	ENST00000518868	C14orf159
isotig21132	3.134	3.897	4.501	4.251	3639	0.076068986	isogroup06223	ENSG00000146376	ENST00000275189	ARHGAP18
isotig21133	4.015	4.817	3.377	3.41	3631	0.20295709	isogroup06224	ENSG00000133103	ENST00000455146	COG6
isotig21134	4.404	3.344	4.21	3.445	3632	0.329892162	isogroup06225	ENSG00000104164	ENST00000220531	PLDN
isotig21135	2.319	3.257	3.683	3.874	3635	0.435672954	isogroup06226	ENSG00000150510	ENST00000322475	FAM124A
isotig21136	8.091	9.284	12.225	8.736	3631	0.152100398	isogroup06227	ENSG00000164924	ENST00000457309	YWHAZ
isotig21137	4.135	2.692	4.923	5.567	3627	0.032651988	isogroup06228	ENSG00000197415	ENST00000392832	VEPH1
isotig21138	6.965	4.536	7.335	6.753	3630	0.414950778	isogroup06229	ENSG00000160799	ENST00000546280	CCDC12
isotig21139	2.758	3.924	2.83	2.616	3628	0.35611896	isogroup06230	ENSG00000115935	ENST00000392546	WIPF1
isotig21140	8.568	8.008	9.612	9.067	3627	0.063810401	isogroup06231	ENSG00000240230	ENST00000344111	COX19
isotig21141	9.746	8.701	7.274	6.342	3625	0.417064327	isogroup06232	ENSG00000167978	ENST00000426305	SRRM2
isotig21142	2.678	5.158	4.173	5.776	3624	0.671385361	isogroup06233	ENSG00000146122	ENST00000398904	DAAM2
isotig21143	11.391	6.737	8.779	6.607	3622	0.646097918	isogroup06234	ENSG00000172845	ENST00000310015	SP3
isotig21144	8.474	7.377	6.412	5.473	3620	0.10313181	isogroup06235	ENSG00000173905	ENST00000470487	GOLIM4
isotig21145	3.371	3.218	2.117	2.585	3620	0.150869843	isogroup06236	ENSG00000140199	ENST00000560611	SLC12A6
isotig21146	5.845	7.202	5.308	3.91	3618	0.150766514	isogroup06237	ENSG00000136636	ENST00000259154	KCTD3
isotig21147	20.092	11.477	15.427	11.753	3617	0.672352897	isogroup06238	ENSG00000165458	ENST00000538751	INPPL1
isotig21148	5.258	4.585	4.177	3.948	3612	0.054877132	isogroup06239	ENSG00000198876	ENST00000361264	DCAF12
isotig21149	3.804	4.755	7.059	3.277	3607	0.29918464	isogroup06240	ENSG00000131016	ENST00000402676	AKAP12
isotig21150	7.509	4.759	7.014	5.089	3612	0.585659803	isogroup06241	ENSG00000062598	ENST00000396391	ELMO2
isotig21151	9.205	9.654	10.313	8.425	3606	0.093634929	isogroup06242	ENSG00000119396	ENST00000373840	RAB14
isotig21152	2.893	15.892	2.695	11.641	3604	0.933080334	isogroup06243	ENSG00000150347	ENST00000309334	ARID5B
isotig21154	63.064	60.34	81.53	76.62	3598	0.023962952	isogroup06245	ENSG00000184007	ENST00000344035	PTP4A2
isotig21155	2.337	4.148	5.44	4.623	3598	0.149658075	isogroup06246	ENSG00000101115	ENST00000395997	SALL4
isotig21156	5.821	6.369	8.753	9.244	3589	0.254302247	isogroup06247	ENSG00000183248	ENST00000539422	AC010336.1
isotig21157	5.881	3.986	5.833	4.551	3596	0.520712783	isogroup06248	ENSG00000196700	ENST00000450537	ZNF512B
isotig21158	8.343	7.968	6.63	6.349	3593	0.048282859	isogroup06249	ENSG00000198752	ENST00000361246	CDC42BPB
isotig21159	3.956	4.692	4.033	4.098	3594	0.185475689	isogroup06250	ENSG00000133422	ENST00000397641	MORC2
isotig21160	3.915	2.327	2.946	2.768	3591	0.418088224	isogroup06251	ENSG00000136715	ENST00000357702	SAP130
isotig21161	5.448	5.102	5.321	4.646	3585	0.160714286	isogroup06252	ENSG00000163512	ENST00000479665	AZI2
isotig21162	7.2	5.985	5.657	5.676	3581	0.179830165	isogroup06253	ENSG00000139567	ENST00000550683	ACVRL1
isotig21163	2.236	2.71	2.562	3.35	3582	0.245829263	isogroup06254	ENSG00000136167	ENST00000398576	LCP1
isotig21164	8.607	1.644	7.372	2.465	3578	0.8993293	isogroup06255	ENSG00000137699	ENST00000475051	TRIM29
isotig21165	4.275	4.25	3.528	4.037	3581	0.11341775	isogroup06256	ENSG00000117481	ENST00000474844	NSUN4
isotig21166	7.171	3.939	4.177	4.407	3576	0.473425641	isogroup06257	ENSG00000215012	ENST00000407472	C22orf29
isotig21167	3.937	3.763	3.759	3.975	3576	0.029937251	isogroup06258	ENSG00000151502	ENST00000281187	VPS26B
isotig21168	9.765	6.822	11.563	8.881	3577	0.535235215	isogroup06259	ENSG00000126107	ENST00000372172	HECTD3
isotig21169	9.169	6.235	7.552	5.728	3572	0.492015481	isogroup06260	ENSG00000117153	ENST00000435533	KLHL12
isotig21170	5.668	5.674	9.362	8.182	3576	0.170699632	isogroup06261	ENSG00000120709	ENST00000434981	FAM53C
isotig21171	3.041	1.838	3.96	2.294	3572	0.581094537	isogroup06262	ENSG00000171467	ENST00000361428	ZNF318
isotig21172	7.606	6.913	6.743	7.234	3571	0.017143233	isogroup06263	ENSG00000168495	ENST00000397802	POLR3D
isotig21173	5.003	3.681	5.711	5.839	3573	0.157961975	isogroup06264	ENSG00000154229	ENST00000413366	PRKCA
isotig21174	7.177	10.098	6.848	9.118	3570	0.654946645	isogroup06265	ENSG00000110917	ENST00000228506	MLEC
isotig21175	3.66	2.661	2.727	1.938	3569	0.411324867	isogroup06266	ENSG00000139517	ENST00000316334	LNX2
isotig21176	1.038	2.464	4.361	2.232	3561	0.010060494	isogroup06267	ENSG00000170962	ENST00000393158	PDGFD
isotig21177	13.615	7.122	7.728	4.238	3560	0.809667844	isogroup06268	ENSG00000137332	ENST00000553261	DDR1
isotig21178	2.234	2.086	3.368	2.555	3558	0.217178928	isogroup06269	ENSG00000069248	ENST00000537506	NUP133
isotig21179	14.766	11.216	16.383	18.642	3557	0.142998046	isogroup06270	ENSG00000125755	ENST00000245934	SYMPK
isotig21180	24.295	19.184	25.443	17.892	3556	0.552810551	isogroup06271	ENSG00000054118	ENST00000469141	THRAP3
isotig21181	2.648	4.517	3.155	5.609	3552	0.608100999	isogroup06272	ENSG00000183044	ENST00000567812	ABAT
isotig21182	9.354	6.096	4.251	4.169	3544	0.42048358	isogroup06273	ENSG00000168283	ENST00000376663	BMI1
isotig21183	18.282	29.804	29.631	25.637	3547	0.391250845	isogroup06274	ENSG00000109906	ENST00000392996	ZBTB16
isotig21184	4.161	3.209	3.384	3.2	3547	0.18463027	isogroup06275	ENSG00000118058	ENST00000534358	MLL
isotig21185	18.556	12.752	14.466	12.028	3542	0.565040956	isogroup06276	ENSG00000170248	ENST00000307296	PDCD6IP
isotig21186	3.854	13.223	2.569	6.38	3544	0.905914181	isogroup06277	ENSG00000117479	ENST00000236137	SLC19A2
isotig21187	46.136	42.673	58.561	53.598	3534	0.183249418	isogroup06278	ENSG00000163655	ENST00000541628	GMPS
isotig21189	7.618	6.059	6.495	4.542	3534	0.478197565	isogroup06280	ENSG00000105176	ENST00000542441	URI1
isotig21190	3.371	3.955	3.555	3.684	3537	0.209814383	isogroup06281	ENSG00000114988	ENST00000377079	LMAN2L
isotig21191	4.269	12.103	6.447	12.69	3537	0.864300744	isogroup06282	ENSG00000075426	ENST00000264716	FOSL2
isotig21192	2.131	1.503	1.542	1.369	3533	0.221950853	isogroup06283	ENSG00000146802	ENST00000454074	TMEM168
isotig21193	21.502	14.305	21.845	19.986	3534	0.498675509	isogroup06284	ENSG00000226589	ENST00000457796	VARS
isotig21194	7.266	5.066	6.3	5.369	3532	0.314909446	isogroup06285	ENSG00000144857	ENST00000273395	BOC
isotig21196	14.442	11.111	2.6	4.777	3529	0.227558804	isogroup06287	ENSG00000198300	ENST00000561033	ZIM2
isotig21197	8.364	4.097	8.546	3.718	3522	0.839032088	isogroup06288	ENSG00000110987	ENST00000538010	BCL7A
isotig21198	16.154	15.867	16.183	12.862	3521	0.273756294	isogroup06289	ENSG00000124207	ENST00000542325	CSE1L
isotig21199	5.713	6.646	3.835	2.964	3520	0.045878109	isogroup06290	ENSG00000117528	ENST00000454898	ABCD3
isotig21200	9.534	7.486	11.428	8.499	3519	0.478996017	isogroup06291	ENSG00000100227	ENST00000252115	POLDIP3
isotig21201	8.022	7.998	7.654	6.904	3515	0.160723679	isogroup06292	ENSG00000154930	ENST00000323482	ACSS1
isotig21202	3.364	1.914	3.735	2.938	3520	0.544290599	isogroup06293	ENSG00000163935	ENST00000394752	SFMBT1
isotig21203	6.489	4.274	4.923	4.404	3518	0.474205305	isogroup06294	ENSG00000157181	ENST00000287859	C1orf27
isotig21204	15.733	12.285	15.251	12.242	3511	0.447048556	isogroup06295	ENSG00000100220	ENST00000216038	C22orf28
isotig21205	13.94	13.064	16.434	15.613	3510	0.112760201	isogroup06296	ENSG00000154945	ENST00000285243	ANKRD40
isotig21206	14.229	6.861	12.479	7.29	3504	0.824838431	isogroup06297	ENSG00000134717	ENST00000313334	BTF3L4

isotig21207	8.017	6.999	5.513	4.433	3500	0.257204855	isogroup06298	ENSG00000133104	ENST00000451493	SPG20
isotig21208	8.173	6.027	7.958	7.562	3499	0.301767867	isogroup06299	ENSG00000140157	ENST00000398014	NIPA2
isotig21209	44.416	2.854	29.467	23.098	3495	0.962623056	isogroup06300	ENSG00000099814	ENST00000556508	KIAA0284
isotig21210	7.152	9.414	9.607	10.195	3496	0.44978207	isogroup06301	ENSG00000185274	ENST00000333538	WBSCR17
isotig21211	10.728	7.567	9.935	7.586	3494	0.58068122	isogroup06302	ENSG00000169762	ENST00000405303	TAPT1
isotig21212	7.244	9.711	7.025	6.678	3493	0.315623356	isogroup06303	ENSG00000005812	ENST00000355619	FBXL3
isotig21213	3.333	4.94	4.941	5.913	3487	0.477220636	isogroup06304	ENSG00000213160	ENST00000392647	KLHL23
isotig21214	6.097	5.086	7.022	5.443	3492	0.336909146	isogroup06305	ENSG00000183495	ENST00000541296	EP400
isotig21216	5.338	5.693	4.411	3.672	3487	0.10625047	isogroup06307	ENSG00000131269	ENST00000253577	ABCB7
isotig21217	8.656	6.481	9.826	7.681	3486	0.460913429	isogroup06308	ENSG00000140553	ENST00000418476	UNC45A
isotig21218	4.282	4.103	4.887	4.421	3484	0.185475689	isogroup06309	ENSG00000132024	ENST00000318003	CC2D1A
isotig21219	16.127	12.866	16.656	13.666	3481	0.391044187	isogroup06310	ENSG00000197157	ENST00000438400	SND1
isotig21220	13.582	11.108	12.17	9.51	3470	0.428825054	isogroup06311	ENSG00000122068	ENST00000241502	FYTTD1
isotig21221	16.438	9.721	15.172	10.559	3460	0.632261216	isogroup06312	ENSG00000143401	ENST00000314136	ANP32E
isotig21222	4.055	4.882	4.283	5.406	3462	0.293398211	isogroup06313	ENSG00000197579	ENST00000379858	TOPORS
isotig21224	39.844	22.175	25.231	16.827	3455	0.79879011	isogroup06315	ENSG00000172348	ENST00000405162	RCAN2
isotig21225	11.86	10.857	13.843	11.368	3452	0.281872323	isogroup06316	ENSG00000169439	ENST00000545117	SDC2
isotig21226	14.005	16.547	14.848	13.908	3451	0.201172315	isogroup06317	ENSG00000111142	ENST00000551840	METAP2
isotig21227	8.084	4.786	11.573	8.466	3456	0.598801383	isogroup06318	ENSG00000173473	ENST00000254480	SMARCC1
isotig21228	2.646	4.785	2.992	2.577	3457	0.558249418	isogroup06319	ENSG00000170004	ENST00000380358	CHD3
isotig21229	4.264	3.137	3.498	2.918	3452	0.354418727	isogroup06320	ENSG00000164252	ENST00000312916	AGGF1
isotig21230	10.321	5.718	8.221	7.153	3452	0.579798226	isogroup06321	ENSG00000070614	ENST00000261797	NDST1
isotig21231	7.375	7.785	8.734	9.11	3450	0.161127602	isogroup06322	ENSG00000130939	ENST00000343090	UBE4B
isotig21232	7.077	5.466	5.773	6.364	3451	0.061292929	isogroup06323	ENSG00000100393	ENST00000263253	EP300
isotig21233	2.99	4.463	3.156	3.398	3446	0.455192756	isogroup06324	ENSG00000106536	ENST00000403058	POU6F2
isotig21234	12.462	7.583	16.84	10.582	3444	0.730705644	isogroup06325	ENSG00000180901	ENST00000375286	KCTD2
isotig21235	17.05	30.934	16.484	12.974	3439	0.618912978	isogroup06326	ENSG00000070159	ENST00000446349	PTPN3
isotig21236	10.427	19.604	10.365	13.302	3440	0.810813857	isogroup06327	ENSG00000134531	ENST00000256951	EMP1
isotig21237	10.936	9.821	9.86	8.385	3444	0.179266551	isogroup06328	ENSG00000058262	ENST00000243253	SEC61A1
isotig21238	2.247	2.49	2.571	2.362	3440	0.010060494	isogroup06329	ENSG00000260117	ENST00000570246	ZNF828.1
isotig21240	2.972	2.7	1.98	2.238	3441	0.006660029	isogroup06331	ENSG00000227345	ENST00000402038	PARG
isotig21241	7.114	5.629	6.223	5.37	3439	0.399827159	isogroup06332	ENSG00000012232	ENST00000220562	EXTL3
isotig21242	4.704	4.721	3.936	3.131	3439	0.151104682	isogroup06333	ENSG00000166734	ENST00000299957	CASC4
isotig21243	2.983	3.17	2.768	3.063	3435	0.097401743	isogroup06334	ENSG00000116198	ENST00000378230	CEP104
isotig21244	11.059	9.717	11.104	11.023	3424	0.139109116	isogroup06335	ENSG00000113282	ENST00000523908	CLINT1
isotig21245	7.951	6.79	6.41	5.259	3431	0.283844969	isogroup06336	ENSG00000136100	ENST00000378060	VPS36
isotig21246	17.098	21.789	18.093	13.673	3427	0.332916886	isogroup06337	ENSG00000142192	ENST00000358918	APP
isotig21247	6.55	9.159	9.645	8.283	3427	0.202618922	isogroup06338	ENSG00000110344	ENST00000252108	UBE4A
isotig21248	15.029	12.784	15.384	12.655	3423	0.286663034	isogroup06339	ENSG00000011454	ENST00000373647	RABGAP1
isotig21249	65.047	20.491	33.137	26.867	3423	0.89803299	isogroup06340	ENSG00000183762	ENST00000400338	KREMEN1
isotig21250	20.431	19.001	18.4	16.209	3432	0.129987976	isogroup06341	ENSG00000112308	ENST00000378119	C6orf62
isotig21251	4.61	2.87	2.493	2.519	3422	0.127818066	isogroup06342	ENSG00000167548	ENST00000301067	MLL2
isotig21252	3.295	4.359	3.602	3.627	3419	0.325242354	isogroup06343	ENSG00000135049	ENST00000376083	AGTPBP1
isotig21253	11.046	10.523	9.684	11.839	3417	0.291472533	isogroup06344	ENSG00000235758	ENST00000452427	AGPAT1
isotig21255	44.385	63.55	52.603	72.286	3407	0.462341249	isogroup06346	ENSG00000163517	ENST00000433119	HDAC11
isotig21256	4.373	3.309	3.178	2.822	3411	0.259562636	isogroup06347	ENSG00000159256	ENST00000400485	MORC3
isotig21257	4.491	4.542	2.891	2.356	3411	0.029937251	isogroup06348	ENSG00000116062	ENST00000544857	MSH6
isotig21258	26.912	37.425	43.933	55.286	3401	0.535009769	isogroup06349	ENSG00000123472	ENST00000371937	ATPAF1
isotig21259	8.018	15.283	4.798	2.501	3410	0.003503795	isogroup06350	ENSG00000151835	ENST00000402364	SACS
isotig21260	4.308	3.213	4.319	3.732	3407	0.331216653	isogroup06351	ENSG00000169446	ENST00000433339	MMGT1
isotig21261	17.655	14.19	18.839	20.475	3407	0.18965582	isogroup06352	ENSG00000163930	ENST00000460680	BAP1
isotig21262	6.383	4.697	4.481	3.696	3404	0.37285827	isogroup06353	ENSG00000047644	ENST00000454666	WWC3
isotig21263	4.743	3.571	4.337	3.773	3406	0.294497257	isogroup06354	ENSG00000167257	ENST00000531452	RNF214
isotig21264	8.966	10.349	7.686	5.443	3397	0.03617457	isogroup06355	ENSG00000163539	ENST00000480013	CLASP2
isotig21265	6.263	9.851	3.793	3.596	3393	0.444127151	isogroup06356	ENSG00000164074	ENST00000437077	C4orf29
isotig21266	41.68	39.197	32.14	35.307	3389	0.040110468	isogroup06357	ENSG00000065675	ENST00000263125	PRKCQ
isotig21268	5.628	5.381	4.68	5.621	3389	0.057742166	isogroup06359	ENSG00000178764	ENST00000314393	ZHX2
isotig21269	3.006	3.326	3.156	2.716	3388	0.135511385	isogroup06360	ENSG00000149428	ENST00000535579	HYOU1
isotig21270	5.407	4.951	4.955	4.825	3387	0.084175622	isogroup06361	ENSG00000113360	ENST00000513349	DROSHA
isotig21271	1.494	2.791	3.662	3.487	3383	0.213036372	isogroup06362	ENSG00000085563	ENST00000543898	ABCB1
isotig21272	4.702	4.351	4.281	3.845	3377	0.163006312	isogroup06363	ENSG00000174197	ENST00000566586	MGA
isotig21273	3.701	2.891	2.602	2.253	3378	0.147910874	isogroup06364	ENSG00000130702	ENST00000252999	LAMA5
isotig21276	31.478	37.464	28.404	28.999	3372	0.164434132	isogroup06367	ENSG00000018625	ENST00000447527	ATP1A2
isotig21277	8.217	10.26	12.549	9.39	3371	0.096039678	isogroup06368	ENSG00000171812	ENST00000481785	COL8A2
isotig21278	3.958	3.128	3.434	3.885	3371	0.161428196	isogroup06369	ENSG00000179409	ENST00000319004	GEMIN4
isotig21279	1.938	2.954	2.718	2.352	3370	0.337566694	isogroup06370	ENSG00000134250	ENST00000256646	NOTCH2
isotig21280	5.076	4.041	6.091	5.091	3368	0.421122342	isogroup06371	ENSG00000166987	ENST00000547545	MBD6
isotig21281	10.182	6.985	10.299	7.249	3360	0.614601338	isogroup06372	ENSG00000012963	ENST00000013070	UBR7
isotig21282	2.154	3.947	4.57	4.715	3366	0.26301007	isogroup06373	ENSG00000121691	ENST00000241052	CAT
isotig21283	7.25	5.248	8.925	5.367	3366	0.595570001	isogroup06374	ENSG00000145860	ENST00000521606	RNF145
isotig21285	4.27	3.866	2.921	2.583	3363	0.051664537	isogroup06376	ENSG00000188157	ENST00000379370	AGRN
isotig21286	4.421	2.69	3.395	2.484	3361	0.556821598	isogroup06377	ENSG00000188177	ENST00000409871	ZC3H6
isotig21287	65.656	46.593	55.433	49.871	3338	0.49600774	isogroup06378	ENSG00000120438	ENST00000321394	TCP1
isotig21289	4.482	2.859	3.418	2.415	3355	0.528180657	isogroup06380	ENSG00000198586	ENST00000521943	TLK1
isotig21290	4.648	2.221	2.077	2.445	3351	0.483326445	isogroup06381	ENSG00000122741	ENST00000377724	DCAF10
isotig21291	5.134	4.621	4.797	3.552	3353	0.239394679	isogroup06382	ENSG00000165102	ENST00000458501	HGSNAT
isotig21292	2.929	2.398	3.045	2.526	3341	0.284126775	isogroup06383	ENSG00000117597	ENST00000491415	DIEXF
isotig21294	10.612	6.112	5.824	4.513	3348	0.443958067	isogroup06385	ENSG00000119699	ENST00000238682	TGFB3
isotig21295	2.662	2.304	3.013	2.201	3346	0.243067558	isogroup06386	ENSG00000038210	ENST00000537420	PI4K2B
isotig21296	11.257	6.671	8.012	6.26	3344	0.648634178	isogroup06387	ENSG00000146872	ENST00000326270	TLK2
isotig21297	5.251	3.911	5.403	4.485	3339	0.388808522	isogroup06388	ENSG00000130699	ENST00000436129	TAF4
isotig21298	21.15	14.631	16.827	12.673	3337	0.562589239	isogroup06389	ENSG00000138071	ENST00000377982	ACTR2

isotig21299	7.958	9.537	8.305	7.74	3335	0.175819118	isogroup06390	ENSG00000075142	ENST00000394641	SRI
isotig21300	8.917	4.666	7.581	5.447	3337	0.693685654	isogroup06391	ENSG00000105127	ENST00000269701	AKAP8
isotig21301	22.604	14.426	15.134	12.652	3337	0.534305253	isogroup06392	ENSG00000095139	ENST00000359415	ARCN1
isotig21302	2.653	1.49	1.816	2.269	3335	0.143242279	isogroup06393	ENSG00000100426	ENST00000216268	ZBED4
isotig21303	2.255	2.304	2.512	2.071	3330	0.112769595	isogroup06394	ENSG00000213121	ENST00000392385	AL590867.1
isotig21304	11.589	9.149	14.119	12.857	3331	0.394454047	isogroup06395	ENSG00000125912	ENST00000246117	NCLN
isotig21305	3.292	2.967	3.549	4.276	3330	0.003503795	isogroup06396	ENSG00000049239	ENST00000377403	H6PD
isotig21306	3.87	2.562	3.645	2.933	3328	0.405031187	isogroup06397	ENSG00000215193	ENST00000399746	PEX26
isotig21308	4.712	3.896	4.989	2.839	3327	0.504546479	isogroup06399	ENSG00000160305	ENST00000417564	DIP2A
isotig21309	5.421	5.47	5.757	4.864	3323	0.148333584	isogroup06400	ENSG00000198908	ENST00000457056	BHLHB9
isotig21310	4.359	3.897	2.915	2.591	3326	0.245885624	isogroup06401	ENSG00000111300	ENST00000261745	NAA25
isotig21311	4.7	3.838	3.517	2.958	3323	0.295070264	isogroup06402	ENSG00000145388	ENST00000388822	METTL14
isotig21312	4.766	7.415	2.231	1.868	3319	0.559245134	isogroup06403	ENSG00000198420	ENST00000479870	FAM115A
isotig21313	1	1.355	2.708	3.891	3314	0.221406027	isogroup06404	ENSG00000110002	ENST00000392744	VWA5A
isotig21314	4.297	2.389	3.716	3.479	3314	0.435672954	isogroup06405	ENSG00000118855	ENST00000415822	MFSD1
isotig21315	4.204	3.662	3.954	2.788	3316	0.350520403	isogroup06406	ENSG00000101474	ENST00000217456	C20orf3
isotig21317	4.101	2.357	3.268	2.184	3311	0.546206884	isogroup06408	ENSG00000124198	ENST00000371917	ARFGEF2
isotig21318	10.26	10.63	11.975	8.24	3309	0.223632299	isogroup06409	ENSG00000100266	ENST00000403744	PACSIN2
isotig21320	4.776	2.351	4.873	3.832	3312	0.570667694	isogroup06411	ENSG00000081913	ENST00000262719	PHLPP1
isotig21321	3.631	5.032	4.407	4.645	3306	0.2660254	isogroup06412	ENSG00000102699	ENST00000381989	PARP4
isotig21322	7.088	7.545	7.771	7.173	3305	0.090431728	isogroup06413	ENSG00000171723	ENST00000478722	GPHN
isotig21324	11.292	8.71	10.558	8.359	3302	0.451200496	isogroup06415	ENSG00000138449	ENST00000261024	SLC40A1
isotig21325	17.457	14.064	20.694	22.888	3305	0.039152326	isogroup06416	ENSG00000138867	ENST00000435822	C22orf13
isotig21326	4.002	3.866	3.744	3.413	3299	0.074284211	isogroup06417	ENSG00000168802	ENST00000306585	CHTF8
isotig21327	10.183	14.408	15.304	19.536	3283	0.520928834	isogroup06418	ENSG00000204007	ENST00000371763	GLT6D1
isotig21328	2.981	1.964	2.162	2.426	3299	0.284098595	isogroup06419	ENSG00000023909	ENST00000370238	GCLM
isotig21329	5.792	4.522	3.339	3.98	3296	0.092404374	isogroup06420	ENSG00000163349	ENST00000443627	HIPK1
isotig21330	25.226	17.264	15.272	16.333	3291	0.48685842	isogroup06421	ENSG00000159140	ENST00000455528	SON
isotig21331	8.842	7.785	8.556	6.462	3291	0.438472233	isogroup06422	ENSG00000143622	ENST00000368322	RIT1
isotig21332	6.928	4.51	7.611	4.842	3292	0.616451867	isogroup06423	ENSG00000104973	ENST00000312881	MED25
isotig21333	5.181	5.598	7.733	6.72	3288	0.048282859	isogroup06424	ENSG00000174165	ENST00000526986	ZDHHC24
isotig21334	35.699	39.225	34.337	41.794	3276	0.343747652	isogroup06425	ENSG00000123091	ENST00000242719	RNF11
isotig21335	2.515	1.8	2.566	2.376	3288	0.211307958	isogroup06426	ENSG00000170322	ENST00000524746	NFRKB
isotig21336	13.344	11.827	16.314	18.601	3288	0.112638085	isogroup06427	ENSG00000198837	ENST00000368646	DENND4B
isotig21337	23.346	40.926	29.66	27.574	3284	0.519970692	isogroup06428	ENSG00000148672	ENST00000277865	GLUD1
isotig21338	3.244	3.887	3.664	3.76	3281	0.439514917	isogroup06429	ENSG00000050405	ENST00000341247	LIMA1
isotig21339	4.048	112.883	2.96	5.793	3281	0.983983993	isogroup06430	ENSG00000169710	ENST00000306749	FASN
isotig21340	5.431	5.38	6.108	5.685	3280	0.045004509	isogroup06431	ENSG00000174306	ENST00000560361	ZHX3
isotig21341	3.257	2.101	5.061	3.174	3280	0.587895469	isogroup06432	ENSG00000043143	ENST00000448712	PHF15
isotig21342	9.842	8.214	10.192	9.162	3279	0.2737469	isogroup06433	ENSG00000109667	ENST00000506583	SLC2A9
isotig21343	17.254	13.321	21.743	24.791	3278	0.085584655	isogroup06434	ENSG00000141720	ENST00000269554	PIP4K2B
isotig21345	13.232	36.397	21.216	22.339	3269	0.796131735	isogroup06436	ENSG00000168827	ENST00000486715	GFM1
isotig21346	12.583	8.248	9.809	8.481	3268	0.452975877	isogroup06437	ENSG00000134910	ENST00000529196	STT3A
isotig21347	4.805	3.474	4.612	3.497	3271	0.486088149	isogroup06438	ENSG00000198382	ENST00000356136	UVRAG
isotig21348	2.972	5.379	7.187	6.45	3273	0.136572856	isogroup06439	ENSG00000113492	ENST00000231420	AGXT2
isotig21349	2.616	6.776	4.03	3.913	3267	0.433042759	isogroup06440	ENSG00000166348	ENST00000428547	USP54
isotig21350	4.42	3.875	5.612	3.07	3267	0.56063538	isogroup06441	ENSG00000204634	ENST00000409318	TBC1D8
isotig21351	11.555	8.832	6.915	5.599	3263	0.389663335	isogroup06442	ENSG00000129083	ENST00000439561	COPB1
isotig21352	4.382	2.415	5.285	3.588	3263	0.667440069	isogroup06443	ENSG00000143643	ENST00000366661	TTC13
isotig21353	6.063	2.968	6.511	3.34	3264	0.780350567	isogroup06444	ENSG00000186472	ENST00000423517	PCLO
isotig21354	4.547	3.465	5.444	3.766	3263	0.52848125	isogroup06445	ENSG00000140598	ENST00000359445	EFTUD1
isotig21355	10.461	32.227	14.409	17.18	3255	0.86400015	isogroup06446	ENSG00000173598	ENST00000415493	NUDT4
isotig21356	33.77	26.954	38.162	29.952	3252	0.432751559	isogroup06447	ENSG00000173692	ENST00000308696	PSMD1
isotig21357	9.59	4.74	6.183	4.462	3261	0.674954911	isogroup06448	ENSG00000182827	ENST00000366812	ACBD3
isotig21362	2.347	1.892	4.873	2.906	3259	0.432159766	isogroup06453	ENSG00000161021	ENST00000376951	MAML1
isotig21363	4.378	5.605	3.935	3.822	3257	0.336561584	isogroup06454	ENSG00000234876	ENST00000457094	NOTCH4
isotig21364	3.628	3.698	4.184	3.448	3253	0.09609604	isogroup06455	ENSG00000119392	ENST00000309971	GLE1
isotig21365	2.686	1.619	2.677	3.41	3256	0.227521229	isogroup06456	ENSG00000196116	ENST00000422139	TDRD7
isotig21366	1.748	2.723	3.464	3.794	3257	0.173724356	isogroup06457	ENSG00000117983	ENST00000529681	MUC5B
isotig21367	18.645	14.756	16.83	15.062	3252	0.368424513	isogroup06458	ENSG00000143799	ENST00000366794	PARP1
isotig21368	57.215	39.752	63.405	59.916	3257	0.332869918	isogroup06459	ENSG00000141385	ENST00000269143	AFG3L2
isotig21369	10.179	6.095	12.978	6.921	3251	0.767331104	isogroup06460	ENSG00000033327	ENST00000361507	GAB2
isotig21370	5.079	6.269	3.944	4.747	3240	0.363596228	isogroup06461	ENSG00000087502	ENST00000360150	ERGIC2
isotig21372	4.059	2.702	3.332	2.323	3247	0.448100624	isogroup06463	ENSG00000107566	ENST00000421367	ERLIN1
isotig21373	2.666	2.084	1.816	1.595	3243	0.229437514	isogroup06464	ENSG00000032219	ENST00000355431	ARID4A
isotig21374	31.999	44.494	39.915	44.366	3241	0.459072293	isogroup06465	ENSG00000244274	ENST00000372720	DBNDD2
isotig21375	3.164	3.62	3.119	3.754	3244	0.363521079	isogroup06466	ENSG00000100221	ENST00000216039	JOSD1
isotig21377	10.737	8.349	9.956	9.44	3242	0.261272263	isogroup06468	ENSG00000016391	ENST00000315251	CHDH
isotig21378	5.833	5.155	8.612	8.438	3242	0.260492598	isogroup06469	ENSG00000136878	ENST00000372429	USP20
isotig21379	6.891	2.898	4.19	3.003	3234	0.683380927	isogroup06470	ENSG00000129595	ENST00000261486	EPB41L4A
isotig21380	5.806	5.393	5.762	4.256	3240	0.358608251	isogroup06471	ENSG00000078747	ENST00000535650	ITCH
isotig21381	5.395	3.485	2.765	3.449	3239	0.177904486	isogroup06472	ENSG00000116539	ENST00000392403	ASH1L
isotig21382	3.674	3.896	6.138	3.426	3234	0.333226873	isogroup06473	ENSG00000197256	ENST00000355150	KANK2
isotig21383	5.118	3.617	1.469	2.605	3231	0.046479297	isogroup06474	ENSG00000138593	ENST00000559471	SECISBP2L
isotig21384	5.573	6.714	5.856	5.054	3229	0.043971218	isogroup06475	ENSG00000178974	ENST00000440021	FBXO34
isotig21385	1.584	3.019	3.678	3.336	3228	0.145271286	isogroup06476	ENSG00000131979	ENST00000491895	GCH1
isotig21386	3.179	5.38	6.359	2.653	3223	0.17548095	isogroup06477	ENSG00000173848	ENST00000380359	NET1
isotig21387	4.825	4.599	5.235	5.173	3224	0.007420906	isogroup06478	ENSG00000162129	ENST00000538039	CLPB
isotig21388	3.466	3.356	2.574	2.592	3221	0.049513414	isogroup06479	ENSG00000148110	ENST00000375344	HIATL1
isotig21389	3.215	3.17	4.083	5.715	3224	0.272084241	isogroup06480	ENSG00000109066	ENST00000335464	TMEM104
isotig21390	7.687	4.121	6.083	3.875	3224	0.694296235	isogroup06481	ENSG00000163625	ENST00000322366	WDFY3
isotig21391	6.818	5.724	9.066	9.081	3222	0.211843391	isogroup06482	ENSG00000106608	ENST00000453200	URGCP

isotig21393	2.455	1.873	4.202	2.287	3221	0.472176298	isogroup06484	ENSG00000144668	ENST00000264741	ITGA9
isotig21394	6.423	1.371	6.443	6.318	3221	0.658046517	isogroup06485	ENSG00000176490	ENST00000323469	DIRAS1
isotig21395	5.061	4.066	3.571	2.562	3216	0.444061396	isogroup06486	ENSG00000117000	ENST00000535839	RLF
isotig21396	4.416	3.293	4.301	3.768	3220	0.293200947	isogroup06487	ENSG00000060491	ENST00000370469	OGFR
isotig21397	5.747	5.211	5.589	4.96	3218	0.199058766	isogroup06488	ENSG00000176095	ENST00000460540	IP6K1
isotig21398	4.732	2.634	2.141	2.666	3216	0.311386864	isogroup06489	ENSG00000048707	ENST00000356315	VPS13D
isotig21399	4.754	4.708	7.589	7.293	3214	0.006660029	isogroup06490	ENSG00000108924	ENST00000226067	HLF
isotig21400	6.224	6.607	5.318	5.271	3215	0.218428271	isogroup06491	ENSG00000100403	ENST00000351589	ZC3H7B
isotig21401	7.822	5.221	6.668	4.865	3212	0.521924551	isogroup06492	ENSG00000090989	ENST00000349598	EXOC1
isotig21402	2.512	3.757	3.162	3.938	3212	0.474055009	isogroup06493	ENSG00000197024	ENST00000540950	ZNF398
isotig21403	5.906	6.056	5.225	5.037	3209	0.032651988	isogroup06494	ENSG00000166224	ENST00000373202	SGPL1
isotig21404	21.167	15.43	17.964	12.192	3206	0.495406553	isogroup06495	ENSG00000102024	ENST00000539310	PLS3
isotig21405	10.694	11.893	10.983	12.861	3201	0.153969715	isogroup06496	ENSG00000108094	ENST00000537177	CUL2
isotig21406	5.61	4.889	5.014	4.22	3204	0.287283009	isogroup06497	ENSG00000184634	ENST00000536756	MED12
isotig21407	12.985	8.691	9.659	5.02	3202	0.677218757	isogroup06498	ENSG00000104522	ENST00000530474	TSTA3
isotig21408	2.191	4.796	5.16	4.136	3199	0.520684602	isogroup06499	ENSG00000165124	ENST00000401783	SVEP1
isotig21409	23.996	30.865	59.751	19.232	3200	0.715290824	isogroup06500	ENSG00000087245	ENST00000219070	MMP2
isotig21410	5.341	3.229	4.015	2.916	3201	0.571447359	isogroup06501	ENSG00000166471	ENST00000528080	TMEM41B
isotig21411	3.238	3.122	2.537	2.733	3195	0.087162771	isogroup06502	ENSG00000141424	ENST00000269187	SLC39A6
isotig21412	5.485	3.953	2.287	2.423	3197	0.239930112	isogroup06503	ENSG00000148400	ENST00000277541	NOTCH1
isotig21413	27.956	19.708	30.9	26.908	3194	0.516899001	isogroup06504	ENSG00000172531	ENST00000376745	PPP1CA
isotig21414	32.45	24.043	41.887	42.301	3196	0.300762756	isogroup06505	ENSG00000142208	ENST00000554848	AKT1
isotig21415	1.57	3.661	4.547	2.419	3192	0.38674194	isogroup06506	ENSG00000154734	ENST00000284984	ADAMTS1
isotig21416	9.676	9.252	11.45	9.612	3193	0.220945743	isogroup06507	ENSG00000119950	ENST00000239007	MXI1
isotig21418	85.555	92.006	96.658	117.834	3187	0.335716164	isogroup06509	ENSG00000159239	ENST00000517883	C2orf81
isotig21419	3.863	1.796	4.492	2.863	3190	0.651771624	isogroup06510	ENSG00000134698	ENST00000373210	EIF2C4
isotig21420	4.332	6.528	4.608	5.804	3190	0.520506125	isogroup06511	ENSG00000136802	ENST00000372599	LRRC8A
isotig21421	4.247	4.944	3.504	3.497	3188	0.133745397	isogroup06512	ENSG00000162433	ENST00000545314	AK4
isotig21422	15.895	9.562	14.476	7.753	3185	0.762127076	isogroup06513	ENSG00000184640	ENST00000423034	9-Sep
isotig21423	8.623	6.399	10.585	8.786	3180	0.492475765	isogroup06514	ENSG00000009780	ENST00000373954	FAM76A
isotig21425	8.682	7.52	12.186	8.964	3179	0.386375592	isogroup06516	ENSG00000184216	ENST00000429936	IRAK1
isotig21426	5.49	3.044	5.541	4.032	3176	0.631688209	isogroup06517	ENSG00000242612	ENST00000219481	DECR2
isotig21427	4.421	3.343	5.311	3.523	3171	0.529693019	isogroup06518	ENSG00000127603	ENST00000545844	MACF1
isotig21429	19.102	10.517	12.118	13.238	3171	0.488145337	isogroup06520	ENSG00000174738	ENST00000396676	NR1D2
isotig21430	4.479	1.951	8.527	3.474	3170	0.790298339	isogroup06521	ENSG00000180730	ENST00000319420	SHISA2
isotig21432	15.245	12.215	13.441	9.885	3157	0.494918088	isogroup06523	ENSG00000197006	ENST00000358154	METTL9
isotig21433	3.783	3.931	3.124	3.448	3162	0.027992786	isogroup06524	ENSG00000136874	ENST00000534052	STX17
isotig21434	8.584	4.709	5.761	4.751	3157	0.596932066	isogroup06525	ENSG00000134265	ENST00000322897	NAPG
isotig21435	19.8	31.629	15.842	14.863	3154	0.457550537	isogroup06526	ENSG00000168575	ENST00000520262	SLC20A2
isotig21436	6.446	4.977	4.131	3.112	3151	0.415692868	isogroup06527	ENSG00000163714	ENST00000473835	U2SURP
isotig21437	3.98	4.645	4.5	3.574	3151	0.006660029	isogroup06528	ENSG00000104756	ENST00000221200	KCTD9
isotig21438	7.101	2.925	5.879	4.655	3150	0.801983918	isogroup06529	ENSG00000154358	ENST00000474237	OBSCN
isotig21439	1.547	2.829	3.518	3.292	3149	0.183136695	isogroup06530	ENSG00000165240	ENST00000350425	ATP7A
isotig21440	2.749	2.856	1.925	1.665	3146	0.014428496	isogroup06531	ENSG00000139291	ENST00000547816	TMEM19
isotig21441	2.297	4.337	2.042	3.898	3147	0.653866386	isogroup06532	ENSG00000171681	ENST00000544627	ATF7IP
isotig21442	3.187	1.537	1.526	1.309	3146	0.445987074	isogroup06533	ENSG00000152217	ENST00000282030	SETBP1
isotig21443	7.678	6.967	9.968	7.92	3144	0.292853385	isogroup06534	ENSG00000259431	ENST00000404535	THTPA
isotig21444	1.701	2.952	4.06	3.5	3147	0.05706583	isogroup06535	ENSG00000180902	ENST00000321264	D2HGDH
isotig21446	11.459	9.632	12.51	11.257	3144	0.209466822	isogroup06537	ENSG00000166598	ENST00000421266	HSP90B1
isotig21447	5.759	4.628	5.565	5.308	3142	0.212012475	isogroup06538	ENSG00000171302	ENST00000392446	CANT1
isotig21448	4.368	4.945	5.044	4.405	3141	0.071052829	isogroup06539	ENSG00000124532	ENST00000378386	MRS2
isotig21449	2.47	3.21	2.59	3.14	3137	0.383510558	isogroup06540	ENSG00000146411	ENST00000275230	SLC2A12
isotig21450	15.128	5.144	8.928	6.416	3140	0.818591719	isogroup06541	ENSG00000150687	ENST00000280258	PRSS23
isotig21451	11.803	3.199	7.158	4.745	3142	0.869655069	isogroup06542	ENSG00000162408	ENST00000377705	NOL9
isotig21452	7.827	7.393	8.095	6.65	3141	0.263404599	isogroup06543	ENSG00000104142	ENST00000220509	VPS18
isotig21453	4.567	19.113	8.974	12.69	3140	0.884928985	isogroup06544	ENSG00000159423	ENST00000375341	ALDH4A1
isotig21454	24.325	19.775	30.455	26.826	3137	0.415636507	isogroup06545	ENSG00000078902	ENST00000382211	TOLLIP
isotig21455	11.105	9.812	11.683	10.296	3131	0.208630796	isogroup06546	ENSG00000163904	ENST00000296257	SENP2
isotig21456	21.503	22.698	27.502	28.9	3128	0.193328699	isogroup06547	ENSG00000221823	ENST00000234310	PPP3R1
isotig21457	12.79	10.442	15.204	14.266	3137	0.283375291	isogroup06548	ENSG00000175221	ENST00000395808	MED16
isotig21458	3.937	3.977	4.317	3.649	3135	0.124520929	isogroup06549	ENSG00000116266	ENST00000370008	STXBP3
isotig21459	5.569	5.471	6.263	4.934	3131	0.151574359	isogroup06550	ENSG00000182197	ENST00000378204	EXT1
isotig21460	4.546	5.342	4.516	3.431	3132	0.194296235	isogroup06551	ENSG00000125844	ENST00000377813	RRBP1
isotig21462	18.133	4.141	10.465	8.247	3130	0.874239122	isogroup06553	ENSG00000156313	ENST00000378505	RPGR
isotig21463	19.485	14.528	21.82	20.811	3126	0.359585181	isogroup06554	ENSG00000206053	ENST00000562684	HN1L
isotig21464	5.695	4.272	5.673	4.616	3128	0.376456001	isogroup06555	ENSG00000158545	ENST00000301011	ZC3H18
isotig21465	12.416	9.279	9.759	7.823	3122	0.472119937	isogroup06556	ENSG00000137055	ENST00000397292	PLAA
isotig21467	14.049	11.727	13.745	10.907	3126	0.373421883	isogroup06558	ENSG00000006715	ENST00000310301	VPS41
isotig21468	7.56	6.676	9.283	6.734	3125	0.385173217	isogroup06559	ENSG00000071205	ENST00000336498	ARHGAP10
isotig21469	5.435	6.595	5.966	6.322	3123	0.190219433	isogroup06560	ENSG00000094880	ENST00000394886	CDC23
isotig21471	4.363	2.937	3.323	2.356	3117	0.480677463	isogroup06562	ENSG00000198689	ENST00000370695	SLC9A6
isotig21473	12.684	3.741	9.253	4.78	3119	0.895543699	isogroup06564	ENSG00000091073	ENST00000446820	DTX2.1
isotig21474	3.788	4.179	4.825	4.383	3111	0.065830014	isogroup06565	ENSG00000100425	ENST00000404034	BRD1
isotig21476	3.918	5.792	2.566	4.112	3109	0.565172466	isogroup06567	ENSG00000114126	ENST00000489671	TFDP2
isotig21477	8.545	9.627	8.222	8.26	3106	0.077675284	isogroup06568	ENSG00000141646	ENST00000398417	SMAD4
isotig21478	3.875	3.865	3.915	3.4	3108	0.122933419	isogroup06569	ENSG00000004766	ENST00000544910	CCDC132
isotig21479	4.017	4.221	3.904	4.121	3107	0.101177951	isogroup06570	ENSG00000119401	ENST00000450136	TRIM32
isotig21480	4.736	3.215	4.081	2.593	3108	0.516354174	isogroup06571	ENSG00000154059	ENST00000284202	IMPACT
isotig21481	2.871	3.093	2.917	2.591	3102	0.038353874	isogroup06572	ENSG00000120802	ENST00000556029	TMPO
isotig21482	2.723	5.294	7.254	6.623	3103	0.211073119	isogroup06573	ENSG00000109171	ENST00000264313	SLAIN2
isotig21483	5.059	3.918	4.498	3.689	3095	0.376756594	isogroup06574	ENSG00000205783	ENST00000381778	AC091435.1
isotig21484	3.319	5.909	1.984	3.251	3099	0.64261291	isogroup06575	ENSG00000215695	ENST00000345034	RSC1A1

isotig21485	8.596	9.434	8.52	6.99	3097	0.10699256	isogroup06576	ENSG00000170955	ENST00000530979	PRKCDBP
isotig21486	6.807	4.792	7.569	6.619	3098	0.44251146	isogroup06577	ENSG00000130311	ENST00000359866	DDA1
isotig21487	3.291	3.795	4.57	4.362	3095	0.090957767	isogroup06578	ENSG00000169122	ENST00000361488	FAM110B
isotig21488	3.825	3.08	2.887	2.516	3096	0.235176975	isogroup06579	ENSG00000168944	ENST00000306481	CEP120
isotig21489	5.835	5.069	5.305	5.709	3094	0.017143233	isogroup06580	ENSG00000175137	ENST00000366472	SH3BP5L
isotig21491	20.191	15.155	19.984	20.283	3086	0.397074848	isogroup06582	ENSG00000118518	ENST00000368314	RNF146
isotig21492	5.542	3.964	3.613	3.236	3092	0.275944991	isogroup06583	ENSG00000141376	ENST00000405070	BCAS3
isotig21493	3.709	2.894	3.035	2.709	3091	0.263846096	isogroup06584	ENSG00000131196	ENST00000542384	NFATC1
isotig21495	3.301	3.023	2.927	2.489	3085	0.228150597	isogroup06586	ENSG00000213121	ENST00000392385	AL590867.1
isotig21496	12.975	11.3	12.051	9.808	3086	0.332935673	isogroup06587	ENSG00000072210	ENST00000395575	ALDH3A2
isotig21497	4.116	3.133	2.411	2.336	3086	0.232809799	isogroup06588	ENSG00000164151	ENST00000296564	KIAA0947
isotig21498	13.024	9.807	10.615	7.365	3072	0.573504546	isogroup06589	ENSG00000171314	ENST00000334828	PGAM1
isotig21499	8.417	2.39	4.805	4.845	3082	0.788983242	isogroup06590	ENSG00000106113	ENST00000348438	CRHR2
isotig21500	3.641	4.063	3.162	3.609	3083	0.25301533	isogroup06591	ENSG00000198182	ENST00000395835	ZNF607
isotig21501	12.079	9.807	11.779	11.658	3082	0.209851958	isogroup06592	ENSG00000090372	ENST00000391910	STRN4
isotig21502	7.247	5.375	5.274	4.185	3081	0.412959345	isogroup06593	ENSG00000108406	ENST00000251241	DHX40
isotig21503	3.175	2.328	2.81	3.436	3082	0.108279477	isogroup06594	ENSG00000114554	ENST00000393409	PLXNA1
isotig21504	6.126	5.097	4.471	4.374	3081	0.209100473	isogroup06595	ENSG00000112367	ENST00000230124	FIG4
isotig21505	5.881	5.074	4.256	5.328	3080	0.094884271	isogroup06596	ENSG00000140992	ENST00000342085	PDPK1
isotig21506	7.308	3.557	3.727	3.114	3077	0.660310363	isogroup06597	ENSG00000136152	ENST00000349995	COG3
isotig21507	2.509	2.577	2.002	1.516	3072	0.003503795	isogroup06598	ENSG00000164144	ENST00000451320	ARFIP1
isotig21508	8.034	5.206	10.172	8.328	3075	0.59895168	isogroup06599	ENSG00000164086	ENST00000495880	DUSP7
isotig21509	5.356	6.345	4.855	5.249	3054	0.273671752	isogroup06600	ENSG00000080819	ENST00000264193	CPOX
isotig21510	26.392	23.829	29.773	23.545	3073	0.338778462	isogroup06601	ENSG00000196365	ENST00000540670	LONP1
isotig21511	3.816	3.705	2.176	2.376	3072	0.06991621	isogroup06602	ENSG00000011258	ENST00000415868	MBTD1
isotig21512	2.069	1.563	3.158	1.976	3070	0.527175547	isogroup06603	ENSG00000126003	ENST00000246229	PLAGL2
isotig21513	30.464	20.95	27.445	24.006	3070	0.506143383	isogroup06604	ENSG00000214706	ENST00000429673	IFRD2
isotig21514	3.74	3.502	3.458	3.053	3065	0.086157661	isogroup06605	ENSG00000038219	ENST00000040738	BOD1L
isotig21515	3.3	2.575	2.125	2.554	3068	0.060757496	isogroup06606	ENSG00000148843	ENST00000369797	PDCD11
isotig21516	2.7	2.232	2.985	3.261	3062	0.017143233	isogroup06607	ENSG00000179630	ENST00000441843	LACC1
isotig21517	4.801	4.625	4.74	3.833	3066	0.045878109	isogroup06608	ENSG00000008710	ENST00000423118	PKD1
isotig21518	4.861	4.043	4.494	3.506	3065	0.348369279	isogroup06609	ENSG00000111581	ENST00000229179	NUP107
isotig21519	4.543	2.05	5.231	5.86	3064	0.362187195	isogroup06610	ENSG00000106683	ENST00000419043	LIMK1
isotig21520	3.513	2.671	3.254	2.959	3064	0.254612234	isogroup06611	ENSG00000198373	ENST00000448661	WWP2
isotig21521	10.331	7.279	7.327	4.993	3062	0.53818479	isogroup06612	ENSG00000137449	ENST00000538197	CPEB2
isotig21522	1.821	2.028	1.153	2.239	3060	0.354681746	isogroup06613	ENSG00000120696	ENST00000379483	KBTBD7
isotig21523	1.515	2.439	3.103	2.971	3059	0.09882017	isogroup06614	ENSG00000167264	ENST00000565263	DUS2L
isotig21524	6.392	3.534	6.386	4.552	3059	0.614920718	isogroup06615	ENSG00000109118	ENST00000332830	PHF12
isotig21525	3.377	2.665	3.123	2.79	3057	0.193676261	isogroup06616	ENSG00000198646	ENST00000374796	NCOA6
isotig21527	2.747	4.02	4.752	3.526	3054	0.097777486	isogroup06618	ENSG00000183248	ENST00000539422	AC010336.1
isotig21528	10.11	17.663	13.229	8.855	3054	0.45594424	isogroup06619	ENSG00000168497	ENST00000304141	SDPR
isotig21530	8.425	6.855	6.189	5.534	3046	0.309583302	isogroup06621	ENSG00000102158	ENST00000358075	MAGT1
isotig21531	3.897	2.697	4.384	2.541	3049	0.556323739	isogroup06622	ENSG00000105501	ENST00000429354	SIGLEC5
isotig21532	3.035	2.524	4.502	2.594	3047	0.485411813	isogroup06623	ENSG00000099991	ENST00000398319	CABIN1
isotig21533	7.228	4.901	6.822	5.499	3045	0.503128053	isogroup06624	ENSG00000143756	ENST00000366862	FBXO28
isotig21534	5.418	4.782	4.979	3.946	3041	0.340441121	isogroup06625	ENSG00000064703	ENST00000369702	DDX20
isotig21536	4.715	5.471	4.973	2.753	3041	0.403349741	isogroup06627	ENSG00000004864	ENST00000416240	SLC25A13
isotig21538	11.224	7.422	10.214	9.525	3039	0.375037574	isogroup06629	ENSG00000068912	ENST00000185150	ERLEC1
isotig21539	2.942	6.115	5.003	7.126	3041	0.651198617	isogroup06630	ENSG00000081760	ENST00000316519	AACS
isotig21540	15.753	17.225	22.17	19.583	3030	0.021999699	isogroup06631	ENSG00000103342	ENST00000434724	GSPT1
isotig21541	7.632	7.055	6.522	5.903	3038	0.077149245	isogroup06632	ENSG00000239305	ENST00000237455	RNF103
isotig21544	22.917	20.675	13.763	16.982	3034	0.101074622	isogroup06635	ENSG00000011638	ENST00000261388	TMEM159
isotig21545	4.082	3.527	3.653	3.403	3033	0.268580446	isogroup06636	ENSG00000183735	ENST00000331710	TBK1
isotig21546	9.732	5.916	9.144	7.29	3031	0.587613662	isogroup06637	ENSG00000123178	ENST00000361840	SPRYD7
isotig21547	22.654	15.39	28.334	18.44	3029	0.703285865	isogroup06638	ENSG00000123064	ENST00000314045	DDX54
isotig21548	14.018	4.653	11.005	11.116	3031	0.721227549	isogroup06639	ENSG00000183248	ENST00000539422	AC010336.1
isotig21549	5.075	5.277	3.79	4.374	3030	0.275663185	isogroup06640	ENSG00000009954	ENST00000404251	BAZ1B
isotig21550	1.372	2.533	3.14	2.994	3028	0.18122041	isogroup06641	ENSG00000116690	ENST00000445192	PRG4
isotig21551	4.051	3.257	3.77	3.535	3027	0.211570978	isogroup06642	ENSG00000051382	ENST00000493568	PIK3CB
isotig21552	6.738	3.595	4.062	3.236	3023	0.587491546	isogroup06643	ENSG00000185760	ENST00000402622	KCNQ5
isotig21553	4.986	4.67	4.946	3.714	3020	0.239394679	isogroup06644	ENSG00000152620	ENST00000381937	NADKD1
isotig21554	8.717	6.059	8.346	6.312	3022	0.566243331	isogroup06645	ENSG00000076201	ENST00000265562	PTPN23
isotig21555	5.392	3.88	4.968	3.741	3022	0.478075449	isogroup06646	ENSG00000165684	ENST00000298532	SNAPC4
isotig21557	4.66	5.23	4.755	3.569	3017	0.081930563	isogroup06648	ENSG00000198642	ENST00000537938	KLHL9
isotig21558	1.925	3.208	3.833	3.773	3020	0.173912227	isogroup06649	ENSG00000117983	ENST00000529681	MUC5B
isotig21559	3.712	6.893	9.288	8.582	3018	0.155782671	isogroup06650	ENSG00000113407	ENST00000541634	TARS
isotig21560	3.264	3.47	2.619	4.17	3017	0.291002856	isogroup06651	ENSG00000152223	ENST00000282041	EPG5
isotig21561	14.577	12.485	17.821	18.25	3014	0.099167731	isogroup06652	ENSG00000153786	ENST00000313732	ZDHHC7
isotig21563	4.936	6.164	7.45	10.287	3014	0.543060044	isogroup06654	ENSG00000142279	ENST00000270288	WTIP
isotig21564	6.325	6.768	7.614	6.615	3012	0.142011723	isogroup06655	ENSG00000130640	ENST00000543663	TUBGCP2
isotig21565	1.585	2.559	1.373	1.338	3012	0.254236492	isogroup06656	ENSG00000137513	ENST00000281038	NARS2
isotig21566	1.704	3.292	4.211	4.259	3012	0.281618697	isogroup06657	ENSG00000100347	ENST00000350028	SAMM50
isotig21567	8.126	12.795	5.009	6.765	3008	0.643815285	isogroup06658	ENSG00000145907	ENST00000394123	G3BP1
isotig21568	5.58	5.819	4.784	4.041	3003	0.027992786	isogroup06659	ENSG00000186522	ENST00000397712	10-Sep
isotig21569	5.88	4.154	5.805	3.865	3006	0.52773916	isogroup06660	ENSG00000050820	ENST00000538440	BCAR1
isotig21570	1.256	1.874	2.881	2.867	3006	0.102201849	isogroup06661	ENSG00000167676	ENST00000301286	PLIN4
isotig21572	4.891	3.412	2.559	1.76	3001	0.432169159	isogroup06663	ENSG00000112419	ENST00000440869	PHACTR2
isotig21573	3.809	4.879	5.803	4.225	3004	0.10625047	isogroup06664	ENSG00000166888	ENST00000556155	STAT6
isotig21574	3.56	3.044	2.75	2.111	3001	0.282849252	isogroup06665	ENSG00000134897	ENST00000257336	BIVM
isotig21575	8.251	6.893	6.255	4.598	2999	0.144989479	isogroup06666	ENSG00000135842	ENST00000367511	FAM129A
isotig21576	4.815	3.764	4.938	3.374	2999	0.404439393	isogroup06667	ENSG00000232045	ENST00000551865	EHMT2
isotig21577	62.302	71.175	104.951	76.767	2992	0.334635906	isogroup06668	ENSG00000167676	ENST00000301286	PLIN4

isotig21578	3.652	1.588	2.68	2.159	2997	0.585556474	isogroup06669	ENSG00000108557	ENST00000395776	RAI1
isotig21579	3.26	5.051	6.262	6.165	2996	0.133529345	isogroup06670	ENSG00000119711	ENST00000553458	ALDH6A1
isotig21580	8.752	5.966	7.242	4.498	2996	0.480292327	isogroup06671	ENSG00000239264	ENST00000379757	TXNDC5
isotig21581	23.173	18.049	30.004	24.166	2994	0.450899902	isogroup06672	ENSG00000101161	ENST00000266079	PRPF6
isotig21582	5.908	3.851	3.859	3.094	2995	0.447367927	isogroup06673	ENSG00000127947	ENST00000248594	PTPN12
isotig21583	8.64	6.147	7.322	5.965	2991	0.479118133	isogroup06674	ENSG00000175220	ENST00000311956	ARHGAP1
isotig21584	4.68	4.142	5.285	5.141	2987	0.120115353	isogroup06675	ENSG00000166167	ENST00000370187	BTRC
isotig21585	9.618	3.433	2.435	2.789	2988	0.772413016	isogroup06676	ENSG00000147274	ENST00000562646	RBMX
isotig21586	7.02	3.549	2.547	2.945	2987	0.546723529	isogroup06677	ENSG00000198624	ENST00000355417	CCDC69
isotig21587	8.604	8.035	7.417	6.525	2985	0.186274141	isogroup06678	ENSG00000198839	ENST00000361822	ZNF277
isotig21588	8.18	4.488	8.812	5.361	2986	0.686339896	isogroup06679	ENSG00000163171	ENST00000295324	CDC42EP3
isotig21589	15.449	16.94	21.128	15.515	2981	0.070141655	isogroup06680	ENSG00000123384	ENST00000243077	LRP1
isotig21590	6.771	5.655	6.552	6.365	2979	0.155078154	isogroup06681	ENSG00000166326	ENST00000299413	TRIM44
isotig21591	4.882	2.103	6.876	4.172	2978	0.752940182	isogroup06682	ENSG00000164244	ENST00000512535	PRRC1
isotig21592	2.091	1.578	2.398	1.87	2980	0.235449388	isogroup06683	ENSG00000083520	ENST00000545453	DIS3
isotig21595	2.702	2.664	3.087	2.089	2979	0.176241828	isogroup06686	ENSG00000152404	ENST00000282251	CWF19L2
isotig21596	72.385	31.358	52.143	51.686	2972	0.682122191	isogroup06687	ENSG00000140416	ENST00000560959	TPM1
isotig21597	39.686	34	32.503	30.116	2966	0.225858571	isogroup06688	ENSG00000149269	ENST00000356341	PAK1
isotig21598	3.082	3.264	2.974	3.281	2968	0.222110543	isogroup06689	ENSG00000144233	ENST00000393001	AMMECR1L
isotig21599	2.568	2.294	1.896	1.959	2971	0.003503795	isogroup06690	ENSG00000001629	ENST00000265742	ANKIB1
isotig21600	10.814	8.297	13.936	11.715	2964	0.344837304	isogroup06691	ENSG00000149474	ENST00000377681	CSRP2BP
isotig21601	2.631	2.706	2.732	2.404	2961	0.035704892	isogroup06692	ENSG00000136169	ENST00000354234	SETDB2
isotig21602	6.888	4.734	7.665	6.928	2963	0.462745172	isogroup06693	ENSG00000007541	ENST00000409527	PIGQ
isotig21603	2.653	5.982	3.906	5.462	2959	0.709711054	isogroup06694	ENSG00000107957	ENST00000540321	SH3PXD2A
isotig21604	8.532	6.126	8.961	7.228	2961	0.467160141	isogroup06695	ENSG00000037474	ENST00000264670	NSUN2
isotig21605	7.183	5.978	7.876	6.449	2961	0.236332381	isogroup06696	ENSG00000111737	ENST00000229340	RAB35
isotig21606	3.742	4.796	4.178	4.338	2957	0.2660254	isogroup06697	ENSG00000131778	ENST00000369258	CHD1L
isotig21607	13.129	8.499	14.321	12.057	2954	0.556981288	isogroup06698	ENSG00000185359	ENST00000329138	HGS
isotig21608	19.584	13.986	20.54	15.15	2945	0.572612159	isogroup06699	ENSG00000110696	ENST00000228136	C11orf58
isotig21609	8.102	12.621	10.317	11.637	2953	0.486576614	isogroup06700	ENSG00000154803	ENST00000285071	FLCN
isotig21610	5.864	4.563	4.633	3.047	2953	0.513752161	isogroup06701	ENSG00000157107	ENST00000512348	FCHO2
isotig21611	1.279	2.392	3.29	2.654	2952	0.050227324	isogroup06702	ENSG00000167468	ENST00000354171	GPX4
isotig21612	21.569	15.045	17.958	17.574	2949	0.384186894	isogroup06703	ENSG00000115806	ENST00000234160	GORASP2
isotig21613	10.605	6.485	8.559	7.057	2947	0.609603968	isogroup06704	ENSG00000160691	ENST00000368445	SHC1
isotig21614	3.299	2.62	2.78	2.1	2942	0.318178402	isogroup06705	ENSG00000148634	ENST00000373700	HERC4
isotig21615	17.577	15.648	22.208	15.69	2945	0.444211693	isogroup06706	ENSG00000099995	ENST00000215793	SF3A1
isotig21616	4.959	4.998	3.965	2.749	2948	0.241151274	isogroup06707	ENSG00000099889	ENST00000406259	ARVCF
isotig21617	4.483	2.605	4.652	3.048	2945	0.62341249	isogroup06708	ENSG00000074590	ENST00000261402	NUAK1
isotig21618	10.434	7.527	4.528	3.729	2936	0.588346359	isogroup06709	ENSG00000114120	ENST00000324194	SLC25A36
isotig21619	1.574	2.767	3.399	3.278	2944	0.142387465	isogroup06710	ENSG00000114270	ENST00000454817	COL7A1
isotig21620	21.159	35.92	31.758	39.972	2942	0.645421583	isogroup06711	ENSG00000145391	ENST00000274031	SETD7
isotig21622	12.864	10.345	15.069	13.712	2942	0.242560307	isogroup06713	ENSG00000105649	ENST00000222256	RAB3A
isotig21623	6.256	5.664	4.351	4.307	2937	0.108533103	isogroup06714	ENSG00000138688	ENST00000438707	KIAA1109
isotig21624	8.569	6.949	7.539	6.124	2939	0.393533479	isogroup06715	ENSG00000103978	ENST00000389834	TMEM87A
isotig21625	1.854	3.736	4.955	4.5	2940	0.197837604	isogroup06716	ENSG00000133226	ENST00000447431	SRRM1
isotig21626	5.373	4.351	5.407	5.041	2940	0.290176223	isogroup06717	ENSG00000127580	ENST00000293883	WDR24
isotig21628	15.704	12.863	13.612	10.957	2930	0.39988352	isogroup06719	ENSG00000169251	ENST00000460469	NMD3
isotig21629	2.237	1.627	2.138	3.239	2937	0.021652138	isogroup06720	ENSG00000168016	ENST00000429976	TRANK1
isotig21630	10.68	7.849	9.314	8.598	2932	0.456676937	isogroup06721	ENSG00000063978	ENST00000506706	RNF4
isotig21631	4.73	1.449	5.476	3.041	2930	0.77215939	isogroup06722	ENSG00000149798	ENST00000544348	CDC42EP2
isotig21632	11.258	7.732	10.56	8.237	2927	0.518063801	isogroup06723	ENSG00000108669	ENST00000446868	CYTH1
isotig21633	4.716	2.511	5.023	3.275	2926	0.659680995	isogroup06724	ENSG00000055483	ENST00000542802	USP36
isotig21634	43.128	34.589	47.503	38.296	2927	0.492785752	isogroup06725	ENSG00000105401	ENST00000222005	CDC37
isotig21635	3.942	3.855	3.07	4.182	2923	0.106607425	isogroup06726	ENSG00000196715	ENST00000360768	VKORC1L1
isotig21636	31.7	28.962	33.787	35.742	2920	0.039847449	isogroup06727	ENSG00000079332	ENST00000431664	SAR1A
isotig21637	20.579	13.063	14.675	13.732	2923	0.513291876	isogroup06728	ENSG00000149658	ENST00000370339	YTHDF1
isotig21638	4.03	3.735	3.532	1.936	2923	0.117494552	isogroup06729	ENSG00000126785	ENST00000316754	RHOJ
isotig21640	1.572	2.678	3.698	3.303	2921	0.152945818	isogroup06731	ENSG00000145020	ENST00000273588	AMT
isotig21641	7.749	6.712	10.257	7.479	2920	0.470166078	isogroup06732	ENSG00000240303	ENST00000264990	ACAD11
isotig21642	3.337	3.291	4.444	3.234	2921	0.25301533	isogroup06733	ENSG00000141564	ENST00000544334	RPTOR
isotig21643	7.608	7.492	6.237	4.609	2919	0.231851657	isogroup06734	ENSG00000096401	ENST00000371477	CDC5L
isotig21644	4.236	4.762	4.569	3.729	2918	0.027992786	isogroup06735	ENSG00000132952	ENST00000255304	USPL1
isotig21645	6.306	4.791	3.383	3.81	2920	0.164171113	isogroup06736	ENSG00000251322	ENST00000262795	SHANK3
isotig21646	5.003	3.183	3.676	3.667	2918	0.357302548	isogroup06737	ENSG00000005238	ENST00000378557	FAM214B
isotig21647	3.651	4.876	5.262	4.814	2916	0.193140828	isogroup06738	ENSG00000029639	ENST00000367166	TFB1M
isotig21648	8.654	8.144	7.691	8.995	2917	0.149423236	isogroup06739	ENSG00000196704	ENST00000392720	AMZ2
isotig21649	9.728	6.776	7.232	6.402	2913	0.483918239	isogroup06740	ENSG00000014123	ENST00000369278	UFL1
isotig21651	8.183	6.225	7.217	6.446	2914	0.411183963	isogroup06742	ENSG00000197622	ENST00000540998	CDC42SE1
isotig21652	3.021	3.056	3.528	2.565	2915	0.205596678	isogroup06743	ENSG00000124571	ENST00000439465	XPO5
isotig21653	5.044	4.435	4.492	2.969	2910	0.269576163	isogroup06744	ENSG00000184743	ENST00000398868	ATL3
isotig21654	10.401	6.297	9.502	8.69	2911	0.522535132	isogroup06745	ENSG00000119777	ENST00000321326	TMEM214
isotig21655	2.885	4.341	3.208	3.589	2907	0.312110168	isogroup06746	ENSG00000168297	ENST00000356151	PXK
isotig21656	7.899	9.114	8.409	7.575	2905	0.120237469	isogroup06747	ENSG00000204178	ENST00000374343	TMEM57
isotig21657	2.369	2.396	2.095	1.694	2908	0.057310062	isogroup06748	ENSG00000137040	ENST00000259569	RANBP6
isotig21660	24.721	22	27.834	27.125	2901	0.178721725	isogroup06751	ENSG00000138107	ENST00000369905	ACTR1A
isotig21661	3.72	2.537	3.643	3.469	2904	0.276208011	isogroup06752	ENSG00000136935	ENST00000373555	GOLGA1
isotig21662	12.145	11.471	13.025	11.097	2899	0.155181483	isogroup06753	ENSG00000004487	ENST00000542151	KDM1A
isotig21663	17.678	13.762	15.866	12.161	2892	0.539124145	isogroup06754	ENSG00000099246	ENST00000356940	RAB18
isotig21664	12.691	12.154	13.743	10.327	2894	0.295013902	isogroup06755	ENSG00000134108	ENST00000438743	ARL8B
isotig21665	23.45	26.022	31.3	23.934	2893	0.193666867	isogroup06756	ENSG00000110958	ENST00000262033	PTGES3
isotig21666	37.47	32.417	40.899	32.284	2893	0.383228752	isogroup06757	ENSG00000069345	ENST00000317089	DNAJA2
isotig21667	3.18	2.446	2.711	2.165	2895	0.362159014	isogroup06758	ENSG00000153107	ENST00000341068	ANAPC1

isotig21668	2.794	4.708	5.581	5.351	2895	0.359068535	isogroup06759	ENSG00000187391	ENST00000419488	MAGI2
isotig21669	1.926	1.599	2.183	2.035	2893	0.117710603	isogroup06760	ENSG00000135482	ENST00000257940	ZC3H10
isotig21670	15.76	11.698	21.024	20.715	2893	0.289950778	isogroup06761	ENSG00000065029	ENST00000373953	ZNF76
isotig21671	18.455	7.858	16.647	11.197	2885	0.815069137	isogroup06762	ENSG00000177885	ENST00000392564	GRB2
isotig21672	15.921	15.906	14.439	11.999	2890	0.173282859	isogroup06763	ENSG00000147416	ENST00000276390	ATP6V1B2
isotig21673	4.279	2.84	2.895	2.852	2889	0.38729616	isogroup06764	ENSG00000260045	ENST00000565101	STAM.1
isotig21674	1.209	2.515	2.881	2.772	2891	0.196813707	isogroup06765	ENSG00000196449	ENST00000373044	YRDC
isotig21675	6.201	5.277	7.504	6.625	2889	0.402814308	isogroup06766	ENSG00000176953	ENST00000320805	NFATC2IP
isotig21676	10.186	9.965	18.676	17.278	2888	0.199913579	isogroup06767	ENSG00000169184	ENST00000302326	MN1
isotig21677	14.876	9.454	9.223	9.335	2885	0.487365672	isogroup06768	ENSG00000013364	ENST00000395353	MVP
isotig21678	10.577	7.305	9.984	7.13	2887	0.601826107	isogroup06769	ENSG00000228333	ENST00000455462	RXRB
isotig21680	25.13	11.289	13.291	9.763	2880	0.790674081	isogroup06771	ENSG00000136240	ENST00000258739	KDELR2
isotig21681	3.975	2.781	3.938	3.069	2874	0.425960021	isogroup06772	ENSG00000144840	ENST00000273375	RABL3
isotig21682	6.444	10.118	11.18	3.822	2882	0.306915533	isogroup06773	ENSG00000131471	ENST00000308423	AOC3
isotig21684	3.832	2.893	2.127	2.493	2882	0.108279477	isogroup06775	ENSG00000173064	ENST00000550722	C12orf51
isotig21685	2.626	4.669	6.157	5.445	2882	0.130373112	isogroup06776	ENSG00000141385	ENST00000537174	AFG3L2
isotig21686	6.495	4.116	3.583	3.613	2881	0.452111671	isogroup06777	ENSG00000124151	ENST00000371998	NCOA3
isotig21687	4.032	3.982	3.901	3.664	2878	0.110299091	isogroup06778	ENSG00000075303	ENST00000341119	SLC25A40
isotig21689	2.533	4.729	6.127	5.041	2878	0.068676261	isogroup06780	ENSG00000151806	ENST00000281543	GUF1
isotig21690	4.399	2.172	4.395	2.532	2874	0.646952732	isogroup06781	ENSG00000132005	ENST00000254325	RFX1
isotig21691	11.396	13.205	14.544	11.618	2870	0.156073871	isogroup06782	ENSG00000152518	ENST00000282388	ZFP36L2
isotig21692	11.941	17.373	20.591	13.487	2875	0.003503795	isogroup06783	ENSG00000162407	ENST00000371250	PPAP2B
isotig21693	34.228	24.282	33.363	34.691	2873	0.285507628	isogroup06784	ENSG00000132612	ENST00000254950	VPS4A
isotig21694	8.186	5.543	6.778	6.785	2874	0.407886826	isogroup06785	ENSG00000135775	ENST00000366669	COG2
isotig21695	2.662	3.253	1.973	1.668	2873	0.126700233	isogroup06786	ENSG00000138442	ENST00000261015	WDR12
isotig21697	3.723	2.911	2.67	2.363	2870	0.121242579	isogroup06788	ENSG00000078687	ENST00000544502	TNRC6C
isotig21698	7.722	7.082	8.981	6.479	2868	0.455577891	isogroup06789	ENSG00000132128	ENST00000343304	LRRC41
isotig21699	6.121	4.068	4.601	3.505	2868	0.450721425	isogroup06790	ENSG00000128050	ENST00000512576	PAICS
isotig21700	7.58	11.677	10.245	11.491	2864	0.504330428	isogroup06791	ENSG00000055147	ENST00000520667	FAM114A2
isotig21701	5.594	3.539	5.461	4.56	2867	0.507486661	isogroup06792	ENSG00000130479	ENST00000544059	MAP1S
isotig21702	5.083	5.915	4.726	3.478	2864	0.054877132	isogroup06793	ENSG00000131724	ENST00000371666	IL13RA1
isotig21703	1.598	3.439	2.413	1.549	2862	0.4533798	isogroup06794	ENSG00000184500	ENST00000407433	PROS1
isotig21704	3.293	2.424	4.95	3.166	2850	0.459551364	isogroup06795	ENSG00000125351	ENST00000345865	UPF3B
isotig21705	3.986	7.844	10.449	9.202	2861	0.10313181	isogroup06796	ENSG00000261272	ENST00000561890	MUC22
isotig21706	1.872	2.29	2.164	7.439	2859	0.64930112	isogroup06797	ENSG00000243414	ENST00000427199	TICAM2
isotig21707	4.315	4.745	3.338	3.859	2860	0.304867739	isogroup06798	ENSG00000104973	ENST00000377077	MED25
isotig21709	6.984	7.346	4.153	4.232	2854	0.17450402	isogroup06800	ENSG00000131378	ENST00000334133	RFTN1
isotig21710	5.748	2.659	5.036	4.154	2856	0.602756068	isogroup06801	ENSG00000135631	ENST00000258098	RAB11FIP5
isotig21711	6.463	4.98	6.274	4.826	2855	0.475501616	isogroup06802	ENSG00000090686	ENST00000308271	USP48
isotig21712	7.343	4.93	1.411	5.155	2853	0.151104682	isogroup06803	ENSG00000152332	ENST00000489294	UHMK1
isotig22088	5.597	4.736	5.698	4.997	2576	0.336307958	isogroup07179	ENSG00000181915	ENST00000373783	ADO
isotig22089	4.634	3.08	3.267	2.58	2577	0.468860374	isogroup07180	ENSG00000234539	ENST00000439240	ATF6B
isotig22090	8.405	5.145	8.618	6.034	2571	0.655049974	isogroup07181	ENSG00000176422	ENST00000338146	SPRYD4
isotig22091	11.012	12.342	13.227	13.726	2577	0.170831142	isogroup07182	ENSG00000172009	ENST00000307741	THOP1
isotig22093	3.065	2.559	2.475	2.609	2574	0.112901105	isogroup07184	ENSG00000104884	ENST00000391945	ERCC2
isotig22094	1.629	2.551	3.623	3.2	2574	0.050227324	isogroup07185	ENSG00000213024	ENST00000422090	NUP62
isotig22095	5.557	3.011	4.837	4.028	2573	0.492447584	isogroup07186	ENSG00000186174	ENST00000526143	BCL9L
isotig22096	6.483	6.527	6.462	5.037	2569	0.255391899	isogroup07187	ENSG00000092439	ENST00000313478	TRPM7
isotig22098	6.204	7.32	6.497	5.385	2569	0.086899752	isogroup07189	ENSG00000101367	ENST00000375571	MAPRE1
isotig22099	1.634	1.409	1.446	1.631	2567	0.048414368	isogroup07190	ENSG00000069974	ENST00000569493	RAB27A
isotig22100	15.953	12.652	21.791	25.738	2565	0.038757797	isogroup07191	ENSG00000196498	ENST00000447011	NCOR2
isotig22101	4.422	6.159	6.133	5.577	2565	0.333039002	isogroup07192	ENSG00000254019	ENST00000518520	RP11-10J21.3.1
isotig22102	2.171	3.878	5.179	4.448	2567	0.097777486	isogroup07193	ENSG00000104859	ENST00000544944	CLASRP
isotig22103	3.812	2.654	6.69	6.175	2567	0.340441121	isogroup07194	ENSG00000135503	ENST00000563546	ACVR1B
isotig22104	4.722	2.803	2.928	3.235	2567	0.366583377	isogroup07195	ENSG00000128159	ENST00000439308	TUBGCP6
isotig22105	3.939	4.757	2.179	1.751	2563	0.166134365	isogroup07196	ENSG00000100504	ENST00000544180	PYGL
isotig22106	4.458	3.296	2.801	3.238	2558	0.15215676	isogroup07197	ENSG00000112305	ENST00000370455	SMAP1
isotig22107	10.632	24.743	16.009	12.259	2564	0.67664575	isogroup07198	ENSG00000134253	ENST00000256649	TRIM45
isotig22108	5.895	4.415	5.162	3.252	2561	0.485580897	isogroup07199	ENSG00000144867	ENST00000466490	SRPRB
isotig22109	15.975	10.486	19.231	18.661	2554	0.392951078	isogroup07200	ENSG00000165424	ENST00000372336	ZCCHC24
isotig22110	2.953	4.031	2.766	2.713	2550	0.287076351	isogroup07201	ENSG00000175387	ENST00000402690	SMAD2
isotig22111	5.746	5.194	8.467	5.426	2558	0.404984219	isogroup07202	ENSG00000167778	ENST00000301463	SPRYD3
isotig22112	3.249	1.757	3.17	1.935	2557	0.575317502	isogroup07203	ENSG00000185238	ENST00000331079	PRMT3
isotig22113	15.568	14.443	22.449	19.639	2558	0.261892237	isogroup07204	ENSG00000238227	ENST00000557985	C9orf69
isotig22114	3.84	3.377	3.932	2.904	2559	0.281036297	isogroup07205	ENSG00000185009	ENST00000372745	AP3M1
isotig22115	10.079	6.025	7.708	5.667	2556	0.57623807	isogroup07206	ENSG00000229006	ENST00000431123	TRIM27
isotig22116	2.624	1.318	2.282	1.479	2557	0.462923649	isogroup07207	ENSG00000171467	ENST00000361428	ZNF318
isotig22117	5.331	3.539	6.793	4.819	2555	0.502188698	isogroup07208	ENSG00000002822	ENST00000406869	MAD1L1
isotig22118	27.804	28.495	27.077	27.907	2547	0.118199068	isogroup07209	ENSG00000172239	ENST00000306846	PAIP1
isotig22119	4.152	4.993	5.365	4.365	2554	0.103761178	isogroup07210	ENSG00000198108	ENST00000305031	CHSY3
isotig22120	3.443	3.834	3.162	2.702	2552	0.038353874	isogroup07211	ENSG00000164941	ENST00000523731	INTS8
isotig22121	1.087	4.546	2.306	4.496	2554	0.739620125	isogroup07212	ENSG00000142178	ENST00000270162	SIK1
isotig22122	12.857	10.867	12.199	11.948	2552	0.226206132	isogroup07213	ENSG00000168003	ENST00000535296	SLC3A2
isotig22123	7.207	7.369	8.219	7.176	2552	0.117353648	isogroup07214	ENSG00000176248	ENST00000323927	ANAPC2
isotig22124	3.085	2.895	4.452	2.993	2551	0.365108589	isogroup07215	ENSG00000120942	ENST00000376810	UBIAD1
isotig22125	10.467	6.668	8.794	5.507	2549	0.66503532	isogroup07216	ENSG00000065268	ENST00000251289	WDR18
isotig22126	6.78	11.922	12.392	23.75	2545	0.794074547	isogroup07217	ENSG00000159403	ENST00000542285	C1R
isotig22127	5.541	6.073	6.761	6.379	2548	0.0314684	isogroup07218	ENSG00000163798	ENST00000326019	SLC4A1AP
isotig22128	4.464	3.554	4.076	3.551	2528	0.280886	isogroup07219	ENSG00000006530	ENST00000355413	AGK
isotig22130	5.273	5.37	4.491	5.498	2545	0.133529345	isogroup07221	ENSG00000149809	ENST00000279263	TM7SF2
isotig22131	27.3	19.012	22.206	19.184	2537	0.512963102	isogroup07222	ENSG00000120705	ENST00000360541	ETF1
isotig22132	6.581	5.987	6.71	5.958	2540	0.206348163	isogroup07223	ENSG00000214517	ENST00000328257	PPME1

isotig22133	4.959	3.251	6.882	4.969	2541	0.60469114	isogroup07224	ENSG00000181523	ENST00000326317	SGSH
isotig22134	2.439	1.5	2.015	3.714	2538	0.028237018	isogroup07225	ENSG00000170542	ENST00000380698	SERPINB9
isotig22135	2.699	2.505	4.242	3.802	2538	0.148117532	isogroup07226	ENSG00000178031	ENST00000380548	ADAMTSL1
isotig22137	1.205	4.097	1.609	2.815	2537	0.720626362	isogroup07228	ENSG00000128918	ENST00000249750	ALDH1A2
isotig22138	4.873	4.029	3.333	3.15	2537	0.135426843	isogroup07229	ENSG00000169299	ENST00000381967	PGM2
isotig22139	7.374	5.128	6.417	4.054	2535	0.546422935	isogroup07230	ENSG00000143776	ENST00000535525	CDC42BPA
isotig22140	5.426	9.337	8.431	2.984	2536	0.543961825	isogroup07231	ENSG00000106624	ENST00000223357	AEBP1
isotig22141	50.152	42.743	69.734	56.131	2529	0.34712933	isogroup07232	ENSG00000127124	ENST00000372583	HIVEP3
isotig22143	6.105	5.706	4.273	4.333	2532	0.057742166	isogroup07234	ENSG00000011198	ENST00000458276	ABHD5
isotig22144	4.758	6.949	7.872	6.43	2531	0.209138048	isogroup07235	ENSG00000180902	ENST00000321264	D2HGDH
isotig22145	22.677	15.372	25.162	23.643	2531	0.470109717	isogroup07236	ENSG00000068308	ENST00000455452	OTUD5
isotig22146	37.375	22.436	21.341	17.084	2529	0.713280604	isogroup07237	ENSG00000131653	ENST00000326181	TRAF7
isotig22148	17.034	6.787	7.625	3.082	2525	0.896623957	isogroup07239	ENSG00000124701	ENST00000426505	APOBEC2
isotig22149	8.922	7.033	4.051	2.66	2525	0.417590366	isogroup07240	ENSG00000010319	ENST00000231721	SEMA3G
isotig22150	15.095	12.011	17.184	11.739	2523	0.538109642	isogroup07241	ENSG00000107937	ENST00000360803	GTPBP4
isotig22151	70.977	22.731	91.539	50.917	2518	0.937438942	isogroup07242	ENSG00000119938	ENST00000238994	PPP1R3C
isotig22152	5.074	4.685	4.625	3.413	2519	0.142171414	isogroup07243	ENSG00000126804	ENST00000554015	ZBTB1
isotig22153	2.002	3.273	4.631	4.754	2524	0.176147892	isogroup07244	ENSG00000128694	ENST00000522700	OSGEPL1
isotig22154	2.778	5.621	1.881	3.975	2523	0.74844067	isogroup07245	ENSG00000115993	ENST00000332624	TRAK2
isotig22156	12.144	11.68	11.045	8.445	2521	0.278556399	isogroup07247	ENSG00000106524	ENST00000306999	ANKMY2
isotig22157	19.844	23.825	23.369	21.205	2519	0.086542797	isogroup07248	ENSG00000116906	ENST00000366647	GNPAT
isotig22158	3.296	3.307	2.798	2.948	2521	0.072142481	isogroup07249	ENSG00000168803	ENST00000562188	ADAL
isotig22159	116.103	96.535	28.239	69.32	2519	0.199829037	isogroup07250	ENSG00000164309	ENST00000446378	CMYA5
isotig22160	8.471	5.193	6.673	4.846	2518	0.643495904	isogroup07251	ENSG00000087111	ENST00000308360	PIGS
isotig22161	2.663	5.823	1.956	3.607	2520	0.738849853	isogroup07252	ENSG00000175556	ENST00000439603	LONRF3
isotig22162	7.518	6.176	11.353	8.925	2521	0.346058465	isogroup07253	ENSG00000176783	ENST00000319449	RUFY1
isotig22163	4.337	4.904	3.907	2.435	2519	0.158647704	isogroup07254	ENSG00000196586	ENST00000369975	MYO6
isotig22164	2.902	2.63	2.522	2.888	2519	0.014428496	isogroup07255	ENSG00000137802	ENST00000514566	MAPKBP1
isotig22165	3.724	2.664	4.479	3.115	2515	0.367964229	isogroup07256	ENSG00000183801	ENST00000530135	OLFML1
isotig22167	3.112	2.557	3.141	2.002	2516	0.392781994	isogroup07258	ENSG00000096717	ENST00000432464	SIRT1
isotig22168	5.944	6.105	6.914	5.487	2513	0.153593973	isogroup07259	ENSG00000171490	ENST00000396503	RSL1D1
isotig22169	7.976	3.604	7.602	5.877	2514	0.716511986	isogroup07260	ENSG00000126903	ENST00000393586	SLC10A3
isotig22170	2.424	3.196	2.189	2.031	2513	0.227521229	isogroup07261	ENSG00000066422	ENST00000312938	ZBTB11
isotig22171	11.951	4.724	11.425	6.876	2510	0.813660104	isogroup07262	ENSG00000138758	ENST00000505788	11-Sep
isotig22172	5.426	5.126	7.749	8.183	2514	0.046836252	isogroup07263	ENSG00000176974	ENST00000316694	SHMT1
isotig22173	6.744	7.073	7.399	7.462	2508	0.301767867	isogroup07264	ENSG00000078237	ENST00000179259	C12orf5
isotig22175	1.336	1.485	1.551	3.119	2512	0.34710115	isogroup07266	ENSG00000055332	ENST00000395127	EIF2AK2
isotig22176	7.785	5.644	3.363	4.131	2509	0.160714286	isogroup07267	ENSG00000196914	ENST00000397843	ARHGEF12
isotig22177	5.298	5.968	4.552	3.992	2507	0.069775306	isogroup07268	ENSG00000118217	ENST00000367942	ATF6
isotig22178	3.591	4.508	4.057	4.186	2508	0.328755542	isogroup07269	ENSG00000093000	ENST00000347635	NUP50
isotig22179	55.611	57.794	78.536	105.621	2507	0.46525325	isogroup07270	ENSG00000261309	ENST00000567955	KCNJ12.1
isotig22180	3.21	2.733	3.182	3.224	2507	0.006660029	isogroup07271	ENSG00000172375	ENST00000336702	C2CD2L
isotig22181	2.095	3.834	4.574	4.126	2507	0.197837604	isogroup07272	ENSG00000060971	ENST00000333167	ACAA1
isotig22182	4.718	3.767	4.413	3.77	2506	0.295699632	isogroup07273	ENSG00000035687	ENST00000366535	ADSS
isotig22184	2.319	3.449	3.124	3.341	2506	0.259196288	isogroup07275	ENSG00000186480	ENST00000340368	INSIG1
isotig22185	4.463	2.81	2.792	2.412	2506	0.343606748	isogroup07276	ENSG00000213462	ENST00000394323	ERV3-1
isotig22187	1.963	3.612	2.943	1.959	2503	0.060757496	isogroup07278	ENSG00000150995	ENST00000443694	ITPR1
isotig22188	39.032	18.859	66.556	33.567	2503	0.839210566	isogroup07279	ENSG00000112280	ENST00000370499	COL9A1
isotig22189	6.268	2.671	5.397	3.169	2501	0.709213196	isogroup07280	ENSG00000061938	ENST00000381916	TNK2
isotig22190	2.657	5.034	3.821	5.429	2503	0.695977681	isogroup07281	ENSG00000005102	ENST00000318579	MEOX1
isotig22191	2.968	3.924	2.774	3.247	2503	0.363521079	isogroup07282	ENSG00000001631	ENST00000412043	KRIT1
isotig22193	3.473	2.945	3.721	3.16	2501	0.243912978	isogroup07284	ENSG00000089818	ENST00000545179	NECAP1
isotig22194	7.354	5.748	6.223	4.826	2497	0.421094161	isogroup07285	ENSG00000158435	ENST00000289382	C2orf29
isotig22195	15.394	10.621	16.899	13.907	2500	0.519219208	isogroup07286	ENSG00000184682	ENST00000391480	C11orf89
isotig22196	1.732	1.536	2.526	6.6	2498	0.417646727	isogroup07287	ENSG00000163840	ENST00000296161	DTX3L
isotig22197	1.471	1.44	2.898	1.882	2498	0.306098294	isogroup07288	ENSG00000236112	ENST00000444738	AC006035.2
isotig22198	3.483	4.355	4.912	4.37	2500	0.126258736	isogroup07289	ENSG00000155256	ENST00000359980	ZFYVE27
isotig22199	5.003	3.214	4.611	3.897	2499	0.429369881	isogroup07290	ENSG00000060491	ENST00000370469	OGFR
isotig22201	10.535	7.236	11.564	8.241	2491	0.553984745	isogroup07292	ENSG00000147654	ENST00000531677	EBAG9
isotig22202	2.301	2.972	1.853	1.68	2491	0.217714361	isogroup07293	ENSG00000164219	ENST00000419445	PGGT1B
isotig22203	3.29	3.845	4.396	4.504	2495	0.296958368	isogroup07294	ENSG00000184857	ENST00000333050	TMEM186
isotig22204	4.287	3.057	4.592	5.101	2497	0.205991208	isogroup07295	ENSG00000140854	ENST00000379661	KATNB1
isotig22205	7.927	5.571	4.857	3.599	2494	0.558277598	isogroup07296	ENSG00000121741	ENST00000456228	ZMYM2
isotig22206	3.869	1.344	2.711	2.11	2493	0.583997144	isogroup07297	ENSG00000117155	ENST00000437941	SSX2IP
isotig22208	4.004	4.213	4.435	4.425	2494	0.065830014	isogroup07299	ENSG00000048991	ENST00000429703	R3HDM1
isotig22209	11.911	6.515	9.562	7.196	2495	0.655510258	isogroup07300	ENSG00000197785	ENST00000378756	ATAD3A
isotig22211	5.63	4.396	3.154	3.447	2494	0.160310363	isogroup07302	ENSG00000073614	ENST00000399788	KDM5A
isotig22212	7.532	15.083	18.213	17.084	2495	0.27102277	isogroup07303	ENSG00000130699	ENST00000252996	TAF4
isotig22213	2.603	4.381	5.656	5.027	2494	0.140912678	isogroup07304	ENSG00000110955	ENST00000262030	ATP5B
isotig22214	7.243	5.901	4.813	4.99	2490	0.151574359	isogroup07305	ENSG00000139921	ENST00000457354	TMX1
isotig22215	2.973	3.631	2.489	2.27	2485	0.054116255	isogroup07306	ENSG00000135111	ENST00000349155	TBX3
isotig22216	3.617	3.96	3.226	2.742	2490	0.143270459	isogroup07307	ENSG00000073060	ENST00000261693	SCARB1
isotig22217	3.606	3.069	4.262	2.124	2487	0.378888931	isogroup07308	ENSG00000135269	ENST00000358204	TES
isotig22218	7.168	5.501	6.835	7.036	2488	0.245171714	isogroup07309	ENSG00000100393	ENST00000263253	EP300
isotig22219	19.939	21.007	22.931	22.216	2489	0.039424739	isogroup07310	ENSG00000144579	ENST00000273062	CTDSP1
isotig22220	5.89	4.642	4.139	4.605	2489	0.10625047	isogroup07311	ENSG00000095370	ENST00000373277	SH2D3C
isotig22222	22.575	19.494	24.491	19.252	2485	0.412508454	isogroup07313	ENSG00000033050	ENST00000222388	ABCF2
isotig22223	1.625	2.5	3.474	3.256	2482	0.142387465	isogroup07314	ENSG00000147162	ENST00000373719	OGT
isotig22224	2.645	2.26	2.743	2.469	2484	0.160639137	isogroup07315	ENSG00000111335	ENST00000392583	OAS2
isotig22225	5.327	4.233	5.423	5.132	2482	0.254837679	isogroup07316	ENSG00000138942	ENST00000518626	RNF185
isotig22226	4.94	3.857	4.255	4.014	2482	0.198626663	isogroup07317	ENSG00000116731	ENST00000235372	PRDM2
isotig22227	49.083	36.596	64.597	50.97	2482	0.495359585	isogroup07318	ENSG00000015676	ENST00000355451	NUDCD3

isotig22228	2.367	2.594	2.308	1.742	2480	0.108908845	isogroup07319	ENSG00000117139	ENST00000538292	KDM5B
isotig22230	5.118	5.699	4.203	4.662	2478	0.241677313	isogroup07321	ENSG00000106397	ENST00000223127	PLOD3
isotig22231	12.591	8.295	11.022	15.326	2476	0.26502029	isogroup07322	ENSG00000231925	ENST00000434618	TAPBP
isotig22232	12.577	5.485	8.411	6.823	2474	0.724327422	isogroup07323	ENSG00000137251	ENST00000370865	TINAG
isotig22233	2.422	4.693	5.993	6.234	2474	0.214426618	isogroup07324	ENSG00000110713	ENST00000397007	NUP98
isotig22234	8.617	6.827	10.281	7.034	2473	0.50898963	isogroup07325	ENSG00000116918	ENST00000366639	TSNAX
isotig22235	6.953	3.787	5.831	3.639	2473	0.662038777	isogroup07326	ENSG00000116260	ENST00000367602	QSOX1
isotig22236	6.04	4.518	5.719	4.038	2473	0.505786428	isogroup07327	ENSG00000169180	ENST00000565698	XPO6
isotig22237	18.184	16.376	21.545	25.251	2470	0.069850455	isogroup07328	ENSG00000090432	ENST00000264198	MUL1
isotig22239	5.308	3.91	4.609	3.338	2468	0.441393627	isogroup07330	ENSG00000143889	ENST00000409636	HNRPLL
isotig22241	2.497	3.006	4.588	2.29	2463	0.243912978	isogroup07332	ENSG00000103855	ENST00000564751	CD276
isotig22242	7.737	5.289	6.487	4.532	2465	0.632308184	isogroup07333	ENSG00000156502	ENST00000359655	SUPV3L1
isotig22243	3.868	4.226	5.589	3.891	2468	0.130373112	isogroup07334	ENSG00000110906	ENST00000228495	KCTD10
isotig22245	7.97	9.807	7.208	8.521	2467	0.35498234	isogroup07336	ENSG00000100100	ENST00000215912	PIK3IP1
isotig22247	4.674	4.851	6.721	5.16	2461	0.185522657	isogroup07338	ENSG00000112079	ENST00000229812	STK38
isotig22248	42.869	20.263	41.983	43.128	2463	0.554097467	isogroup07339	ENSG00000173442	ENST00000309295	EHBP1L1
isotig22249	7.13	5.08	5.857	3.419	2462	0.573025475	isogroup07340	ENSG00000198722	ENST00000378495	UNC13B
isotig22251	5.485	10.588	8.585	6.149	2454	0.56981288	isogroup07342	ENSG00000136859	ENST00000373425	ANGPTL2
isotig22252	2.93	3.109	2.341	1.619	2461	0.102323965	isogroup07343	ENSG00000175356	ENST00000450649	SCUBE2
isotig22255	2.955	4.557	4.391	4.204	2460	0.393834072	isogroup07346	ENSG00000196531	ENST00000454682	NACA
isotig22256	4.155	1.984	3.827	2.862	2456	0.584307132	isogroup07347	ENSG00000142197	ENST00000399151	DOPEY2
isotig22257	4.192	4.948	3.561	3.179	2458	0.160310363	isogroup07348	ENSG00000075711	ENST00000450955	DLG1
isotig22258	58.916	165.477	111.359	102.201	2445	0.796629593	isogroup07349	ENSG00000185437	ENST00000333634	SH3BGR
isotig22259	6.684	2.123	2.684	3.254	2456	0.721913279	isogroup07350	ENSG00000197535	ENST00000399233	MYO5A
isotig22260	3.104	1.282	3.149	2.192	2451	0.622980386	isogroup07351	ENSG00000235531	ENST00000537896	RP11-383H13.1.1
isotig22262	5.129	3.453	5.804	4.025	2453	0.54090892	isogroup07353	ENSG00000172070	ENST00000381962	SRXN1
isotig22264	8.304	11.841	9.65	8.715	2453	0.287809048	isogroup07355	ENSG00000166508	ENST00000303887	MCM7
isotig22265	6.032	4.024	3.233	2.568	2450	0.44935936	isogroup07356	ENSG00000132640	ENST00000405977	BTBD3
isotig22266	1.548	2.601	3.466	3.288	2451	0.19021004	isogroup07357	ENSG00000171811	ENST00000368586	TTC40
isotig22267	6.469	5.566	4.758	4.477	2445	0.059639663	isogroup07358	ENSG00000101052	ENST00000373030	IFT52
isotig22268	11.164	6.754	9.342	9.223	2449	0.478216352	isogroup07359	ENSG00000156471	ENST00000517309	PTDSS1
isotig22269	1.65	1.76	1.601	1.904	2445	0.121392876	isogroup07360	ENSG00000184178	ENST00000401642	SCFD2
isotig22270	11.622	9.933	12.345	11.326	2447	0.331695724	isogroup07361	ENSG00000112996	ENST00000507110	MRPS30
isotig22272	2.464	8.328	2.014	5.039	2448	0.863906215	isogroup07363	ENSG00000170145	ENST00000304987	SIK2
isotig22273	2.294	4.545	4.393	2.032	2446	0.129828286	isogroup07364	ENSG00000153823	ENST00000392055	PID1
isotig22274	10.157	6.27	7.321	6.458	2444	0.49487112	isogroup07365	ENSG00000168118	ENST00000366690	RAB4A
isotig22276	7.3	6.887	6.704	5.691	2443	0.182300669	isogroup07367	ENSG00000144635	ENST00000273130	DYNC1LI1
isotig22278	8.279	9.112	9.13	6.368	2445	0.189176749	isogroup07369	ENSG00000175029	ENST00000531469	CTBP2
isotig22279	4.441	3.04	3.584	2.237	2442	0.514268806	isogroup07370	ENSG00000079246	ENST00000392132	XRCC5
isotig22280	2.655	2.499	2.273	2.088	2445	0.071109191	isogroup07371	ENSG00000198198	ENST00000562955	SZT2
isotig22281	27.108	26.802	33.468	31.138	2443	0.103047268	isogroup07372	ENSG00000124164	ENST00000475243	VAPB
isotig22282	6.096	4.93	3.702	4.04	2444	0.017143233	isogroup07373	ENSG00000134287	ENST00000541959	ARF3
isotig22283	2.518	4.254	2.993	2.951	2443	0.405284813	isogroup07374	ENSG00000214013	ENST00000318010	GANC
isotig22284	12.199	15.954	8.843	11.17	2441	0.450834147	isogroup07375	ENSG00000139531	ENST00000548274	SUOX
isotig22285	4.345	5.279	4.41	3.925	2441	0.092404374	isogroup07376	ENSG00000146067	ENST00000443375	FAM193B
isotig22286	4.092	2.676	2.58	2.22	2440	0.417458856	isogroup07377	ENSG00000172748	ENST00000398612	ZNF596
isotig22287	4.304	4.453	3.275	2.779	2441	0.042524611	isogroup07378	ENSG00000151092	ENST00000417874	NGLY1
isotig22288	4.24	3.393	3.829	3.881	2440	0.168726986	isogroup07379	ENSG00000183495	ENST00000541296	EP400
isotig22289	9.087	6.464	9.456	7.327	2435	0.517810175	isogroup07380	ENSG00000116455	ENST00000235090	WDR77
isotig22290	3.324	3.754	2.62	2.8	2439	0.186405651	isogroup07381	ENSG00000204138	ENST00000373836	PHACTR4
isotig22291	3.292	5.229	3.793	4.241	2439	0.432366424	isogroup07382	ENSG00000121634	ENST00000369235	GJA8
isotig22292	7.877	4.885	7.442	4.975	2439	0.644209814	isogroup07383	ENSG00000228177	ENST00000442972	C6orf47
isotig22294	9.064	7.287	7.001	5.503	2437	0.404101225	isogroup07385	ENSG00000141985	ENST00000269886	SH3GL1
isotig22295	3.499	4.147	3.508	3.233	2437	0.084044112	isogroup07386	ENSG00000184194	ENST00000332582	GPR173
isotig22296	3.46	2.43	3.54	2.619	2436	0.391213271	isogroup07387	ENSG00000119725	ENST00000555044	ZNF410
isotig22297	12.97	12.221	14.894	9.658	2436	0.494091456	isogroup07388	ENSG00000183751	ENST00000568546	TBL3
isotig22298	22.751	26.129	25.75	23.217	2428	0.117400616	isogroup07389	ENSG00000146731	ENST00000275603	CCT6A
isotig22299	1.583	2.689	3.329	3.227	2435	0.187241677	isogroup07390	ENSG00000171195	ENST00000456088	MUC7
isotig22300	19.224	20.443	21.98	18.64	2432	0.085434358	isogroup07391	ENSG00000130638	ENST00000252934	ATXN10
isotig22301	2.461	2.815	3.133	2.631	2434	0.140846923	isogroup07392	ENSG00000111412	ENST00000261318	C12orf49
isotig22302	6.37	4.277	3.49	3.772	2439	0.178017209	isogroup07393	ENSG00000007944	ENST00000356840	MYLIP
isotig22303	5.501	4.796	5.435	4.286	2429	0.291726159	isogroup07394	ENSG00000148187	ENST00000344641	MRRF
isotig22304	1.387	2.283	3.293	3.302	2434	0.164330803	isogroup07395	ENSG00000213339	ENST00000250237	QTRT1
isotig22307	19.156	17.941	21.484	18.017	2403	0.234810626	isogroup07398	ENSG00000116473	ENST00000545460	RAP1A
isotig22308	11.432	11.663	7.827	7.306	2430	0.03617457	isogroup07399	ENSG00000248458	ENST00000502413	AL139147.1
isotig22309	17.038	16.012	25.673	27.157	2431	0.0234557	isogroup07400	ENSG00000260283	ENST00000562963	CNP.1
isotig22310	3.108	6.41	3.831	4	2429	0.689984595	isogroup07401	ENSG00000147113	ENST00000398000	CXorf36
isotig22312	25.709	17.575	23.1	22.488	2426	0.413804764	isogroup07403	ENSG00000149823	ENST00000279281	C11orf2
isotig22313	26.812	3.97	7.847	3.387	2426	0.953201323	isogroup07404	ENSG00000110237	ENST00000263674	ARHGEF17
isotig22316	3.472	2.656	2.318	2.791	2422	0.128428647	isogroup07407	ENSG00000145012	ENST00000543006	LPP
isotig22319	23.092	23.225	30.66	21.199	2412	0.43949613	isogroup07410	ENSG00000170515	ENST00000303305	PA2G4
isotig22320	5.125	4.003	4.555	4.311	2420	0.226018261	isogroup07411	ENSG00000148356	ENST00000373322	LRSAM1
isotig22321	2.271	4.078	3.169	3.018	2418	0.377714737	isogroup07412	ENSG00000168936	ENST00000536901	TMEM129
isotig22322	15.618	5.605	16.044	9.61	2418	0.860787555	isogroup07413	ENSG00000167945	ENST00000301698	PRR25
isotig22323	4.193	3.31	3.448	2.948	2415	0.288560532	isogroup07414	ENSG00000135365	ENST00000257821	PHF21A
isotig22324	4.666	2.302	4.1	2.921	2414	0.63816976	isogroup07415	ENSG00000213121	ENST00000392385	AL590867.1
isotig22326	2.418	2.557	2.641	3.703	2412	0.273427519	isogroup07417	ENSG00000132109	ENST00000254436	TRIM21
isotig22328	1.705	2.863	3.023	2.217	2413	0.296009619	isogroup07419	ENSG00000138119	ENST00000371501	MYOF
isotig22329	2.849	2.29	4.273	2.767	2411	0.394745247	isogroup07420	ENSG00000161381	ENST00000444911	PLXDC1
isotig22330	2.696	4.015	2.891	2.77	2408	0.28474675	isogroup07421	ENSG00000164597	ENST00000297135	COG5
isotig22331	23.029	17.897	24.709	23.929	2410	0.177970241	isogroup07422	ENSG00000106105	ENST00000389266	GARS
isotig22334	6.934	8.78	6.572	7.54	2402	0.417458856	isogroup07425	ENSG00000138381	ENST00000260952	ASNSD1

isotig22336	1.741	3.429	4.017	4.052	2409	0.193676261	isogroup07427	ENSG00000185100	ENST00000330877	ADSSL1
isotig22337	2.148	4.782	4.148	4.035	2408	0.664922597	isogroup07428	ENSG00000111452	ENST00000535015	GPR133
isotig22338	6.15	6.608	10.382	6.949	2408	0.481522883	isogroup07429	ENSG00000104973	ENST00000355584	MED25
isotig22339	1.294	2.412	3	2.735	2407	0.119720824	isogroup07430	ENSG00000123453	ENST00000539227	SARDH
isotig22340	4.198	3.179	5.079	4.055	2407	0.426044563	isogroup07431	ENSG00000197586	ENST00000376652	ENTPD6
isotig22341	4.399	3.948	5.227	4.671	2406	0.178017209	isogroup07432	ENSG00000132740	ENST00000255078	IGHMBP2
isotig22342	3.056	4.052	2.941	3.453	2405	0.368246036	isogroup07433	ENSG00000163877	ENST00000436196	SNIP1
isotig22346	4.876	4.14	2.585	2.556	2400	0.065830014	isogroup07437	ENSG00000059573	ENST00000371224	ALDH18A1
isotig22349	4.297	2.731	2.495	2.334	2398	0.379856467	isogroup07440	ENSG00000198301	ENST00000356260	SDAD1
isotig22350	3.004	2.974	3.193	2.733	2400	0.128428647	isogroup07441	ENSG00000121350	ENST00000240651	PYROXD1
isotig22351	5.324	4.836	3.559	2.849	2397	0.138536109	isogroup07442	ENSG00000065135	ENST00000369851	GNAI3
isotig22352	8.337	11.557	10.548	11.329	2393	0.497369805	isogroup07443	ENSG00000114544	ENST00000360370	SLC41A3
isotig22353	13.751	11.379	13.455	10.258	2392	0.441421808	isogroup07444	ENSG00000125633	ENST00000376300	CCDC93
isotig22354	13.784	12.501	18.666	15.452	2392	0.403312167	isogroup07445	ENSG00000149792	ENST00000526319	MRPL49
isotig22355	4.551	9.048	3.892	4.662	2396	0.587829714	isogroup07446	ENSG00000205268	ENST00000401827	PDE7A
isotig22357	2.769	2.425	2.291	1.44	2394	0.309057263	isogroup07448	ENSG00000136891	ENST00000444730	TEX10
isotig22358	3.071	2.402	2.083	2.273	2393	0.177697828	isogroup07449	ENSG00000083223	ENST00000375963	ZCCHC6
isotig22359	4.956	2.736	5.815	8.547	2394	0.058775457	isogroup07450	ENSG00000065717	ENST00000262953	TLE2
isotig22360	7.653	3.214	6.273	4.126	2393	0.734557	isogroup07451	ENSG00000047662	ENST00000265018	FAM184B
isotig22361	19.186	15.598	21.991	14.734	2393	0.565613963	isogroup07452	ENSG00000180011	ENST00000322342	ZADH2
isotig22362	5.187	4.752	4.186	4.629	2391	0.019651311	isogroup07453	ENSG00000188706	ENST00000371064	ZDHHC9
isotig22363	3.103	7.03	2.59	2.578	2388	0.84453671	isogroup07454	ENSG00000106819	ENST00000375544	ASPN
isotig22365	8.038	5.192	3.894	1.705	2390	0.647666642	isogroup07456	ENSG00000106025	ENST00000415871	TSPAN12
isotig22366	1.954	1.767	1.537	1.582	2391	0.041209514	isogroup07457	ENSG00000104497	ENST00000396330	SNX16
isotig22368	3.054	3.086	3.063	2.551	2387	0.161343654	isogroup07459	ENSG00000082516	ENST00000285873	GEMIN5
isotig22369	6.765	4.671	3.294	3.722	2388	0.306239197	isogroup07460	ENSG00000154727	ENST00000400075	GABPA
isotig22370	10.052	7.188	9.372	8.172	2386	0.425011272	isogroup07461	ENSG00000243725	ENST00000371281	TTC4
isotig22372	2.693	1.562	7.369	2.353	2388	0.767866536	isogroup07463	ENSG00000099331	ENST00000397274	MYO9B
isotig22374	8.037	5.101	7.724	6.206	2388	0.551758473	isogroup07465	ENSG00000170604	ENST00000302165	IRF2BP1
isotig22376	50.101	47.872	43.622	40.351	2359	0.166763733	isogroup07467	ENSG00000198898	ENST00000361183	CAPZA2
isotig22377	14.606	6.254	10.44	6.23	2386	0.83937965	isogroup07468	ENSG00000091536	ENST00000205890	MYO15A
isotig22379	14.773	12.899	15.005	15.059	2386	0.186772	isogroup07470	ENSG00000170638	ENST00000395829	TRABD
isotig22380	5.724	6.678	6.114	3.795	2383	0.201688961	isogroup07471	ENSG00000123545	ENST00000316149	NDUFAF4
isotig22381	6.364	6.257	4.139	3.891	2382	0.096866311	isogroup07472	ENSG00000114978	ENST00000396049	MOB1A
isotig22383	2.317	1.88	3.322	2.183	2379	0.418905463	isogroup07474	ENSG00000140263	ENST00000267814	SORD
isotig22587	7.644	8.064	8.497	7.456	2273	0.064392801	isogroup07678	ENSG00000147164	ENST00000374274	SNX12
isotig22588	26.156	31.176	23.988	27.732	2270	0.442229654	isogroup07679	ENSG00000004478	ENST00000001008	FKBP4
isotig22589	2.431	3.563	2.88	2.469	2276	0.208518073	isogroup07680	ENSG00000256043	ENST00000433477	CTSO
isotig22590	6.604	1.973	5.084	3.49	2277	0.786531525	isogroup07681	ENSG00000068137	ENST00000412503	PLEKHH3
isotig22591	6.865	3.907	7.962	7.469	2275	0.500065755	isogroup07682	ENSG00000071051	ENST00000393349	NCK2
isotig22594	3.707	2.919	3.712	2.918	2263	0.423226497	isogroup07685	ENSG00000047621	ENST00000545746	C12orf4
isotig22595	5.577	4.847	5.213	5.267	2274	0.1324303	isogroup07686	ENSG00000168434	ENST00000307149	COG7
isotig22596	2.77	6.965	3.699	4.406	2271	0.717507703	isogroup07687	ENSG00000167112	ENST00000372890	TRUB2
isotig22597	2.261	2.583	2.733	2.298	2270	0.09882017	isogroup07688	ENSG00000163510	ENST00000410053	CWC22
isotig22599	7.341	5	7.251	5.821	2269	0.513329451	isogroup07690	ENSG00000157837	ENST00000353487	SPPL3.1
isotig22600	4.386	3.368	4.397	4.03	2272	0.303684151	isogroup07691	ENSG00000160410	ENST00000291842	SHKBP1
isotig22601	2.146	3.386	2.943	3.401	2271	0.566149395	isogroup07692	ENSG00000067057	ENST00000381125	PFKP
isotig22602	3.115	1.677	2.221	1.538	2270	0.415364094	isogroup07693	ENSG00000138801	ENST00000265174	PAPSS1
isotig22604	15.801	10.037	14.853	12.367	2270	0.665016533	isogroup07695	ENSG00000158773	ENST00000368021	USF1
isotig22605	5.004	3.526	4.447	3.239	2267	0.480254753	isogroup07696	ENSG00000125695	ENST00000336174	STRADA
isotig22606	12.317	4.05	6.345	4.793	2270	0.847749305	isogroup07697	ENSG00000127838	ENST00000258362	PNKD
isotig22609	2.834	2.819	2.791	2.417	2269	0.097401743	isogroup07700	ENSG00000149582	ENST00000533102	TMEM25
isotig22611	32.536	84.751	21.383	36.007	2265	0.933869392	isogroup07702	ENSG00000023330	ENST00000484952	ALAS1
isotig22613	5.972	5.439	5.13	3.233	2264	0.245312617	isogroup07704	ENSG00000164035	ENST00000296420	EMCN
isotig22614	6.725	3.284	8.263	3.932	2266	0.829244007	isogroup07705	ENSG00000165655	ENST00000535216	ZNF503
isotig22616	4.684	4.128	3.156	2.828	2264	0.176147892	isogroup07707	ENSG00000133731	ENST00000449740	IMPA1
isotig22617	5.765	5.326	3.335	3.673	2267	0.301072744	isogroup07708	ENSG00000058668	ENST00000367219	ATP2B4
isotig22618	1.409	1.93	1.978	1.885	2268	0.178778087	isogroup07709	ENSG00000119397	ENST00000454238	CNTRL
isotig22619	3.809	2.756	3.139	2.51	2265	0.287076351	isogroup07710	ENSG00000069849	ENST00000286371	ATP1B3
isotig22620	2.568	3.617	1.722	1.326	2263	0.177697828	isogroup07711	ENSG00000138413	ENST00000446179	IDH1
isotig22621	5.813	6.02	5.644	5.023	2264	0.150766514	isogroup07712	ENSG00000119953	ENST00000369592	SMNDC1
isotig22623	7.292	4.311	6.106	5.12	2265	0.564148568	isogroup07714	ENSG00000182095	ENST00000430969	TNRC18
isotig22624	23.97	29.544	39.166	43.081	2262	0.286052454	isogroup07715	ENSG00000163820	ENST00000535325	FYCO1
isotig22625	2.161	3.674	4.885	4.578	2264	0.155266025	isogroup07716	ENSG00000126522	ENST00000395331	ASL
isotig22626	4.695	2.843	4.227	2.928	2263	0.569934997	isogroup07717	ENSG00000241945	ENST00000291576	PWP2
isotig22628	4.344	10.064	6.326	5.394	2262	0.598895318	isogroup07719	ENSG00000151498	ENST00000281182	ACAD8
isotig22631	2.991	2.994	2.217	2.222	2257	0.054116255	isogroup07722	ENSG00000168014	ENST00000334126	C2CD3
isotig22633	7.728	1.046	2.644	1.558	2256	0.858476742	isogroup07724	ENSG00000158445	ENST00000538812	KCNB1
isotig22634	2.903	2.717	3.364	3.176	2256	0.108279477	isogroup07725	ENSG00000049167	ENST00000265038	ERCC8
isotig22635	6.114	16.222	12.026	6.406	2254	0.505232209	isogroup07726	ENSG00000147257	ENST00000394299	GPC3
isotig22636	3.447	5.675	3.875	3.724	2254	0.435663561	isogroup07727	ENSG00000164253	ENST00000296679	WDR41
isotig22637	5.63	5.881	7.874	6.291	2256	0.128071692	isogroup07728	ENSG00000169919	ENST00000304895	GUSB
isotig22638	9.604	3.927	5.89	4.023	2254	0.76502029	isogroup07729	ENSG00000183248	ENST00000539422	AC010336.1
isotig22639	5.58	8.221	5.437	3.634	2255	0.138761554	isogroup07730	ENSG00000161996	ENST00000549091	WDR90
isotig22640	7.537	16.485	8.508	8.518	2255	0.760069888	isogroup07731	ENSG00000113657	ENST00000398514	DPYSL3
isotig22641	2.872	1.783	3.401	2.673	2253	0.444352596	isogroup07732	ENSG00000106780	ENST00000426959	MEGF9
isotig22643	15.083	26.983	27.652	27.598	2251	0.484143684	isogroup07734	ENSG00000157557	ENST00000360938	ETS2
isotig22644	3.229	1.522	2.535	1.323	2251	0.622247689	isogroup07735	ENSG00000163257	ENST00000536863	DCAF16
isotig22645	26.008	23.119	25.749	20.622	2246	0.310362967	isogroup07736	ENSG00000144224	ENST00000272638	UBXN4
isotig22646	13.169	22.064	23.807	26.289	2243	0.542571579	isogroup07737	ENSG00000161558	ENST00000293261	TMEM143
isotig22647	4.777	4.113	5.386	2.787	2249	0.56063538	isogroup07738	ENSG00000117569	ENST00000236228	PTBP2
isotig22648	5.155	4.042	4.758	3.212	2251	0.465018411	isogroup07739	ENSG00000153815	ENST00000537098	CMIP

isotig22649	15.142	9.229	18.555	16.367	2250	0.520928834	isogroup07740	ENSG00000130433	ENST00000252729	CACNG6
isotig22651	14.705	10.148	15.016	11.945	2249	0.552716615	isogroup07742	ENSG00000124155	ENST00000279036	PIGT
isotig22652	6.097	5.311	4.741	4.683	2247	0.109547607	isogroup07743	ENSG00000156650	ENST00000372714	KAT6B
isotig22654	2.099	3.722	5.132	4.478	2245	0.042524611	isogroup07745	ENSG00000130707	ENST00000372394	ASS1
isotig22655	1.52	2.389	3.049	3.118	2247	0.123215225	isogroup07746	ENSG00000176438	ENST00000557275	C14orf49
isotig22656	22.425	23.293	20.34	14.383	2246	0.116301571	isogroup07747	ENSG00000122642	ENST00000538336	FKBP9
isotig22657	4.428	3.492	4.176	3.967	2246	0.204507026	isogroup07748	ENSG00000148843	ENST00000369797	PDCD11
isotig22658	5.44	1.841	4.495	3.084	2243	0.747820696	isogroup07749	ENSG00000138443	ENST00000417864	ABI2
isotig22660	1.89	4.979	3.619	2.868	2240	0.534718569	isogroup07751	ENSG00000144891	ENST00000542281	AGTR1
isotig22661	2.528	2.451	2.213	2.324	2244	0.035704892	isogroup07752	ENSG00000196345	ENST00000426540	ZNF167
isotig22662	6.056	2.176	6.078	5.239	2239	0.664913204	isogroup07753	ENSG00000204519	ENST00000359821	ZNF551
isotig22665	6.538	4.321	4.711	3.405	2242	0.613831066	isogroup07756	ENSG00000121749	ENST00000550746	TBC1D15
isotig22666	2.635	2.079	2.025	2.276	2243	0.084617119	isogroup07757	ENSG00000131899	ENST00000316843	LLGL1
isotig22667	4.385	2.013	3.634	2.457	2241	0.673151349	isogroup07758	ENSG00000130803	ENST00000360385	ZNF317
isotig22668	1.802	2.946	3.994	4.323	2242	0.249755768	isogroup07759	ENSG00000184470	ENST00000542719	TXNRD2
isotig22669	2.476	3.583	3.385	3.395	2242	0.196062223	isogroup07760	ENSG00000145996	ENST00000378610	CDKAL1
isotig22670	6.657	3.355	5.108	2.767	2241	0.739498009	isogroup07761	ENSG00000099331	ENST00000397274	MYO9B
isotig23087	11.005	16.285	16.893	11.836	2056	0.18722289	isogroup08178	ENSG00000124343	ENST00000419513	XG
isotig23088	5.454	3.922	4.845	3.655	2058	0.518796498	isogroup08179	ENSG00000034693	ENST00000367591	PEX3
isotig23089	2.243	2.248	2.316	2.111	2057	0.084617119	isogroup08180	ENSG00000156603	ENST00000431606	MED19
isotig23090	90.321	63.185	82.655	72.951	2053	0.541763733	isogroup08181	ENSG00000100129	ENST00000425539	EIF3L
isotig23091	3.545	3.139	2.91	3.212	2056	0.072142481	isogroup08182	ENSG00000196943	ENST00000267425	C14orf21
isotig23092	4.711	5.546	5.461	4.562	2056	0.007420906	isogroup08183	ENSG00000105447	ENST00000253237	GRWD1
isotig23093	34.177	31.647	31.735	36.773	2051	0.08824303	isogroup08184	ENSG00000168724	ENST00000342382	DNAJC21
isotig23094	3.984	2.418	3.019	2.113	2055	0.500864207	isogroup08185	ENSG00000169155	ENST00000449886	ZBTB43
isotig23096	4.017	2.543	2.835	2.339	2058	0.449406327	isogroup08187	ENSG00000146373	ENST00000521654	RNF217
isotig23097	2.931	5.847	8.67	6.298	2051	0.125544826	isogroup08188	ENSG00000183248	ENST00000539422	AC010336.1
isotig23098	5.7	4.126	4.434	4.58	2053	0.228413617	isogroup08189	ENSG00000089234	ENST00000419234	BRAP
isotig23099	2.825	2.73	2.873	1.933	2050	0.210312242	isogroup08190	ENSG00000104613	ENST00000397977	INTS10
isotig23100	15.445	9.638	15.864	21.121	2052	0.142998046	isogroup08191	ENSG00000111321	ENST00000228918	LTBR
isotig23101	39.122	38.804	39.575	28.09	2045	0.315031562	isogroup08192	ENSG00000138363	ENST00000435675	ATIC
isotig23103	5.078	8.778	4.632	4.02	2049	0.382618171	isogroup08194	ENSG00000113070	ENST00000507104	HBEGF
isotig23104	5.073	3.61	4.159	3.351	2051	0.391635981	isogroup08195	ENSG00000226916	ENST00000457382	WDR46
isotig23105	8.865	5.135	7.881	5.493	2050	0.658384685	isogroup08196	ENSG00000010322	ENST00000479054	NISCH
isotig23106	6.056	7.026	7.673	7.291	2048	0.032651988	isogroup08197	ENSG00000003509	ENST00000002125	C2orf56
isotig23107	6.276	3.596	3.407	2.74	2043	0.571672804	isogroup08198	ENSG00000021776	ENST00000156471	AQR
isotig23108	1.598	2.648	3.352	3.081	2049	0.109575787	isogroup08199	ENSG00000254614	ENST00000526623	AP003068.23.1
isotig23109	3.022	2.461	2.577	1.787	2042	0.313481626	isogroup08200	ENSG00000174842	ENST00000370360	GLMN
isotig23110	8.084	4.833	4.391	3.542	2048	0.520506125	isogroup08201	ENSG00000173269	ENST00000372027	MMRN2
isotig23111	4.538	3.524	3.644	4.056	2042	0.180957391	isogroup08202	ENSG00000127081	ENST00000395506	ZNF484
isotig23113	2.555	4.461	6.122	5.662	2048	0.164171113	isogroup08204	ENSG00000204262	ENST00000374866	COL5A2
isotig23115	3.648	1.957	2.894	2.094	2042	0.557601262	isogroup08206	ENSG00000166004	ENST00000411936	KIAA1731
isotig23118	11.108	10.159	9.711	8.895	2046	0.193864132	isogroup08209	ENSG00000077721	ENST00000371558	UBE2A
isotig23119	4.016	2.868	4.119	2.672	2044	0.538823552	isogroup08210	ENSG00000181026	ENST00000332810	AEN
isotig23120	5.642	3.377	4.81	3.304	2046	0.592798903	isogroup08211	ENSG00000145868	ENST00000340253	FBXO38
isotig23121	3.451	3.54	3.046	3.737	2043	0.206319982	isogroup08212	ENSG00000123815	ENST00000324464	ADCK4
isotig23122	63.401	120.277	19.784	18.838	2044	0.708837454	isogroup08213	ENSG00000175445	ENST00000311322	LPL
isotig23125	40.028	37.876	53.502	48.162	2043	0.18264823	isogroup08216	ENSG00000184787	ENST00000345496	UBE2G2
isotig23127	12.243	4.637	7.434	5.17	2044	0.771877583	isogroup08218	ENSG00000153902	ENST00000392225	LGI4
isotig23128	6.107	6.696	7.905	6.692	2044	0.062016232	isogroup08219	ENSG00000183207	ENST00000221413	RUVBL2
isotig23129	44.378	49.368	45.141	40.802	2035	0.056699481	isogroup08220	ENSG00000163468	ENST00000295688	CCT3
isotig23130	4.454	4.012	4.652	4.269	2040	0.014428496	isogroup08221	ENSG00000162174	ENST00000415229	ASRGL1
isotig23133	18.17	13.268	16.039	12.73	2040	0.558709702	isogroup08224	ENSG00000153487	ENST00000375774	ING1
isotig23135	6.144	5.884	4.874	4.086	2038	0.234143684	isogroup08226	ENSG00000133818	ENST00000545643	RRAS2
isotig23136	4.858	3.685	4.038	3.091	2035	0.386572856	isogroup08227	ENSG00000163964	ENST00000392391	PIGX
isotig23137	5.492	5.197	3.729	3.632	2040	0.045004509	isogroup08228	ENSG00000134954	ENST00000319397	ETS1
isotig23139	3.921	6.229	5.479	3.228	2032	0.143448937	isogroup08230	ENSG00000154721	ENST00000480456	JAM2
isotig23141	3.834	3.514	4.306	3.572	2037	0.191280905	isogroup08232	ENSG00000235708	ENST00000553210	COL11A2
isotig23142	3.273	3.306	4.82	4.207	2038	0.076068986	isogroup08233	ENSG00000185129	ENST00000331327	PURA
isotig23143	6.817	7.096	4.634	3.639	2036	0.017143233	isogroup08234	ENSG00000162618	ENST00000370742	ELTD1
isotig23144	9.863	7.905	7.732	8.715	2037	0.10699256	isogroup08235	ENSG00000038274	ENST00000321757	MAT2B
isotig23145	1.863	1.71	1.614	1.264	2037	0.189054633	isogroup08236	ENSG00000147650	ENST00000520873	LRP12
isotig23146	3.718	3.506	4.389	3.727	2036	0.145365221	isogroup08237	ENSG00000170854	ENST00000360258	MINA
isotig23147	1.275	2.533	3.111	3.111	2036	0.260126249	isogroup08238	ENSG00000108439	ENST00000225573	PNPO
isotig23148	4.816	8.994	4.226	3.574	2036	0.629264673	isogroup08239	ENSG00000080573	ENST00000264828	COL5A3
isotig23150	3.855	6.133	2.821	5.234	2036	0.579460059	isogroup08241	ENSG00000221963	ENST00000409652	APOL6
isotig23151	1.726	2.917	3.401	3.277	2036	0.222739911	isogroup08242	ENSG00000119596	ENST00000549293	YLPM1
isotig23152	5.603	2.946	3.012	2.926	2035	0.541641617	isogroup08243	ENSG00000189308	ENST00000505397	LIN54
isotig23154	3.062	2.235	3.294	3.377	2035	0.123543999	isogroup08245	ENSG00000139718	ENST00000542440	SETD1B
isotig23156	15.685	7.155	13.682	12.414	2027	0.692248441	isogroup08247	ENSG00000107960	ENST00000369764	OBFC1
isotig23158	6.863	5.849	7.644	6.383	2034	0.354597204	isogroup08249	ENSG00000179115	ENST00000314606	FARSA
isotig23159	6.209	7.818	7.72	7.026	2033	0.089792966	isogroup08250	ENSG00000148308	ENST00000372097	GTF3C5
isotig23160	3.715	2.782	3.662	3.702	2032	0.257862403	isogroup08251	ENSG00000131748	ENST00000336308	STARD3
isotig23161	6.831	2.735	5.57	2.875	2032	0.794910573	isogroup08252	ENSG00000154358	ENST00000570156	OBSCN
isotig23162	1.957	1.649	1.482	1.246	2031	0.1098482	isogroup08253	ENSG00000104064	ENST00000543881	GABPB1
isotig23163	5.21	3.088	2.559	2.429	2028	0.477474262	isogroup08254	ENSG00000121864	ENST00000496856	ZNF639
isotig23164	3.163	3.305	10.867	2.176	2027	0.869063275	isogroup08255	ENSG00000170345	ENST00000303562	FOS
isotig23166	3.876	3.179	2.783	2.635	2030	0.263846096	isogroup08257	ENSG00000131626	ENST00000389547	PPFIA1
isotig23167	1.348	2.232	2.829	2.801	2030	0.143242279	isogroup08258	ENSG00000087077	ENST00000200457	TRIP6
isotig23168	3.5	1.842	2.115	1.558	2026	0.475980687	isogroup08259	ENSG00000059769	ENST00000313525	DNAJC25
isotig23169	7.904	4.39	6.68	4.424	2029	0.646107312	isogroup08260	ENSG00000132613	ENST00000338779	MTSS1L
isotig23170	21.797	16.889	20.591	15.835	2023	0.488042008	isogroup08261	ENSG00000065548	ENST00000337859	ZC3H15

isotig23171	3.177	2.35	2.881	2.058	2020	0.263686406	isogroup08262	ENSG00000179981	ENST00000322038	TSHZ1
isotig23172	3.994	4.446	3.985	4.698	2025	0.221105433	isogroup08263	ENSG00000142188	ENST00000542230	TMEM50B
isotig23173	1.537	2.563	3.416	3.744	2027	0.306567972	isogroup08264	ENSG00000003989	ENST00000470360	SLC7A2
isotig23174	5.313	4.237	3.964	3.569	2026	0.277241302	isogroup08265	ENSG00000213380	ENST00000306875	COG8
isotig23175	238.618	164.408	65.797	33.307	2011	0.710716164	isogroup08266	ENSG00000215405	ENST00000427390	GOLGA6L6
isotig23176	5.558	5.646	2.766	2.966	2023	0.027992786	isogroup08267	ENSG00000101343	ENST00000536226	CRNKL1
isotig23177	3.608	4.199	2.041	3.116	2026	0.435362967	isogroup08268	ENSG00000131389	ENST00000454876	SLC6A6
isotig23178	5.096	4.519	4.748	4.066	2026	0.324209063	isogroup08269	ENSG00000137824	ENST00000338376	FAM82A2
isotig23179	12.38	16.993	14.385	13.976	2023	0.325749605	isogroup08270	ENSG00000089006	ENST00000377759	SNX5
isotig23182	7.276	2.766	5.394	3.065	2023	0.769820395	isogroup08273	ENSG00000119685	ENST00000557636	TTLL5
isotig23183	3.131	4.867	5.662	3.321	2022	0.102577591	isogroup08274	ENSG00000091972	ENST00000473539	CD200
isotig23185	3.049	2.342	2.272	2.057	2024	0.112901105	isogroup08276	ENSG00000180357	ENST00000326648	ZNF609
isotig23186	6.525	2.437	4.847	3.364	2023	0.754753137	isogroup08277	ENSG00000105663	ENST00000420124	MLL4.1
isotig23187	13.564	8.13	14.339	12.649	2023	0.549644924	isogroup08278	ENSG00000196531	ENST00000454682	NACA
isotig23188	2.705	2.597	2.346	2.877	2023	0.152945818	isogroup08279	ENSG00000135114	ENST00000257570	OASL
isotig23189	5.652	4.662	5.044	3.423	2021	0.481419554	isogroup08280	ENSG00000145293	ENST00000273920	ENOPH1
isotig23190	7.892	8.727	3.169	4.362	2022	0.433719095	isogroup08281	ENSG00000164292	ENST00000379982	RHOBTB3
isotig23191	6.045	3.699	6.292	4.75	2022	0.554172616	isogroup08282	ENSG00000101216	ENST00000370077	GMEB2
isotig23192	7.052	4.773	8.721	8.06	2022	0.323842714	isogroup08283	ENSG00000146834	ENST00000310512	MEPCE
isotig23193	1.304	1.939	2.055	1.661	2022	0.166247088	isogroup08284	ENSG00000107815	ENST00000311916	C10orf2
isotig23194	6.608	4.697	6.593	6.167	2022	0.348444428	isogroup08285	ENSG00000167962	ENST00000431526	ZNF598
isotig23195	1.57	3.041	3.735	3.554	2022	0.206009995	isogroup08286	ENSG00000141504	ENST00000269298	SAT2
isotig23196	3.004	3.098	2.905	1.839	2021	0.248656722	isogroup08287	ENSG00000095002	ENST00000543555	MSH2
isotig23197	2.431	3.438	3.371	2.924	2021	0.205596678	isogroup08288	ENSG00000163155	ENST00000368908	LYSMD1
isotig23198	2.56	3.091	2.669	3.591	2020	0.366038551	isogroup08289	ENSG00000064652	ENST00000261369	SNX24
isotig23199	4.501	3.714	4.033	3.903	2019	0.006660029	isogroup08290	ENSG00000080603	ENST00000395059	SRCAP
isotig23202	1.772	3.028	4.3	3.661	2018	0.079835801	isogroup08293	ENSG00000171793	ENST00000543104	CTPS
isotig23203	1.871	3.217	4.154	4.071	2018	0.208020215	isogroup08294	ENSG00000162572	ENST00000379101	SCNN1D
isotig23204	5.017	4.471	5.481	3.938	2018	0.514400316	isogroup08295	ENSG00000162601	ENST00000472487	MYSM1
isotig23207	7.142	2.984	4.552	3.631	2016	0.696268881	isogroup08298	ENSG00000065970	ENST00000428177	FOXJ2
isotig23208	2.993	2.588	1.915	2.222	2015	0.010060494	isogroup08299	ENSG00000155304	ENST00000285667	HSPA13
isotig23209	3.717	2.475	3.904	2.867	2015	0.440444879	isogroup08300	ENSG00000184682	ENST00000391480	C11orf89
isotig23210	1.537	2.731	2.989	3.168	2014	0.263179154	isogroup08301	ENSG00000143942	ENST00000295304	CHAC2
isotig23211	29.53	23.24	33.464	43.04	2014	0.164988352	isogroup08302	ENSG00000228672	ENST00000434752	C5orf65
isotig23212	4.859	5.151	4.355	4.246	2014	0.067426918	isogroup08303	ENSG00000080802	ENST00000423368	CNOT4
isotig23214	14.73	11.508	12.929	12.866	2008	0.323232133	isogroup08305	ENSG00000204899	ENST00000377818	MZT1
isotig23215	7.378	4.193	6.418	4.876	2011	0.598942286	isogroup08306	ENSG00000126890	ENST00000369585	CTAG2
isotig23216	2.984	3.735	2.933	3.266	2008	0.337115804	isogroup08307	ENSG00000135090	ENST00000419821	TAOK3
isotig23218	5.45	2.948	3.347	2.174	2008	0.6252912	isogroup08309	ENSG00000213121	ENST00000392385	AL590867.1
isotig23219	7.889	7.59	7.141	10.559	2010	0.329732472	isogroup08310	ENSG00000170633	ENST00000392465	RNF34
isotig23222	2.862	3.117	2.922	2.515	2003	0.046479297	isogroup08313	ENSG00000106344	ENST00000415472	RBM28
isotig23223	3.524	2.306	2.946	2.846	2006	0.321475539	isogroup08314	ENSG00000168096	ENST00000304283	ANKS3
isotig23225	1.932	2.449	2.091	2.724	2005	0.358880664	isogroup08316	ENSG00000107938	ENST00000356792	C10orf137
isotig23226	1.304	1.917	2.706	2.556	2006	0.034718569	isogroup08317	ENSG00000167978	ENST00000301740	SRRM2
isotig23227	12.234	15.851	14.501	10.461	2003	0.200120237	isogroup08318	ENSG00000117519	ENST00000538964	CNN3
isotig23228	3.735	3.664	3.407	2.914	2005	0.006660029	isogroup08319	ENSG00000197381	ENST00000539173	ADARB1
isotig23229	15.23	12.604	16.499	14.947	2001	0.245162321	isogroup08320	ENSG00000133313	ENST00000324262	CNDP2
isotig23230	15.728	27.445	35.484	33.457	2005	0.19380777	isogroup08321	ENSG00000173372	ENST00000339353	C1QA
isotig23232	3.59	4.124	3.411	3.442	2003	0.256209138	isogroup08323	ENSG00000143554	ENST00000368661	SLC27A3
isotig23233	4.783	2.484	5.973	3.246	2000	0.701275644	isogroup08324	ENSG00000213901	ENST00000455516	SLC23A3
isotig23234	1.847	2.959	3.34	2.987	2001	0.094423987	isogroup08325	ENSG00000149256	ENST00000530738	ODZ4
isotig23235	11.226	10.337	9.39	8.984	1996	0.17979259	isogroup08326	ENSG00000134001	ENST00000466499	EIF2S1
isotig23236	37.126	28.332	38.84	30.659	2000	0.4685316	isogroup08327	ENSG00000214753	ENST00000301785	HNRNPUL2
isotig23237	23.526	27.599	25.916	24.827	2000	0.147666642	isogroup08328	ENSG00000109519	ENST00000264954	GRPEL1
isotig23238	2.64	2.325	2.496	1.872	1997	0.253485008	isogroup08329	ENSG00000145414	ENST00000274054	NAF1
isotig23239	4.214	3.559	3.952	3.896	2000	0.22511648	isogroup08330	ENSG00000168591	ENST00000538716	TMUB2
isotig23240	9.016	5.697	6.7	5.121	1998	0.559254528	isogroup08331	ENSG00000176209	ENST00000490331	C8orf40
isotig23243	2.977	1.79	1.506	1.747	1999	0.122106786	isogroup08334	ENSG00000137574	ENST00000260129	TGS1
isotig23244	4.658	20.759	6.072	3.574	1998	0.944643796	isogroup08335	ENSG00000130635	ENST00000371817	COL5A1
isotig23245	7.342	4.133	5.824	2.829	1994	0.73582513	isogroup08336	ENSG00000179938	ENST00000567927	GOLGA8J
isotig23247	5.879	5.164	4.232	4.682	1994	0.095861201	isogroup08338	ENSG00000108064	ENST00000487519	TFAM
isotig23248	4.308	3.42	2.819	2.353	1998	0.365878861	isogroup08339	ENSG00000110583	ENST00000377793	NAA40
isotig23249	10.751	6.929	7.249	5.997	1997	0.515461787	isogroup08340	ENSG00000001036	ENST00000002165	FUCA2
isotig23250	4.399	4.546	2.011	1.134	1993	0.313021342	isogroup08341	ENSG00000138639	ENST00000395183	ARHGAP24
isotig23251	3.292	2.661	2.747	1.929	1996	0.345513639	isogroup08342	ENSG00000172262	ENST00000509634	ZNF131
isotig23253	5.765	4.768	4.857	3.338	1992	0.4138987	isogroup08344	ENSG00000169621	ENST00000303795	APLF
isotig23254	2.181	2.918	1.979	2.209	1996	0.356607425	isogroup08345	ENSG00000099954	ENST00000400585	CECR2
isotig23255	2.943	1.613	8.885	6.295	1995	0.609491245	isogroup08346	ENSG00000176978	ENST00000443858	DPP7
isotig23257	9.147	5.933	7.659	5.414	1989	0.59924288	isogroup08348	ENSG00000124784	ENST00000379834	RIOK1
isotig23258	1.413	1.413	1.446	1.303	1993	0.080145788	isogroup08349	ENSG00000119509	ENST00000262457	INVS
isotig23259	3.019	1.778	2.949	2.373	1991	0.44909634	isogroup08350	ENSG00000137261	ENST00000543707	KIAA0319
isotig23261	6.553	5.557	9.418	6.581	1990	0.403199444	isogroup08352	ENSG00000168496	ENST00000305885	FEN1
isotig23263	8.181	10.874	9.205	10.396	1992	0.375037574	isogroup08354	ENSG00000124788	ENST00000450222	ATXN1
isotig23264	1.163	2.513	2.892	3.63	1992	0.542768843	isogroup08355	ENSG00000179588	ENST00000319555	ZFPM1
isotig23265	3.934	2.927	3.6	3.341	1990	0.270534305	isogroup08356	ENSG00000157191	ENST00000492095	NECAP2
isotig23267	6.885	6.456	6.254	4.721	1987	0.24399752	isogroup08358	ENSG00000179163	ENST00000374479	FUCA1
isotig23268	6.701	5.813	5.861	5.723	1991	0.228667243	isogroup08359	ENSG00000100023	ENST00000492445	PPIL2
isotig23269	3.814	4.189	4.306	3.971	1990	0.074284211	isogroup08360	ENSG00000198026	ENST00000426788	ZNF335
isotig23270	10.628	9.402	5.889	4.718	1988	0.227183062	isogroup08361	ENSG00000066056	ENST00000433781	TIE1
isotig23271	2.159	3.838	4.809	3.984	1989	0.09609604	isogroup08362	ENSG00000100038	ENST00000398793	TOP3B
isotig23272	1.667	3.167	4.242	4.029	1989	0.186405651	isogroup08363	ENSG00000121897	ENST00000340169	LIAS
isotig23273	7.982	5.512	6.34	5.155	1989	0.550030059	isogroup08364	ENSG00000150403	ENST00000434316	TMCO3

isotig23274	3.484	3.493	2.618	3.095	1989	0.296479297	isogroup08365	ENSG00000067141	ENST00000379842	NEO1
isotig23275	4.442	4.388	4.646	2.332	1987	0.421084767	isogroup08366	ENSG00000215910	ENST00000449278	C1orf167
isotig23276	2.985	2.951	3.447	1.78	1987	0.4133163	isogroup08367	ENSG00000188906	ENST00000298910	LRRK2
isotig23278	30.511	21.128	29.505	28.418	1982	0.366029158	isogroup08369	ENSG00000102181	ENST00000466436	CD99L2
isotig23279	2.826	2.779	2.268	1.77	1986	0.133181784	isogroup08370	ENSG00000139746	ENST00000438737	RBM26
isotig23281	18.794	14.422	16.307	12.677	1985	0.575758999	isogroup08372	ENSG00000090674	ENST00000264079	MCOLN1
isotig23282	9.099	5.071	6.996	4.947	1982	0.671498084	isogroup08373	ENSG00000167842	ENST00000381165	MIS12
isotig23283	20.313	19.351	18.893	21.202	1984	0.161475163	isogroup08374	ENSG00000160310	ENST00000397637	PRMT2
isotig23284	10.601	6.409	9.335	6.7	1978	0.599487112	isogroup08375	ENSG00000143570	ENST00000429040	SLC39A1
isotig23286	5.212	5.16	4.381	4.859	1981	0.068676261	isogroup08377	ENSG00000215305	ENST00000380445	VPS16
isotig23287	4.97	2.998	6.459	3.221	1981	0.717432554	isogroup08378	ENSG00000131591	ENST00000421241	C1orf159
isotig23290	2.876	3.915	4.945	3.654	1979	0.053402345	isogroup08381	ENSG00000134463	ENST00000379215	ECHDC3
isotig23292	6.257	5.146	4.506	3.19	1979	0.350943113	isogroup08383	ENSG00000111145	ENST00000228741	ELK3
isotig23293	1.574	2.818	3.62	3.608	1979	0.222739911	isogroup08384	ENSG00000029639	ENST00000367166	TFB1M
isotig23294	51.214	47.744	39.388	46.561	1979	0.120218682	isogroup08385	ENSG00000162734	ENST00000368076	PEA15
isotig23296	9.602	9.344	11.482	7.776	1978	0.398023597	isogroup08387	ENSG00000167978	ENST00000544933	SRRM2
isotig23298	4.829	6.056	5.959	5.107	1977	0.098782596	isogroup08389	ENSG00000171861	ENST00000304478	RNMTL1
isotig23299	2.224	3.391	2.897	3.172	1976	0.239451041	isogroup08390	ENSG00000163686	ENST00000478253	ABHD6
isotig23302	1.913	2.25	1.966	1.437	1963	0.108307658	isogroup08393	ENSG00000196437	ENST00000392149	ZNF569
isotig23303	1.193	2.196	2.841	2.662	1976	0.203548884	isogroup08394	ENSG00000117983	ENST00000529681	MUC5B
isotig23304	2.846	2.693	2.996	2.838	1972	0.108279477	isogroup08395	ENSG00000198746	ENST00000361720	GPATCH3
isotig23305	13.601	6.38	10.387	5.815	1975	0.821597655	isogroup08396	ENSG00000087074	ENST00000200453	PPP1R15A
isotig23307	2.46	2.152	2.499	1.418	1973	0.310879612	isogroup08398	ENSG00000223766	ENST00000549475	PRR3
isotig23309	10.417	9.774	16.031	10.082	1965	0.686856542	isogroup08400	ENSG00000128016	ENST00000248673	ZFP36
isotig23310	12.754	14.76	10.51	10.643	1973	0.124633651	isogroup08401	ENSG00000166260	ENST00000299335	COX11
isotig23311	8.039	12.058	8.96	7.795	1970	0.481560457	isogroup08402	ENSG00000135540	ENST00000427025	NHSL1
isotig23315	4.567	5.15	4.691	5.818	1972	0.277927031	isogroup08406	ENSG00000130939	ENST00000343090	UBE4B
isotig23318	7.411	4.58	7.677	6.455	1970	0.561912903	isogroup08409	ENSG00000117411	ENST00000372324	B4GALT2
isotig23319	3.338	2.26	3.9	3.723	1969	0.15215676	isogroup08410	ENSG00000213121	ENST00000392385	AL590867.1
isotig23320	12.626	12.45	13.094	11.176	1963	0.125563613	isogroup08411	ENSG00000134453	ENST00000446108	RBM17
isotig23321	2.514	4.512	7.039	4.829	1970	0.267509581	isogroup08412	ENSG00000104368	ENST00000429089	PLAT
isotig23322	5.889	4.8	4.891	4.143	1967	0.351920042	isogroup08413	ENSG00000050426	ENST00000262055	LETMD1
isotig23323	14.717	12.538	16.148	13.143	1969	0.364197415	isogroup08414	ENSG00000133612	ENST00000473312	AGAP3
isotig23325	1.961	4.669	4.811	7.075	1965	0.624652439	isogroup08416	ENSG00000203747	ENST00000540048	FCGR3A
isotig23326	1.441	2.476	3.49	3.117	1968	0.064064026	isogroup08417	ENSG00000115705	ENST00000469607	TPO
isotig23327	5.198	4.819	4.553	5.269	1967	0.159765537	isogroup08418	ENSG00000025800	ENST00000373625	KPNA6
isotig23328	2.916	2.227	2.695	2.425	1966	0.256669422	isogroup08419	ENSG00000241258	ENST00000395326	CRCP
isotig23329	5.959	5.379	5.208	5.095	1964	0.137305553	isogroup08420	ENSG00000103671	ENST00000261884	TRIP4
isotig23330	6.115	3.776	7.401	4.498	1966	0.718221613	isogroup08421	ENSG00000102878	ENST00000521374	HSF4
isotig23331	5.995	7.887	6.964	7.462	1962	0.393533479	isogroup08422	ENSG00000170502	ENST00000302174	NUDT9
isotig23332	3.464	2.517	4.47	2.847	1966	0.533628917	isogroup08423	ENSG00000100297	ENST00000216122	MCM5
isotig23333	2.815	3.546	3.693	2.13	1965	0.059555121	isogroup08424	ENSG00000087460	ENST00000306120	GNAS
isotig23334	1.406	3.088	1.357	1.451	1960	0.518232885	isogroup08425	ENSG00000123342	ENST00000322569	MMP19
isotig23335	2.053	4.689	3.674	4.249	1965	0.574819644	isogroup08426	ENSG00000156345	ENST00000325303	CDK20
isotig23336	18.304	10.132	11.342	6.084	1965	0.874107613	isogroup08427	ENSG00000172890	ENST00000527852	NADSYN1
isotig23338	1.759	2.53	3.347	3.283	1964	0.084401067	isogroup08429	ENSG00000117983	ENST00000529681	MUC5B
isotig23340	55.559	51.979	57.653	48.394	1954	0.213506049	isogroup08431	ENSG00000067560	ENST00000418115	RHOA
isotig23341	2.268	2.714	2.125	1.869	1964	0.084617119	isogroup08432	ENSG00000122376	ENST00000298786	FAM35A
isotig23342	29.636	27.404	81.442	26.648	1965	0.805468926	isogroup08433	ENSG00000159176	ENST00000532460	CSRP1
isotig23343	18.465	19.82	19.368	18.008	1963	0.054003532	isogroup08434	ENSG00000011485	ENST00000012443	PPP5C
isotig23344	52.492	54.654	53.643	39.817	1951	0.289415345	isogroup08435	ENSG00000166226	ENST00000299300	CCT2
isotig23345	3.096	3.436	3.87	4.361	1961	0.176147892	isogroup08436	ENSG00000167280	ENST00000545583	ENGASE
isotig23346	4.033	2.553	2.853	2.215	1961	0.437326219	isogroup08437	ENSG00000067369	ENST00000382044	TP53BP1
isotig23348	2.591	2.207	2.622	2.115	1958	0.18122041	isogroup08439	ENSG00000147601	ENST00000276602	TERF1
isotig23349	5.426	8.477	6.026	5.945	1958	0.446156158	isogroup08440	ENSG00000250021	ENST00000398333	C15orf38-AP3S2
isotig23350	60.809	52.053	70.147	65.413	1960	0.305281055	isogroup08441	ENSG00000174903	ENST00000311481	RAB1B
isotig23351	3.984	4.691	4.296	3.335	1960	0.042524611	isogroup08442	ENSG00000099899	ENST00000403707	TRMT2A
isotig23352	2.1	4.362	3.016	2.731	1959	0.610806343	isogroup08443	ENSG00000134247	ENST00000544471	PTGFRN
isotig23353	3.558	3.586	2.649	3.462	1959	0.220175472	isogroup08444	ENSG00000154309	ENST00000284476	DISP1
isotig23355	2.856	2.897	2.999	2.87	1958	0.035704892	isogroup08446	ENSG00000126883	ENST00000451030	NUP214
isotig23358	9.465	12.132	13.872	11.948	1958	0.08237206	isogroup08449	ENSG00000141084	ENST00000317506	RANBP10
isotig23359	14.491	6.711	6.171	10.524	1957	0.407407755	isogroup08450	ENSG00000133226	ENST00000323848	SRRM1
isotig23360	3.919	4.396	3.529	3.981	1957	0.215037198	isogroup08451	ENSG00000174684	ENST00000311181	B3GNT1
isotig23361	2.052	3.61	4.73	4.624	1956	0.186330503	isogroup08452	ENSG00000151093	ENST00000280701	OXSM
isotig23363	5.84	3.951	4.994	3.182	1956	0.624652439	isogroup08454	ENSG00000060069	ENST00000299543	CTDP1
isotig23364	1.47	2.277	2.577	2.919	1956	0.256209138	isogroup08455	ENSG00000151413	ENST00000281081	NUBPL
isotig23365	1.983	2.104	1.276	1.226	1954	0.07293154	isogroup08456	ENSG00000134444	ENST00000256858	KIAA1468
isotig23367	18.652	32.757	38.868	29.127	1953	0.179548358	isogroup08458	ENSG00000188199	ENST00000448135	FAM22B
isotig23368	7.233	9.654	7.208	6.365	1952	0.199453295	isogroup08459	ENSG00000142657	ENST00000270776	PGD
isotig23371	3.142	1.814	2.868	2.215	1951	0.469348839	isogroup08462	ENSG00000103168	ENST00000567759	TAF1C
isotig23372	18.954	12.347	15.682	12.172	1944	0.644331931	isogroup08463	ENSG00000117748	ENST00000373909	RPA2
isotig23373	16.853	14.087	18.247	19.857	1947	0.038851732	isogroup08464	ENSG00000176946	ENST00000407315	THAP4
isotig23374	4.553	4.349	5.645	4.551	1943	0.144688886	isogroup08465	ENSG00000244045	ENST00000292114	TMEM199
isotig23375	4.959	4.043	4.87	4.411	1949	0.259665965	isogroup08466	ENSG00000166847	ENST00000300087	DCTN5
isotig23377	3.002	2.373	3.117	3.113	1949	0.169271812	isogroup08468	ENSG00000037637	ENST00000375592	FBXO42
isotig23378	7.453	8.354	7.384	8.309	1948	0.200533554	isogroup08469	ENSG00000100106	ENST00000403663	TRIOBP
isotig23379	2.205	2.825	1.347	1.483	1947	0.481626212	isogroup08470	ENSG00000197380	ENST00000391916	DACT3
isotig23380	11.867	11.976	14.652	9.635	1947	0.268890434	isogroup08471	ENSG00000100600	ENST00000393218	LGMN
isotig23382	1.488	2.524	3.179	3.313	1946	0.17765086	isogroup08473	ENSG00000117682	ENST00000360009	DHDDS
isotig23384	1.297	2.265	3	1.915	1945	0.010060494	isogroup08475	ENSG00000230344	ENST00000535339	AC007308.1
isotig23385	40.979	29.031	66.441	43.081	1944	0.658882543	isogroup08476	ENSG00000086589	ENST00000540000	RBM22
isotig23386	6.479	5.637	6.076	5.824	1945	0.25415195	isogroup08477	ENSG00000146576	ENST00000344417	C7orf26

isotig23388	1.748	2.636	1.731	1.487	1942	0.205822124	isogroup08479	ENSG00000122482	ENST00000370440	ZNF644
isotig23390	6.705	3.611	5.506	3.925	1945	0.603000301	isogroup08481	ENSG00000109501	ENST00000503569	WFS1
isotig23391	9.965	11.485	13.279	11.285	1944	0.110646652	isogroup08482	ENSG00000006712	ENST00000221265	PAF1
isotig23393	4.502	3.154	4.828	3.825	1943	0.499699406	isogroup08484	ENSG00000104907	ENST00000437766	TRMT1
isotig23394	53.157	70.129	59.644	57.627	1942	0.228798753	isogroup08485	ENSG00000106049	ENST00000265395	HIBADH
isotig23396	9.564	6.887	7.365	8.244	1943	0.254302247	isogroup08487	ENSG00000140632	ENST00000321919	GLYR1
isotig23400	22.868	11.124	16.72	15.545	1937	0.641786278	isogroup08491	ENSG00000070010	ENST00000263202	UFD1L
isotig23401	8.525	3.783	4.583	3.666	1942	0.719179755	isogroup08492	ENSG00000148358	ENST00000372406	GPR107
isotig23403	2.76	3.229	2.419	2.24	1939	0.05706583	isogroup08494	ENSG00000112685	ENST00000448181	EXOC2
isotig23404	1.964	3.127	4.193	4.046	1942	0.082137221	isogroup08495	ENSG00000167978	ENST00000426305	SRRM2
isotig23405	6.074	4.455	5.987	5.435	1940	0.389653942	isogroup08496	ENSG00000120055	ENST00000239125	C10orf95
isotig23406	3.43	3.86	4.079	3.347	1933	0.003503795	isogroup08497	ENSG00000113456	ENST00000382038	RAD1
isotig23409	8.2	6.227	3.643	4.648	1941	0.379715563	isogroup08500	ENSG00000113240	ENST00000522556	CLK4
isotig23410	2.593	1.344	2.207	1.522	1941	0.459767416	isogroup08501	ENSG00000196449	ENST00000373044	YRDC
isotig23411	21.708	22.502	24.878	31.68	1932	0.071259487	isogroup08502	ENSG00000002549	ENST00000226299	LAP3
isotig23412	37.254	19.712	37.282	24.2	1939	0.783920117	isogroup08503	ENSG00000220205	ENST00000488857	VAMP2
isotig23414	2.606	3.769	1.828	3.948	1939	0.565736079	isogroup08505	ENSG00000138756	ENST00000502613	BMP2K
isotig23416	3.317	2.866	3.781	3.498	1939	0.157802285	isogroup08507	ENSG00000197323	ENST00000369543	TRIM33
isotig23418	2.204	3.583	4.99	4.133	1939	0.027992786	isogroup08509	ENSG00000144381	ENST00000388968	HSPD1
isotig23419	20.084	15.565	17.025	13.21	1937	0.602455475	isogroup08510	ENSG00000100393	ENST00000263253	EP300
isotig23420	4.677	3.087	4.42	3.967	1930	0.41347599	isogroup08511	ENSG00000188095	ENST00000341735	MESP2
isotig23421	1.35	2.249	3.163	2.599	1938	0.021652138	isogroup08512	ENSG00000198650	ENST00000355962	TAT
isotig23422	3.289	2.555	2.831	2.6	1937	0.263686406	isogroup08513	ENSG00000172936	ENST00000396334	MYD88
isotig23423	3.346	3.591	3.303	3.173	1940	0.072142481	isogroup08514	ENSG00000115750	ENST00000263663	TAF1B
isotig23424	4.544	1.846	4.287	3.57	1938	0.622538889	isogroup08515	ENSG00000147130	ENST00000373998	ZMYM3
isotig23426	1.68	1.759	1.392	1.676	1937	0.1098482	isogroup08517	ENSG00000163328	ENST00000392551	GPR155
isotig23427	5.066	2.975	2.898	3.358	1937	0.322123694	isogroup08518	ENSG00000171843	ENST00000380338	MLLT3
isotig23428	4.435	5.181	4.855	4.683	1936	0.217629819	isogroup08519	ENSG00000164902	ENST00000297540	PHAX
isotig23429	3.755	2.242	3.338	2.301	1936	0.534361614	isogroup08520	ENSG00000215529	ENST00000400522	EFCAB8
isotig23430	3.951	3.244	3.367	3.619	1936	0.172428045	isogroup08521	ENSG00000002919	ENST00000393405	SNX11
isotig23432	2.957	3.29	3.269	2.375	1932	0.038353874	isogroup08523	ENSG00000128708	ENST00000264108	HAT1
isotig23434	5.713	6.332	4.948	4.563	1933	0.006660029	isogroup08525	ENSG00000124356	ENST00000409707	STAMBP
isotig23435	10.296	3.136	8.352	4.663	1930	0.874210942	isogroup08526	ENSG00000241713	ENST00000455499	LY6G5B
isotig23436	5.716	6.049	6.088	7.729	1932	0.255391899	isogroup08527	ENSG00000099940	ENST00000215730	SNAP29
isotig23437	3.595	3.417	5.416	3.671	1933	0.421084767	isogroup08528	ENSG00000011451	ENST00000545156	WIZ
isotig23438	1.583	2.099	1.998	1.136	1931	0.034718569	isogroup08529	ENSG00000109466	ENST00000538127	KLHL2
isotig23439	4.3	2.305	2.772	1.831	1932	0.578915233	isogroup08530	ENSG00000130787	ENST00000253083	HIP1R
isotig23441	6.671	7.373	6.35	4.858	1931	0.033563162	isogroup08532	ENSG00000106617	ENST00000492843	PRKAG2
isotig23442	4.548	3.714	4.903	2.514	1932	0.500807845	isogroup08533	ENSG00000129473	ENST00000556599	BCL2L2
isotig23444	2.804	2.476	2.784	1.612	1930	0.233533103	isogroup08535	ENSG00000110876	ENST00000550948	SELPLG
isotig23446	28.875	17.466	23.461	20.89	1929	0.589097843	isogroup08537	ENSG00000107331	ENST00000371605	ABCA2
isotig23589	3.913	13.424	2.363	3.448	1883	0.883952055	isogroup08680	ENSG00000120833	ENST00000549122	SOCS2
isotig23590	5.463	3.89	4.216	3.563	1879	0.331263621	isogroup08681	ENSG00000147383	ENST00000440023	NSDHL
isotig23591	6.703	6.611	10.542	3.578	1883	0.638986999	isogroup08682	ENSG00000157227	ENST00000311852	MMP14
isotig23592	11.95	16.222	10.764	11.331	1875	0.372360412	isogroup08683	ENSG00000164022	ENST00000394701	AIMP1
isotig23593	37.809	26.65	37.97	28.537	1882	0.59712933	isogroup08684	ENSG00000141522	ENST00000541078	ARHGDIA
isotig23594	2.102	2.388	1.708	1.44	1881	0.010060494	isogroup08685	ENSG00000205189	ENST00000519370	ZBTB10
isotig23595	7.379	7.539	4.142	4.844	1881	0.217676787	isogroup08686	ENSG00000109670	ENST00000296555	FBXW7
isotig23597	55.362	19.602	35.327	24.199	1878	0.901320733	isogroup08688	ENSG00000111684	ENST00000261407	LPCAT3
isotig23598	5.898	8.32	6.273	6.001	1869	0.294572406	isogroup08689	ENSG00000165416	ENST00000310528	SUGT1
isotig23599	3.176	3.624	2.966	2.763	1880	0.145975802	isogroup08690	ENSG00000112297	ENST00000535438	AIM1
isotig23600	3.773	4.062	3.902	4.658	1879	0.295699632	isogroup08691	ENSG00000119965	ENST00000481909	C10orf88
isotig23601	1.487	2.329	3.172	3.001	1880	0.146783648	isogroup08692	ENSG00000124228	ENST00000371764	DDX27
isotig23605	15.486	12.652	12.647	12.164	1879	0.213449688	isogroup08696	ENSG00000137714	ENST00000260270	FDX1
isotig23607	2.733	5.341	6.322	6.318	1878	0.287283009	isogroup08698	ENSG00000178605	ENST00000400701	GTPBP6
isotig23608	1.488	3.393	1.533	1.275	1879	0.474214699	isogroup08699	ENSG00000055813	ENST00000407862	CCDC85A
isotig23611	8.358	7.328	8.534	6.687	1878	0.27945818	isogroup08702	ENSG00000172725	ENST00000393893	CORO1B
isotig23612	8.768	9.456	9.805	7.889	1870	0.093212219	isogroup08703	ENSG00000153130	ENST00000394201	SCOC
isotig23614	7.258	11.096	6.287	6.357	1877	0.487309311	isogroup08705	ENSG00000185973	ENST00000369439	TMLHE
isotig23615	4.199	1.412	2.148	1.147	1874	0.711984259	isogroup08706	ENSG00000120370	ENST00000367763	GORAB
isotig23616	2.408	10.828	4.158	8.41	1874	0.923066807	isogroup08707	ENSG00000163710	ENST00000295992	PCOLCE2
isotig23619	8.059	7.072	8.406	6.408	1876	0.383256932	isogroup08710	ENSG00000136271	ENST00000258772	DDX56
isotig23621	11.49	8.277	12.951	10.882	1872	0.487666266	isogroup08712	ENSG00000132792	ENST00000361383	CTNNBL1
isotig23622	8.358	7.427	6.869	6.593	1871	0.131631848	isogroup08713	ENSG00000066855	ENST00000518800	MTFR1
isotig23623	3.762	5.139	4.729	5.758	1874	0.398136319	isogroup08714	ENSG00000158850	ENST00000367998	B4GALT3
isotig23625	7.581	8.618	7.818	6.813	1873	0.131631848	isogroup08716	ENSG00000185339	ENST00000215838	TCN2
isotig23626	6.557	6.077	6.398	6.335	1873	0.045878109	isogroup08717	ENSG00000026508	ENST00000360158	CD44
isotig23627	6.298	7.233	6.602	4.75	1871	0.032651988	isogroup08718	ENSG00000213977	ENST00000225525	TAX1BP3
isotig23628	3.677	3.596	3.636	2.544	1874	0.09609604	isogroup08719	ENSG00000119537	ENST00000406396	KDSR
isotig23629	3.207	5.238	9.529	6.376	1868	0.28818479	isogroup08720	ENSG00000198825	ENST00000361976	INPP5F
isotig23634	1.302	2.554	3.188	3.123	1871	0.150381378	isogroup08725	ENSG00000145439	ENST00000504561	CBR4
isotig23635	6.596	5.148	7.421	5.611	1871	0.477577591	isogroup08726	ENSG00000068438	ENST00000456787	FTSJ1
isotig23636	1.709	2.993	3.782	3.436	1870	0.21826858	isogroup08727	ENSG00000166133	ENST00000417769	RPUSD2
isotig23637	7.679	1.476	5.312	3.412	1870	0.867250319	isogroup08728	ENSG00000066735	ENST00000423312	KIF26A
isotig23638	8.554	7.03	7.929	7.005	1863	0.337651236	isogroup08729	ENSG00000122958	ENST00000373382	VPS26A
isotig23640	1.282	2.28	3.101	3.089	1870	0.187579845	isogroup08731	ENSG00000183248	ENST00000539422	AC010336.1
isotig23641	3.697	2.292	2.608	2.264	1869	0.347044788	isogroup08732	ENSG00000167566	ENST00000433948	NCKAP5L
isotig23642	1.549	2.75	3.448	3.558	1869	0.142387465	isogroup08733	ENSG00000170004	ENST00000439235	CHD3
isotig23643	1.625	3.159	3.45	3.571	1868	0.242062448	isogroup08734	ENSG00000143631	ENST00000392689	FLG
isotig23644	1.512	2.554	3.419	3.509	1863	0.160639137	isogroup08735	ENSG00000171174	ENST00000302188	RBKS
isotig23646	13.697	10.253	10.462	8.729	1862	0.447583978	isogroup08737	ENSG00000086065	ENST00000223500	CHMP5
isotig23647	5.787	4.751	5.341	4.448	1867	0.31349102	isogroup08738	ENSG00000140400	ENST00000563622	MAN2C1

isotig23648	6.468	4.429	6.277	3.637	1865	0.602352145	isogroup08739	ENSG00000129484	ENST00000250416	PARP2
isotig23649	14.766	12.141	23.803	12.017	1861	0.69951905	isogroup08740	ENSG00000103642	ENST00000261893	LACTB
isotig23653	32.126	20.133	21.599	19.554	1854	0.545089051	isogroup08744	ENSG00000163866	ENST00000521580	C1orf212
isotig23654	8.247	6.096	6.903	5.803	1867	0.517368678	isogroup08745	ENSG00000124151	ENST00000371998	NCOA3
isotig23655	1.776	2.773	3.58	2.986	1867	0.017143233	isogroup08746	ENSG00000170445	ENST00000504156	HARS
isotig23658	3.136	2.213	1.989	3.887	1866	0.270910047	isogroup08749	ENSG00000007047	ENST00000300843	MARK4
isotig23660	2.313	3.144	2.089	3.517	1865	0.514710303	isogroup08751	ENSG00000116285	ENST00000377482	ERRFI1
isotig23661	2.807	2.369	1.595	1.117	1864	0.214736605	isogroup08752	ENSG00000046889	ENST00000288368	PREX2
isotig23664	1.457	2.89	4.081	3.242	1864	0.118715714	isogroup08755	ENSG00000105409	ENST00000545399	ATP1A3
isotig23666	4.352	3.801	4.113	3.641	1863	0.170314496	isogroup08757	ENSG00000150760	ENST00000280333	DOCK1
isotig23668	34.543	31.088	35.677	26.726	1857	0.368321184	isogroup08759	ENSG00000150753	ENST00000280326	CCT5
isotig23669	1.995	3.404	4.73	4.545	1862	0.137606147	isogroup08760	ENSG00000141179	ENST00000417982	PCTP
isotig23670	17.225	15.037	17.007	18.359	1862	0.100229203	isogroup08761	ENSG00000158109	ENST00000378344	TPRG1L
isotig23671	2.453	2.256	2.079	1.954	1856	0.084617119	isogroup08762	ENSG00000159596	ENST00000372025	TMEM69
isotig23672	4.722	3.314	3.105	3.801	1861	0.017143233	isogroup08763	ENSG00000064393	ENST00000406875	HIPK2
isotig23674	3.745	3.6	3.691	3.609	1861	0.084044112	isogroup08765	ENSG00000198951	ENST00000402937	NAGA
isotig23676	15.452	9.65	10.834	8.829	1856	0.579751259	isogroup08767	ENSG00000136045	ENST00000412830	PWP1
isotig23681	1.697	2.949	3.814	3.673	1859	0.145271286	isogroup08772	ENSG00000005471	ENST00000545634	ABCB4
isotig23683	9.427	5.874	6.81	3.863	1860	0.596218156	isogroup08774	ENSG00000164181	ENST00000508821	ELOVL7
isotig23684	67.347	68.601	62.377	54.384	1859	0.105386263	isogroup08775	ENSG00000135624	ENST00000258091	CCT7
isotig23685	2.757	3.012	2.544	2.868	1858	0.087162771	isogroup08776	ENSG00000197375	ENST00000435065	SLC22A5
isotig23686	4.278	2.53	3.29	2.121	1858	0.472749305	isogroup08777	ENSG00000105287	ENST00000433867	PRKD2
isotig23692	1.098	1.508	1.065	1.098	1856	0.137840986	isogroup08783	ENSG00000256683	ENST00000243644	ZNF350
isotig23693	4.888	4.935	2.986	2.691	1855	0.153725483	isogroup08784	ENSG00000107798	ENST00000541980	LIPA
isotig23695	2.142	2.104	3.026	2.583	1855	0.136507102	isogroup08786	ENSG00000198342	ENST00000438182	ZNF442
isotig23696	20.111	10.621	18.445	12.963	1853	0.748844593	isogroup08787	ENSG00000108465	ENST00000536708	CDK5RAP3
isotig23697	3.731	2.189	2.164	1.789	1852	0.383022094	isogroup08788	ENSG00000153902	ENST00000392225	LGI4
isotig23698	2.933	1.994	2.702	1.596	1852	0.460997971	isogroup08789	ENSG00000120800	ENST00000261637	UTP20
isotig23699	2.635	4.149	1.798	1.913	1849	0.506622454	isogroup08790	ENSG00000033170	ENST00000557164	FUT8
isotig23700	2.263	4.571	5.092	4.676	1853	0.279570903	isogroup08791	ENSG00000167325	ENST00000536894	RRM1
isotig23701	22.846	18.766	28.846	21.115	1848	0.553468099	isogroup08792	ENSG00000111652	ENST00000539735	COPS7A
isotig23702	2.209	6.338	3.996	2.247	1853	0.593494026	isogroup08793	ENSG00000179262	ENST00000316856	RAD23A
isotig23703	2.209	2.404	3.308	2.368	1853	0.122942812	isogroup08794	ENSG00000164088	ENST00000457351	PPM1M
isotig23704	3.397	4.018	4.477	3.409	1852	0.017143233	isogroup08795	ENSG00000198189	ENST00000358290	HSD17B11
isotig23706	6.97	3.579	2.733	2.346	1851	0.603742391	isogroup08797	ENSG00000123094	ENST00000542865	RASSF8
isotig23709	6.506	3.023	6.993	5.696	1851	0.653847599	isogroup08800	ENSG00000148832	ENST00000278060	PAOX
isotig23710	5.914	4.504	4.129	4.351	1847	0.216605922	isogroup08801	ENSG00000135052	ENST00000388711	GOLM1
isotig23711	3.636	3.692	4.14	3.701	1851	0.065830014	isogroup08802	ENSG00000232312	ENST00000453746	GPANK1
isotig23712	7.887	21.574	4.848	7.39	1851	0.843916736	isogroup08803	ENSG00000165030	ENST00000375724	NFIL3
isotig23713	4.251	4.257	2.324	2.733	1851	0.014428496	isogroup08804	ENSG00000168813	ENST00000544431	ZNF507
isotig23714	9.041	4.118	11.654	6.187	1849	0.822987901	isogroup08805	ENSG00000119888	ENST00000263735	EPCAM
isotig23715	21.342	24.436	29.534	29.272	1850	0.208424138	isogroup08806	ENSG00000136448	ENST00000258960	NMT1
isotig23716	3.854	2.418	2.895	1.851	1850	0.532802285	isogroup08807	ENSG00000099331	ENST00000397274	MYO9B
isotig23717	13.929	15.457	14.503	13.267	1841	0.052453596	isogroup08808	ENSG00000151247	ENST00000504432	EIF4E
isotig23718	7.842	5.641	7.31	6.211	1849	0.379386789	isogroup08809	ENSG00000138674	ENST00000511338	SEC31A
isotig23719	4.703	3.72	3.769	3.782	1847	0.249389419	isogroup08810	ENSG00000065665	ENST00000379033	SEC61A2
isotig23720	1.426	3.448	4.294	4.129	1849	0.245246863	isogroup08811	ENSG00000130948	ENST00000375263	HSD17B3
isotig23721	10.175	6.938	7.839	7.567	1849	0.411221538	isogroup08812	ENSG00000099377	ENST00000297679	HSD3B7
isotig23723	13.022	4.048	8.123	4.506	1848	0.906346284	isogroup08814	ENSG00000197380	ENST00000391916	DACT3
isotig23724	1.918	1.684	1.743	1.397	1843	0.216690464	isogroup08815	ENSG00000165832	ENST00000298746	TRUB1
isotig23725	1.125	1.856	2.321	2.644	1846	0.199603592	isogroup08816	ENSG00000167978	ENST00000544933	SRRM2
isotig23726	10.819	8.074	6.441	7.737	1846	0.149103855	isogroup08817	ENSG00000074695	ENST00000251047	LMAN1
isotig23727	4.243	2.662	4.143	3.361	1845	0.395430976	isogroup08818	ENSG00000087152	ENST00000389384	ATXN7L3
isotig23729	10.186	6.386	10.418	8.469	1845	0.507261216	isogroup08820	ENSG00000167548	ENST00000526209	MLL2
isotig23731	3.412	3.489	3.214	2.003	1844	0.275249868	isogroup08822	ENSG00000183018	ENST00000329078	SPNS2
isotig23732	2.754	3.865	2.195	1.665	1842	0.193300519	isogroup08823	ENSG00000117114	ENST00000449420	LPHN2
isotig23733	3.164	1.642	5.04	4.519	1843	0.466587135	isogroup08824	ENSG00000142686	ENST00000270815	C1orf216
isotig23734	3.957	1.901	4.15	3.804	1842	0.476873074	isogroup08825	ENSG00000181513	ENST00000431281	ACBD4
isotig23735	8.453	7.436	8.629	8.002	1838	0.254302247	isogroup08826	ENSG00000184194	ENST00000332582	GPR173
isotig23736	10.359	14.263	42.39	9.523	1843	0.759496881	isogroup08827	ENSG00000125868	ENST00000246069	DSTN
isotig23737	16.086	14.481	19.481	19.312	1835	0.053815661	isogroup08828	ENSG00000141551	ENST00000392334	CSNK1D
isotig23739	4.727	7.709	8.485	14.086	1842	0.738276847	isogroup08830	ENSG00000182871	ENST00000539645	COL18A1
isotig23740	17.085	13.025	16.884	14.038	1834	0.450176599	isogroup08831	ENSG00000105618	ENST00000321030	PRPF31
isotig23741	4.068	3.515	2.989	3.539	1841	0.064082814	isogroup08832	ENSG00000197818	ENST00000361573	SLC9A8
isotig23743	6.563	5.428	5.647	6.007	1841	0.097862028	isogroup08834	ENSG00000167461	ENST00000300935	RAB8A
isotig23744	4.295	2.637	3.089	3.454	1841	0.384947772	isogroup08835	ENSG00000213079	ENST00000367186	SCAF8
isotig23745	2.159	6.307	3.33	1.982	1841	0.675114601	isogroup08836	ENSG00000103196	ENST00000262424	CRISPLD2
isotig23746	7.402	5.865	7.496	6.241	1840	0.407482904	isogroup08837	ENSG00000105671	ENST00000247003	DDX49
isotig23747	6.686	5.803	7.677	5.682	1840	0.423705568	isogroup08838	ENSG00000132635	ENST00000360652	FAM113A
isotig23748	38.835	71.285	36.578	34.31	1840	0.707419028	isogroup08839	ENSG00000105376	ENST00000221980	ICAM5
isotig23749	2.484	5.037	6.195	6.546	1833	0.329197039	isogroup08840	ENSG00000165240	ENST00000341514	ATP7A
isotig23755	2.423	3.49	4.591	3.629	1838	0.108561284	isogroup08846	ENSG00000100997	ENST00000339157	ABHD12
isotig23757	2.219	2.582	2.675	1.756	1836	0.140236342	isogroup08848	ENSG00000119684	ENST00000556740	MLH3
isotig23760	11.802	9.797	9.011	8.694	1836	0.194164725	isogroup08851	ENSG00000111726	ENST00000229329	CMAS
isotig23761	1.924	2.974	4.694	4.972	1837	0.353244533	isogroup08852	ENSG00000182568	ENST00000454909	SATB1
isotig23762	2.926	4.562	6.285	5.475	1836	0.093738258	isogroup08853	ENSG00000065978	ENST00000436427	YBX1
isotig23764	2.05	1.896	1.129	1.491	1832	0.022976629	isogroup08855	ENSG00000175105	ENST00000309495	ZNF654
isotig23766	52.552	75.528	63.357	53.123	1829	0.27546592	isogroup08857	ENSG00000242110	ENST00000335606	AMACR
isotig23769	8.298	5.096	5.417	3.796	1834	0.545089051	isogroup08860	ENSG00000173269	ENST00000443699	MMRN2
isotig23770	8.684	6.75	9.439	12.14	1833	0.107715864	isogroup08861	ENSG00000164897	ENST00000482202	TMUB1
isotig23771	29.068	26.729	38.189	39.155	1833	0.103413617	isogroup08862	ENSG00000104969	ENST00000221566	SGTA
isotig23772	3.912	3.78	3.224	1.795	1833	0.190520027	isogroup08863	ENSG00000010327	ENST00000321725	STAB1

isotig23773	6.155	5.492	5.792	2.067	1830	0.501456001	isogroup08864	ENSG00000176971	ENST00000318627	FIBIN	
isotig23774	3.37	5.229	3.328	3.277	1831	0.417449463	isogroup08865	ENSG00000186314	ENST00000505416	PRELID2	
isotig23775	3.199	2.664	2.695	2.558	1829	0.203191929	isogroup08866	ENSG00000110060	ENST00000530811	PUS3	
isotig23777	8.433	9.086	8.049	8.793	1833	0.176824228	isogroup08868	ENSG00000151093	ENST00000280701	OXSM	
isotig23778	3.379	2.784	3.558	2.603	1832	0.313284362	isogroup08869	ENSG00000166526	ENST00000424697	ZNF3	
isotig23779	11.725	11.289	14.381	13.767	1831	0.052660254	isogroup08870	ENSG00000256269	ENST00000278715	HMBS	
isotig23780	1.189	1.518	1.613	1.345	1831	0.129903434	isogroup08871	ENSG00000146842	ENST00000462753	TMEM209	
isotig23781	4.884	4.623	3.961	3.043	1832	0.302350267	isogroup08872	ENSG00000213339	ENST00000250237	QTRT1	
isotig23782	4.319	3.098	4.569	3.375	1831	0.443582325	isogroup08873	ENSG00000171206	ENST00000302424	TRIM8	
isotig23783	5.271	4.233	2.442	2.802	1830	0.251437213	isogroup08874	ENSG00000116521	ENST00000302631	SCAMP3	
isotig23784	1.659	2.434	2.836	2.948	1830	0.150381378	isogroup08875	ENSG00000086015	ENST00000372009	MAST2	
isotig23785	2.577	2.518	1.888	2.093	1829	0.088308785	isogroup08876	ENSG00000108854	ENST00000262435	SMURF2	
isotig23786	2.839	3.465	3.352	3.109	1829	0.017143233	isogroup08877	ENSG00000151779	ENST00000442506	NBAS	
isotig23788	2.603	2.522	3.074	2.182	1826	0.142387465	isogroup08879	ENSG00000165675	ENST00000394363	ENOX2	
isotig23789	1.605	2.758	3.352	3.513	1829	0.222739911	isogroup08880	ENSG00000110871	ENST00000288532	COQ5	
isotig23790	52.91	28.864	28.162	34.609	1828	0.631566093	isogroup08881	ENSG00000189060	ENST00000340857	H1F0	
isotig23791	107.244	75.091	94.1	79.898	1819	0.5635192	isogroup08882	ENSG00000126247	ENST00000246533	CAPNS1	
isotig23792	9.898	10.528	12.145	12.8	1826	0.111097543	isogroup08883	ENSG00000186141	ENST00000334163	POLR3C	
isotig23793	1.683	2.478	2.919	3.208	1828	0.18122041	isogroup08884	ENSG00000124574	ENST00000443394	ABCC10	
isotig23794	3.279	3.938	4.356	3.345	1827	0.194174119	isogroup08885	ENSG00000110104	ENST00000227520	CCDC86	
isotig23795	4.267	6.907	6.7	6.484	1830	0.370237469	isogroup08886	ENSG00000128833	ENST00000261839	MYO5C	
isotig23796	3.387	1.52	2.545	2.339	1828	0.526208011	isogroup08887	ENSG00000142197	ENST00000399151	DOPEY2	
isotig23798	3.367	3.73	3.264	3.296	1827	0.172428045	isogroup08889	ENSG00000171456	ENST00000421155	ASXL1	
isotig23799	9.187	5.64	8.423	6.314	1826	0.634882017	isogroup08890	ENSG00000061936	ENST00000541286	SFSWAP	
isotig23802	2.559	4.837	5.734	5.394	1826	0.271774254	isogroup08893	ENSG00000165283	ENST00000356493	STOML2	
isotig23803	6.653	3.025	7.214	3.717	1825	0.767490794	isogroup08894	ENSG00000061938	ENST00000416152	TNK2	
isotig23805	1.379	2.586	3.318	2.858	1825	0.109575787	isogroup08896	ENSG00000184985	ENST00000507866	SORCS2	
isotig23806	12.981	9.496	16.669	8.93	1820	0.687016232	isogroup08897	ENSG00000162368	ENST00000371873	CMPK1	
isotig23808	10.046	7.726	9.916	7.872	1823	0.437429548	isogroup08899	ENSG00000174109	ENST00000442039	C16orf91	
isotig23809	1.248	2.463	2.966	2.995	1824	0.157041407	isogroup08900	ENSG00000142173	ENST00000409416	COL6A2	
isotig23810	63.603	112.442	64.212	79.851	1824	0.641964755	isogroup08901	ENSG00000136383	ENST00000258888	ALPK3	
isotig23811	2.146	4.31	5.061	4.553	1824	0.259468701	isogroup08902	ENSG00000184956	ENST00000421673	MUC6	
isotig23812	4.251	4.072	3.312	2.726	1823	0.228122417	isogroup08903	ENSG00000069020	ENST00000443808	MAST4	
isotig23814	1.195	2.256	2.838	2.789	1822	0.125901781	isogroup08905	ENSG00000162572	ENST00000379101	SCNN1D	
isotig23816	3.371	4.594	4.304	2.548	1819	0.065830014	isogroup08907	ENSG00000083857	ENST00000512772	FAT1	
isotig23817	2.226	6.146	2.393	6.835	1821	0.840488089	isogroup08908	ENSG00000185950	ENST00000375856	IRS2	
isotig23818	7.544	9.622	12.204	7.169	1808	0.323795747	isogroup08909	ENSG00000121152	ENST00000455200	NCAPH	
isotig23820	5.451	5.027	4.39	5.188	1821	0.006660029	isogroup08911	ENSG00000110066	ENST00000304363	SUV420H1	
isotig23821	9.387	9.229	11.562	10.424	1819	0.109190652	isogroup08912	ENSG00000184470	ENST00000542719	TXNRD2	
isotig23822	2.591	1.611	2.456	1.957	1819	0.417440069	isogroup08913	ENSG00000167972	ENST00000382381	ABCA3	
isotig23824	1.198	2.514	3.364	1.663	1817	0.06751146	isogroup08915	ENSG00000164128	ENST00000296533	NPY1R	
isotig23826	7.766	4.02	5.942	4.248	1818	0.698983618	isogroup08917	ENSG00000132478	ENST00000293218	UNK	
isotig23827	9.986	5.135	8.719	6.686	1818	0.709739235	isogroup08918	ENSG00000138785	ENST00000451321	INTS12	
isotig23828	3.911	3.811	1.686	2.869	1818	0.181304952	isogroup08919	ENSG00000138688	ENST00000388738	KIAA1109	
isotig23832	7.078	6.307	6.595	5.286	1817	0.316064853	isogroup08923	ENSG00000197976	ENST00000313871	AKAP17A	
isotig23834	15.522	21.265	17.25	15.333	1809	0.331921169	isogroup08925	ENSG00000117791	ENST00000366913		2-Mar
isotig23835	7.896	6.792	9.751	7.532	1816	0.189176749	isogroup08926	ENSG00000124212	ENST00000244043	PTGIS	
isotig23836	2.743	1.816	4.389	2.741	1813	0.581808447	isogroup08927	ENSG00000104973	ENST00000536547	MED25	
isotig23837	21.523	17.314	19.391	17.339	1814	0.372041031	isogroup08928	ENSG00000103502	ENST00000569956	CDIPT	
isotig23839	14.805	18.131	23.211	26.906	1813	0.387192831	isogroup08930	ENSG00000158805	ENST00000568064	ZNF276	
isotig23840	4.794	4.517	5.535	5.027	1808	0.042524611	isogroup08931	ENSG00000180448	ENST00000536472	HMHA1	
isotig23843	3.52	4.28	1.296	1.793	1810	0.379245886	isogroup08934	ENSG00000102189	ENST00000322349	EEA1	
isotig23844	7.627	7.101	8.649	6.577	1804	0.372181934	isogroup08935	ENSG00000159593	ENST00000290810	NAE1	
isotig23846	6.69	6.608	11.065	10.729	1809	0.018711956	isogroup08937	ENSG00000140995	ENST00000268676	DEF8	
isotig23847	1.821	3.325	4.746	4.121	1809	0.082137221	isogroup08938	ENSG00000126759	ENST00000396992	CFP	
isotig23849	5.644	5.538	4.085	3.603	1808	0.144688886	isogroup08940	ENSG00000181788	ENST00000312960	SIAH2	
isotig23850	5.641	3.862	3.022	2.624	1809	0.358598858	isogroup08941	ENSG00000169188	ENST00000374987	APEX2	
isotig23851	19.595	12.936	17.533	16.269	1804	0.441553318	isogroup08942	ENSG00000124209	ENST00000244040	RAB22A	
isotig23853	37.612	36.524	53.979	45.806	1800	0.243236642	isogroup08944	ENSG00000206418	ENST00000329286	RAB12	
isotig23855	3.437	3.455	4.097	2.379	1807	0.374342451	isogroup08946	ENSG00000166173	ENST00000299213	LARP6	
isotig23856	4.742	5.562	4.251	4.262	1807	0.193140828	isogroup08947	ENSG00000160972	ENST00000435887	PPP1R16A	
isotig24088	3.354	2.014	2.963	2.06	1739	0.505269783	isogroup09179	ENSG00000106733	ENST00000361092	C9orf95	
isotig24089	1.584	4.868	3.48	3.86	1740	0.635971669	isogroup09180	ENSG00000100299	ENST00000547805	ARSA	
isotig24090	3.928	2.66	2.792	2.768	1739	0.157802285	isogroup09181	ENSG00000183495	ENST00000542457	EP400	
isotig24091	23.61	18.55	25.225	27.294	1729	0.147281506	isogroup09182	ENSG00000117899	ENST00000561312	MESDC2	
isotig24093	4.763	4.243	7.116	7.467	1736	0.047550162	isogroup09184	ENSG00000130669	ENST00000542377	PAK4	
isotig24094	38.859	29.501	34.511	26.34	1733	0.500272413	isogroup09185	ENSG00000120805	ENST00000261636	ARL1	
isotig24587	3.972	1.722	4.759	4.203	1614	0.374821523	isogroup09678	ENSG00000149557	ENST00000278919	FEZ1	
isotig24588	1.226	2.454	2.862	2.942	1613	0.193197189	isogroup09679	ENSG00000112031	ENST00000367231	MTRF1L	
isotig24589	6.934	5.098	3.434	1.888	1613	0.44692643	isogroup09680	ENSG00000174611	ENST00000423778	KY	
isotig24590	3.219	7.576	13.622	11.006	1613	0.048282859	isogroup09681	ENSG00000008056	ENST00000340666	SYN1	
isotig24592	7.515	10.373	16.542	8.48	1613	0.197367927	isogroup09683	ENSG00000162576	ENST00000477278	MXRA8	
isotig24593	6.058	7.061	6.621	5.694	1612	0.034305253	isogroup09684	ENSG00000068745	ENST00000328631	IP6K2	
isotig24594	2.67	10.511	4.306	5.277	1608	0.839022695	isogroup09685	ENSG00000103534	ENST00000564959	TMC5	
isotig24597	3.174	8.696	5.467	8.074	1611	0.796808071	isogroup09688	ENSG00000118162	ENST00000338134	KPTN	
isotig24598	2.568	3.18	3.624	2.976	1610	0.102370933	isogroup09689	ENSG00000139187	ENST00000356986	KLRG1	
isotig24599	12.291	7.77	10.077	12.119	1607	0.152917637	isogroup09690	ENSG00000065802	ENST00000264607	ASB1	
isotig24600	2.715	2.068	2.831	2.404	1611	0.275813482	isogroup09691	ENSG00000183826	ENST00000481247	BTBD9	
isotig24601	5.775	4.451	5.65	4.288	1608	0.440444879	isogroup09692	ENSG00000153485	ENST00000415050	C14orf109	
isotig24602	4.881	3.48	4.743	3.244	1608	0.527804915	isogroup09693	ENSG00000187531	ENST00000328666	SIRT7	
isotig24603	11.451	10.405	25.159	6.391	1608	0.924569775	isogroup09694	ENSG00000100292	ENST00000216117	HMOX1	
isotig24607	3.833	2.991	2.81	2.197	1610	0.317426918	isogroup09698	ENSG00000105186	ENST00000306065	ANKRD27	

isotig24608	2.237	1.196	1.747	1.922	1607	0.189533704	isogroup09699	ENSG00000183150	ENST00000540510	GPR19
isotig24610	6.632	6.558	7.716	7.03	1608	0.183456076	isogroup09701	ENSG00000100056	ENST00000252137	DGCR14
isotig24612	10.414	5.614	9.882	7.551	1607	0.657783497	isogroup09703	ENSG00000011009	ENST00000374514	LYPLA2
isotig24614	3.79	2.736	3.034	3.232	1607	0.210002254	isogroup09705	ENSG00000117036	ENST00000368192	ETV3
isotig24616	9.403	10.45	9.141	5.958	1612	0.148690539	isogroup09707	ENSG00000105538	ENST00000222145	RASIP1
isotig24617	1.816	1.363	1.494	1.366	1606	0.091953483	isogroup09708	ENSG00000176393	ENST00000295640	RNPEP
isotig24618	3.66	2.385	1.589	1.451	1604	0.320695874	isogroup09709	ENSG00000121481	ENST00000367510	RNF2
isotig24621	16.088	16.805	18.442	13.237	1605	0.232293154	isogroup09712	ENSG00000123933	ENST00000337190	MXD4
isotig24622	4.656	2.362	1.704	1.34	1604	0.486473285	isogroup09713	ENSG00000108821	ENST00000225964	COL1A1
isotig24623	3.013	3.786	3.177	2.484	1604	0.027992786	isogroup09714	ENSG00000103544	ENST00000438132	C16orf62
isotig24624	2.549	1.239	1.86	1.233	1598	0.457353273	isogroup09715	ENSG00000253710	ENST00000521508	ALG11
isotig24625	5.124	4.86	5.129	4.779	1602	0.108533103	isogroup09716	ENSG00000132434	ENST00000254770	LANCL2
isotig24627	5.98	5.593	3.939	3.785	1603	0.191938453	isogroup09718	ENSG00000162729	ENST00000368086	IGSF8
isotig24628	11.558	9.803	11.425	9.374	1600	0.400681972	isogroup09719	ENSG00000144048	ENST00000272444	DUSP11
isotig24630	6.079	8.185	4.097	2.672	1600	0.19734914	isogroup09721	ENSG00000182636	ENST00000331837	NDN
isotig24631	4.749	4.479	3.224	2.928	1598	0.110299091	isogroup09722	ENSG00000196141	ENST00000451764	SPATS2L
isotig24632	15.555	10.403	19.503	11.662	1601	0.701454122	isogroup09723	ENSG00000143819	ENST00000366837	EPHX1
isotig24633	1.522	2.118	2.666	2.644	1600	0.129649808	isogroup09724	ENSG00000115935	ENST00000392548	WIPF1
isotig24634	4.234	6.773	4.123	3.99	1599	0.387869167	isogroup09725	ENSG00000162341	ENST00000294309	TPCN2
isotig24635	2.771	4.937	6.624	6.632	1600	0.206301195	isogroup09726	ENSG00000104419	ENST00000517599	NDRG1
isotig24637	5.529	5.889	6.197	5.149	1600	0.160714286	isogroup09728	ENSG00000101464	ENST00000217446	PIGU
isotig24638	3.46	2.726	1.282	1.946	1599	0.021652138	isogroup09729	ENSG00000166128	ENST00000321437	RAB8B
isotig24639	17.847	3.25	3.684	1.512	1599	0.93135192	isogroup09730	ENSG00000151067	ENST00000399649	CACNA1C
isotig24640	1.751	1.847	2.058	1.598	1597	0.021652138	isogroup09731	ENSG00000120458	ENST00000526629	C11orf61
isotig24641	3.099	4.264	3.351	3.88	1598	0.436903509	isogroup09732	ENSG00000051596	ENST00000265097	THOC3
isotig24642	21.792	13.324	21.159	12.939	1590	0.737450214	isogroup09733	ENSG00000155438	ENST00000285814	MKI67IP
isotig24643	7.973	8.529	11.255	6.725	1598	0.382251822	isogroup09734	ENSG00000115935	ENST00000392546	WIPF1
isotig24644	12.985	8.85	12.063	12.944	1598	0.293323063	isogroup09735	ENSG00000178951	ENST00000322357	ZBTB7A
isotig24645	4.287	2.347	2.542	3.222	1598	0.268214098	isogroup09736	ENSG00000054965	ENST00000064778	FAM168A
isotig24646	6.832	4.21	4.813	4.18	1596	0.466380476	isogroup09737	ENSG00000122254	ENST00000261374	HS3ST2
isotig24647	3.299	1.977	3.421	2.69	1598	0.42849628	isogroup09738	ENSG00000115935	ENST00000409891	WIPF1
isotig24649	2.062	1.477	2.277	1.107	1598	0.431257985	isogroup09740	ENSG00000164604	ENST00000501255	GPR85
isotig24650	67.853	102.001	96.765	84.844	1597	0.215647779	isogroup09741	ENSG00000127884	ENST00000368547	ECHS1
isotig24651	11.9	8.36	12.645	9.174	1596	0.543078831	isogroup09742	ENSG00000008128	ENST00000404249	CDK11A
isotig25087	3.318	2.755	2.538	2.27	1499	0.173724356	isogroup10178	ENSG00000185085	ENST00000330574	INTS5
isotig25088	14.229	12.813	20.756	17.145	1499	0.330033065	isogroup10179	ENSG00000144115	ENST00000358591	THNSL2
isotig25091	1.785	2.883	2.242	2.315	1499	0.303975351	isogroup10182	ENSG00000187961	ENST00000338591	KLHL17
isotig25094	5.63	3.942	2.454	2.468	1497	0.293567295	isogroup10185	ENSG00000151553	ENST00000369248	FAM160B1
isotig25095	6.563	29.987	2.737	4.264	1491	0.963440295	isogroup10186	ENSG00000132386	ENST00000254722	SERPINF1
isotig25096	4.439	6.747	5.029	5.44	1498	0.419816638	isogroup10187	ENSG00000187713	ENST00000537254	TMEM203
isotig25097	2.949	2.442	3.417	2.318	1497	0.416547682	isogroup10188	ENSG00000172932	ENST00000511455	ANKRD13D
isotig25099	4.882	3.789	5.353	3.335	1497	0.461843391	isogroup10190	ENSG00000129473	ENST00000250405	BCL2L2
isotig25101	1.615	2.723	3.345	2.918	1496	0.112901105	isogroup10192	ENSG00000168066	ENST00000486867	SF1
isotig25103	1.897	3.667	3.965	3.324	1495	0.176260615	isogroup10194	ENSG00000213923	ENST00000431611	CSNK1E
isotig25104	1.229	1.642	2.828	2.755	1495	0.203548884	isogroup10195	ENSG00000100033	ENST00000420436	PRODH
isotig25105	4.776	2.614	4.959	2.129	1493	0.708020215	isogroup10196	ENSG00000227124	ENST00000478296	ZNF717
isotig25107	6.674	5.342	6.703	5.128	1493	0.471697227	isogroup10198	ENSG00000145919	ENST00000311086	BOD1
isotig25108	1.736	2.763	3.397	3.429	1495	0.13943789	isogroup10199	ENSG00000176542	ENST00000478658	KIAA2018
isotig25109	27.283	19.595	29.415	28.605	1494	0.452233787	isogroup10200	ENSG00000149541	ENST00000265471	B3GAT3
isotig25111	5.016	4.067	3.933	4.468	1493	0.152588863	isogroup10202	ENSG00000144647	ENST00000441964	C3orf39
isotig25112	3.317	3.927	3.117	3.419	1493	0.383379049	isogroup10203	ENSG00000065308	ENST00000182527	TRAM2
isotig25114	3.466	11.214	4.962	6.512	1492	0.880795822	isogroup10205	ENSG00000086061	ENST00000330899	DNAJA1
isotig25116	1.553	1.939	2.092	2.34	1492	0.241207635	isogroup10207	ENSG00000141564	ENST00000544334	RPTOR
isotig25117	8.821	9.351	15.643	10.113	1492	0.448072443	isogroup10208	ENSG00000049246	ENST00000377532	PER3
isotig25118	2.677	3.636	2.017	2.281	1492	0.329741865	isogroup10209	ENSG00000121542	ENST00000492595	SEC22A
isotig25119	1.197	1.705	1.069	1.296	1485	0.167515218	isogroup10210	ENSG00000157741	ENST00000473989	UBN2
isotig25120	3.058	2.377	3.088	2.3	1489	0.188754039	isogroup10211	ENSG00000134851	ENST00000542052	TMEM165
isotig25123	2.038	1.393	1.029	1.307	1490	0.022976629	isogroup10214	ENSG00000133030	ENST00000313485	MPRIP
isotig25128	8.584	8.13	3.212	1.864	1489	0.306624333	isogroup10219	ENSG00000133026	ENST00000396239	MYH10
isotig25129	2.911	5.804	4.018	5.232	1489	0.643289246	isogroup10220	ENSG00000138674	ENST00000505472	SEC31A
isotig25130	3.286	2.148	2.815	1.729	1489	0.419760277	isogroup10221	ENSG00000105497	ENST00000262259	ZNF175
isotig25132	8.845	5.935	7.582	5.416	1488	0.596950853	isogroup10223	ENSG00000182093	ENST00000333781	WRB
isotig25133	3.075	2.33	2.98	2.032	1487	0.340037198	isogroup10224	ENSG00000196605	ENST00000397902	ZNF846
isotig25134	20.32	17.205	20.996	19.905	1488	0.292740663	isogroup10225	ENSG00000104946	ENST00000221543	TBC1D17
isotig25137	3.317	1.754	2.384	2.605	1487	0.354343579	isogroup10228	ENSG00000139626	ENST00000422257	ITGB7
isotig25139	4.405	1.651	3.396	2.379	1487	0.702468626	isogroup10230	ENSG00000139718	ENST00000542440	SETD1B
isotig25141	4.328	4.496	2.56	2.124	1486	0.177904486	isogroup10232	ENSG00000188760	ENST00000373883	TMEM198
isotig25142	2.701	1.972	2.87	2.049	1486	0.460997971	isogroup10233	ENSG00000099814	ENST00000556508	KIAA0284
isotig25144	6.621	5.464	3.658	3.334	1484	0.317520854	isogroup10235	ENSG00000198040	ENST00000539354	ZNF84
isotig25145	3.043	5.481	2.54	1.872	1483	0.416237694	isogroup10236	ENSG00000150540	ENST00000410115	HNMT
isotig25146	2.059	2.142	1.555	2.052	1484	0.111369956	isogroup10237	ENSG00000151779	ENST00000441750	NBAS
isotig25148	1.156	2.243	2.512	2.216	1485	0.231588638	isogroup10239	ENSG00000198090	ENST00000345847	KRTAP4-6
isotig25149	8.527	8.741	7.859	7.673	1485	0.078163748	isogroup10240	ENSG00000181873	ENST00000366711	IBA57
isotig25150	1.694	3.281	3.593	3.89	1485	0.315416698	isogroup10241	ENSG00000136381	ENST00000258886	IREB2
isotig25151	33.082	29.261	35.378	30.759	1476	0.285780041	isogroup10242	ENSG00000113312	ENST00000522793	TTC1
isotig25152	4.315	3.187	3.167	3.219	1481	0.214323288	isogroup10243	ENSG00000205785	ENST00000431698	AC091435.2
isotig25153	6.191	5.99	6.657	5.265	1484	0.311076877	isogroup10244	ENSG00000233149	ENST00000456968	GTF2H4
isotig25154	5.437	7.022	27.61	6.487	1484	0.747388592	isogroup10245	ENSG00000133392	ENST00000452625	MYH11
isotig25157	4.498	3.684	5.067	3.653	1483	0.496514992	isogroup10248	ENSG00000184281	ENST00000451491	TSSC4
isotig25159	79.223	64.963	67.667	78.58	1482	0.008181784	isogroup10250	ENSG00000108878	ENST00000226021	CACNG1
isotig25160	2.291	4.072	5.071	5.196	1482	0.204507026	isogroup10251	ENSG00000135297	ENST00000415954	MTO1
isotig25161	4.655	1.775	2.82	1.664	1480	0.69851394	isogroup10252	ENSG00000179588	ENST00000319555	ZFPM1

isotig25162	4.353	3.134	3.053	3.248	1482	0.161428196	isogroup10253	ENSG00000070444	ENST00000404961	MNT
isotig25163	1.685	3.239	1.82	1.799	1481	0.516805065	isogroup10254	ENSG00000229328	ENST00000446776	OR2W1
isotig25165	19.392	8.766	17.195	13.103	1483	0.736238446	isogroup10256	ENSG00000126461	ENST00000360565	SCAF1
isotig25166	7.053	7.095	2.855	2.826	1481	0.198326069	isogroup10257	ENSG00000091409	ENST00000409532	ITGA6
isotig25167	21.393	13.111	12.168	9.653	1475	0.66305328	isogroup10258	ENSG00000100528	ENST00000216416	CNIH
isotig25168	2.05	2.236	1.633	2.041	1481	0.128719847	isogroup10259	ENSG00000010072	ENST00000295050	C1orf124
isotig25171	2.051	3.408	4.628	4.084	1480	0.082137221	isogroup10262	ENSG00000149743	ENST00000394546	TRPT1
isotig25174	1.7	3.459	4.205	3.933	1480	0.251906891	isogroup10265	ENSG00000167978	ENST00000544933	SRRM2
isotig25175	3.121	1.945	2.274	1.378	1478	0.469715187	isogroup10266	ENSG00000182963	ENST00000426548	GJC1
isotig25176	10.019	19.112	2.097	7.462	1479	0.852662133	isogroup10267	ENSG00000101752	ENST00000261537	MIB1
isotig25177	7.986	7.766	12.131	7.089	1478	0.407116555	isogroup10268	ENSG00000100139	ENST00000402631	MICALL1
isotig25178	2.552	2.355	2.522	2.861	1479	0.074481476	isogroup10269	ENSG00000132849	ENST00000371158	INADL
isotig25179	3.04	2.447	2.236	2.166	1478	0.084401067	isogroup10270	ENSG00000127989	ENST00000419292	MTERF
isotig25183	6.067	4.005	5.21	4.165	1478	0.421817464	isogroup10274	ENSG00000230230	ENST00000455000	TRIM26
isotig25185	37.214	24.053	45.556	30.757	1479	0.668623657	isogroup10276	ENSG00000090273	ENST00000321265	NUDC
isotig25186	9.275	10.156	10.102	12.506	1475	0.379161344	isogroup10277	ENSG00000135845	ENST00000367728	PIGC
isotig25188	6.382	3.622	6.801	3.198	1477	0.743772075	isogroup10279	ENSG00000184956	ENST00000421673	MUC6
isotig25191	2.222	1.072	2.029	1.322	1474	0.470419704	isogroup10282	ENSG00000186714	ENST00000335185	CCDC73
isotig25193	3.301	2.218	3.803	2.563	1476	0.49143308	isogroup10284	ENSG00000125485	ENST00000438527	DDX31
isotig25195	7.455	6.833	10.012	10.965	1468	0.048837078	isogroup10286	ENSG00000100813	ENST00000555053	ACIN1
isotig25197	2.665	4.578	2.539	2.078	1476	0.459560757	isogroup10288	ENSG00000080573	ENST00000264828	COL5A3
isotig25198	6.968	3.701	7.118	5.4	1476	0.575749605	isogroup10289	ENSG00000076944	ENST00000320400	STXBP2
isotig25200	45.723	25.964	29.782	19.351	1475	0.784117382	isogroup10291	ENSG00000173171	ENST00000368376	MTX1
isotig25203	8.313	7.162	9.464	6.936	1475	0.387850379	isogroup10294	ENSG00000136819	ENST00000372447	C9orf78
isotig25204	1.966	3.566	1.893	2.021	1473	0.587200346	isogroup10295	ENSG00000143344	ENST00000536277	RGL1
isotig25205	1.706	2.555	1.677	2.353	1475	0.386215901	isogroup10296	ENSG00000118508	ENST00000367495	RAB32
isotig25206	11.062	6.142	8.838	5.479	1475	0.7099365	isogroup10297	ENSG00000064607	ENST00000452918	SUGP2
isotig25209	6.237	5.285	4.162	2.599	1473	0.469564891	isogroup10300	ENSG00000165475	ENST00000298248	CRYL1
isotig25210	2.195	4.068	4.923	4.313	1474	0.167120688	isogroup10301	ENSG00000204262	ENST00000452536	COL5A2
isotig25211	2.223	3.257	4.262	4.498	1473	0.106607425	isogroup10302	ENSG00000198324	ENST00000450786	FAM109A
isotig25213	5.749	4.85	3.777	3.906	1473	0.17548095	isogroup10304	ENSG00000181090	ENST00000460843	EHMT1
isotig25214	1.695	2.119	2.47	2.462	1473	0.17035207	isogroup10305	ENSG00000149260	ENST00000529629	CAPN5
isotig25215	3.267	3.114	4.889	4.149	1473	0.089520553	isogroup10306	ENSG00000213533	ENST00000355083	TMEM110
isotig25216	1.628	4.241	2.609	1.779	1472	0.354343579	isogroup10307	ENSG00000213121	ENST00000392385	AL590867.1
isotig25217	3.446	1.254	2.17	2.175	1472	0.52575712	isogroup10308	ENSG00000142197	ENST00000399151	DOPEY2
isotig25221	1.302	2.483	2.66	2.464	1472	0.166209514	isogroup10312	ENSG00000187758	ENST00000209668	ADH1A
isotig25224	2.037	4.309	5.523	6.712	1471	0.633125423	isogroup10315	ENSG00000139445	ENST00000299162	FOXN4
isotig25227	6.272	5.031	7.094	6.161	1468	0.337679417	isogroup10318	ENSG00000173409	ENST00000310256	ARV1
isotig25229	7.472	5.736	6.771	5.954	1470	0.395224318	isogroup10320	ENSG00000105063	ENST00000412770	PPP6R1
isotig25230	1.359	2.033	2.726	3.046	1470	0.183258811	isogroup10321	ENSG00000072518	ENST00000425897	MARK2
isotig25232	1.577	2.579	3.356	3.078	1469	0.039415345	isogroup10323	ENSG00000006788	ENST00000418404	MYH13
isotig25233	1.074	2.229	3.042	2.622	1469	0.091699857	isogroup10324	ENSG00000181143	ENST00000397910	MUC16
isotig25234	7.368	8.513	6.611	8.854	1469	0.326998948	isogroup10325	ENSG00000137393	ENST00000259939	RNF144B
isotig25235	21.915	14.097	22.361	17.769	1469	0.520355828	isogroup10326	ENSG00000133627	ENST00000256001	ACTR3B
isotig25236	7.613	2.384	3.859	4.023	1469	0.736510859	isogroup10327	ENSG00000054967	ENST00000393580	RELT
isotig25238	3.472	2.608	1.586	1.587	1467	0.237074472	isogroup10329	ENSG00000117543	ENST00000488176	DPH5
isotig25239	1.319	2.697	3.22	2.455	1468	0.003503795	isogroup10330	ENSG00000149503	ENST00000394818	INCENP
isotig25240	1.7	2.542	1.031	1.129	1468	0.256303074	isogroup10331	ENSG00000132964	ENST00000381527	CDK8
isotig25245	1.96	2.725	3.172	3.403	1467	0.310738709	isogroup10336	ENSG00000067704	ENST00000366922	IARS2
isotig25246	15.919	11.962	19.221	14.404	1464	0.592639212	isogroup10337	ENSG00000131943	ENST00000392278	C19orf12
isotig25247	4.414	2.519	3.791	2.382	1466	0.458189299	isogroup10338	ENSG00000128656	ENST00000295497	CHN1
isotig25248	3.235	2.42	3.079	2.28	1463	0.210781919	isogroup10339	ENSG00000243279	ENST00000376390	PRAF2
isotig25249	3.228	1.436	4.135	3.107	1462	0.564721575	isogroup10340	ENSG00000166348	ENST00000408019	USP54
isotig25250	7.95	6.189	10.875	7.554	1467	0.581442098	isogroup10341	ENSG00000183779	ENST00000331569	ZNF703
isotig25252	1.43	2.519	3.275	3.39	1466	0.214163598	isogroup10343	ENSG00000170961	ENST00000443194	HAS2
isotig25253	3.106	3.505	5.038	5.711	1466	0.210575261	isogroup10344	ENSG00000167945	ENST00000301698	PRR25
isotig25254	9.501	13.57	10.716	10.36	1463	0.393185917	isogroup10345	ENSG00000145979	ENST00000379300	TBC1D7
isotig25255	1.145	2.183	2.609	2.72	1465	0.229437514	isogroup10346	ENSG00000226763	ENST00000526798	SRRM5
isotig25256	2.59	3.794	2.899	2.537	1465	0.298714962	isogroup10347	ENSG00000186635	ENST00000393605	ARAP1
isotig25258	17.722	15.773	11.949	9.704	1465	0.444841061	isogroup10349	ENSG00000177192	ENST00000376649	PUS1
isotig25259	2.726	2.04	1.866	1.926	1465	0.15977493	isogroup10350	ENSG00000141431	ENST00000269197	ASXL3
isotig25260	11.613	7.966	14.81	10.979	1460	0.491743068	isogroup10351	ENSG00000146834	ENST00000310512	MEPCE
isotig25263	4.504	4.593	4.783	2.739	1463	0.185475689	isogroup10354	ENSG00000167642	ENST00000301244	SPINT2
isotig25265	5.61	3.07	4.657	4.055	1463	0.532097768	isogroup10356	ENSG00000118600	ENST00000261234	TMEM5
isotig25266	136.271	151.15	134.478	144.499	1456	0.106757721	isogroup10357	ENSG00000169564	ENST00000303577	PCBP1
isotig25267	2.976	2.042	2.474	2.081	1463	0.334898925	isogroup10358	ENSG00000100207	ENST00000359486	TCF20
isotig25268	1.544	3.515	3.36	3.384	1462	0.365089802	isogroup10359	ENSG00000101444	ENST00000217426	AHCY
isotig25269	1.981	1.458	2.614	1.256	1462	0.381941835	isogroup10360	ENSG00000072952	ENST00000558540	MRVI1
isotig25270	38.875	37.782	35.097	20.18	1461	0.394942511	isogroup10361	ENSG00000167566	ENST00000335999	NCKAP5L
isotig25271	51.211	40.503	29.68	31.406	1462	0.301495454	isogroup10362	ENSG00000184887	ENST00000536364	BTBD6
isotig25272	3.062	2.997	3.235	3.141	1461	0.10439994	isogroup10363	ENSG00000164318	ENST00000354891	EGFLAM
isotig25273	5.14	3.27	6.559	3.938	1461	0.562570452	isogroup10364	ENSG00000175463	ENST00000526387	TBC1D10C
isotig25274	4.216	3.118	3.341	2.025	1459	0.472749305	isogroup10365	ENSG00000013503	ENST00000539066	POLR3B
isotig25275	2.051	3.14	1.947	2.176	1461	0.421573232	isogroup10366	ENSG00000137413	ENST00000465926	TAF8
isotig25276	6.762	4.123	4.72	2.716	1459	0.637249192	isogroup10367	ENSG00000143630	ENST00000368358	HCN3
isotig25277	2.505	1.996	2.139	1.826	1459	0.235449388	isogroup10368	ENSG00000095485	ENST00000472872	CWF19L1
isotig25278	5.143	4.094	5.438	5.536	1460	0.360073645	isogroup10369	ENSG00000218891	ENST00000325421	ZNF579
isotig25279	48.368	40.884	54.446	41.968	1460	0.610966033	isogroup10370	ENSG00000151929	ENST00000369085	BAG3
isotig25281	2.998	2.153	2.404	2.854	1459	0.174287969	isogroup10372	ENSG00000064932	ENST00000361757	SBNO2
isotig25282	16.26	19.86	18.604	16.455	1453	0.038522958	isogroup10373	ENSG00000136710	ENST00000259229	CCDC115
isotig25283	14.67	15.744	12.475	10.287	1458	0.068760803	isogroup10374	ENSG00000130429	ENST00000451682	ARPC1B
isotig25286	4.767	7.964	7.801	7.232	1458	0.356691967	isogroup10377	ENSG00000008382	ENST00000262966	MPND

isotig25287	4.383	3.512	4.167	4.099	1458	0.18262005	isogroup10378	ENSG00000176092	ENST00000475866	AIM1L
isotig25289	2.421	3.569	1.738	1.855	1328	0.400588036	isogroup10380	ENSG00000040933	ENST00000409540	INPP4A
isotig25292	10.589	4.366	7.166	4.475	1456	0.810560231	isogroup10383	ENSG00000147099	ENST00000373573	HDAC8
isotig25293	3.517	2.985	2.54	1.494	1454	0.318178402	isogroup10384	ENSG00000101333	ENST00000378501	PLCB4
isotig25294	4.142	2.81	2.346	1.522	1456	0.515330277	isogroup10385	ENSG00000261023	ENST00000565218	TRRAP.1
isotig25295	1.646	2.876	3.488	3.475	1456	0.125807845	isogroup10386	ENSG00000198324	ENST00000450786	FAM109A
isotig25297	9.903	5.778	8.776	6.212	1456	0.685259638	isogroup10388	ENSG00000064961	ENST00000333651	HMG20B
isotig25298	12.139	12.812	16.575	16.959	1456	0.021999699	isogroup10389	ENSG00000185928	ENST00000320330	C16orf53
isotig25299	2.236	2.379	1.661	1.492	1456	0.089576914	isogroup10390	ENSG00000198046	ENST00000342634	ZNF667
isotig25301	1.18	1.766	2.652	1.908	1455	0.132355151	isogroup10392	ENSG00000124615	ENST00000425303	MOCS1
isotig25302	1.757	5.743	3.325	4.339	1455	0.806567972	isogroup10393	ENSG00000162706	ENST00000368124	CADM3
isotig25303	2.91	2.891	2.36	2.016	1455	0.129912828	isogroup10394	ENSG00000186283	ENST00000367627	TOR3A
isotig25307	6.428	2.073	4.131	2.797	1454	0.779223341	isogroup10398	ENSG00000155287	ENST00000370495	SLC25A28
isotig25308	4.115	2.873	2.85	2.916	1451	0.371843766	isogroup10399	ENSG00000134058	ENST00000256443	CDK7
isotig25309	1.264	1.454	1.741	1.556	1453	0.042270985	isogroup10400	ENSG00000145348	ENST00000432496	TBCK
isotig25311	3.248	4.657	3.691	2.654	1451	0.082137221	isogroup10402	ENSG00000141349	ENST00000269097	G6PC3
isotig25313	2.32	1.698	2.72	1.564	1446	0.453191929	isogroup10404	ENSG00000213121	ENST00000392385	AL590867.1
isotig25314	3.003	2.967	1.897	1.775	1450	0.106053205	isogroup10405	ENSG00000205464	ENST00000439350	ATP6AP1L
isotig25318	3.214	1.71	3.548	1.854	1445	0.602023371	isogroup10409	ENSG00000071082	ENST00000409733	RPL31
isotig25319	2.867	2.706	3.44	2.039	1449	0.323279101	isogroup10410	ENSG00000240654	ENST00000382071	C1QTNF9
isotig25320	2.01	2.02	2.138	1.543	1451	0.111369956	isogroup10411	ENSG00000163611	ENST00000295872	SPICE1
isotig25321	6.872	2.972	9.632	5.41	1450	0.797484407	isogroup10412	ENSG00000131408	ENST00000411902	NR1H2
isotig25322	8.19	14.05	9.837	5.878	1451	0.15046592	isogroup10413	ENSG00000100075	ENST00000215882	SLC25A1
isotig25323	1.886	1.338	1.858	1.913	1451	0.254236492	isogroup10414	ENSG00000138111	ENST00000238936	TMEM180
isotig25324	3.926	3.872	3.081	2.625	1450	0.06991621	isogroup10415	ENSG00000130988	ENST00000397180	RGN
isotig25325	5.523	6.419	5.819	5.219	1450	0.073692418	isogroup10416	ENSG00000091947	ENST00000206380	TMEM101
isotig25326	6.859	3.032	4.61	3.551	1450	0.718503419	isogroup10417	ENSG00000115556	ENST00000450993	PLCD4
isotig25328	3.239	2.273	3.275	1.684	1449	0.496477418	isogroup10419	ENSG00000167674	ENST00000398364	AC011498.1
isotig25330	2.894	1.852	2.179	1.73	1449	0.372585857	isogroup10421	ENSG00000164404	ENST00000378673	GDF9
isotig25331	1.323	2.036	2.867	2.859	1449	0.021652138	isogroup10422	ENSG00000205441	ENST00000380102	KRTAP10-7
isotig25333	5.141	5.53	3.925	3.631	1449	0.0314684	isogroup10424	ENSG00000165476	ENST00000373758	REEP3
isotig25334	4.444	10.168	4.568	1.887	1447	0.462228526	isogroup10425	ENSG00000129596	ENST00000250535	CDO1
isotig25337	5.362	5.123	5.773	7.308	1449	0.085274667	isogroup10428	ENSG00000114767	ENST00000232888	RRP9
isotig25338	1.433	2.509	3.199	3.213	1449	0.112769595	isogroup10429	ENSG00000186472	ENST00000423517	PCLO
isotig25340	2.426	2.916	5.505	2.796	1449	0.474055009	isogroup10431	ENSG00000124608	ENST00000244571	AARS2
isotig25341	3.641	2.76	3.876	2.891	1448	0.416237694	isogroup10432	ENSG00000188687	ENST00000423644	SLC4A5
isotig25342	8.027	5.876	11.26	11.256	1448	0.358007064	isogroup10433	ENSG00000100429	ENST00000454936	HDAC10
isotig25343	70.857	47.448	85.075	57.559	1448	0.741733674	isogroup10434	ENSG00000168802	ENST00000306585	CHTF8
isotig25344	5.2	5.363	7.6	6.138	1448	0.154420606	isogroup10435	ENSG00000141295	ENST00000290216	SCRN2
isotig25345	2.46	1.567	2.032	1.147	1445	0.408619524	isogroup10436	ENSG00000152284	ENST00000282111	TCF7L1
isotig25349	13.677	10.603	13.782	11.944	1447	0.453314045	isogroup10440	ENSG00000167978	ENST00000426305	SRRM2
isotig25350	1.2	2.249	2.744	2.986	1447	0.183258811	isogroup10441	ENSG00000186212	ENST00000334306	ANKRD56
isotig25351	6.363	5.919	5.908	5.477	1447	0.075402044	isogroup10442	ENSG00000156469	ENST00000287025	MTERFD1
isotig25352	25.16	20.31	25.394	25.976	1447	0.252508078	isogroup10443	ENSG00000007314	ENST00000435607	SCN4A
isotig25353	19.433	6.88	4.479	3.187	1447	0.918135192	isogroup10444	ENSG00000076944	ENST00000320400	STXBP2
isotig25354	2.677	2.315	1.659	1.686	1447	0.034718569	isogroup10445	ENSG00000072042	ENST00000553384	RDH11
isotig25356	3.301	3.58	4.954	4.165	1446	0.017143233	isogroup10447	ENSG00000104880	ENST00000359920	ARHGEF18
isotig25358	2.863	2.411	2.878	2.757	1437	0.145271286	isogroup10449	ENSG00000042429	ENST00000427225	MED17
isotig25360	6.201	4.555	5.746	4.784	1446	0.444878635	isogroup10451	ENSG00000075891	ENST00000370296	PAX2
isotig25362	6.891	5.844	10.088	9.351	1446	0.147769971	isogroup10453	ENSG00000123159	ENST00000393033	GIPC1
isotig25363	4.917	4.396	2.362	1.889	1446	0.390396032	isogroup10454	ENSG00000123384	ENST00000243077	LRP1
isotig25364	8.583	15.188	26.26	17.584	1445	0.084889532	isogroup10455	ENSG00000256642	ENST00000539813	LINC00273
isotig25366	1.452	2.425	2.672	2.961	1444	0.263057038	isogroup10457	ENSG00000197915	ENST00000368801	HRNR
isotig25368	13.337	6.759	8.272	5.387	1442	0.758031487	isogroup10459	ENSG00000171282	ENST00000307745	BAHCC1
isotig25369	14.098	9.861	21.538	14.19	1442	0.608899451	isogroup10460	ENSG00000168066	ENST00000443908	SF1
isotig25370	3.637	5.101	5.064	5.411	1444	0.272798151	isogroup10461	ENSG00000100162	ENST00000215980	CENPM
isotig25372	1.592	3.39	3.493	3.568	1443	0.306567972	isogroup10463	ENSG00000185013	ENST00000406971	NT5C1B
isotig25373	2.825	3.353	2.958	2.577	1442	0.087162771	isogroup10464	ENSG00000181789	ENST00000314797	COPG
isotig25376	1.467	2.691	3.852	3.444	1443	0.145271286	isogroup10467	ENSG00000197380	ENST00000391916	DACT3
isotig25378	2.618	2.35	3.098	2.965	1442	0.135605321	isogroup10469	ENSG00000172273	ENST00000350777	HINFP
isotig25379	5.488	7.291	8.842	6.276	1441	0.157032013	isogroup10470	ENSG00000139428	ENST00000545712	MMAB
isotig25380	2.051	1.162	1.768	1.056	1441	0.238295634	isogroup10471	ENSG00000172071	ENST00000535951	EIF2AK3
isotig25385	4.628	4.053	3.585	2.788	1437	0.180957391	isogroup10476	ENSG00000068028	ENST00000395126	RASSF1
isotig25387	6.994	1.429	1.937	1.805	1439	0.812420155	isogroup10478	ENSG00000075643	ENST00000261326	MOCOS
isotig25388	19.818	16.483	22.183	15.158	1440	0.574425115	isogroup10479	ENSG00000175573	ENST00000530188	C11orf68
isotig25389	4.562	5.406	4.488	5.441	1440	0.264738484	isogroup10480	ENSG00000125843	ENST00000379567	C20orf29
isotig25390	6.601	3.08	4.655	2.363	1440	0.756838506	isogroup10481	ENSG00000129993	ENST00000268679	CBFA2T3
isotig25392	4.858	4.14	2.695	2.753	1439	0.029937251	isogroup10483	ENSG00000138594	ENST00000544199	TMOD3
isotig25396	3.719	3.254	3.503	2.94	1439	0.358147967	isogroup10487	ENSG00000104957	ENST00000221554	CCDC130
isotig25399	1.389	2.872	3.12	3.098	1438	0.287865409	isogroup10490	ENSG00000183248	ENST00000539422	AC010336.1
isotig25400	2	2.435	1.656	1.541	1438	0.130824002	isogroup10491	ENSG00000108306	ENST00000394294	FBXL20
isotig25402	22.11	18.412	18.889	17.063	1438	0.234989103	isogroup10493	ENSG00000105058	ENST00000263384	FAM32A
isotig25403	16.107	20.994	15.342	15.719	1437	0.144219208	isogroup10494	ENSG00000130522	ENST00000252818	JUND
isotig25404	3.599	6.067	9.111	7.073	1437	0.032651988	isogroup10495	ENSG00000023697	ENST00000428559	DERA
isotig25405	4.253	5.007	3.932	4.471	1437	0.258604494	isogroup10496	ENSG00000143319	ENST00000368219	ISG20L2
isotig25406	1.88	1.738	1.932	1.437	1434	0.156853536	isogroup10497	ENSG00000186448	ENST00000396058	ZNF197
isotig25407	3.335	5.868	7.774	7.852	1437	0.188143458	isogroup10498	ENSG00000111275	ENST00000552234	ALDH2
isotig25408	2.913	2.338	2.422	1.922	1433	0.200956264	isogroup10499	ENSG00000091009	ENST00000265271	RBM27
isotig25409	1.182	1.577	2.148	2.26	1436	0.13859247	isogroup10500	ENSG00000164442	ENST00000537332	CITED2
isotig25411	11.672	16.164	22.956	19.664	1439	0.023023597	isogroup10502	ENSG00000179922	ENST00000325351	ZNF784
isotig25412	15.569	18.341	17.302	14.899	1429	0.021999699	isogroup10503	ENSG00000104671	ENST00000221114	DCTN6
isotig25414	2.183	8.006	4.217	4.381	1436	0.673705568	isogroup10505	ENSG00000103876	ENST00000561421	FAH

isotig25416	2.477	4.205	2.946	3.198	1435	0.668614263	isogroup10507	ENSG00000152661	ENST00000282561	GJA1
isotig25417	5.65	5.902	6.383	5.71	1435	0.176298189	isogroup10508	ENSG00000103249	ENST00000382745	CLCN7
isotig25418	20.166	19.737	25.774	27.675	1435	0.070808597	isogroup10509	ENSG00000176108	ENST00000325167	CHMP6
isotig25419	4.064	2.587	2.162	2.137	1435	0.334457428	isogroup10510	ENSG00000134987	ENST00000513710	WDR36
isotig25421	3.133	5.907	7.211	6.744	1434	0.297944691	isogroup10512	ENSG00000099769	ENST00000568221	IGFALS
isotig25422	2.2	1.381	1.738	2.357	1434	0.045840535	isogroup10513	ENSG00000175595	ENST00000389138	ERCC4
isotig25423	2.702	1.089	1.352	2.529	1434	0.028237018	isogroup10514	ENSG00000130699	ENST00000252996	TAF4
isotig25424	14.874	10.154	15.864	9.944	1434	0.609970316	isogroup10515	ENSG00000186212	ENST00000334306	ANKRD56
isotig25426	3.855	2.825	3.204	1.747	1435	0.454629143	isogroup10517	ENSG00000163219	ENST00000543533	ARHGAP25
isotig25587	12.448	6.313	6.599	4.673	1399	0.738323815	isogroup10678	ENSG00000228867	ENST00000431322	NRM
isotig25588	3.868	3.919	4.369	3.089	1399	0.254621628	isogroup10679	ENSG00000198788	ENST00000441003	MUC2
isotig25590	5.421	3.848	4.221	2.814	1400	0.495434734	isogroup10681	ENSG00000198453	ENST00000415168	ZNF568
isotig25594	1.485	3.129	3.168	4.001	1399	0.394942511	isogroup10685	ENSG00000204839	ENST00000529971	C8orf73
isotig25595	7.665	8.367	9.833	8.001	1399	0.093907342	isogroup10686	ENSG00000213923	ENST00000400206	CSNK1E
isotig25596	3.54	5.66	2.257	2.896	1399	0.444906816	isogroup10687	ENSG00000088179	ENST00000263708	PTPN4
isotig25597	4.203	2.534	2.881	1.811	1397	0.507251822	isogroup10688	ENSG00000106536	ENST00000558333	POU6F2
isotig25598	9.263	7.589	9.53	6.074	1397	0.53432404	isogroup10689	ENSG00000185112	ENST00000329759	FAM43A
isotig25599	10.635	9.756	13.648	11.954	1399	0.26657962	isogroup10690	ENSG00000173020	ENST00000308595	ADRBK1
isotig25600	1.299	2.34	2.429	2.24	1399	0.147647855	isogroup10691	ENSG00000122729	ENST00000432017	ACO1
isotig25601	3.626	2.667	2.534	2.881	1399	0.156205381	isogroup10692	ENSG00000164180	ENST00000512429	TMEM161B
isotig25603	1.913	2.435	1.439	1.184	1399	0.095898775	isogroup10694	ENSG00000151689	ENST00000541441	INPP1
isotig25604	6.414	3.935	7.119	3.283	1398	0.653424889	isogroup10695	ENSG00000129226	ENST00000250092	CD68
isotig25605	2.77	2.215	3.002	2.329	1384	0.33795183	isogroup10696	ENSG00000156983	ENST00000383829	BRPF1
isotig25607	17.494	15.2	16.824	17.171	1395	0.084992861	isogroup10698	ENSG00000116096	ENST00000234454	SPR
isotig25612	3.044	4.639	3.279	3.009	1391	0.391344781	isogroup10703	ENSG00000213066	ENST00000366847	FGFR1OP
isotig25613	5.036	3.818	2.394	2.325	1396	0.161428196	isogroup10704	ENSG00000137942	ENST00000370256	FNBP1L
isotig25619	2.777	1.61	3.151	2.472	1395	0.407999549	isogroup10710	ENSG00000157617	ENST00000380486	C2CD2
isotig25620	1.589	2.51	3.057	3.37	1395	0.174287969	isogroup10711	ENSG00000130254	ENST00000252542	SAFB2
isotig25621	12.923	11.333	13.829	9.427	1394	0.322208236	isogroup10712	ENSG00000214279	ENST00000333815	RP11-108K14.4.1
isotig25623	3.209	3.537	3.775	2.457	1394	0.157802285	isogroup10714	ENSG00000068831	ENST00000394432	RASGRP2
isotig25624	1.246	2.826	3.444	3.581	1394	0.304463816	isogroup10715	ENSG00000153902	ENST00000392225	LGI4
isotig25626	4.874	3.006	4.228	2.732	1393	0.594837304	isogroup10717	ENSG00000168542	ENST00000304636	COL3A1
isotig25630	1.994	2.147	2.136	2.718	1392	0.233570677	isogroup10721	ENSG00000168538	ENST00000357207	TRAPPC11
isotig25631	4.347	3.583	3.78	2.509	1392	0.389757271	isogroup10722	ENSG00000135439	ENST00000547588	AGAP2
isotig25632	9.654	9.764	11.148	7.742	1388	0.386093785	isogroup10723	ENSG00000136104	ENST00000336617	RNASEH2B
isotig25634	4.566	3.216	2.894	3.556	1391	0.087961223	isogroup10725	ENSG00000120925	ENST00000534961	RNF170
isotig25636	11.055	6.651	5.058	2.495	1391	0.589266927	isogroup10727	ENSG00000126016	ENST00000524145	AMOT
isotig25638	95.872	13.69	15.487	22.596	1389	0.977878184	isogroup10729	ENSG00000196169	ENST00000389916	KIF19
isotig25640	10.977	3.341	7.811	5.669	1390	0.821165552	isogroup10731	ENSG00000166987	ENST00000431731	MBD6
isotig25643	24.851	22.361	24.678	18.815	1379	0.413616893	isogroup10734	ENSG00000118363	ENST00000263672	SPCS2
isotig25644	4.27	5.207	3.975	3.014	1389	0.140912678	isogroup10735	ENSG00000165669	ENST00000369172	FAM204A
isotig25646	2.543	5.971	3.068	2.481	1390	0.593681897	isogroup10737	ENSG00000127955	ENST00000457358	GNAI1
isotig25647	1.553	2.028	2.486	2.885	1389	0.155763884	isogroup10738	ENSG00000196177	ENST00000358776	ACADSB
isotig25648	3.595	4.029	2.698	1.886	1389	0.006660029	isogroup10739	ENSG00000130307	ENST00000324554	USHBP1
isotig25652	7.68	4.82	6.448	5.54	1386	0.441966634	isogroup10743	ENSG00000110801	ENST00000541212	PSMD9
isotig25655	15.079	10.642	17.64	20.839	1388	0.173395581	isogroup10746	ENSG00000143368	ENST00000271628	SF3B4
isotig25656	10.05	8.303	8.387	6.311	1387	0.516805065	isogroup10747	ENSG00000142444	ENST00000270502	C19orf52
isotig25659	25.96	30.422	26.016	23.681	1384	0.008313294	isogroup10750	ENSG00000135972	ENST00000258455	MRPS9
isotig25660	2.061	2.706	1.439	1.405	1386	0.164396558	isogroup10751	ENSG00000090020	ENST00000374086	SLC9A1
isotig25663	5.188	6.06	4.035	4.487	1382	0.231297437	isogroup10754	ENSG00000117862	ENST00000371626	TXNDC12
isotig25665	3.185	2.733	1.039	1.728	1386	0.021652138	isogroup10756	ENSG00000104133	ENST00000558319	SPG11
isotig25667	5.692	2.595	3.104	2.504	1386	0.686668671	isogroup10758	ENSG00000136925	ENST00000341170	TSTD2
isotig25669	6.066	6.139	6.887	6.122	1386	0.089144811	isogroup10760	ENSG00000163481	ENST00000295704	RNF25
isotig25670	10.807	3.996	7.554	9.077	1386	0.626286917	isogroup10761	ENSG00000136383	ENST00000258888	ALPK3
isotig25671	1.067	2.075	2.733	2.053	1385	0.028237018	isogroup10762	ENSG00000131149	ENST00000393243	KIAA0182
isotig25673	22.408	20.243	24.584	20.803	1385	0.349674983	isogroup10764	ENSG00000177830	ENST00000323541	CHID1
isotig25674	8.032	3.628	10.066	3.313	1385	0.898202074	isogroup10765	ENSG00000226618	ENST00000550382	PRRC2A
isotig25676	2.206	3.312	4.906	4.768	1385	0.165260765	isogroup10767	ENSG00000101544	ENST00000262198	ADNP2
isotig25678	3.723	3.879	3.349	4.675	1384	0.201397761	isogroup10769	ENSG00000213689	ENST00000422277	TREX1
isotig25679	22.878	8.904	11.458	10.099	1381	0.828454949	isogroup10770	ENSG00000240542	ENST00000398470	KRTAP9-1
isotig25680	8.11	7.688	11.794	7.618	1384	0.48611633	isogroup10771	ENSG00000047849	ENST00000426837	MAP4
isotig25681	3.91	1.664	2.922	2.928	1384	0.576670174	isogroup10772	ENSG00000130803	ENST00000247956	ZNF317
isotig25682	1.661	2.064	1.362	1.262	1383	0.157933794	isogroup10773	ENSG00000068885	ENST00000496589	IFT80
isotig25683	38.091	9.579	9.867	3.274	1381	0.960434358	isogroup10774	ENSG00000078814	ENST00000262873	MYH7B
isotig25684	3.74	2.615	4.232	3.189	1383	0.500685729	isogroup10775	ENSG00000090539	ENST00000450923	CHRD
isotig25685	9.011	6.069	6.636	8.191	1383	0.311593522	isogroup10776	ENSG00000107331	ENST00000265662	ABCA2
isotig25687	2.5	2.534	3.34	3.515	1382	0.035704892	isogroup10778	ENSG00000261493	ENST00000568937	REXO4.1
isotig25689	4.469	3.694	4.279	4.377	1381	0.054877132	isogroup10780	ENSG00000111707	ENST00000543473	SUDS3
isotig25691	2.78	3.244	2.544	2.175	1379	0.188754039	isogroup10782	ENSG00000137842	ENST00000564494	TMEM62
isotig25692	12.451	9.235	12.02	9.873	1381	0.392368678	isogroup10783	ENSG00000103496	ENST00000313843	STX4
isotig26087	10.054	5.345	11.062	7.16	1307	0.70464793	isogroup11178	ENSG00000164853	ENST00000316333	UNCX
isotig26088	3.68	1.819	2.527	1.951	1308	0.562608026	isogroup11179	ENSG00000161681	ENST00000391813	SHANK1
isotig26089	8.764	4.671	9.783	4.125	1308	0.823279101	isogroup11180	ENSG00000203485	ENST00000392634	INF2
isotig26091	1.695	4.365	3.797	2.259	1308	0.603667243	isogroup11182	ENSG00000196666	ENST00000538490	FAM180B
isotig26092	4.584	5.403	3.447	3.357	1307	0.089520553	isogroup11183	ENSG00000180957	ENST00000335272	PITPNB
isotig26093	4.324	2.155	3.278	2.107	1307	0.619298114	isogroup11184	ENSG00000183337	ENST00000406200	BCOR
isotig26094	4.193	6.739	4.061	2.487	1305	0.375742091	isogroup11185	ENSG00000175274	ENST00000533940	TP53I11
isotig26095	1.574	2.141	1.836	1.936	1307	0.3366837	isogroup11186	ENSG00000091656	ENST00000521891	ZFHX4
isotig26097	2.049	1.56	1.682	2.005	1306	0.204244007	isogroup11188	ENSG00000100207	ENST00000359486	TCF20
isotig26098	10.337	3.046	4.962	3.857	1306	0.831254227	isogroup11189	ENSG00000131149	ENST00000405402	KIAA0182
isotig26099	3.701	1.123	3.591	1.423	1306	0.766645375	isogroup11190	ENSG00000109572	ENST00000515420	CLCN3
isotig26100	1.544	2.317	3.464	3.462	1306	0.115756745	isogroup11191	ENSG00000198788	ENST00000359061	MUC2

isotig26101	5.199	6.325	4.227	4.234	1306	0.266711129	isogroup11192	ENSG00000146425	ENST00000367089	DYNLT1
isotig26102	35.483	45.854	64.344	67.742	1308	0.274780191	isogroup11193	ENSG00000161681	ENST00000391813	SHANK1
isotig26104	34.877	24.329	20.983	20.208	1305	0.365343428	isogroup11195	ENSG00000185825	ENST00000458587	BCAP31
isotig26105	17.876	24.894	13.743	21.17	1306	0.639945142	isogroup11196	ENSG00000128309	ENST00000397129	MPST
isotig26106	2.893	5.032	6.41	5.541	1305	0.093738258	isogroup11197	ENSG00000147576	ENST00000415254	ADHFE1
isotig26108	7.438	5.572	2.66	3.534	1305	0.131340648	isogroup11199	ENSG00000251322	ENST00000445220	SHANK3
isotig26110	2.221	2.788	2.804	3.262	1304	0.308643947	isogroup11201	ENSG00000156990	ENST00000383820	RPUSD3
isotig26111	4.423	3.457	6.254	5.747	1305	0.289274442	isogroup11202	ENSG00000100092	ENST00000357436	SH3BP1
isotig26112	3.489	2.698	1.67	1.818	1305	0.123215225	isogroup11203	ENSG00000114573	ENST00000273398	ATP6V1A
isotig26113	4.319	3.27	3.512	2.712	1304	0.40805591	isogroup11204	ENSG00000125648	ENST00000334510	SLC25A23
isotig26114	1.823	2.876	3.923	3.537	1304	0.110749981	isogroup11205	ENSG00000235758	ENST00000549912	AGPAT1
isotig26117	132.813	13.837	15.137	9.734	1304	0.985486962	isogroup11208	ENSG00000205890	ENST00000382225	AC108134.1
isotig26118	2.332	1.709	2.147	1.869	1303	0.342413767	isogroup11209	ENSG00000185736	ENST00000381310	ADARB2
isotig26119	2.391	1.622	2.678	2.921	1303	0.364413467	isogroup11210	ENSG00000213121	ENST00000392385	AL590867.1
isotig26120	3.201	3.459	4.39	2.987	1304	0.108561284	isogroup11211	ENSG00000105137	ENST00000342784	SYDE1
isotig26121	9.808	6.511	10.622	5.256	1295	0.76216465	isogroup11212	ENSG00000126953	ENST00000372902	TIMM8A
isotig26122	3.111	2.367	2.593	1.598	1303	0.359650936	isogroup11213	ENSG00000166503	ENST00000299633	RP11-382A20.3.1
isotig26123	3.451	2.602	3.12	1.995	1302	0.315416698	isogroup11214	ENSG00000144229	ENST00000413152	THSD7B
isotig26124	1.502	2.147	1.474	1.735	1303	0.367579094	isogroup11215	ENSG00000133069	ENST00000358024	TMCC2
isotig26125	3.792	2.985	2.101	2.839	1301	0.135605321	isogroup11216	ENSG00000061987	ENST00000552115	MON2
isotig26128	5.941	6.379	2.086	2.444	1301	0.267199594	isogroup11219	ENSG00000166265	ENST00000299340	CYYR1
isotig26129	1.643	3.072	3.449	3.045	1301	0.13943789	isogroup11220	ENSG00000108828	ENST00000355653	VAT1
isotig26130	7.835	10.22	8.281	10.402	1300	0.49828098	isogroup11221	ENSG00000146109	ENST00000274849	ABT1
isotig26131	8.972	10.249	7.67	7.008	1300	0.034840685	isogroup11222	ENSG00000119820	ENST00000238831	YIPF4
isotig26134	2.916	2.458	3.037	2.633	1301	0.219818517	isogroup11225	ENSG00000100726	ENST00000262319	TELO2
isotig26138	10.942	12.003	10.474	10.432	1294	0.095795446	isogroup11229	ENSG00000124380	ENST00000450162	SNRNP27
isotig26139	1.812	3.628	3.974	3.82	1301	0.299889156	isogroup11230	ENSG00000241935	ENST00000370646	HOGA1
isotig26140	2.55	2.592	1.889	2.618	1300	0.299081311	isogroup11231	ENSG00000048991	ENST00000409606	R3HDM1
isotig26142	2.379	2.165	2.824	3.115	1301	0.077224393	isogroup11233	ENSG00000133935	ENST00000256319	C14orf1
isotig26145	25.117	38.185	24.913	27.295	1300	0.569080183	isogroup11236	ENSG00000158710	ENST00000368096	TAGLN2
isotig26147	16.376	20.674	18.01	15.137	1300	0.0383163	isogroup11238	ENSG00000138629	ENST00000565335	UBL7
isotig26149	3.172	2.994	4.442	2.727	1297	0.340751108	isogroup11240	ENSG00000109881	ENST00000328697	CCDC34
isotig26150	5.976	4.949	5.625	4.443	1300	0.294919967	isogroup11241	ENSG00000130723	ENST00000357304	PRRC2B
isotig26151	4.163	3.192	4.801	2.566	1300	0.505373112	isogroup11242	ENSG00000021762	ENST00000525498	OSBPL5
isotig26587	24.423	24.734	31.596	29.126	1229	0.117729391	isogroup11678	ENSG00000172500	ENST00000357519	FIBP
isotig26591	1.005	1.962	2.368	3.035	1232	0.235092433	isogroup11682	ENSG00000158158	ENST00000377075	CNNM4
isotig26592	2.095	1.975	2.243	4.225	1231	0.193911099	isogroup11683	ENSG00000177409	ENST00000437805	SAMD9L
isotig26593	7.625	10.901	10.74	9.439	1231	0.388376418	isogroup11684	ENSG00000153774	ENST00000283882	CFDP1
isotig26595	2.434	4.933	2.67	2.691	1231	0.515086045	isogroup11686	ENSG00000135929	ENST00000258415	CYP27A1
isotig27088	5.07	4.783	1.12	2.114	1154	0.025447133	isogroup12179	ENSG00000091039	ENST00000446075	OSBPL8
isotig27089	8.123	10.438	7.909	7.984	1158	0.270027053	isogroup12180	ENSG00000169740	ENST00000395797	ZNF32
isotig27090	1.29	1.147	1.289	1.214	1161	0.042674908	isogroup12181	ENSG00000198939	ENST00000523286	ZFP2
isotig27092	2.907	2.459	2.243	1.678	1161	0.221302698	isogroup12183	ENSG00000174227	ENST00000506402	PIGG
isotig27093	5.608	3.771	6.409	5.264	1161	0.508519952	isogroup12184	ENSG00000156860	ENST00000568722	FBRS
isotig27094	11.542	6.607	8.604	6.961	1161	0.547052303	isogroup12185	ENSG00000185477	ENST00000333209	GPRIN3
isotig27097	1.549	3.298	4.329	4.265	1160	0.247510709	isogroup12188	ENSG00000162572	ENST00000379101	SCNN1D
isotig27098	1.274	2.428	2.642	2.625	1160	0.069775306	isogroup12189	ENSG00000212857	ENST00000391545	AL356585.1
isotig27099	17.647	14.243	10.124	4.671	1160	0.743452694	isogroup12190	ENSG00000230128	ENST00000450236	IER3
isotig27100	2.203	1.74	1.512	1.621	1160	0.028237018	isogroup12191	ENSG00000221818	ENST00000520164	EBF2
isotig27101	2.29	1.838	1.926	3.242	1160	0.028237018	isogroup12192	ENSG00000157873	ENST00000355716	TNFRSF14
isotig27102	4.476	2.609	3.136	3.397	1160	0.296479297	isogroup12193	ENSG00000105298	ENST00000429344	C19orf29
isotig27105	4.118	3.399	5.528	5.659	1159	0.290768017	isogroup12196	ENSG00000143633	ENST00000366649	C1orf131
isotig27106	29.431	34.078	6.07	3.013	1159	0.008040881	isogroup12197	ENSG00000123728	ENST00000370874	RAP2C
isotig27588	1.118	2.149	2.852	2.689	1107	0.166209514	isogroup12679	ENSG00000241233	ENST00000398534	KRTAP5-8
isotig27589	2.922	5.502	3.086	2.103	1107	0.485205155	isogroup12680	ENSG00000169515	ENST00000307522	CCDC8
isotig27590	2.039	5.932	3.97	1.881	1107	0.469236116	isogroup12681	ENSG00000147642	ENST00000533171	SYBU
isotig27592	4.027	5.713	3.572	1.902	1106	0.294497257	isogroup12683	ENSG00000108821	ENST00000225964	COL1A1
isotig27593	3.82	5.28	2.762	3.004	1103	0.351816713	isogroup12684	ENSG00000054267	ENST00000366603	ARID4B
isotig27597	4.388	4.526	5.242	4.089	1106	0.202806793	isogroup12688	ENSG00000101811	ENST00000415585	CSTF2
isotig27598	2.149	1.17	1.97	1.7	1106	0.288119035	isogroup12689	ENSG00000104774	ENST00000536796	MAN2B1
isotig27599	13.182	10.154	2.487	3.785	1106	0.277419779	isogroup12690	ENSG00000180818	ENST00000303460	HOXC10
isotig27600	2.618	1.532	2.822	2.753	1106	0.313040129	isogroup12691	ENSG00000090924	ENST00000458508	PLEKHG2
isotig28087	3.389	3.502	2.711	2.433	1049	0.200054483	isogroup13178	ENSG00000150764	ENST00000440460	DIXDC1
isotig28088	1.278	2.036	1.358	1.069	1047	0.18463027	isogroup13179	ENSG00000186469	ENST00000553432	GNG2
isotig28090	1.142	3.466	1.743	2.603	1049	0.779899677	isogroup13181	ENSG00000154175	ENST00000495063	ABI3BP
isotig28095	16.407	15.166	20.297	16.171	1048	0.381556699	isogroup13186	ENSG00000013583	ENST00000014930	HEBP1
isotig28096	3.846	1.942	1.901	1.75	1049	0.446353423	isogroup13187	ENSG00000198722	ENST00000396787	UNC13B
isotig28097	1.218	2.304	2.816	2.625	1049	0.111369956	isogroup13188	ENSG00000105464	ENST00000263269	GRIN2D
isotig28098	1.432	1.984	3.407	2.633	1046	0.095447885	isogroup13189	ENSG00000179168	ENST00000334928	GGN
isotig28099	1.925	2.089	2.167	2.2	1048	0.028237018	isogroup13190	ENSG00000168795	ENST00000307750	ZBTB5
isotig28100	4.155	1.381	3.489	3.546	1048	0.421216277	isogroup13191	ENSG00000084731	ENST00000455394	KIF3C
isotig28102	5.031	1.471	6.572	4.208	1048	0.734801232	isogroup13193	ENSG00000135451	ENST00000551245	TROAP
isotig28103	4.364	1.852	2.678	2.285	1048	0.550678214	isogroup13194	ENSG00000152217	ENST00000282030	SETBP1
isotig28105	2.991	3.651	3.63	2.67	1048	0.017143233	isogroup13196	ENSG00000133997	ENST00000554963	MED6
isotig28106	1.62	2.306	1.991	1.581	1048	0.277006463	isogroup13197	ENSG00000176438	ENST00000334258	C14orf49
isotig28108	1.353	2.143	2.537	2.563	1048	0.045840535	isogroup13199	ENSG00000060718	ENST00000512756	COL11A1
isotig28111	4.673	3.509	6.649	1.989	1047	0.448664237	isogroup13202	ENSG00000132470	ENST00000449880	ITGB4
isotig28112	1.45	2.527	3.033	3.164	1047	0.263179154	isogroup13203	ENSG00000074800	ENST00000234590	ENO1
isotig28113	2.139	1.638	2.648	1.413	1046	0.346772375	isogroup13204	ENSG00000182118	ENST00000366654	FAM89A
isotig28116	27.01	23.379	23.773	24.888	1047	0.252930788	isogroup13207	ENSG00000184897	ENST00000333762	H1FX
isotig28118	5.284	3.982	3.88	3.763	1047	0.345870594	isogroup13209	ENSG00000147475	ENST00000519638	ERLIN2
isotig28119	2.613	2.2	1.375	1.711	1047	0.059301495	isogroup13210	ENSG00000198265	ENST00000358691	HELZ

isotig28120	1.907	2.964	3.844	3.485	1047	0.06991621	isogroup13211	ENSG00000143384	ENST00000307940	MCL1
isotig28122	3.665	4.258	2.383	2.825	1046	0.208020215	isogroup13213	ENSG00000214655	ENST00000412198	KIAA0913
isotig28125	1.89	3.986	4.872	3.938	1046	0.218043135	isogroup13216	ENSG00000130957	ENST00000375337	FBP2
isotig28126	2.941	4.641	6.584	6.25	1046	0.131340648	isogroup13217	ENSG00000153902	ENST00000392225	LGI4
isotig28127	1.107	1.984	2.259	3.146	1046	0.270543699	isogroup13218	ENSG00000151093	ENST00000280701	OXSM
isotig28128	4.201	6.715	2.048	3.446	1046	0.625319381	isogroup13219	ENSG00000205213	ENST00000379214	LGR4
isotig28129	1.279	2.22	2.489	2.633	1046	0.189618246	isogroup13220	ENSG00000020181	ENST00000416514	GPR124
isotig28131	1.718	2.875	3.495	3.36	1046	0.122942812	isogroup13222	ENSG00000100577	ENST00000553586	GSTZ1
isotig28132	3.303	2.075	3.249	1.562	1046	0.571823101	isogroup13223	ENSG00000115183	ENST00000454300	TANC1
isotig28133	2.922	1.837	2.859	2.804	1046	0.173724356	isogroup13224	ENSG00000072609	ENST00000537522	CHFR
isotig28134	3.181	5.188	2.835	3.932	1042	0.535376118	isogroup13225	ENSG00000132541	ENST00000254878	HRSP12
isotig28135	22.974	17.721	32.932	32.122	1044	0.35238972	isogroup13226	ENSG00000165238	ENST00000448251	WNK2
isotig28136	5.714	7.796	7.686	10.818	1045	0.495913805	isogroup13227	ENSG00000171443	ENST00000301073	ZNF524
isotig28137	2.064	2.659	2.386	2.035	1045	0.177697828	isogroup13228	ENSG00000136213	ENST00000258711	CHST12
isotig28140	2.045	2.258	1.432	1.992	1045	0.143232885	isogroup13231	ENSG00000139687	ENST00000542917	RB1
isotig28141	2.179	1.129	2.332	2.7	1045	0.172869542	isogroup13232	ENSG00000136828	ENST00000438723	RALGPS1
isotig28142	1.492	3.136	1.291	2.145	1044	0.700308109	isogroup13233	ENSG00000173548	ENST00000308527	SNX33
isotig28146	5.414	6.563	7.994	4.953	1044	0.140010897	isogroup13237	ENSG00000109046	ENST00000262394	WSB1
isotig28148	5.031	2.242	3.767	1.951	1044	0.682216127	isogroup13239	ENSG00000125378	ENST00000559087	BMP4
isotig28149	1.301	2.261	3.148	3.281	1044	0.214623882	isogroup13240	ENSG00000174444	ENST00000569696	RPL4
isotig28150	1.836	2.206	2.76	2.187	1044	0.102201849	isogroup13241	ENSG00000253304	ENST00000521452	TMEM200B
isotig28152	1.838	1.406	1.345	1.502	1043	0.010060494	isogroup13243	ENSG00000196576	ENST00000399991	PLXNB2
isotig28154	5.093	1.917	3.523	2.104	1043	0.719423987	isogroup13245	ENSG00000175115	ENST00000529757	PACS1
isotig28155	1.283	1.953	2.319	2.919	1043	0.256556699	isogroup13246	ENSG00000148187	ENST00000394315	MRRF
isotig28157	1.742	3.194	3.576	3.692	1043	0.259196288	isogroup13248	ENSG00000100429	ENST00000454936	HDAC10
isotig28161	7.444	3.876	7.077	4.646	1041	0.747031637	isogroup13252	ENSG00000131669	ENST00000375446	NINJ1
isotig28163	2.281	3.836	2.799	3.481	1042	0.541068611	isogroup13254	ENSG00000160049	ENST00000377036	DFFA
isotig28164	8.606	8.238	6.22	2.406	1042	0.523126926	isogroup13255	ENSG00000148840	ENST00000413464	PPRC1
isotig28167	2.472	1.937	1.772	2.084	1042	0.06751146	isogroup13258	ENSG00000132970	ENST00000361042	WASF3
isotig28168	1.418	2.046	3.083	2.836	1042	0.025447133	isogroup13259	ENSG00000204060	ENST00000372591	FOXO6
isotig28172	1.188	2.01	2.41	2.492	1042	0.119880514	isogroup13263	ENSG00000136824	ENST00000536893	SMC2
isotig28173	20.7	22.271	37.693	33.376	1041	0.365916435	isogroup13264	ENSG00000177606	ENST00000371222	JUN
isotig28175	1.037	1.812	1.208	1.463	1040	0.32851131	isogroup13266	ENSG00000020922	ENST00000393241	MRE11A
isotig28181	4.681	2.889	1.207	1.764	1041	0.281440219	isogroup13272	ENSG00000106772	ENST00000428286	PRUNE2
isotig28182	1.439	2.67	3.175	3.742	1041	0.221321485	isogroup13273	ENSG00000072778	ENST00000543245	ACADVL
isotig28183	2.264	3.548	3.158	2.323	1041	0.309113624	isogroup13274	ENSG00000186716	ENST00000305877	BCR
isotig28186	1.401	2.267	2.377	2.646	1041	0.222993537	isogroup13277	ENSG00000153902	ENST00000392225	LGI4
isotig28188	1.113	2.105	2.362	2.605	1040	0.263780341	isogroup13279	ENSG00000131269	ENST00000535115	ABCB7
isotig28189	3.744	2.453	4.367	2.992	1040	0.567520854	isogroup13280	ENSG00000115257	ENST00000300954	PCSK4
isotig28190	3.714	10.603	1.805	3.306	1043	0.864986473	isogroup13281	ENSG00000183248	ENST00000539422	AC010336.1
isotig28192	2.209	1.483	2.628	1.356	1040	0.424175246	isogroup13283	ENSG00000187479	ENST00000528572	C11orf96
isotig28193	1.689	1.428	2.131	1.83	1040	0.059301495	isogroup13284	ENSG00000135899	ENST00000540870	SP110
isotig28198	7.804	3.737	6.523	5.603	1039	0.654185767	isogroup13289	ENSG00000143971	ENST00000272342	ETAA1
isotig28199	4.844	4.342	5.331	4.525	1039	0.230442624	isogroup13290	ENSG00000142039	ENST00000269967	CCDC97
isotig28203	3.671	2.381	3.305	2.693	1039	0.407698955	isogroup13294	ENSG00000005238	ENST00000378557	FAM214B
isotig28204	6.055	2.583	5.758	3.921	1039	0.736304201	isogroup13295	ENSG00000006062	ENST00000344686	MAP3K14
isotig28205	11.871	7.72	8.04	7.807	1038	0.437429548	isogroup13296	ENSG00000115446	ENST00000409975	UNC50
isotig28206	1.289	2.567	2.935	3.688	1038	0.371965883	isogroup13297	ENSG00000143631	ENST00000368799	FLG
isotig28207	1.096	1.32	2.081	1.661	1038	0.061255354	isogroup13298	ENSG00000187166	ENST00000335017	H1FNT
isotig28208	5.868	6.418	3.86	4.551	1038	0.370434734	isogroup13299	ENSG00000162733	ENST00000367922	DDR2
isotig28209	48.465	42.323	37.445	48.289	1038	0.227436687	isogroup13300	ENSG00000136732	ENST00000409836	GYPC
isotig28210	3.084	1.948	4.794	4.063	1036	0.432826708	isogroup13301	ENSG00000080603	ENST00000395059	SRCAP
isotig28211	2.829	3.099	3.611	2.07	1038	0.304989855	isogroup13302	ENSG00000169180	ENST00000565698	XPO6
isotig28212	2.482	3.355	2.259	4.853	1038	0.544581799	isogroup13303	ENSG00000141664	ENST00000269499	ZCCHC2
isotig28215	2.879	2.46	2.333	3.087	1037	0.074481476	isogroup13306	ENSG00000100100	ENST00000441972	PIK3IP1
isotig28217	5.667	5.236	4.545	3.09	1036	0.217629819	isogroup13308	ENSG00000197976	ENST00000381261	AKAP17A
isotig28218	1.155	2.257	1.923	2.18	1037	0.21596716	isogroup13309	ENSG00000167766	ENST00000545872	ZNF83
isotig28223	1.81	2.9	2.867	2.656	1037	0.194089577	isogroup13314	ENSG00000180438	ENST00000429201	TPRXL
isotig28229	73.964	71.512	48.217	42.968	1029	0.228873901	isogroup13320	ENSG00000170634	ENST00000394666	ACYP2
isotig28231	1.465	2.333	2.478	2.701	1036	0.139334561	isogroup13322	ENSG00000018280	ENST00000539932	SLC11A1
isotig28234	1.409	2.097	2.98	3.136	1035	0.260126249	isogroup13325	ENSG00000162572	ENST00000379101	SCNN1D
isotig28237	1.409	2.483	3.14	3.439	1035	0.224674983	isogroup13328	ENSG00000165879	ENST00000371021	FRAT1
isotig28240	2.239	4.248	6.155	5.883	1035	0.188096491	isogroup13331	ENSG00000189001	ENST00000452271	SBSN
isotig28241	1.504	1.267	1.996	1.21	1036	0.246327121	isogroup13332	ENSG00000159069	ENST00000433269	FBXW5
isotig28243	3.126	2.575	1.981	1.947	1035	0.204178252	isogroup13334	ENSG00000134109	ENST00000445686	EDEM1
isotig28245	3.032	2.798	3.053	2.251	1034	0.263686406	isogroup13336	ENSG00000173638	ENST00000485649	SLC19A1
isotig28247	1.236	2.172	2.768	2.403	1034	0.084617119	isogroup13338	ENSG00000089169	ENST00000548866	RPH3A
isotig28248	3.051	3.387	1.804	1.905	1034	0.167580972	isogroup13339	ENSG00000125746	ENST00000536630	EML2
isotig28250	2.221	2.135	3.393	2.187	1034	0.275813482	isogroup13341	ENSG00000101224	ENST00000439880	CDC25B
isotig28251	2.453	2.246	1.232	1.255	1034	0.100435861	isogroup13342	ENSG00000151327	ENST00000396472	FAM177A1
isotig28252	7.475	7.419	8.196	6.127	1034	0.346603292	isogroup13343	ENSG00000126562	ENST00000246914	WNK4
isotig28255	5.646	3.685	3.592	3.164	1033	0.448241527	isogroup13346	ENSG00000177200	ENST00000564845	CHD9
isotig28257	1.024	1.64	2.253	2.341	1033	0.078182536	isogroup13348	ENSG00000111432	ENST00000539839	FZD10
isotig28261	2.206	3.136	2.326	2.928	1032	0.301730292	isogroup13352	ENSG00000114904	ENST00000383721	NEK4
isotig28263	3.648	2.14	2.702	2.452	1032	0.339201172	isogroup13354	ENSG00000122483	ENST00000557479	CCDC18
isotig28267	1.607	2.324	2.898	3.037	1032	0.028237018	isogroup13358	ENSG00000125877	ENST00000380113	ITPA
isotig28269	4.006	1.862	2.469	2.69	1032	0.319324416	isogroup13360	ENSG00000167548	ENST00000301067	MLL2
isotig28271	33.188	4.399	25.063	10.979	1031	0.964417224	isogroup13362	ENSG00000117983	ENST00000537836	MUC5B
isotig28273	2.073	1.367	1.558	3.604	1031	0.124586684	isogroup13364	ENSG00000183347	ENST00000370456	GBP6
isotig28274	69.791	55.423	68.361	50.419	1031	0.550199143	isogroup13365	ENSG00000101421	ENST00000217402	CHMP4B
isotig28275	1.259	1.827	2.723	2.497	1031	0.05275419	isogroup13366	ENSG00000177679	ENST00000388802	SRRM3
isotig28276	3.429	1.292	2.471	1.781	1031	0.60482265	isogroup13367	ENSG00000010292	ENST00000545962	NCAPD2

isotig28277	20.514	22.25	14.001	18.018	1030	0.43463027	isogroup13368	ENSG00000175606	ENST00000312184	TMEM70
isotig28279	1.317	2.252	2.919	3.018	1031	0.167487037	isogroup13370	ENSG00000205277	ENST00000536621	MUC12
isotig28280	1.531	2.326	3.367	3.496	1031	0.19021004	isogroup13371	ENSG00000165672	ENST00000356951	PRDX3
isotig28281	6.189	6.093	4.847	5.659	1030	0.138761554	isogroup13372	ENSG00000006625	ENST00000275428	GGCT
isotig28285	1.318	1.884	2.563	3.13	1030	0.172869542	isogroup13376	ENSG00000212935	ENST00000391620	KRTAP10-3
isotig28287	1.144	1.682	2.313	2.109	1030	0.034718569	isogroup13378	ENSG00000186493	ENST00000397835	C5orf38
isotig28289	6.54	4.736	6.155	4.569	1029	0.523568423	isogroup13380	ENSG00000213015	ENST00000545125	ZNF580
isotig28291	1.553	3.145	3.464	4.569	1029	0.430328023	isogroup13382	ENSG00000113889	ENST00000265023	KNG1
isotig28295	5.88	6.677	7.644	6.179	1029	0.0314684	isogroup13386	ENSG00000254901	ENST00000462790	MEF2BNB
isotig28300	2.761	2.298	2.211	1.765	1027	0.140236342	isogroup13391	ENSG00000141013	ENST00000268699	GAS8
isotig28305	2.061	2.78	1.366	1.831	1027	0.401940708	isogroup13396	ENSG00000106299	ENST00000223023	WASL
isotig28307	3.656	2.465	1.618	1.395	1027	0.368734501	isogroup13398	ENSG00000151623	ENST00000544252	NR3C2
isotig28308	1.828	3.095	4.477	3.989	1027	0.115456151	isogroup13399	ENSG00000164077	ENST00000455683	MON1A
isotig28309	7.66	5.148	7.945	5.269	1026	0.629621628	isogroup13400	ENSG00000182095	ENST00000430969	TNRC18
isotig28310	3.056	2.801	2.97	2.16	1026	0.158844969	isogroup13401	ENSG00000139613	ENST00000394023	SMARCC2
isotig28311	44.866	48.365	46.396	66.157	1026	0.38642256	isogroup13402	ENSG00000181856	ENST00000317370	SLC2A4
isotig28314	1.432	1.375	2.928	1.037	1025	0.42749117	isogroup13405	ENSG00000123095	ENST00000540731	BHLHE41
isotig28316	1.109	1.818	2.717	2.618	1026	0.087435184	isogroup13407	ENSG00000160200	ENST00000539520	CBS
isotig28318	5.431	2.374	4.139	3.426	1026	0.5858101	isogroup13409	ENSG00000163795	ENST00000407879	ZNF513
isotig28320	5.314	4.694	3.689	1.876	1026	0.488229879	isogroup13411	ENSG00000089639	ENST00000445806	GMIP
isotig28322	7.358	4.063	7.18	4.364	1026	0.691515744	isogroup13413	ENSG00000100413	ENST00000407461	POLR3H
isotig28323	8.687	5.377	6.625	4.443	1023	0.628175021	isogroup13414	ENSG00000103037	ENST00000394266	SETD6
isotig28325	6.073	21.223	1.907	2.836	1026	0.933878786	isogroup13416	ENSG00000204291	ENST00000375001	COL15A1
isotig28326	12.853	13.365	13.957	9.182	1024	0.126399639	isogroup13417	ENSG00000063176	ENST00000340932	SPHK2
isotig28327	2.864	2.099	2.396	2.73	1025	0.09882017	isogroup13418	ENSG00000141499	ENST00000534050	WRAP53
isotig28331	37.565	28.041	51.81	53.313	1025	0.258416623	isogroup13422	ENSG00000105327	ENST00000449228	BBC3
isotig28332	2.189	2.105	1.68	1.139	1022	0.13859247	isogroup13423	ENSG00000198162	ENST00000356554	MAN1A2
isotig28335	1.209	1.766	2.092	1.945	1024	0.178778087	isogroup13426	ENSG00000240303	ENST00000264990	ACAD11
isotig28337	3.179	2.454	1.695	2.077	1024	0.190473059	isogroup13428	ENSG00000070061	ENST00000374647	IKBKAP
isotig28338	3.55	2.844	3.228	2.405	1024	0.379856467	isogroup13429	ENSG00000183248	ENST00000539422	AC010336.1
isotig28340	2.516	4.703	6.022	5.487	1024	0.20023296	isogroup13431	ENSG00000243147	ENST00000296102	MRPL33
isotig28342	1.874	2.363	3.375	3.326	1023	0.077224393	isogroup13433	ENSG00000107959	ENST00000451104	PITRM1
isotig28343	1.796	2.475	3.231	3.261	1023	0.091530773	isogroup13434	ENSG00000133731	ENST00000449740	IMPA1
isotig28344	1.776	1.135	1.949	1.838	1023	0.292186443	isogroup13435	ENSG00000125386	ENST00000545951	FAM193A
isotig28345	3.456	3.573	3.141	2.385	1023	0.173912227	isogroup13436	ENSG00000112312	ENST00000356509	GMNN
isotig28348	4.709	4.6	4.066	3.482	1022	0.231062599	isogroup13439	ENSG00000106348	ENST00000354269	IMPDH1
isotig28350	4.377	2.719	1.842	2.037	1022	0.300490343	isogroup13441	ENSG00000105321	ENST00000504556	CCDC9
isotig28353	1.408	2.293	1.573	1.754	1021	0.350041332	isogroup13444	ENSG00000115998	ENST00000420306	C2orf42
isotig28357	7.08	5.817	2.966	3.684	1021	0.120115353	isogroup13448	ENSG00000083312	ENST00000337273	TNPO1
isotig28362	3.364	5.388	2.062	2.39	1021	0.565191253	isogroup13453	ENSG00000196531	ENST00000454682	NACA
isotig28363	6.201	5.873	5.715	4.67	1021	0.176298189	isogroup13454	ENSG00000089597	ENST00000346178	GANAB
isotig28364	1.624	2.349	3.563	3.333	1021	0.193300519	isogroup13455	ENSG00000073578	ENST00000504309	SDHA
isotig28365	9.115	2.945	5.064	2.74	1021	0.84810626	isogroup13456	ENSG00000185730	ENST00000518575	ZNF696
isotig28367	8.372	5.929	8.518	8.025	1021	0.386366198	isogroup13458	ENSG00000012061	ENST00000300853	ERCC1
isotig28370	14.718	12.291	11.217	5.91	1019	0.649348087	isogroup13461	ENSG00000121210	ENST00000440693	KIAA0922
isotig28371	1.919	2.799	1.901	2.326	1020	0.406675058	isogroup13462	ENSG00000139508	ENST00000380814	SLC46A3
isotig28372	2.447	4.385	2.444	1.887	1020	0.336843391	isogroup13463	ENSG00000100065	ENST00000406271	CARD10
isotig28373	8.341	8.92	13.142	10.36	1020	0.123431277	isogroup13464	ENSG00000187566	ENST00000340650	NHLRC1
isotig28374	1.352	2.372	3.008	3.098	1019	0.221302698	isogroup13465	ENSG00000203786	ENST00000368773	KPRP
isotig28378	2.803	2.13	2.918	2.504	1019	0.236736304	isogroup13469	ENSG00000118900	ENST00000569442	UBN1
isotig28381	2.415	2.208	1.761	2.157	1017	0.041209514	isogroup13472	ENSG00000070061	ENST00000537196	IKBKAP
isotig28384	4.022	2.637	1.941	3.936	1018	0.021652138	isogroup13475	ENSG00000059728	ENST00000264444	MXD1
isotig28389	8.435	6.993	11.736	10.578	1018	0.13615954	isogroup13480	ENSG00000144040	ENST00000272433	SFXN5
isotig28390	8.592	3.179	5.877	3.453	1018	0.825834147	isogroup13481	ENSG00000161544	ENST00000293230	CYGB
isotig28391	3.296	3.684	1.614	2.683	1018	0.339201172	isogroup13482	ENSG00000198252	ENST00000442123	STYX
isotig28392	2.496	4.477	2.581	2.071	1016	0.300490343	isogroup13483	ENSG00000150630	ENST00000280193	VEGFC
isotig28394	2.474	3.145	4.485	4.139	1017	0.108561284	isogroup13485	ENSG00000166140	ENST00000355341	ZFYVE19
isotig28395	2.454	2.358	4.03	2.861	1017	0.321475539	isogroup13486	ENSG00000101190	ENST00000335351	TCFL5
isotig28396	1.846	3.07	3.305	4.182	1017	0.387221011	isogroup13487	ENSG00000128059	ENST00000264220	PPAT
isotig28397	1.198	1.928	3.141	3.147	1017	0.150381378	isogroup13488	ENSG00000122390	ENST00000414063	NAA60
isotig28398	1.847	2.136	1.817	1.873	1016	0.187795897	isogroup13489	ENSG00000198369	ENST00000452315	SPRED2
isotig28399	10.074	29.334	12.6	12.524	1009	0.896426693	isogroup13490	ENSG00000204839	ENST00000529971	C8orf73
isotig28400	2.439	7.707	1.783	3.705	1015	0.798386188	isogroup13491	ENSG00000069667	ENST00000449337	RORA
isotig28401	3.499	3.466	4.816	3.723	1016	0.308982115	isogroup13492	ENSG00000088320	ENST00000201979	REM1
isotig28404	1.376	2.342	2.926	2.6	1016	0.112769595	isogroup13495	ENSG00000074855	ENST00000159087	ANO8
isotig28409	1.318	2.363	2.929	3.154	1015	0.217714361	isogroup13500	ENSG00000182795	ENST00000359470	C1orf116
isotig28412	1.653	1.482	1.928	1.941	1015	0.078182536	isogroup13503	ENSG00000141577	ENST00000450824	AZI1
isotig28415	1.614	2.325	3.202	2.956	1015	0.081207259	isogroup13506	ENSG00000111605	ENST00000266679	CPSF6
isotig28417	1.536	2.722	3.478	2.98	1014	0.006660029	isogroup13508	ENSG00000155034	ENST00000297035	FBXL18
isotig28419	1.458	2.384	3.113	2.606	1014	0.06751146	isogroup13510	ENSG00000025708	ENST00000395678	TYMP
isotig28422	1.458	2.309	3.278	2.561	1014	0.025447133	isogroup13513	ENSG00000090621	ENST00000527718	PABPC4
isotig28423	38.519	43.679	48.381	45.903	1011	0.040317126	isogroup13514	ENSG00000185262	ENST00000327490	FAM100B
isotig28424	1.655	3.079	4.589	4.04	1014	0.166134365	isogroup13515	ENSG00000072778	ENST00000543245	ACADVL
isotig28427	5.633	3.285	3.588	3.599	1014	0.407999549	isogroup13518	ENSG00000177374	ENST00000399849	HIC1
isotig28429	2.258	1.926	1.495	1.304	1013	0.089576914	isogroup13520	ENSG00000137770	ENST00000558966	CTDSPL2
isotig28433	3.667	3.758	2.771	4.685	1013	0.301288795	isogroup13524	ENSG00000103187	ENST00000262428	COTL1
isotig28434	8.615	6.783	8.476	9.613	1013	0.119392049	isogroup13525	ENSG00000178188	ENST00000395532	SH2B1
isotig28436	3.509	4.472	3.172	2.564	1013	0.125638762	isogroup13527	ENSG00000167978	ENST00000544933	SRRM2
isotig28439	6.848	3.95	3.211	2.434	1012	0.516354174	isogroup13530	ENSG00000181588	ENST00000402693	MEX3D
isotig28440	2.171	1.975	1.241	2.389	1012	0.235092433	isogroup13531	ENSG00000167716	ENST00000409644	WDR81
isotig28441	4.085	2.219	2.5	2.588	1012	0.309113624	isogroup13532	ENSG00000083812	ENST00000536459	ZNF324
isotig28445	2.513	2.566	3.258	2.307	1009	0.313566168	isogroup13536	ENSG00000204262	ENST00000452536	COL5A2

isotig28446	3.951	2.786	2.119	1.882	1011	0.273427519	isogroup13537	ENSG00000102531	ENST00000541916	FNDC3A
isotig28447	1.133	2.256	2.629	2.564	1004	0.17921019	isogroup13538	ENSG00000183624	ENST00000509042	C3orf37
isotig28450	7.98	5.799	8.219	6.223	1011	0.553552641	isogroup13541	ENSG00000079313	ENST00000543452	REXO1
isotig28454	3.5	3.166	1.828	2.593	1010	0.115756745	isogroup13545	ENSG00000052795	ENST00000379346	FNIP2
isotig28455	3.895	3.957	2.816	3.015	1010	0.025447133	isogroup13546	ENSG00000096063	ENST00000423325	SRPK1
isotig28458	10.974	4.787	7.367	5.209	1010	0.803984745	isogroup13549	ENSG00000196497	ENST00000561462	IPO4
isotig28460	2.808	4.9	6.71	6.694	1010	0.182469753	isogroup13551	ENSG00000161996	ENST00000549091	WDR90
isotig28462	1.404	2.054	2.194	2.217	1010	0.022976629	isogroup13553	ENSG00000169876	ENST00000379439	MUC17
isotig28463	77.113	15.657	24.54	7.743	1009	0.978704817	isogroup13554	ENSG00000078814	ENST00000262873	MYH7B
isotig28464	1.286	1.924	2.397	2.374	1009	0.265903284	isogroup13555	ENSG00000141338	ENST00000541225	ABCA8
isotig28465	3.464	5.49	4.356	3.572	1009	0.254790712	isogroup13556	ENSG00000179604	ENST00000335793	CDC42EP4
isotig28468	4.671	1.358	3.496	1.442	1009	0.799983092	isogroup13559	ENSG00000182253	ENST00000560674	SYNM
isotig28469	2.87	1.396	2.397	1.575	1009	0.574622379	isogroup13560	ENSG00000068323	ENST00000315869	TFE3
isotig28471	2.383	2.517	3.964	2.826	1006	0.349214699	isogroup13562	ENSG00000186496	ENST00000399057	ZNF396
isotig28472	1.049	2.491	3.403	2.64	1009	0.140236342	isogroup13563	ENSG00000100429	ENST00000216271	HDAC10
isotig28473	1.386	2.547	2.653	2.907	1009	0.220325768	isogroup13564	ENSG00000133195	ENST00000542342	SLC39A11
isotig29088	2.853	2.519	3.685	2.352	952	0.094226723	isogroup14179	ENSG00000186340	ENST00000366787	THBS2
isotig29091	6.099	5.333	2.596	2.49	953	0.365709777	isogroup14182	ENSG00000121749	ENST00000550746	TBC1D15
isotig29092	3.406	2.412	1.594	1.249	951	0.300837905	isogroup14183	ENSG00000076108	ENST00000549884	BAZ2A
isotig29094	5.601	7.058	6.324	4.327	952	0.047550162	isogroup14185	ENSG00000145012	ENST00000543006	LPP
isotig29097	3.063	2.999	1.687	1.904	952	0.060757496	isogroup14188	ENSG00000163635	ENST00000538065	ATXN7
isotig29098	293.256	208.678	275.072	240.687	953	0.614883144	isogroup14189	ENSG00000125534	ENST00000370177	PPDPF
isotig29101	1.742	2.619	3.801	2.61	952	0.112901105	isogroup14192	ENSG00000084774	ENST00000403525	CAD
isotig29102	7.195	4.519	5.742	4.045	952	0.565294582	isogroup14193	ENSG00000168802	ENST00000306585	CHTF8
isotig29103	2.224	2.919	3.316	3.222	952	0.188754039	isogroup14194	ENSG00000186051	ENST00000334077	TAL2
isotig29104	1.344	2.462	2.913	3.343	951	0.263057038	isogroup14195	ENSG00000241123	ENST00000400372	KRTAP10-5
isotig29105	12.058	9.618	12.088	7.826	944	0.542440069	isogroup14196	ENSG00000177889	ENST00000318066	UBE2N
isotig29108	2.751	2.302	2.485	2.401	951	0.054116255	isogroup14199	ENSG00000176024	ENST00000391794	ZNF613
isotig29109	5.376	4.384	4	4.943	951	0.116282783	isogroup14200	ENSG00000126767	ENST00000376983	ELK1
isotig29110	1.176	1.841	2.232	1.671	951	0.021652138	isogroup14201	ENSG00000146966	ENST00000537639	DENND2A
isotig29111	1.365	2.601	3.165	3.295	951	0.174287969	isogroup14202	ENSG00000137747	ENST00000528626	TMPRSS13
isotig29113	1.091	1.821	2.136	2.001	951	0.114948899	isogroup14204	ENSG00000198173	ENST00000358047	FAM47C
isotig29114	31.101	55.442	27.319	28.907	951	0.697151875	isogroup14205	ENSG00000240583	ENST00000311813	AQP1
isotig29115	6.048	2.122	1.436	1.813	951	0.656393252	isogroup14206	ENSG00000111913	ENST00000259698	FAM65B
isotig29117	13.223	7.814	6.686	6.645	950	0.531280529	isogroup14208	ENSG00000121900	ENST00000373463	TMEM54
isotig29125	1.535	2.144	3.187	3.723	950	0.149996243	isogroup14216	ENSG00000187260	ENST00000477459	WDR86
isotig29126	7.084	6.272	6.822	4.666	950	0.317192079	isogroup14217	ENSG00000152022	ENST00000369308	LIX1L
isotig29127	3.447	2.524	3.848	2.804	950	0.361764485	isogroup14218	ENSG00000141013	ENST00000536122	GAS8
isotig29129	1.368	2.708	2.374	1.817	949	0.380241602	isogroup14220	ENSG00000196132	ENST00000536311	MYT1
isotig29130	4.924	6.298	6.673	7.572	949	0.362215375	isogroup14221	ENSG00000117983	ENST00000447027	MUC5B
isotig29131	4.651	5.135	2.023	1.817	949	0.119354475	isogroup14222	ENSG00000196642	ENST00000435930	C9orf86
isotig29132	5.633	4.4	7.264	5.152	943	0.493433907	isogroup14223	ENSG00000197932	ENST00000369446	F8A1
isotig29133	5.709	3.896	3.896	3.094	948	0.411362441	isogroup14224	ENSG00000111358	ENST00000543341	GTF2H3
isotig29135	2.189	1.965	1.945	1.769	949	0.106814083	isogroup14226	ENSG00000244462	ENST00000374114	RBM12
isotig29137	3.325	2.387	2.218	2.383	949	0.240146164	isogroup14228	ENSG00000096654	ENST00000377419	ZNF184
isotig29139	6.424	5.82	1.392	1.854	942	0.040580146	isogroup14230	ENSG00000138448	ENST00000261023	ITGAV
isotig29142	23.843	19.235	44.478	44.16	949	0.165138649	isogroup14233	ENSG00000132819	ENST00000440234	RBM38
isotig29148	3.224	2.992	3.039	2.574	948	0.110749981	isogroup14239	ENSG00000185619	ENST00000521023	PCGF3
isotig29150	1.2	2.59	2.669	2.526	948	0.242729391	isogroup14241	ENSG00000087842	ENST00000380421	PIR
isotig29155	3.585	3.96	2.048	3.073	947	0.254612234	isogroup14246	ENSG00000181555	ENST00000543224	SETD2
isotig29157	1.35	2.714	3.354	3.073	947	0.081207259	isogroup14248	ENSG00000171467	ENST00000361428	ZNF318
isotig29159	3.206	2.131	2.106	2.718	947	0.046479297	isogroup14250	ENSG00000161021	ENST00000292599	MAML1
isotig29160	1.202	3.015	3.08	2.931	947	0.362074472	isogroup14251	ENSG00000130487	ENST00000395676	KLHDC7B
isotig29166	8.276	5.634	8.608	6.129	946	0.618809649	isogroup14257	ENSG00000138136	ENST00000370193	LBX1
isotig29168	1.984	1.59	1.522	1.586	946	0.084682874	isogroup14259	ENSG00000104973	ENST00000542221	MED25
isotig29170	1.14	2.234	3.357	2.65	946	0.077975877	isogroup14261	ENSG00000177679	ENST00000388802	SRRM3
isotig29171	14.311	14.001	12.135	11.022	946	0.141354174	isogroup14262	ENSG00000124370	ENST00000244217	MCEE
isotig29172	2.941	2.113	2.857	3.106	937	0.128428647	isogroup14263	ENSG00000148985	ENST00000300730	PGAP2
isotig29173	2.428	1.971	1.581	1.846	946	0.083029608	isogroup14264	ENSG00000077713	ENST00000326714	SLC25A43
isotig29174	1.606	2.941	3.557	3.601	945	0.183136695	isogroup14265	ENSG00000103740	ENST00000560817	ACSBG1
isotig29176	1.078	1.953	2.384	2.63	945	0.258153603	isogroup14267	ENSG00000170801	ENST00000382512	HTRA3
isotig29178	1.31	2.659	2.892	2.701	945	0.125901781	isogroup14269	ENSG00000033050	ENST00000287844	ABCF2
isotig29182	1.965	3.888	5.237	4.999	945	0.256209138	isogroup14273	ENSG00000109705	ENST00000382438	NKX3-2
isotig29183	10.303	8.146	10.875	9.059	944	0.363351995	isogroup14274	ENSG00000163344	ENST00000368467	PMVK
isotig29184	1.543	2.236	3.673	3.624	945	0.081207259	isogroup14275	ENSG00000172009	ENST00000307741	THOP1
isotig29185	1.88	2.054	1.075	2.132	945	0.211129481	isogroup14276	ENSG00000169083	ENST00000544984	AR
isotig29187	2.012	1.474	1.958	1.709	943	0.20753175	isogroup14278	ENSG00000170949	ENST00000429604	ZNF160
isotig29190	1.417	3.004	3.325	3.13	944	0.29045803	isogroup14281	ENSG00000064932	ENST00000438103	SBNO2
isotig29191	3.241	3.331	3.368	2.469	943	0.113323815	isogroup14282	ENSG00000112320	ENST00000317357	SOBP
isotig29192	4.469	6.661	4.641	5.792	943	0.462228526	isogroup14283	ENSG00000177303	ENST00000433559	CASKIN2
isotig29195	3.177	2.402	3.133	2.896	943	0.21826858	isogroup14286	ENSG00000130783	ENST00000392441	CCDC62
isotig29199	13.702	16.531	8.185	7.336	943	0.370707147	isogroup14290	ENSG00000118137	ENST00000375323	APOA1
isotig29200	1.249	2.1	2.898	2.848	943	0.102201849	isogroup14291	ENSG00000203786	ENST00000368773	KPRP
isotig29204	4.235	3.209	3.211	2.944	943	0.259562636	isogroup14295	ENSG00000176293	ENST00000506786	ZNF135
isotig29205	1.61	2.583	3.231	2.848	943	0.197386714	isogroup14296	ENSG00000185737	ENST00000556918	NRG3
isotig29207	18.587	21.287	30.571	28.711	938	0.117729391	isogroup14298	ENSG00000112787	ENST00000434748	FBRSL1
isotig29208	2.12	4.122	4.92	4.064	942	0.094226723	isogroup14299	ENSG00000123297	ENST00000323833	TSFM
isotig29210	4.748	4.223	2.725	3.469	942	0.027992786	isogroup14301	ENSG00000171100	ENST00000543350	MTM1
isotig29215	40.328	38.314	43.494	37.319	931	0.212266101	isogroup14306	ENSG00000158042	ENST00000288937	MRPL17
isotig29216	3.625	1.98	1.882	1.45	942	0.531928684	isogroup14307	ENSG00000171560	ENST00000403106	FGA
isotig29217	5.046	2.688	4.939	1.093	942	0.806333133	isogroup14308	ENSG00000134363	ENST00000256759	FST
isotig29218	15.179	10.305	11.408	13.546	942	0.19906816	isogroup14309	ENSG00000115844	ENST00000234198	DLX2

isotig29219	1.187	1.96	1.783	2.115	942	0.264898174	isogroup14310	ENSG00000043514	ENST00000545233	TRIT1
isotig29222	4.993	5.164	3.85	3.024	940	0.101177951	isogroup14313	ENSG00000101040	ENST00000540497	ZMYND8
isotig29223	66.365	55.707	67.129	53.219	941	0.400916811	isogroup14314	ENSG00000130724	ENST00000312547	CHMP2A
isotig29225	1.103	2.165	2.944	2.426	941	0.06751146	isogroup14316	ENSG00000176381	ENST00000322583	PRR18
isotig29227	2.801	2.245	1.511	2.165	941	0.021652138	isogroup14318	ENSG00000112249	ENST00000369162	ASCC3
isotig29228	2.218	1.791	3.267	2.117	941	0.423311039	isogroup14319	ENSG00000171282	ENST00000307745	BAHCC1
isotig29232	24.476	13.83	21.234	21.601	940	0.585556474	isogroup14323	ENSG00000175602	ENST00000312579	CCDC85B
isotig29233	5.8	2.024	4.557	2.763	940	0.745162321	isogroup14324	ENSG00000187642	ENST00000433179	C1orf170
isotig29234	2.911	2.41	1.67	1.596	940	0.119720824	isogroup14325	ENSG00000160305	ENST00000466639	DIP2A
isotig29235	6.714	4.496	4.105	5.549	940	0.108533103	isogroup14326	ENSG00000248487	ENST00000273596	ABHD14A
isotig29239	4.611	3.544	3.987	4.43	940	0.204507026	isogroup14330	ENSG00000078699	ENST00000342704	CBFA2T2
isotig29240	3.356	5.63	1.926	2.429	940	0.548592846	isogroup14331	ENSG00000163539	ENST00000468888	CLASP2
isotig29241	2.167	4.86	2.514	6.883	940	0.820827384	isogroup14332	ENSG00000185950	ENST00000375856	IRS2
isotig29244	1.892	1.459	2.674	1.24	939	0.428890809	isogroup14335	ENSG00000198393	ENST00000534834	ZNF26
isotig29249	4.253	7.642	4.297	3.886	939	0.417787631	isogroup14340	ENSG00000104897	ENST00000221494	SF3A2
isotig29250	3.042	5.351	6.509	6.175	939	0.157961975	isogroup14341	ENSG00000143252	ENST00000367975	SDHC
isotig29252	4.424	5.656	2.537	2.789	939	0.51346096	isogroup14343	ENSG00000174705	ENST00000311601	SH3PXD2B
isotig29254	1.404	2.594	3.029	3.218	939	0.218071316	isogroup14345	ENSG00000126883	ENST00000438605	NUP214
isotig29256	3.339	3.061	4.012	3.505	939	0.283553769	isogroup14347	ENSG00000172534	ENST00000369984	HCFC1
isotig29257	2.34	3.872	5.171	5.222	939	0.251061471	isogroup14348	ENSG00000184470	ENST00000542719	TXNRD2
isotig29259	16.005	22.502	20.877	19.352	928	0.292881566	isogroup14350	ENSG00000167862	ENST00000301585	ICT1
isotig29260	16.756	18.324	41.506	24.738	938	0.646980912	isogroup14351	ENSG00000121904	ENST00000373381	CSMD2
isotig29262	3.683	2.151	2.697	1.671	938	0.546366574	isogroup14353	ENSG00000185614	ENST00000333323	FAM212A
isotig29269	1.044	1.4	2.028	2.004	938	0.054135042	isogroup14360	ENSG00000064687	ENST00000433129	ABCA7
isotig29270	4.301	4.445	6.339	4.487	938	0.319286842	isogroup14361	ENSG00000198589	ENST00000535741	LRBA
isotig29274	3.41	3.067	3.488	2.867	937	0.315163072	isogroup14365	ENSG00000221838	ENST00000429084	AP4M1
isotig29275	2.473	5.648	6.463	6.45	937	0.385924701	isogroup14366	ENSG00000074603	ENST00000559233	DPP8
isotig29277	1.449	2.397	3.685	3.68	937	0.115756745	isogroup14368	ENSG00000129282	ENST00000250156	MRM1
isotig29278	1.449	3.819	2.857	3.393	937	0.599684377	isogroup14369	ENSG00000073849	ENST00000448044	ST6GAL1
isotig29279	1.15	1.28	1.064	1.194	937	0.173752536	isogroup14370	ENSG00000188215	ENST00000563934	DCUN1D3
isotig29280	1.748	2.905	3.488	3.751	937	0.115756745	isogroup14371	ENSG00000228919	ENST00000429019	AC097381.1.1
isotig29281	1.899	2.257	3.137	2.248	936	0.043172766	isogroup14372	ENSG00000126562	ENST00000246914	WNK4
isotig29282	1.775	1.04	1.542	1.246	934	0.262540392	isogroup14373	ENSG00000145247	ENST00000508632	OCIAD2
isotig29283	2.454	4.23	4.261	2.296	936	0.139954535	isogroup14374	ENSG00000176125	ENST00000388761	UFSP1
isotig29284	2.645	4.311	4.557	3.947	936	0.24500263	isogroup14375	ENSG00000188153	ENST00000508186	COL4A5
isotig29288	1.387	1.993	2.387	2.248	936	0.208067183	isogroup14379	ENSG00000154358	ENST00000570156	OBSCN
isotig29290	2.675	2.57	1.148	1.968	933	0.111369956	isogroup14381	ENSG00000119041	ENST00000263956	GTF3C3
isotig29291	3.097	3.013	3.812	2.418	935	0.294299992	isogroup14382	ENSG00000101596	ENST00000320876	SMCHD1
isotig29293	1.114	1.981	2.972	2.618	932	0.050227324	isogroup14384	ENSG00000107957	ENST00000538130	SH3PXD2A
isotig29295	3.208	1.895	2.491	2.013	934	0.324218456	isogroup14386	ENSG00000168556	ENST00000302327	ING2
isotig29296	2.033	2.755	2.137	2.328	933	0.163504171	isogroup14387	ENSG00000158636	ENST00000533248	C11orf30
isotig29297	2.862	4.56	5.905	5.172	935	0.006660029	isogroup14388	ENSG00000099998	ENST00000418439	GGT5
isotig29299	30.566	35.334	44.371	40.695	933	0.165439243	isogroup14390	ENSG00000172216	ENST00000303004	CEBPB
isotig29300	4.207	2.749	3.338	4.214	935	0.206319982	isogroup14391	ENSG00000051009	ENST00000524416	FAM160A2
isotig29303	3.421	2.201	2.847	1.894	934	0.466859548	isogroup14394	ENSG00000153044	ENST00000283006	CENPH
isotig29304	6.5	1.549	5.872	1.821	934	0.883125423	isogroup14395	ENSG00000140931	ENST00000565922	CMTM3
isotig29307	1.604	1.895	2.431	1.223	934	0.143232885	isogroup14398	ENSG00000153814	ENST00000283928	JAZF1
isotig29308	7.898	6.937	8.055	7.175	933	0.239507402	isogroup14399	ENSG00000167978	ENST00000426305	SRRM2
isotig29314	1.988	3.158	3.183	3.427	934	0.142387465	isogroup14405	ENSG00000111640	ENST00000396858	GAPDH
isotig29319	13.892	6.222	12.527	10.222	933	0.686377471	isogroup14410	ENSG00000143862	ENST00000272217	ARL8A
isotig29321	1.263	2.244	3.384	3.264	933	0.157116555	isogroup14412	ENSG00000138029	ENST00000317799	HADHB
isotig29323	8.305	5.569	1.999	2.64	933	0.229512663	isogroup14414	ENSG00000136295	ENST00000403167	TTYH3
isotig29325	1.693	3.206	3.487	3.892	932	0.319324416	isogroup14416	ENSG00000156928	ENST00000466681	C7orf30
isotig29326	5.936	3.676	3.328	4.371	932	0.338449688	isogroup14417	ENSG00000168101	ENST00000304301	NUDT16L1
isotig29327	8.571	16.032	28.292	20.297	932	0.038757797	isogroup14418	ENSG00000212857	ENST00000391545	AL356585.1
isotig29329	1.114	2.206	3.031	2.835	932	0.233570677	isogroup14420	ENSG00000167978	ENST00000426305	SRRM2
isotig29331	3.3	2.859	2.813	2.835	932	0.035704892	isogroup14422	ENSG00000158615	ENST00000543650	PPP1R15B
isotig29333	2.408	2.111	1.47	1.157	929	0.149310513	isogroup14424	ENSG00000115355	ENST00000436346	CCDC88A
isotig29335	1.287	1.901	2.757	2.357	931	0.063566168	isogroup14426	ENSG00000204038	ENST00000356374	AL359195.1
isotig29336	6.671	4.13	4.859	3.655	931	0.57880251	isogroup14427	ENSG00000124151	ENST00000371998	NCOA3
isotig29339	1.545	2.147	2.777	3.415	931	0.112769595	isogroup14430	ENSG00000169184	ENST00000302326	MN1
isotig29340	2.102	2.412	1.607	2.116	931	0.280810851	isogroup14431	ENSG00000171634	ENST00000335221	BPTF
isotig29343	6.635	1.862	3.176	1.516	930	0.836627339	isogroup14434	ENSG00000213121	ENST00000392385	AL590867.1
isotig29346	1.889	2.129	2.362	3.082	930	0.272347261	isogroup14437	ENSG00000167114	ENST00000300456	SLC27A4
isotig29347	1.053	2.026	2.998	2.359	930	0.149488991	isogroup14438	ENSG00000002016	ENST00000539046	RAD52
isotig29349	1.718	3.398	4.646	4.526	930	0.182713985	isogroup14440	ENSG00000180340	ENST00000541149	FZD2
isotig29350	1.846	3.786	2.581	1.565	930	0.359650936	isogroup14441	ENSG00000148737	ENST00000534894	TCF7L2
isotig29353	2.233	4.483	1.529	1.709	930	0.508191178	isogroup14444	ENSG00000142279	ENST00000270288	WTIP
isotig29587	4.954	5.578	5.351	3.563	911	0.156008116	isogroup14678	ENSG00000005238	ENST00000378557	FAM214B
isotig29590	1.425	3.469	2.007	1.671	911	0.374210942	isogroup14681	ENSG00000150995	ENST00000423119	ITPR1
isotig29591	2.505	2.471	1.543	1.798	909	0.17677726	isogroup14682	ENSG00000100596	ENST00000216484	SPTLC2
isotig29593	4.1	2.925	3.709	3.563	911	0.329290975	isogroup14684	ENSG00000100335	ENST00000404569	SMCR7L
isotig29595	2.238	2.322	2.191	2.903	910	0.390659052	isogroup14686	ENSG00000119608	ENST00000556489	PROX2
isotig29596	1.076	1.862	2.922	2.558	910	0.080728188	isogroup14687	ENSG00000138685	ENST00000264498	FGF2
isotig29601	1.712	2.824	3.774	3.174	910	0.021652138	isogroup14692	ENSG00000131979	ENST00000491895	GCH1
isotig29610	1.23	2.241	2.153	3.004	909	0.344395807	isogroup14701	ENSG00000127586	ENST00000317063	CHTF18
isotig29615	4.153	3.497	1.686	2.118	909	0.014428496	isogroup14706	ENSG00000151694	ENST00000310823	ADAM17
isotig29616	3.123	2.494	3.64	1.972	908	0.319390171	isogroup14707	ENSG00000178498	ENST00000551632	DTX3
isotig29620	1.275	2.285	2.4	2.269	908	0.141429323	isogroup14711	ENSG00000140488	ENST00000437872	CELF6
isotig29621	5.806	3.627	4.759	4.586	908	0.472345382	isogroup14712	ENSG00000070404	ENST00000166139	FSTL3
isotig29625	1.65	3.669	2.806	3.599	908	0.53519764	isogroup14716	ENSG00000074855	ENST00000159087	ANO8
isotig29628	2.771	4.968	4.149	3.205	908	0.266457504	isogroup14719	ENSG00000065057	ENST00000219066	NTHL1

isotig29631	1.497	2.183	2.749	2.74	907	0.140236342	isogroup14722	ENSG00000162105	ENST00000425049	SHANK2
isotig29632	5.702	3.127	3.298	4.023	907	0.405735703	isogroup14723	ENSG00000198324	ENST00000450786	FAM109A
isotig29633	4.426	2.708	6.169	3.753	907	0.616451867	isogroup14724	ENSG00000124782	ENST00000379938	RREB1
isotig29634	2.73	1.721	3.278	2.196	907	0.466915909	isogroup14725	ENSG00000008735	ENST00000399912	MAPK8IP2
isotig29636	1.321	2.749	3.257	2.74	907	0.077975877	isogroup14727	ENSG00000128050	ENST00000512576	PAICS
isotig29638	108.574	115.066	144.889	172.948	907	0.405557226	isogroup14729	ENSG00000129250	ENST00000320785	KIF1C
isotig29641	1.036	1.786	1.549	2.15	906	0.246890734	isogroup14732	ENSG00000204060	ENST00000372591	FOXO6
isotig29642	4.012	1.744	2.263	2.643	906	0.505016157	isogroup14733	ENSG00000126464	ENST00000418929	PRR12
isotig29643	63.881	42.712	57.168	76.945	906	0.290946494	isogroup14734	ENSG00000196218	ENST00000359596	RYR1
isotig29644	1.83	3.256	3.994	4.151	906	0.161343654	isogroup14735	ENSG00000124313	ENST00000375368	IQSEC2
isotig29647	3.88	4.202	3.2	3.657	906	0.117494552	isogroup14738	ENSG00000254996	ENST00000532219	ANKHD1-EIF4EBP3
isotig29649	1.675	3.088	3.342	2.792	906	0.149996243	isogroup14740	ENSG00000148835	ENST00000369839	TAF5
isotig29650	1.389	1.597	2.731	2.348	906	0.069775306	isogroup14741	ENSG00000144488	ENST00000343063	ESPNL
isotig29651	5.907	4.558	1.773	3.459	906	0.185475689	isogroup14742	ENSG00000101966	ENST00000434753	XIAP
isotig29653	14.132	11.53	15.52	22.114	903	0.201613812	isogroup14744	ENSG00000228672	ENST00000434752	C5orf65
isotig29656	9.555	16.846	23.81	21.422	905	0.084992861	isogroup14747	ENSG00000165246	ENST00000382868	NLGN4Y
isotig29658	1.589	3.344	3.162	3.611	905	0.342460735	isogroup14749	ENSG00000177679	ENST00000388802	SRRM3
isotig29660	11.033	5.208	8.834	5.997	905	0.768542872	isogroup14751	ENSG00000075415	ENST00000551917	SLC25A3
isotig29662	340.878	1128.972	394.024	839.455	902	0.976065229	isogroup14753	ENSG00000103066	ENST00000568082	PLA2G15
isotig29666	4.136	6.472	5.848	5.33	903	0.319033216	isogroup14757	ENSG00000146909	ENST00000275820	NOM1
isotig29668	2.076	4.169	5.004	4.854	904	0.236942962	isogroup14759	ENSG00000255730	ENST00000540732	CTC-435M10.3.1
isotig29670	4.263	3.347	4.433	3.393	904	0.398230255	isogroup14761	ENSG00000120029	ENST00000370033	C10orf76
isotig29671	1.215	2.78	3.412	2.971	904	0.236773878	isogroup14762	ENSG00000111640	ENST00000396861	GAPDH
isotig29672	4.418	3.58	2.329	2.649	904	0.029937251	isogroup14763	ENSG00000105329	ENST00000221930	TGFB1
isotig29673	2.894	2.042	2.92	1.733	904	0.499755768	isogroup14764	ENSG00000166507	ENST00000309979	NDST2
isotig29674	3.207	1.813	3.15	2.107	903	0.582325092	isogroup14765	ENSG00000197483	ENST00000391718	ZNF628
isotig29675	3.667	2.19	2.492	1.684	904	0.390367852	isogroup14766	ENSG00000167695	ENST00000451373	FAM57A
isotig29677	1.237	1.494	1.062	1.04	904	0.029392425	isogroup14768	ENSG00000080802	ENST00000428680	CNOT4
isotig29678	74.879	51.418	71.408	67.21	904	0.4759619	isogroup14769	ENSG00000159692	ENST00000382952	CTBP1
isotig29680	2.223	2.008	2.594	4.399	904	0.275108965	isogroup14771	ENSG00000164309	ENST00000446378	CMYA5
isotig29683	4.733	5.227	3.435	2.107	903	0.101177951	isogroup14774	ENSG00000090661	ENST00000559450	CERS4
isotig29689	5.086	4.237	3.64	2.702	903	0.384055384	isogroup14780	ENSG00000059588	ENST00000040877	TARBP1
isotig29691	1.681	2.044	3.068	2.95	903	0.091530773	isogroup14782	ENSG00000106290	ENST00000472509	TAF6
isotig29692	2.831	2.023	1.819	1.661	903	0.264052754	isogroup14783	ENSG00000167258	ENST00000447079	CDK12
isotig29698	3.344	3.018	2.661	2.333	902	0.038353874	isogroup14789	ENSG00000052795	ENST00000379346	FNIP2
isotig29700	2.234	2.825	1.8	1.215	903	0.091699857	isogroup14791	ENSG00000147044	ENST00000421587	CASK
isotig29701	1.862	1.986	2.767	1.715	901	0.229437514	isogroup14792	ENSG00000140443	ENST00000558762	IGF1R
isotig29702	16.799	14.286	19.539	15.3	894	0.429905313	isogroup14793	ENSG00000236826	ENST00000455705	LSM2
isotig29703	2.059	3.271	4.667	3.797	902	0.072142481	isogroup14794	ENSG00000180438	ENST00000429201	TPRXL
isotig29704	5.558	3.882	6.448	4.12	902	0.622604644	isogroup14795	ENSG00000130940	ENST00000377022	CASZ1
isotig29706	1.816	2.004	3.705	2.854	902	0.282032013	isogroup14797	ENSG00000125835	ENST00000303103	SNRPB
isotig29708	2.28	3.735	4.667	4.269	902	0.038353874	isogroup14799	ENSG00000204572	ENST00000376536	KRTAP5-10
isotig29712	1.241	2.154	2.131	3.007	901	0.362638085	isogroup14803	ENSG00000170439	ENST00000394252	METTL7B
isotig29714	3.214	3.423	2.685	1.64	901	0.099938003	isogroup14805	ENSG00000169744	ENST00000515064	LDB2
isotig29715	1.507	1.373	1.271	1.342	901	0.147779364	isogroup14806	ENSG00000070366	ENST00000544865	SMG6
isotig29717	2.001	2.161	1.768	1.695	898	0.076266251	isogroup14808	ENSG00000198939	ENST00000523286	ZFP2
isotig29719	2.35	4.162	6.271	5.168	901	0.029937251	isogroup14810	ENSG00000189058	ENST00000421243	APOD
isotig29720	3.772	2.221	2.79	1.841	900	0.457306305	isogroup14811	ENSG00000164049	ENST00000458736	FBXW12
isotig29722	1.441	1.246	2.397	2.162	901	0.26946344	isogroup14813	ENSG00000126003	ENST00000246229	PLAGL2
isotig29727	28.581	20.706	34.96	28.68	900	0.528997896	isogroup14818	ENSG00000007080	ENST00000445755	CCDC124
isotig29729	1.687	2.728	3.406	4.004	900	0.270910047	isogroup14820	ENSG00000063854	ENST00000455446	HAGH
isotig29731	1.509	2.136	2.852	2.661	900	0.06751146	isogroup14822	ENSG00000111077	ENST00000546602	TENC1
isotig29733	4.46	3.341	3.878	3.657	900	0.288879913	isogroup14824	ENSG00000113360	ENST00000507438	DROSHA
isotig29736	1.155	1.673	1.191	1.27	899	0.252883821	isogroup14827	ENSG00000174718	ENST00000312561	C12orf35
isotig29737	4.172	1.417	2.503	2.687	900	0.536775757	isogroup14828	ENSG00000143603	ENST00000271915	KCNN3
isotig29738	1.043	2.221	2.729	2.04	900	0.041209514	isogroup14829	ENSG00000118160	ENST00000391903	SLC8A2
isotig29739	1.242	2.306	2.77	2.836	900	0.187185316	isogroup14830	ENSG00000232367	ENST00000549225	TAP1
isotig29740	5.71	2.838	2.239	2.74	899	0.43965582	isogroup14831	ENSG00000127124	ENST00000429157	HIVEP3
isotig29741	3.954	3.409	4.314	3.188	899	0.326942587	isogroup14832	ENSG00000167548	ENST00000301067	MLL2
isotig29748	1.733	2.986	3.533	3.536	899	0.122942812	isogroup14839	ENSG00000139219	ENST00000395281	COL2A1
isotig29749	4.11	1.631	3.512	1.693	899	0.713130307	isogroup14840	ENSG00000134030	ENST00000382998	CTIF
isotig29752	1.555	2.562	3.101	2.44	899	0.021652138	isogroup14843	ENSG00000143520	ENST00000388718	FLG2
isotig29755	59.001	86.756	75.524	72.745	898	0.291434959	isogroup14846	ENSG00000171812	ENST00000481785	COL8A2
isotig29756	1.644	2.372	2.732	3.337	899	0.263179154	isogroup14847	ENSG00000180438	ENST00000429201	TPRXL
isotig29760	1.49	2.48	3.063	4.013	898	0.46382543	isogroup14851	ENSG00000166689	ENST00000530489	PLEKHA7
isotig29761	1.601	2.48	3.085	3.116	898	0.230771399	isogroup14852	ENSG00000127152	ENST00000357195	BCL11B
isotig29762	1.135	1.886	2.837	2.542	898	0.069775306	isogroup14853	ENSG00000061273	ENST00000430670	HDAC7
isotig29763	1.157	1.061	1.832	2.595	897	0.060513264	isogroup14854	ENSG00000115956	ENST00000234313	PLEK
isotig29766	3.923	3.463	2.081	2.273	896	0.242062448	isogroup14857	ENSG00000173273	ENST00000518281	TNKS
isotig29767	1.401	2.543	2.591	2.268	898	0.203548884	isogroup14858	ENSG00000146232	ENST00000477930	NFKBIE
isotig29770	2.377	2.266	1.746	1.444	899	0.039415345	isogroup14861	ENSG00000082269	ENST00000505769	FAM135A
isotig29771	9.974	11.441	11.306	11.556	895	0.095560607	isogroup14862	ENSG00000205858	ENST00000401542	LRRC72
isotig29772	2.202	2.013	2.056	2.119	898	0.06751146	isogroup14863	ENSG00000163832	ENST00000296149	C3orf75
isotig29773	2.669	4.727	3.063	3.665	898	0.634121139	isogroup14864	ENSG00000074855	ENST00000159087	ANO8
isotig29775	3.905	5.955	4.105	3.777	895	0.439580672	isogroup14866	ENSG00000216490	ENST00000407280	IFI30
isotig29778	3.941	1.719	3.499	2.297	897	0.612168408	isogroup14869	ENSG00000131400	ENST00000253719	NAPSA
isotig29780	1.692	2.037	1.564	1.074	897	0.141805065	isogroup14871	ENSG00000198455	ENST00000374888	ZXDB
isotig29782	1.804	2.313	3.499	3.494	897	0.017143233	isogroup14873	ENSG00000186143	ENST00000335524	C2orf53
isotig29783	10.197	3.629	6.279	4.243	897	0.850482829	isogroup14874	ENSG00000127080	ENST00000287996	IPPK
isotig29788	6.612	1.337	5.455	2.071	897	0.875760878	isogroup14879	ENSG00000162650	ENST00000369870	ATXN7L2
isotig29790	4.257	4.079	3.215	3.023	896	0.064082814	isogroup14881	ENSG00000131626	ENST00000544950	PPFIA1
isotig29792	4.044	3.513	2.767	2.729	894	0.038353874	isogroup14883	ENSG00000143776	ENST00000366767	CDC42BPA

isotig29793	1.605	2.145	1.195	1.4	896	0.239883144	isogroup14884	ENSG00000198920	ENST00000542606	KIAA0753
isotig29794	1.916	1.317	1.979	2.074	896	0.063566168	isogroup14885	ENSG00000068654	ENST00000409681	POLR1A
isotig29797	5.037	3.038	3.318	3.173	896	0.409577666	isogroup14888	ENSG00000066422	ENST00000461821	ZBTB11
isotig29799	1.339	2.297	3.673	3.602	895	0.071109191	isogroup14890	ENSG00000238227	ENST00000561457	C9orf69
isotig29803	7.922	3.041	7.489	4.752	895	0.803139325	isogroup14894	ENSG00000105516	ENST00000222122	DBP
isotig29804	1.918	2.764	3.012	3.402	895	0.314430375	isogroup14895	ENSG00000224940	ENST00000480290	PRRT4
isotig29805	3.016	2.512	6.134	4.157	894	0.354230856	isogroup14896	ENSG00000163704	ENST00000411976	PRRT3
isotig29813	7.416	7.111	4.689	3.004	894	0.454535207	isogroup14904	ENSG00000221968	ENST00000525588	FADS3
isotig29814	1.407	1.682	1.364	1.252	894	0.159737356	isogroup14905	ENSG00000181240	ENST00000458275	SLC25A41
isotig29815	2.122	2.279	1.384	2.604	894	0.316515744	isogroup14906	ENSG00000129038	ENST00000566011	LOXL1
isotig29816	1.362	2.022	2.54	2.529	894	0.05275419	isogroup14907	ENSG00000117983	ENST00000529681	MUC5B
isotig29817	24.015	9.879	10.513	5.034	894	0.943328699	isogroup14908	ENSG00000204262	ENST00000374866	COL5A2
isotig29821	4.512	4.301	2.726	3.004	894	0.051664537	isogroup14912	ENSG00000141579	ENST00000269394	ZNF750
isotig29822	1.161	2.534	3.655	3.831	894	0.129912828	isogroup14913	ENSG00000228760	ENST00000550393	BAG6
isotig29824	5.942	15.99	1.673	2.78	894	0.812410761	isogroup14915	ENSG00000119927	ENST00000423155	GPAM
isotig29827	2.572	4.518	5.066	5.264	893	0.204507026	isogroup14918	ENSG00000133115	ENST00000423210	STOML3
isotig29828	2.595	4.903	2.316	1.981	893	0.513827309	isogroup14919	ENSG00000141449	ENST00000431264	GREB1L
isotig29829	1.409	2.749	3.205	2.432	893	0.003503795	isogroup14920	ENSG00000074416	ENST00000536024	MGLL
isotig29831	2.057	2.515	2.191	1.88	893	0.014428496	isogroup14922	ENSG00000151779	ENST00000281513	NBAS
isotig29835	5.099	3.773	3.577	3.209	893	0.408938904	isogroup14926	ENSG00000163788	ENST00000454177	SNRK
isotig29839	4.747	5.399	5.465	4.919	892	0.133529345	isogroup14930	ENSG00000159840	ENST00000354434	ZYX
isotig29841	1.41	6.039	3.312	1.254	892	0.67635455	isogroup14932	ENSG00000175183	ENST00000552330	CSRP2
isotig29845	1.164	2.048	2.36	2.836	892	0.176880589	isogroup14936	ENSG00000117983	ENST00000538459	MUC5B
isotig29847	1.12	2.582	2.919	2.786	892	0.199706921	isogroup14938	ENSG00000139219	ENST00000395281	COL2A1
isotig29851	1.142	1.984	2.567	2.008	892	0.021652138	isogroup14942	ENSG00000184956	ENST00000421673	MUC6
isotig29852	4.465	2.438	3.818	3.848	890	0.431577365	isogroup14943	ENSG00000213015	ENST00000545125	ZNF580
isotig29854	2.645	1.624	1.824	1.582	891	0.332907492	isogroup14945	ENSG00000130589	ENST00000467148	RP4-697K14.7.1
isotig29855	1.927	1.731	1.907	1.407	891	0.155397535	isogroup14946	ENSG00000243335	ENST00000275532	KCTD7
isotig29856	1.367	2.093	2.798	2.764	891	0.125901781	isogroup14947	ENSG00000167978	ENST00000544933	SRRM2
isotig29859	1.524	2.457	3.026	2.99	891	0.170915683	isogroup14950	ENSG00000238227	ENST00000561457	C9orf69
isotig29860	1.664	1.886	1.518	1.891	888	0.338994514	isogroup14951	ENSG00000241241	ENST00000440582	AC100808.7.1
isotig29862	4.976	5.166	6.08	5.205	899	0.108533103	isogroup14953	ENSG00000123179	ENST00000242827	EBPL
isotig29864	7.351	1.239	4.663	2.061	891	0.868734501	isogroup14955	ENSG00000130701	ENST00000252998	C20orf151
isotig29865	1.145	2.01	2.137	2.163	890	0.213590591	isogroup14956	ENSG00000182601	ENST00000331351	HS3ST4
isotig29866	4.084	1.754	3.216	2.315	890	0.631143383	isogroup14957	ENSG00000105663	ENST00000420124	MLL4.1
isotig29870	1.301	2.694	3.091	2.792	890	0.174006162	isogroup14961	ENSG00000188766	ENST00000338502	SPRED3
isotig29871	5.745	1.369	3.382	3.798	890	0.741310964	isogroup14962	ENSG00000203852	ENST00000403683	HIST2H3A
isotig29872	4.465	4.77	3.921	3.798	890	0.367588487	isogroup14963	ENSG00000079819	ENST00000528282	EPB41L2
isotig29874	2.404	2.249	1.766	1.838	889	0.05275419	isogroup14965	ENSG00000182511	ENST00000450438	FES
isotig30087	1.142	1.719	1.393	1.611	875	0.376061471	isogroup15178	ENSG00000205277	ENST00000536621	MUC12
isotig30089	2.099	1.784	1.794	1.766	875	0.178778087	isogroup15180	ENSG00000143995	ENST00000407092	MEIS1
isotig30090	1.05	2.241	2.343	1.995	875	0.149310513	isogroup15181	ENSG00000113318	ENST00000535995	MSH3
isotig30091	3.789	2.001	2.511	2.763	875	0.4379368	isogroup15182	ENSG00000189114	ENST00000433642	BLOC1S3
isotig30092	2.054	1.806	1.603	1.202	875	0.233457954	isogroup15183	ENSG00000116747	ENST00000416058	TROVE2
isotig30588	3.196	1.588	1.743	3.678	842	0.106053205	isogroup15679	ENSG00000130303	ENST00000416178	BST2
isotig30592	4.725	1.788	4.918	2.422	841	0.719996994	isogroup15683	ENSG00000129152	ENST00000250003	MYOD1
isotig30594	1.377	2.512	3.6	2.795	841	0.003503795	isogroup15685	ENSG00000100116	ENST00000323205	GCAT
isotig30595	14.272	13.127	18.706	21.189	841	0.020365221	isogroup15686	ENSG00000108509	ENST00000414043	CAMTA2
isotig30596	4.061	4.753	2.568	2.848	841	0.272243932	isogroup15687	ENSG00000103335	ENST00000301015	PIEZO1
isotig30603	2.425	2.651	1.802	2.851	840	0.340769896	isogroup15694	ENSG00000197070	ENST00000371421	ARRDC1
isotig30607	2.404	2.223	1.321	1.2	839	0.010060494	isogroup15698	ENSG00000068796	ENST00000401507	KIF2A
isotig30609	11.93	9.21	15.024	10.137	837	0.52102277	isogroup15700	ENSG00000101624	ENST00000423709	CEP76
isotig30610	18.23	7.925	12.007	5.357	840	0.902081611	isogroup15701	ENSG00000092841	ENST00000553056	MYL6
isotig30611	3.118	2.586	1.497	1.361	839	0.286531525	isogroup15702	ENSG00000196843	ENST00000359765	ARID5A
isotig30612	7.212	5.989	6.955	5.523	839	0.460584655	isogroup15703	ENSG00000135686	ENST00000564996	KLHL36
isotig30613	1.499	1.248	1.584	1.12	839	0.105508379	isogroup15704	ENSG00000102312	ENST00000537758	PORCN
isotig30614	7.959	6.7	3.019	2.484	838	0.4530886	isogroup15705	ENSG00000005238	ENST00000378557	FAM214B
isotig30615	4.309	2.496	4.578	1.788	839	0.758407229	isogroup15706	ENSG00000136205	ENST00000545849	TNS3
isotig30616	2.094	4.174	5.546	4.963	839	0.111839633	isogroup15707	ENSG00000158805	ENST00000568064	ZNF276
isotig30619	7.164	3.471	3.114	2.628	839	0.58107575	isogroup15710	ENSG00000088280	ENST00000485644	ASAP3
isotig30621	2.547	1.089	2.025	1.174	839	0.486059968	isogroup15712	ENSG00000110237	ENST00000263674	ARHGEF17
isotig30623	4.213	5.331	3.346	2.936	839	0.20295709	isogroup15714	ENSG00000168067	ENST00000377350	MAP4K2
isotig30624	1.881	1.657	1.518	1.334	839	0.078182536	isogroup15715	ENSG00000103550	ENST00000219837	C16orf88
isotig30632	12.24	6.026	11.648	3.932	837	0.903415496	isogroup15723	ENSG00000184675	ENST00000330258	FAM123B
isotig30633	1.286	2.952	4.011	3.339	838	0.132946945	isogroup15724	ENSG00000186834	ENST00000332499	HEXIM1
isotig30636	1.001	2.34	2.314	2.939	838	0.319023822	isogroup15727	ENSG00000155640	ENST00000286067	C10orf12
isotig30638	9.295	8.245	8.572	9.377	838	0.020365221	isogroup15729	ENSG00000196670	ENST00000502412	ZFP62
isotig30641	6.346	4.367	8.603	5.51	837	0.60754678	isogroup15732	ENSG00000122145	ENST00000442340	TBX22
isotig30643	1.122	1.82	2.205	2.514	837	0.197095514	isogroup15734	ENSG00000149485	ENST00000350997	FADS1
isotig30645	6.657	5.344	4.346	4.2	837	0.233382806	isogroup15736	ENSG00000124333	ENST00000286448	VAMP7
isotig30646	25.53	29.404	36.002	40.708	837	0.240916435	isogroup15737	ENSG00000100321	ENST00000318801	SYNGR1
isotig30648	1.438	2.582	3.169	2.688	833	0.18420756	isogroup15739	ENSG00000143520	ENST00000388718	FLG2
isotig30649	1.312	1.637	2.25	1.792	837	0.022976629	isogroup15740	ENSG00000124588	ENST00000380430	NQO2
isotig30653	4.08	1.342	3.706	3.103	837	0.61598219	isogroup15744	ENSG00000126464	ENST00000418929	PRR12
isotig30654	1.551	1.933	2.317	2.407	837	0.098416247	isogroup15745	ENSG00000183250	ENST00000330551	C21orf67
isotig30658	7.717	6.535	12.656	8.141	836	0.571776133	isogroup15749	ENSG00000142279	ENST00000270288	WTIP
isotig30659	2.867	2.914	1.281	1.58	836	0.115681596	isogroup15750	ENSG00000118922	ENST00000377669	KLF12
isotig30663	2.571	1.186	2.675	1.662	835	0.584194409	isogroup15754	ENSG00000173166	ENST00000374493	RAPH1
isotig30664	1.337	2.14	2.562	3.106	836	0.242729391	isogroup15755	ENSG00000142544	ENST00000421832	CTU1
isotig30665	20.807	14.594	38.078	26.993	836	0.639954535	isogroup15756	ENSG00000132329	ENST00000254661	RAMP1
isotig30666	1.219	2.414	3.004	2.758	836	0.112769595	isogroup15757	ENSG00000177303	ENST00000433559	CASKIN2
isotig30673	15.122	16.006	23.236	26.913	836	0.191327873	isogroup15764	ENSG00000124104	ENST00000491381	SNX21

isotig30674	1.433	3.324	3.733	3.455	836	0.237929285	isogroup15765	ENSG00000215454	ENST00000400374	KRTAP10-4
isotig30678	1.22	2.325	3.45	2.493	835	0.050227324	isogroup15769	ENSG00000117362	ENST00000369109	APH1A
isotig30679	3.185	4.77	3.055	2.819	834	0.533619524	isogroup15770	ENSG00000066735	ENST00000423312	KIF26A
isotig30684	2.941	2.553	2.853	2.119	835	0.275108965	isogroup15775	ENSG00000221838	ENST00000421755	AP4M1
isotig30685	4.759	3.601	2.675	2.091	835	0.257862403	isogroup15776	ENSG00000005108	ENST00000423059	THSD7A
isotig30688	2.583	2.667	2.234	2.305	835	0.060757496	isogroup15779	ENSG00000078687	ENST00000544502	TNRC6C
isotig30689	3.169	2.453	3.35	2.511	838	0.415354701	isogroup15780	ENSG00000131591	ENST00000448924	C1orf159
isotig30690	1.459	1.322	1.393	1.368	835	0.054895919	isogroup15781	ENSG00000185219	ENST00000425708	ZNF445
isotig30691	5.293	2.259	3.941	3.651	834	0.586138874	isogroup15782	ENSG00000119682	ENST00000356357	KIAA0317
isotig30692	2.945	1.894	2.701	2.872	834	0.160639137	isogroup15783	ENSG00000149115	ENST00000532437	TNKS1BP1
isotig30693	10.629	8.45	7.382	7.106	838	0.377930788	isogroup15784	ENSG00000172301	ENST00000302362	C17orf79
isotig30694	1.436	2.1	2.59	2.819	834	0.139334561	isogroup15785	ENSG00000238035	ENST00000456544	AC138035.1
isotig30695	2.754	2.556	3.366	2.631	834	0.215525663	isogroup15786	ENSG00000214014	ENST00000263084	OVCA2
isotig30696	26.963	30.879	33.565	34.788	834	0.103376043	isogroup15787	ENSG00000152082	ENST00000409255	MZT2B
isotig30697	22.534	9.928	6.842	6.577	834	0.833499286	isogroup15788	ENSG00000141150	ENST00000268864	RASL10B
isotig30699	2.107	1.643	2.014	1.53	834	0.271783648	isogroup15790	ENSG00000156860	ENST00000356166	FBRS
isotig30700	5.423	5.146	5.158	3.355	834	0.241677313	isogroup15791	ENSG00000100994	ENST00000216962	PYGB
isotig30705	2.277	1.691	2.881	2.553	833	0.298583452	isogroup15796	ENSG00000177352	ENST00000321895	CCDC71
isotig30708	4.71	3.116	3.933	1.509	831	0.585274667	isogroup15799	ENSG00000003096	ENST00000545703	KLHL13
isotig30711	2.948	2.788	1.131	1.291	833	0.132824829	isogroup15802	ENSG00000073614	ENST00000399788	KDM5A
isotig30718	8.546	2.15	5.66	3.417	832	0.875648155	isogroup15809	ENSG00000204176	ENST00000374323	SYT15
isotig30724	3.072	5.285	5.703	4.332	832	0.17548095	isogroup15815	ENSG00000135835	ENST00000367588	KIAA1614
isotig30725	1.638	1.723	1.381	1.485	829	0.170136019	isogroup15816	ENSG00000100842	ENST00000216733	EFS
isotig30727	1.177	2.471	2.907	2.933	832	0.224045615	isogroup15818	ENSG00000136010	ENST00000258494	ALDH1L2
isotig30728	3.216	6.2	3.707	5.005	832	0.583452318	isogroup15819	ENSG00000148396	ENST00000290037	SEC16A
isotig30731	1.344	2.264	2.929	3.121	832	0.157041407	isogroup15822	ENSG00000116141	ENST00000402574	MARK1
isotig30732	2.527	13.875	1.98	1.834	830	0.887465244	isogroup15823	ENSG00000162341	ENST00000356782	TPCN2
isotig30733	1.082	2.68	2.578	2.747	831	0.314712182	isogroup15824	ENSG00000187626	ENST00000377294	ZKSCAN4
isotig30735	1.586	2.016	3.733	2.801	831	0.174287969	isogroup15826	ENSG00000173540	ENST00000480687	GMPPB
isotig30736	1.59	2.25	2.005	1.566	829	0.277006463	isogroup15827	ENSG00000176809	ENST00000400877	LRRC37A3
isotig30742	2.358	3.486	1.713	2.428	830	0.592996167	isogroup15833	ENSG00000063169	ENST00000396720	GLTSCR1
isotig30744	1.684	2.729	2.981	2.724	830	0.157116555	isogroup15835	ENSG00000133226	ENST00000447431	SRRM1
isotig30751	1.588	3.028	2.936	3.236	830	0.359143684	isogroup15842	ENSG00000223496	ENST00000435634	EXOSC6
isotig30753	2.43	1.698	1.646	1.861	830	0.045840535	isogroup15844	ENSG00000241839	ENST00000323544	PLEKHO2
isotig30754	1.516	1.881	2.313	2.967	830	0.254724957	isogroup15845	ENSG00000198788	ENST00000441003	MUC2
isotig30755	1.275	1.467	1.78	2.347	830	0.204244007	isogroup15846	ENSG00000130193	ENST00000520217	C8orf55
isotig30760	2.071	1.7	1.805	1.458	829	0.184818141	isogroup15851	ENSG00000068654	ENST00000409681	POLR1A
isotig30763	1.036	1.814	2.339	2.052	829	0.065266401	isogroup15854	ENSG00000169436	ENST00000545577	COL22A1
isotig30766	1.831	2.824	3.497	3.375	829	0.091530773	isogroup15857	ENSG00000091986	ENST00000439685	CCDC80
isotig30768	1.952	1.791	2.45	2.485	829	0.087435184	isogroup15859	ENSG00000028137	ENST00000376259	TNFRSF1B
isotig30770	2.189	2.935	2.247	1.646	830	0.250385136	isogroup15861	ENSG00000138175	ENST00000260746	ARL3
isotig30771	1.517	3.054	3.542	3.349	829	0.112901105	isogroup15862	ENSG00000171282	ENST00000307745	BAHCC1
isotig30776	1.374	2.621	2.921	2.353	828	0.095447885	isogroup15867	ENSG00000157637	ENST00000288439	SLC38A10
isotig30777	1.668	2.972	2.839	1.654	826	0.126700233	isogroup15868	ENSG00000109113	ENST00000447716	RAB34
isotig30779	2.484	7.471	5.063	4.217	828	0.588233636	isogroup15870	ENSG00000205726	ENST00000440794	ITSN1
isotig30781	2.318	1.611	1.764	1.759	827	0.271783648	isogroup15872	ENSG00000146857	ENST00000275764	STRA8
isotig30788	1.642	2.532	3.104	2.68	827	0.081207259	isogroup15879	ENSG00000101004	ENST00000278886	NINL
isotig30790	10.625	10.058	9.556	9.421	827	0.135811979	isogroup15881	ENSG00000198858	ENST00000361574	R3HDM4
isotig30797	41.306	51.044	48.841	47.722	820	0.135201398	isogroup15888	ENSG00000174547	ENST00000310999	MRPL11
isotig30801	1.376	3.222	2.97	3.005	827	0.380175847	isogroup15892	ENSG00000099822	ENST00000251287	HCN2
isotig30803	1.644	2.581	3.331	3.523	826	0.102323965	isogroup15894	ENSG00000129749	ENST00000534359	CHRNA10
isotig30805	1.62	2.42	2.638	2.873	826	0.084617119	isogroup15896	ENSG00000132837	ENST00000255189	DMGDH
isotig30809	4.134	1.567	3.153	2.575	826	0.572076727	isogroup15900	ENSG00000161682	ENST00000293443	FAM171A2
isotig30811	1.571	1.844	2.482	2.033	826	0.094264297	isogroup15902	ENSG00000162390	ENST00000371316	ACOT11
isotig30814	60.686	53.992	86.266	64.505	826	0.497980386	isogroup15905	ENSG00000136270	ENST00000494076	TBRG4
isotig30817	2.902	2.143	2.705	2.521	826	0.275813482	isogroup15908	ENSG00000158615	ENST00000367188	PPP1R15B
isotig30818	1.378	2.719	3.644	2.9	826	0.010060494	isogroup15909	ENSG00000171174	ENST00000302188	RBKS
isotig30819	8.68	5.738	5.544	4.174	826	0.589304501	isogroup15910	ENSG00000147789	ENST00000446747	ZNF7
isotig30821	90.557	72.73	95.45	78.34	827	0.454394304	isogroup15912	ENSG00000130985	ENST00000377269	UBA1
isotig30823	1.622	2.469	3.29	3.744	825	0.180356204	isogroup15914	ENSG00000164692	ENST00000545487	COL1A2
isotig30825	4.962	4.96	5.215	3.799	825	0.261760727	isogroup15916	ENSG00000196689	ENST00000174621	TRPV1
isotig30834	3.511	5.557	2.392	4.221	822	0.586138874	isogroup15925	ENSG00000119711	ENST00000553458	ALDH6A1
isotig30835	6.851	7.521	5.349	7.028	825	0.2820508	isogroup15926	ENSG00000099849	ENST00000454668	RASSF7
isotig30836	2.034	2.953	4.074	3.175	825	0.077224393	isogroup15927	ENSG00000254997	ENST00000528743	KRTAP5-9
isotig30842	1.454	1.917	2.621	2.038	824	0.034718569	isogroup15933	ENSG00000154265	ENST00000392676	ABCA5
isotig30846	2.932	4.943	3.921	3.994	824	0.511610431	isogroup15937	ENSG00000227500	ENST00000316097	SCAMP4
isotig30847	1.091	2.125	2.24	2.2	824	0.266542046	isogroup15938	ENSG00000206252	ENST00000383146	AP000525.1
isotig30848	214.029	163.741	185.368	154.578	820	0.406346284	isogroup15939	ENSG00000130528	ENST00000252825	HRC
isotig30849	2.108	2.263	1.771	2.417	824	0.267781994	isogroup15940	ENSG00000048991	ENST00000409606	R3HDM1
isotig30850	7.513	3.881	5.422	3.45	824	0.648220861	isogroup15941	ENSG00000205047	ENST00000446344	AC010536.1
isotig30851	2.981	2.449	2.465	2.011	824	0.180356204	isogroup15942	ENSG00000132781	ENST00000450313	MUTYH
isotig30852	4.46	2.541	3.002	2.064	824	0.571297062	isogroup15943	ENSG00000164116	ENST00000506455	GUCY1A3
isotig30853	8.159	3.738	4.616	3.385	820	0.651198617	isogroup15944	ENSG00000232098	ENST00000444227	AC012313.1
isotig30854	6.398	2.171	4.751	2.663	824	0.764513038	isogroup15945	ENSG00000171992	ENST00000307662	SYNPO
isotig30855	3.563	2.38	1.479	2.853	824	0.043172766	isogroup15946	ENSG00000119682	ENST00000556202	KIAA0317
isotig30857	2.012	1.34	1.569	1.684	824	0.127789885	isogroup15948	ENSG00000168056	ENST00000530866	LTBP3
isotig30858	2.763	1.802	1.792	1.169	824	0.408619524	isogroup15949	ENSG00000063169	ENST00000396720	GLTSCR1
isotig30860	3.199	2.194	2.824	2.364	824	0.284126775	isogroup15951	ENSG00000141252	ENST00000437048	VPS53
isotig31087	1.382	2.471	2.122	1.412	809	0.047521981	isogroup16178	ENSG00000133895	ENST00000443283	MEN1
isotig31090	1.037	1.836	1.826	1.937	809	0.270449763	isogroup16181	ENSG00000116871	ENST00000373151	MAP7D1
isotig31092	6.492	2.941	3.333	2.048	809	0.573589088	isogroup16183	ENSG00000145632	ENST00000274289	PLK2
isotig31095	1.333	2.706	2.67	2.878	809	0.317417525	isogroup16186	ENSG00000168542	ENST00000317840	COL3A1

isotig31096	3.431	2.964	2.009	1.743	809	0.194089577	isogroup16187	ENSG00000105750	ENST00000421385	ZNF85
isotig31099	1.261	1.578	1.463	1.358	808	0.297352897	isogroup16190	ENSG00000138119	ENST00000463743	MYOF
isotig31100	5.067	5.913	2.376	3.214	808	0.38356692	isogroup16191	ENSG00000059573	ENST00000371224	ALDH18A1
isotig31101	2.595	1.908	2.537	1.718	808	0.256772751	isogroup16192	ENSG00000119408	ENST00000540326	NEK6
isotig31104	2.224	3.51	5.302	4.35	808	0.014428496	isogroup16195	ENSG00000163558	ENST00000295797	PRKCI
isotig31108	3.139	3.51	2.697	2.825	808	0.025447133	isogroup16199	ENSG00000255103	ENST00000530275	KIAA0754
isotig31111	3.337	2.073	3.863	2.134	808	0.565736079	isogroup16202	ENSG00000092758	ENST00000537652	COL9A3
isotig31112	1.334	1.979	2.583	2.632	808	0.193197189	isogroup16203	ENSG00000181222	ENST00000322644	POLR2A
isotig31115	8.7	5.653	6.307	5.153	808	0.387803412	isogroup16206	ENSG00000131781	ENST00000441068	FMO5
isotig31120	1.385	2.948	3.341	2.857	807	0.259590817	isogroup16211	ENSG00000198083	ENST00000431129	KRTAP9-9
isotig31121	3.658	2.568	5.073	5.292	808	0.099505899	isogroup16212	ENSG00000180353	ENST00000428394	HCLS1
isotig31122	5.073	3.916	2.219	3.052	807	0.204150071	isogroup16213	ENSG00000061987	ENST00000546600	MON2
isotig31125	1.361	2.736	3.455	3.079	807	0.126700233	isogroup16216	ENSG00000175727	ENST00000539039	MLXIP
isotig31126	5.766	18.515	1.579	2.081	807	0.924457053	isogroup16217	ENSG00000204291	ENST00000375001	COL15A1
isotig31127	2.03	1.297	1.67	1.747	807	0.175011272	isogroup16218	ENSG00000197362	ENST00000491431	ZNF786
isotig31129	1.931	3.019	2.722	2.275	807	0.194089577	isogroup16220	ENSG00000181481	ENST00000535605	RNF135
isotig31130	8.736	6.981	12.607	7.601	807	0.480808973	isogroup16221	ENSG00000158747	ENST00000289749	NBL1
isotig31131	1.561	1.653	2.474	2.695	806	0.039415345	isogroup16222	ENSG00000186675	ENST00000373359	MAGEE2
isotig31132	3.84	1.629	2.589	1.361	806	0.639973322	isogroup16223	ENSG00000134644	ENST00000525997	PUM1
isotig31133	2.602	1.629	1.924	1.722	806	0.360609078	isogroup16224	ENSG00000198844	ENST00000421050	ARHGEF15
isotig31134	2.751	4.959	6.826	6.61	806	0.232368302	isogroup16225	ENSG00000091140	ENST00000539590	DLD
isotig31136	2.155	2.337	2.978	1.722	806	0.399348087	isogroup16227	ENSG00000173928	ENST00000312423	SWSAP1
isotig31139	2.06	2.766	1.491	1.863	805	0.311471406	isogroup16230	ENSG00000164933	ENST00000297578	SLC25A32
isotig31142	4.565	3.406	4.646	3.767	806	0.407999549	isogroup16233	ENSG00000143578	ENST00000368607	CREB3L4
isotig31589	1.36	1.37	1.021	1.812	778	0.242607274	isogroup16680	ENSG00000099834	ENST00000397542	CDHR5
isotig31592	1.419	6.492	1.121	1.099	774	0.775512888	isogroup16683	ENSG00000165810	ENST00000491209	BTNL9
isotig31593	14.067	2.569	5.958	5.352	778	0.900108965	isogroup16684	ENSG00000203814	ENST00000545683	HIST2H2BF
isotig31595	4.595	2.251	3.204	2.648	778	0.580690614	isogroup16686	ENSG00000117308	ENST00000374497	GALE
isotig31600	2.31	2.275	2.041	1.698	778	0.095447885	isogroup16691	ENSG00000158683	ENST00000289672	PKD1L1
isotig32087	5.599	2.541	5.67	3.168	749	0.771248215	isogroup17178	ENSG00000132688	ENST00000368223	NES
isotig32088	1.845	1.452	1.483	1.408	747	0.183315172	isogroup17179	ENSG00000106125	ENST00000409881	FAM188B
isotig32090	1.359	2.36	2.289	2.805	750	0.358072819	isogroup17181	ENSG00000120055	ENST00000239125	C10orf95
isotig32094	15.465	3.862	11.513	6.366	749	0.907323213	isogroup17185	ENSG00000130699	ENST00000252996	TAF4
isotig32097	84.544	57.459	82.057	67.728	750	0.599609228	isogroup17188	ENSG00000089693	ENST00000539187	MLF2
isotig32098	1.359	2.436	2.905	2.836	750	0.190473059	isogroup17189	ENSG00000165240	ENST00000350425	ATP7A
isotig32105	1.065	2.107	1.6	2.029	750	0.340272037	isogroup17196	ENSG00000129749	ENST00000534359	CHRNA10
isotig32107	1.84	1.829	2.096	2.242	749	0.034718569	isogroup17198	ENSG00000205593	ENST00000413817	FAM116B
isotig32108	4.758	4.996	4.573	3.766	743	0.174325543	isogroup17199	ENSG00000140307	ENST00000396061	GTF2A2
isotig32111	20.318	13.291	21.866	16.977	749	0.622820696	isogroup17202	ENSG00000184990	ENST00000329967	SIVA1
isotig32112	2.746	1.601	2.047	1.853	749	0.36140753	isogroup17203	ENSG00000212857	ENST00000391545	AL356585.1
isotig32113	1.599	2.515	2.563	2.84	749	0.190473059	isogroup17204	ENSG00000127152	ENST00000357195	BCL11B
isotig32117	1.546	2.745	2.983	2.72	749	0.129649808	isogroup17208	ENSG00000228672	ENST00000434752	C5orf65
isotig32118	3.252	4.194	3.673	3.318	749	0.19978207	isogroup17209	ENSG00000107938	ENST00000356792	C10orf137
isotig32120	2.59	1.171	1.358	1.377	748	0.32967611	isogroup17211	ENSG00000256223	ENST00000426665	ZNF10
isotig32587	1.406	1.444	2.828	2.007	725	0.249943639	isogroup17678	ENSG00000100578	ENST00000556134	KIAA0586
isotig32588	3.591	6.871	4.649	5.357	723	0.624201548	isogroup17679	ENSG00000150907	ENST00000379561	FOXO1
isotig32589	1.157	2.048	2.903	2.593	725	0.108908845	isogroup17680	ENSG00000169876	ENST00000379439	MUC17
isotig32591	1.295	2.363	1.91	2.069	725	0.335049222	isogroup17682	ENSG00000020129	ENST00000373253	NCDN
isotig32592	1.378	2.127	2.572	2.687	725	0.17921019	isogroup17683	ENSG00000133226	ENST00000447431	SRRM1
isotig32594	3.058	2.232	4.101	2.872	725	0.50428346	isogroup17685	ENSG00000187642	ENST00000433179	C1orf170
isotig32595	1.931	3.155	1.352	2.443	724	0.57824829	isogroup17686	ENSG00000206282	ENST00000547127	RGL2
isotig32596	4.083	1.577	2.805	1.917	724	0.547578342	isogroup17687	ENSG00000185909	ENST00000332780	KLHDC8B
isotig32600	2.321	2.238	2.784	2.26	723	0.221302698	isogroup17691	ENSG00000197380	ENST00000391916	DACT3
isotig32604	1.384	1.398	1.253	1.054	722	0.10667318	isogroup17695	ENSG00000187889	ENST00000343433	C1orf168
isotig32606	5.076	2.709	3.928	3.865	724	0.624398813	isogroup17697	ENSG00000108557	ENST00000353383	RAI1
isotig32614	1.269	2.182	2.652	2.102	724	0.098416247	isogroup17705	ENSG00000239886	ENST00000455970	KRTAP9-2
isotig32622	2.183	3.369	1.073	1.393	723	0.396548809	isogroup17713	ENSG00000198677	ENST00000358746	TTC37
isotig32623	1.188	2.08	2.069	2.91	723	0.384139926	isogroup17714	ENSG00000196365	ENST00000540670	LONP1
isotig32625	2.651	3.502	2.707	2.415	723	0.021652138	isogroup17716	ENSG00000116874	ENST00000235521	WARS2
isotig32626	1.851	1.923	1.303	1.95	723	0.210133764	isogroup17717	ENSG00000165806	ENST00000369315	CASP7
isotig32631	3.145	2.523	2.269	1.546	724	0.402109792	isogroup17722	ENSG00000138640	ENST00000508369	FAM13A
isotig32633	3.701	4.001	3.882	3.004	723	0.076068986	isogroup17724	ENSG00000196387	ENST00000355557	ZNF140
isotig32634	11.064	8.673	6.547	6.884	722	0.281919291	isogroup17725	ENSG00000196504	ENST00000410080	PRPF40A
isotig32635	7.136	44.317	33.298	39.41	722	0.805018036	isogroup17726	ENSG00000130222	ENST00000252506	GADD45G
isotig32636	2.406	2.294	2.507	1.953	722	0.126700233	isogroup17727	ENSG00000178691	ENST00000322652	SUZ12
isotig32637	3.734	2.162	3.043	2.511	722	0.456723905	isogroup17728	ENSG00000070614	ENST00000523767	NDST1
isotig32639	6.058	4.007	6.522	4.526	722	0.611332381	isogroup17730	ENSG00000181588	ENST00000402693	MEX3D
isotig32640	36.014	5.062	9.386	3.039	722	0.964839934	isogroup17731	ENSG00000078814	ENST00000262873	MYH7B
isotig32641	1.891	1.936	1.312	1.621	718	0.060513264	isogroup17732	ENSG00000086619	ENST00000354619	ERO1LB
isotig32643	1.576	1.819	3.248	3.162	722	0.071109191	isogroup17734	ENSG00000204038	ENST00000356374	AL359195.1
isotig32645	3.457	2.452	2.404	3.039	722	0.010060494	isogroup17736	ENSG00000158470	ENST00000371711	B4GALT5
isotig32650	2.13	2.953	3.964	4.31	722	0.148117532	isogroup17741	ENSG00000163362	ENST00000413687	C1orf106
isotig32651	1.328	2.241	2.891	2.946	722	0.180675584	isogroup17742	ENSG00000120055	ENST00000239125	C10orf95
isotig32652	1.051	1.687	2.276	2.45	722	0.108307658	isogroup17743	ENSG00000136378	ENST00000388820	ADAMTS7
isotig32654	1.273	2.662	3.402	3.504	722	0.126700233	isogroup17745	ENSG00000133226	ENST00000323848	SRRM1
isotig32655	2.572	3.374	3.094	2.698	722	0.017143233	isogroup17746	ENSG00000080603	ENST00000395059	SRCAP
isotig32662	6.897	2.692	2.868	1.459	721	0.752245059	isogroup17753	ENSG00000165757	ENST00000375377	KIAA1462
isotig32664	2.908	3.723	2.202	2.918	721	0.291472533	isogroup17755	ENSG00000132881	ENST00000375599	RSG1
isotig32665	2.327	1.795	2.254	2.111	721	0.166209514	isogroup17756	ENSG00000119596	ENST00000549293	YLPM1
isotig32666	1.911	2.297	1.51	1.459	721	0.034718569	isogroup17757	ENSG00000132182	ENST00000254508	NUP210
isotig32669	3.822	4.251	2.868	2.515	721	0.082137221	isogroup17760	ENSG00000130307	ENST00000431146	USHBP1
isotig32670	3.356	2.062	2.796	2.053	720	0.326566845	isogroup17761	ENSG00000221986	ENST00000357155	MYBPHL

isotig32672	1.219	1.928	2.075	2.391	721	0.218484632	isogroup17763	ENSG00000175600	ENST00000335693	C7orf10
isotig32674	4.022	3.965	1.718	2.457	720	0.21337454	isogroup17765	ENSG00000114982	ENST00000440133	KANSL3
isotig32675	2.94	2.221	1.846	1.928	720	0.028237018	isogroup17766	ENSG00000147526	ENST00000522904	TACC1
isotig32683	1.054	1.771	2.333	3.11	720	0.331611182	isogroup17774	ENSG00000159131	ENST00000430874	GART
isotig32684	6.963	7.429	6.796	4.694	720	0.221180582	isogroup17775	ENSG00000177989	ENST00000401779	ODF3B
isotig32687	16.032	15.069	18.674	23.822	720	0.129208311	isogroup17778	ENSG00000100994	ENST00000216962	PYGB
isotig32688	2.746	1.744	1.769	2.55	720	0.014428496	isogroup17779	ENSG00000182095	ENST00000430969	TNRC18
isotig32690	1.825	2.927	2.967	2.388	722	0.245829263	isogroup17781	ENSG00000064419	ENST00000482320	TNPO3
isotig32693	1.886	2.669	4.308	3.825	720	0.077224393	isogroup17784	ENSG00000153902	ENST00000392225	LGI4
isotig32695	1.692	2.458	3.181	2.922	720	0.028237018	isogroup17786	ENSG00000120055	ENST00000239125	C10orf95
isotig32706	1.722	2.462	4.391	3.331	719	0.150869843	isogroup17797	ENSG00000178035	ENST00000429182	IMPDH2
isotig32707	2.033	3.238	2.678	1.81	717	0.157116555	isogroup17798	ENSG00000138050	ENST00000505747	THUMPD2
isotig32708	2.555	3.177	4.957	4.919	719	0.061837755	isogroup17799	ENSG00000104687	ENST00000541648	GSR
isotig32709	8.611	8.868	10.94	8.75	719	0.246994063	isogroup17800	ENSG00000187051	ENST00000334678	RPS19BP1
isotig32711	8.388	14.268	11.043	11.707	719	0.546300819	isogroup17802	ENSG00000125652	ENST00000245812	ALKBH7
isotig32713	3.36	3.467	4.879	2.771	719	0.326942587	isogroup17804	ENSG00000122367	ENST00000361373	LDB3
isotig32714	1.362	2.594	3.082	1.339	719	0.080728188	isogroup17805	ENSG00000175707	ENST00000320567	C1orf172
isotig32715	1.167	2.092	2.363	3.051	719	0.258153603	isogroup17806	ENSG00000165092	ENST00000297785	ALDH1A1
isotig32716	1.334	2.039	2.722	2.242	719	0.010060494	isogroup17807	ENSG00000127954	ENST00000414498	STEAP4
isotig32721	13.583	8.604	9.887	5.885	719	0.656214774	isogroup17812	ENSG00000184160	ENST00000509482	ADRA2C
isotig32722	1.499	1.668	1.747	2.335	719	0.192314196	isogroup17813	ENSG00000139835	ENST00000375431	GRTP1
isotig32724	72.467	32.166	81.563	60.84	719	0.75914932	isogroup17815	ENSG00000091536	ENST00000205890	MYO15A
isotig32726	1.029	2.624	3.241	3.398	718	0.332278124	isogroup17817	ENSG00000172901	ENST00000379578	LVRN.1
isotig32728	4.006	2.703	1.826	1.777	718	0.35670136	isogroup17819	ENSG00000106772	ENST00000428286	PRUNE2
isotig32729	2.614	2.571	2.006	1.684	718	0.050227324	isogroup17820	ENSG00000153922	ENST00000422663	CHD1
isotig32731	12.099	13.334	10.26	18.084	718	0.464135417	isogroup17822	ENSG00000128591	ENST00000346177	FLNC
isotig32732	2.031	10.074	4.603	3.461	718	0.842498309	isogroup17823	ENSG00000077274	ENST00000324068	CAPN6
isotig32735	2.281	2.439	1.981	1.652	718	0.003503795	isogroup17826	ENSG00000188827	ENST00000294008	SLX4
isotig32737	1.641	2.412	3.008	3.336	718	0.106053205	isogroup17828	ENSG00000119535	ENST00000338937	CSF3R
isotig32740	1.029	2.015	2.315	2.401	718	0.201397761	isogroup17831	ENSG00000153902	ENST00000392225	LGI4
isotig32744	1.587	2.522	3.477	3.997	717	0.173724356	isogroup17835	ENSG00000172534	ENST00000369984	HCFC1
isotig32745	52.612	50.171	52.032	50.699	714	0.056943714	isogroup17836	ENSG00000172590	ENST00000397496	MRPL52
isotig32746	3.425	2.283	4.893	2.529	717	0.644688886	isogroup17837	ENSG00000105647	ENST00000426902	PIK3R2
isotig32750	1.559	4.114	3.605	2.997	717	0.36987112	isogroup17841	ENSG00000047648	ENST00000534860	ARHGAP6
isotig32751	2.229	1.222	2.729	1.842	717	0.469724581	isogroup17842	ENSG00000160796	ENST00000450053	NBEAL2
isotig32753	2.785	2.628	2.55	1.686	717	0.054116255	isogroup17844	ENSG00000155508	ENST00000542339	CNOT8
isotig32754	6.768	5.045	7.7	5.621	717	0.497980386	isogroup17845	ENSG00000127554	ENST00000248114	GFER
isotig32756	5.209	3.185	1.854	2.904	717	0.333442925	isogroup17847	ENSG00000085719	ENST00000521271	CPNE3
isotig32759	1.531	2.283	2.575	2.092	717	0.306915533	isogroup17850	ENSG00000185340	ENST00000406549	GAS2L1
isotig32760	6.471	4.573	5.158	3.392	716	0.520712783	isogroup17851	ENSG00000105372	ENST00000221975	RPS19
isotig32761	1.503	2.018	1.417	1.061	717	0.010060494	isogroup17852	ENSG00000163913	ENST00000504021	IFT122
isotig32765	4.54	4.778	2.729	1.593	717	0.061837755	isogroup17856	ENSG00000196411	ENST00000358173	EPHB4
isotig32766	1.727	3.291	4.25	4.152	717	0.097401743	isogroup17857	ENSG00000173566	ENST00000522379	NUDT18
isotig32767	10.586	6.948	14.54	8.736	715	0.716408657	isogroup17858	ENSG00000049759	ENST00000456986	NEDD4L
isotig32768	2.285	1.619	1.905	2.061	717	0.354681746	isogroup17859	ENSG00000114867	ENST00000457456	EIF4G1
isotig32770	1.534	1.807	1.521	1.626	716	0.047521981	isogroup17861	ENSG00000143458	ENST00000446567	GABPB2
isotig32772	1.617	1.25	1.754	1.751	716	0.116996693	isogroup17863	ENSG00000110318	ENST00000532529	KIAA1377
isotig32773	4.909	2.074	3.327	2.032	716	0.644688886	isogroup17864	ENSG00000144677	ENST00000273179	CTDSPL
isotig32774	4.156	2.101	1.831	1.719	716	0.460246487	isogroup17865	ENSG00000130787	ENST00000253083	HIP1R
isotig32775	1.172	1.86	2.398	2.282	716	0.106814083	isogroup17866	ENSG00000146166	ENST00000485906	LGSN
isotig32778	22.983	20.922	30.56	41.49	716	0.072048546	isogroup17869	ENSG00000205978	ENST00000382554	NYNRIN
isotig32780	1.673	2.898	2.733	2.877	716	0.18122041	isogroup17871	ENSG00000011376	ENST00000414984	LARS2
isotig32783	4.434	1.968	2.682	2.001	716	0.588975727	isogroup17874	ENSG00000179094	ENST00000317276	PER1
isotig32786	4.49	3.669	3.534	4.659	716	0.155266025	isogroup17877	ENSG00000123908	ENST00000519980	EIF2C2
isotig32787	1.284	2.446	2.425	2.908	716	0.271680319	isogroup17878	ENSG00000167978	ENST00000544933	SRRM2
isotig32788	13.732	10.884	12.568	9.117	717	0.395496731	isogroup17879	ENSG00000160124	ENST00000291458	CCDC58
isotig32790	2.785	2.389	1.622	1.936	717	0.106053205	isogroup17881	ENSG00000168952	ENST00000546511	STXBP6
isotig32791	1.648	2.555	1.085	1.597	715	0.427951454	isogroup17882	ENSG00000066739	ENST00000359933	ATG2B
isotig32794	2.485	1.89	2.144	1.659	715	0.210180732	isogroup17885	ENSG00000081913	ENST00000400316	PHLPP1
isotig33087	1.37	2.856	3.561	2.879	700	0.112901105	isogroup18178	ENSG00000164073	ENST00000513559	MFSD8
isotig33089	2.91	2.013	2.69	1.854	700	0.4144811	isogroup18180	ENSG00000187609	ENST00000479452	EXD3
isotig33090	4.85	5.303	3.851	2.783	700	0.090957767	isogroup18181	ENSG00000100599	ENST00000556418	RIN3
isotig33092	2.767	3.726	2.981	2.879	700	0.21337454	isogroup18183	ENSG00000167625	ENST00000301215	ZNF526
isotig33095	2.996	1.141	2.347	1.471	700	0.517706846	isogroup18186	ENSG00000126464	ENST00000418929	PRR12
isotig33100	6.819	9.463	7.386	5.501	700	0.143157737	isogroup18191	ENSG00000175287	ENST00000426694	PHYHD1
isotig33103	3.48	2.094	1.873	1.311	700	0.304482603	isogroup18194	ENSG00000099331	ENST00000397274	MYO9B
isotig33104	44.307	46.471	64.864	77.266	700	0.374530322	isogroup18195	ENSG00000185340	ENST00000333679	GAS2L1
isotig33105	5.25	5.737	4.537	6.013	700	0.281524761	isogroup18196	ENSG00000179335	ENST00000568488	CLK3
isotig33107	3.286	5.582	5.864	6.117	699	0.251822349	isogroup18198	ENSG00000129250	ENST00000320785	KIF1C
isotig33108	3.485	1.715	1.823	1.986	699	0.419525438	isogroup18199	ENSG00000119682	ENST00000555249	KIAA0317
isotig33112	1.571	2.777	4.016	2.627	699	0.03231382	isogroup18203	ENSG00000155495	ENST00000285879	MAGEC1
isotig33115	4.114	5.037	1.057	2.273	699	0.621468024	isogroup18206	ENSG00000091409	ENST00000442250	ITGA6
isotig33116	1.057	1.742	1.797	2.306	699	0.275747727	isogroup18207	ENSG00000065154	ENST00000368845	OAT
isotig33120	5.98	3.872	7.037	5.259	698	0.50243293	isogroup18211	ENSG00000237190	ENST00000458198	CDKN2AIPNL
isotig33122	1.485	2.887	3.09	3.01	699	0.194089577	isogroup18213	ENSG00000204262	ENST00000452536	COL5A2
isotig33123	1.914	3.268	4.887	3.907	699	0.123543999	isogroup18214	ENSG00000084636	ENST00000373672	COL16A1
isotig33125	65.378	11.726	18.861	13.407	698	0.962500939	isogroup18216	ENSG00000129991	ENST00000344887	TNNI3
isotig33127	3.234	1.282	2.143	1.347	698	0.59797475	isogroup18218	ENSG00000198315	ENST00000536028	ZNF192
isotig33131	1.345	1.718	1.085	2.501	698	0.473801383	isogroup18222	ENSG00000188786	ENST00000373036	MTF1
isotig33132	2.861	2.973	2.804	1.86	698	0.115756745	isogroup18223	ENSG00000112214	ENST00000541107	FHL5
isotig33133	1.544	1.991	1.112	1.251	698	0.105508379	isogroup18224	ENSG00000005810	ENST00000407578	MYCBP2
isotig33134	2.489	1.636	1.613	1.219	698	0.361050575	isogroup18225	ENSG00000168542	ENST00000304636	COL3A1

isotig33135	6.324	4.145	7.381	3.977	698	0.683935147	isogroup18226	ENSG00000040633	ENST00000320316	PHF23
isotig33138	6.752	3.191	6.057	3.785	698	0.732189825	isogroup18229	ENSG00000222033	ENST00000409786	AC007405.2.1
isotig33141	1.345	2.4	3.095	2.791	698	0.077975877	isogroup18232	ENSG00000171763	ENST00000559860	SPATA5L1
isotig33142	6.095	11.754	28.782	20.88	698	0.482734651	isogroup18233	ENSG00000179979	ENST00000324803	CRIPAK
isotig33143	5.236	4.254	3.519	3.079	698	0.185475689	isogroup18234	ENSG00000135439	ENST00000547588	AGAP2
isotig33144	3.119	2.372	2.46	2.854	698	0.152945818	isogroup18235	ENSG00000164889	ENST00000485713	SLC4A2
isotig33149	1.374	2.128	2.751	3.047	698	0.136638611	isogroup18240	ENSG00000153902	ENST00000392225	LGI4
isotig33156	13.963	2.046	8.703	4.042	698	0.927726009	isogroup18247	ENSG00000110723	ENST00000533052	EXPH5
isotig33157	2.46	1.201	2.54	1.603	698	0.456836627	isogroup18248	ENSG00000135074	ENST00000517905	ADAM19
isotig33158	1.403	1.799	1.984	1.924	698	0.095898775	isogroup18249	ENSG00000251359	ENST00000506413	C4orf38
isotig33159	2.463	3.987	5.696	4.561	697	0.077571955	isogroup18250	ENSG00000258429	ENST00000288022	PDF
isotig33160	2.034	2.923	3.364	2.923	697	0.292064327	isogroup18251	ENSG00000105556	ENST00000264819	MIER2
isotig33163	2.35	2.923	2.463	1.735	697	0.003503795	isogroup18254	ENSG00000076928	ENST00000316079	ARHGEF1
isotig33164	1.462	2.349	2.623	2.538	697	0.250018787	isogroup18255	ENSG00000117594	ENST00000367027	HSD11B1
isotig33165	6.551	3.944	7.891	6.475	695	0.480846547	isogroup18256	ENSG00000154928	ENST00000398015	EPHB1
isotig33166	2.121	13.08	3.443	1.188	697	0.841934696	isogroup18257	ENSG00000160307	ENST00000397648	S100B
isotig33168	237.594	193.011	218.011	234.725	699	0.137991283	isogroup18259	ENSG00000136717	ENST00000259238	BIN1
isotig33172	1.032	2.131	3.126	2.152	697	0.084617119	isogroup18263	ENSG00000076826	ENST00000446248	CAMSAP3
isotig33173	4.533	5.032	8.807	5.951	696	0.454723078	isogroup18264	ENSG00000106299	ENST00000223023	WASL
isotig33178	6.342	5.688	2.069	3.602	696	0.126258736	isogroup18269	ENSG00000217128	ENST00000544351	FNIP1
isotig33179	1.464	2.761	3.635	3.409	696	0.232884948	isogroup18270	ENSG00000225312	ENST00000454191	HSD17B8
isotig33181	1.549	2.543	3.05	2.927	696	0.126700233	isogroup18272	ENSG00000103569	ENST00000536493	AQP9
isotig33184	4.103	4.211	2.652	2.766	696	0.127818066	isogroup18275	ENSG00000139722	ENST00000267202	VPS37B
isotig33185	2.582	1.586	2.096	1.93	696	0.295887503	isogroup18276	ENSG00000197128	ENST00000319969	ZNF772
isotig33188	1.38	3.314	3.427	3.157	695	0.350313745	isogroup18279	ENSG00000059573	ENST00000371224	ALDH18A1
isotig33191	8.965	12.708	15.542	12.885	695	0.019651311	isogroup18282	ENSG00000213563	ENST00000534680	C8orf82
isotig33195	3.276	3.807	3.506	1.868	695	0.22553919	isogroup18286	ENSG00000164885	ENST00000485972	CDK5
isotig33198	3.793	2.109	1.514	1.578	695	0.386215901	isogroup18289	ENSG00000129566	ENST00000262715	TEP1
isotig33203	1.265	2.137	2.577	1.965	695	0.087435184	isogroup18294	ENSG00000184956	ENST00000421673	MUC6
isotig33204	1.87	1.837	2.474	2.161	694	0.162226648	isogroup18295	ENSG00000063245	ENST00000544375	EPN1
isotig33206	1.18	1.865	2.448	3.161	694	0.301176073	isogroup18297	ENSG00000120913	ENST00000308354	PDLIM2
isotig33207	1.496	2.414	2.501	2.677	694	0.271248215	isogroup18298	ENSG00000117983	ENST00000349637	MUC5B
isotig33215	1.209	1.427	2.794	2.419	694	0.087435184	isogroup18306	ENSG00000150401	ENST00000438545	DCUN1D2
isotig33216	9.382	4.854	4.151	3.419	694	0.678693545	isogroup18307	ENSG00000059145	ENST00000508903	UNKL
isotig33588	2.709	2.162	1.689	1.288	678	0.212425791	isogroup18679	ENSG00000155657	ENST00000360870	TTN
isotig33590	3.652	3.173	5.665	3.103	678	0.576613812	isogroup18681	ENSG00000101665	ENST00000545051	SMAD7
isotig33591	3.299	3.593	4.249	2.807	678	0.270534305	isogroup18682	ENSG00000229363	ENST00000549252	RDBP
isotig33592	15.229	13.055	16.476	11.689	678	0.603986624	isogroup18683	ENSG00000059915	ENST00000541902	PSD
isotig33593	2.945	2.386	2.886	1.618	678	0.326933193	isogroup18684	ENSG00000133318	ENST00000543552	RTN3
isotig33595	4.396	4.527	3.49	4.563	677	0.197283385	isogroup18686	ENSG00000170265	ENST00000479907	ZNF282
isotig33596	1.092	1.996	2.945	2.843	677	0.039415345	isogroup18687	ENSG00000079805	ENST00000408974	DNM2
isotig33597	2.536	2.502	3.028	2.414	677	0.310419328	isogroup18688	ENSG00000176842	ENST00000320990	IRX5
isotig33599	1.357	2.502	2.809	2.942	677	0.187185316	isogroup18690	ENSG00000138468	ENST00000394091	SENP7
isotig33604	1.298	1.883	2.7	2.744	677	0.091699857	isogroup18695	ENSG00000184860	ENST00000328945	SDR42E1
isotig33605	2.713	1.462	2.919	2.083	677	0.432263095	isogroup18696	ENSG00000068654	ENST00000263857	POLR1A
isotig33607	2.92	2.194	2.319	1.62	677	0.45639513	isogroup18698	ENSG00000187741	ENST00000567943	FANCA
isotig33608	157.555	40.13	66.322	23.467	677	0.983777335	isogroup18699	ENSG00000078814	ENST00000262873	MYH7B
isotig33614	1.003	2.334	2.072	2.448	677	0.383022094	isogroup18705	ENSG00000214248	ENST00000539278	AC010336.2
isotig33615	1.507	2.675	3.442	3.377	676	0.014428496	isogroup18706	ENSG00000258539	ENST00000494792	RP11-12J10.3.1
isotig33620	1.595	2.59	2.95	2.781	676	0.18122041	isogroup18711	ENSG00000213619	ENST00000263774	NDUFS3
isotig33621	1.831	1.38	1.038	1.225	676	0.042270985	isogroup18712	ENSG00000145715	ENST00000274376	RASA1
isotig33622	5.022	3.774	4.043	2.385	676	0.438350116	isogroup18713	ENSG00000187626	ENST00000377294	ZKSCAN4
isotig33623	9.572	4.562	12.374	10.365	676	0.67460735	isogroup18714	ENSG00000180787	ENST00000318833	ZFP3
isotig33626	16.574	6.448	2.485	3.146	676	0.765039077	isogroup18717	ENSG00000166987	ENST00000431731	MBD6
isotig33630	1.861	2.506	3.087	3.213	676	0.074481476	isogroup18721	ENSG00000163655	ENST00000541628	GMPS
isotig33632	5.496	3.463	2.404	1.059	676	0.61368077	isogroup18723	ENSG00000144959	ENST00000475381	NCEH1
isotig33635	1.624	2.393	2.814	3.643	676	0.263179154	isogroup18726	ENSG00000171357	ENST00000371980	C1orf190
isotig33639	7.692	5.95	9.547	6.501	675	0.511760727	isogroup18730	ENSG00000176261	ENST00000468695	ZBTB8OS
isotig33640	2.101	2.2	2.681	2.289	675	0.039415345	isogroup18731	ENSG00000119688	ENST00000356924	ABCD4
isotig33641	4.409	1.692	4.021	2.621	675	0.715178102	isogroup18732	ENSG00000196700	ENST00000450537	ZNF512B
isotig33642	1.154	2.481	3.119	2.785	675	0.227483655	isogroup18733	ENSG00000172893	ENST00000525137	DHCR7
isotig33646	1.967	2.103	2.233	2.239	670	0.184395431	isogroup18737	ENSG00000155115	ENST00000329970	GTF3C6
isotig33648	2.338	2.228	1.861	2.52	675	0.010060494	isogroup18739	ENSG00000063169	ENST00000396720	GLTSCR1
isotig33650	2.19	2.453	7.769	8.921	675	0.131340648	isogroup18741	ENSG00000141068	ENST00000398985	KSR1
isotig33651	2.249	4.258	4.65	4.41	675	0.141889607	isogroup18742	ENSG00000158636	ENST00000533248	C11orf30
isotig33652	10	8.799	18.711	20.298	675	0.019651311	isogroup18743	ENSG00000008056	ENST00000340666	SYN1
isotig33653	20.917	17.314	16.167	14.56	675	0.317173292	isogroup18744	ENSG00000179271	ENST00000316939	GADD45GIP1
isotig33654	1.796	3.604	2.963	2.467	675	0.479550237	isogroup18745	ENSG00000146426	ENST00000528391	TIAM2
isotig33657	1.753	3.766	1.593	1.667	675	0.481654392	isogroup18748	ENSG00000240563	ENST00000498273	L1TD1
isotig33658	1.538	3.159	3.72	4.212	675	0.296319606	isogroup18749	ENSG00000140983	ENST00000569197	RHOT2
isotig33659	1.953	4.089	3.228	2.52	675	0.193300519	isogroup18750	ENSG00000012211	ENST00000540849	PRICKLE3
isotig33662	2.547	2.823	2.438	3.355	674	0.242062448	isogroup18753	ENSG00000188827	ENST00000294008	SLX4
isotig33668	1.364	2.429	3.534	3.288	674	0.136507102	isogroup18759	ENSG00000006695	ENST00000537334	COX10
isotig33669	2872.38	3003.948	1294.872	1838.918	671	0.396088525	isogroup18760	ENSG00000092054	ENST00000544444	MYH7
isotig33670	1.393	1.892	2.521	3.022	674	0.087435184	isogroup18761	ENSG00000122971	ENST00000242592	ACADS
isotig33674	2.755	6.636	3.288	4.185	674	0.601224919	isogroup18765	ENSG00000172519	ENST00000308940	OR10H5
isotig33678	1.512	2.88	3.671	3.355	674	0.152945818	isogroup18769	ENSG00000134250	ENST00000256646	NOTCH2
isotig33679	1.156	2.542	3.644	3.654	674	0.174287969	isogroup18770	ENSG00000179598	ENST00000427497	PLD6
isotig33683	7.152	5.826	1.866	2.793	673	0.129086195	isogroup18774	ENSG00000181827	ENST00000559447	RFX7
isotig33688	41.514	49.636	67.055	50.395	673	0.259618998	isogroup18779	ENSG00000161011	ENST00000454378	SQSTM1
isotig33690	2.67	2.094	3.293	3.759	673	0.059555121	isogroup18781	ENSG00000099904	ENST00000334554	ZDHHC8
isotig33700	6.262	2.715	3.511	1.996	673	0.711354926	isogroup18791	ENSG00000170873	ENST00000518547	MTSS1

isotig33703	1.574	2.21	2.638	2.432	672	0.039415345	isogroup18794	ENSG00000167978	ENST00000544933	SRRM2
isotig33707	6.845	11.8	17.171	14.68	671	0.051805441	isogroup18798	ENSG00000234876	ENST00000457094	NOTCH4
isotig33709	1.397	2.295	3.71	2.698	672	0.170915683	isogroup18800	ENSG00000167978	ENST00000544933	SRRM2
isotig33710	1.368	2.662	3.682	2.765	672	0.010060494	isogroup18801	ENSG00000104687	ENST00000546342	GSR
isotig33712	3.21	2.662	1.841	1.866	672	0.064064026	isogroup18803	ENSG00000136051	ENST00000332180	KIAA1033
isotig33713	3.269	2.097	2.802	1.6	672	0.508736004	isogroup18804	ENSG00000061918	ENST00000502959	GUCY1B3
isotig33715	7.49	2.804	4.177	2.499	672	0.781374465	isogroup18806	ENSG00000159884	ENST00000426546	CCDC107
isotig33717	179.502	219.557	233.339	202.111	675	0.041444353	isogroup18808	ENSG00000182035	ENST00000537425	ADIG
isotig33722	4.256	4.142	2.806	2.903	671	0.025447133	isogroup18813	ENSG00000150457	ENST00000542899	LATS2
isotig34087	2.398	2.488	2.778	1.123	658	0.312213497	isogroup19178	ENSG00000183248	ENST00000539422	AC010336.1
isotig34090	1.763	3.592	3.709	3.645	657	0.287414519	isogroup19181	ENSG00000196663	ENST00000380088	TECPR2
isotig34092	3.982	2.318	2.755	2.352	657	0.440360337	isogroup19183	ENSG00000225614	ENST00000565624	ZNF469
isotig34093	1.793	2.521	3.373	3.68	657	0.156205381	isogroup19184	ENSG00000126259	ENST00000360202	KIRREL2
isotig34094	54.988	69.271	100.502	122.633	657	0.416510107	isogroup19185	ENSG00000196296	ENST00000536376	ATP2A1
isotig34097	5.997	1.277	4.392	1.399	656	0.854362366	isogroup19188	ENSG00000173085	ENST00000311469	COQ2
isotig34099	10.03	9.387	7.982	5.145	657	0.411475163	isogroup19190	ENSG00000103024	ENST00000219302	NME3
isotig34100	5.836	3.361	4.075	3.645	657	0.532435936	isogroup19191	ENSG00000103037	ENST00000422445	SETD6
isotig34103	1.103	1.693	2.463	2.919	652	0.106814083	isogroup19194	ENSG00000123095	ENST00000540731	BHLHE41
isotig34104	5.927	2.289	4.694	2.93	657	0.778462463	isogroup19195	ENSG00000125637	ENST00000441564	PSD4
isotig34108	1.338	2.81	3.035	2.146	657	0.477455475	isogroup19199	ENSG00000154734	ENST00000284984	ADAMTS1
isotig34109	1.58	2.463	3.12	2.624	657	0.153913354	isogroup19200	ENSG00000165804	ENST00000451119	ZNF219
isotig34112	1.945	3.129	4.16	5.35	657	0.296479297	isogroup19203	ENSG00000126001	ENST00000422671	CEP250
isotig34115	4.621	1.854	1.855	2.011	657	0.505081912	isogroup19206	ENSG00000038382	ENST00000537187	TRIO
isotig34116	3.319	3.163	2.956	1.877	656	0.210781919	isogroup19207	ENSG00000081087	ENST00000193322	OSTM1
isotig34119	1.401	2.611	2.195	1.774	656	0.355010521	isogroup19210	ENSG00000136213	ENST00000258711	CHST12
isotig34120	1.064	1.072	1.349	1.158	657	0.02329601	isogroup19211	ENSG00000159450	ENST00000368804	TCHH
isotig34123	1.735	2.553	3.434	3.378	656	0.183136695	isogroup19214	ENSG00000137992	ENST00000370132	DBT
isotig34127	1.127	2.148	2.505	1.843	656	0.034718569	isogroup19218	ENSG00000145107	ENST00000446879	TM4SF19
isotig34131	2.009	2.351	2.308	1.468	656	0.132824829	isogroup19222	ENSG00000166444	ENST00000534127	ST5
isotig34132	1.096	2.059	2.759	2.014	656	0.006660029	isogroup19223	ENSG00000105321	ENST00000221922	CCDC9
isotig34137	1.553	2.553	3.069	2.662	656	0.09882017	isogroup19228	ENSG00000244486	ENST00000405555	SCARF2
isotig34138	3.38	2.843	1.915	2.014	656	0.088308785	isogroup19229	ENSG00000130702	ENST00000252999	LAMA5
isotig34148	3.994	3.487	1.832	1.777	655	0.049513414	isogroup19239	ENSG00000055609	ENST00000360104	MLL3
isotig34587	2.781	2.74	1.474	1.366	639	0.03231382	isogroup19678	ENSG00000138686	ENST00000506636	BBS7
isotig34588	1.376	2.681	2.947	2.732	639	0.340769896	isogroup19679	ENSG00000130528	ENST00000434964	HRC
isotig34590	2.348	2.328	2.257	1.754	638	0.143242279	isogroup19681	ENSG00000156858	ENST00000542965	PRR14
isotig34593	3.161	2.058	2.981	2.702	638	0.390367852	isogroup19684	ENSG00000092758	ENST00000343916	COL9A3
isotig34596	3.381	2.804	3.183	1.966	638	0.408469227	isogroup19687	ENSG00000122778	ENST00000440172	KIAA1549
isotig34600	1.315	1.73	2.141	2.632	638	0.216728038	isogroup19691	ENSG00000175221	ENST00000269814	MED16
isotig34602	13.137	15.777	15.016	13.496	637	0.019651311	isogroup19693	ENSG00000164167	ENST00000504181	LSM6
isotig34604	1.158	1.969	2.315	2.983	638	0.364732847	isogroup19695	ENSG00000112280	ENST00000357250	COL9A1
isotig34611	1.752	3.252	4.254	3.544	638	0.077224393	isogroup19702	ENSG00000131747	ENST00000423485	TOP2A
isotig34613	1.286	2.421	3.276	2.882	637	0.014428496	isogroup19704	ENSG00000179588	ENST00000319555	ZFPM1
isotig34614	1.16	1.942	2.725	2.531	637	0.010060494	isogroup19705	ENSG00000136531	ENST00000375437	SCN2A
isotig34622	3.266	2.544	1.975	1.197	636	0.266232058	isogroup19713	ENSG00000151693	ENST00000315273	ASAP2
isotig35088	1.031	2.087	2.532	3.394	620	0.32507327	isogroup20179	ENSG00000060971	ENST00000450296	ACAA1
isotig35090	5.895	4.635	1.935	1.985	620	0.310616593	isogroup20181	ENSG00000163714	ENST00000463563	U2SURP
isotig35091	1.579	2.732	3.366	4.081	620	0.379245886	isogroup20182	ENSG00000144485	ENST00000436051	HES6
isotig35092	253.998	116.053	209.835	136.023	618	0.860130007	isogroup20183	ENSG00000168542	ENST00000304636	COL3A1
isotig35098	1.258	2.859	3.042	2.677	619	0.119720824	isogroup20189	ENSG00000117528	ENST00000536817	ABCD3
isotig35587	1.194	2.309	2.668	1.45	602	0.021652138	isogroup20678	ENSG00000162814	ENST00000366933	SPATA17
isotig35591	1.095	1.96	2.269	2.939	602	0.259694146	isogroup20682	ENSG00000152784	ENST00000504452	PRDM8
isotig35594	4.013	2.94	2.453	3.198	602	0.125638762	isogroup20685	ENSG00000129566	ENST00000556935	TEP1
isotig35596	3.583	1.739	3.007	1.748	602	0.585584655	isogroup20687	ENSG00000166507	ENST00000309979	NDST2
isotig35598	1.161	2.467	3.681	2.975	602	0.021652138	isogroup20689	ENSG00000086544	ENST00000263370	ITPKC
isotig35599	4.08	3.636	2.301	1.859	602	0.035704892	isogroup20690	ENSG00000077235	ENST00000561623	GTF3C1
isotig35603	1.128	2.15	2.668	1.897	602	0.057310062	isogroup20694	ENSG00000135299	ENST00000522441	ANKRD6
isotig35605	1.128	1.771	2.638	1.711	602	0.089576914	isogroup20696	ENSG00000116685	ENST00000376576	KIAA2013
isotig35609	4.512	2.055	2.791	1.786	602	0.602990907	isogroup20700	ENSG00000166548	ENST00000564917	TK2
isotig35612	7.878	4.158	7.1	3.627	600	0.750169084	isogroup20703	ENSG00000090006	ENST00000396819	LTBP4
isotig35614	1.329	2.408	2.458	1.973	601	0.157924401	isogroup20705	ENSG00000240505	ENST00000261652	TNFRSF13B
isotig35616	3.023	3.358	3.042	3.651	601	0.046479297	isogroup20707	ENSG00000196428	ENST00000361875	TSC22D2
isotig35619	2.626	2.121	2.12	1.825	601	0.17035207	isogroup20710	ENSG00000232251	ENST00000547450	DPCR1
isotig35622	1.529	1.361	2.428	1.005	601	0.334391674	isogroup20713	ENSG00000056972	ENST00000392555	TRAF3IP2
isotig35624	3.562	1.935	3.662	1.642	600	0.640959645	isogroup20715	ENSG00000090006	ENST00000308370	LTBP4
isotig35625	1.03	2.058	2.028	2.273	601	0.374812129	isogroup20716	ENSG00000114270	ENST00000454817	COL7A1
isotig35628	3.323	4.845	2.304	2.719	601	0.376953859	isogroup20719	ENSG00000164741	ENST00000512044	DLC1
isotig35631	2.89	1.489	1.782	2.346	601	0.309555121	isogroup20722	ENSG00000105784	ENST00000394654	RUNDC3B
isotig35632	1.463	2.661	2.519	2.346	601	0.183258811	isogroup20723	ENSG00000114270	ENST00000454817	COL7A1
isotig35633	3.889	3.958	3.594	3.428	601	0.064082814	isogroup20724	ENSG00000171456	ENST00000421155	ASXL1
isotig35636	4.427	4.252	3.2	1.642	600	0.499934245	isogroup20727	ENSG00000131504	ENST00000546094	DIAPH1
isotig35639	1.562	2.755	3.288	2.757	601	0.021652138	isogroup20730	ENSG00000239264	ENST00000539054	TXNDC5
isotig36092	3.294	5.374	3.423	3.959	588	0.530660555	isogroup21183	ENSG00000172315	ENST00000372114	TP53RK
isotig36093	4.043	2.881	4.648	2.932	588	0.52617983	isogroup21184	ENSG00000198844	ENST00000421050	ARHGEF15
isotig36095	5.027	5.374	6.187	5.179	588	0.046836252	isogroup21186	ENSG00000005961	ENST00000353281	ITGA2B
isotig36099	4.721	2.039	2.544	1.333	588	0.678655971	isogroup21190	ENSG00000102547	ENST00000410043	CAB39L
isotig36101	1.291	2.687	3.265	3.046	588	0.18122041	isogroup21192	ENSG00000064205	ENST00000372865	WISP2
isotig36105	13.235	12.354	14.438	9.877	587	0.431530398	isogroup21196	ENSG00000174306	ENST00000557816	ZHX3
isotig36112	3.198	4.346	5.221	3.584	587	0.236942962	isogroup21203	ENSG00000127124	ENST00000372583	HIVEP3
isotig36115	2.893	2.918	3.523	3.051	587	0.06991621	isogroup21206	ENSG00000116199	ENST00000263733	FAM20B
isotig36117	1.668	2.562	3.555	3.7	587	0.183136695	isogroup21208	ENSG00000157216	ENST00000357475	SSBP3
isotig36126	1.566	2.107	2.988	2.555	587	0.060757496	isogroup21217	ENSG00000070669	ENST00000454046	ASNS

isotig36128	1.532	2.172	3.806	3.242	587	0.074481476	isogroup21219	ENSG00000177679	ENST00000388802	SRRM3
isotig36129	1.055	1.46	3.051	1.336	587	0.361050575	isogroup21220	ENSG00000244045	ENST00000509083	TMEM199
isotig36134	225.304	253.097	191.611	160.145	586	0.264409709	isogroup21225	ENSG00000247596	ENST00000499914	TWF2
isotig36138	1.5	2.761	2.741	2.828	586	0.227483655	isogroup21229	ENSG00000185033	ENST00000559074	SEMA4B
isotig36146	1.056	1.819	1.985	1.986	586	0.253391072	isogroup21237	ENSG00000142494	ENST00000457293	SLC47A1
isotig36148	2.249	2.208	2.49	1.91	586	0.136638611	isogroup21239	ENSG00000165632	ENST00000344293	TAF3
isotig36150	1.534	3.541	5.104	4.012	586	0.106607425	isogroup21241	ENSG00000261150	ENST00000568225	EPPK1
isotig36153	1.295	1.527	2.111	1.834	586	0.029392425	isogroup21244	ENSG00000115425	ENST00000265322	PECR
isotig36162	1.537	2.017	2.304	2.45	585	0.183258811	isogroup21253	ENSG00000169738	ENST00000306869	DCXR
isotig36165	2.249	1.527	1.166	1.07	586	0.28376982	isogroup21256	ENSG00000099364	ENST00000567912	FBXL19
isotig36169	3.311	2.798	1.61	2.027	585	0.20724055	isogroup21260	ENSG00000109686	ENST00000514152	SH3D19
isotig36178	2.868	1.887	2.904	1.263	585	0.501465394	isogroup21269	ENSG00000124177	ENST00000373233	CHD6
isotig36185	1.195	1.887	3.219	3.099	585	0.133181784	isogroup21276	ENSG00000213923	ENST00000431611	CSNK1E
isotig36186	1.742	1.952	2.651	2.144	585	0.074039979	isogroup21277	ENSG00000188827	ENST00000294008	SLX4
isotig36187	3.488	3.683	4.521	5.289	584	0.110299091	isogroup21278	ENSG00000185163	ENST00000397333	DDX51
isotig36192	41.187	31.206	73.539	35.151	577	0.801279402	isogroup21283	ENSG00000163191	ENST00000271638	S100A11
isotig36196	1.368	1.858	3.099	2.721	584	0.016354174	isogroup21287	ENSG00000136573	ENST00000526097	BLK
isotig36201	1.539	2.901	3.288	3.603	584	0.17064327	isogroup21292	ENSG00000100445	ENST00000399395	SDR39U1
isotig36202	2.736	3.226	2.909	2.721	584	0.097401743	isogroup21293	ENSG00000143537	ENST00000526491	ADAM15
isotig36204	3.248	3.878	1.801	1.188	584	0.142387465	isogroup21295	ENSG00000206181	ENST00000332567	TCEB3B
isotig36205	3.009	1.368	3.13	1.457	584	0.663973848	isogroup21296	ENSG00000112659	ENST00000372647	CUL9
isotig36212	85.937	65.935	106.741	118.565	584	0.106194108	isogroup21303	ENSG00000147274	ENST00000562646	RBMX
isotig36213	1.777	2.705	1.991	2.031	584	0.111369956	isogroup21304	ENSG00000108469	ENST00000443199	RECQL5
isotig36226	1.777	1.14	1.676	1.38	584	0.230001127	isogroup21317	ENSG00000013810	ENST00000313288	TACC3
isotig36229	6.497	7.432	9.517	8.471	584	0.078708574	isogroup21320	ENSG00000076201	ENST00000456408	PTPN23
isotig36232	1.576	1.338	1.298	1.383	583	0.080145788	isogroup21323	ENSG00000129219	ENST00000263088	PLD2
isotig36234	1.027	1.338	2.566	1.652	583	0.090788683	isogroup21325	ENSG00000100599	ENST00000555589	RIN3
isotig36587	4.002	2.885	2.991	1.873	574	0.509637785	isogroup21678	ENSG00000215612	ENST00000400677	HMX1
isotig36591	1.497	2.023	2.991	2.261	574	0.021652138	isogroup21682	ENSG00000175600	ENST00000540834	C7orf10
isotig36594	1.392	2.786	3.892	2.73	574	0.006660029	isogroup21685	ENSG00000198026	ENST00000243961	ZNF335
isotig36597	2.859	2.525	1.805	1.368	573	0.119720824	isogroup21688	ENSG00000134987	ENST00000513710	WDR36
isotig36598	1.639	2.325	1.579	1.485	573	0.292477643	isogroup21689	ENSG00000105875	ENST00000423565	WDR91
isotig36602	5.054	2.426	2.74	4.024	573	0.259562636	isogroup21693	ENSG00000198740	ENST00000430262	ZNF652
isotig36603	3.45	2.923	2.03	4.611	573	0.459504396	isogroup21694	ENSG00000145362	ENST00000357077	ANK2
isotig36604	1.115	2.192	2.867	1.757	573	0.106814083	isogroup21695	ENSG00000129749	ENST00000534359	CHRNA10
isotig36605	2.754	5.482	2.771	2.07	573	0.609406703	isogroup21696	ENSG00000131435	ENST00000379018	PDLIM4
isotig36609	1.812	2.325	3.255	3.476	573	0.094423987	isogroup21700	ENSG00000128564	ENST00000445482	VGF
isotig36615	1.499	3.188	5.028	3.009	573	0.061837755	isogroup21706	ENSG00000115935	ENST00000392548	WIPF1
isotig36619	1.115	2.391	4.188	2.579	573	0.164330803	isogroup21710	ENSG00000186648	ENST00000342740	LRRC16B
isotig36630	4.958	1.164	3.551	1.722	572	0.811518374	isogroup21721	ENSG00000221938	ENST00000408899	OR2A14
isotig36631	4.503	4.026	1.969	1.096	572	0.289622003	isogroup21722	ENSG00000110048	ENST00000378235	OSBP
isotig36637	4.329	4.126	4.649	3.719	572	0.264841813	isogroup21728	ENSG00000122678	ENST00000414235	POLM
isotig36640	1.257	1.93	1.549	1.918	572	0.239883144	isogroup21731	ENSG00000188039	ENST00000549814	NWD1
isotig36650	24.902	14.235	21.278	13.487	571	0.744983843	isogroup21741	ENSG00000109265	ENST00000541073	KIAA1211
isotig36651	3.142	2.163	1.872	1.956	572	0.214623882	isogroup21742	ENSG00000149582	ENST00000533102	TMEM25
isotig36654	1.327	1.664	2.26	2.231	572	0.047521981	isogroup21745	ENSG00000205978	ENST00000382554	NYNRIN
isotig36660	5.028	3.428	1.42	2.427	572	0.33554708	isogroup21751	ENSG00000154265	ENST00000392676	ABCA5
isotig36661	1.99	3.061	2.97	4.306	572	0.4053036	isogroup21752	ENSG00000147576	ENST00000415254	ADHFE1
isotig36662	3.077	2.635	3.615	2.622	572	0.309113624	isogroup21753	ENSG00000140332	ENST00000559929	TLE3
isotig37087	1.036	1.873	3.27	2.363	559	0.074039979	isogroup22178	ENSG00000227402	ENST00000456261	SLC39A7
isotig37093	3.751	2.725	3.138	2.444	559	0.353939656	isogroup22184	ENSG00000005187	ENST00000562251	ACSM3
isotig37097	1.537	2.417	3.27	2.723	559	0.224064402	isogroup22188	ENSG00000259938	ENST00000568052	CUX1.1
isotig37101	1.878	2.525	2.366	1.726	558	0.094264297	isogroup22192	ENSG00000154175	ENST00000497395	ABI3BP
isotig37112	1.857	3.508	4.757	5.687	559	0.407548659	isogroup22203	ENSG00000115762	ENST00000404460	PLEKHB2
isotig37113	1.107	2.145	2.181	2.964	559	0.376540543	isogroup22204	ENSG00000184956	ENST00000421673	MUC6
isotig37119	1.857	2.145	2.973	2.403	559	0.095447885	isogroup22210	ENSG00000008056	ENST00000340666	SYN1
isotig37120	1.36	2.183	3.308	2.487	558	0.136638611	isogroup22211	ENSG00000153902	ENST00000392225	LGI4
isotig37123	3.292	6.208	8.14	7.502	558	0.112807169	isogroup22214	ENSG00000091542	ENST00000261650	ALKBH5
isotig37132	2.9	4.776	6.22	6.419	558	0.251822349	isogroup22223	ENSG00000133115	ENST00000423210	STOML3
isotig37133	3.83	1.194	2.681	1.324	558	0.704272188	isogroup22224	ENSG00000014919	ENST00000370483	COX15
isotig37142	3.113	1.023	1.952	1.364	558	0.55762005	isogroup22233	ENSG00000111676	ENST00000544325	ATN1
isotig37149	31.53	22.104	35.704	23.029	558	0.671592019	isogroup22240	ENSG00000185739	ENST00000330063	SRL
isotig37150	3.4	2.695	3.409	2.167	558	0.545755993	isogroup22241	ENSG00000135093	ENST00000392784	USP30
isotig37152	2.685	2.525	3.011	2.808	558	0.145271286	isogroup22243	ENSG00000101191	ENST00000395343	DIDO1
isotig37156	6.407	5.595	5.989	4.855	558	0.240390396	isogroup22247	ENSG00000116809	ENST00000537142	ZBTB17
isotig37158	4.832	4.4	11.548	5.978	558	0.610148794	isogroup22249	ENSG00000122574	ENST00000409290	WIPF3
isotig37159	1.396	2.695	3.342	3.33	558	0.298564665	isogroup22250	ENSG00000179044	ENST00000563889	EXOC3L1
isotig37160	174.464	294.078	192.013	183.701	549	0.536738183	isogroup22251	ENSG00000090266	ENST00000465506	NDUFB2
isotig37161	20.364	16.237	28.459	29.127	558	0.437316826	isogroup22252	ENSG00000154035	ENST00000399011	C17orf103
isotig37163	3.007	3.991	1.059	1.405	558	0.298583452	isogroup22254	ENSG00000058272	ENST00000547330	PPP1R12A
isotig37168	3.013	3.075	1.591	1.165	557	0.039415345	isogroup22259	ENSG00000115020	ENST00000264380	PIKFYVE
isotig37180	1.255	3.178	2.221	2.372	557	0.52446081	isogroup22271	ENSG00000145861	ENST00000393975	C1QTNF2
isotig37181	1.473	2.637	2.824	2.698	556	0.221302698	isogroup22272	ENSG00000163933	ENST00000394738	RFT1
isotig37184	2.366	2.05	1.293	2.372	557	0.192051176	isogroup22275	ENSG00000159516	ENST00000368748	SPRR2G
isotig37190	1.327	1.914	2.851	1.849	557	0.135248366	isogroup22281	ENSG00000034533	ENST00000264992	ASTE1
isotig37191	44.926	19.684	38.786	45.537	557	0.502122943	isogroup22282	ENSG00000173442	ENST00000533237	EHBP1L1
isotig37203	1.183	1.811	3.25	2.411	557	0.091699857	isogroup22294	ENSG00000239886	ENST00000377721	KRTAP9-2
isotig37204	7.817	3.485	3.215	3.375	557	0.63629105	isogroup22295	ENSG00000205899	ENST00000391429	BHLHA9
isotig37208	1.434	2.323	2.519	2.774	557	0.258472984	isogroup22299	ENSG00000144021	ENST00000488633	CIAO1
isotig37209	3.048	2.905	2.189	1.729	557	0.247153754	isogroup22300	ENSG00000160013	ENST00000291294	PTGIR
isotig37224	1.077	1.883	2.856	3.059	556	0.291998572	isogroup22315	ENSG00000139800	ENST00000267294	ZIC5
isotig37228	2.91	2.396	1.761	1.45	556	0.077975877	isogroup22319	ENSG00000164151	ENST00000296564	KIAA0947

isotig37230	4.777	5.032	6.376	7.248	556	0.085274667	isogroup22321	ENSG00000120709	ENST00000434981	FAM53C
isotig37232	2.011	2.43	3.155	3.101	556	0.043172766	isogroup22323	ENSG00000204873	ENST00000411528	KRTAP9-3
isotig37239	1.221	2.156	2.656	2.737	556	0.039415345	isogroup22330	ENSG00000124006	ENST00000456147	OBSL1
isotig37240	1.903	1.369	1.761	1.811	556	0.108307658	isogroup22331	ENSG00000129925	ENST00000431232	TMEM8A
isotig37241	2.694	1.849	2.558	1.45	556	0.461805816	isogroup22332	ENSG00000115266	ENST00000535453	APC2
isotig37242	1.365	1.437	2.158	1.732	556	0.059301495	isogroup22333	ENSG00000126461	ENST00000360565	SCAF1
isotig37589	14.577	10.004	15.668	19.078	548	0.299804614	isogroup22680	ENSG00000165238	ENST00000427277	WNK2
isotig37597	3.651	2.993	1.013	1.841	547	0.025447133	isogroup22688	ENSG00000170677	ENST00000397942	SOCS6
isotig37600	1.863	1.984	2.497	1.965	547	0.039415345	isogroup22691	ENSG00000173085	ENST00000439031	COQ2
isotig37602	2.446	2.993	2.161	2.782	547	0.329741865	isogroup22693	ENSG00000119688	ENST00000356924	ABCD4
isotig38092	1.711	1.737	3.438	2.877	537	0.204178252	isogroup23183	ENSG00000162571	ENST00000379289	TTLL10
isotig38094	3.607	7.019	3.507	4.46	537	0.566572105	isogroup23185	ENSG00000203883	ENST00000340356	SOX18
isotig38098	4.054	2.445	3.404	2.084	537	0.572612159	isogroup23189	ENSG00000225697	ENST00000447978	SLC26A6
isotig38099	2.976	5.352	3.233	4.127	537	0.586674307	isogroup23190	ENSG00000159873	ENST00000249064	CCDC117
isotig38102	4.017	3.048	2.855	2.417	537	0.21810889	isogroup23193	ENSG00000198887	ENST00000361138	SMC5
isotig38104	1.823	1.311	1.341	1.542	537	0.092620425	isogroup23195	ENSG00000108821	ENST00000225964	COL1A1
isotig38106	4.861	3.386	3.596	1.76	534	0.576839258	isogroup23197	ENSG00000053747	ENST00000399516	LAMA3
isotig38115	1.637	2.942	4.022	4.086	537	0.099938003	isogroup23206	ENSG00000205726	ENST00000437126	ITSN1
isotig38117	1.525	3.19	3.68	3.544	537	0.244758398	isogroup23208	ENSG00000182199	ENST00000553837	SHMT2
isotig38118	2.389	2.669	2.417	2.845	535	0.318178402	isogroup23209	ENSG00000182774	ENST00000330244	RPS17L
isotig38125	1.004	2.48	2.786	2.084	537	0.201961374	isogroup23216	ENSG00000119927	ENST00000423155	GPAM
isotig38130	1.192	1.67	1.791	2.131	536	0.221950853	isogroup23221	ENSG00000114857	ENST00000232978	NKTR
isotig38132	2.72	2.166	1.998	2.047	536	0.233166754	isogroup23223	ENSG00000196159	ENST00000394329	FAT4
isotig38136	1.267	3.302	2.791	2.549	536	0.466915909	isogroup23227	ENSG00000183779	ENST00000331569	ZNF703
isotig38142	2.536	2.262	2.237	1.757	535	0.230433231	isogroup23233	ENSG00000213121	ENST00000392385	AL590867.1
isotig38145	2.757	2.841	3.065	2.965	536	0.074481476	isogroup23236	ENSG00000049246	ENST00000377532	PER3
isotig38150	2.347	2.06	2.412	2.006	536	0.233166754	isogroup23241	ENSG00000180263	ENST00000546711	FGD6
isotig38161	2.086	1.918	1.379	1.463	536	0.022976629	isogroup23252	ENSG00000001629	ENST00000265742	ANKIB1
isotig38165	3.838	3.231	2.687	2.131	536	0.242870294	isogroup23256	ENSG00000115286	ENST00000435801	NDUFS7
isotig38171	10.229	14.891	14.341	12.726	533	0.376052078	isogroup23262	ENSG00000124357	ENST00000455662	NAGK
isotig38172	2.576	2.705	1.45	2.217	535	0.473801383	isogroup23263	ENSG00000109686	ENST00000514152	SH3D19
isotig38176	5.897	5.372	6.039	6.486	535	0.176298189	isogroup23267	ENSG00000099910	ENST00000444967	KLHL22
isotig38177	1.269	1.85	2.209	2.553	535	0.288203577	isogroup23268	ENSG00000102606	ENST00000545635	ARHGEF7
isotig38180	1.12	2.312	2.484	2.804	535	0.297446832	isogroup23271	ENSG00000113643	ENST00000538719	RARS
isotig38181	1.12	1.458	2.209	2.05	535	0.041209514	isogroup23272	ENSG00000151093	ENST00000448177	OXSM
isotig38183	2.998	1.285	2.426	1.133	533	0.587726385	isogroup23274	ENSG00000187800	ENST00000338302	PEAR1
isotig38186	1.867	1.351	1.864	2.553	535	0.021652138	isogroup23277	ENSG00000163013	ENST00000521871	FBXO41
isotig38188	1.157	1.886	2.312	2.26	535	0.021652138	isogroup23279	ENSG00000076201	ENST00000456408	PTPN23
isotig38189	3.359	2.989	1.622	3.013	535	0.17064327	isogroup23280	ENSG00000185933	ENST00000329905	CALHM1
isotig38190	1.344	2.312	1.207	1.966	535	0.469555497	isogroup23281	ENSG00000120008	ENST00000263461	WDR11
isotig38195	849.289	545.338	630.248	499.242	532	0.665739836	isogroup23286	ENSG00000105372	ENST00000221975	RPS19
isotig38198	3.321	3.06	2.209	2.176	535	0.046479297	isogroup23289	ENSG00000160305	ENST00000427143	DIP2A
isotig38205	3.024	3.308	2.105	2.135	535	0.125807845	isogroup23296	ENSG00000162341	ENST00000539166	TPCN2
isotig38206	1.68	2.241	2.622	3.976	535	0.332278124	isogroup23297	ENSG00000171467	ENST00000361428	ZNF318
isotig38214	3.105	1.64	1.556	1.635	534	0.388479748	isogroup23305	ENSG00000261443	ENST00000565618	RAD54L2.1
isotig38587	1.554	2.095	3.154	3.567	527	0.136507102	isogroup23678	ENSG00000105464	ENST00000263269	GRIN2D
isotig38590	2.994	1.552	1.262	1.615	527	0.364122266	isogroup23681	ENSG00000101493	ENST00000443185	ZNF516
isotig38610	1.592	2.167	2.522	2.719	527	0.240164951	isogroup23701	ENSG00000169093	ENST00000416733	ASMTL
isotig38612	4.888	3.937	2.909	3.951	527	0.155266025	isogroup23703	ENSG00000091157	ENST00000444065	WDR7
isotig38616	1.137	1.59	1.366	2.38	527	0.313979484	isogroup23707	ENSG00000140464	ENST00000565239	PML
isotig38624	2.463	5.057	6.201	3.909	527	0.126258736	isogroup23715	ENSG00000175567	ENST00000536983	UCP2
isotig38629	3.865	2.818	2.173	1.615	527	0.323842714	isogroup23720	ENSG00000091157	ENST00000444065	WDR7
isotig38631	1.516	2.782	3.258	3.398	527	0.20724055	isogroup23722	ENSG00000164099	ENST00000296498	PRSS12
isotig38636	1.671	2.679	3.65	3.745	526	0.071109191	isogroup23727	ENSG00000145794	ENST00000503335	MEGF10
isotig38639	1.981	2.87	1.727	1.239	524	0.384835049	isogroup23730	ENSG00000152952	ENST00000494950	PLOD2
isotig38640	4.139	2.932	4.106	3.192	526	0.392631698	isogroup23731	ENSG00000116574	ENST00000366691	RHOU
isotig38644	3.416	2.496	2.458	2.213	526	0.229813256	isogroup23735	ENSG00000049246	ENST00000539773	PER3
isotig38653	4.669	4.667	2.141	1.106	526	0.143270459	isogroup23744	ENSG00000151692	ENST00000320892	RNF144A
isotig38656	77.606	108.958	99.73	76.439	526	0.074162095	isogroup23747	ENSG00000154723	ENST00000457143	ATP5J
isotig38658	2.506	1.266	2.527	2.127	526	0.366310964	isogroup23749	ENSG00000126243	ENST00000246529	LRFN3
isotig38662	2.81	2.46	1.791	1.234	526	0.205991208	isogroup23753	ENSG00000101911	ENST00000380668	PRPS2
isotig38664	4.859	3.04	3.827	4.213	526	0.291923424	isogroup23755	ENSG00000130338	ENST00000367097	TULP4
isotig38674	6.074	2.388	4.458	1.574	526	0.840046592	isogroup23765	ENSG00000169026	ENST00000515118	MFSD7
isotig38686	3.423	5.728	1.547	1.876	525	0.371261366	isogroup23777	ENSG00000125503	ENST00000435544	PPP1R12C
isotig38687	2.701	2.537	2.392	1.705	525	0.091530773	isogroup23778	ENSG00000125630	ENST00000541869	POLR1B
isotig38688	1.903	2.356	2.709	1.663	525	0.084617119	isogroup23779	ENSG00000167925	ENST00000428494	GHDC
isotig38711	3.537	2.684	1.794	1.45	525	0.386215901	isogroup23802	ENSG00000153922	ENST00000284049	CHD1
isotig38712	1.598	2.61	2.884	3.581	525	0.197396107	isogroup23803	ENSG00000182272	ENST00000329962	B4GALNT4
isotig38713	1.903	2.139	2.813	3.027	525	0.054116255	isogroup23804	ENSG00000225828	ENST00000432622	RP4-811H24.6.1
isotig38726	103.735	97.274	131.189	114.577	525	0.350708274	isogroup23817	ENSG00000178952	ENST00000313511	TUFM
isotig38735	1.293	3.154	2.884	2.472	525	0.306915533	isogroup23826	ENSG00000111785	ENST00000552619	RIC8B
isotig38736	4.261	1.088	1.758	1.705	525	0.686743819	isogroup23827	ENSG00000197535	ENST00000553916	MYO5A
isotig38740	1.941	2.829	2.709	2.558	525	0.077975877	isogroup23831	ENSG00000070047	ENST00000534320	PHRF1
isotig38741	4.299	2.756	5.381	3.112	525	0.503654092	isogroup23832	ENSG00000164880	ENST00000389470	INTS1
isotig38742	6.314	2.356	3.834	1.621	525	0.816572105	isogroup23833	ENSG00000138190	ENST00000443748	EXOC6
isotig39090	1.539	1.466	1.958	2.2	519	0.415993462	isogroup24181	ENSG00000188993	ENST00000343457	LRRC66
isotig39098	1.311	1.432	2.496	2.118	518	0.065266401	isogroup24189	ENSG00000104897	ENST00000221494	SF3A2
isotig39102	1.311	2.241	1.961	2.376	518	0.310832644	isogroup24193	ENSG00000197019	ENST00000357949	SERTAD1
isotig39106	1.929	1.579	1.427	1.34	518	0.141805065	isogroup24197	ENSG00000147854	ENST00000276893	UHRF2
isotig39111	2.351	1.506	2.459	2.637	518	0.129649808	isogroup24202	ENSG00000080603	ENST00000395059	SRCAP
isotig39119	1.851	3.454	3.6	3.544	518	0.228150597	isogroup24210	ENSG00000138035	ENST00000447944	PNPT1
isotig39123	3.431	2.645	3.849	2.766	518	0.353864507	isogroup24214	ENSG00000158985	ENST00000505065	CDC42SE2

isotig39124	244.817	372.969	217.959	220.583	518	0.568713835	isogroup24215	ENSG00000109846	ENST00000531198	CRYAB
isotig39588	1.838	2.832	1.554	2.454	511	0.577600135	isogroup24679	ENSG00000123191	ENST00000542656	ATP7B
isotig39594	5.454	4.004	4.815	4.232	509	0.366527016	isogroup24685	ENSG00000151164	ENST00000358071	RAD9B
isotig39609	1.292	2.649	2.969	3.073	510	0.263057038	isogroup24700	ENSG00000167548	ENST00000301067	MLL2
isotig39610	1.606	2.388	1.737	1.931	510	0.367306681	isogroup24701	ENSG00000135829	ENST00000399175	DHX9
isotig39612	1.88	2.8	1.592	1.404	510	0.278697302	isogroup24703	ENSG00000112685	ENST00000230449	EXOC2
isotig39619	1.884	2.654	1.595	1.583	509	0.205822124	isogroup24710	ENSG00000162600	ENST00000456980	OMA1
isotig39622	1.763	2.09	2.535	2.722	510	0.014428496	isogroup24713	ENSG00000120055	ENST00000239125	C10orf95
isotig39624	1.841	2.425	2.716	2.984	510	0.227521229	isogroup24715	ENSG00000090376	ENST00000457197	IRAK3
isotig39627	1.371	2.463	2.824	2.59	510	0.112769595	isogroup24718	ENSG00000188155	ENST00000400368	KRTAP10-6
isotig39632	1.426	1.081	1.523	1.183	507	0.197001578	isogroup24723	ENSG00000188554	ENST00000542611	NBR1
isotig39636	15.459	16.569	13.078	12.116	501	0.06909897	isogroup24727	ENSG00000196333	ENST00000358740	AL158821.1
isotig39642	2.153	2.09	1.882	1.888	510	0.045840535	isogroup24733	ENSG00000102967	ENST00000219240	DHODH
isotig39648	1.567	1.867	2.353	1.449	510	0.028237018	isogroup24739	ENSG00000129467	ENST00000554781	ADCY4
isotig39656	1.413	1.908	1.415	2.155	509	0.334391674	isogroup24747	ENSG00000197070	ENST00000371421	ARRDC1
isotig39660	1454.464	1997.811	1523.487	1203.851	509	0.354888405	isogroup24751	ENSG00000198938	ENST00000362079	MT-CO3
isotig39672	1.609	1.57	1.415	1.363	509	0.134825656	isogroup24763	ENSG00000143363	ENST00000368936	PRUNE
isotig39673	1.491	2.43	2.866	3.079	509	0.167487037	isogroup24764	ENSG00000112077	ENST00000418071	RHAG
isotig39676	5.847	4.039	4.389	3.342	509	0.49656196	isogroup24767	ENSG00000131435	ENST00000418373	PDLIM4
isotig39677	4.316	3.589	1.56	2.33	509	0.038353874	isogroup24768	ENSG00000136731	ENST00000375990	UGGT1
isotig39679	1.884	2.43	1.705	2.859	509	0.414227474	isogroup24770	ENSG00000185033	ENST00000411539	SEMA4B
isotig39686	1.845	1.982	1.051	1.232	509	0.20052416	isogroup24777	ENSG00000162607	ENST00000371146	USP1
isotig39694	1.059	1.684	2.576	3.387	509	0.304491997	isogroup24785	ENSG00000197496	ENST00000359271	SLC2A10
isotig39695	2.747	3.104	2.974	1.978	509	0.20052416	isogroup24786	ENSG00000165055	ENST00000262432	METTL2B
isotig39699	14.008	10.957	20.352	9.587	509	0.781637484	isogroup24790	ENSG00000196872	ENST00000397899	C2orf55
isotig39700	2.668	1.684	3.411	2.155	509	0.599928609	isogroup24791	ENSG00000172830	ENST00000308127	SSH3
isotig39713	3.373	2.88	1.851	1.363	509	0.014428496	isogroup24804	ENSG00000133858	ENST00000552994	ZFC3H1
isotig39719	15.646	3.86	3.598	2.6	508	0.908037123	isogroup24810	ENSG00000134013	ENST00000524144	LOXL2
isotig39733	9.317	6.183	12.394	6.654	508	0.673968588	isogroup24824	ENSG00000183779	ENST00000331569	ZNF703
isotig39736	3.067	3.484	2.581	1.587	508	0.269331931	isogroup24827	ENSG00000109436	ENST00000442267	TBC1D9
isotig39739	2.122	2.098	1.744	1.498	508	0.106814083	isogroup24830	ENSG00000156650	ENST00000287239	KAT6B
isotig39741	1.73	1.199	1.49	1.323	508	0.066496956	isogroup24832	ENSG00000196576	ENST00000399964	PLXNB2
isotig39757	4.404	1.724	3.272	2.689	508	0.604071166	isogroup24848	ENSG00000126464	ENST00000418929	PRR12
isotig39760	1.337	1.949	1.126	1.057	508	0.155312993	isogroup24851	ENSG00000129493	ENST00000543095	HEATR5A
isotig39762	1.73	1.874	2.289	2.732	508	0.06751146	isogroup24853	ENSG00000119596	ENST00000549293	YLPM1
isotig39766	4.364	1.911	3.344	3.348	508	0.58782032	isogroup24857	ENSG00000163795	ENST00000407879	ZNF513
isotig39770	1.455	2.659	3.018	2.909	508	0.106053205	isogroup24861	ENSG00000110446	ENST00000442626	SLC15A3
isotig39774	2.24	2.585	2.254	1.762	508	0.088308785	isogroup24865	ENSG00000168542	ENST00000304636	COL3A1
isotig40091	1.231	1.704	2.606	2.048	503	0.016354174	isogroup25182	ENSG00000189001	ENST00000452271	SBSN
isotig40092	1.072	2.346	2.165	2.002	503	0.363915608	isogroup25183	ENSG00000227804	ENST00000449113	PPP1R10
isotig40093	5.201	3.899	3.01	3.115	503	0.189796724	isogroup25184	ENSG00000165494	ENST00000530660	PCF11
isotig40098	6.484	3.829	1.95	1.829	502	0.372294657	isogroup25189	ENSG00000176105	ENST00000359834	YES1
isotig40100	2.262	1.211	2.642	1.602	503	0.500695123	isogroup25191	ENSG00000164076	ENST00000487726	CAMKV
isotig40110	2.183	2.082	1.431	2.27	503	0.267650485	isogroup25201	ENSG00000173064	ENST00000550968	C12orf51
isotig40116	4.098	6.9	1.729	2.185	502	0.697236417	isogroup25207	ENSG00000112769	ENST00000522006	LAMA4
isotig40117	2.984	1.972	1.839	2.096	502	0.240164951	isogroup25208	ENSG00000112320	ENST00000317357	SOBP
isotig40119	6.524	5.309	4.966	3.657	502	0.441111821	isogroup25210	ENSG00000130699	ENST00000252996	TAF4
isotig40126	1.631	1.213	2.207	1.783	502	0.089576914	isogroup25217	ENSG00000171475	ENST00000323571	WIPF2
isotig40595	1.167	1.881	2.569	3.024	496	0.219226723	isogroup25686	ENSG00000111834	ENST00000368581	RSPH4A
isotig40610	1.574	2.653	3.022	2.442	495	0.043172766	isogroup25701	ENSG00000083123	ENST00000541767	BCKDHB
isotig40615	1.129	2.345	2.648	3.301	495	0.346941459	isogroup25706	ENSG00000179168	ENST00000334928	GGN
isotig40618	1.21	1.576	2.238	2.034	495	0.065266401	isogroup25709	ENSG00000257743	ENST00000477922	RP11-1220K2.2.1
isotig40638	10.448	18.265	7.76	7.689	495	0.812814684	isogroup25729	ENSG00000142798	ENST00000374695	HSPG2
isotig40649	1.291	2.962	3.693	3.754	495	0.245829263	isogroup25740	ENSG00000167186	ENST00000569127	COQ7
isotig40661	1.129	1.923	1.493	2.081	495	0.416867062	isogroup25752	ENSG00000205047	ENST00000446344	AC010536.1
isotig40667	2.501	2.808	2.574	3.257	495	0.316844518	isogroup25758	ENSG00000198720	ENST00000488766	ANKRD13B
isotig40673	1.089	2.885	2.499	3.527	495	0.484087322	isogroup25764	ENSG00000162878	ENST00000401498	PKDCC
isotig40684	2.628	1.579	2.168	2.492	494	0.236510859	isogroup25775	ENSG00000136295	ENST00000429448	TTYH3
isotig40686	3.113	4.354	2.729	2.628	494	0.208020215	isogroup25777	ENSG00000131584	ENST00000353662	ACAP3
isotig40687	1.294	1.194	1.383	1.314	494	0.106053205	isogroup25778	ENSG00000087338	ENST00000282570	GMCL1
isotig40693	1.901	1.889	1.233	1.223	494	0.016354174	isogroup25784	ENSG00000125247	ENST00000342624	TMTC4
isotig40700	1.621	6.022	1.46	1.726	493	0.700270534	isogroup25791	ENSG00000069667	ENST00000261523	RORA
isotig40708	1.496	1.579	2.579	2.401	494	0.065266401	isogroup25799	ENSG00000167978	ENST00000426305	SRRM2
isotig40717	3.194	3.66	3.14	2.719	494	0.09210378	isogroup25808	ENSG00000085741	ENST00000322563	WNT11
isotig40719	1.514	1.99	1.74	1.834	488	0.10352634	isogroup25810	ENSG00000229809	ENST00000567855	ZNF688
isotig40720	2.022	2.312	2.204	2.901	494	0.129649808	isogroup25811	ENSG00000206384	ENST00000453409	COL6A6
isotig40727	3.921	4.162	4.597	4.123	494	0.259468701	isogroup25818	ENSG00000161249	ENST00000429837	DMKN
isotig40731	3.881	2.619	3.103	3.263	494	0.304557752	isogroup25822	ENSG00000084693	ENST00000487078	AGBL5
isotig40734	2.83	1.425	2.393	1.36	494	0.464501766	isogroup25825	ENSG00000104728	ENST00000518288	ARHGEF10
isotig40749	2.063	1.04	1.682	1.676	494	0.307760953	isogroup25840	ENSG00000106479	ENST00000223210	ZNF862
isotig40752	5.753	4.826	2.884	5.176	493	0.178017209	isogroup25843	ENSG00000168781	ENST00000453080	PPIP5K1
isotig40754	87.986	67.951	63.596	90.546	493	0.680778913	isogroup25845	ENSG00000154358	ENST00000570156	OBSCN
isotig40763	5.631	4.016	7.416	5.994	493	0.388066431	isogroup25854	ENSG00000198917	ENST00000372618	C9orf114
isotig40764	2.998	2.702	2.996	3.178	493	0.089605095	isogroup25855	ENSG00000168542	ENST00000317840	COL3A1
isotig40765	1.79	3.102	1.843	1.642	491	0.078182536	isogroup25856	ENSG00000196333	ENST00000358740	AL158821.1
isotig40766	1.134	1.7	1.836	1.544	493	0.029392425	isogroup25857	ENSG00000116984	ENST00000417743	MTR
isotig40768	4.941	2.897	4.345	2.907	493	0.62029383	isogroup25859	ENSG00000156218	ENST00000567476	ADAMTSL3
isotig40772	21.915	26.564	30.937	20.617	493	0.238408357	isogroup25863	ENSG00000157881	ENST00000435556	PANK4
isotig40774	5.266	3.166	2.434	2.316	493	0.360486962	isogroup25865	ENSG00000163788	ENST00000454177	SNRK
isotig40780	2.268	2.201	2.061	2.134	493	0.034718569	isogroup25871	ENSG00000106344	ENST00000478061	RBM28
isotig40784	2.911	3.03	3.191	2.079	493	0.312241677	isogroup25875	ENSG00000205653	ENST00000381078	AL035696.1
isotig40787	10.856	12.162	7.229	7.266	493	0.078708574	isogroup25878	ENSG00000178950	ENST00000511163	GAK

isotig40802	1.053	2.008	2.473	2.497	493	0.2102277	isogroup25893	ENSG00000126001	ENST00000422671	CEP250
isotig41089	1.266	1.753	1.624	1.877	489	0.170981438	isogroup26180	ENSG00000152518	ENST00000282388	ZFP36L2
isotig41092	4.861	4.593	5.098	3.708	489	0.315491846	isogroup26183	ENSG00000068024	ENST00000345617	HDAC4
isotig41093	1.061	2.763	2.605	2.701	489	0.369570527	isogroup26184	ENSG00000188155	ENST00000400368	KRTAP10-6
isotig41103	1.391	3.043	3.633	3.166	488	0.173724356	isogroup26194	ENSG00000180438	ENST00000532924	TPRXL
isotig41104	2.578	1.756	1.855	1.33	488	0.361632975	isogroup26195	ENSG00000138617	ENST00000261888	PARP16
isotig41107	22.59	32.256	45.027	45.232	488	0.28675697	isogroup26198	ENSG00000197587	ENST00000371956	DMBX1
isotig41112	5.77	2.496	4.578	3.807	488	0.632110919	isogroup26203	ENSG00000139219	ENST00000395281	COL2A1
isotig41113	2.578	3.861	2.383	3.256	488	0.467244683	isogroup26204	ENSG00000164597	ENST00000393603	COG5
isotig41118	6.793	5.422	5.486	4.451	488	0.448326069	isogroup26209	ENSG00000167978	ENST00000426305	SRRM2
isotig41122	1.678	2.107	2.119	1.652	488	0.069775306	isogroup26213	ENSG00000186635	ENST00000455638	ARAP1
isotig41129	1.023	1.404	2.422	2.57	488	0.095898775	isogroup26220	ENSG00000111605	ENST00000456847	CPSF6
isotig41131	4.174	2.807	3.141	1.652	488	0.572076727	isogroup26222	ENSG00000165118	ENST00000376344	C9orf64
isotig41132	1.268	2.223	2.156	2.111	488	0.313171639	isogroup26223	ENSG00000120694	ENST00000438061	HSPH1
isotig41140	4.748	3.432	2.838	1.881	488	0.351187345	isogroup26231	ENSG00000152520	ENST00000399613	PAN3
isotig41142	1.473	1.598	5.221	3.211	488	0.517021117	isogroup26233	ENSG00000133226	ENST00000447431	SRRM1
isotig41146	2.537	2.184	1.475	2.844	488	0.26200496	isogroup26237	ENSG00000178919	ENST00000375123	FOXE1
isotig41162	1.145	1.676	2.762	2.203	488	0.003503795	isogroup26253	ENSG00000104687	ENST00000546342	GSR
isotig41166	2.701	2.691	3.141	2.982	488	0.173724356	isogroup26257	ENSG00000196428	ENST00000361875	TSC22D2
isotig41590	12.1	2.606	5.593	2.647	482	0.907295033	isogroup26681	ENSG00000172037	ENST00000418109	LAMB2
isotig41594	1.533	3.396	4.023	3.529	482	0.181304952	isogroup26685	ENSG00000130299	ENST00000361619	GTPBP3
isotig41603	252.95	198.162	216.635	208.17	482	0.309827534	isogroup26694	ENSG00000101605	ENST00000400569	MYOM1
isotig41608	1.741	1.618	2.068	2.602	482	0.028237018	isogroup26699	ENSG00000154655	ENST00000535782	L3MBTL4
isotig41615	4.235	4.035	3.339	2.653	481	0.279608477	isogroup26706	ENSG00000198324	ENST00000450786	FAM109A
isotig41617	1.204	2.058	2.688	2.746	481	0.242729391	isogroup26708	ENSG00000138814	ENST00000523694	PPP3CA
isotig41620	1.245	1.464	1.688	2.326	481	0.216690464	isogroup26711	ENSG00000198324	ENST00000450786	FAM109A
isotig41626	6.767	7.083	7.37	5.493	481	0.432582475	isogroup26717	ENSG00000099364	ENST00000427128	FBXL19
isotig41631	3.819	1.266	1.881	1.164	481	0.643843466	isogroup26722	ENSG00000139044	ENST00000266383	B4GALNT3
isotig41634	4.942	3.166	3.11	4.141	481	0.09609604	isogroup26725	ENSG00000164362	ENST00000508104	TERT
isotig41635	1.626	2.031	1.26	1.218	481	0.259966559	isogroup26726	ENSG00000162384	ENST00000294360	C1orf123
isotig41642	1.578	2.89	3.033	3.304	481	0.340403547	isogroup26733	ENSG00000171812	ENST00000481785	COL8A2
isotig41650	1.079	2.216	2.879	2.699	481	0.111369956	isogroup26741	ENSG00000244537	ENST00000458321	KRTAP4-2
isotig41651	1.786	1.979	2.112	1.304	481	0.20753175	isogroup26742	ENSG00000108786	ENST00000225929	HSD17B1
isotig41653	2.574	1.701	1.266	2.979	481	0.139334561	isogroup26744	ENSG00000022976	ENST00000559098	ZNF839
isotig41655	2.099	1.239	2.483	1.88	476	0.359369129	isogroup26746	ENSG00000159579	ENST00000568505	RSPRY1
isotig41658	2.077	1.821	2.341	1.349	481	0.388338844	isogroup26749	ENSG00000167094	ENST00000373289	TTC16
isotig41663	8.071	7.535	8.271	13.619	480	0.537827835	isogroup26754	ENSG00000171443	ENST00000301073	ZNF524
isotig41666	3.204	1.904	1.269	2.379	480	0.003503795	isogroup26757	ENSG00000197892	ENST00000521515	KIF13B
isotig41670	6.948	13.125	14.233	13.573	480	0.333583828	isogroup26761	ENSG00000255181	ENST00000542437	CCDC166
isotig41671	1.748	2.498	3.54	3.685	480	0.043172766	isogroup26762	ENSG00000130518	ENST00000392413	KIAA1683
isotig41678	1.54	2.023	3.077	2.379	480	0.187185316	isogroup26769	ENSG00000182199	ENST00000553474	SHMT2
isotig41687	3.204	3.212	3.117	5.223	480	0.334138048	isogroup26778	ENSG00000116857	ENST00000455367	TMEM9
isotig41692	1.706	2.379	2.577	3.125	480	0.211307958	isogroup26783	ENSG00000115310	ENST00000405240	RTN4
isotig41694	2679.201	1967.606	892.541	921.847	477	0.517659878	isogroup26785	ENSG00000160808	ENST00000395869	MYL3
isotig41699	1.123	1.308	2.117	2.052	480	0.016354174	isogroup26790	ENSG00000131379	ENST00000435614	C3orf20
isotig41710	1.498	2.815	2.694	2.938	480	0.243386939	isogroup26801	ENSG00000162129	ENST00000544382	CLPB
isotig41723	1.042	2.146	1.658	2.524	479	0.3666961	isogroup26814	ENSG00000183248	ENST00000539422	AC010336.1
isotig41725	1.209	2.384	2.505	3.177	479	0.340037198	isogroup26816	ENSG00000129749	ENST00000534359	CHRNA10
isotig41734	19.929	8.463	12.72	10.096	479	0.811743819	isogroup26825	ENSG00000149925	ENST00000564595	ALDOA
isotig41737	4.253	3.576	4.933	2.852	479	0.447123694	isogroup26828	ENSG00000106004	ENST00000222726	HOXA5
isotig42091	2.121	1.616	2.038	1.047	471	0.355395656	isogroup27182	ENSG00000163162	ENST00000295317	RNF149
isotig42096	1.013	1.649	1.209	1.514	473	0.214313895	isogroup27187	ENSG00000197024	ENST00000475153	ZNF398
isotig42107	1.14	1.529	2.928	2.603	473	0.014428496	isogroup27198	ENSG00000180525	ENST00000441152	C10orf108
isotig42108	1.774	3.662	3.357	4.26	473	0.409117382	isogroup27199	ENSG00000130299	ENST00000361619	GTPBP3
isotig42120	1.816	2.294	4.607	2.651	473	0.287414519	isogroup27211	ENSG00000171729	ENST00000428417	TMEM51
isotig42121	2.527	2.824	2.415	2.929	465	0.129912828	isogroup27212	ENSG00000196333	ENST00000358740	AL158821.1
isotig42125	14.504	13.668	17.143	18.711	470	0.022563313	isogroup27216	ENSG00000183248	ENST00000539422	AC010336.1
isotig42128	4.053	3.059	2.772	2.507	473	0.287076351	isogroup27219	ENSG00000170917	ENST00000304430	NUDT6
isotig42129	1.013	1.852	2.108	2.036	473	0.139945142	isogroup27220	ENSG00000105327	ENST00000449228	BBC3
isotig42132	1.947	2.419	2.778	2.799	472	0.129912828	isogroup27223	ENSG00000117523	ENST00000495585	PRRC2C
isotig42142	2.75	1.936	2.074	1.566	472	0.381941835	isogroup27233	ENSG00000166317	ENST00000394810	SYNPO2L
isotig42146	3.765	5.646	2.661	2.703	472	0.145975802	isogroup27237	ENSG00000196422	ENST00000356818	PPP1R26
isotig42154	1.989	3.307	3.873	4.269	472	0.277363418	isogroup27245	ENSG00000163541	ENST00000393868	SUCLG1
isotig42158	135.65	352.699	155.15	214.051	472	0.928252048	isogroup27249	ENSG00000148180	ENST00000545652	GSN
isotig42162	1.354	1.693	1.761	2.324	472	0.133698429	isogroup27253	ENSG00000135213	ENST00000453279	POM121C
isotig42165	1.947	2.258	2.229	1.375	472	0.175114601	isogroup27256	ENSG00000108219	ENST00000429989	TSPAN14
isotig42181	3.047	3.066	2.739	1.47	472	0.3515443	isogroup27272	ENSG00000089558	ENST00000264661	KCNH4
isotig42185	1.824	1.051	2.157	1.616	471	0.343963703	isogroup27276	ENSG00000149476	ENST00000529479	DAK
isotig42187	1.102	2.828	2.588	2.567	471	0.340037198	isogroup27278	ENSG00000221843	ENST00000408964	C2orf16
isotig42189	1.611	3.032	2.783	2.425	471	0.088308785	isogroup27280	ENSG00000169297	ENST00000453287	NR0B1
isotig42199	2.671	2.626	1.648	2.138	471	0.247153754	isogroup27290	ENSG00000099954	ENST00000400585	CECR2
isotig42212	2.926	2.747	2.274	1.473	471	0.221302698	isogroup27303	ENSG00000167945	ENST00000301698	PRR25
isotig42213	2.713	1.779	3.214	2.518	471	0.390659052	isogroup27304	ENSG00000147724	ENST00000467365	FAM135B
isotig42215	3.563	2.868	2.195	1.427	471	0.390255129	isogroup27306	ENSG00000140859	ENST00000565397	KIFC3
isotig42218	2.756	6.021	3.919	2.947	471	0.699800857	isogroup27309	ENSG00000261321	ENST00000566692	FAM101B.1
isotig42227	2.416	2.384	1.255	1.236	471	0.069775306	isogroup27318	ENSG00000151553	ENST00000369248	FAM160B1
isotig42228	6.126	5.319	9.242	5.361	476	0.4762531	isogroup27319	ENSG00000185483	ENST00000544776	ROR1
isotig42230	1.611	2.384	3.057	3.518	471	0.329741865	isogroup27321	ENSG00000171396	ENST00000390661	KRTAP4-4
isotig42231	1.527	2.344	1.569	1.427	471	0.227117307	isogroup27322	ENSG00000143971	ENST00000272342	ETAA1
isotig42233	16.147	4.334	10.843	7.336	470	0.912264222	isogroup27324	ENSG00000133619	ENST00000485033	KRBA1
isotig42236	28.936	20.696	27.383	36.485	470	0.102878184	isogroup27327	ENSG00000120913	ENST00000381194	PDLIM2
isotig42245	616.966	430.472	379.013	373.423	470	0.435006012	isogroup27336	ENSG00000204628	ENST00000512805	GNB2L1

isotig42246	3.315	1.945	2.789	3.096	470	0.397788758	isogroup27337	ENSG00000117461	ENST00000540385	PIK3R3
isotig42247	3.443	1.66	2.121	1.715	470	0.44578981	isogroup27338	ENSG00000124177	ENST00000373233	CHD6
isotig42251	1.913	2.713	3.221	4.477	470	0.266185091	isogroup27342	ENSG00000064687	ENST00000433129	ABCA7
isotig42254	2.506	1.457	1.611	1.477	470	0.141429323	isogroup27345	ENSG00000100106	ENST00000417174	TRIOBP
isotig42258	18.565	13.311	13.465	11.026	469	0.580145788	isogroup27349	ENSG00000196810	ENST00000357591	C4orf42
isotig42265	1.402	1.985	3.34	1.668	470	0.304491997	isogroup27356	ENSG00000160299	ENST00000359568	PCNT
isotig42266	2.677	4.172	2.474	1.619	470	0.300490343	isogroup27357	ENSG00000083857	ENST00000441802	FAT1
isotig42591	2.11	2.707	3.024	2.894	464	0.149996243	isogroup27682	ENSG00000072364	ENST00000378595	AFF4
isotig42593	1.506	3.078	4.616	3.716	464	0.014428496	isogroup27684	ENSG00000198816	ENST00000394341	ZNF358
isotig42594	1.463	2.379	2.506	2.075	464	0.098416247	isogroup27685	ENSG00000064687	ENST00000433129	ABCA7
isotig42597	1.463	2.297	2.19	2.412	464	0.164396558	isogroup27688	ENSG00000133226	ENST00000323848	SRRM1
isotig42599	1.42	3.651	4.099	3.957	464	0.302462989	isogroup27690	ENSG00000148218	ENST00000409155	ALAD
isotig42600	1.162	1.64	2.19	2.703	464	0.178778087	isogroup27691	ENSG00000171560	ENST00000403106	FGA
isotig42603	16.14	15.302	15.44	9.746	464	0.273183287	isogroup27694	ENSG00000188786	ENST00000373036	MTF1
isotig42606	1.075	1.806	1.353	1.496	464	0.437288645	isogroup27697	ENSG00000251359	ENST00000506413	C4orf38
isotig42626	2.153	1.806	2.069	1.207	464	0.394613737	isogroup27717	ENSG00000212935	ENST00000391620	KRTAP10-3
isotig42631	2.409	1.231	2.267	1.157	464	0.555431352	isogroup27722	ENSG00000213999	ENST00000409447	MEF2B
isotig42643	13.587	14.963	15.194	11.555	463	0.172540768	isogroup27734	ENSG00000102103	ENST00000447146	PQBP1
isotig42647	3.286	2.472	2.119	1.6	462	0.371543173	isogroup27738	ENSG00000170340	ENST00000405767	B3GNT2
isotig42653	1.38	2.59	3.909	3.674	463	0.071109191	isogroup27744	ENSG00000139574	ENST00000267017	NPFF
isotig42657	1.683	2.59	3.071	4.207	463	0.287724506	isogroup27748	ENSG00000120055	ENST00000239125	C10orf95
isotig42659	118.618	147.629	138.626	164.976	463	0.351534906	isogroup27750	ENSG00000079739	ENST00000371084	PGM1
isotig42665	1.898	2.015	3.749	1.933	463	0.425762756	isogroup27756	ENSG00000162591	ENST00000294599	MEGF6
isotig42677	2.071	2.959	1.516	1.838	463	0.262145863	isogroup27768	ENSG00000163728	ENST00000492617	TTC14
isotig42678	6.039	1.644	3.708	1.644	463	0.841446231	isogroup27769	ENSG00000213445	ENST00000534313	SIPA1
isotig42682	3.019	2.836	1.555	1.741	463	0.222993537	isogroup27773	ENSG00000179755	ENST00000319817	CTD-2144E22.5.1
isotig42694	31.297	27.933	25.139	28.154	462	0.055854062	isogroup27785	ENSG00000136274	ENST00000490531	NACAD
isotig42695	1.08	1.236	2.918	3.102	462	0.057310062	isogroup27786	ENSG00000116874	ENST00000537870	WARS2
isotig42701	2.119	1.524	1.439	1.017	462	0.228798753	isogroup27792	ENSG00000119596	ENST00000549293	YLPM1
isotig43092	2.809	1.714	1.218	1.132	455	0.195461036	isogroup28183	ENSG00000125834	ENST00000493263	STK35
isotig43102	1.404	2.552	2.393	2.558	455	0.175114601	isogroup28193	ENSG00000231350	ENST00000547796	C6orf205.4
isotig43104	1.141	1.714	2.312	1.919	455	0.091953483	isogroup28195	ENSG00000198691	ENST00000370225	ABCA4
isotig43590	1.808	2.412	3.676	3.595	442	0.198589088	isogroup28681	ENSG00000010404	ENST00000541269	IDS
isotig43595	1.265	1.292	1.964	1.468	442	0.104832043	isogroup28686	ENSG00000226650	ENST00000435029	KIF4B
isotig43597	2.665	2.197	1.253	1.873	442	0.216690464	isogroup28688	ENSG00000170832	ENST00000300896	USP32
isotig43599	5.242	3.532	1.129	1.267	442	0.278274592	isogroup28690	ENSG00000182670	ENST00000438055	TTC3
isotig43600	3.163	3.36	1.505	2.278	442	0.194089577	isogroup28691	ENSG00000165494	ENST00000530660	PCF11
isotig43603	4.586	3.532	1.817	2.609	442	0.121242579	isogroup28694	ENSG00000145740	ENST00000396591	SLC30A5
isotig43605	1.31	2.068	3.552	3.443	442	0.217178928	isogroup28696	ENSG00000128059	ENST00000264220	PPAT
isotig43606	4.744	5.597	2.088	2.077	442	0.174457053	isogroup28697	ENSG00000129467	ENST00000554068	ADCY4
isotig43607	2.484	3.014	2.758	1.824	442	0.010060494	isogroup28698	ENSG00000161847	ENST00000293677	RAVER1
isotig43608	2.665	1.896	1.505	1.57	442	0.057310062	isogroup28699	ENSG00000255181	ENST00000542437	CCDC166
isotig43609	1.219	2.197	2.59	2.683	442	0.238915608	isogroup28700	ENSG00000188626	ENST00000340249	RP11-578F21.5.1
isotig43610	3.434	2.885	3.133	2.837	442	0.203492523	isogroup28701	ENSG00000089094	ENST00000538379	KDM2B
isotig43612	1.174	1.679	2.674	3.749	442	0.212425791	isogroup28703	ENSG00000182095	ENST00000399544	TNRC18
isotig43616	2.801	1.55	2.548	1.367	442	0.517706846	isogroup28707	ENSG00000181472	ENST00000325144	ZBTB2
isotig43617	4.247	3.145	2.59	2.584	442	0.188434658	isogroup28708	ENSG00000198722	ENST00000535471	UNC13B
isotig43620	1.038	2.326	2.758	2.937	442	0.264052754	isogroup28711	ENSG00000178921	ENST00000314666	PFAS
isotig43623	23.458	26.975	26.964	19.646	441	0.086664913	isogroup28714	ENSG00000130402	ENST00000445727	ACTN4
isotig43625	25.678	19.379	21.522	24.061	441	0.20694935	isogroup28716	ENSG00000159692	ENST00000515399	CTBP1
isotig43626	1.313	2.762	2.68	1.93	441	0.115681596	isogroup28717	ENSG00000137656	ENST00000260210	BUD13
isotig43627	1.313	1.943	2.848	3.299	441	0.233570677	isogroup28718	ENSG00000148400	ENST00000277541	NOTCH1
isotig43630	1.744	1.057	1.404	1.143	441	0.166303449	isogroup28721	ENSG00000166181	ENST00000531273	API5
isotig43631	2.626	1.424	1.549	1.37	441	0.332071466	isogroup28722	ENSG00000107872	ENST00000440407	FBXL15
isotig43632	7.653	13.596	8.583	6.651	441	0.652100398	isogroup28723	ENSG00000124193	ENST00000483871	SRSF6
isotig43638	1.041	2.331	2.512	2.943	441	0.35027617	isogroup28729	ENSG00000198130	ENST00000409934	HIBCH
isotig43642	81.83	114.723	134.612	111.476	441	0.106560457	isogroup28733	ENSG00000105953	ENST00000543843	OGDH
isotig43643	1.494	1.726	1.213	1.472	441	0.245857443	isogroup28734	ENSG00000146648	ENST00000533450	EGFR
isotig43645	2.49	2.59	2.177	1.828	441	0.277006463	isogroup28736	ENSG00000167978	ENST00000544933	SRRM2
isotig43646	3.125	2.676	2.512	2.59	441	0.021652138	isogroup28737	ENSG00000188786	ENST00000373036	MTF1
isotig43653	123.991	52.356	154.166	65.383	441	0.952158638	isogroup28744	ENSG00000117984	ENST00000429746	CTSD
isotig43655	1.132	2.374	2.385	3.603	441	0.384957165	isogroup28746	ENSG00000081307	ENST00000494238	UBA5
isotig43656	13.841	12.848	14.195	11.1	441	0.297503194	isogroup28747	ENSG00000090621	ENST00000437136	PABPC4
isotig43657	23.435	23.587	22.673	23.732	441	0.206507853	isogroup28748	ENSG00000184227	ENST00000557556	ACOT1
isotig43658	4.63	2.207	1.845	1.018	440	0.638808522	isogroup28749	ENSG00000204278	ENST00000421688	TMEM235
isotig43660	10.897	10.559	4.299	4.6	438	0.033563162	isogroup28751	ENSG00000198604	ENST00000543083	BAZ1A
isotig43661	1.361	1.6	1.595	1.627	440	0.141016007	isogroup28752	ENSG00000142751	ENST00000374135	GPN2
isotig43664	35.493	18.775	28.452	19.893	440	0.78934959	isogroup28755	ENSG00000108821	ENST00000225964	COL1A1
isotig43667	3.268	2.423	1.259	1.627	440	0.149488991	isogroup28758	ENSG00000100354	ENST00000454349	TNRC6B
isotig43668	4.936	8.712	6.76	6.712	437	0.341305328	isogroup28759	ENSG00000204839	ENST00000529971	C8orf73
isotig43671	4.584	3.936	2.225	2.695	440	0.176260615	isogroup28762	ENSG00000196586	ENST00000428345	MYO6
isotig43673	2.814	3.159	2.434	1.832	440	0.116480048	isogroup28764	ENSG00000119139	ENST00000539225	TJP2
isotig43685	1.27	2.898	3.064	3.155	440	0.348838957	isogroup28776	ENSG00000066735	ENST00000423312	KIF26A
isotig43687	1.316	2.509	3.568	3.052	440	0.03231382	isogroup28778	ENSG00000174791	ENST00000530056	RIN1
isotig43692	2.866	2.645	4.164	3.467	439	0.270158563	isogroup28783	ENSG00000159363	ENST00000452699	ATP13A2
isotig43694	2.774	2.125	3.492	1.938	439	0.424147065	isogroup28785	ENSG00000138759	ENST00000380674	FRAS1
isotig43695	1.957	2.991	1.346	1.376	439	0.189533704	isogroup28786	ENSG00000135541	ENST00000457866	AHI1
isotig43697	8.28	4.075	6.014	3.722	439	0.669102728	isogroup28788	ENSG00000196405	ENST00000402714	EVL
isotig43698	24.884	23.458	27.383	22.235	439	0.323852108	isogroup28789	ENSG00000162746	ENST00000367946	FCRLB
isotig43699	3.185	3.599	4.501	3.314	439	0.042524611	isogroup28790	ENSG00000076944	ENST00000320400	STXBP2
isotig43703	2.321	1.344	2.018	1.581	439	0.3666961	isogroup28794	ENSG00000076944	ENST00000320400	STXBP2
isotig43706	3.048	1.387	1.893	1.376	439	0.439618246	isogroup28797	ENSG00000171448	ENST00000373656	ZBTB26

isotig43711	1.592	1.474	2.524	1.989	439	0.187795897	isogroup28802	ENSG00000204038	ENST00000356374	AL359195.1
isotig43713	3.093	2.428	2.062	3.059	439	0.014428496	isogroup28804	ENSG00000197927	ENST00000355171	C2orf27A
isotig43717	1.501	1.863	3.155	2.499	439	0.115681596	isogroup28808	ENSG00000166855	ENST00000546194	CLPX
isotig43718	6.05	3.772	3.702	3.62	439	0.523934771	isogroup28809	ENSG00000167377	ENST00000564528	ZNF23
isotig43720	1.729	2.125	3.071	3.467	439	0.088308785	isogroup28811	ENSG00000183751	ENST00000568546	TBL3
isotig43724	1.319	2.472	3.743	3.212	439	0.074481476	isogroup28815	ENSG00000166881	ENST00000379391	TMEM194A
isotig43727	4.549	4.378	2.396	3.825	439	0.376258736	isogroup28818	ENSG00000178229	ENST00000321545	ZNF543
isotig43729	1.592	1.863	2.902	1.836	439	0.250629368	isogroup28820	ENSG00000146950	ENST00000380913	SHROOM2
isotig43730	1.046	1.344	2.777	2.804	439	0.003503795	isogroup28821	ENSG00000183624	ENST00000509551	C3orf37
isotig43737	1.957	3.426	3.071	4.03	439	0.270910047	isogroup28828	ENSG00000187634	ENST00000341065	SAMD11
isotig43740	1.231	1.824	1.854	1.534	438	0.210133764	isogroup28831	ENSG00000130202	ENST00000252483	PVRL2
isotig43742	47.374	18.164	57.08	38.486	438	0.839060269	isogroup28833	ENSG00000099834	ENST00000397542	CDHR5
isotig43747	1.916	1.868	1.687	1.329	438	0.205305478	isogroup28838	ENSG00000115266	ENST00000535453	APC2
isotig43749	10.623	9.082	16.737	17.429	438	0.141476291	isogroup28840	ENSG00000177169	ENST00000542419	ULK1
isotig43753	1.094	1.607	2.023	2.708	438	0.322057939	isogroup28844	ENSG00000198324	ENST00000450786	FAM109A
isotig43756	1.185	2.043	3.119	2.658	438	0.050227324	isogroup28847	ENSG00000055118	ENST00000430723	KCNH2
isotig43757	2.735	1.824	2.699	2.096	438	0.334898925	isogroup28848	ENSG00000184857	ENST00000333050	TMEM186
isotig43758	2.735	1.957	1.644	1.737	438	0.151724656	isogroup28849	ENSG00000132881	ENST00000375599	RSG1
isotig43760	6.384	4.084	8.306	2.811	438	0.718935523	isogroup28851	ENSG00000092758	ENST00000537652	COL9A3
isotig43764	4.321	3.21	2.565	2.396	439	0.490972796	isogroup28855	ENSG00000174748	ENST00000510788	RPL15
isotig43766	1.737	1.828	3.085	1.538	437	0.404646051	isogroup28857	ENSG00000178279	ENST00000312693	TNP2
isotig43773	2.098	1.26	1.518	1.073	438	0.090788683	isogroup28864	ENSG00000152689	ENST00000402538	RASGRP3
isotig43776	1.371	1.437	2.451	2.51	437	0.128719847	isogroup28867	ENSG00000249428	ENST00000506607	RP11-503N18.3.1
isotig43777	1.691	2.787	2.325	2.664	437	0.230433231	isogroup28868	ENSG00000158163	ENST00000327532	DZIP1L
isotig43778	2.467	1.35	2.156	1.332	437	0.344395807	isogroup28869	ENSG00000108592	ENST00000427159	FTSJ3
isotig43786	2.97	1.961	3.041	1.588	437	0.551288795	isogroup28877	ENSG00000189348	ENST00000338885	AC092070.1
isotig43790	32.492	22.302	32.874	23.462	437	0.663823552	isogroup28881	ENSG00000006015	ENST00000358607	C19orf60
isotig43792	1.188	1.654	2.705	2.254	437	0.041209514	isogroup28883	ENSG00000178965	ENST00000326665	C1orf173
isotig43796	1.188	3.398	3.465	2.97	437	0.287724506	isogroup28887	ENSG00000099991	ENST00000405822	CABIN1
isotig43798	1.416	1.437	1.817	1.435	437	0.104832043	isogroup28889	ENSG00000132906	ENST00000546424	CASP9
isotig43799	1.783	1.918	3.549	2.204	437	0.23384309	isogroup28890	ENSG00000170004	ENST00000452447	CHD3
isotig43802	1.416	1.568	2.577	2.101	437	0.189533704	isogroup28893	ENSG00000130997	ENST00000511885	POLN
isotig43803	1.511	2.14	2.752	3.644	436	0.304482603	isogroup28894	ENSG00000183044	ENST00000566590	ABAT
isotig43807	2.977	3.493	2.456	3.08	436	0.118715714	isogroup28898	ENSG00000133454	ENST00000543971	MYO18B
isotig43812	1.236	1.615	2.202	1.541	436	0.090788683	isogroup28903	ENSG00000117983	ENST00000529681	MUC5B
isotig43815	1.053	2.227	2.287	1.899	436	0.173818291	isogroup28906	ENSG00000139613	ENST00000550164	SMARCC2
isotig43825	1.787	2.183	1.312	1.181	436	0.204244007	isogroup28916	ENSG00000101811	ENST00000475126	CSTF2
isotig43826	3.803	4.933	2.287	3.337	436	0.523549636	isogroup28917	ENSG00000137941	ENST00000480174	TTLL7
isotig43827	1.144	1.789	3.048	2.362	436	0.010060494	isogroup28918	ENSG00000169682	ENST00000566059	SPNS1
isotig43828	2.062	2.358	1.61	3.287	436	0.384835049	isogroup28919	ENSG00000105556	ENST00000264819	MIER2
isotig43830	1.282	1.922	2.626	2.567	436	0.248619148	isogroup28921	ENSG00000142347	ENST00000305795	MYO1F
isotig43838	2.159	1.575	2.503	1.802	435	0.269782821	isogroup28929	ENSG00000135144	ENST00000257600	DTX1
isotig43841	3.03	2.494	2.166	1.389	435	0.40546329	isogroup28932	ENSG00000233831	ENST00000458265	ZNF311
isotig43843	1.929	2.145	4.287	3.602	435	0.297888329	isogroup28934	ENSG00000173327	ENST00000524848	MAP3K11
isotig43844	1.423	1.487	3.184	2.625	435	0.074039979	isogroup28935	ENSG00000173692	ENST00000409643	PSMD1
isotig43845	1.285	2.189	2.122	2.572	435	0.274770797	isogroup28936	ENSG00000167306	ENST00000324581	MYO5B
isotig43847	2.251	1.313	2.207	1.595	435	0.405688735	isogroup28938	ENSG00000182500	ENST00000330079	ORAI1
isotig43849	1.699	1.487	2.166	2.676	435	0.083029608	isogroup28940	ENSG00000176978	ENST00000443858	DPP7
isotig43850	3.214	2.451	3.439	3.037	435	0.339821147	isogroup28941	ENSG00000149782	ENST00000540288	PLCB3
isotig44091	1.4	2.446	2.848	4.185	428	0.486473285	isogroup29182	ENSG00000115266	ENST00000535453	APC2
isotig44092	2.706	3.558	1.897	2.04	428	0.313566168	isogroup29183	ENSG00000107625	ENST00000541832	DDX50
isotig44093	2.612	2.446	1.855	2.407	428	0.084617119	isogroup29184	ENSG00000218336	ENST00000511685	ODZ3
isotig44095	1.54	2.668	2.848	3.505	428	0.3515443	isogroup29186	ENSG00000187642	ENST00000433179	C1orf170
isotig44098	1.026	2.491	2.374	2.72	428	0.166247088	isogroup29189	ENSG00000169282	ENST00000490337	KCNAB1
isotig44101	1.259	2.757	3.063	2.876	428	0.227483655	isogroup29192	ENSG00000234876	ENST00000457094	NOTCH4
isotig44108	3.5	2.668	2.416	1.516	428	0.383022094	isogroup29199	ENSG00000260665	ENST00000566884	DUX4L4
isotig44109	1.586	2.136	3.322	2.668	428	0.054116255	isogroup29200	ENSG00000099991	ENST00000405822	CABIN1
isotig44113	1.496	2.941	3.632	4.77	427	0.394538589	isogroup29204	ENSG00000180011	ENST00000537114	ZADH2
isotig44114	1.496	2.852	3.677	4.981	427	0.32766589	isogroup29205	ENSG00000177508	ENST00000329734	IRX3
isotig44115	3.508	2.363	2.335	1.625	427	0.369504772	isogroup29206	ENSG00000135823	ENST00000542060	STX6
isotig44118	2.34	4.637	5.45	5.506	427	0.329957917	isogroup29209	ENSG00000125648	ENST00000334510	SLC25A23
isotig44119	1.829	1.34	1.516	1.157	426	0.241968513	isogroup29210	ENSG00000113761	ENST00000503039	ZNF346
isotig44120	1.262	1.337	1.471	1.625	427	0.016354174	isogroup29211	ENSG00000172728	ENST00000524021	FUT10
isotig44122	1.075	1.916	2.379	2.15	427	0.189533704	isogroup29213	ENSG00000169876	ENST00000379439	MUC17
isotig44124	2.246	1.07	1.859	1.311	427	0.40001503	isogroup29215	ENSG00000165684	ENST00000298532	SNAPC4
isotig44126	1.075	2.808	3.199	3.199	427	0.204178252	isogroup29217	ENSG00000167107	ENST00000541920	ACSF2
isotig44128	1.215	2.052	2.724	2.307	427	0.139334561	isogroup29219	ENSG00000124440	ENST00000528563	HIF3A
isotig44129	24.261	18.986	29.164	20.154	421	0.483796122	isogroup29220	ENSG00000054965	ENST00000356467	FAM168A
isotig44133	1.356	2.763	3.244	3.199	427	0.269331931	isogroup29224	ENSG00000170004	ENST00000449744	CHD3
isotig44135	3.696	2.585	3.112	2.569	427	0.357725257	isogroup29226	ENSG00000187987	ENST00000481983	ZSCAN23
isotig44136	3.415	3.208	3.934	3.04	427	0.257862403	isogroup29227	ENSG00000213999	ENST00000410050	MEF2B
isotig44137	2.293	3.255	2.81	2.15	427	0.210312242	isogroup29228	ENSG00000086827	ENST00000535142	ZW10
isotig44138	1.356	1.337	2.724	1.52	427	0.256472158	isogroup29229	ENSG00000172037	ENST00000418109	LAMB2
isotig44140	3.602	1.916	2.119	1.311	427	0.512869167	isogroup29231	ENSG00000184809	ENST00000380612	C21orf88
isotig44142	4.5	3.664	3.467	2.155	426	0.446437965	isogroup29233	ENSG00000130518	ENST00000427634	KIAA1683
isotig44143	1.171	1.296	1.256	1.523	426	0.125	isogroup29234	ENSG00000122778	ENST00000440172	KIAA1549
isotig44145	2.298	2.19	1.906	3.207	426	0.263057038	isogroup29236	ENSG00000212857	ENST00000391545	AL356585.1
isotig44146	2.298	1.563	2.514	2.838	426	0.003503795	isogroup29237	ENSG00000132768	ENST00000255108	DPH2
isotig44147	1.594	1.251	1.561	1.577	426	0.11153904	isogroup29238	ENSG00000147439	ENST00000519513	BIN3
isotig44148	1.359	2.235	2.469	1.998	426	0.201961374	isogroup29239	ENSG00000197299	ENST00000560509	BLM
isotig44149	1.406	1.474	2.601	2.681	426	0.041209514	isogroup29240	ENSG00000227804	ENST00000449113	PPP1R10
isotig44152	2.906	2.279	3.035	2.155	426	0.444512287	isogroup29243	ENSG00000174953	ENST00000544526	DHX36

isotig44153	1.359	1.786	1.993	2.469	426	0.230508379	isogroup29244	ENSG00000103876	ENST00000561421	FAH
isotig44599	1.063	2.305	2.726	2.927	413	0.265903284	isogroup29690	ENSG00000187867	ENST00000340790	PALM3
isotig44601	2.128	1.337	2.862	1.356	413	0.480076276	isogroup29692	ENSG00000162004	ENST00000482878	CCDC78
isotig44602	1.111	2.213	2.547	2.332	413	0.141429323	isogroup29693	ENSG00000122729	ENST00000432017	ACO1
isotig44604	1.983	1.107	1.833	1.789	413	0.243687533	isogroup29695	ENSG00000198788	ENST00000441003	MUC2
isotig44605	6.092	4.886	2.995	1.789	413	0.090957767	isogroup29696	ENSG00000196411	ENST00000358173	EPHB4
isotig44607	1.596	1.659	2.414	1.518	413	0.195461036	isogroup29698	ENSG00000181026	ENST00000562177	AEN
isotig44608	5.755	3.225	2.637	2.603	413	0.496017134	isogroup29699	ENSG00000171100	ENST00000542741	MTM1
isotig44614	3.869	17.651	4.426	2.981	413	0.936969264	isogroup29705	ENSG00000130635	ENST00000371817	COL5A1
isotig44617	1.354	1.567	1.789	2.818	413	0.288119035	isogroup29708	ENSG00000171467	ENST00000361428	ZNF318
isotig44622	1.208	1.153	1.252	1.194	413	0.087209739	isogroup29713	ENSG00000084463	ENST00000261167	WBP11
isotig44623	6.044	2.765	2.191	1.734	413	0.63012888	isogroup29714	ENSG00000128917	ENST00000249749	DLL4
isotig44627	33.932	92.862	91.607	85.58	412	0.671770497	isogroup29718	ENSG00000101460	ENST00000397709	MAP1LC3A
isotig44629	1.697	1.709	1.794	2.01	412	0.078182536	isogroup29720	ENSG00000167291	ENST00000310924	TBC1D16
isotig44635	1.939	1.893	1.032	1.25	412	0.016354174	isogroup29726	ENSG00000198131	ENST00000415203	ZNF544
isotig44637	2.036	2.357	1.255	2.5	412	0.290937101	isogroup29728	ENSG00000106771	ENST00000374587	C9orf5
isotig44640	4.121	3.141	4.214	3.532	412	0.315163072	isogroup29731	ENSG00000160447	ENST00000291906	PKN3
isotig45091	10.839	7.651	6.123	5.063	398	0.637155257	isogroup30182	ENSG00000144711	ENST00000429247	IQSEC1
isotig45095	1.405	2.869	3.156	2.701	398	0.116480048	isogroup30186	ENSG00000164393	ENST00000296862	GPR111
isotig45098	1.807	2.008	2.784	3.093	398	0.084617119	isogroup30189	ENSG00000103056	ENST00000568373	SMPD3
isotig45100	2.759	2.583	2.367	2.08	398	0.077224393	isogroup30191	ENSG00000160305	ENST00000427143	DIP2A
isotig45101	1.455	1.435	2.877	2.812	398	0.063566168	isogroup30192	ENSG00000127948	ENST00000450476	POR
isotig45104	2.46	3.156	2.553	2.53	398	0.173724356	isogroup30195	ENSG00000099995	ENST00000536049	SF3A1
isotig45106	1.405	1.053	2.922	2.475	398	0.014428496	isogroup30197	ENSG00000163348	ENST00000368457	PYGO2
isotig45107	1.957	2.965	4.5	4.332	398	0.027992786	isogroup30198	ENSG00000159259	ENST00000314103	CHAF1B
isotig45109	1.153	2.106	2.04	2.588	398	0.377461111	isogroup30200	ENSG00000141497	ENST00000433935	ZMYND15
isotig45111	2.56	1.769	2.412	2.307	398	0.246129856	isogroup30202	ENSG00000139636	ENST00000551782	LMBR1L
isotig45112	1.505	4.113	3.156	3.206	398	0.469339445	isogroup30203	ENSG00000234409	ENST00000540078	AC006547.14.1
isotig45115	22.58	16.452	12.201	14.399	398	0.275400165	isogroup30206	ENSG00000159140	ENST00000455528	SON
isotig45591	1.198	1.54	2.507	1.812	383	0.076266251	isogroup30682	ENSG00000099960	ENST00000403586	SLC7A4
isotig45594	1.407	1.888	3.183	1.93	383	0.222993537	isogroup30685	ENSG00000100092	ENST00000357436	SH3BP1
isotig45597	1.094	1.441	1.35	1.695	383	0.173245284	isogroup30688	ENSG00000108511	ENST00000484302	HOXB6
isotig45598	1.303	1.193	2.893	1.577	383	0.303120538	isogroup30689	ENSG00000081189	ENST00000514015	MEF2C
isotig45600	3.441	3.577	2.796	2.454	383	0.115456151	isogroup30691	ENSG00000144229	ENST00000543459	THSD7B
isotig45602	2.086	3.031	4.82	3.859	383	0.025447133	isogroup30693	ENSG00000197483	ENST00000391718	ZNF628
isotig45604	41.298	67.042	68.7	94.692	383	0.698870895	isogroup30695	ENSG00000177238	ENST00000322122	TRIM72
isotig45607	1.616	2.386	2.217	1.869	383	0.045840535	isogroup30698	ENSG00000050327	ENST00000056217	ARHGEF5
isotig45608	1.094	1.689	2.41	2.629	383	0.132355151	isogroup30699	ENSG00000005339	ENST00000543883	CREBBP
isotig45614	1.564	2.883	3.713	3.097	383	0.178749906	isogroup30705	ENSG00000135776	ENST00000344517	ABCB10
isotig45616	2.347	2.585	1.59	1.462	383	0.087435184	isogroup30707	ENSG00000130702	ENST00000370677	LAMA5
isotig45617	1.721	1.838	1.397	1.577	383	0.146079131	isogroup30708	ENSG00000167202	ENST00000409931	TBC1D2B
isotig45618	3.285	3.031	2.747	4.384	383	0.312110168	isogroup30709	ENSG00000186260	ENST00000318282	MKL2
isotig45622	1.202	1.793	2.754	1.935	382	0.010060494	isogroup30713	ENSG00000063015	ENST00000540419	SEZ6
isotig45627	1.045	2.194	2.466	2.99	382	0.227370933	isogroup30718	ENSG00000157322	ENST00000568461	CLEC18A
isotig45628	330.908	401.344	325.719	285.759	381	0.075251747	isogroup30719	ENSG00000159199	ENST00000393366	ATP5G1
isotig45629	1.62	2.691	5.173	5.393	382	0.094226723	isogroup30720	ENSG00000162105	ENST00000425049	SHANK2
isotig45636	2.406	1.346	1.982	2.11	382	0.155763884	isogroup30727	ENSG00000089195	ENST00000203001	TRMT6
isotig45638	1.149	1.346	3.385	2.052	382	0.292158263	isogroup30729	ENSG00000127527	ENST00000535753	EPS15L1
isotig45640	1.045	1.495	1.45	1.874	382	0.278669122	isogroup30731	ENSG00000164877	ENST00000413446	MICALL2
isotig45641	1.568	2.042	3.141	2.343	382	0.039415345	isogroup30732	ENSG00000184154	ENST00000427369	LRTOMT
isotig45642	6.377	2.741	3.874	2.814	382	0.767349891	isogroup30733	ENSG00000038382	ENST00000513206	TRIO
isotig45643	3.712	2.042	2.466	1.466	382	0.566262118	isogroup30734	ENSG00000079313	ENST00000543452	REXO1
isotig45645	8.783	2.791	2.223	1.23	382	0.800631247	isogroup30736	ENSG00000108479	ENST00000437911	GALK1
isotig45646	4.704	8.372	5.026	3.809	382	0.360176975	isogroup30737	ENSG00000119669	ENST00000238647	IRF2BPL
isotig46093	13.744	6.103	10.047	10.009	340	0.515499361	isogroup31184	ENSG00000185899	ENST00000332690	TAS2R60
isotig46094	42.475	69.342	65.853	93.112	339	0.691994815	isogroup31185	ENSG00000177238	ENST00000322122	TRIM72
isotig46096	180.679	137.382	305.971	291.488	340	0.393740137	isogroup31187	ENSG00000154358	ENST00000366709	OBSCN
isotig46101	30.48	15.228	17.895	17.083	325	0.730837153	isogroup31192	ENSG00000203930	ENST00000370535	RP1-177G6.2.1
isotig46104	148.24	111.984	150.804	193.502	317	0.008501165	isogroup31195	ENSG00000261109	ENST00000568193	PLEC.1
isotig46106	150.351	204.437	195.576	238.959	316	0.514747877	isogroup31197	ENSG00000108528	ENST00000544061	SLC25A11
isotig46109	3.338	3.611	5.759	2.807	311	0.519491621	isogroup31200	ENSG00000189221	ENST00000338702	MAOA
isotig46111	114.605	99.402	128.529	149.098	306	0.480226572	isogroup31202	ENSG00000154358	ENST00000570156	OBSCN
isotig46115	135.694	179.064	102.024	216.178	297	0.736717517	isogroup31206	ENSG00000128591	ENST00000325888	FLNC
isotig46118	43.934	61.107	59.659	94.334	290	0.759928985	isogroup31209	ENSG00000136826	ENST00000411706	KLF4
isotig46119	28.847	131.587	53.726	97.632	288	0.948110017	isogroup31210	ENSG00000158458	ENST00000545385	NRG2
isotig46121	62.39	111.911	114.585	162.741	282	0.729014804	isogroup31212	ENSG00000177238	ENST00000322122	TRIM72
isotig46122	80.379	59.33	141.496	139.482	282	0.27747614	isogroup31213	ENSG00000154358	ENST00000570156	OBSCN
isotig46124	518.88	442.883	296.179	190.832	274	0.598566544	isogroup31215	ENSG00000143549	ENST00000515609	TPM3
isotig46126	113.219	109.888	91.673	113.015	269	0.262737657	isogroup31217	ENSG00000196923	ENST00000505074	PDLIM7
isotig46127	155.985	133.674	138.345	161.371	264	0.090995341	isogroup31218	ENSG00000018625	ENST00000392233	ATP1A2
isotig46128	31.843	23.737	39.161	39.365	255	0.499558503	isogroup31219	ENSG00000196531	ENST00000454682	NACA
isotig46129	2.236	1.544	2.637	2.162	259	0.368058165	isogroup31220	ENSG00000131115	ENST00000418980	ZNF227
isotig46138	245.669	443.285	356.163	410.268	239	0.679445029	isogroup31229	ENSG00000100412	ENST00000396512	ACO2
isotig46141	158.161	73.636	143.962	81.292	236	0.879048621	isogroup31232	ENSG00000154380	ENST00000284563	ENAH
isotig46142	2.645	2.197	2.368	2.201	234	0.03231382	isogroup31233	ENSG00000170209	ENST00000303941	ANKK1
isotig46144	160.217	114.032	183.561	271.982	221	0.186386864	isogroup31235	ENSG00000110651	ENST00000526072	CD81

Identified Proteins

KEGG ID	GOTerm	Nr. Genes	ssociated G	Term PValue (adj.*)	Associated Genes Found
10	Glycolysis / Gluconeogenesis	21	31.81818	6.97E-12	ALDH2, ALDOA, ALDOC, DLAT, DLD, ENO1, ENO3, FBP2, GAPDH, GPI, LDHA, LDHB, PDHA1, PDHB, PFKM, PGAM1, PGAM2, PGK1, PGM1, PKM2, TPI1
20	Citrate cycle (TCA cycle)	20	66.66666	6.97E-19	ACO2, CS, DLAT, DLD, DLST, FH, IDH2, IDH3A, IDH3B, IDH3G, MDH1, MDH2, OGDH, PDHA1, PDHB, SDHA, SDHB, SUCLA2, SUCLG1, SUCLG2
30	Pentose phosphate pathway	6	20.68966	8.54E-03	ALDOA, ALDOC, FBP2, GPI, PFKM, PGM1
51	Fructose and mannose metabolism	7	19.44445	5.56E-03	AKR1B10, ALDOA, ALDOC, FBP2, PFKM, PHPT1, TPI1
62	Fatty acid elongation	5	21.73913	1.52E-02	ACAA2, ECHS1, HADH, HADHA, HADHB
71	Fatty acid metabolism	12	27.27273	3.69E-06	ACAA2, ACADM, ACADS, ACADSB, ACADVL, ACAT1, ACSL1, ALDH2, ECHS1, HADH, HADHA, HADHB
190	Oxidative phosphorylation	34	25.56391	6.20E-16	ATP5A1, ATP5B, ATP5C1, ATP5F1, ATP5H, ATP5J2, ATP5O, COX5A, COX6B1, COX6C, COX7A2, CYC1, NDUFA11, NDUFA13, NDUFA4, NDUFA5, NDUFA8, NDUFA9, NDUFAB1, NDUFB10, NDUFB8, NDUFS1, NDUFS2, NDUFS3, NDUFS4, NDUFS6, NDUFS7, NDUFV1, NDUFV2, SDHA, SDHB, UQCRB, UQCRC1, UQCRC2
270	Cysteine and methionine metabolism	6	17.14286	1.82E-02	AHCY, GOT1, GOT2, LDHA, LDHB, MPST
280	Valine, leucine and isoleucine degradation	14	31.81818	5.41E-08	ACAA2, ACADM, ACADS, ACADSB, ACAT1, ALDH2, ALDH6A1, DLD, ECHS1, HADH, HADHA, HADHB, HIBADH, HSD17B10
310	Lysine degradation	8	16.32653	7.73E-03	ACAT1, ALDH2, DLST, ECHS1, HADH, HADHA, NSD1, OGDH
330	Arginine and proline metabolism	8	14.03509	1.67E-02	ACY1, ALDH2, CKM, CKMT1A, CKMT2, GAMT, GOT1, GOT2
360	Phenylalanine metabolism	4	22.22222	2.98E-02	GOT1, GOT2, MIF, PRDX6
380	Tryptophan metabolism	7	17.5	9.08E-03	ACAT1, ALDH2, CAT, ECHS1, HADH, HADHA, OGDH
410	beta-Alanine metabolism	5	17.24138	3.26E-02	ACADM, ALDH2, ALDH6A1, ECHS1, HADHA
500	Starch and sucrose metabolism	7	12.5	4.38E-02	AGL, GPI, GYS1, PGM1, PYGL, PYGM, UGP2
620	Pyruvate metabolism	15	36.58537	1.70E-09	ACAT1, ACYP2, AKR1B10, ALDH2, DLAT, DLD, GLO1, LDHA, LDHB, MDH1, MDH2, ME1, PDHA1, PDHB, PKM2
630	Glyoxylate and dicarboxylate metabolism	7	29.16667	4.66E-04	ACAT1, ACO2, CAT, CS, MDH1, MDH2, PGP
640	Propanoate metabolism	11	34.375	9.05E-07	ACADM, ACAT1, ALDH2, ALDH6A1, ECHS1, HADHA, LDHA, LDHB, SUCLA2, SUCLG1, SUCLG2
650	Butanoate metabolism	8	27.58621	2.25E-04	ACADS, ACAT1, BDH2, ECHS1, HADH, HADHA, PDHA1, PDHB
910	Nitrogen metabolism	5	18.51852	2.59E-02	CA2, CA3, CYC1, ETFA, ETFB
3050	Proteasome	6	13.63636	4.66E-02	PSMA5, PSMA6, PSMA8, PSMB1, PSMB4, PSMB6
4020	Calcium signaling pathway	17	9.289618	1.53E-02	ATP2A1, ATP2A2, CALML3, CAMK2A, CAMK2D, CAMK2G, MYLK2, PHKA1, PHKB, PRKACB, RYR1, SLC25A4, TNNC1, TNNC2, VDAC1, VDAC2, VDAC3
4066	HIF-1 signaling pathway	11	10.37736	2.90E-02	ALDOA, CAMK2A, CAMK2D, CAMK2G, ENO1, ENO3, GAPDH, LDHA, PDHA1, PDHB, PGK1
4114	Oocyte meiosis	12	10.71429	1.84E-02	ADCY5, ANAPC1, CALML3, CAMK2A, CAMK2D, CAMK2G, PPP2R1B, PRKACB, YWHAB, YWHAE, YWHAG, YWHAZ
4260	Cardiac muscle contraction	18	23.37662	6.68E-08	ATP1A1, ATP2A2, COX5A, COX6B1, COX6C, COX7A2, CYC1, MYH6, MYH7, MYL2, MYL3, TNNC1, TPM1, TPM2, TPM3, UQCRB, UQCRC1, UQCRC2
4510	Focal adhesion	24	11.65049	1.19E-04	ACTG1, ACTN2, ACTN3, ACTN4, CAV1, COL11A1, COL1A1, COL3A1, COL4A1, COL4A2, COL6A1, COL6A2, COL6A3, FLNC, LAMA2, LAMB1, LAMB2, LAMC1, MYL2, MYL9, MYLK2, MYLPF, RAP1A, VCL
4512	ECM-receptor interaction	13	14.94253	7.79E-04	COL11A1, COL1A1, COL3A1, COL4A1, COL4A2, COL6A1, COL6A2, COL6A3, HSPG2, LAMA2, LAMB1, LAMB2, LAMC1
4530	Tight junction	18	13.43284	2.10E-04	ACTG1, ACTN2, ACTN3, ACTN4, CSDA, MYH1, MYH13, MYH2, MYH3, MYH4, MYH6, MYH7, MYH8, MYH9, MYL2, MYL9, MYLPF, PPP2R1B
4971	Gastric acid secretion	10	13.33333	9.23E-03	ADCY5, ATP1A1, CA2, CALML3, CAMK2A, CAMK2D, CAMK2G, MYLK2, PRKACB, SLC4A2
4974	Protein digestion and absorption	12	13.63636	3.11E-03	ATP1A1, COL11A1, COL12A1, COL14A1, COL1A1, COL3A1, COL4A1, COL4A2, COL6A1, COL6A2, COL6A3, PRSS3
5010	Alzheimer's disease	39	22.94118	1.80E-16	ATP2A1, ATP2A2, ATP5A1, ATP5B, ATP5C1, ATP5F1, ATP5H, ATP5O, CALML3, COX5A, COX6B1, COX6C, COX7A2, CYC1, CYCS, GAPDH, HSD17B10, NDUFA11, NDUFA13, NDUFA4, NDUFA5, NDUFA8, NDUFA9, NDUFAB1, NDUFB10, NDUFB8, NDUFS1, NDUFS2, NDUFS3, NDUFS4, NDUFS6, NDUFS7, NDUFV1, NDUFV2, SDHA, SDHB, UQCRB, UQCRC1, UQCRC2
5012	Parkinson's disease	41	31.29771	1.65E-22	ATP5A1, ATP5B, ATP5C1, ATP5F1, ATP5H, ATP5O, COX5A, COX6B1, COX6C, COX7A2, CYC1, CYCS, NDUFA11, NDUFA13, NDUFA4, NDUFA5, NDUFA8, NDUFA9, NDUFAB1, NDUFB10, NDUFB8, NDUFS1, NDUFS2, NDUFS3, NDUFS4, NDUFS6, NDUFS7, NDUFV1, NDUFV2, PARK7, SDHA, SDHB, SLC25A4, UBA1, UCHL1, UQCRB, UQCRC1, UQCRC2, VDAC1, VDAC2, VDAC3
5016	Huntington's disease	39	21.31148	1.65E-15	ATP5A1, ATP5B, ATP5C1, ATP5F1, ATP5H, ATP5O, COX5A, COX6B1, COX6C, COX7A2, CYC1, CYCS, NDUFA11, NDUFA13, NDUFA4, NDUFA5, NDUFA8, NDUFA9, NDUFAB1, NDUFB10, NDUFB8, NDUFS1, NDUFS2, NDUFS3, NDUFS4, NDUFS6, NDUFS7, NDUFV1, NDUFV2, SDHA, SDHB, SLC25A4, SOD2, UQCRB, UQCRC1, UQCRC2, VDAC1, VDAC2, VDAC3
5134	Legionellosis	10	18.18182	8.84E-04	ARF1, C3, CYCS, EEF1A2, EEF1G, HSPA1A, HSPA8, HSPD1, RAB1A, VCP
5146	Amoebiasis	15	13.76147	6.47E-04	ACTN2, ACTN3, ACTN4, COL11A1, COL1A1, COL3A1, COL4A1, COL4A2, HSPB1, LAMA2, LAMB1, LAMB2, LAMC1, PRKACB, VCL
5410	Hypertrophic cardiomyopathy (HCM)	15	17.64706	4.08E-05	ACTG1, ATP2A2, DES, DMD, LAMA2, LMNA, MYH6, MYH7, MYL2, MYL3, TNNC1, TPM1, TPM2, TPM3, TTN
5412	Arrhythmogenic right ventricular cardiomyopathy (ARVC)	9	12	2.52E-02	ACTG1, ACTN2, ACTN3, ACTN4, ATP2A2, DES, DMD, LAMA2, LMNA
5414	Dilated cardiomyopathy	17	18.68132	4.58E-06	ACTG1, ADCY5, ATP2A2, DES, DMD, LAMA2, LMNA, MYH6, MYH7, MYL2, MYL3, PRKACB, TNNC1, TPM1, TPM2, TPM3, TTN
5416	Viral myocarditis	14	18.91892	3.95E-05	ACTG1, CAV1, CYCS, DMD, LAMA2, MYH1, MYH13, MYH2, MYH3, MYH4, MYH6, MYH7, MYH8, MYH9

Regulated Proteins

KEGG ID	GOTerm	Nr. Genes	ssociated G	Term PValue	Associated Genes Found
5010	Alzheimer's disease	9	23.07692	0.994	ATP2A1, ATP5A1, ATP5B, COX7A2, NDUFA4, NDUFB10, NDUFS2, NDUFV2, SDHB
5012	Parkinson's disease	9	21.95122	0.909	ATP5A1, ATP5B, COX7A2, NDUFA4, NDUFB10, NDUFS2, NDUFV2, SDHB, VDAC3
5016	Huntington's disease	9	23.07692	0.998	ATP5A1, ATP5B, COX7A2, NDUFA4, NDUFB10, NDUFS2, NDUFV2, SDHB, VDAC3
	Citrate cycle (TCA cycle)	8	40	0.004	CS, FH, IDH3G, MDH2, OGDH, PDHA1, PDHB, SDHB
20					
190	Oxidative phosphorylation	8	23.52941	0.962	ATP5A1, ATP5B, COX7A2, NDUFA4, NDUFB10, NDUFS2, NDUFV2, SDHB
	Protein digestion and absorption	6	50	0.866	COL11A1, COL12A1, COL14A1, COL6A1, COL6A2, COL6A3
4974					
4151	PI3K-Akt signaling pathway	5	22.72727	1	COL11A1, COL6A1, COL6A2, COL6A3, YWHAG
4510	Focal adhesion	5	20.83333	1	ACTN2, COL11A1, COL6A1, COL6A2, COL6A3
4512	ECM-receptor interaction	5	38.46154	0.937	COL11A1, COL6A1, COL6A2, COL6A3, HSPG2
4020	Calcium signaling pathway	4	23.52941	1	ATP2A1, RYR1, TNNC2, VDAC3
10	Glycolysis / Gluconeogenesis	3	14.28571	0.963	GPI, PDHA1, PDHB
620	Pyruvate metabolism	3	20	0.762	MDH2, PDHA1, PDHB
630	Glyoxylate and dicarboxylate metabolism	3	42.85714	0.391	CAT, CS, MDH2
5203	Viral carcinogenesis	3	20	1	ACTN2, VDAC3, YWHAG
380	Tryptophan metabolism	2	28.57143	0.908	CAT, OGDH
650	Butanoate metabolism	2	25	0.776	PDHA1, PDHB
4066	HIF-1 signaling pathway	2	18.18182	1	PDHA1, PDHB
4713	Circadian entrainment	2	22.22222	1	PER3, RYR1
4972	Pancreatic secretion	2	25	1	ATP2A1, SLC4A2
5146	Amoebiasis	2	13.33333	1	ACTN2, COL11A1
5161	Hepatitis B	2	33.33333	1	HSPG2, VDAC3
5322	Systemic lupus erythematosus	2	25	1	ACTN2, HIST1H2AB

Identified Transcripts

KEGG ID	GOTerm	Nr. Genes	ssociated G	Term PValue (adj.*)	Associated Genes Found
20	Citrate cycle (TCA cycle)	15	50.00	4.92E-02	ACO1, ACO2, CS, DLAT, DLD, DLST, IDH1, IDH3A, OGDH, PC, SDHA, SDHB, SDHC, SUCLA2, SUCLG1
30	Pentose phosphate pathway	15	51.72	3.88E-02	ALDOA, DERA, FBP2, GPI, H6PD, PFKM, PFKP, PGD, PGM1, PGM2, PRPS2, RBKS, RGN, TALDO1, TKT
71	Fatty acid metabolism	21	47.73	3.00E-02	ACAA1, ACAA2, ACADS, ACADSB, ACADVL, ACOX1, ACSBG1, ACSL1, ACSL3, ACSL4, ADH1A, ADH5, ALDH2, ALDH3A2, ALDH9A1, CPT2, ECHS1, ECI2, GCDH, HADHA, HADHB
130	Ubiquinone and other terpenoid-quinone biosynthesis	7	70.00	4.58E-02	COQ2, COQ3, COQ5, COQ6, COQ7, TAT, VKORC1
250	Alanine, aspartate and glutamate metabolism	19	59.38	4.48E-03	ABAT, ADSS, ADSSL1, AGXT2, ALDH4A1, ALDH5A1, ASL, ASNS, ASPA, ASS1, CAD, GLS, GLUD1, GLUL, GOT1, GPT, GPT2, NIT2, PPAT
280	Valine, leucine and isoleucine degradation	21	47.73	3.00E-02	ABAT, ACAA1, ACAA2, ACAD8, ACADS, ACADSB, ALDH2, ALDH3A2, ALDH6A1, ALDH9A1, BCKDHB, DBT, DLD, ECHS1, HADHA, HADHB, HIBADH, HIBCH, MCCC1, MCEE, PCCB
310	Lysine degradation	25	51.02	6.68E-03	ALDH2, ALDH3A2, ALDH9A1, ASH1L, DLST, DOT1L, ECHS1, EHMT1, EHMT2, GCDH, HADHA, MLL, MLL2, MLL3, OGDH, PLOD1, PLOD2, PLOD3, SETD1B, SETD2, SETD7, SETDB2, SUV420H1, TMLHE, WHSC1
1040	Biosynthesis of unsaturated fatty acids	12	57.14	3.40E-02	ACAA1, ACOT1, ACOT2, ACOT4, ACOX1, BAAT, FADS1, HADHA, HSD17B12, PECR, SCD, SCD5
3013	RNA transport	61	36.97	3.52E-02	ACIN1, ALYREF, CASC3, CYFIP1, DDX20, DDX39B, EIF2B3, EIF2B5, EIF2S1, EIF3A, EIF3B, EIF3D, EIF3E, EIF3H, EIF4E, EIF4EBP1, EIF4G1, FXR2, GEMIN2, GEMIN4, GEMIN5, KPNB1, NMD3, NUP107, NUP133, NUP160, NUP188, NUP210, NUP214, NUP50, NUP62, NUP85, NUP93, NUP98, NXT1, PABPC1L, PABPC4, PAIP1, PHAX, POM121C, POP5, RANBP2, RPP21, RPP30, RPP38, SAP18, SENP2, SMN1, SRRM1, STRAP, SUMO2, SUMO3, TACC3, TGS1, THOC3, TPR, UBE2I, UPF2, UPF3B, XPO1, XPO5
3015	mRNA surveillance pathway	37	40.22	3.88E-02	ACIN1, ALYREF, BCL2L2-PABPN1, CASC3, CPSF6, CPSF7, CSTF1, CSTF2, DDX39B, ETF1, FIP1L1, GSPT1, GSPT2, NXT1, PABPC1L, PABPC4, PCF11, PPP1CA, PPP1CB, PPP2R1A, PPP2R2A, PPP2R3A, PPP2R5B, PPP2R5C, PPP2R5D, RNMT, SAP18, SMG1, SMG5, SMG6, SMG7, SRRM1, SSU72, SYMPK, UPF2, UPF3B, WDR33
3040	Spliceosome	59	44.70	8.43E-04	ACIN1, ALYREF, AQR, BCAS2, CCDC12, CDC40, CDC5L, CRNKL1, CTNNBL1, DDX39B, DDX46, DDX5, DHX15, DHX16, EFTUD2, HNRNPA1, HNRNPK, HSPA1B, HSPA8, LSM2, LSM6, PCBP1, PLRG1, PPIE, PQBP1, PRPF31, PRPF38B, PRPF4, PRPF40A, PRPF40B, PRPF6, PRPF8, RBM17, RBM22, RBM25, RBMX, SF3A1, SF3A2, SF3B4, SF3B5, SMNDC1, SNRNP200, SNRNP27, SNRNP70, SNRPA1, SNRPB, SNRPC, SNRPE, SNW1, SRSF1, SRSF10, SRSF4, SRSF6, THOC3, TRA2A, TRA2B, U2SURP, USP39, WBP11
3050	Proteasome	24	54.55	4.56E-03	PSMA1, PSMA2, PSMA3, PSMA4, PSMA5, PSMA6, PSMB2, PSMB3, PSMB5, PSMB7, PSMB8, PSMB9, PSMC2, PSMC4, PSMC6, PSMD1, PSMD11, PSMD2, PSMD4, PSMD8, PSME1, PSME4, PSMF1, SHFM1
4012	ErbB signaling pathway	35	39.77	4.92E-02	AKT1, AKT2, ARAF, BAD, BRAF, CAMK2A, CAMK2G, CRKL, EGF, EGFR, EIF4EBP1, ELK1, ERBB2, GAB1, GRB2, GSK3B, HBEGF, JUN, MAP2K2, MAPK1, MTOR, NCK1, NCK2, NRG2, NRG3, PAK1, PAK4, PIK3CB, PIK3R2, PIK3R3, PLCG1, PRKCA, RPS6KB1, RPS6KB2, SHC1
4066	HIF-1 signaling pathway	44	41.51	1.56E-02	AKT1, AKT2, ALDOA, ANGPT4, ARNT, CAMK2A, CAMK2G, CREBBP, CUL2, EGF, EGFR, EGLN1, EIF4E, EIF4EBP1, ENO1, ENO3, EP300, ERBB2, FLT1, GAPDH, HIF1A, HMOX1, IGF1R, INSR, LTBR, MAP2K2, MAPK1, MKNK2, MTOR, NFKB1, NOS3, PFKFB2, PFKFB3, PIK3CB, PIK3R2, PIK3R3, PLCG1, PRKCA, RELA, RPS6, RPS6KB1, RPS6KB2, TCEB2, TFRC
4120	Ubiquitin mediated proteolysis	61	44.20	6.91E-04	ANAPC1, ANAPC13, ANAPC2, ANAPC4, BIRC6, BTRC, CDC16, CDC20, CDC23, CUL1, CUL2, CUL3, CUL5, ERCC8, FBXW11, FBXW7, HERC1, HERC2, HERC3, HERC4, HUWE1, ITCH, KLHL13, KLHL9, NEDD4L, NHLRC1, PIAS2, PIAS3, PML, PPIL2, RHOBTB1, SAE1, SKP1, SMURF2, TCEB2, TRIM32, UBA1, UBA2, UBA3, UBA7, UBE2A, UBE2D1, UBE2D2, UBE2D3, UBE2E1, UBE2E3, UBE2F, UBE2G1, UBE2G2, UBE2H, UBE2I, UBE2M, UBE2N, UBE2O, UBE3B, UBE4A, UBE4B, UBR5, WWP1, WWP2, XIAP
4141	Protein processing in endoplasmic reticulum	74	44.31	2.93E-04	ATF4, ATF6, ATF6B, BAG2, BAK1, BCAP31, CAPN2, CRYAB, CUL1, DERL1, DNAJA1, DNAJA2, DNAJB1, DNAJB12, DNAJB2, DNAJC3, EDEM1, EDEM2, EIF2AK2, EIF2AK3, EIF2S1, ERLEC1, ERO1LB, ERP29, FBXO6, GANAB, HSP90AA1, HSP90B1, HSPA1B, HSPA5, HSPA8, HSPH1, HYOU1, LMAN1, MAN1A2, MAN1B1, NGLY1, OS9, P4HB, PLAA, PPP1R15A, PRKCSH, RAD23A, RNF185, RNF5, RPN1, RRBP1, SAR1A, SAR1B, SEC24C, SEC31A, SEC61A1, SEC61A2, SEC61G, SEC62, SEC63, SKP1, SSR3, SSR4, STT3A, TXNDC5, UBE2D1, UBE2D2, UBE2D3, UBE2E1, UBE2E3, UBE2G1, UBE2G2, UBE4B, UBQLN1, UBQLN2, UFD1L, UGGT1, WFS1
4142	Lysosome	51	41.80	7.81E-03	ABCA2, ACP2, AGA, AP1B1, AP1M1, AP3D1, AP3M1, AP3S2, AP4M1, ARSA, ASAH1, ATP6V0A1, ATP6V1H, CD164, CD63, CD68, CLN3, CLN5, CLTC, CLTCL1, CTSC, CTSD, CTSO, FUCA1, GAA, GGA1, GGA2, GGA3, GNPTAB, GUSB, HGSNAT, IDS, IGF2R, LAMP2, LGMN, LIPA, MAN2B1, MCOLN1, MFSD8, NAGA, NAPSA, NPC1, NPC2, PLA2G15, PPT2, SGSH, SLC11A1, SORT1, SUMF1, TCIRG1, TPP1
4144	Endocytosis	72	35.47	4.65E-02	ACAP3, ADRBK1, AGAP2, AGAP3, AP2A2, AP2M1, ARAP1, ARF6, ARFGAP2, ASAP2, ASAP3, CDC42, CHMP2A, CHMP4B, CHMP5, CHMP6, CLTC, CLTCL1, DAB2, DNM2, EEA1, EGF, EGFR, EPN1, EPS15, FAM125A, FLT1, GIT2, HGS, HSPA1B, HSPA8, IGF1R, IQSEC1, IQSEC2, ITCH, LDLRAP1, NEDD4L, NTRK1, PARD3, PDCD6IP, PLD2, PML, PRKCI, PSD, PSD4, RAB11A, RAB11B, RAB11FIP3, RAB11FIP5, RAB22A, RAB4A, RAB7A, RHOA, RUFY1, SH3GL1, SMAD2, SMAD7, SMAP1, SMURF2, STAMBP, TFRC, TGFB1, TGFB3, TGFBR2, TSG101, USP8, VPS36, VPS37B, VPS4A, VPS4B, WWP1, ZFYVE16
4146	Peroxisome	33	40.74	4.52E-02	ABCD3, ABCD4, ACAA1, ACOX1, ACSL1, ACSL3, ACSL4, AMACR, BAAT, CAT, CROT, DECR2, ECH1, ECI2, EPHX2, FAR1, GNPAT, HACL1, IDH1, PAOX, PECR, PEX1, PEX13, PEX14, PEX2, PEX26, PEX3, PEX5, PEX6, PMVK, PXMP4, SCP2, SLC25A17
4150	mTOR signaling pathway	30	50.00	4.20E-03	AKT1, AKT2, BRAF, CAB39L, EIF4E, EIF4EBP1, HIF1A, IKBKB, IRS1, MAPK1, MLST8, MTOR, PDPK1, PIK3CB, PIK3R2, PIK3R3, PRKAA2, PRKCA, RHEB, RPS6, RPS6KA3, RPS6KB1, RPS6KB2, RPTOR, RRAGB, STK11, STRADA, TSC1, ULK1, ULK2
4330	Notch signaling pathway	24	50.00	1.10E-02	ADAM17, APH1A, CREBBP, CTBP1, CTBP2, DLL4, DTX1, DTX3, DTX3L, DVL1, EP300, JAG1, JAG2, KAT2B, MAML1, NCOR2, NOTCH1, NOTCH2, NOTCH3, NOTCH4, NUMB, PSENEN, RFNG, SNW1
4510	Focal adhesion	84	40.78	6.59E-04	ACTN2, ACTN4, AKT1, AKT2, ARHGAP35, ARHGAP5, BAD, BCAR1, BRAF, CAPN2, CCND3, CDC42, COL11A1, COL11A2, COL1A1, COL1A2, COL27A1, COL2A1, COL3A1, COL4A4, COL4A5, COL5A1, COL5A2, COL5A3, COL6A1, COL6A2, COL6A6, CRKL, CTNNB1, DIAPH1, DOCK1, EGF, EGFR, ELK1, ERBB2, FLNA, FLNC, FLT1, FN1, FYN, GRB2, GSK3B, IGF1R, ILK, ITGA2B, ITGA6, ITGA7, ITGA9, ITGAV, ITGB4, ITGB5, ITGB6, ITGB7, JUN, LAMA3, LAMA4, LAMA5, LAMB2, MAPK1, MYLK, MYLK2, PAK1, PAK4, PDGFD, PDPK1, PIK3CB, PIK3R2, PIK3R3, PPP1CA, PPP1CB, PPP1R12A, PRKCA, PXN, RAP1A, RAPGEF1, RHOA, SHC1, THBS1, THBS2, TLN1, TNXB, VEGFC, XIAP, ZYX
4512	ECM-receptor interaction	39	44.83	8.00E-03	AGRN, CD36, CD44, CD47, COL11A1, COL11A2, COL1A1, COL1A2, COL27A1, COL2A1, COL3A1, COL4A4, COL4A5, COL5A1, COL5A2, COL5A3, COL6A1, COL6A2, COL6A6, DAG1, FN1, HSPG2, ITGA2B, ITGA6, ITGA7, ITGA9, ITGAV, ITGB4, ITGB5, ITGB6, ITGB7, LAMA3, LAMA4, LAMA5, LAMB2, SDC4, THBS1, THBS2, TNXB
4520	Adherens junction	32	43.84	2.03E-02	ACTN2, ACTN4, BAIAP2, CDC42, CREBBP, CTNNA1, CTNNB1, CTNND1, EGFR, EP300, ERBB2, FYN, IGF1R, INSR, IQGAP1, MAPK1, PARD3, PTPRB, PTPRM, PVRL2, RHOA, SMAD2, SMAD4, SORBS1, SSX2IP, TCF7L1, TCF7L2, TGFBR2, TJP1, WASF3, WASL, YES1
4710	Circadian rhythm	21	67.74	3.48E-04	BHLHE40, BHLHE41, BTRC, CRY1, CRY2, CSNK1D, CSNK1E, CUL1, FBXL3, FBXW11, PER1, PER2, PER3, PRKAA2, PRKAB2, PRKAG1, PRKAG2, PRKAG3, RORA, RORC, SKP1

4910	Insulin signaling pathway	55	39.29	1.58E-02	AKT1, AKT2, ARAF, BAD, BRAF, CALM3, CRKL, EIF4E, EIF4EBP1, ELK1, FASN, FBP2, FOXO1, G6PC3, GRB2, GSK3B, IKBKB, INPPL1, INSR, IRS1, IRS2, MAP2K2, MAPK1, MKNK2, MTOR, PDPK1, PHKG1, PHKG2, PIK3CB, PIK3R2, PIK3R3, PPP1CA, PPP1CB, PPP1R3A, PPP1R3C, PRKAA2, PRKAB2, PRKAG1, PRKAG2, PRKAG3, PRKAR1A, PRKCI, PYGB, PYGL, RAPGEF1, RHEB, RPS6, RPS6KB1, RPS6KB2, RPTOR, SHC1, SLC2A4, SOCS2, SORBS1, TSC1
4974	Protein digestion and absorption	36	40.91	3.64E-02	ATP1A1, ATP1A2, ATP1A3, ATP1B2, ATP1B3, ATP1B4, COL11A1, COL11A2, COL14A1, COL15A1, COL18A1, COL1A1, COL1A2, COL22A1, COL27A1, COL2A1, COL3A1, COL4A4, COL4A5, COL5A1, COL5A2, COL5A3, COL6A1, COL6A2, COL6A6, COL7A1, COL9A1, COL9A3, DPP4, ELN, MME, SLC16A10, SLC38A2, SLC3A2, SLC8A2, SLC8A3
5010	Alzheimer's disease	67	39.41	7.16E-03	ADAM10, ADAM17, APH1A, APP, ATF6, ATP2A1, ATP2A2, ATP5B, ATP5C1, ATP5G1, ATP5G2, ATP5H, ATP5J, ATP5O, BAD, CACNA1C, CACNA1S, CALM3, CAPN2, CASP7, CASP9, CDK5, COX6A2, COX7A2, COX7A2L, COX7B, COX8A, CYC1, EIF2AK3, FADD, GAPDH, GRIN2D, GSK3B, ITPR1, LPL, LRP1, MAPK1, MME, NAE1, NDUFA1, NDUFA10, NDUFA13, NDUFA2, NDUFA6, NDUFA8, NDUFA9, NDUFB10, NDUFB2, NDUFC1, NDUFC2, NDUFS2, NDUFS3, NDUFS7, NDUFS8, NDUFV2, NOS1, PLCB3, PLCB4, PPP3CA, PPP3R1, PSENEN, SDHA, SDHB, SDHC, UQCR10, UQCR11, UQCRFS1
5210	Colorectal cancer	28	45.16	2.05E-02	AKT1, AKT2, APC, APC2, ARAF, BAD, BRAF, CASP9, CTNNB1, FOS, GSK3B, JUN, MAPK1, MSH2, MSH3, MSH6, PIK3CB, PIK3R2, PIK3R3, RALGDS, RHOA, SMAD2, SMAD4, TCF7L1, TCF7L2, TGFB1, TGFB3, TGFBR2
5211	Renal cell carcinoma	31	46.97	8.40E-03	AKT1, AKT2, ARAF, ARNT, BRAF, CDC42, CREBBP, CRKL, CUL2, EGLN1, EP300, EPAS1, ETS1, FLCN, GAB1, GRB2, HIF1A, JUN, MAP2K2, MAPK1, PAK1, PAK4, PIK3CB, PIK3R2, PIK3R3, PTPN11, RAP1A, RAPGEF1, TCEB2, TGFB1, TGFB3
5212	Pancreatic cancer	29	43.94	2.73E-02	AKT1, AKT2, ARAF, BAD, BRAF, CASP9, CDC42, EGF, EGFR, ERBB2, IKBKB, IKBKG, JAK1, MAPK1, NFKB1, PIK3CB, PIK3R2, PIK3R3, RALB, RALBP1, RALGDS, RB1, RELA, SMAD2, SMAD4, STAT1, TGFB1, TGFB3, TGFBR2
5213	Endometrial cancer	25	48.08	1.56E-02	AKT1, AKT2, APC, APC2, ARAF, BAD, BRAF, CASP9, CTNNA1, CTNNB1, EGF, EGFR, ELK1, ERBB2, GRB2, GSK3B, ILK, MAP2K2, MAPK1, PDPK1, PIK3CB, PIK3R2, PIK3R3, TCF7L1, TCF7L2
5215	Prostate cancer	38	42.70	1.59E-02	AKT1, AKT2, AR, ARAF, ATF4, BAD, BRAF, CASP9, CREB3L3, CREB3L4, CREBBP, CTNNB1, EGF, EGFR, EP300, ERBB2, FOXO1, GRB2, GSK3B, GSTP1, HSP90AA1, HSP90B1, IGF1R, IKBKB, IKBKG, MAP2K2, MAPK1, MTOR, NFKB1, PDGFD, PDPK1, PIK3CB, PIK3R2, PIK3R3, RB1, RELA, TCF7L1, TCF7L2
5410	Hypertrophic cardiomyopathy (HCM)	36	42.35	2.15E-02	ACTC1, ATP2A2, CACNA1C, CACNA1S, CACNA2D1, CACNG1, CACNG6, DAG1, EMD, ITGA2B, ITGA6, ITGA7, ITGA9, ITGAV, ITGB4, ITGB5, ITGB6, ITGB7, LMNA, MYH7, MYL3, PRKAA2, PRKAB2, PRKAG1, PRKAG2, PRKAG3, SGCA, SGCD, TGFB1, TGFB3, TNNI3, TPM1, TPM2, TPM3, TPM4, TTN

Regulated Transcripts

KEGG ID	GOTerm	Nr. Genes	ssociated G	Term PValue	Associated Genes Found
51	Fructose and mannose metabolism	3	27.27273	3.30E-02	ALDOA, PFKFB2, PFKFB3
330	Arginine and proline metabolism	4	17.3913	2.52E-02	ALDH2, ALDH4A1, GLUL, SAT2
4066	HIF-1 signaling pathway	6	13.63636	1.75E-02	ALDOA, ANGPT4, HMOX1, PFKFB2, PFKFB3, TFRC
4115	p53 signaling pathway	4	44.44444	4.41E-02	GADD45A, GADD45G, SESN1, THBS1
4151	PI3K-Akt signaling pathway	14	13.59223	7.38E-03	ANGPT4, COL11A1, COL1A1, COL3A1, COL5A1, COL5A2, COL6A1, COL6A2, GRB2, IRS1, ITGA7, LAMB2, THBS1, TNXB
4510	Focal adhesion	12	14.28571	6.34E-04	COL11A1, COL1A1, COL3A1, COL5A1, COL5A2, COL6A1, COL6A2, GRB2, ITGA7, LAMB2, THBS1, TNXB
4512	ECM-receptor interaction	12	30.76923	8.79E-08	COL11A1, COL1A1, COL3A1, COL5A1, COL5A2, COL6A1, COL6A2, HSPG2, ITGA7, LAMB2, THBS1, TNXB
4910	Insulin signaling pathway	7	12.72727	1.97E-02	FASN, GRB2, IRS1, IRS2, PHKG1, PPP1R3C, SOCS2
4930	Type II diabetes mellitus	4	30.76923	1.42E-02	CACNA1C, IRS1, IRS2, SOCS2
4974	Protein digestion and absorption	12	33.33333	1.00E-07	COL11A1, COL14A1, COL15A1, COL18A1, COL1A1, COL3A1, COL5A1, COL5A2, COL6A1, COL6A2, COL9A1, ELN
5146	Amoebiasis	6	17.64706	1.98E-02	COL11A1, COL1A1, COL3A1, COL5A1, COL5A2, LAMB2
5410	Hypertrophic cardiomyopathy (HCM)	5	13.88889	2.53E-02	CACNA1C, ITGA7, SGCA, TNNI3, TPM3
5414	Dilated cardiomyopathy	5	13.88889	3.28E-02	CACNA1C, ITGA7, SGCA, TNNI3, TPM3

Regulated in Aging

KEGG ID	GOTerm	Nr. Genes	ssociated G	Term PValue (adj.*)	Associated Genes Found
10	Glycolysis / Gluconeogenesis	5	8.474576	7.59E-03	DLAT, DLD, PDHA1, PDHB, TPI1
20	Citrate cycle (TCA cycle)	10	33.33333	2.24E-11	ACO2, DLAT, DLD, IDH2, MDH2, PDHA1, PDHB, SDHB, SDHC, SUCLA2
190	Oxidative phosphorylation	18	17.30769	5.11E-13	ATP5B, ATP5G3, ATP5J, ATP5L, COX7B, COX7C, NDUFA10, NDUFB4, NDUFS1, NDUFS3, NDUFV2, PPA1, SDHB, SDHC, UQCRB, UQCRC2, UQCRFS1, UQCRH
250	Alanine, aspartate and glutamate metabolism	3	11.11111	3.13E-02	GLUD1, GOT1, GOT2
260	Glycine, serine and threonine metabolism	4	12.5	6.98E-03	ALAS1, CTH, DLD, GRHPR
270	Cysteine and methionine metabolism	3	9.375	3.66E-02	CTH, GOT1, GOT2
330	Arginine and proline metabolism	4	8.510638	2.38E-02	CKMT2, GLUD1, GOT1, GOT2
400	Phenylalanine, tyrosine and tryptophan biosynthesis	2	50	9.13E-03	GOT1, GOT2
620	Pyruvate metabolism	7	20	6.17E-06	DLAT, DLD, GRHPR, MDH2, ME2, PDHA1, PDHB
630	Glyoxylate and dicarboxylate metabolism	3	15	1.61E-02	ACO2, GRHPR, MDH2
910	Nitrogen metabolism	3	12.5	2.14E-02	CA4, GLUD1, UQCRFS1
3010	Ribosome	8	6.956522	1.99E-03	MRPL13, MRPL15, MRPL18, MRPL3, MRPL34, MRPS15, MRPS18A, MRPS7
3013	RNA transport	11	8.333333	4.70E-05	CASC3, EIF3A, EIF3B, EIF3J, EIF4E, NUP43, PNN, RBM8A, RNPS1, UBE2I, UPF2
3015	mRNA surveillance pathway	7	9.459459	1.14E-03	CASC3, CPSF7, PNN, PPP2R5C, RBM8A, RNPS1, UPF2
3040	Spliceosome	11	10	6.85E-06	DDX42, HNRNPC, HNRNPM, HSPA8, PRPF31, RBM8A, SF3B2, SNRNP200, SNRNP40, SNRNP70, SRSF5
4066	HIF-1 signaling pathway	7	6.730769	2.26E-03	CDKN1A, EIF4E, PDHA1, PDHB, PIK3R1, TFRC, VEGFA
4260	Cardiac muscle contraction	6	9.523809	2.38E-03	COX7B, COX7C, UQCRB, UQCRC2, UQCRFS1, UQCRH
4370	VEGF signaling pathway	4	7.272728	3.08E-02	NFATC1, PIK3R1, PLA2G4C, VEGFA
4964	Proximal tubule bicarbonate reclamation	3	13.63636	1.51E-02	AQP1, CA4, GLUD1
5010	Alzheimer's disease	17	11.4094	2.59E-10	ATP5B, ATP5G3, ATP5J, COX7B, COX7C, CYCS, NDUFA10, NDUFB4, NDUFS1, NDUFS3, NDUFV2, SDHB, SDHC, UQCRB, UQCRC2, UQCRFS1, UQCRH
5012	Parkinson's disease	19	18.4466	7.25E-14	ATP5B, ATP5G3, ATP5J, COX7B, COX7C, CYCS, NDUFA10, NDUFB4, NDUFS1, NDUFS3, NDUFV2, PPIF, SDHB, SDHC, SLC25A4, UQCRB, UQCRC2, UQCRFS1, UQCRH
5016	Huntington's disease	21	13.3758	1.27E-13	ATP5B, ATP5G3, ATP5J, CLTA, COX7B, COX7C, CYCS, NDUFA10, NDUFB4, NDUFS1, NDUFS3, NDUFV2, POLR2K, PPIF, SDHB, SDHC, SLC25A4, UQCRB, UQCRC2, UQCRFS1, UQCRH

Guilty by Association

KEGG ID	GOTerm	Nr. Genes	ssociated G	Term PValue (adj*)	Associated Genes Found
5205	Proteoglycans in cancer	26	11.45	2.13E-03	ANK2, ANK3, CAMK2B, CAMK2D, CAV2, CD63, COL21A1, CTSL1, DCN, FGF12, FGFR1, FZD4, GPC1, HSPB2, IGF1, ITGB1, LUM, MRAS, PPP1R12B, PRKACA, PXN, RRAS, SDC4, STAT3, TIMP3, TWIST1
5414	Dilated cardiomyopathy	26	28.57	6.15E-11	ACTC1, ADCY4, ADCY9, ATP2A2, DAG1, DES, DMD, IGF1, ITGA7, ITGA9, ITGB1, LAMA2, MYBPC3, MYL2, MYL3, PLN, PRKACA, SGCA, SGCB, SGCD, SGCG, TNNC1, TNNT2, TPM1, TPM2, TTN
4510	Focal adhesion	23	11.17	5.83E-03	ACTN2, BCAR1, CAV2, COL5A3, FIGF, IGF1, ILK, ITGA7, ITGA9, ITGB1, LAMA2, LAMA5, LAMB2, MYL12A, MYL2, MYL9, PARVA, PDGFD, PDGFRB, PPP1R12B, PXN, TNXB, VEGFB
5410	Hypertrophic cardiomyopathy (HCM)	23	27.06	2.56E-09	ACTC1, ATP2A2, DAG1, DES, DMD, IGF1, ITGA7, ITGA9, ITGB1, LAMA2, MYBPC3, MYL2, MYL3, PRKAG2, SGCA, SGCB, SGCD, SGCG, TNNC1, TNNT2, TPM1, TPM2, TTN
4810	Regulation of actin cytoskeleton	22	10.23	1.69E-02	ACTN2, BCAR1, CFL2, CHRM2, FGF12, FGFR1, GSN, ITGA7, ITGA9, ITGB1, MRAS, MYH14, MYL12A, MYL2, MYL9, NCKAP1, PDGFD, PDGFRB, PPP1R12B, PXN, RRAS, WASL
3320	PPAR signaling pathway	17	23.94	3.77E-06	ACOX1, ACOX2, ACSL1, ADIPOQ, AQP7, CD36, FABP4, ILK, LPL, PCK1, PLTP, PPARA, RXRA, SCP2, SLC27A1, SORBS1, UBC
4910	Insulin signaling pathway	17	12.14	1.09E-02	ACACB, GYS1, INPP5K, INSR, IRS2, PCK1, PHKA1, PPARGC1A, PPP1R3B, PPP1R3C, PRKACA, PRKAG2, PRKAR1A, PYGM, RHOQ, SLC2A4, SORBS1
4142	Lysosome	15	12.3	1.67E-02	ASAH1, CD63, CLTB, CTSD, CTSF, CTSK, CTSL1, CTSO, CTSZ, HYAL1, LAPTM4A, NAGLU, PSAP, SCARB2, SORT1
4260	Cardiac muscle contraction	14	18.18	7.94E-04	ACTC1, ATP1A2, ATP2A2, COX5B, COX6A2, COX7A1, MYL2, MYL3, TNNC1, TNNT2, TPM1, TPM2, UQCRB, UQCRC1
4270	Vascular smooth muscle contraction	14	11.67	2.85E-02	[ADCY4, ADCY9, AGTR1, CALCRL, EDNRA, GUCY1A3, MYL9, NPR1, PLA2G5, PPP1R12B, PRKACA, PRKG1, RAMP2, RAMP3]
4670	Leukocyte transendothelial migration	14	11.86	2.67E-02	[ACTN2, BCAR1, CDH5, CLDN5, CTNNA3, CXCL12, ESAM, ITGB1, JAM2, MYL12A, MYL2, MYL9, PXN, RAPGEF3]
5412	Arrhythmogenic right ventricular cardiomyopathy (ARVC)	14	18.67	6.66E-04	[ACTN2, ATP2A2, CTNNA3, DAG1, DES, DMD, ITGA7, ITGA9, ITGB1, LAMA2, SGCA, SGCB, SGCD, SGCG]
640	Propanoate metabolism	12	37.5	1.76E-06	[ACACB, ACAT1, ACSS2, ACSS3, ALDH2, ALDH3A2, ALDH6A1, HADHA, MLYCD, PCCA, SUCLG1, SUCLG2]
4920	Adipocytokine signaling pathway	12	16.9	3.90E-03	[ACACB, ACSL1, ADIPOQ, CD36, IRS2, PCK1, PPARA, PPARGC1A, PRKAG2, RXRA, SLC2A4, STAT3]
71	Fatty acid metabolism	11	25	3.82E-04	[ACAA2, ACADVL, ACAT1, ACOX1, ACSL1, ADH1B, ADH5, ALDH2, ALDH3A2, HADHA, HADHB]
4512	ECM-receptor interaction	11	12.64	3.85E-02	[CD36, COL5A3, DAG1, ITGA7, ITGA9, ITGB1, LAMA2, LAMA5, LAMB2, SDC4, TNXB]
280	Valine, leucine and isoleucine degradation	10	22.73	1.30E-03	[ACAA2, ACAT1, ALDH2, ALDH3A2, ALDH6A1, AUH, HADHA, HADHB, HIBADH, PCCA]
4610	Complement and coagulation cascades	10	14.49	2.49E-02	[A2M, C1QA, C1QB, C1QC, C1R, C1S, CFH, F8, MASP1, SERPING1]
10	Glycolysis / Gluconeogenesis	9	13.64	4.60E-02	[ACSS2, ADH1B, ADH5, ALDH2, ALDH3A2, ENO3, PCK1, PFKM, PGM1]
20	Citrate cycle (TCA cycle)	9	30	4.08E-04	[ACO1, ACO2, CS, DLST, OGDH, PCK1, SDHA, SUCLG1, SUCLG2]
620	Pyruvate metabolism	9	21.95	3.26E-03	[ACACB, ACAT1, ACSS2, ACYP2, ALDH2, ALDH3A2, LDHD, ME3, PCK1]
380	Tryptophan metabolism	8	20	1.12E-02	[ACAT1, ALDH2, ALDH3A2, HADHA, INMT, MAOA, MAOB, OGDH]
410	beta-Alanine metabolism	6	20.7	2.60E-02	[ALDH2, ALDH3A2, ALDH6A1, AOC3, HADHA, MLYCD]
630	Glyoxylate and dicarboxylate metabolism	6	25	1.27E-02	[ACAT1, ACO1, ACO2, CS, GLUL, PCCA]
62	Fatty acid elongation	5	21.74	4.01E-02	[ACAA2, ACOT1, HADHA, HADHB, PTPLA]
360	Phenylalanine metabolism	5	27.78	1.76E-02	[AOC3, GOT1, MAOA, MAOB, PRDX6]
